# Catalysis enabled synthesis, structures, and reactivities of fluorinated S_8_-corona[*n*]arenes (*n* = 8–12)†

**DOI:** 10.1039/d2sc05348a

**Published:** 2022-11-16

**Authors:** Andrew. T. Turley, Magnus W. D. Hanson-Heine, Stephen. P. Argent, Yaoyang Hu, Thomas. A. Jones, Michael Fay, Simon Woodward

**Affiliations:** GSK Carbon Neutral Laboratories for Sustainable Chemistry, University of Nottingham, Jubilee Campus Nottingham NG7 2TU UK andrew.turley@nottingham.ac.uk simon.woodward@nottingham.ac.uk; School of Chemistry, University of Nottingham, University Park Campus Nottingham NG7 2RD UK; Nanoscale and Microscale Research Centre, University of Nottingham, University Park Campus Cripps South Building Nottingham NG7 2RD UK

## Abstract

Previously inaccessible large S_8_-corona[*n*]arene macrocycles (*n* = 8–12) with alternating aryl and 1,4-C_6_F_4_ subunits are easily prepared on up to gram scales, without the need for chromatography (up to 45% yield, 10 different examples) through new high acceleration S_N_Ar substitution protocols (catalytic NR_4_F in pyridine, R = H, Me, Bu). Macrocycle size and functionality are tunable by precursor and catalyst selection. Equivalent simple NR_4_F catalysis allows facile late-stage S_N_Ar difunctionalisation of the ring C_6_F_4_ units with thiols (8 derivatives, typically 95+% yields) providing two-step access to highly functionalised fluoromacrocycle libraries. Macrocycle host binding supports fluoroaryl catalytic activation through contact ion pair binding of NR_4_F and solvent inclusion. In the solid-state, solvent inclusion also intimately controls macrocycle conformation and fluorine–fluorine interactions leading to spontaneous self-assembly into infinite columns with honeycomb-like lattices.

## Abstract 2

NR_4_F salts simultaneously catalyse and template the reaction of dithiols with C_6_F_6_ to generate the first fluorinated corona[*n*]arenes. Simple S_N_Ar functionalisation of the fluoraryl units easily afford diverse libraries of macrocyclic building blocks.

## Graphical abstract

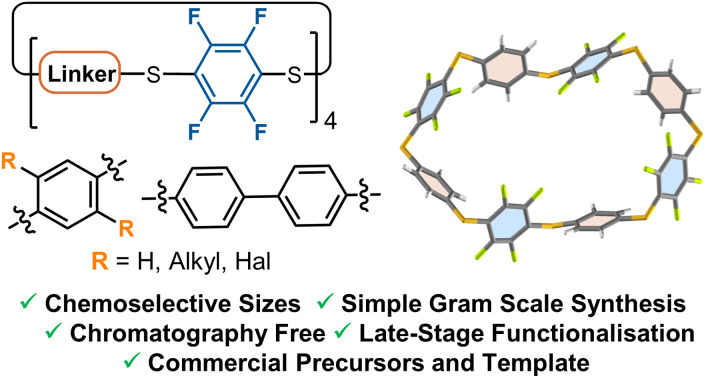

## Introduction

**Fig. 1:**
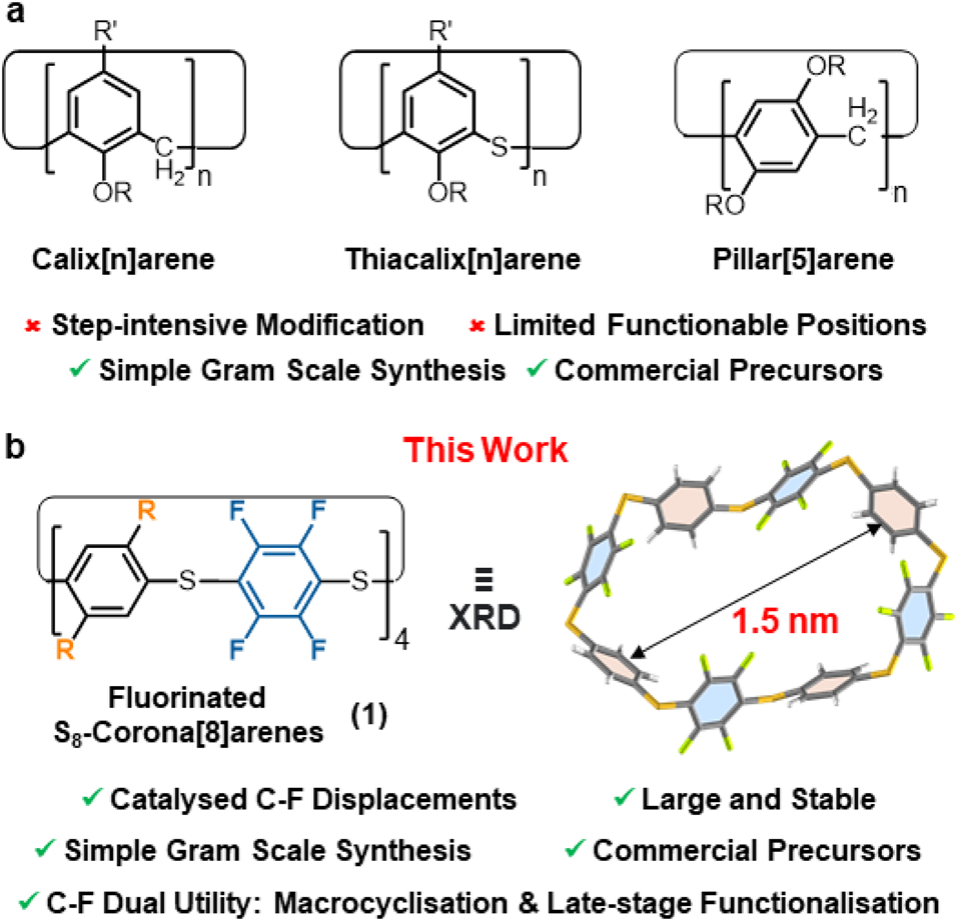
(a) Widely used, but harder to functionalise, arene macrocycles. (b) A typical S_8_-corona[8]arene (1a, R = H) with catalyst labile C–F motifs for new one-step synthesis and late-stage catalytic functionalisation.

Accessing functionalised macrocyclic molecules with well-defined cavities is a major focus in supramolecular chemistry due to their synthetic challenge, unique shapes, and selective guest binding abilities.^1–3^ Such characteristics are vital in chemical sensing,^4,5^ drug delivery,^6^ self-healing materials,^7^ catalysis,^8^ and molecular electronic applications,^9^ and more generally, when (self)assembling any complex supramolecular architecture.^10^ Outside cycloamides and cyclodextrins, easily accessed macrocycles capable of single-step functionalisation are remarkably rare.^2^ For example, while aryl-rich macrocycles abound, *e.g.* pillar[*n*]arenes,^11–13^ calix[*n*]arenes,^14–17^ and corona[*n*]arenes;^18,19^ installing suitable functional handles within these is typically time-consuming and problematic (Fig. 1a) and this prevents rapid access to onward functionality diversification.^20–24^ For such cases derivatisation is required, leading to longer synthetic routes, reduced overall efficiency and time-consuming chromatographic separations of multiple intermediates. Potentially, catalytic macrocyclisation of commercial C_6_F_6_ (or a simple analogue thereof) offers an elegant solution to such problems, as both the macrocyclisation and subsequent derivatisation can potentially be achieved by simple C–F displacements in single-step reactions. The resultant novel fluorinated macrocycles would offer significant advantages over their non-fluorinated forbearers.^25^

Herein, we report one-pot catalytic protocols using C_6_F_6_ (and analogues) to form the robust, fluorinated macrocycle S_8_-corona[8]arene 1a (R = H) and its derivatives (Fig. 1b), whereby the C–F bonds are rapidly displaced in new templated catalytic macrocyclisation approaches and late-stage functionalisations. Highly catalyst accelerated S_N_Ar substitutions of perfluoroaryl units enable these new mechanism driven protocols.‡‡Catalysed S_N_Ar substitutions using simple acyclic models of the sub-units within 1 also complete within minutes (compared to hours for the control uncatalysed reactions). Thus, the rate acceleration observed for NR_4_F/pyridine is in addition to any global ring templating effects (ESI Fig. S215†).

The aryl macrocycles (1) are prepared from low cost materials on up to gram scales in 1–3 steps without any special conditions. Purification is achieved without the need for chromatographic separations or recrystallisation. Instead, all the macrocycles reported here are isolated simply by filtering off any insoluble by-products formed. To the best of our knowledge, this is the first report of readily accessible corona[*n*]arenes with *n* > 6,^19^ and the first to realise one-step aryl functionalisation. The reactions are general and libraries of S_8_-corona[*n*]arene (1) derivatives with varying symmetries and substitution patterns are readily attained. Catalytic functionalisation of the derived S_8_-corona[*n*]arenes (1) themselves with thiols is also rapid at room temperature, allowing further highly diverse functional fluorinated macrocycles to be attained in just two steps. Additionally, the solvent and ammonium guest binding behaviour of 1 is in accord with the new NR_4_F/pyridine C–F activation protocol proposed and demonstrates the wide potential of S_8_-corona[*n*]arenes (1) in host-guest applications.

## Results and discussion

**Scheme 1:**
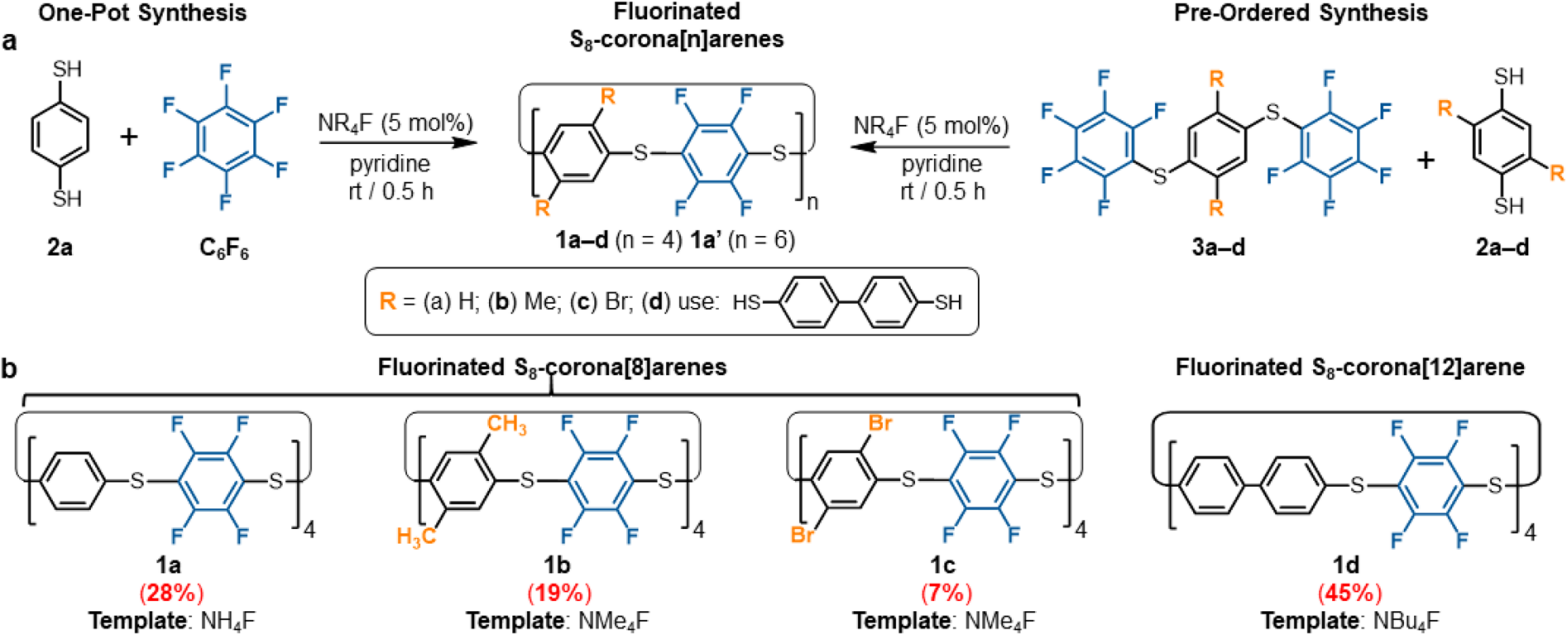
(a) Two routes to synthesise fluorinated S_8_-corona[*n*]arenes (1): from C_6_F_6_ and from analogues (3). (b) Schematic representations of macrocycles (1a–d) synthesised from the pre-ordered route, indicating the optimal NR_4_F catalytic template.

Macrocyclisation of C_6_F_6_ in the presence of dinucleophiles is inordinately challenging due to competing linear oligomerisation. Previous attempts have ended only in polymer production.^26^ Our own initial trials, using commercial C_6_F_6_ and benzene-1,4-dithiol (2a,§§Commercial 2a–d are also easily obtained at a low cost from the 2–3 step chromatography-free procedures outlined in the ESI.† The same is true for fragments 3a–d.Scheme 1a), under a variety of classical S_N_Ar conditions, also just provided an insoluble polymer (P1, ESI Fig. S221–S225† for polymer data). Thus, we sought a new S_N_Ar protocol offering both high rate S_N_Ar acceleration but also the templating needed to favour macrocycle (1) formation. Screening revealed ammonium fluoride catalysts in pyridine to be particularly effective‡ while more traditional S_N_Ar solvents vastly favoured polymerisation. When NH_4_F (AF), NMe_4_F (TMAF), and NBu_4_F (TBAF) are used as catalytic-base-templates in pyridine, the reaction between 2a and C_6_F_6_ immediately provides appreciable amounts of macrocycle 1a (Scheme 1a). To the best of our knowledge, this is the first example of use of ammonium fluorides as a dual-function catalytic-base-templates in one reaction. Catalyst loadings of 1 mol% give equivalent production of 1a and reactions at 0.1 mol% are still practical. Control studies with other NR_4_X (R = Me, Bu; X = Cl, Br, I) promotors, even in stoichiometric amounts, did not yield any 1a, only polymer (P1) and unreacted starting materials are observed by ^1^H and ^19^F NMR spectroscopy in these control reactions. DFT studies (ESI Fig. S216† for full calculation details) indicate an unusual C_6_F_6_⋯NMe_4_F ion pair association mode that increases the sp^3^ character of the fluoroaryl carbons (weaker C=C character). This is the likely origin of the remarkable S_N_Ar acceleration observed (ESI Fig. S213–S215† and data for kinetic simulation of 1a formation). This is in addition to the macrocyclisation effects of the same NR_4_F template-catalysts. The use of pyridine is important as this solvent particularly favours the formation of the necessary NR_4_F contact ion pairs.^27^

**Fig. 2:**
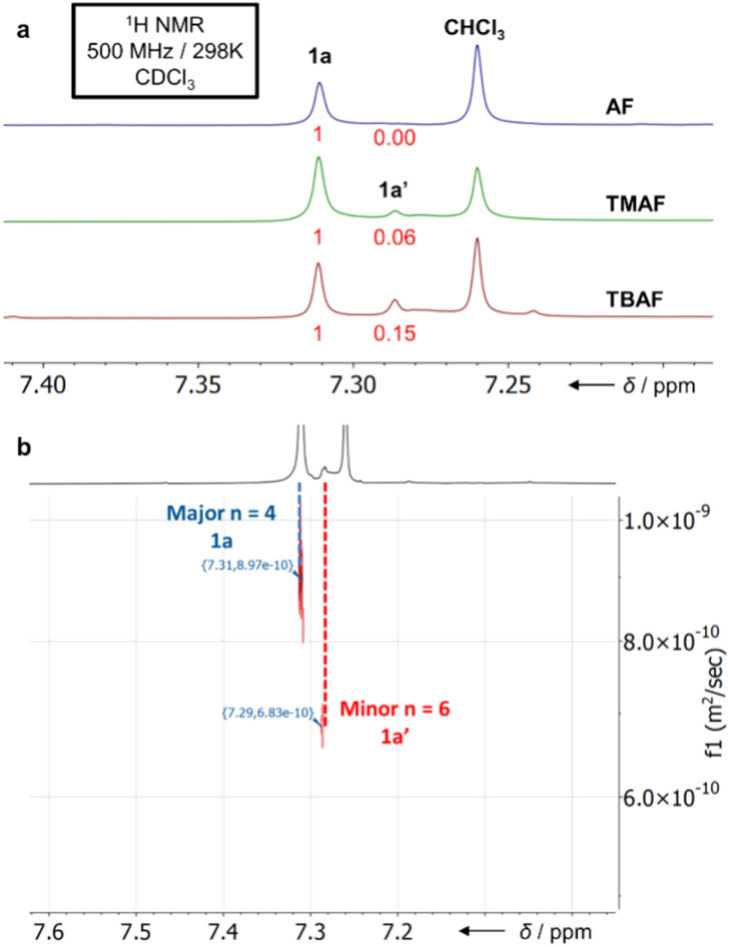
Partial (a) ^1^H NMR spectra (CDCl_3_) of S_8_-corona[8]arene (1a) and S_12_-corona[12]arene (1a′) as synthesised *via*3a promoted by different ammonium fluoride catalysts. (b) Diffusion-Ordered Spectroscopy (DOSY) NMR spectrum of a 1a : 1a′ mixture.

Optimisation provided a one-pot preparation of macrocycle 1a in moderate, but practical and scalable yields, through the 10 min addition of C_6_F_6_ to a vigorously stirred 0.05 M solution of 2a,§ in pyridine containing 5 mol% TMAF at room temperature (Scheme 1a). All starting materials and active intermediates are fully consumed, providing S_8_-corona[8]arene (1a) and minor amounts of the larger S_12_-corona[12]arene 1a′ (1a : 1a′ = 88 : 12) along with some acyclic/cyclic oligomers and insoluble polymer P1. Purification of this mixture is straightforward: P1 is simply removed by filtration once the reaction is completed, leaving only the macrocycles and trace non-macrocyclic materials in the solution. Trituration of the soluble fraction with pentanes removes all non-macrocyclic material, affording a colourless crystalline solid of 1a and 1a′ in a 27–31% yield. Both 1a and 1a′ show singlets in their ^1^H (Fig. 2) and ^19^F NMR spectra with similar chemical shifts.

MALDI-TOF mass spectrometry confirmed the identity of both macrocycles showing the expected mass and S_8_/S_12_ isotope patterns (ESI Fig. S134–S138†). Only negligible change in the relative ratios of 1a and 1a′ is observed in the one-pot reaction as a function of catalyst (compared to the fragment 3a approach below). Diffusion-Ordered NMR Spectroscopy (DOSY) was employed to distinguish the peaks correlating to 1a and 1a′, confirming the major product as 1a (Fig. 2b). Analytically pure 1a can be obtained through sublimation (0.9 mbar, 300–350 °C) of the 1a and 1a′ mixture.

### Pre-ordered synthesis of S_8_-corona[8]arene (1a)

To attain 1a chemospecifically, we devised a ‘pre-ordered’ approach, replacing C_6_F_6_ with thioether 3a (which is easily synthesised in high yields over two steps without extensive purification§). Use of 3a and a NH_4_F catalyst affords 1a chemospecifically (no. S12-corona[12]arene 1a′, Fig. 2a), on gram scales, without the need for chromatography, in a 28% yield (Scheme 1b). Even allowing for the initial preparation of 3a, gram amounts of 1a can be obtained rapidly without extensive purification requirements (24% yield over 3 steps). Again, only insoluble polymeric material results in the absence of the ammonium fluoride catalytic template. When using 3a (compared to C_6_F_6_) the choice of ammonium fluoride catalyst has a direct influence on the ratio of 1a to 1a′ (Fig. 2a). As might be anticipated, larger ammonium fluoride templates favour 1a′. While NH_4_F (AF) catalyses the formation of only 1a chemospecifically (Fig. 2a, 1a : 1a′ ratio 1 : 0.00), as the size of the catalytic template increases the amount of 1a′ also increases (up to a 1a : 1a′ ratio of 1 : 0.15 for TBAF). However, in each instance, the macrocycle yields remain comparable and DOSY NMR confirms only 1a and 1a′ form (Fig. 2b).

### S_8_-Corona[8]arene (1b–d and 1ab–cd) library synthesis

**Fig. 3:**
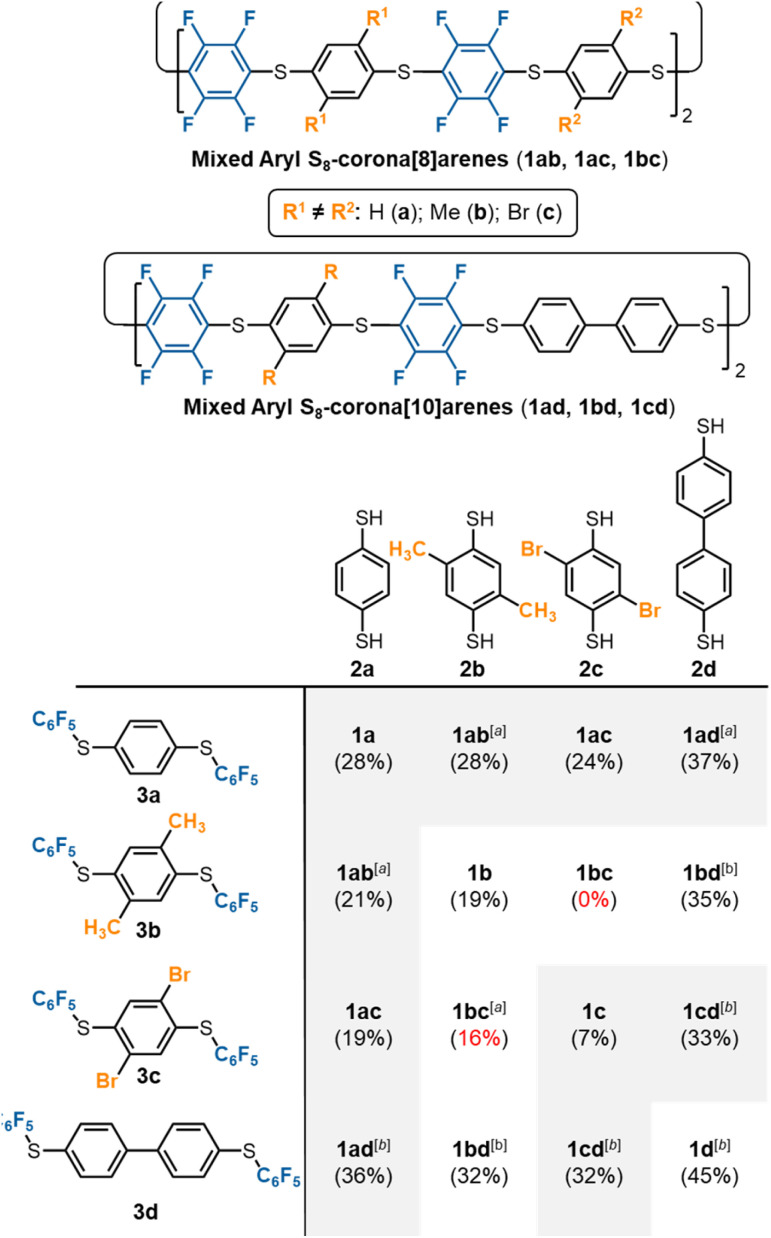
Library synthesis of mixed S_8_-corona[*n*]arenes (1). Footnotes: ^a^ mixture of both S_8_ (*n* = 2, major) and S_12_ (*n* = 3, minor) 1 formed; ^b^TBAF used as catalyst instead of TMAF.

The scope of the chemoselective macrocyclisation procedure was investigated using a range of easily prepared benzene-1,4-dithiols (2b–d) and thioether linkers (3b–d).§ With TMAF catalysis macrocycles 1b–d are synthesised chemoselectively as the desired S_8_-corona[*n*]arenes in a range of yields (7–45%). DFT calculations revealed a negligible difference in the C^δ+^–F^δ–^ polarisation energies (natural atomic charges) at the point of substitution within 3, yet significant differences in the rotational energy barrier (from 0.2 to 5.9 kcal mol^−1^) for S–C_6_F_5_ bond rotation of the terminal fluorobenzenoids in the thioethers (3b > 3c > 3a > 3d) (ESI Scheme S4 and Table S4†). As expected, increase in the steric demands of 3b and 3c compared to 3a results in lower yields of the corresponding macrocycles. Conversely, reducing the steric constraints by employing a 1,1′-biphenyl linker leads to the S_8_-corona[12]arene (1d) in higher yield (45%) although an appropriate (larger) TBAF catalytic template is needed. Using pairs of different 2a–d and 3a–d linkers allows rapid access to the library of dissymmetric S_8_-corona[*n*]arenes 1ab–cd chemoselectively (Fig. 3). As anticipated, some combinations of 2 and 3 are higher-yielding than others, which is attributed to the steric hindrance associated with the final macrocycle, and the rotational barrier of linkers 3a–d as previously discussed. Additionally, Fig. 3 shows the importance of pairing the nucleophile and electrophile combinations appropriately. The clearest example of this is the synthesis of 1bc (yields in red). A combination of the high rotational barrier linker 3b and low nucleophilicity 1,4-dithiol 2c, provides no macrocycle at all and only insoluble polymeric materials (92% by mass) and unreacted 3b (8% by mass) are recovered. Using instead the matched combination of 2b and 3c (simultaneously increasing nucleophile and electrophile reactivity) realises 1bc in modest yield. The poor nucleophilicity of 2c, can also be overcome with linker 3d, which has reduced rotational barriers allowing access to the large tetrabromide S_8_-corona[10]arene, 1cd in improved (32%) yield. In most cases of 1, the 8-ring S_8_-corona[*n*]arene framework forms highly chemoselectively, the exceptions being 1ab and 1bc where both the corresponding S_8_-corona[8]arene and S_12_-corona[12]arene are formed (independent of the precursor combination or catalyst used). Proton NMR analysis provides a [8] : [12] ratio of 1 : 0.13 for 1ab and 1 : 0.33 for 1bc respectively, with the smaller, S_8_-corona[8]arene being the major product for both (ESI Fig. S41, S81, and S132–S133†). DOSY/2D NMR spectroscopy (ESI Fig. S39–S133†) and MALDI-TOF mass spectrometry (ESI Fig. S134–S175†) confirm the preparation of all ten new S_8_-corona[*n*]arenes. In particular, each macrocycle shows a clear S_8_ isotope pattern at the expected mass, while the dissymmetric mixed aryl systems 1ab–cd show two ^19^F NMR environments as ‘AB’ systems or overlapping multiplets at *ca.* −133.0 ppm.

### Solid state structure

**Fig. 4:**
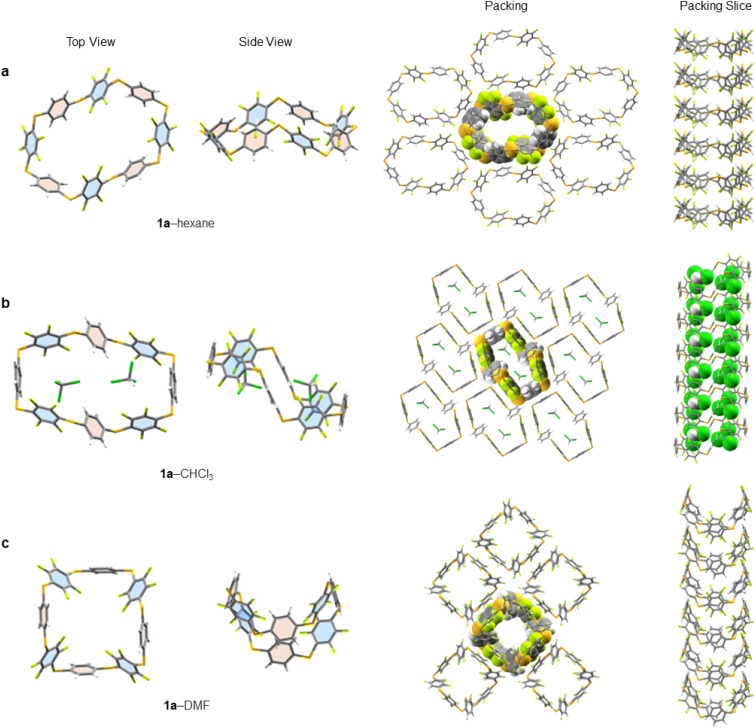
X-ray solid-state structures of 1a crystallised from: (a) hexane (b) CHCl_3_ and (c) DMF, viewed from the top and side. The solid-state packing of each crystal is viewed from the top and side where a central molecule or guest (space-filling representation) is shown embedded in a section of the lattice to illustrate the unidirectional crystal packing. All disordered solvent is removed for clarity, leaving highly ordered CHCl_3_ (part b of figure) to demonstrate the role of solvent in the final solid-state conformation of 1a.

Single crystals of 1a, suitable for X-ray diffraction, are obtained by either slow evaporation or solvent layering confirming the connectivity and size of 1a (see ESI† for full crystallographic details, ESI Fig. S139–S211† and cif files). We identified five solvates of 1a (1a–hexane, 1a–CHCl_3_, 1a–THF, 1a–pyridine and 1a–DMF). Three instructive examples are shown here (Fig. 4). While all pack in infinite honeycomb-like channels each solvate exhibits a dramatically different solid-state macrocyclic conformation. That in 1a–hexane (Fig. 4a) has an open crown-like conformation where the average C–S–C bond angle is 101.46°. The internal cavity of 1a–hexane, measured between centroids of opposite aryl units, has a large semi-major axis of 1.46 nm, is absent of ordered solvent and any intramolecular interactions distorting the internal cavity. Its solid-state structure differs from conventional ‘pillar-like’ arene macrocycles^12^ and instead, the arene units tilt alternatingly. To elucidate the important non-covalent interactions in the one-dimensionally assembled 1a–hexane macrocycles, Hirschfield analysis^28^ was employed (ESI Fig. S196†). Intermolecular hydrogen bonding (H–F contacts), type 1 F–F, and F–CH interactions between neighbouring aryl units (above and below) were identified as the major interactions dictating the columnar self-assembly of 1a–hexane (Fig. 4a). Between columns, intermolecular interactions between adjacent C_6_H_4_ units, 3 × CH–π (2.73 Å), and adjacent C_6_F_4_ units 4 × CF–π (2.96 Å) and 1 × F–F (2.77 Å) are present and dictate the adoption of the three-dimensional honeycomb-like lattice.

When crystallised from CHCl_3_, 1a forms a solid-state 1 : 2 host–guest inclusion complex, 1a⊂2CHCl_3_ (Fig. 4b). The macrocycle within this 1a–CHCl_3_ solvate adopts a ‘staircase-like’ conformation where the individual aryl units now point face-inwards to maximise electrostatic interactions between 1a and the guest. To efficiently accommodate both guests, the macrocycle lengthens to 1.57 nm, with an 8.40 Å reduction in width, and an increase in the average C–S–C bond angle to 103.51°, forming two equal and symmetrical binding sites on opposite sides of the macrocycle, to minimise unfavourable interactions between guests. This same conformational shift, in response to guest binding, is also observed in related 1a–THF, where a similar staircase-like conformation arises in response to a 1 : 2 host–solvent complex. While disorder prevents exact THF detection, the staircase conformation explicitly points to it. After 1 : 2 guest binding, the columnar supramolecular packing of 1a remains, generating infinite rigid column arrays (dictated by intermolecular hydrogen bonding between adjacent C_6_H_4_ and C_6_F_4_ units) saturated with guest/solvent molecules, a feature that is highly sought after for many applications, for example, gas exchange materials.^29^

When single crystals of 1a are grown through slow evaporation of DMF solutions, the macrocycle adopts a ‘tub-like’ conformation reminiscent of cyclooctatetraene (1a–DMF, Fig. 4c). Unlike with 1a–CHCl_3_, here the DMF solvent remains disordered amongst sheets of 1a in the solid state. The asymmetric unit reveals a pair of 1a macrocycles stacked on top of each other, with each C_6_H_4_ stacking on to a proximal C_6_F_4_ unit (and *vice versa*) to maximise intermolecular hydrogen bonding between adjacent macrocycles. As in the other structures, these weak hydrogen bonds facilitate the formation of well-defined columns, which are characteristic of all the solvates of 1a identified to date. Substitution of R = H (1a) to other macrocycles changes this behaviour. For example, in the X-ray structure of 1b (R = Me, ESI Fig. S209†) staggered channels form with overlap between adjacent columns, forming infinite chain-linked columns, permitting multiple intermolecular CF–π interactions to form (ESI Fig. S210†).

### Late-stage library functionalisation

All the S_8_-corona[*n*]arene backbones (1), reported here, can be used as versatile macrocyclic backbones for one-step late-stage S_N_Ar-based multi-functionalisations, rapidly and selectively without recourse to multi-step protecting group strategies. They provide excellent building blocks for a wide range of onwards chemistries. This is an important feature for convenient adoption and provides an alternative macrocyclic framework to current air-sensitive hydroxypillarene approaches.^23^

**Scheme 2:**
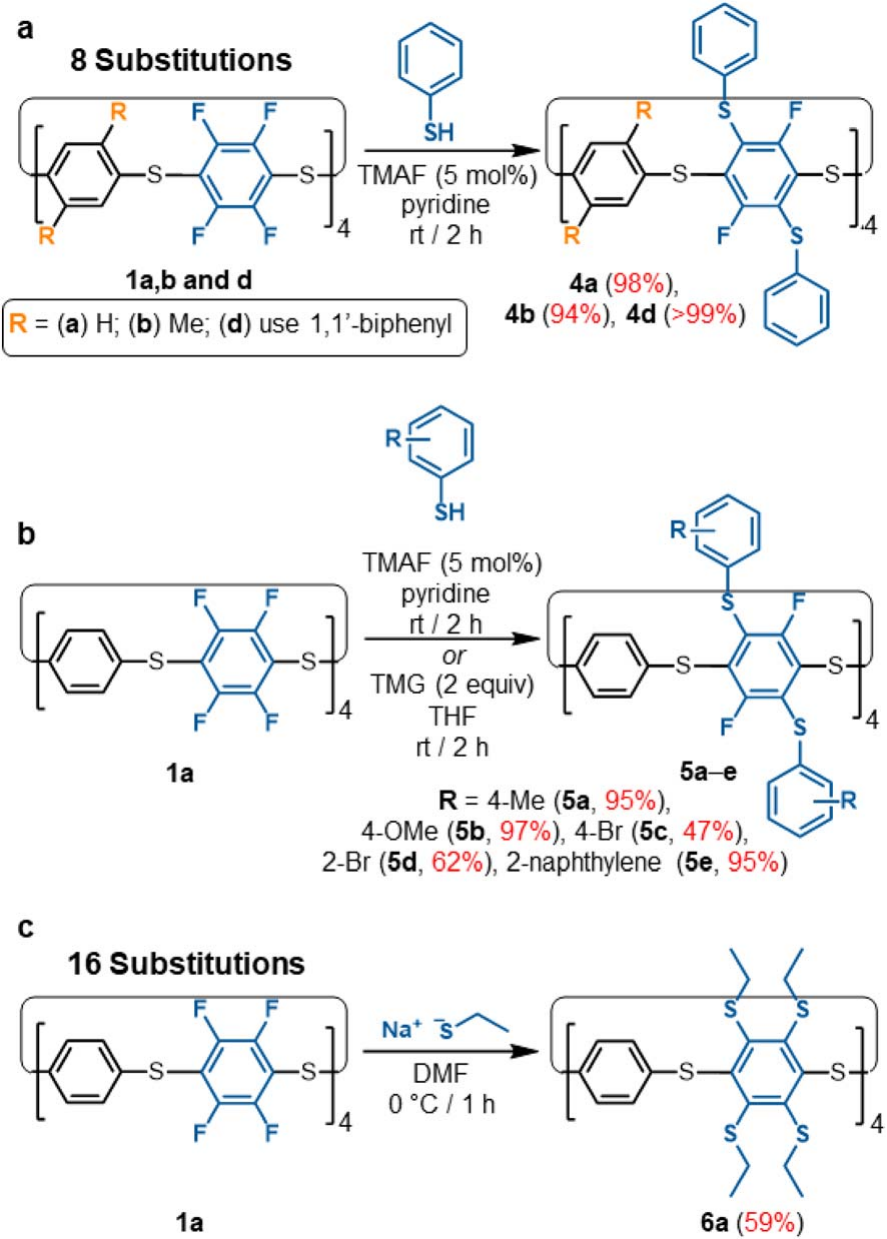
(a) Synthesis of thiophenol functionalised S_8_-corona[*n*]arenes (4a, 4b, 4d). (b) Scope of 8-fold functionalisation of 1a to 5a–e; (c) 16-fold functionalisation of 1a to 6a.

As a proof-of-concept, our TMAF/pyridine S_N_Ar catalysis protocol was used to synthesise a range of thiol substituted S_8_-corona[*n*]arenes under mild conditions in high yields (Scheme 2). In each case, these substitutions converge into a single isomerically pure product, overcoming the need for laborious separations of positional isomers. The large aryl thiol units along with the steric restraints of the macrocycle framework prevent complete substitution of all 16 aryl-fluoride groups within 1a. Instead, only selective 2,5-functionalisation occurs on every C_6_F_4_ unit for a total of 8 substitutions. Initial testing was conducted on NMR scales with a small excesses of thiophenol (10–16 equiv.) being added to a sample of 1a in pyridine containing TMAF with no precautions taken to remove oxygen or moisture: ^19^F NMR analysis shows the singlet at −134 ppm for 1a is quickly disrupted into many peaks spanning −90 to −134 ppm which are attributed to the positional isomers caused by partial substitutions. Over the course of 2 hours, these peaks all converge into a new singlet at *ca.* −90 ppm, representing the targeted 2,5-functionalised macrocycle 4a (ESI Fig. S129† for NMR monitoring). Macrocycles 1b and 1d, show equal success in equivalent functionalisation with thiophenol. In all cases, purification is straightforward with simple trituration with water, MeOH, and then Et_2_O (to remove pyridine HF-pyridine, TMAF, unreacted excess nucleophile, and any disulfide) being sufficient to isolate 4 as pure colourless solids. The [8SPh]S_8_-corona[*n*]arenes (4a, 4b, and 4d) are equivalently attained in excellent yields, selectively, in a single step from 1a, 1b, and 1d respectively (Scheme 2a). To confirm equivalent reactivity at scale, 4a was synthesised under the same conditions on a *ca.* 0.1 g scale and successfully isolated in 98% yield. Numerous thiophenol derivatives also readily participate of which 5a–5e provide a representative series. In all cases the catalysed S_N_Ar reactions converge into a single octafunctionalised derivative in fair to excellent yields without the need for large excesses of thiol.

Attempted complete C–F displacement of 1a using large excesses of PhSH and heating results in significant thiol–thioether exchange causing the macrocyclic framework to break down into an uncharacterisable mixture. However, per-substitution of 1a could be achieved using smaller, non-aryl nucleophiles such as sodium ethanethiolate, providing 6a under mild conditions (Scheme 2c). Restricted rotation barriers result in broad ^1^H NMR signals (ESI Fig. S124†) and prevent ^13^C NMR characterisation. However, the absence of ^19^F NMR signals and a clear MALDI-TOF molecular ion confirm per-substituted (ESI Fig. S191–S192†). With their ease of substitution and convenient ^19^F NMR handle for reaction monitoring, S_8_-corona[*n*]arene backbones 1 are, we propose, highly attractive versatile building blocks for wide fields of application, *e.g.* ‘designer’ guests, molecular machine components, organic frameworks, *etc.*

### Host–guest binding studies

Next, we turned to UV-vis titration experiments of 1a in THF solution with a variety of guests relevant to our catalytic, synthetic and X-ray studies to determine their binding affinities/energetics (ESI Fig. S212† and provided data). Due to the high THF (neat) solvent molecularity (12.3 M), titration of small amounts of alternate guests (G) into THF solutions of 1a (entropically) favours 1 : 1 binding, provided no cooperative guest binding effects occur.^30^ When 10 μM solutions of 1a in THF are titrated with CHCl_3_, DMF, and pyridine these provide reproducible good fits to 1 : 1 binding isotherms, based on simulation of the absorbance maxima at 295 nm (Table 1). Binding of larger NBu_4_Cl follows the same 1 : 1 behaviour, as does NBu_4_F, provided low relative concentrations (1a : NBu_4_F < 1 : 5) of the latter guest are used. At higher relative concentrations of NBu_4_F : 1a a second association process is triggered and a new charge transfer band (*ε*_HG_ = 5.9(9) × 10^4^ M^−1^ cm^−1^) at 370 nm, associated with a clear isosbestic point at *ca.* 315 nm, appears. These indicate the presence of a second species whose formation is induced by the initial NBu_4_F binding. A second 1 : 1 binding constant of *K* = 1.0(3) × 10^3^ M^−1^ (*R*^2^ 0.97) can be determined from absorbance data at 370 nm indicating this second process has Δ*G*° −4.0 kcal mol^−1^. This value is rather close to the gas-phase association energy we determine (DFT) for the interaction of NMe_4_F with C_6_F_6_ (−12 kcal mol^−1^, ESI Fig. S216†). We therefore propose that the charge transfer band at 370 nm is direct experimental evidence for the NR_4_F⋯fluoroaryl interaction we calculate (ESI Fig. S216†), and that this is at the heart of the high S_N_Ar rate accelerations we see in the formation of 1a and in its subsequent derivatisations.

**Table 1:** 1 : 1 Guest binding to macrocycle 1a^a^

Guest	*K* (M^−1^)	*ε*_HG_ (M^−1^ cm^−1^)	Δ*G*^°^ (kcal mol^−1^)	(*R*^2^)
CHCl_3_	3.6(7)	5.85(10) × 10^4^	−0.8	0.99
DMF	7.4(8)^b^	5.56(4) × 10^4^	−1.2	0.99
Pyridine	8(1) × 10^3^	5.7(3) × 10^4^	−5.2	0.97
NBu_4_Cl	2.6(3) × 10^4^	6.14(4) × 10^4^	−5.9	0.98
NBu_4_F	1.5(2) × 10^4^	7.15(10) × 10^4^	−5.6	0.99

a Determined by UV-vis titration in THF containing 190 ± 20 ppm water; the average of two reproducible duplicates is presented. The number in parentheses indicates the standard deviation in the preceding figure.

b *K* = 3.3(6) M^−1^ (*R*^2^ 098) in THF containing 300 ppm water.

Control studies indicate that the absorbance of 1a in THF is only weakly affected by the presence of water (*δ*_Abs_ 0.001–0.003), when the water levels in the THF are 150–250 ppm. All our studies were conducted at 190 ± 20 ppm water in THF as measured by Karl–Fisher titration. Water concentrations, above 300 ppm negatively affect the initial 1 : 1 guest binding (*c.f.* the DMF guest entry data within Table 1). This is most acutely apparent in attempted determinations of NMe_4_F binding to 1a. Hydrated NMe_4_F is insoluble in THF and only readily soluble in DMF containing significant water (*ca.* 2000 ppm). This water reduces NMe_4_F binding to 1a to a point where reproducible accurate *K* values cannot be determined. However, it is still clear from the qualitative UV spectra that the same two-step process is occurring, as the same 370 nm charge transfer band still emerges at higher 1a : NMe_4_F ratios. It is likely, based on our X-ray data, that initial guest binding to macrocycle 1a is driven by H-bonding and π–π interaction, while the second association of additional NR_4_F involves interaction of a C_6_F_4_ unit within 1a with a NR_4_F contact ion pair (DFT calculated, ESI Fig. S216†). To test this hypothesis, NMe_4_F binding to 1a was attempted in dry THF/CH_2_Cl_2_/MeOH mixtures. The presence of MeOH is known to deliver the solvent separated ion pair NMe_4_^+^ F^−^.^27^ Consistent with our proposal, no binding of any type of 1a to NMe_4_F is observed in this solvent mixture.

To help identify the solution state conformation of 1a a DFT conformational search was also made (ESI Fig. S217†). In the absence of guests, the lowest energy conformers are associated with transannular π–π contacts of the two central phenylene rings (either C_6_F_4_⋯C_6_F_4_, or C_6_H_4_⋯C_6_H_4_, see ESI Fig. S217†). These structures are significantly lower in energy than the open conformation of unsolvated 1a (as modelled from the X-ray structure of 1a-hexane). In adopting such π–π stacked conformers the macrocycle 1a twists generating two independent ‘bays’, each suitable for independent binding of a single guest. This arrangement is rather similar to that observed in the X-ray crystal structure of 1a–CHCl_3_, minus its guests. The S_0_ → S_*n*_ absorption spectra of the two calculated lowest energy conformers (in the absence of any guest) using adiabatic linear-response time-dependent density functional theory (TDDFT) with the M06-2X/6-311++G(d,p) functional and electronic basis set combination are in accord with the experimental solution spectra (250–300 nm). Conversely, the charge transfer band (370 nm) observed with NR_4_F (R = Me, Bu) is most consistent with a NR_4_FC_6_F_*n*_ (*n* = 4–6) contact lowering the C=C bond order of the fluoroaryl unit.

## Conclusions

New S_N_Ar catalytic-template protocols using NR_4_F (R = H, Me, Bu) in pyridine allow unprecedented access to large fluorinated S_8_-corona[*n*]arenes 1 (*n* = 8, 10, 12). This new class of functional sulphur-bridged arene macrocycle is easily and rapidly prepared from low cost and widely available reagents. Yields approaching 45% and preparations at gram scales (without recourse to tedious chromatographic separation) are readily attained in technically simple reactions. Kinetic, thermodynamic and computational studies are in accord with catalytic activation arising from fluoroaryl⋯NR_4_F ion pair binding delivering rate acceleration and macrocyclic templating. Importantly, the pyridine solvent favours the necessary ion pairs,^27^ but also binds the product macrocycle sufficiently well (*K* ∼ 8 × 10^3^ M^−1^) that it is calculated to displace the catalyst from 1 (when present in excess) allowing catalytic turn over. An additional benefit of fluorinated S_8_-corona[8]arenes 1 is their predisposition to efficient and highly selective S_N_Ar C_6_F_4_-sub unit substitution reactions with thiols using the same NR_4_F catalysis protocols. Entirely regiospecific eight-fold substitutions in nearly quantitative yields are attained without the need for large excesses of thiol. Overall, the excellent hosting and easy ‘designer’ potential of 1 gives this new class of macrocycle very high potential for use across the molecular, supramolecular and polymeric sciences in many applications, especially as they are so easily prepared.

## Data availability

All available primary data are provided in the main text and ESI.†

## Author contributions

A. T. T. synthesised and characterised all materials. M. W. D. H.-H. performed DFT calculations and simulations. S. P. A. collected and resolved all crystallographic data. M. F. coordinated SEM studies and analysis. Y. H. collected SEM data for 1a and P1. S. W. carried out the kinetic and thermodynamic studies. A. T. T. and S. W. co-wrote the manuscript and conceived the research. All authors have reviewed and given approval to the final version of the manuscript.

## Conflicts of interest

The macrocycle 1a is now commercially available from https://www.keyorganics.net, A. T. T and S. W. declare their interest in this venture.

## Supplementary Material

SC-014-D2SC05348A-s001

SC-014-D2SC05348A-s002

SC-014-D2SC05348A-s003

SC-014-D2SC05348A-s004
