# Supplementary material for: Catalysis enabled synthesis, structures, and reactivities of fluorinated S8-corona[n]arenes (n = 8–12)
Source: Chem Sci. 2022 Nov 16;14(1):70–7. doi: 10.1039/d2sc05348a (PMC9769089; doi:10.1039/d2sc05348a)
Supplement: SC-014-D2SC05348A-s001 [file SC-014-D2SC05348A-s001.pdf]

Electronic Supplementary Material (ESI) for Chemical Science.
This journal is © The Royal Society of Chemistry 2022

Catalysis Enabled Synthesis, Structures, and Reactivities of Fluorinated S_8-Corona[n]arenes (n = 8-12)

Andrew. T. Turley,*[a] Magnus W. D. Hanson-Heine,[b] Stephen. P. Argent,[b] Yaoyang Hu,[a] Thomas. A. Jones,[a] Michael Fay,[c] Simon Woodward*[a]

[a.] *GSK Carbon Neutral Laboratories for Sustainable Chemistry, University of Nottingham, Jubilee Campus, Nottingham NG7 2TU, UK. E-mail: andrew.turley@nottingham.ac.uk, simon.woodward@nottingham.ac.uk.*

[b.] *School of Chemistry, University of Nottingham, University Park Campus, Nottingham NG7 2RD, UK.*

[c.] *Nanoscale and Microscale Research Centre, University of Nottingham, Cripps South Building, University Park Campus, Nottingham, NG7 2RD, UK.*

Electronic Supporting Information

1.	General Methods	S2
2.	Synthetic Procedures	S4
3.	^{1}H, ^{13}C and ^{19}F NMR Spectroscopic Characterization of Synthesised Compounds	S24
3.1.	^{1}H and ^{19}F NMR Characterisation of **1a** and **1a’** from Various Templates.	S146
3.2.	^{1}H NMR Reaction Monitoring	S150
3.3.	Diffusion-Ordered NMR Spectroscopy (DOSY)	S151
4.	Matrix-assisted Laser Desorption/Ionisation TOF Mass Spectrometry	S155
5.	X-Ray Crystallographic Analysis	S214
5.1.	Single Crystal, Packing and Analysis: **1a–Hexane**	S217
5.2.	Single Crystal, Packing and Analysis: **1a–$CHCl_3$**	S221
5.3.	Single Crystal, Packing and Analysis: **1a–THF**	S224
5.4.	Single Crystal, Packing and Analysis: **1a–Pyridine**	S227
5.5.	Single Crystal, Packing and Analysis: **1a–DMF**	S230
5.6.	Single Crystal, Packing and Analysis: **1b–$CHCl_3$**	S233
6.	UV-Vis Titrations and Binding Calculations	S239
7.	NMR Kinetic Analysis	S243
7.1.	^{19}F NMR Kinetic Investigation of One-pot Macrocycle Formation	S243
7.2.	Studies of Rate Acceleration by $NMe_4F{\cdot}4H_2O$	S247
8.	Computational Analysis	S249
8.1	Steric *vs*. Electronic Effects on Macrocyclisation	S254
9.	Comparison of Macrocycle **1a** to Polymeric **P1**	S270
10.	Thermogravimetric Analysis (TGA)	S275
11.	References	S276

1. General Methods

Materials. All reagents were purchased from commercial suppliers (Sigma-Aldrich, Acros Organics, Fluorochem or Alfa Aesar) and used without further purification. Dimethylformamide and acetamide were of an anhydrous grade. **Quenching and waste management safety advice** is given at the end of this experimental section (p S22).

Instrumentation and Analytical Techniques. Analytical thin-layer chromatography (TLC) was performed on neutral aluminium sheet silica gel plates and visualised under UV irradiation (254 nm). Nuclear magnetic resonance (NMR) spectra were recorded using a Bruker Advance (III)-400 (^{1}H 400 MHz and ^{13}C 101 MHz) or a Bruker 500 MHz three channels with broad-band tuneable liquid-nitrogen cooled cryoprobe (^{1}H 500 MHz, ^{13}C 126 MHz and ^{19}F 471 MHz) spectrometer, at a constant temperature of 298 K unless otherwise stated. Chemical shifts (δ) are reported in parts per million (ppm) relative to the signals corresponding to residual non-deuterated solvents [$CDCl_3$: δ = 7.26 or 77.16. CD_2Cl_2: δ = 5.32 or 54.00]. Coupling constants (J) are reported in Hertz (Hz). ^{13}C NMR Experiments were proton decoupled. Assignment of ^{1}H and ^{13}C NMR signals were accomplished by two-dimensional NMR spectroscopy (COSY, NOESY, HSQC and HMBC). For all macrocycles, ^{13}C NMR shifts were attained from both standard ^{13}C{^{1}H} and ^{13}C{^{19}F} decoupled NMR experiments. All NMR spectra were processed using MestReNova version 11. Flourine-19 NMR spectra were directly referenced to C_6F_6 (defined as –164.9 ppm), except where noted otherwise (internal spectrometer referencing used). All NMR data are reported as follows: chemical shift; multiplicity; coupling constants; integral and assignment. Mass spectra (including high resolution studies, HR) were performed using a Bruker ESI-TOF MicroTOF II operating in ESI mode for low mass species. Deviations from the expected exact mass is given as σ values (ppm), theoretical exact masses were calculated using ChemDraw ver. 20. Solid state ^{19}F MAS spectra were collected using both a Bruker Avance III HD 800 MHz spectrometer, equipped with a triple resonance 3.2 mm HCN CP-MAS probe and a Bruker Avance III HD 600 MHz spectrometer equipped with a triple resonance 1.3 mm HCN CP-MAS probe. The temperature of the sample during the all the NMR acquisition was set to 25 °C. MALDI-MS data were acquired for macrocylic species, due to their high mass, using a Brucker Autoflex Max. This system's software was used to simulate the expected isotope mass distributions. MALDI deviations were in the expected range

for a *de novo* molecule class without an identified optimal calibrant. Melting points were recorded using a Gallenkamp (Sanyo) apparatus and are uncorrected. UV-Vis spectra was collected on an Agilent Cary UV-Vis-NIR spectrometer. Scanning Electron Microscopy (SEM) samples were prepared by depositing materials as powders on carbon tape and coated with 8 nm iridium. The SEM images were attained on a Jeol 7000F FEG-SEM. Single crystals were selected and mounted using Fomblin® (YR-1800 perfluoropolyether oil) on a polymer-tipped MiTeGen MicroMountTM and cooled rapidly to 120 K in a stream of cold N_2 using an Oxford Cryosystems open flow cryostat.[1] Single crystal X-ray diffraction data were collected on either a Rigaku XtaLAB PRO MM007 (PILATUS3 R 200K Hybrid Pixel Array detector, mirror-monochromated Cu-Kα radiation source; λ = 1.54184 Å, ω scans) (**1a-Chloroform**, **1a-THF**) or in Experimental Hutch 1 (EH1) on beamline I19 at Diamond Light Source[2] on a Fluid Film Devices 3-circle fixed-chi diffractometer (Dectris PILATUS 2M Detector, wavelength 0.6889 Å) (**1a-Chloroform**, **1a-DMF**, **1a-Pyrdine**, **1b**). Data Collection was handled by either CrysAlisPRO[3] (Rigaku XtaLAB) or GDA (Diamond Light Source). The collected frames were integrated using either CrysAlisPRO (Rigaku XtaLAB) or XIA26 software[4] (Diamond Light Source) and the data were corrected for absorption effects using a Gaussian numerical method with beam profile correction (CrysAlisPro) or AIMLESS[5] an empirical method (Diamond Light Source). Structures were solved within Olex2[6] by dual space iterative methods (SHELXT)[7] and all non-hydrogen atoms refined by full-matrix least-squares on all unique F2 values with anisotropic displacement parameters (SHELXL).[8] Hydrogen atoms were refined with constrained geometries and riding thermal parameters. Structures were checked with checkCIF.[9]

Open Data. CCDC-2175101-2175106 contains the X-ray supplementary data for all compounds appearing in this paper. These data can also be obtained free of charge from The Cambridge Crystallographic Data Centre via www.ccdc.cam.ac.uk/data_request/cif. The primary physical organic chemistry data (guest binding and kinetic studies) for with this manuscript are contained within the files 'UV-vis PRIMARY DATA and BINDING FITS' (Excel) and 'Kinetic PRIMARY DATA and RATE FITS' (Excel) associated with this paper.

2. Synthetic Procedures

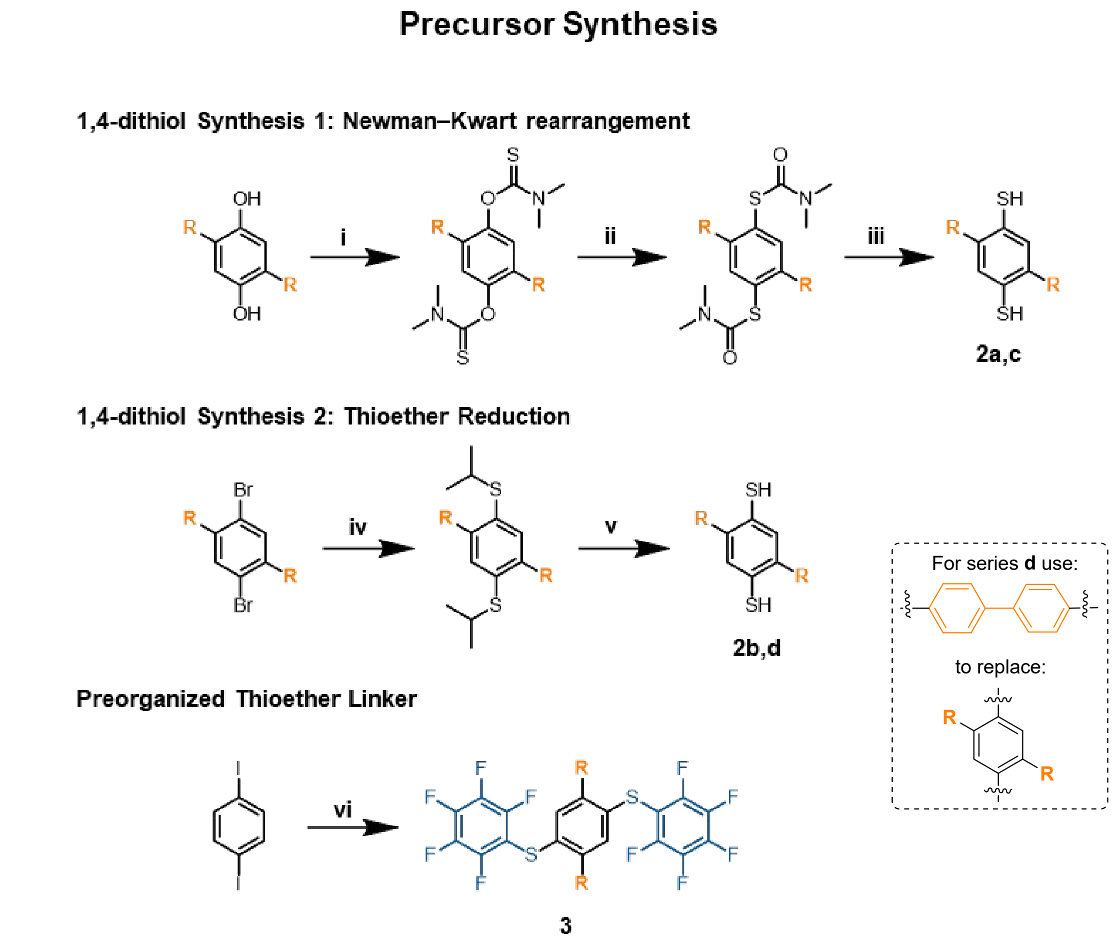

Figure S1. Synthesis routes used to prepare S_8-corona[n]arene precursors. **Reagents and conditions**: (i) Dimethyl thiocarbonyl chloride / DABCO / NMP / 55 °C / 20 h; (ii) sulfolane or NMP / heat / 0.33 h; (iii) KOH / MeOH / reflux / 4 h; (iv) sodium isopropylthiolate / DMA / 110 °C / 20 h; (v) Na / DMA / 110 °C / 2 h; (vi) C_6F_5SCu / DMF / 145 °C / 2.5 h. For dithiol **2d**, 4,4'-dibromo-1,1'-biphenyl was used.

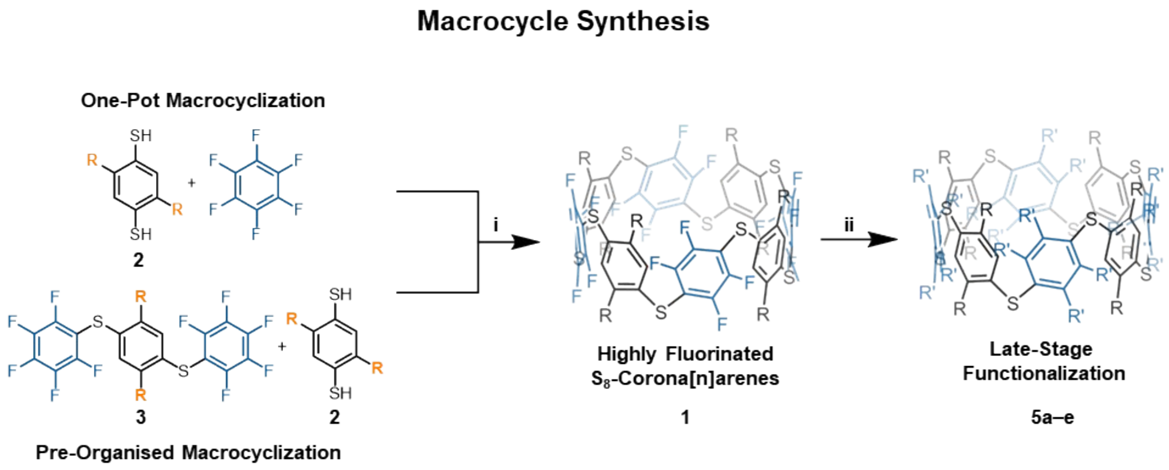

Figure S2. Synthesis routes used to prepare S_8-corona[n]arenes. Reagents and conditions: (i) pyridine / catalyst / rt / 10 min; (ii) nucleophile / catalyst / base / rt / 2 h.

Synthetic note: Primary spectra of all entities prepared are presented in Figures S3-S133.

Benzene-1,4-dithiols (2a-c) and 1,1'-Biphenyl-4,4'-dithiol (2d)

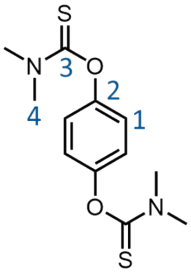

***O,O*'-(1,4-Phenylene)*bis*(dimethylcarbamothioate)**: To an oven-dried Schlenk tube was added hydroquinone (2.00 g, 18.0 mmol), DABCO (5.04 g, 44.9 mmol), and anhydrous *N*-methyl-2-pyrrolidone (30 mL) and the mixture heated to 50 °C. A solution of dimethyl thiocarbonyl chloride (4.74 g, 38.3 mmol) in anhydrous *N*-methyl-2-pyrrolidone (8 mL) was added dropwise to the heated mixture over 30 min. The mixture was then stirred at 55 °C for 20 h. Water (100 mL) was added dropwise over 1 h at 55 °C, yielding a light brown precipitate. The precipitate was isolated by vacuum filtration and washed with water (3 × 25 mL), then dried at 50 °C under reduced pressure. The title product was isolated as a beige powder (2.60 g, 9.15 mmol, 51%). **Mp** 214 – 216 °C [Lit.[10] 214 – 216 °C]. **^{1}H NMR** (500 MHz, $CDCl_3$) δ 7.08 (s, 4H, H_1), 3.46 (s, 6H, H_4), 3.34 (s, 6H, H_4). **^{13}C NMR** (126 MHz, $CDCl_3$) δ 187.9 (C_3), 151.6 (C_2), 123.6 (C_1), 43.4 (C_4), 38.9(C_4). **HR-ESI-MS** *m/z* = 307.0548 $[M+Na]^+$ (calculated for $C_{12}H_{16}N_2O_2S_2Na^+$ = 307.0545) (σ 1). Literature compound, but only mp reported.[10]

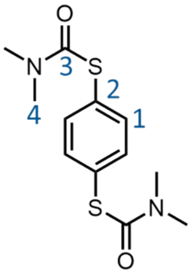

***S,S*'-(1,4-Phenylene)*bis*(dimethylcarbamothioate)**: To an oven-dried Schlenk tube was added, *O,O*'-(1,4-phenylene)*bis*(dimethylcarbamothioate) (0.92 g, 3.24 mmol) and sulfolane (2 mL). The mixture was stirred and heated to 280 °C for 1 h under vigorous stirring with no precautions taken to maintain an inert atmosphere. The partially cooled (still hot) mixture was slowly added dropwise to water (5 mL), to form a light brown precipitate. The precipitate was isolated by vacuum filtration and washed with water (3 × 25 mL), then dried at 50 °C under reduced pressure. The title product was isolated as a beige solid (0.85 g, 3.00 mmol, 93%). **Mp** 201 – 203°C [Lit.[10] 200 – 202 °C]. **^{1}H NMR** (500 MHz, $CDCl_3$) δ 7.50 (s, 4H, H_1), 3.03 (br, s, 12H, H_4). **^{13}C NMR** (126 MHz, $CDCl_3$) δ 166.5 (C_3), 135.9 (C_1), 130.3 (C_2), 37.1 (C_4). **HR-ESI-MS** *m/z* = 307.0552 $[M+Na]^+$ (calculated for $C_{12}H_{16}N_2O_2S_2Na^+$ = 307.0545) (σ 2). Literature compound, but only mp available.[10]

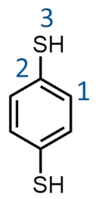

Benzene-1,4-dithiol (2a): To an oven-dried round bottom flask was added, *S,S*'-(1,4-phenylene)*bis*(dimethylcarbamothioate) (0.85 g, 3.00 mmol), KOH (3.36 g, 60.0 mmol, 20 equiv) and MeOH (17 mL). The reaction mixture was heated to reflux for 4 h under an N_2-atmosphere. The reaction mixture was allowed to cool to rt before being poured onto 2 M HCl (20 mL) in an ice bath under nitrogen and stirred for 15 min. The resulting colourless solid was isolated by vacuum filtration and washed extensively with water (5 × 30 mL), and dried under reduced pressure. The title product was isolated as a colourless solid (0.40 g, 2.81 mmol, 94%). **Mp** 96 – 98 °C [Lit.[11] 97 – 99 °C]. **^{1}H NMR** (500 MHz, $CDCl_3$) δ 7.16 (s, 4H, H_1), 3.41 (s, 2H,

H_2). **HR-ESI MS** *m/z* = 140.9829 [M-H]$^-$ (calculated for $C_6H_5S_2^-$ = 140.9838) (σ 6). All data identical to commercial samples, literature compound.[11]

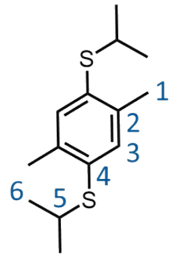

(2,5-Dimethyl-1,4-phenylene)*bis*(isopropylsulfane): To a flame-dried Schlenk flask was added, sodium isopropylthiolate (2.15 g, 21.9 mmol, 4 equiv., **CARE!** *i*-PrSH is very malodorous) and 1,4-dibromo-2,5-dimethylbenzene (1.40 g, 5.47 mmol) and the tube deoxygenated through 3 × vacuum-N_2 cycles. Anhydrous DMA (12 mL) was deoxygenated (3 × freeze-pump-thaw cycles under N_2) and added to the reaction mixture, which was then stirred at 110 °C for 20 h under N_2. The reaction mixture was allowed to cool to rt, poured onto brine (30 mL) and extracted with Et_2O (5 × 25 mL). The combined organic extracts were washed extensively with water (5 × 30 mL), dried ($MgSO_4$), filtered and the solvent was removed under reduced pressure to yield a colourless oil that crystallised on standing. The title product was isolated as a colourless crystalline solid (1.30 g, 5.12 mmol, 93%). **Mp** 38 – 40°C. **^{1}H NMR** (500 MHz, $CDCl_3$) δ 7.19 (s, 2H, H_3), 3.33 (sept, *J* = 6.8 Hz, 2H, H_5), 2.35 (s, 6H, H_1) 1.29 (d, *J* = 6.8 Hz, 12H, H_6). **^{13}C NMR** (126 MHz, $CDCl_3$) δ 137.3 (C_2), 133.3 (C_4), 133.3 (C_3), 37.9 (C_5), 23.3 (C_6), 20.4 (C_1). **HR-ESI-MS** *m/z* = 255.1227 [M+H]$^+$ (calculated for $C_{14}H_{23}S_2^+$ = 255.1236) (σ 4). Literature compound, available data concordant.[12]

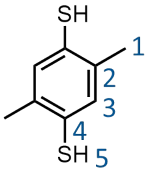

2,5-Dimethylbenzene-1,4-dithiol (2b): To a flame-dried Schlenk flask was added, anhydrous DMA (12 mL) that had been deoxygenated (3 × freeze-pump-thaw cycles under N_2) and sodium metal (83 mg, 3.6 mmol, 4 equiv.) then stirred at rt for 5 min under N_2. Solid (2,5-dimethyl-1,4-phenylene)*bis*(isopropylsulfane) (0.23 g, 0.91 mmol) was added to the reaction mixture which was then stirred at 110 °C for 2.5 h under nitrogen. The reaction mixture was allowed to cool to rt before being pipetted onto ice cooled 2 M HCl (5 mL) under a nitrogen atmosphere (**CARE!** Check all Na **has** been consumed in the reaction first) and stirred for 15 min. The resulting colourless solid was isolated by vacuum filtration and washed extensively with water (5 × 30 mL), and dried under reduced pressure. The title product was isolated without the need for further purification as colourless microcrystals (0.15 g, 0.88 mmol, 97%). **Mp** 120 – 121 °C. **^{1}H NMR** (400 MHz, $CDCl_3$) δ 7.09 (s, 2H, H_3), 3.20 (s, 2H, H_5), 2.25 (s, 6H, H_1). **^{13}C NMR** (126 MHz, $CDCl_3$) δ 134.7 (C_2), 132.0 (C_3), 128.1 (C_4), 20.51 (C_1). **ESI-MS** *m/z* = 171.0272 [M+H]$^+$ (calculated for $C_8H_{11}S_2^+$ = 171.0297) (σ 14). Literature compound, available data concordant.[12] Direct precipitation of **2b** in cold HCl, significantly simplifies its isolation and avoids the disulfide oxidations noted in the primary literature reports.[12] This approach is also applicable to **2a**, **2c** and **2d**.

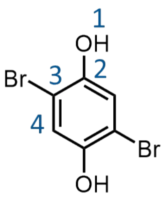

2,5-Dibromohydroquinone: To an oven-dried round bottom flask was added, hydroquinone (2.0 g, 18.2 mmol), acetic acid (15 mL) and the solution cooled to 10 °C in a cold water bath. Neat bromine (1.9 mL, 5.9 g, 37 mmol) was added dropwise over 0.5 h. The reaction mixture was stirred for 20 h, while the temperature was allowed to slowly warm from 10 °C to rt. Upon complete consumption of hydroquinone, a small amount of $Na_2S_2O_3$ was added, turning the reaction mixture from brown to off-white. Water (100 mL) was added over 0.5 h, forming an off-white precipitate. The precipitate was isolated by vacuum filtration and washed with water (3 × 25 mL), then dried at 50 °C under reduced pressure. The title product was isolated as a colourless crystalline solid (3.48 g, 13.1 mmol, 72%). **Mp** 187 – 189 °C [Lit.[13] 187 – 190 °C]. **^{1}H NMR** (500 MHz, DMSO-d_6) δ 9.76 (s, 2H, H_1), 6.95 (s, 2H, H_4). **^{13}C NMR** (126 MHz, DMSO-d_6) δ 147.4 (C_2), 119.5 (C_4), 108.3 (C_3). **HR-ESI-MS** *m/z* = 264.8508 $[M-H]^+$ (calculated for $C_6H_3Br_2O_2^+$ = 264.8494) (σ 5). Literature compound, data concordant.[13]

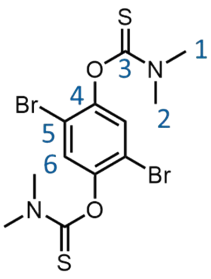

***O,O*'-(2,5-Dibromo-1,4-phenylene)*bis*(dimethylcarbamothioate)**: To an oven-dried two-neck round bottom flask fitted with a condenser and a dropping funnel, was added 2,5-dibromohydroquinone (2.70 g, 10.1 mmol), 1,4-diazabicyclo[2.2.2]octane (2.10 g, 18.8 mmol, 2.5 equiv.) and anhydrous NMP (8 mL). The reaction mixture was heated to 50 °C and a solution of dimethylthiocarbonyl chloride (2.80 g, 22.7 mmol, 2.25 equiv.) in anhydrous NMP (4 mL), was **slowly** added dropwise to the heating mixture over 30 mins. (**CARE!** slow addition is **vital** because the reaction is exothermic). Upon complete addition, the reaction mixture was stirred at 50 °C for 6 h. Water (30 mL) was added dropwise over 30 mins to the heated mixture to induce initial precipitation, before the reaction was cooled slowly to 20 °C. The precipitate was isolated by vacuum filtration and washed with water (3 × 25 mL). The crude solid was purified by column chromatography (Al_2O_3, pentane-CH_2Cl_2, 30%). The title product was isolated as a colourless crystalline solid (3.90 g, 8.82 mmol, 87%). **Mp** 190–192 °C. **^{1}H NMR** (500 MHz, $CDCl_3$) δ 7.40 (s, 2H, H_6), 3.46 (s, 6H, H_2), 3.38 (s, 6H, H_1). **^{13}C NMR** (126 MHz, $CDCl_3$) δ 185.8 (C_3), 149.2 (C_4), 128.8 (C_6), 116.02 (C_5), 43.7 (C_2), 39.1 (C_1). **HR-ESI-MS** *m/z* = 462.8758 $[M+Na]^+$ (calculated for $C_{12}H_{14}Br_2N_2O_2S_2Na^+$ = 462.8756) (σ <1).

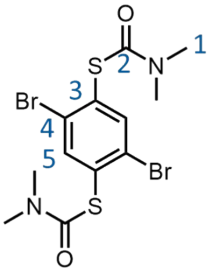

***S,S*'-(2,5-Dibromo-1,4-phenylene)*bis*(dimethylcarbamothioate)**: To an oven-dried microwave vial was added *O,O*'-(2,5-dibromo-1,4-phenylene)*bis*(dimethylcarbamothioate) (1.00 g, 2.26 mmol) and anhydrous NMP (4 mL). The reaction mixture was sparged with N_2 and sealed before

being heated in a microwave reactor at 230 °C for 20 min. Once cooled, the solution was added dropwise to water (30 mL) and the crude brown precipitate was isolated by vacuum filtration and washed with water (3 × 25 mL). The crude mixture was recrystallised from hot EtOH and cooled to –20 °C to further induce crystallisation. The title product was isolated *via* vacuum filtration as fine colourless needles (0.87 g, 1.97 mmol, 87%). **Mp** 192 – 194 °C. **^{1}H NMR** (500 MHz, $CDCl_3$) δ 7.93 (s, 2H, H_5), 3.11–3.05 (m, 12H, H_1). **^{13}C NMR (**126 MHz, $CDCl_3$) δ 165.3 (C_2), 141.2 (C_5), 133.7 (C_3), 128.7 (C_4), 37.2 (C_1). **HR-ESI-MS** *m/z* = 462.8753 $[M+Na]^+$ (calculated for $C_{12}H_{14}Br_2N_2O_2S_2Na^+$ = 462.8756) (σ <1).

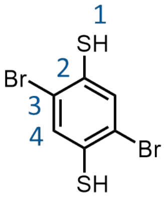

2,5-Dibromobenzene-1,4-dithiol (2c): To a small oven-dried round bottom flask was added *S,S'*-(2,5-dibromo-1,4-phenylene)*bis*(dimethylcarbamothioate) (265 mg, 0.60 mmol) and KOH (0.7 g, 12 mmol, 20 equiv) and MeOH (5 mL) was added and heated to reflux under an N_2-atmosphere for 3 h The reaction mixture was allowed to cool to rt before being poured onto ice cold 2 M HCl (5 mL) under N_2 and stirred for 15 min. The resulting colourless solid was isolated by vacuum filtration and washed extensively with water (5 × 30 mL), and dried under reduced pressure. The title product was isolated as a colourless solid (0.16 g, 0.53 mmol, 88%). **M.P**. 179 – 181 °C. **^{1}H NMR** (500 MHz, $CDCl_3$) δ 7.73 (s, 2H, H_4), 3.94 (m, 2H, H_1). **^{13}C NMR** (126 MHz, $CDCl_3$) δ 132.9 (C_4), 132.4 (C_2), 121.6 (C_3). **HR-ESI-MS** *m/z* = 296.8055 $[M-H]^-$ (calculated for $C_6H_3Br_2S_2^-$ = 296.8048) (σ 2). Compound **2c** is oxidised slowly in air in the solid state (ca. 1-2 days), much faster than the more robust **2a**, **2b** and **2d**.

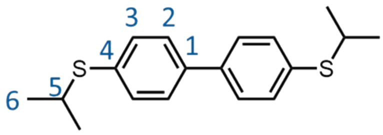

4,4'-*bis*(Isopropylthio)-1,1'-biphenyl: To a flame-dried Schlenk flask was added, sodium isopropylthiolate (1.00 g, 10.1 mmol, 4 equiv., **CARE!** *i*-PrSH is very malodorous) and 4,4'-diibromo-1,1'-biphenyl (0.80 g, 2.56 mmol) and evacuated through 3 × vacuum-N_2 cycles. Anhydrous DMA (6 mL) was deoxygenated (3 × freeze-pump-thaw cycles under N_2) and added to the reaction mixture then stirred at 110 °C for 20 h under N_2. The reaction mixture was allowed to cool to rt, poured onto brine (30 mL), and extracted with Et_2O (5 × 25 mL). The combined organic extracts were washed extensively with water (5 × 30 mL), dried ($MgSO_4$), filtered and the solvent was removed under reduced pressure to yield a colourless oil that crystallised on standing. The title product was isolated as a colourless crystalline solid (0.55 g, 1.82 mmol, 71%). **Mp** 146 – 148 °C [Lit.[14] 158 – 160 °C]. **^{1}H NMR** (500 MHz, $CDCl_3$) δ 7.52 (app. d, *J* = 8.3 Hz, 4H, H_2), 7.45 (app. d, *J* = 8.3 Hz, 4H, H_3), 3.42 (s, 2H, H_5), 1.34 (s, 12H, H_6). **^{13}C NMR** (126 MHz, $CDCl_3$) δ 138.8 (C_1), 135.0

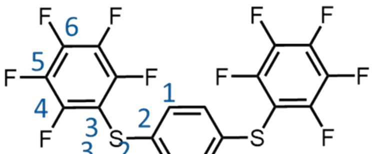

(C_4), 132.2 (C_3), 127.3 (C_2), 38.3 (C_5), 23.3 (C_6). **HR-ESI-MS** *m/z* = 303.1240 $[M+H]^+$ (calculated for $C_{18}H_{23}S_2^+$ = 303.1236) (σ 2). Literature compound, available spectroscopic data concordant.[15]

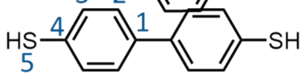

1,1'-Biphenyl-4,4'-dithiol (2d): To a flame-dried Schlenk flask was added, anhydrous DMA (1.5 mL) that had been deoxygenated (3 × freeze-pump-thaw cycles under N_2) and sodium metal (85 mg, 3.6 mmol, 4 equiv) then stirred at rt for 5 min under N_2. Solid 4,4'-*bis*(isopropylthio)-1,1'-biphenyl (0.28 g, 0.93 mmol) was added to the reaction mixture which was then stirred at 110 °C for 2.5 h under N_2. The reaction mixture was allowed to cool to rt before being pipetted onto ice cold 2 M HCl (5 mL) (**CARE!** Check all Na **has** been consumed in the reaction first) under a nitrogen atmosphere and the mixture stirred for 15 min. The resulting colourless solid was isolated by vacuum filtration and washed extensively with water (5 × 30 mL), and dried under reduced pressure. The title product was isolated without the need for further purification as colourless microcrystals (0.18 g, 0.82 mmol, 88%). **Mp** 192 – 194 °C [Lit.[15] 185 °C]. **^{1}H NMR** (500 MHz, $CDCl_3$) δ 7.41 (app. d, *J* = 8.3 Hz, 4H, H_2), 7.34 (app. d, *J* = 8.3 Hz, 4H, H_3), 3.48 (s, 2H, H_5). **^{13}C NMR** (126 MHz, $CDCl_3$) δ 138.0 (C_1), 130.1 (C_4), 130.0 (C_3), 127.6 (C_2). **HR-ESI-MS** *m/z* = 185.0420 $[M-SH]^+$ (calculated for $C_{12}H_9S^+$ = 185.0419) (σ <1). Literature compound, spectral data concordant.[15]

Thioether Linkers (3a-d)

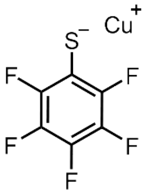

$CuSC_6F_5$: In a 100 mL flask fitted with a dropping funnel $CuSO_4·5H_2O$ (2.49 g, 10 mmol) was dissolved in deoxygenated water (35 mL) and vigorously stirred at rt. Neat pentafluorothiophenol (**CARE!** Corrosive) (2.10 g, 10.5 mmol) in a syringe was added dropwise over 20 min. A yellow precipitate immediately forms. Upon complete addition, the reaction mixture was further stirred for 30 min at rt. The resulting yellow precipitate was isolated by vacuum filtration and washed with water (20 mL), MeOH (5 mL), and Et_2O (5 mL). The yellow solid was then dried under vacuum at 40 °C overnight. The title product was isolated as a yellow solid (2.49 g, 9.50 mmol, 95%). **Mp** 270 – 271°C. NMR spectra could not be collected due to insolubility, but IR analysis was consistent with literature values.[16]

1,4-*bis*((Perfluorophenyl)thio)benzene (3a): To a small oven-dried Schlenk tube was added $CuSC_6F_5$ (11.7 g, 45 mmol), 1,4-diiodobenzene (5.3 g, 16 mmol) and anhydrous DMF (30 mL). The Schlenk tube was capped, and the mixture deoxygenated (3 × freeze-pump-thaw cycles under N_2) then stirred for 2.5 h at 145 °C. Upon cooling to rt, a 10% aqueous solution of HCl (25 mL) was

added. The mixture was extracted with Et_2O (5 × 25 mL) and the organic layers were combined and dried ($MgSO_4$), filtered and the solvent was removed under reduced pressure. The crude yellow solid was purified by column chromatography (SiO_2, hexane-CH_2Cl_2, 5%) and recrystallised from pentane at –20 °C. The title product was isolated as colourless needles (6.10 g, 12.9 mmol, 80%). **Mp** 153 – 154 °C [Lit.[17] 152 – 153 °C]. **^{1}H NMR** (500 MHz, $CDCl_3$) δ 7.24 (s, 4H, H_1). **^{13}C NMR** (126 MHz, $CDCl_3$) δ 148.7 – 146.7 (CF, app. d $^1J_{CF}$ = 248.5 Hz), 143.5 – 141.5 (CF, app. dtt, $^1J_{CF}$ = 255.9, $^2J_{CF}$ = 13.4, $^3J_{CF}$ = 5.0 Hz), 139.0 – 137.0 (CF, app. d $^1J_{CF}$ = 255.9 Hz), 133.3 (C_2), 131.2 (C_1), 108.2 (C_3, dt, $^4J_{CF}$ = 4.5, $^2J_{CF}$ = 20.9 Hz (not all J_{CF} couplings could be detected in this AA'MXX' system). **^{19}F NMR** (376 MHz, $CDCl_3$) δ –131.36 (m, 4F), –150.28 (t, J = 21.0 Hz, 2F), –159.86 (m, 4F); no C_6F_6 ref. **HR-ESI-MS** *m/z* = 496.9501 [M+Na]$^+$ (calculated for $C_{18}H_4F_{10}S_2Na$ = 496.9492) (σ 3). Literature compound, all data concordant.[18]

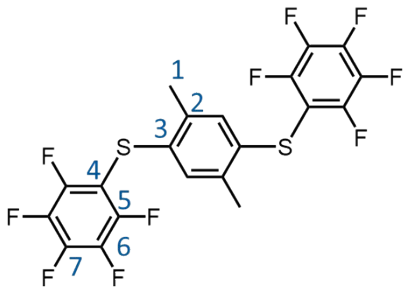

1,4-*bis*((Perfluorophenyl)thio)-2,5-dimethyl benzene (3b): To a flame-dried Schlenk flask was added, 1,4-diiodo-2,4-dimethylbenzene (0.40 g, 1.12 mmol) and $CuSC_6F_5$ (1.00 g, 3.82 mmol, 3.5 equiv) and evacuated through 3 × vacuum-N_2 cycles. Anhydrous DMF (2 mL) that had been deoxygenated (3 × freeze-pump-thaw cycles under N_2) was added to the reaction mixture which was then heated and stirred (145 °C for 3 h under N_2). Upon complete consumption of 1,4-diiodobenzene (as seen by TLC), the reaction mixture was allowed to cool to rt before being poured onto 1 M HCl (5 mL) and extracted with Et_2O (3 × 20 mL). The combined organic extracts were washed extensively with water (5 × 30 mL), dried over anhydrous $MgSO_4$, filtered and the solvent was removed under reduced pressure. The crude off-white solid was recrystallised from a supersaturated solution in hexane cooled to –20 °C. The title product was isolated as a colourless solid (0.52 g, 1.04 mmol, 92%). **Mp** 162 – 164 °C. **^{1}H NMR** (500 MHz, $CDCl_3$) δ 6.97 (s, 2H, H_4), 2.35 (s, 6H, H_1). **^{13}C NMR** (126 MHz, $CDCl_3$) δ 147.6 (CF, $^1J_{CF}$ = 237.4 Hz), 142.1 (CF, $^1J_{CF}$ = 256.8 Hz), 139.3 (C_2), 138.0 (CF, $^1J_{CF}$ = 256.0 Hz), 132.9 (C_4), 132.1 (C_3), 108.5 (C_5), 20.1 (C_1). **^{19}F NMR** δ –131.55, –151.05, –160.08; no C_6F_6 ref. **HR-ESI-MS** *m/z* = 501.9886 [M]$^+$ (calculated for $C_{20}H_8F_{10}S_2$ = 501.9902) (σ 3).

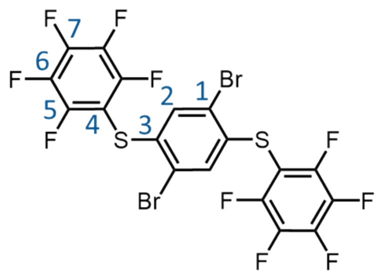

1,4-*bis*((Perfluorophenyl)thio)-2,5-dibromobenzene (3c): To a oven-dried Schlenk flask was added compound **3a** (0.50 g, 1.05 mmol), trifluoroacetic acid (8 mL), and conc. H_2SO_4 (2.5 mL) (**CARE!** corrosive mixture) and heated to 60 °C. Solid *N*-

bromosuccinimide (0.56 g, 3.15 mmol, 3 equiv) was added portion-wise over 6 h to the heated reaction mixture. Upon completion of the addition, the reaction was further heated at 60 °C for 48 h. After cooling the mixture to rt, it was added to ice-cold water (75 mL) and the resulting colourless precipitate isolated *via* vacuum filtration and washing with water (3 × 20 mL). The crude colourless solid was recrystallised from a supersaturated solution in EtOH to provide colourless needles on cooling to 4 °C (0.60 g, 0.95 mmol, 90%). **M.P.** 182 – 184°C. **^{1}H NMR** (500 MHz, $CDCl_3$) δ 7.12 (s, 2H, H_2). **^{13}C NMR** (126 MHz, $CDCl_3$) δ 147.7 (CF, $^{1}J_{CF}$ = 250.6 Hz), 143.1 (CF, $^{1}J_{CF}$ = 259.6 Hz), 138.2 (CF, $^{1}J_{CF}$ = 256.8 Hz), 135.4 (C_3), 133.2 (C_2), 122.8 (C_1), 106.2 (C_4). **^{19}F NMR** δ –130.15, –148.29, –158.81; no C_6F_6 ref. **HR-ESI-MS** *m/z* = 628.7751 $[M-H]^-$ (calculated for $C_{18}HBr_2F_{10}S_2^-$ = 628.7732) (σ 3).

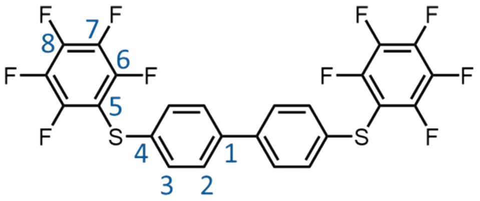

4,4'-*bis*((Perfluorophenyl)thio)-1,1'-biphenyl (3d): To a small oven-dried Schlenk tube was charged with $CuSC_6F_5$ (1.57 g, 5.98 mmol, 3 equiv), 4,4'-diiodo-1,1'-biphenyl (0.81 g, 2.00 mmol), and anhydrous DMF (3 mL). The Schlenk tube was capped, and the mixture deoxygenated (3 × freeze-pump-thaw cycles under N_2) then stirred for 2.5 h at 145 °C. Upon cooling to rt, a 10% aqueous solution of HCl (5 mL) was added. The mixture was extracted with Et_2O (5 × 10 mL) and the organic layers were combined and dried over anhydrous $MgSO_4$, filtered and the solvent was removed under reduced pressure. The crude yellow solid was purified by column chromatography (SiO_2, hexane-CH_2Cl_2) and the combined product fractions were evaporated and the resulting solid was washed with hot pentane. The title product was isolated as a colourless solid (0.79 g, 1.44 mmol, 72%). **Mp** 199 – 201 °C. **^{1}H NMR** (500 MHz, $CDCl_3$) δ 7.45 (m, 4H, H_3), 7.41 (m, 4H, H_2). **^{13}C NMR** (126 MHz, $CDCl_3$) δ 147.7 (CF, $^{1}J_{CF}$ = 248.7 Hz), 142.3 (CF, $^{1}J_{CF}$ = 257.0 Hz), 139.9 (C_1) 138.0 (CF, $^{1}J_{CF}$ = 258.8 Hz), 132.6 (C_4), 131.1 (C_2), 128.1 (C_3), 108.9. **^{19}F NMR** δ –131.49, –150.89, –160.14; no C_6F_6 ref. **HR-ESI-MS MS** *m/z* = 548.9858 $[M-H]^+$ (calculated for $C_{24}H_7F_{10}S_2^+$ = 548.9824) (σ 6).

Symmetric Macrocycles (1a-d)

Synthetic notes: Anhydrous solvent was used throughout for consistency and all aryl-F carbons are reported as chemical shifts based on their $^{13}C\{^{19}F\}$ decoupled spectra.

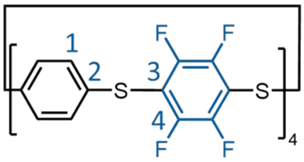

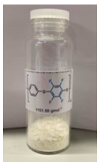

S_8-Corona[4H,4F]arene (1a): To an oven-dried Schlenk tube was added, benzene-1,4-dithiol (**2a**) (1.42 g, 9.98 mmol), tetramethylammonium fluoride tetrahydrate (66 mg, 0.4 mmol, 5 mol% vs. **3a**) and deoxygenated through 3 × vacuum-N_2 cycles. Deoxygenated pyridine

(50 mL) was added to the reaction mixture and stirred for 10 min under an N_2 atmosphere. A solution of **3a** (3.78 g, 7.97 mmol) in pyridine (50 mL) was deoxygenated (3 × freeze-pump-thaw cycles under N_2) before being added dropwise at rt to the stirring mixture using a syringe pump over 10 mins. Upon complete addition the reaction was stirred for a further 0.5 h before the solvent was removed under reduced pressure (water bath temp <35 °C) to yield a crude yellow solid. The crude material was dissolved in $CHCl_3$ (150 mL) and filtered to remove polymeric insoluble material. The organic soluble mixture was successively washed with water (3 × 150 mL), then brine (150 mL), dried ($MgSO_4$), filtered and the solvent was removed under reduced pressure. The resulting solid was washed with warm pentane (3 × 100 mL) to give the title compound as a colourless solid (1.30 g, 1.13 mmol, 28% based on **3a**). **Mp** >300 °C. **^{1}H NMR** (500 MHz, $CDCl_3$) δ 7.31 (s, 16H, H_1). **^{13}C NMR (**126 MHz, $CDCl_3$) δ 146.4 (C_4), 132.6 (C_2), 131.4 (C_1), 115.5 (C_3). **^{19}F NMR** (376 MHz, $CDCl_3$) δ –134.50 (s, 16F). **MALDI-MS** *m/z* = 1152.0 $[M]^+$ (calculated for $C_{48}H_{16}F_{16}S_8^+$ = 1151.9, S_8 isotope pattern observed) for most abundant isotope peak (see Figures S134-135 for MALDI spectra).

Equivalent behaviour is observed using a NH_4F catalyst, except that S_8-Corona[4H,4F]arene (**1a**) is produced entirely specifically. Washing with pentane is normally sufficient to purify all the macrocycles **1**, but washing with hot hexanes is also effective, as is dissolution of crude **1** mixtures in CH_2Cl_2 or $CHCl_3$ followed by their precipitation by slow addition of pentane and then trituration of that mixture. Chromatography on SiO_2 was not found to be effective; **1** is strongly retained.

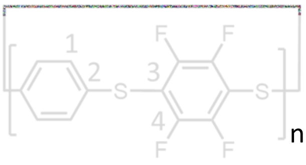

n = 4 (major), 6 (minor)

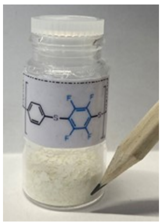

One-pot synthesis of S_8-corona[4H,4F]arene (1a) and S_{12}-corona[4H,4F]arene (1a') mixtures (88:12 ratio): To an oven-dried Schlenk tube was added, benzene-1,4-dithiol **2a** (0.55 g, 3.9 mmol), tetramethylammonium fluoride tetrahydrate (0.68 g, 4.1 mmol, 1.05 equiv, a catalytic protocol may also be used) and deoxygenated through 3 × vacuum-N_2 cycles. Deoxygenated pyridine (35 mL) was added to the reaction mixture and stirred for 10 min under an N_2 atmosphere. A solution of C_6F_6 (470 µL, 4.1 mmol) in pyridine (35 mL) was deoxygenated (3 × freeze-pump-thaw cycles under N_2) before being added dropwise to the stirring mixture using a syringe pump over 10 mins at rt. Upon complete addition, the reaction was stirred for a further 0.5 h before the solvent was removed under reduced pressure (water bath temp <35 °C) to yield a crude yellow solid. The crude material was dissolved in $CHCl_3$ (20 mL) and filtered to remove polymeric insoluble material. The organic mixture was successively washed with 1 M HCl (2 × 30 mL), water (30 mL), brine (30 mL), dried

($MgSO_4$), filtered and the solvent removed under reduced pressure. The resulting solid was washed with warm pentane (3 × 20 mL) to give a mixture of **S_8-corona[4H,4F]arene** (**1a**) and **S_{12}-corona[4H,4F]arene** (**1a'**) (0.35 g, 1:0.14 ratio, 27% overall based on **2a**). A small amount of the mixture was purified via sublimation (300 °C, 1 mbar) to collect analytical data on the major component **1a**: **Mp** >300 °C. **^{1}H NMR** (500 MHz, $CDCl_3$) δ 7.31 (s, 16H, H_1). **^{13}C NMR** (126 MHz, $CDCl_3$) δ 146.4 (C_4), 132.6 (C_2), 131.4 (C_1), 115.5 (C_3). **^{19}F NMR** (376 MHz, $CDCl_3$) δ −134.50 (s, 16F). **MALDI-MS** *m/z* = 1152.0 $[M]^+$ (calculated for $C_{48}H_{16}F_{16}S_8^+$ = 1151.88) (see Figure S134). For **1a'**: **^{19}F NMR** (376 MHz, $CDCl_3$) δ −134.41 (s, 24F). **MALDI-MS** *m/z* = 1727.9 $[M]^+$ (calculated for $C_{72}H_{24}F_{24}S_{12}^+$ = 1727.8, S_{12} isotope pattern observed) for the most abundant isotope peak. See Figures S134-138 for MALDI spectra, isotope patterns and simulated isotope patterns. The one-pot procedure is useful for production of 0.1 to ca. 0.5 g mixtures of **1a/1a'** (yields of 26-31% were realised in repeated runs). Above such scales use of the fragment approach was preferred.

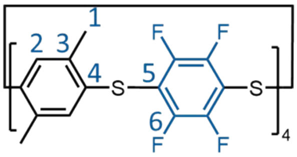

S_8-Corona[4(2,5-dimethyl),4F]arene (1b): To an oven-dried Schlenk tube was added, **2b** (0.23 g, 1.35 mmol), tetramethylammonium fluoride tetrahydrate (9 mg, 54 µmol, 5 mol%) and deoxygenated through 3 × vacuum-N_2 cycles. Deoxygenated pyridine (10 mL) was added to the reaction mixture and stirred for 10 min under an N_2 atmosphere. A solution of **3b** (0.54 g, 1.08 mmol) in deoxygenated pyridine (10 mL, 3 × freeze-pump-thaw cycles under N_2) was added dropwise at rt to the stirred reaction mixture using a syringe pump over 10 mins. Upon complete addition, the reaction was stirred for a further 0.5 h before the solvent was removed under reduced pressure (water bath temp <35 °C) to yield a crude yellow solid. The crude material was dissolved in $CHCl_3$ (50 mL) and filtered to remove polymeric insoluble material. The organic soluble mixture was successively washed with water (3 × 15 mL), brine (15 mL), dried over anhydrous $MgSO_4$, filtered and the solvent was removed under reduced pressure. The resulting solid was washed with warm pentane (3 × 10 mL) to give the title compound as a colourless solid (0.14 g, 0.11 mmol, 19%). **Mp** >300 °C. **^{1}H NMR** (500 MHz, $CDCl_3$) δ 7.08 (s, 8H, H_2), 2.32 (s, 24H, H_1). **^{13}C NMR** (126 MHz, $CDCl_3$) δ 146.49 (C_6), 138.1 (C_3), 133.9 (C_2), 131.9 (C_4), 114.7 (C_5), 20.2 (C_1). **^{19}F NMR** (376 MHz, $CDCl_3$) δ -135.40 (s, 16F). **MALDI-MS** *m/z* = 1264.01 $[M]^+$ (calculated for $C_{56}H_{32}F_{16}S_8^+$ = 1264.00, S_8 isotope pattern observed) for the most abundant isotope peak. See Figures S150-151 for MALDI spectrum, isotope pattern and simulated isotope pattern).

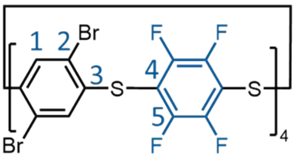

S_8-Corona[4(2,5-dibromo),4F]arene (1c): To an oven-dried Schlenk tube was added freshly prepared 2,5-bromobenzene-1,4-dithiol (**2c**) (60

mg, 0.20 mmol, 1.25 equiv), tetramethylammonium fluoride tetrahydrate (1.4 mg, 8.5 µmol, 5 mol%) and deoxygenated through 3 × vacuum-N_2 cycles. Deoxygenated anhydrous pyridine (2 mL) was added to the reaction mixture and stirred for 10 min under an N_2 atmosphere. A solution of **3c** (107 mg, 0.17 mmol) in anhydrous pyridine (2 mL) was deoxygenated (3 × freeze-pump-thaw cycles under N_2) before being added dropwise at rt to the stirring anaerobic reaction mixture by syringe pump over 10 mins. Upon complete addition, the reaction was stirred for a further 0.5 h before the solvent was removed under reduced pressure (water bath temp <35 °C) to yield a crude yellow solid. The crude material was dissolved in $CHCl_3$ (50 mL) and filtered to remove polymeric insoluble material. The organic soluble mixture was successively washed with water (3 × 15 mL), brine (15 mL), dried over anhydrous $MgSO_4$, filtered and the solvent removed under reduced pressure. The resulting solid was washed with warm pentane (3 × 10 mL) to give the title compound as a colourless solid (10.7 mg, 6.0 µmol, 7%). **Mp** > 300 °C. **^{1}H NMR** (500 MHz, $CDCl_3$) δ 7.40 (s, 8H, H_1). **^{13}C NMR** (126 MHz, $CDCl_3$) δ 146.8 (C_5), 134.5 (C_1), 135.0 (C_3), 124.3 (C_2), 114.0 (C_4). **^{19}F NMR** (376 MHz, $CDCl_3$) δ -133.70 (s, 16F). **MALDI-MS** *m/z* = 1783.89 $[M]^+$ (calculated for $C_{48}H_8Br_8F_{16}S_8^+$ = 1783.15, S_8Br_8 isotope pattern observed) for the most abundant isotope peak. See Figures S160-161 for MALDI spectrum, isotope pattern and simulated isotope pattern).

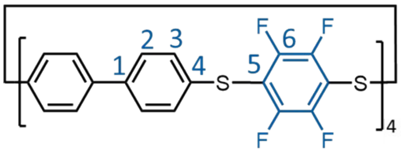

S_8-Corona[4(1,1’-biphenyl)4F]arene (1d): To an oven-dried Schlenk tube was added the 1,1'-biphenyl]-4,4'-dithiol (**2d**) (0.10 g, 0.46 mmol, 1.25 mmol), tetrabutylammonium fluoride trihydrate (6.3 mg, 0.02 mmol, 5 mol%) and deoxygenated through 3 × vacuum-N_2 cycles. Deoxygenated anhydrous pyridine (10 mL) was added to the reaction mixture and stirred for 10 min under an N_2 atmosphere. A solution of **3b** (0.20 g, 0.37 mmol) in anhydrous pyridine (10 mL) was deoxygenated (3 × freeze-pump-thaw cycles under N_2) before being added dropwise at rt under $N_2$to the stirring reaction mixture using a syringe pump over 10 mins. Upon complete addition, the reaction was stirred for a further 0.5 h before the solvent was removed under reduced pressure (water bath temp <35 °C) to yield a crude yellow solid. The crude material was dissolved in $CHCl_3$ (100 mL) and filtered through filter paper to remove polymeric insoluble material. The organic soluble mixture was successively washed with a saturated aqueous solution of NH_4Cl (3 × 20 mL), water (3 × 15 mL), and brine (15 mL), and dried over anhydrous $MgSO_4$, filtered and the solvent was removed under reduced pressure. The resulting solid was washed with warm Et_2O (3 × 10 mL) and then warm pentane (3 × 10 mL) to give the title compound chemoselectively as a colourless solid (0.12 g, 0.08 mmol,

45%). **Mp** >300 °C. **^{1}H NMR** (500 MHz, $CDCl_3$) δ 7.45 (s, 32H, H_{2+3}). **^{13}C NMR** (126 MHz, $CDCl_3$) δ 147.1 (C_6), 140.0 (C_1), 132.2 (C_4), 131.8 (C_3), 128.1 (C_2), 115.5 (C_5). **^{19}F NMR** (376 MHz, $CDCl_3$, with C_6F_6 reference) δ –134.92. ^{13}C NMR (126 MHz, $CDCl_3$) δ 147.1(C_6), 140.0(C_1), 132.2(C_4), 131.8(C_3), 128.0(C_2), 115.5(C_5). **MALDI-MS** *m/z* = 1455.87 $[M]^+$ (calculated for $C_{72}H_{32}F_{16}S_8^+$ = 1456.00, S_8 isotope pattern observed), for the most abundant isotope peak. See Figures S165-167 for MALDI spectrum, isotope pattern and simulated isotope pattern).

Mixed Aryl Macrocycles (1ab-cd)

Synthetic notes: Anhydrous solvent was used throughout for consistency and all aryl-F carbons are reported as chemical shifts based on their ^{13}C{^{19}F) decoupled spectra.

General Procedure: To an oven-dried Schlenk tube was added, **2a-d** (0.35 mmol) and catalytic template (0.018 mmol, 5 mol%) and deoxygenated through 3 × vacuum-N_2 cycles. Deoxygenated anhydrous pyridine (6 mL) was added to the reaction mixture and stirred for 10 min under an N_2 atmosphere (note: deoxygenated and anhydrous solvent used only for consistency). A solution of **3a-3d** (0.32 mmol) in anhydrous pyridine (6 mL) was deoxygenated (3 × freeze-pump-thaw cycles under N_2) before being added dropwise over 10 mins at rt under N_2 to the stirred reaction mixture using a syringe pump. Upon complete addition, the reaction was stirred for a further 0.5 h before the solvent was removed under reduced pressure (water bath temp <35 °C) to yield a crude yellow solid. The crude material was dissolved in $CHCl_3$ and filtered to remove polymeric insoluble material. The organic soluble mixture was successively washed with water (3 × 15 mL), brine (15 mL), dried over anhydrous $MgSO_4$, filtered and the solvent was removed under reduced pressure. The resulting solid was washed with warm pentane (3 × 10 mL) to give the title compounds as colourless solids.

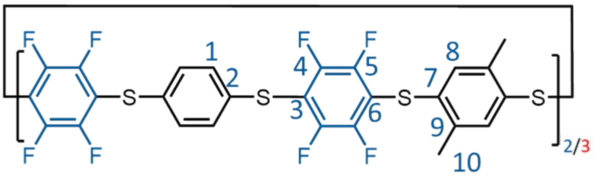

Corona macrocycle (1ab): Template = tetramethyl ammonium fluoride tetrahydrate. The title product was isolated as a colourless solid (60.4 mg, 0.05 mmol, 28%) which was a 88:12 mixture of the S_8 and S_{12} macrocycles. **Mp** >300 °C. **^{1}H NMR** (500 MHz, $CDCl_3$) δ 7.30 (s, 8H, H_1), 7.07 (s, 4H, H_8), 2.33 (s, 12H, H_{10}). **^{13}C NMR** (126 MHz, $CDCl_3$) δ 147.9 (m, C_4 or C_5), 146.1 (m, C_4 or C_5), 138.2 (C_9), 133.9 (C_8), 133.1 (C_2), 131.8 (C_1), 131.5 (C_7), 115.7 (C_3 or C_6), 114.2 (C_3 or C_6), 20.2 (C_{10}). **^{19}F NMR** (376 MHz, $CDCl_3$) δ –132.0 — –131.9 (m (A/B), $F_{4 \text{ or } 5}$), –131.6 — –131.5 (m, (A/B), $F_{4 \text{ or } 5}$). (Only major peaks described here, see Figures S41-45 for detailed spectra). **MALDI-**

MS *m/z* = 1208.22 $[M]^+$ (calculated for $C_{52}H_{24}F_{16}S_8^+$ = 1208.946 (n = 2), S_8 isotope pattern observed) and *m/z* = 1812.220 $[M]^+$ (calculated for $C_{78}H_{36}F_{24}S_{12}^+$ = 1812.916 (n = 3), S_{12} isotope pattern observed). The most abundant isotope peak is picked in each case. See Figures S139-143 for MALDI spectra, isotope patterns and simulated isotope patterns).

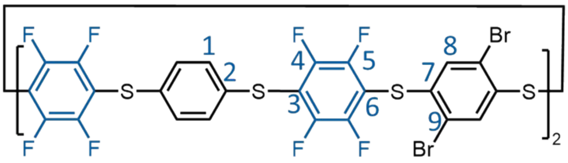

Corona macrocycle (1ac): Template = tetramethyl ammonium fluoride tetrahydrate. The title product was isolated as a colourless solid (61.7 mg, 0.04 mmol, 24%). **Mp** >300 °C. **^{1}H NMR** (500 MHz, $CDCl_3$) δ 7.32 (s, 8H, H_1), 7.31 (s, 4H, H_8). **^{13}C NMR** (126 MHz, $CDCl_3$) δ 147.1 (C_4), 146.9 (C_5) 135.0 (C_7), 132.9 (C_2), 131.9 (C_1), 131.7 (C_8), 124.1 (C_9), 116.2 (C_3), 113.2 (C_6). **^{19}F NMR** (376 MHz, $CDCl_3$) δ –133.8 (bs, F_{4+5}). The same signal was observed at δ –130.7 using the intrinsic internal spectrometer referencing vs. referencing to external C_6F_6. **MALDI-MS** *m/z* = 1469.873 $[M]^+$ (calculated for $C_{48}H_{12}Br_4F_{16}S_8^+$ = 1468.522, S_8Br_4 isotope pattern observed) for the most abundant isotope peak. See Figures S144-146 for MALDI spectrum, isotope pattern and simulated isotope pattern.

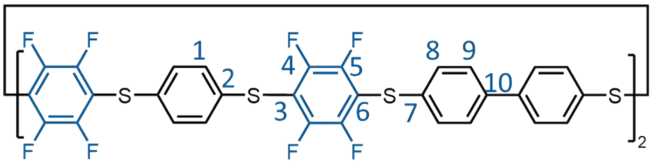

Corona macrocycle (1ad): Template = tetrabutyl ammonium fluoride trihydrate. The title product was isolated as a colourless solid (82.2 mg, 0.063 mmol, 36%). **Mp** >300 °C. **^{1}H NMR** (500 MHz, $CDCl_3$) δ 7.46 (m, 16H, H_{8+9}), 7.29 (s, 8H, H_1). **^{13}C NMR** (126 MHz, $CDCl_3$) δ 148.1, 146.1, 140.1 (C_{10}), 133.0 (C_2), 132.0 ($C_{8\ or\ 9}$), 131.9 (C), 131.8 (C_1), 128.1 ($C_{8\ or\ 9}$), 116.1, 114.4. **^{19}F NMR** (376 MHz, $CDCl_3$) δ -131.5 (s, F_{4+5}). **MALDI-MS** *m/z* = 1304.2 $[M]^+$ (calculated for $C_{60}H_{24}F_{16}S_8^+$ = 1304.9, S_8 isotope pattern observed) for the most abundant isotope peak. See Figures S147-149 for MALDI spectrum, isotope pattern and simulated isotope pattern.

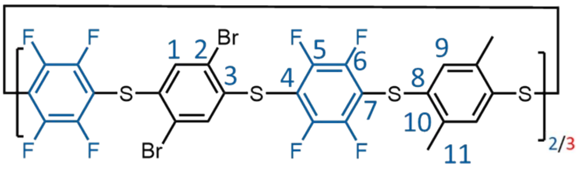

Corona macrocycle (1bc): Template = tetramethyl ammonium fluoride tetrahydrate. The title product was isolated as a colourless solid (42.7 mg, 0.028 mmol, 16%) which was a 75:25 mixture of the S_8 and S_{12} macrocycles. **Mp** >300 °C. **^{1}H NMR** (500 MHz, $CDCl_3$) δ 7.34 (s, 4H, H_1), 7.12 (s, 4H, H_9), 2.32 (s, 12H, H_{11}). **^{13}C NMR** (126 MHz, $CDCl_3$) δ 147.0 ($C_{5\ or\ 6}$), 146.9 ($C_{5\ or\ 6}$), 138.2 (C_{10}), 135.2 (C_3), 135.2 (C_1), 134.0 (C_9), 131.7 (C_8), 124.1 (C_2), 116.7 ($C_{4\ or\ 7}$), 112.1 ($C_{4\ or\ 7}$), 20.2 (C_{11}). **^{19}F NMR** (376 MHz, $CDCl_3$) δ –134.76 (m, $F_{5\ or\ 6}$), –134.76 (m, $F_{5\ or\ 6}$). (Only major peaks described here, see Figures S61—S65 for detailed spectra). **MALDI-MS** *m/z* = 1523.462 $[M]^+$

(calculated for $C_{52}H_{20}Br_4F_{16}S_8^+$ = 1523.589 (n = 2), S_8Br_4 isotope pattern observed) and *m/z* = 2289.089 (calculated for $C_{78}H_{30}Br_6F_{24}S_{12}^+$ = 2288.372 (n = 3), $S_{12}Br_6$ isotope pattern observed). Both based on the most abundant isotope peak, see Figures S152-156 for MALDI spectrum, isotope patterns and simulated isotope patterns.

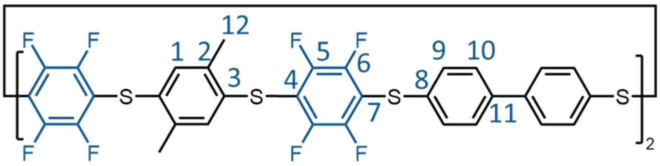

Corona macrocycle (1bd): Template = tetrabutyl ammonium fluoride trihydrate. The title product was isolated as an off white solid (76.2 mg, 0.056 mmol, 32%). **Mp** >300 °C. **^{1}H NMR** (500 MHz, $CDCl_3$) δ 7.47 (m, 8H, H_{9+10}), 7.09 (s, 4H, H_1), 2.32 (s, 12H, H_{11}). **^{13}C NMR** (126 MHz, $CDCl_3$) δ 147.1 ($_{C5+6}$), 147.0 (C_{5+6}), 140.0 (C_{11}), 138.1 (C_2), 133.8 (C_1), 132.2 (C_8), 131.9 (C_3), 131.7 (C_9), 128.1 (C_{10}), 115.2 ($C_{4\ or}\ C_7$), 115.1 ($C_{4\ or}\ C_7$), 20.3 (C_{12}). **^{19}F NMR** (376 MHz, $CDCl_3$) δ –134.84 — –135.03 (m, A/B, $F_{5\ or\ 6}$), -135.13 — -135.32 (m, A/B, $F_{5\ +\ 6}$). The same signals were observed at δ –132.16 — –131.97 using the intrinsic internal spectrometer referencing vs. referencing to external C_6F_6. **MALDI-MS** *m/z* = 1360.056 [M]$^+$ (calculated for $C_{64}H_{32}F_{16}S_8^+$ = 1361.009, S_8 isotope pattern observed) for the most abundant isotope peak. See Figures S157-159 for MALDI spectrum, isotope pattern and simulated isotope pattern.

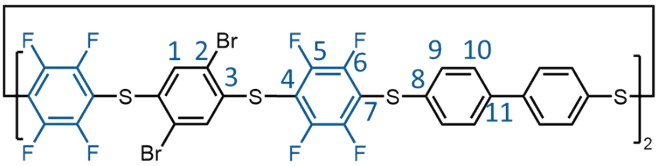

Corona macrocycle (1cd): Template = tetrabutyl ammonium fluoride trihydrate. The title product was isolated as an off white solid (90.7 mg, 0.056 mmol, 32%). **Mp** > 300 °C. **^{1}H NMR** (500 MHz, $CDCl_3$) δ 7.49 — 7.44 (m, 20H, H_{1+9+10}). **^{13}C NMR** (126 MHz, $CDCl_3$) δ 147.2 (C_5 or C_6), 147.0 (C_5 or C_6), 140.0 (2 peaks), 134.7, 131.7, 131.6, 128.0, 123.3, 117.2 (2 peaks, C_4 and C_7). Unambiguous assignment of some carbons could not be made. **^{19}F NMR** (376 MHz, $CDCl_3$) δ –130.9 — –130.7 (m, XF). **MALDI-MS** *m/z* = 1620.073 [M]$^+$ (calculated for $C_{60}H_{20}Br_4F_{16}S_8^+$ = 1620.585, S_8 isotope pattern observed), for the most abundant isotope peak. See Figures S162-164 for MALDI spectrum, isotope pattern and simulated isotope pattern.

Substituted Macrocycles (4-6)

Synthetic notes: Anhydrous solvent was used throughout for consistency and all aryl-F carbons are reported as chemical shifts based on their $^{13}C\{^{19}F\}$ decoupled spectra.

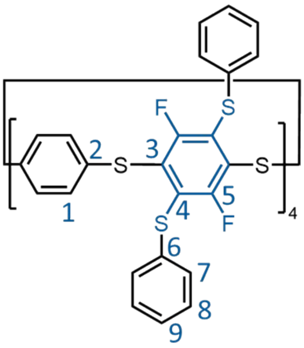

Thiophenol substituted corona macrocyle (4a): To an oven-dried Schlenk flask was added corona macrocycle **1a** (50 mg, 43.4 µmol), tetramethylammonium fluoride tetrahydrate (2 mg, 12.1 µmol, 0.28 equiv.) and pyridine (3 mL) and the mixture deoxygenated (3 × freeze-pump-thaw cycles under N_2). Thiophenol (71 µL, 0.70 mmol, 16 equiv) was added and the mixture was stirred at rt under an N_2-atmosphere for 2 h. The mixture was poured onto 0.5 M HCl (5 mL) and extracted with CH_2Cl_2 (3 × 10 mL). The combined organic phase was then washed with water (3 × 5 mL), brine (10 mL), dried ($MgSO_4$), filtered and evaporated under reduced pressure. The resulting solid was washed with Et_2O (3 × 5 mL) and warm pentane (5 mL) to give the title compound as a colorless solid (79.5 mg, 42.5 µmol, 98%). **Mp** >300 °C. **^{1}H NMR** (500 MHz, $CDCl_3$) δ 7.14 (m, 40H, H_{7+8+9}), 7.03 (s, 16H, H_1). **^{13}C NMR** (126 MHz, $CDCl_3$) δ 159.3 (C_5), 134.6 (C_6), 133.8 (C_2), 130.8 (C_1), 129.8 ($C_{7 \text{ or } 8 \text{ or } 9}$), 129.4 ($C_{7 \text{ or } 8 \text{ or } 9}$), 129.1 ($C_{2 \text{ or } 3}$), 128.9 ($C_{2 \text{ or } 3}$), 127.4 ($C_{7 \text{ or } 8 \text{ or } 9}$). **^{19}F NMR** (376 MHz, $CDCl_3$) δ –93.9 (s, F_5). **MALDI-MS** *m/z* = 1874.178 $[M]^+$ (calculated for $C_{96}H_{56}F_8S_{16}^+$ = 1874.987, S_{16} isotope pattern observed) for the most abundant isotope peak. See Figures S168-170 for MALDI spectrum, isotope pattern and simulated isotope pattern.

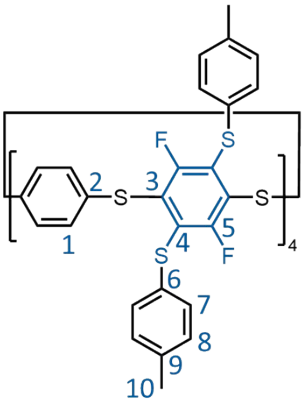

4-Methylthiophenol substituted corona macrocycle (5a): To an oven-dried Schlenk flask was added corona macrocycle **1a** (5 mg, 4.34 µmol), tetramethylammonium fluoride tetrahydrate (1 mg, 6.1 µmol, 1.4 equiv) and pyridine (0.7 mL) and deoxygenated (3 × freeze-pump-thaw cycles under N_2). Next, 4-methylthiophenol (9 mg, 0.07 mmol, 16 equiv) was added and the mixture stirred under an N_2 atmosphere for 4 h at rt. The mixture was poured onto 0.5 M HCl (1 mL) and extracted with CH_2Cl_2 (3 × 5 mL). The combined organic phase was washed with water (3 × 5 mL), brine (10 mL), dried ($MgSO_4$), filtered and the solvent evaporated under reduced pressure. The resulting solid was washed with Et_2O (3 × 3 mL) and warm pentane (2 mL) to give the title compound as a colourless solid (8.2 mg, 4.1 µmol, 95%). **Mp** >300 °C. **^{1}H NMR** (500 MHz, $CDCl_3$) δ 7.06 (d, *J* = 8.3 Hz, 16H, H_7), 7.02 (s, 16H, H_1), 7.01 (d, *J* = 8.3 Hz, 16H, H_8), 2.26 (s, 24H, H_{10}). **^{13}C NMR** (126 MHz, $CDCl_3$) δ 159.1 (C_5), 137.7 (C_9), 133.8 (C_6), 130.9 (C_2), 130.7 (C_1), 130.4 (C_7), 130.1 (C_8), 129.5 (C_{3or4}), 128.5 (C_{3or4}). **^{19}F NMR** (376 MHz, $CDCl_3$) δ –94.57 (s, F_5).

MALDI-MS *m/z* = 1986.282 $[M]^+$ (calculated for $C_{104}H_{72}F_8S_{16}^+$ = 1986.114, S_{16} isotope pattern observed) for the most abundant isotope peak. See Figures S177-179 for MALDI spectrum, isotope pattern and simulated isotope pattern.

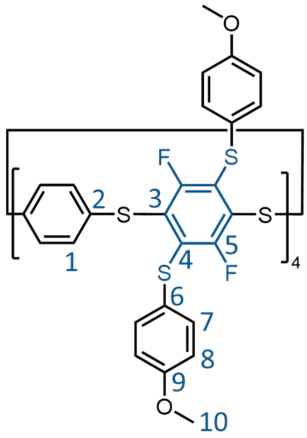

4-Methoxythiophenol substituted corona macrocycle (5b): To an oven-dried Schlenk flask was added corona macrocycle **1a** (10.0 mg, 8.69 µmol), 4-methoxythiophenol (17.2 µL, 0.14 mmol, 16 equiv) and THF (1 mL) and deoxygenated (3 × freeze-pump-thaw cycles under N_2). Next, 1,1,3,3-tetramethylguanidine (TMG) (50 µL, 0.28 mmol, 32 equiv) was added and the mixture was stirred at rt under an N_2-atmosphere for 2 h. The mixture was poured onto 0.5 M HCl (5 mL) and extracted with CH_2Cl_2 (3 × 5 mL). The combined organic phase was then washed with water (3 × 5 mL), brine (10 mL), dried ($MgSO_4$), filtered and the solvent removed under reduced pressure. The resulting solid was washed with Et_2O (3 × 5 mL) and warm pentane (5 mL) to give the title compound as a colourless solid (17.8 mg, 8.42 µmol, 97%). **Mp** >300 °C. **^{1}H NMR** (500 MHz, $CDCl_3$) δ 7.17 (d, *J* = 8.8 Hz, 16H, H_8), 7.00 (s, 16H, H_1), 6.70 (d, *J* = 8.8 Hz, 16H, H_7), 3.70 (s, 24H, H_{10}). **^{13}C NMR** (126 MHz, $CDCl_3$) δ 159.7 (C_9), 133.9 (C_2), 133.5 (C_7), 130.5 (C_1), 124.7 (C_6), 114.9 (C_8), 55.5 (C_{10}). **^{19}F NMR** (376 MHz, $CDCl_3$) δ –95.43 (s, F_5). **MALDI-MS** *m/z* = 2113.966 $[M]^+$ (calculated for $C_{104}H_{72}F_8O_8S_{16}^+$ = 2115.072, S_{16} isotope pattern observed) for the most abundant isotope peak. See Figures S180-182 for MALDI spectrum, isotope pattern and simulated isotope pattern.

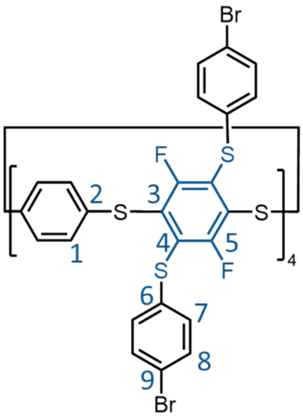

4-Bromothiophenol substituted corona macrocycle (5c): To an oven-dried Schlenk flask was added corona macrocycle **1a** (10.0 mg, 8.69 µmol), 4-bromothiophenol (23 mg, 0.12 mmol, 14 equiv), and THF (1 mL) and deoxygenated (3 × freeze-pump-thaw cycles under argon). Next, 1,1,3,3-tetramethylguanidine (TMG) (22 µL, 0.18 mmol, 32 equiv) was added and the mixture was stirred at rt under an argon atmosphere for 4 h. The mixture was poured onto 0.5 M HCl (5 mL) and extracted with CH_2Cl_2 (3 × 5 mL). The combined organic phase was then washed with water (3 × 5 mL), brine (10 mL), dried over anhydrous $MgSO_4$, filtered and the solvent removed under reduced pressure. The resulting solid was washed with Et_2O (3 × 5 mL) and warm pentane (5 mL) before being purified by preparative TLC (SiO_2, hexane-CH_2Cl_2 40%) to give the title compound as a colourless solid (10.2 mg, 4.08 µmol, 47%). **Mp** >300 °C. **^{1}H NMR** (500 MHz,

$CDCl_3$) δ 7.30 (d, *J* = 8.4 Hz, 16H, H_8), 7.02 (s, 16H, H_1), 6.99 (d, *J* = 8.4 Hz, 16H, H_7). **^{13}C NMR** (126 MHz, $CDCl_3$) δ 133.8 (C_6), 132.5 (C_8), 132.3 (C_2), 131.7 (C_1), 130.8 (C_7), 121.7 (C_9). **^{19}F NMR** (376 MHz, $CDCl_3$) δ –94.14 (s, F_5). **MALDI-MS** *m/z* = 2509.819 $[M]^+$ (calculated for $C_{96}H_{48}F_8S_{16}Br_8^+$ = 2506.262, $S_{16}Br_8$ isotope pattern observed), for the most abundant isotope peak. See Figures S183-185 for MALDI spectrum, isotope pattern and simulated isotope pattern.

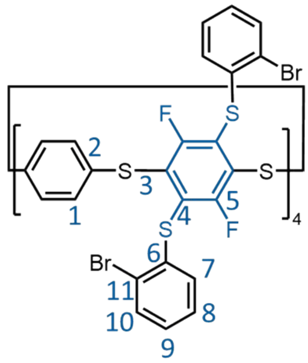

2-Bromothiophenol substituted corona macrocycle (5d): To an oven-dried Schlenk flask was added corona mcactocycle **1a** (10.0 mg, 8.69 µmol), 2-bromothiophenol (23 mg, 0.12 mmol, 14 equiv), and THF (1 mL) and deoxygenated (3 × freeze-pump-thaw cycles under argon). Next, 1,1,3,3-tetramethylguanidine (TMG) (22 µL, 0.18 mmol, 32 equiv) was added and the mixture was stirred at rt under an argon atmosphere for 4 h. The mixture was poured onto 0.5 M HCl (5 mL) and extracted with CH_2Cl_2 (3 × 5 mL). The combined organic phase was then washed with water (3 × 5 mL), brine (10 mL), dried over anhydrous $MgSO_4$, filtered and the solvent was removed under reduced pressure. The resulting solid was washed with Et_2O (3 × 5 mL) and warm pentane (5 mL) and further purified by preparative TLC (SiO_2, hexane-CH_2Cl_2 40%) to give the title compound as a colourless solid (13.5 mg, 5.39 µmol, 62%). **Mp** >300 °C. **^{1}H NMR** (500 MHz, $CDCl_3$) δ 7.48 (d, *J* = 8.5 Hz, 8H, H_{10}), 7.09 (m, 24H, H_{1+8}), 7.02 (m, 8H, H_9), 6.84 (d, *J* = 8.4 Hz, 8H, H_7). **^{13}C NMR** (126 MHz, $CDCl_3$) δ 159.2 (C_5), 135.5 (C_6), 133.5 (C_2), 133.5 (C_{10}), 131.1 (C_1), 129.8 (C_7), 128.8 (C_3 or C_4), 128.5 (C_9), 128.2 (C_3 or C_4), 128.1 (C_8), 123.6 (C_{11}). **^{19}F NMR** (376 MHz, $CDCl_3$) δ –94.67 (s, F_5). **MALDI-MS** *m/z* = 2318.092 $[M\text{-}SC_6H_4Br]^+$ (calculated for $C_{90}H_{44}F_8S_{15}Br_7^+$ = 2317.342, $S_{15}Br_7$ isotope pattern observed) for the most abundant isotope peak. See Figures S186-187 for MALDI spectrum, isotope pattern and simulated isotope pattern.

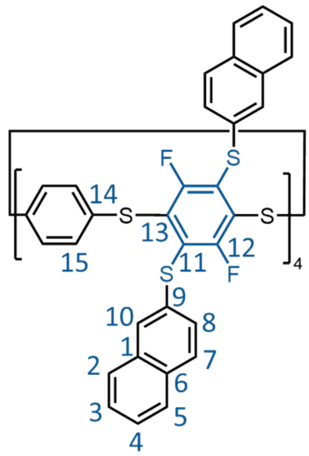

2-Thionapthlene substituted corona macrocycle (5e): To an oven-dried Schlenk flask was added corona macrocycle **1a** (10.0 mg, 8.69 µmol), 2-thionapthalene (22.5 mg, 0.14 mmol, 16 equiv) and THF (1 mL) and deoxygenated (3 × freeze-pump-thaw cycles under N_2). Next, 1,1,3,3-tetramethylguanidine (TMG) (50 µL, 0.28 mmol, 32 equiv) was added and the mixture was stirred under an N_2-atmosphere for 2 h at rt. The mixture was poured onto 0.5 M HCl (5 mL) and extracted with CH_2Cl_2 (3 × 5 mL). The combined organic phase was then washed with water (3 × 5 mL), brine (10 mL), dried ($MgSO_4$), filtered and the solvent

evaporated under reduced pressure. The resulting solid was washed with Et_2O (3 × 5 mL) and warm pentane (5 mL) to give the title compound as a colourless solid (18.7 mg, 8.23 µmol, 95%). **Mp** > 300 °C. **^{1}H NMR** (500 MHz, $CDCl_3$) δ 7.67 (d, 8H, *J* = 7.9 Hz, H_2), 7.58 (d, 8H, *J* = 8.8 Hz, H_5), 7.56 (m, 16H, H_{7+10}). 7.39 (m, 8H, H_4), 7.34 (m, 8H, H_3), 7.13 (m, 8H, H_8), 6.86 (s, 16H, H_{15}). **^{13}C NMR** (126 MHz, $CDCl_3$) δ 159.3 (C_{12}), 133.8 (C_{14}), 133.7 (C_1), 132.4 (C_6), 131.9 (C_9), 130.8 (C_{15}), 129.2 (C_{11} or C_{13}), 129.0 (C_5), 128.9 (C_{11} or C_{13}), 128.8 (C_{10}), 127.9 (C_2), 127.4 (C_7), 127.2 (C_8), 126.9 (C_4), 126.5 (C_3). **^{19}F NMR** (376 MHz, $CDCl_3$) δ –93.96 (s, F_{12}). **MALDI-MS** *m/z* = 2274.283 $[M]^+$ (calculated for $C_{128}H_{72}F_8S_{16}^+$ = 2275.113, S_{16} isotope pattern observed) for the most abundant isotope peak. See Figures S188-190 for MALDI spectrum, isotope pattern and simulated isotope pattern.

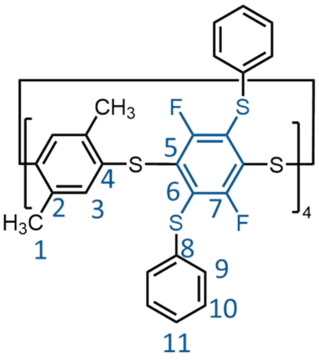

Thiophenol substituted corona macrocycle (4b): To an oven-dried Schlenk flask was added **1b** (5 mg, 3.96 µmol), tetramethylammonium fluoride tetrahydrate (1 mg, 12.1 µmol, 3 equiv.) and pyridine (1 mL) and deoxygenated (3 × freeze-pump-thaw cycles under N_2). Thiophenol (5.9 µL, 63.4 µmol, 16 equiv) was added and the mixture was stirred at rt under an N_2-atmosphere for 2 h. The mixture was poured onto 0.5 M HCl (5 mL) and extracted with CH_2Cl_2 (3 × 5 mL). The combined organic phase was then washed with water (3 × 5 mL), brine (5 mL), dried ($MgSO_4$), filtered and the solvent removed under reduced pressure. The resulting solid was washed with Et_2O (3 × 5 mL) and warm pentane (5 mL) to give the title compound as a colourless solid (7.4 mg, 3.72 µmol, 94%). **Mp** >300 °C. **^{1}H NMR** (500 MHz, $CDCl_3$) δ 7.14 (m, 40H, $H_{9+10+11}$), 6.80 (s, 8H, H_3), 2.10 (s, 24H, H_1). **^{13}C NMR** (126 MHz, $CDCl_3$) δ 159.1 (C_7), 136.7 (C_8), 134.7, 133.0, 132.7, 129.5 ($C_{9 \text{ or } 10 \text{ or } 11}$), 129.4 ($C_{9 \text{ or } 10 \text{ or } 11}$), 127.3 ($C_3$), 115.6 ($C_{5+6}$). **^{19}F NMR** (376 MHz, $CDCl_3$, δ –92.9 (s, F_7). **MALDI-MS** *m/z* = 1986.441 $[M]^+$ (calculated for $C_{104}H_{72}F_8S_{16}^+$ = 1987.112, S_{16} isotope pattern observed) for the most abundant isotope peak. See Figures S171-173 for MALDI spectrum, isotope pattern and simulated isotope pattern.

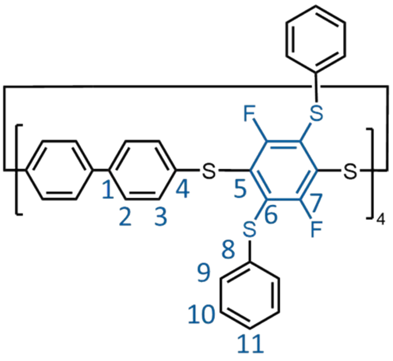

Thiophenol substituted corona macrocycle (4d): To an oven-dried Schlenk flask was added corona macrocycle **1d** (6.3 mg, 4.3 µmol), tetramethylammonium fluoride tetrahydrate (1 mg, 6.50 µmol) and pyridine (1 mL) and deoxygenated (3 × freeze-pump-thaw cycles under N_2). Thiophenol (7 µL, 70 µmol, 16 equiv) was added and the mixture was stirred under an N_2-atmosphere for 2 h at rt. The mixture was poured onto 0.5 M

HCl (5 mL) and extracted with CH_2Cl_2 (3 × 10 mL). The combined organic phase was then washed with water (3 × 5 mL), brine (10 mL), dried over anhydrous $MgSO_4$, filtered and the solvent was removed under reduced pressure. The resulting solid was washed with Et_2O (3 × 5 mL) and warm pentane (5 mL) to give the title compound as a yellow solid (10.0 mg, 4.24 µmol, 99%). **Mp** >300 °C. **^{1}H NMR** (500 MHz, $CDCl_3$) δ 7.31 (d, *J* = 8.5 Hz, 16H, H_2), 7.21 (d, *J* = 8.5 Hz, 16H, H_3), 7.15 (m, 40H, $H_{9,10,11}$). **^{13}C NMR** (126 MHz, $CDCl_3$) δ 139.3 (C_1), 134.8 (C_8), 134.2 (C_4), 130.4 (C_3), 129.5 (C_9 or C_{10} or C_{11}), 129.3 (C_9 or C_{10} or C_{11}), 127.7 (C_2), 127.2 (C_9 or C_{10} or C_{11}). **^{19}F NMR** (376 MHz, $CDCl_3$, C_6F_6 referenced) δ –93.94 (s, F_7). **MALDI-MS** *m/z* = 2177.607 $[M]^+$ (calculated for $C_{120}H_{72}F_8S_{16}$ = 2178.114, S_{16} isotope pattern observed) for the most abundant isotope peak. See Figures S174-176 for MALDI spectrum, isotope pattern and simulated isotope pattern.

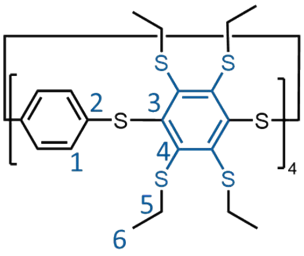

Ethanethiol substituted corona macrocycle (6a): To an oven-dried Schlenk flask was added corona macrocycle **1a** (5.0 mg, 4.3 µmol), and anhydrous DMF (1 mL) and deoxygenated (3 × freeze-pump-thaw cycles under N_2). The mixture was cooled to 0 °C in an ice bath before solid sodium thiophenolate (9.2 mg, 70 µmol, 16.1 equiv) was added in one portion and the mixture was stirred under an N_2 atmosphere for 1 h at rt. The mixture was poured onto 0.5 M HCl (2 mL) and bubbled through vigorously (5 min) with N_2 to displace unreacted ethanethiol (**CARE!** Stench). The mixture was extracted with CH_2Cl_2 (3 × 5 mL). The combined organic phase was then washed with water (3 × 5 mL), brine (5 mL), dried over anhydrous $MgSO_4$, filtered and the solvent was removed under reduced pressure. The resulting crude solid was purified by column chromatography (SiO_2, hexane-Et_2O) to give the title compound as yellow film (4.5 mg, 2.54 µmol, 59%). **Mp** >300 °C. **^{1}H NMR** (500 MHz, $CDCl_3$) δ 7.04-6.82 (m, 16H, H_1), 2.93 (m, 32H, H_5), 1.13 (m, 48H, H_6). **MALDI-MS** *m/z* = 1796.943 $[M\text{-}Et]^+$ (calculated for $C_{78}H_{91}S_{24}^+$ = 1798.048, S_{24} isotope pattern observed) for the most abundant isotope peak. See Figures 191-192 for MALDI spectrum, isotope pattern and simulated isotope pattern.

Quenching and waste management safety advice: Quenching of *i*-PrSNa and EtSNa produces highly malodorous *i*-PrSH or EtSH, that at trace concentrations smell like the odorants put in commercial gas (*t*-BuSH). Reactions venting them, at scale, were purged through 'suck back proof' bleach bubblers. Although all other sulphur-containing species used in our chemistries have only very mild odors; we have quenched all residues on commercial bleach (NaOCl/NaOH), leaving the

glassware overnight. Subsequent aqueous disposal caused no issues in our hands. Issues associated with adventurous HF generation were negated by basic residue quenches.

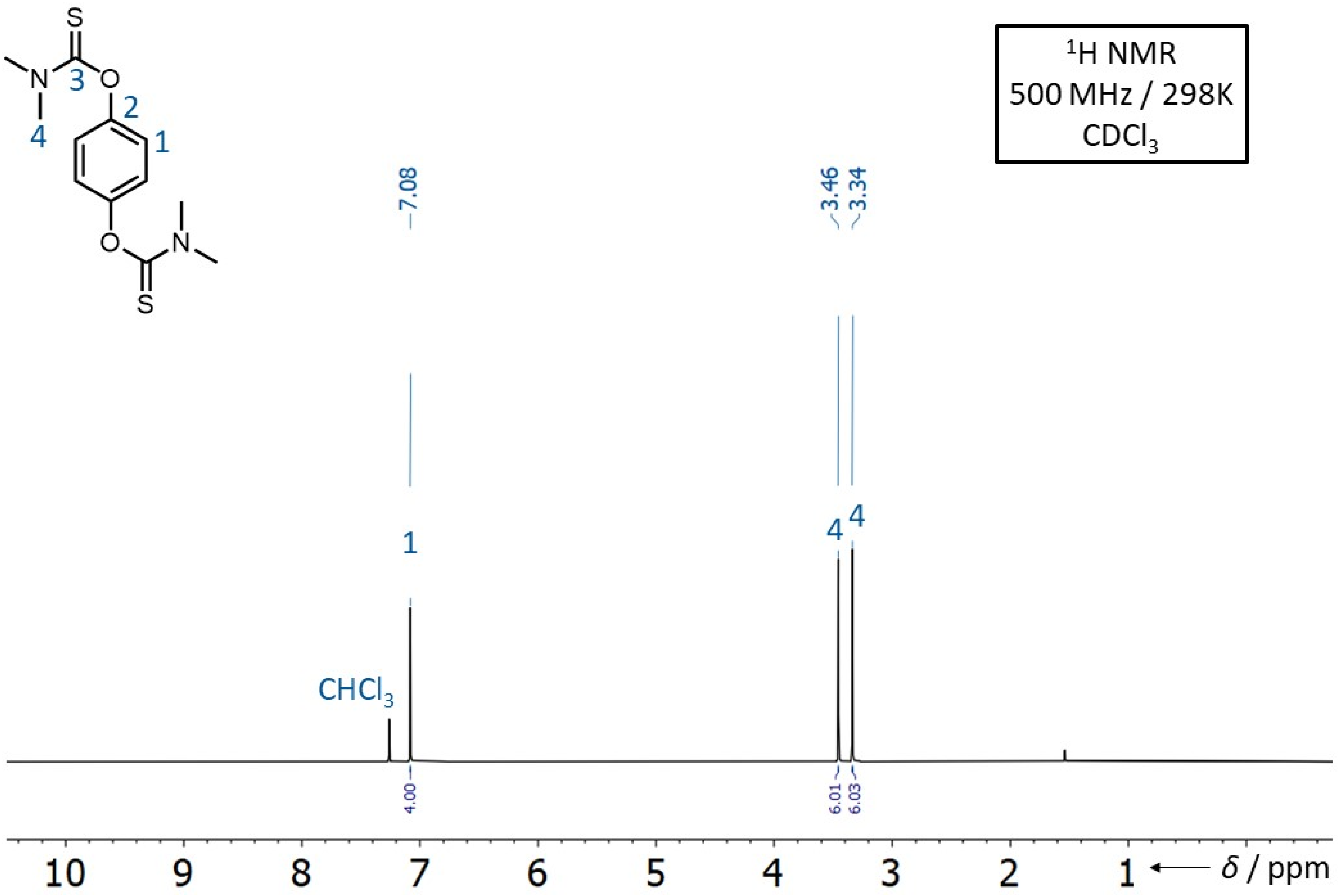

3. ^{1}H, ^{13}C and ^{19}F NMR Spectroscopic Characterization of Synthesised Compounds

Figure S3. ^{1}H NMR Spectrum of ***O,O'*-(1,4-phenylene)*bis*(dimethylcarbamothioate)**.

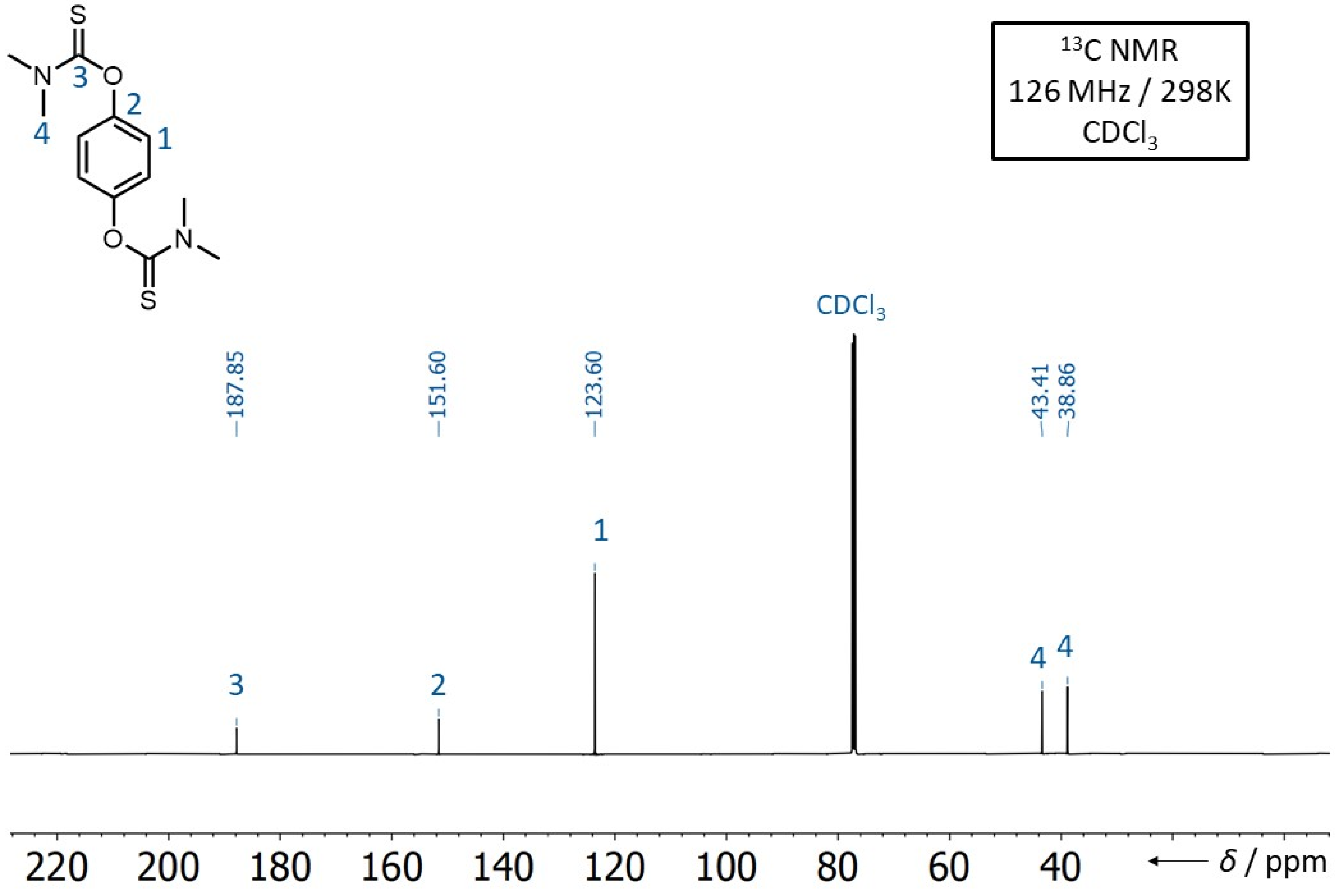

Figure S4. ^{13}C NMR Spectrum of ***O,O*'-(1,4-phenylene)*bis*(dimethylcarbamothioate)**.

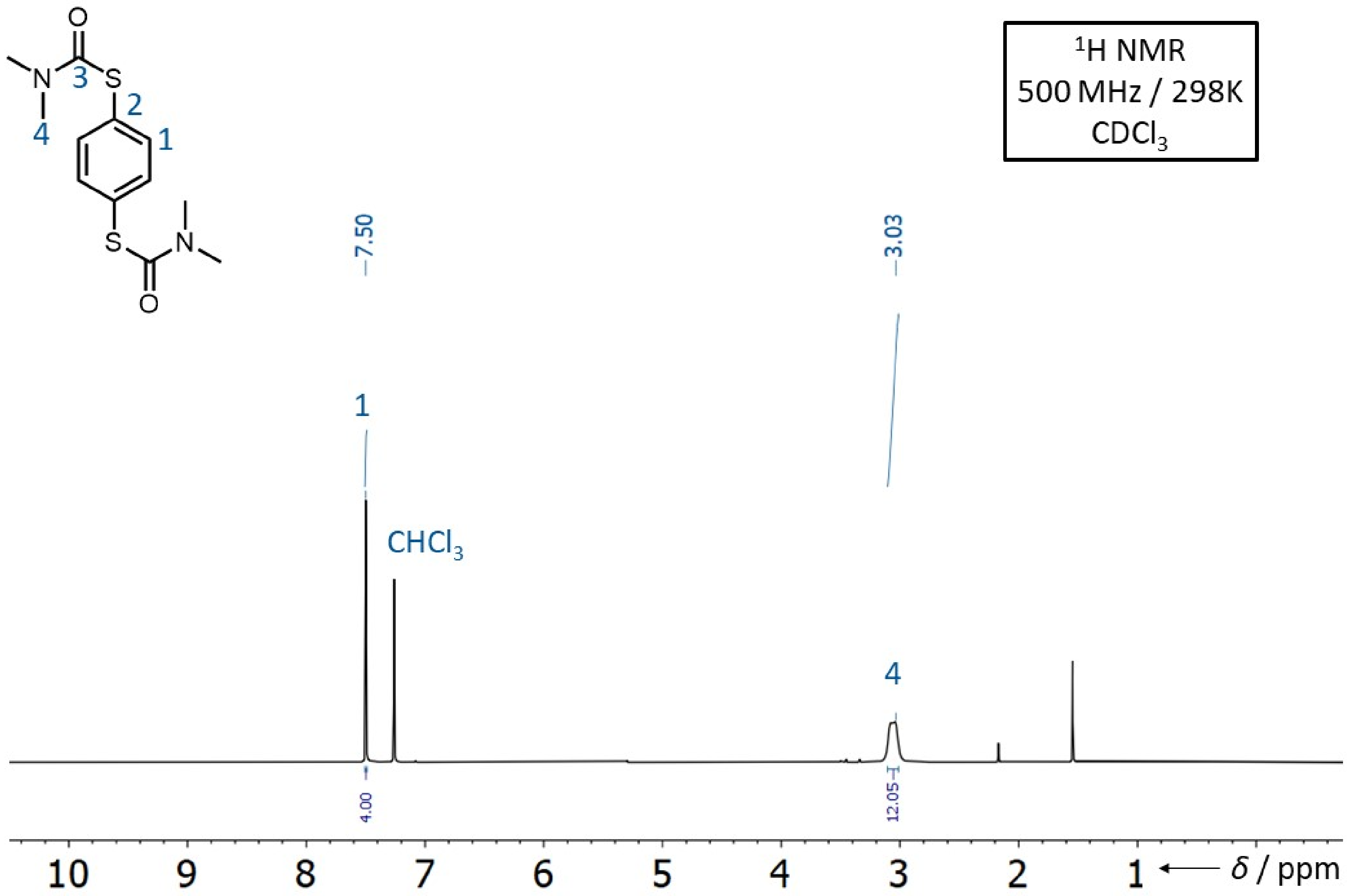

Figure S5. ^{1}H NMR Spectrum of ***S,S*'-(1,4-phenylene)*bis*(dimethylcarbamothioate)**.

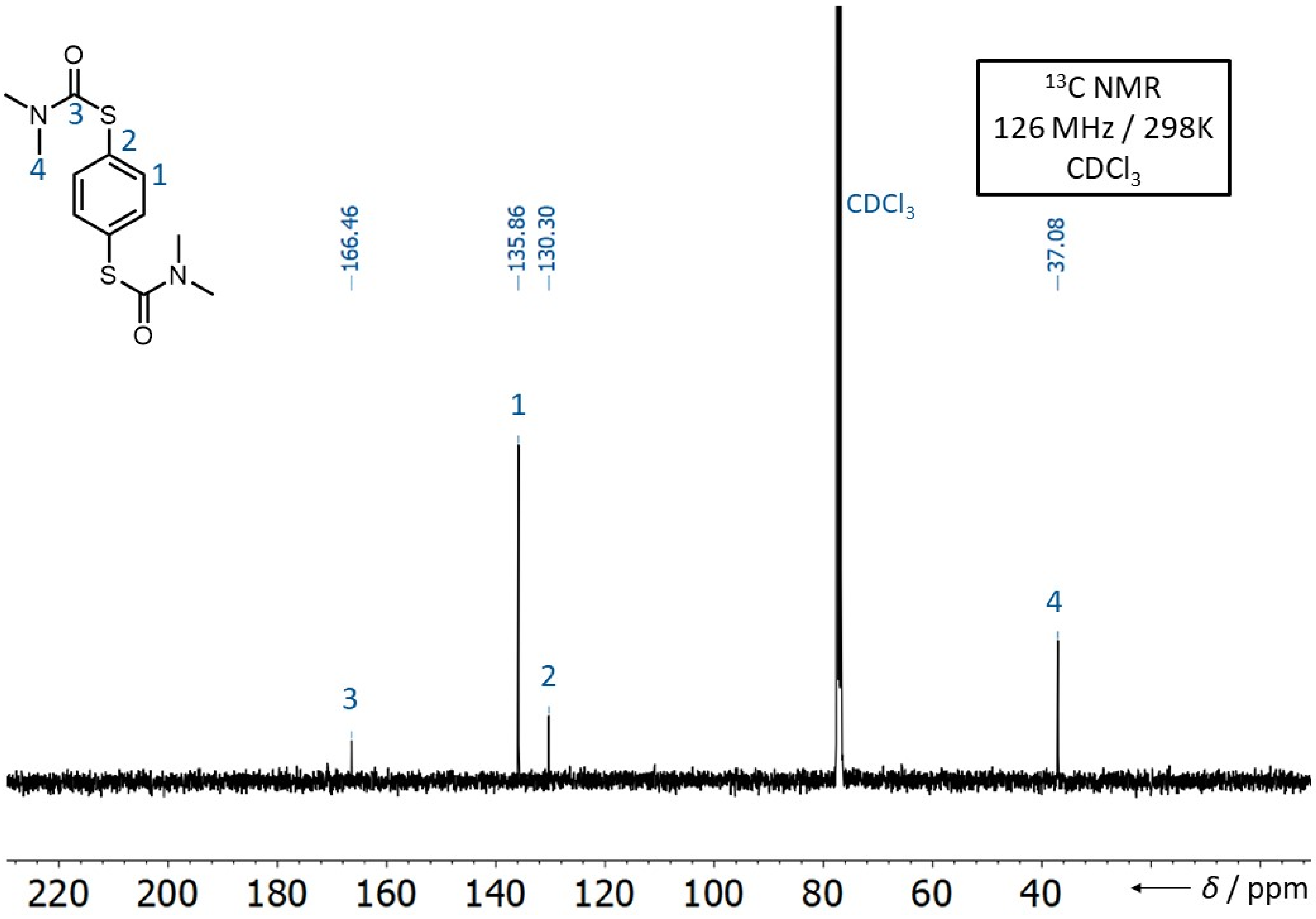

Figure S6. ^{13}C NMR Spectrum of ***S,S*'-(1,4-phenylene)*bis*(dimethylcarbamothioate)**.

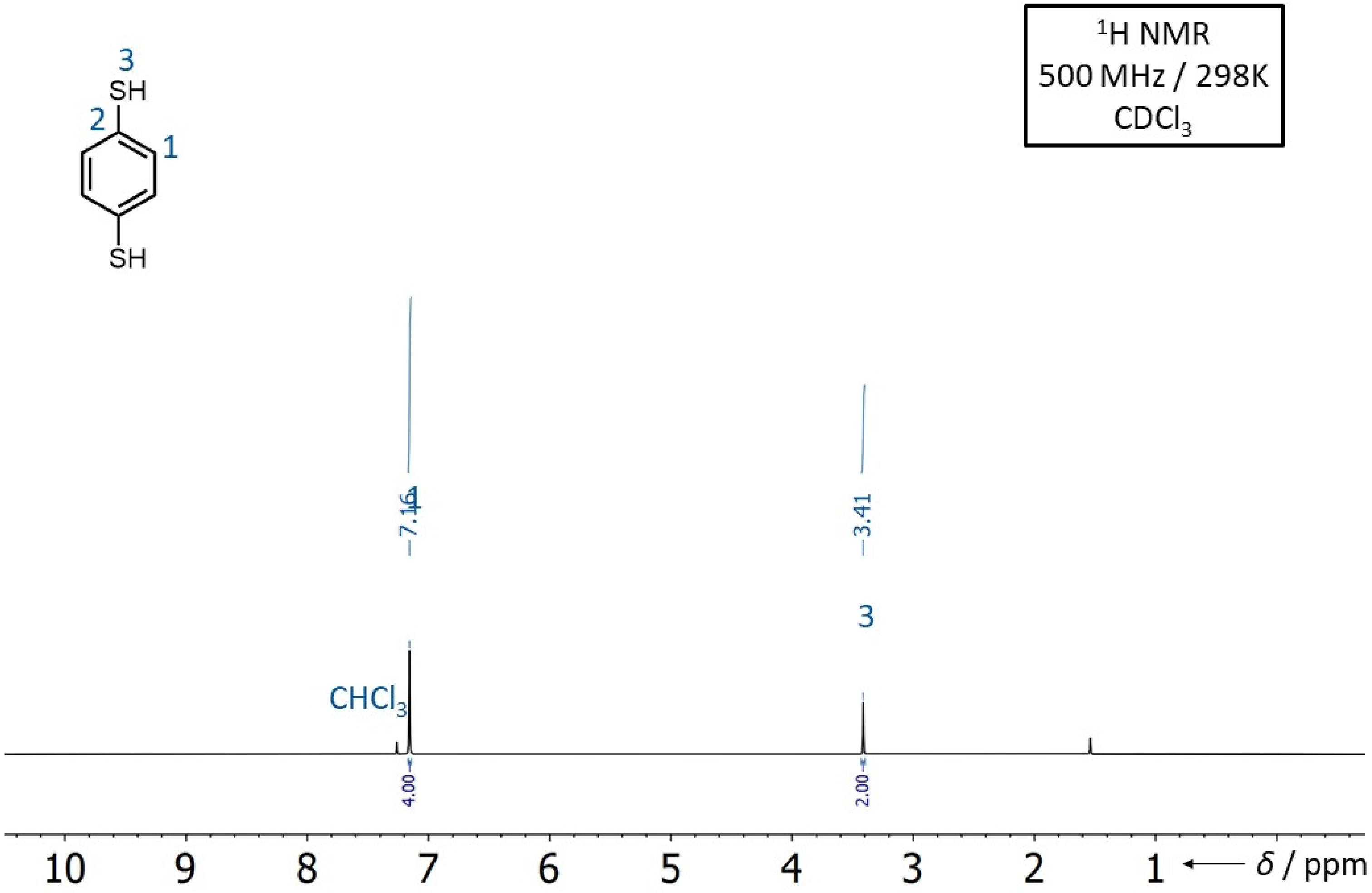

Figure S7. ^{1}H NMR Spectrum of **benzene-1,4-dithiol** (**2a**).

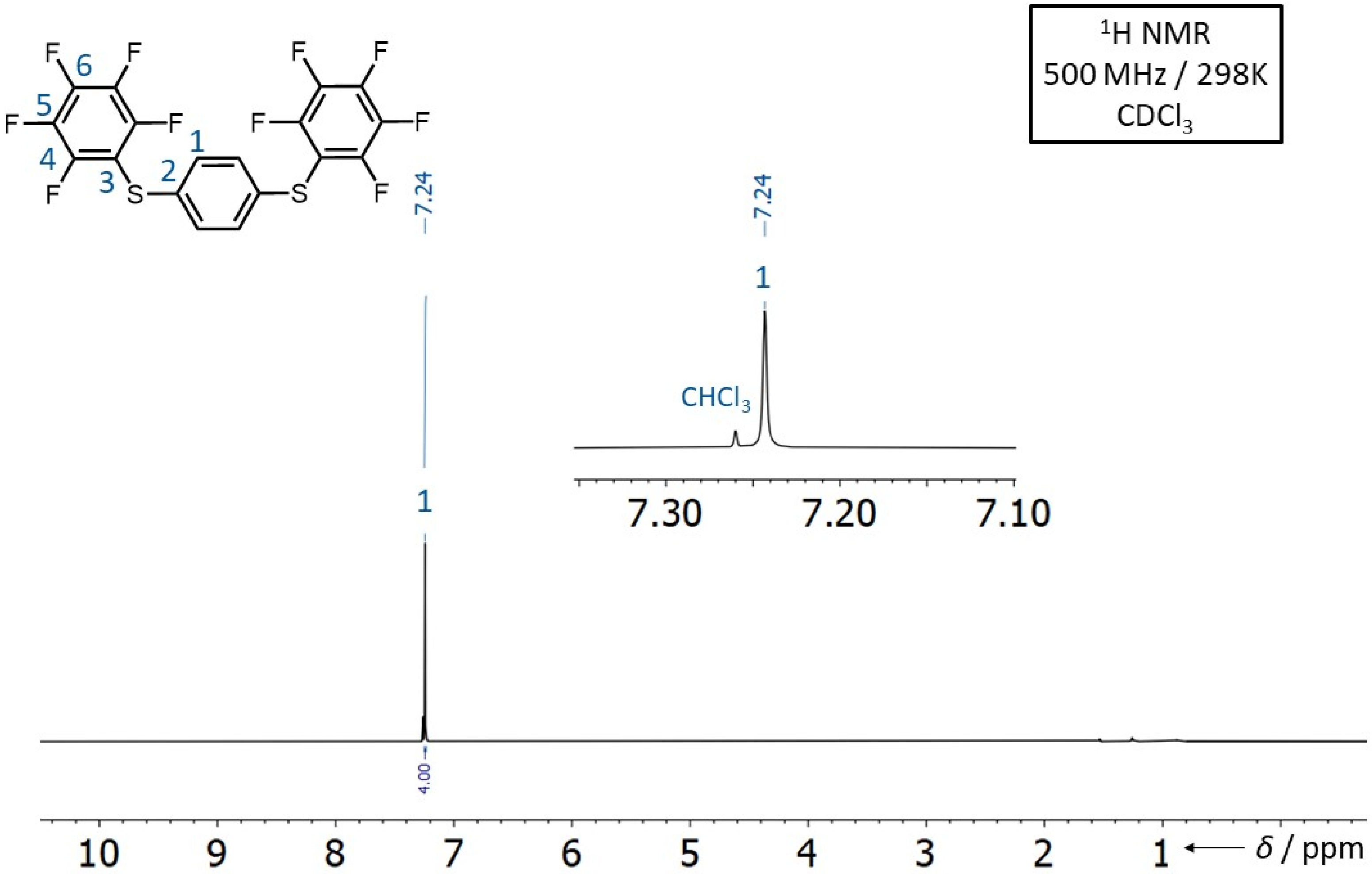

Figure S8. ^{1}H NMR Spectrum of **3a**.

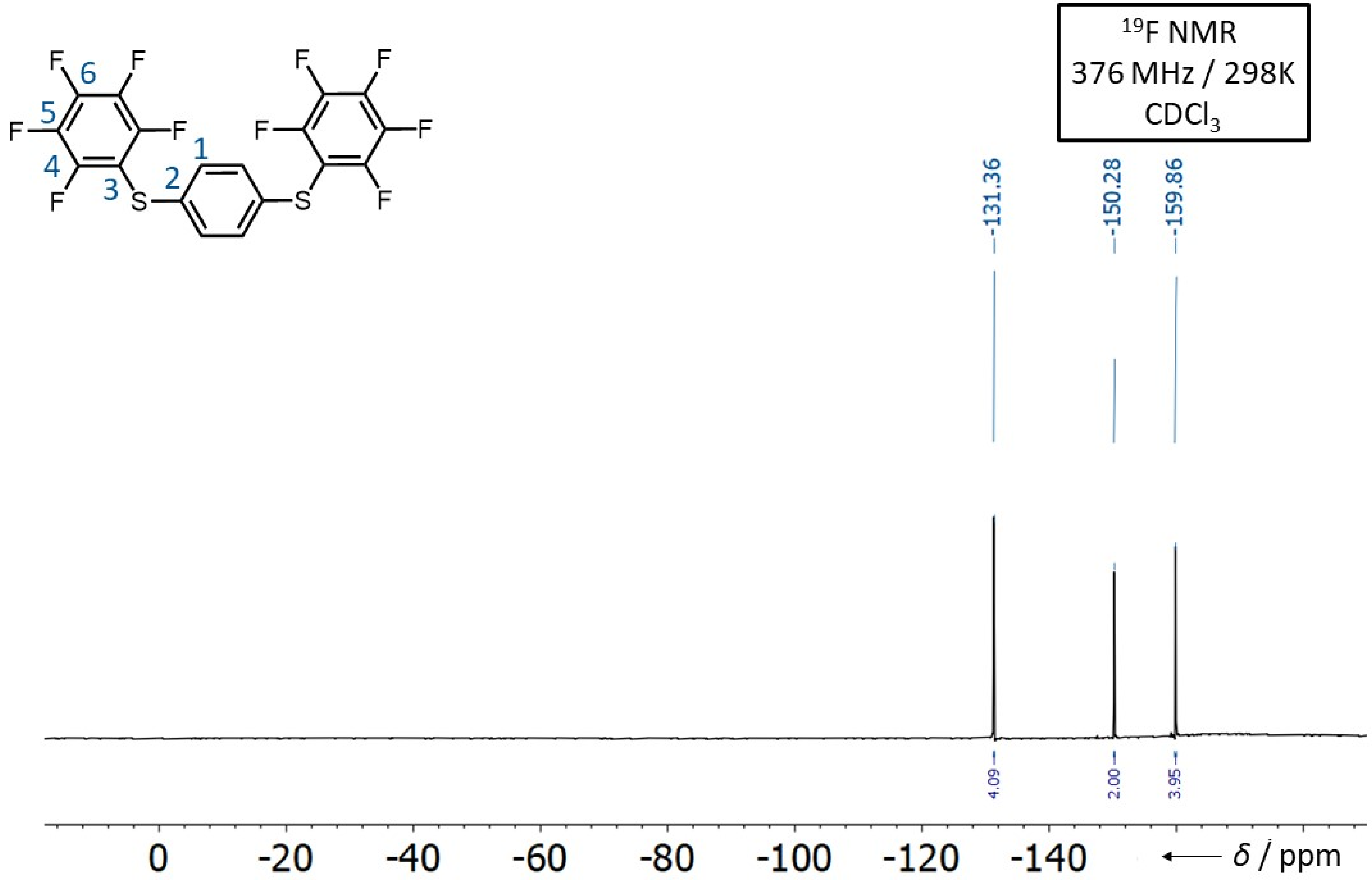

Figure S9. ^{19}F NMR Spectrum of **3a**.

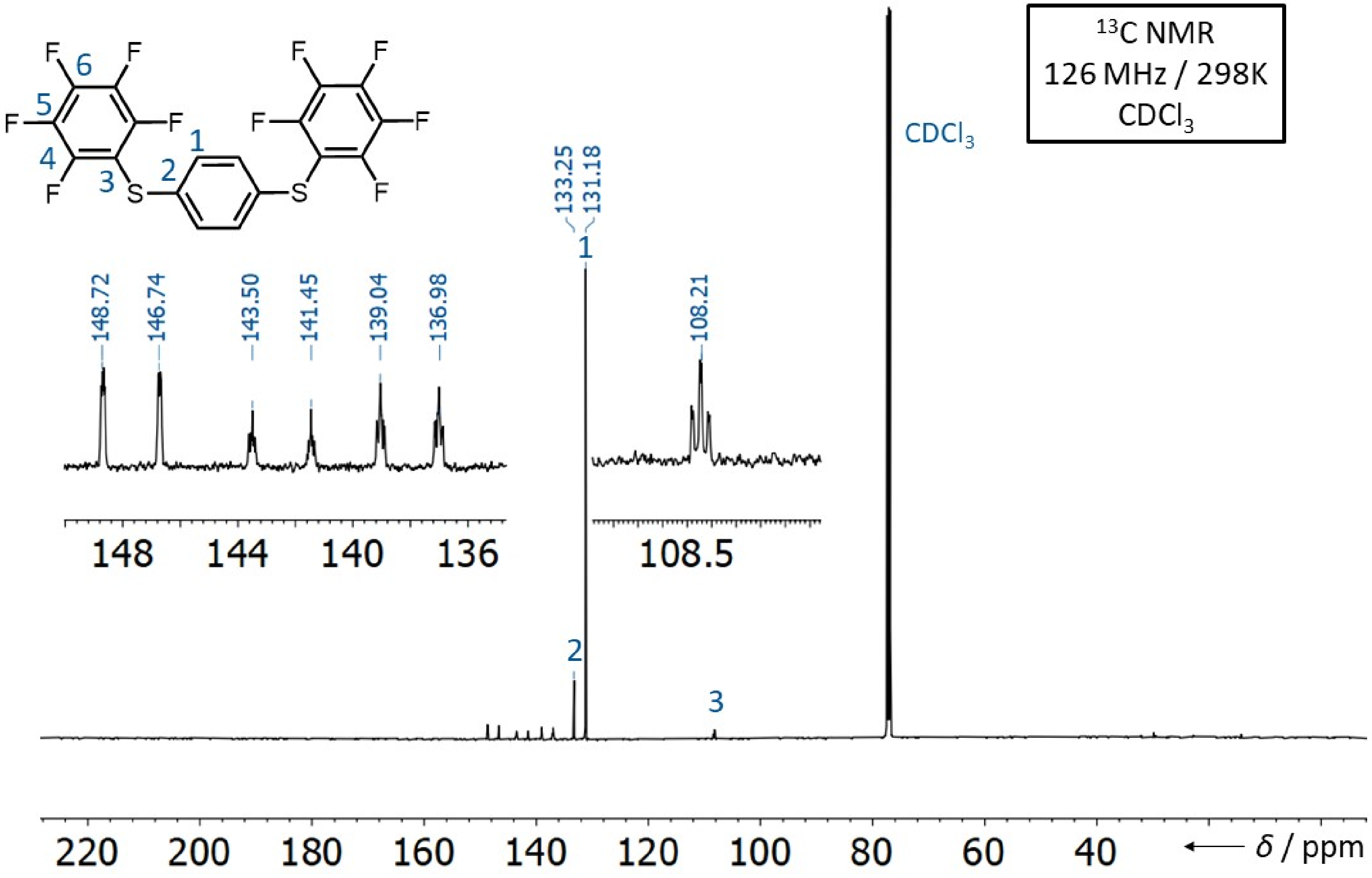

Figure S10. ^{13}C NMR Spectrum of **3a**.

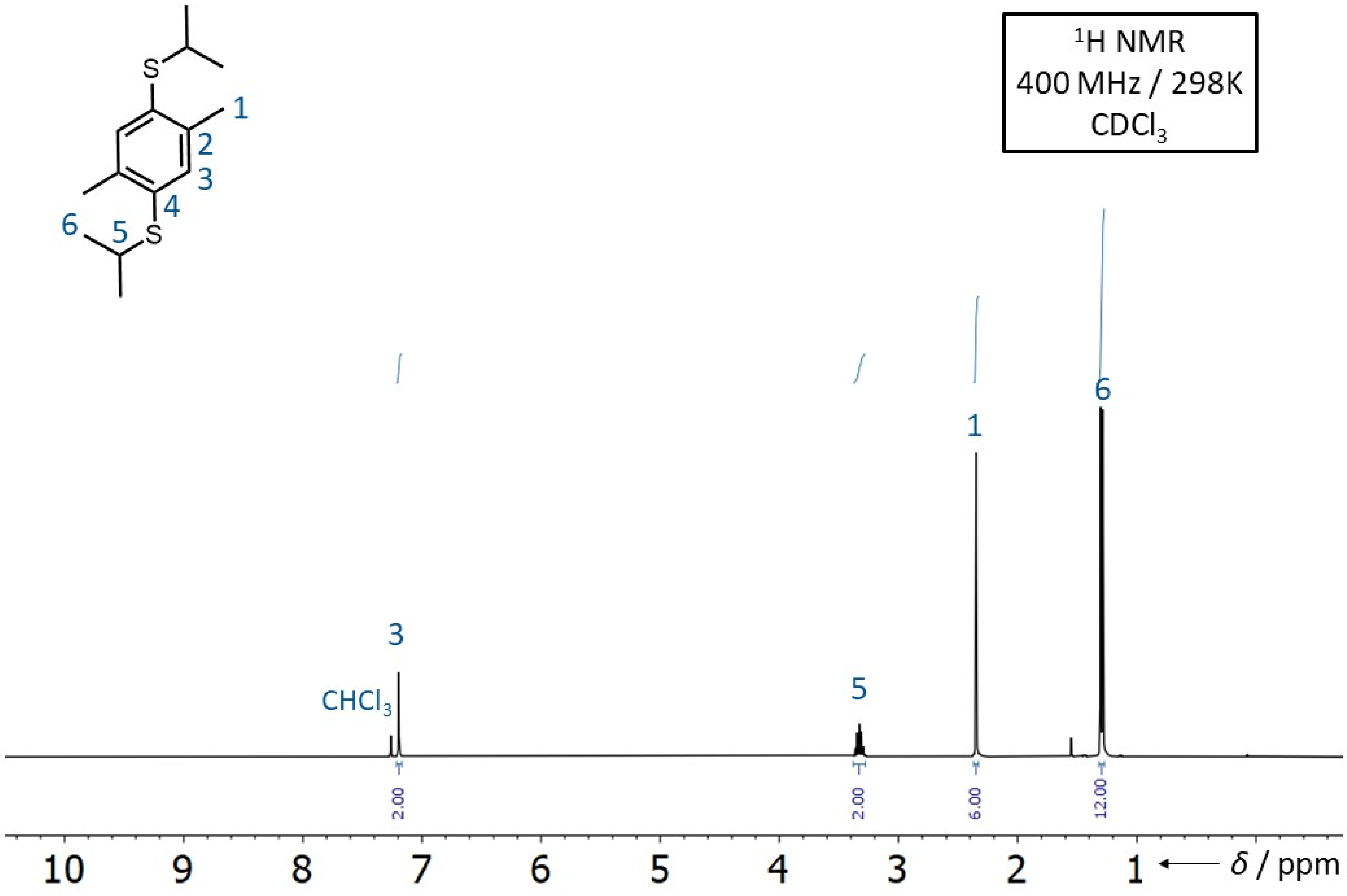

Figure S11. ^{1}H NMR Spectrum of **(2,5-dimethyl-1,4-phenylene)*bis*(isopropylsulfane)**.

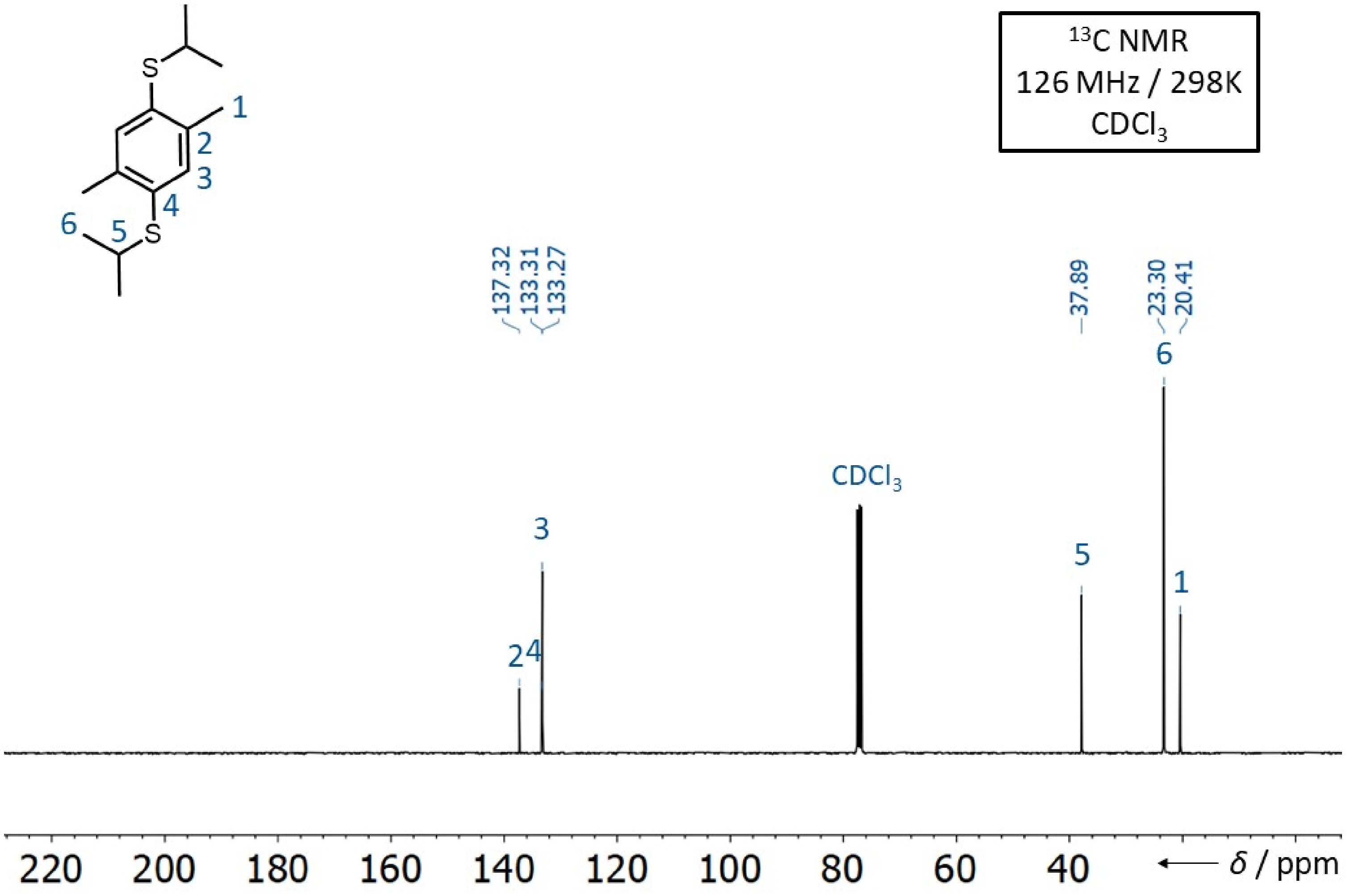

Figure S12. ^{13}C NMR Spectrum of **(2,5-dimethyl-1,4-phenylene)*bis*(isopropylsulfane)**.

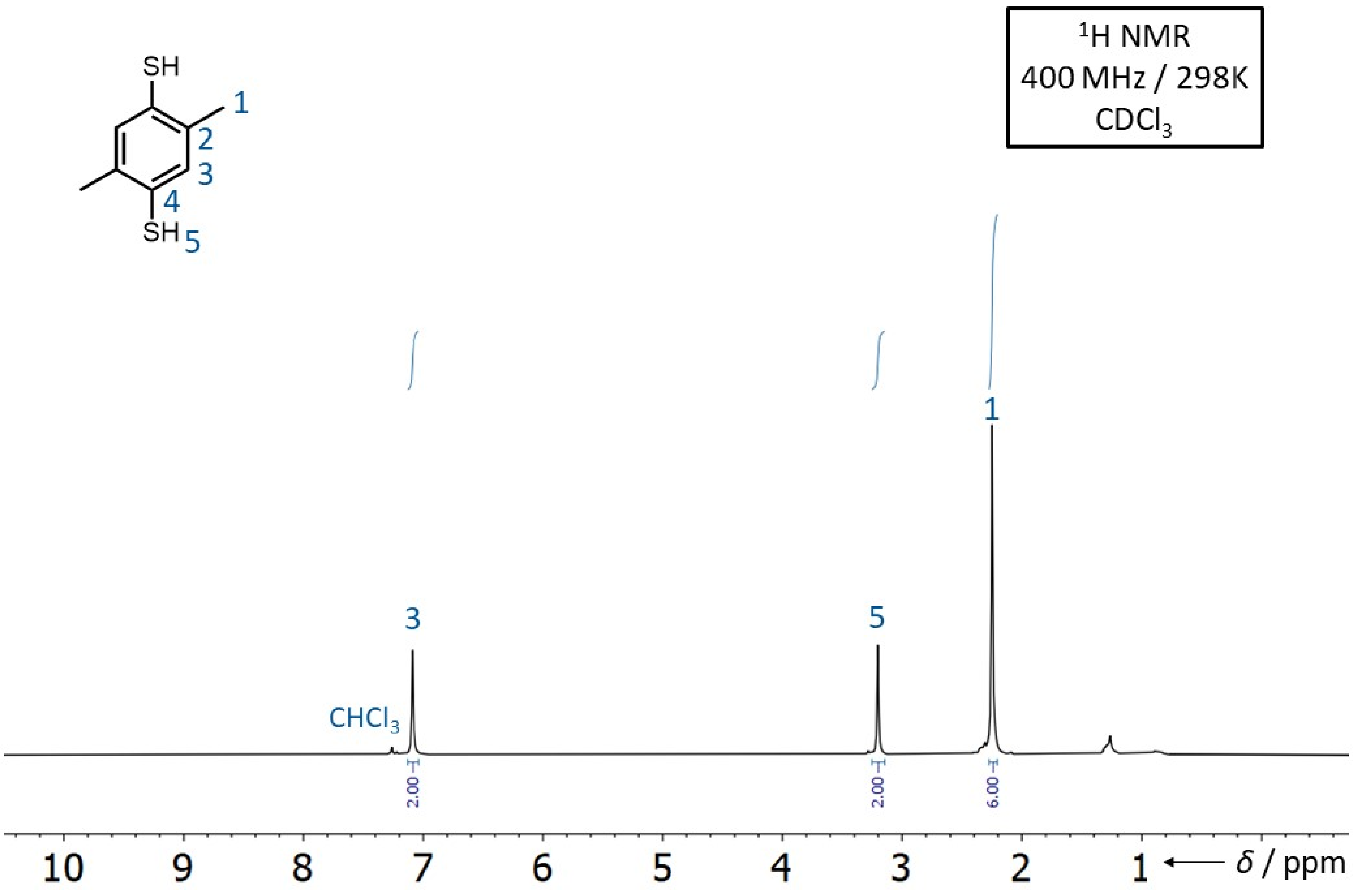

Figure S13. ^{1}H NMR Spectrum of **2,5-dimethylbenzene-1,4-dithiol**.

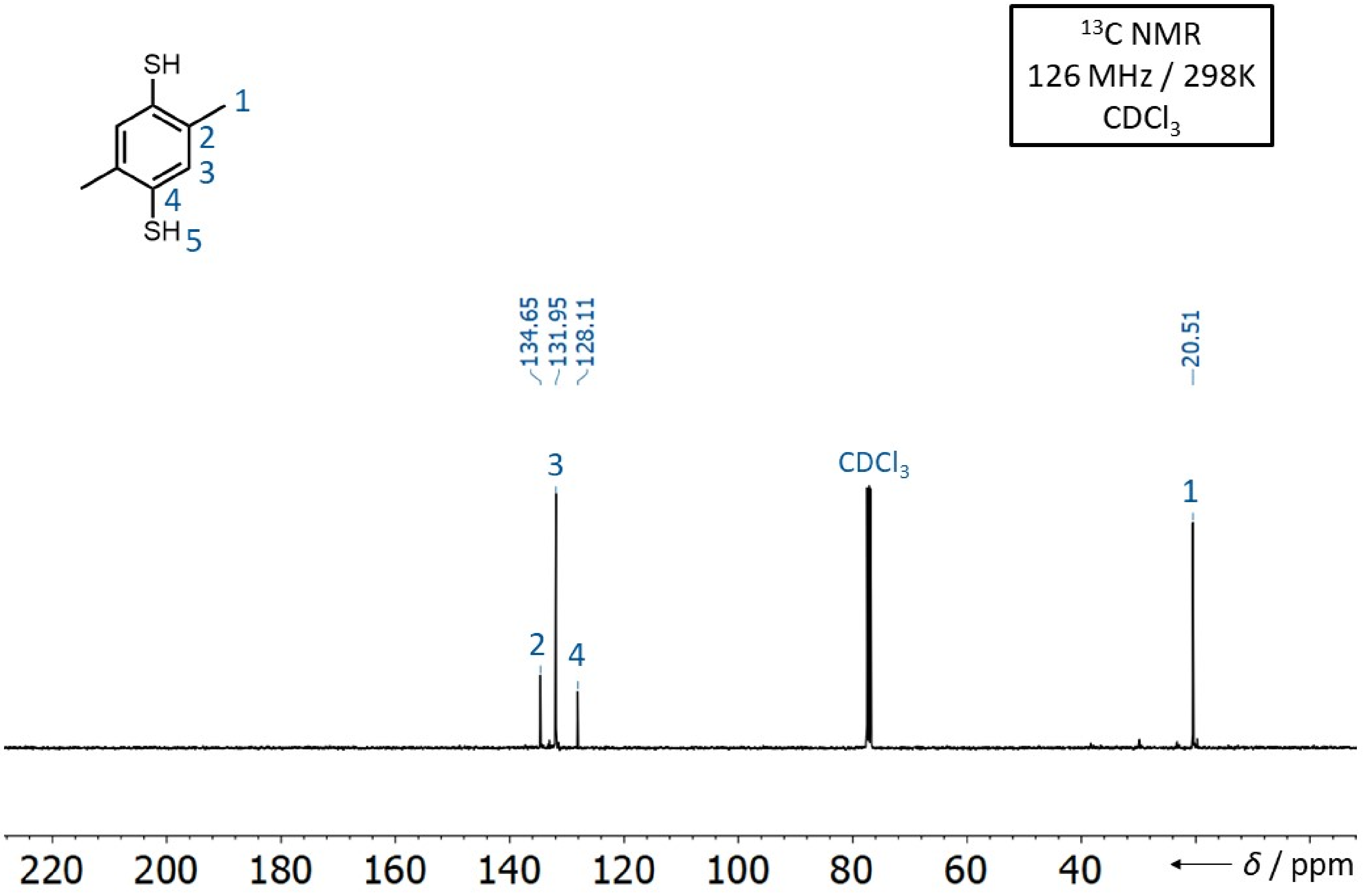

Figure S14. ^{13}C NMR Spectrum of **2,5-dimethylbenzene-1,4-dithiol**.

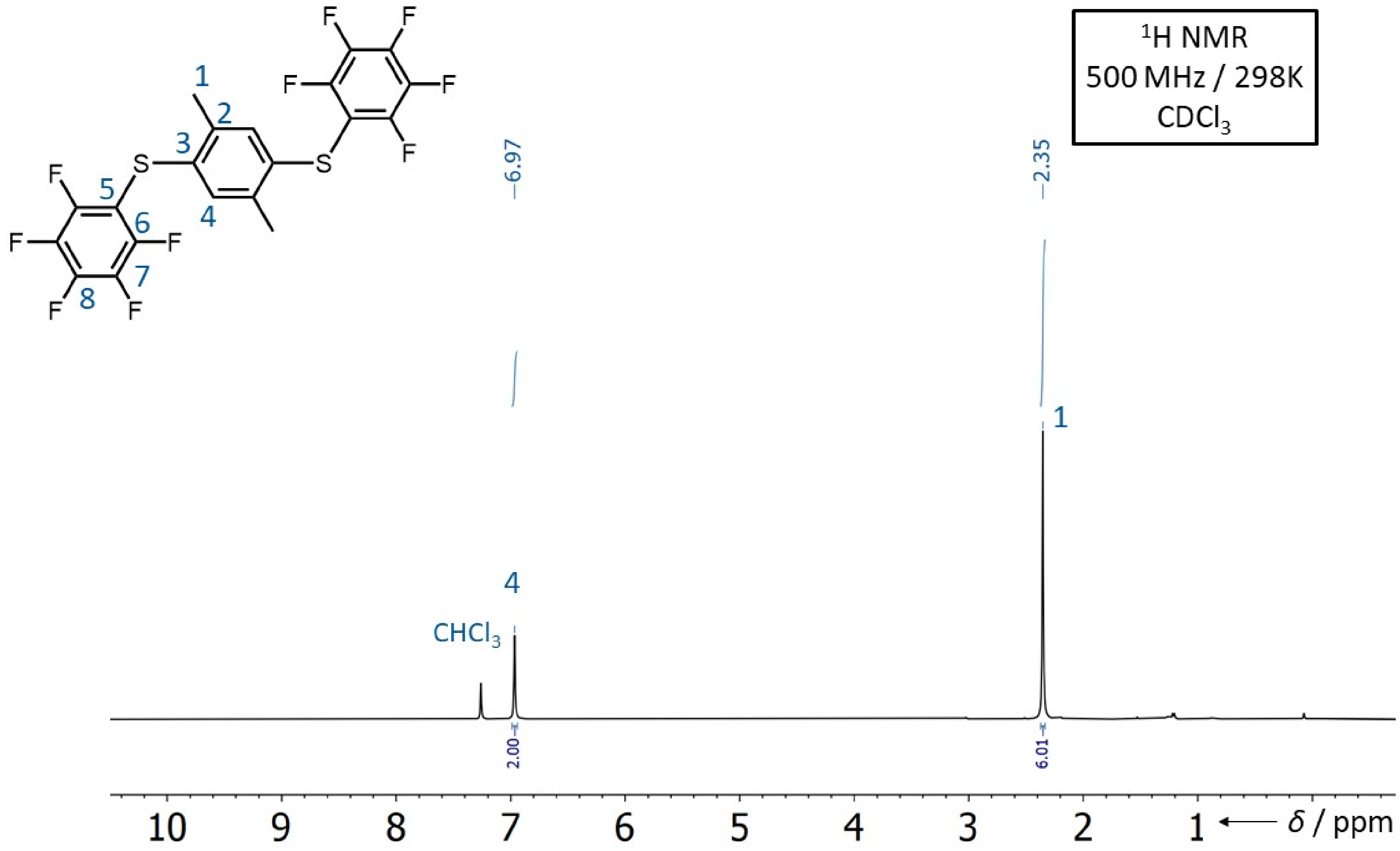

Figure S15. ^{1}H NMR Spectrum of **3b**.

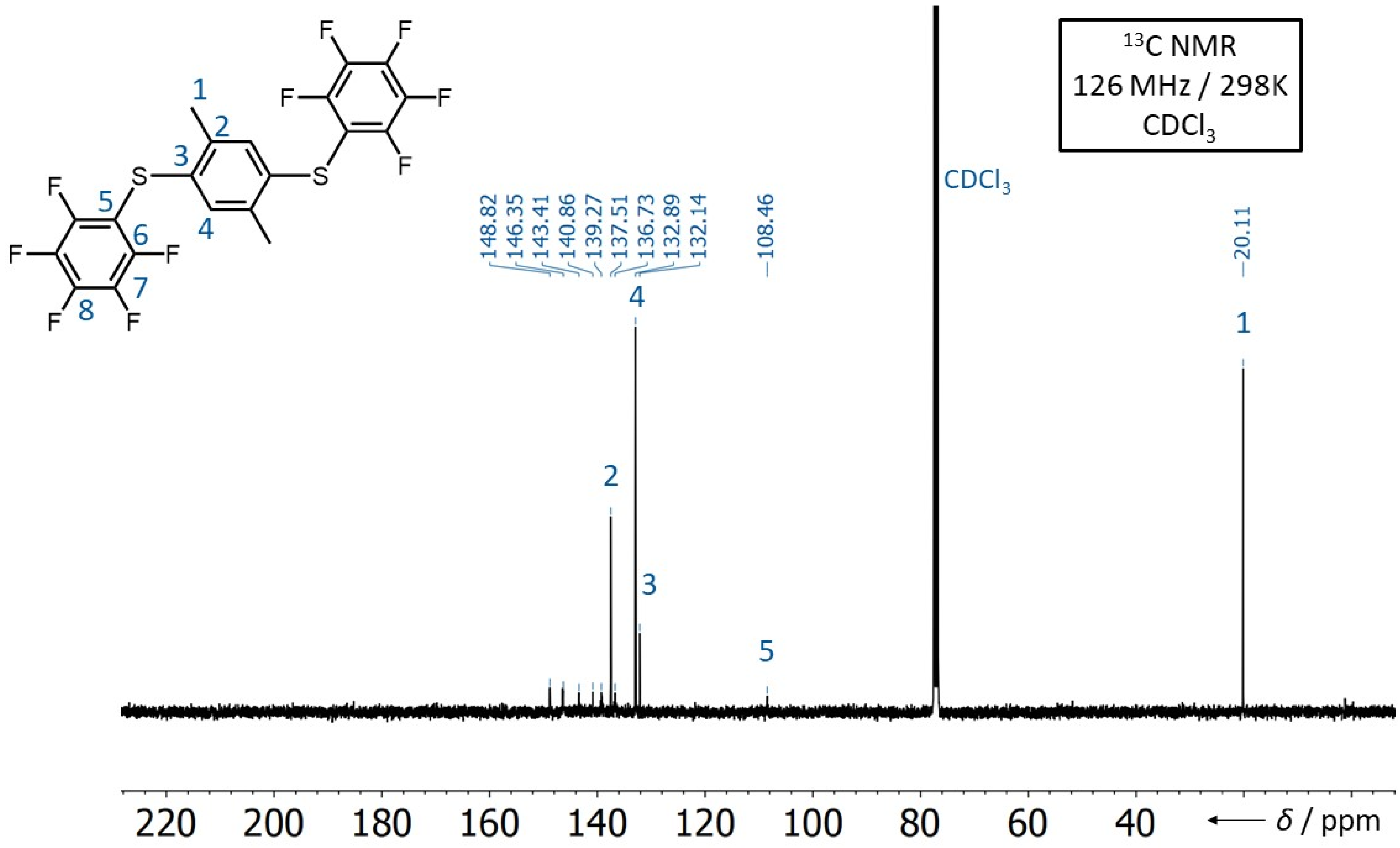

Figure S16. ^{13}C NMR Spectrum of **3b**.

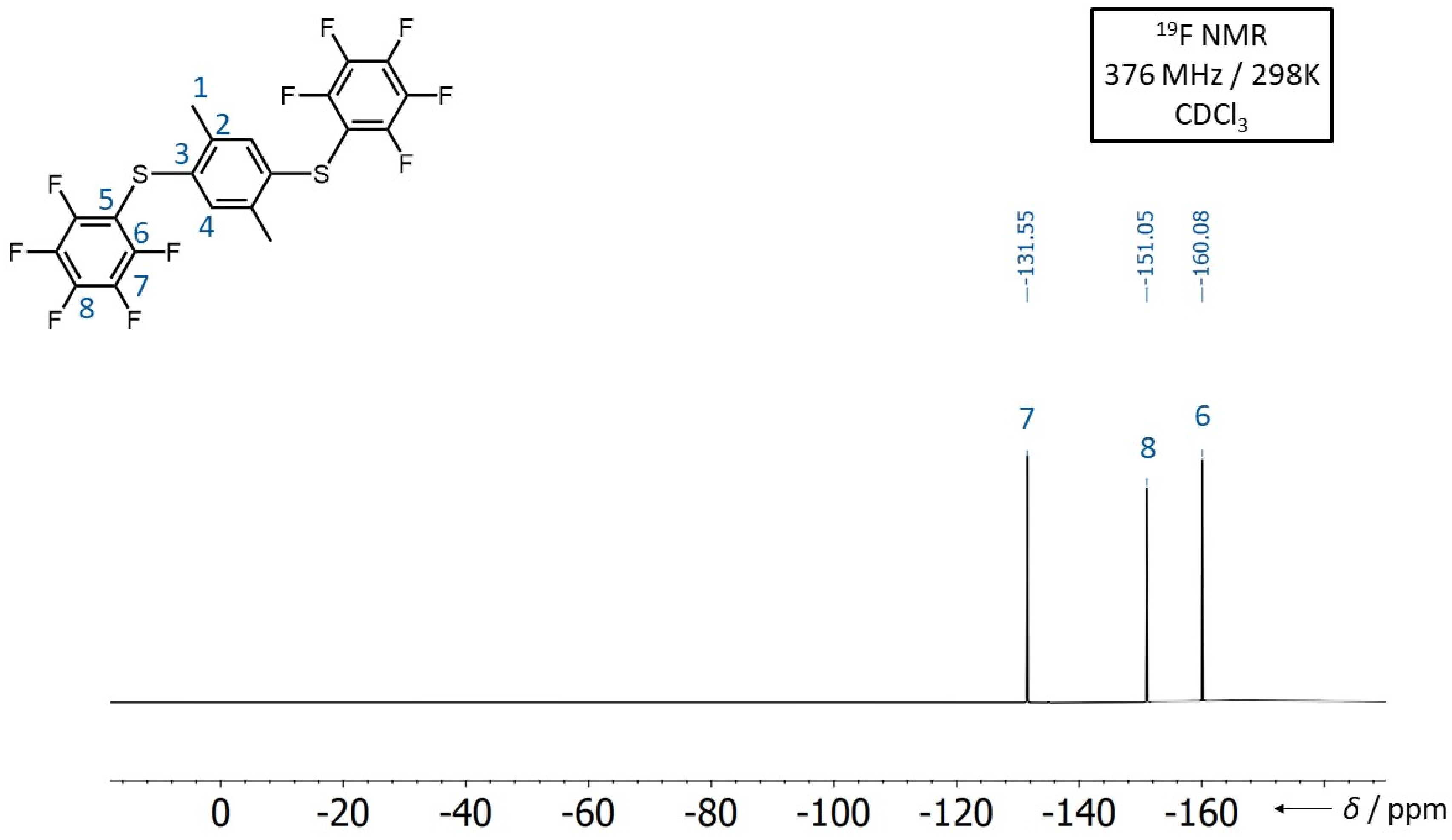

Figure S17. ^{19}F NMR Spectrum of **3b**.

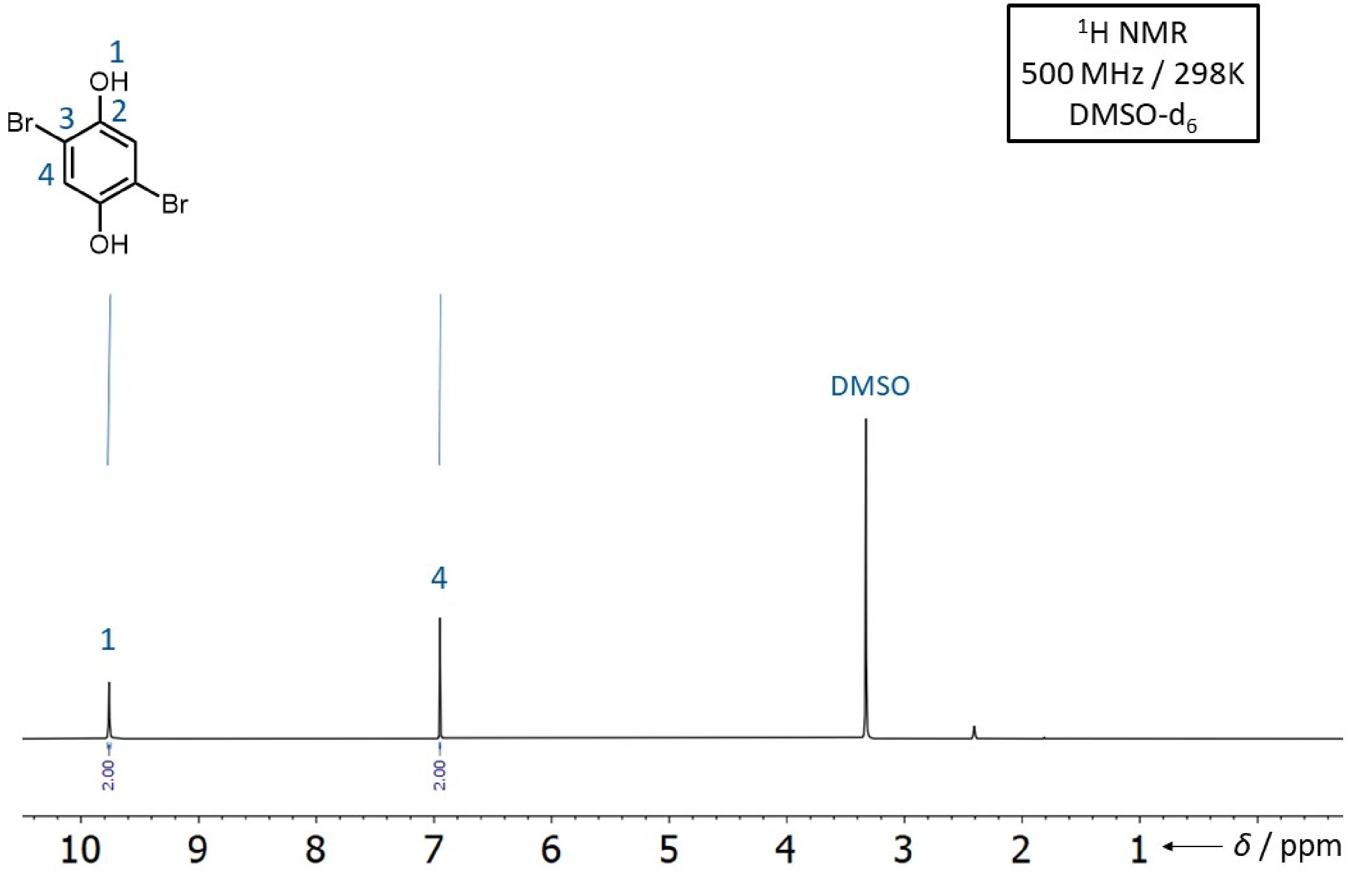

Figure S18. ^{1}H NMR Spectrum of **2,5-dibromohydroquinone**.

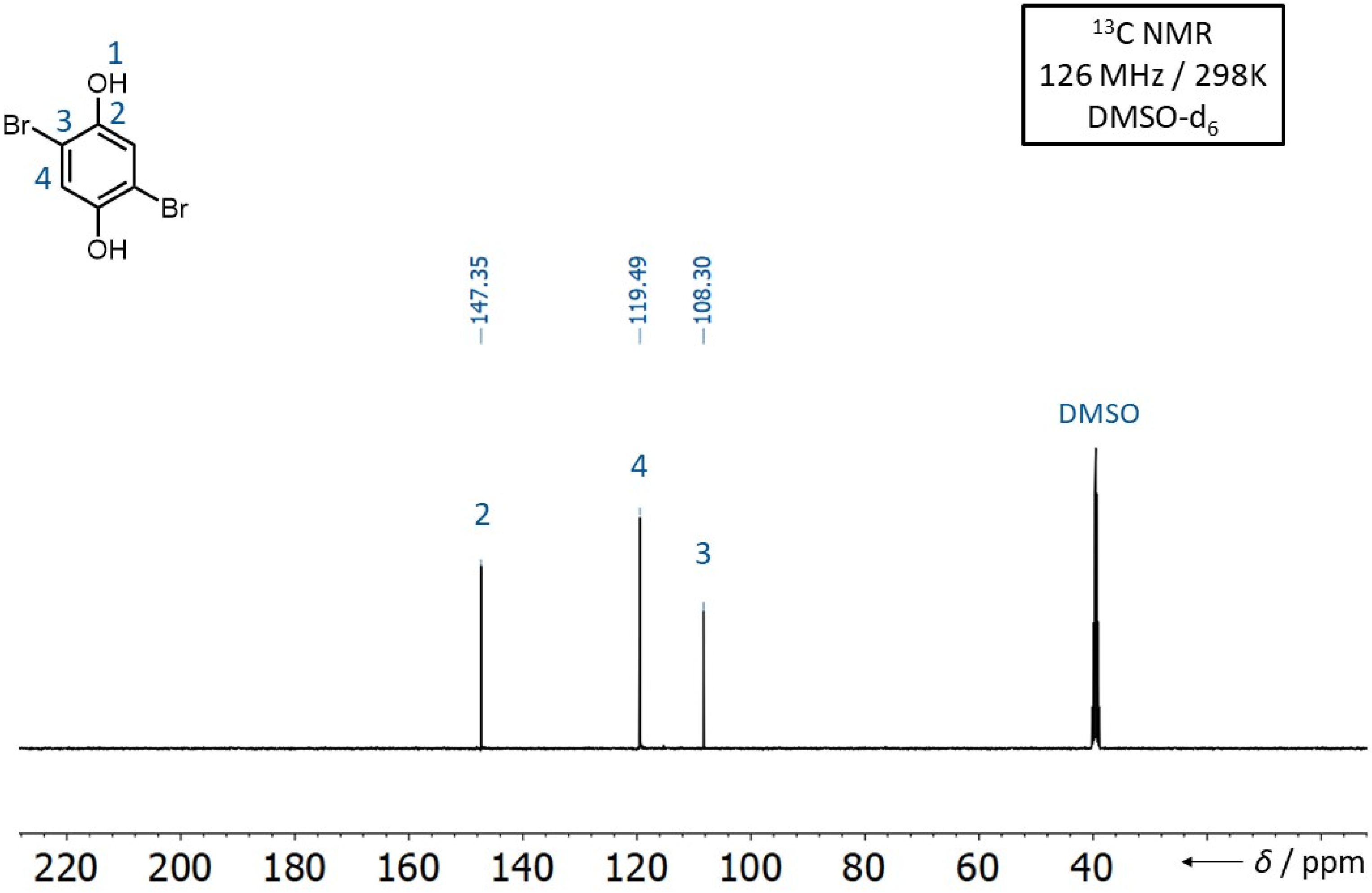

Figure S19. ^{13}C NMR Spectrum of **2,5-dibromohydroquinone**.

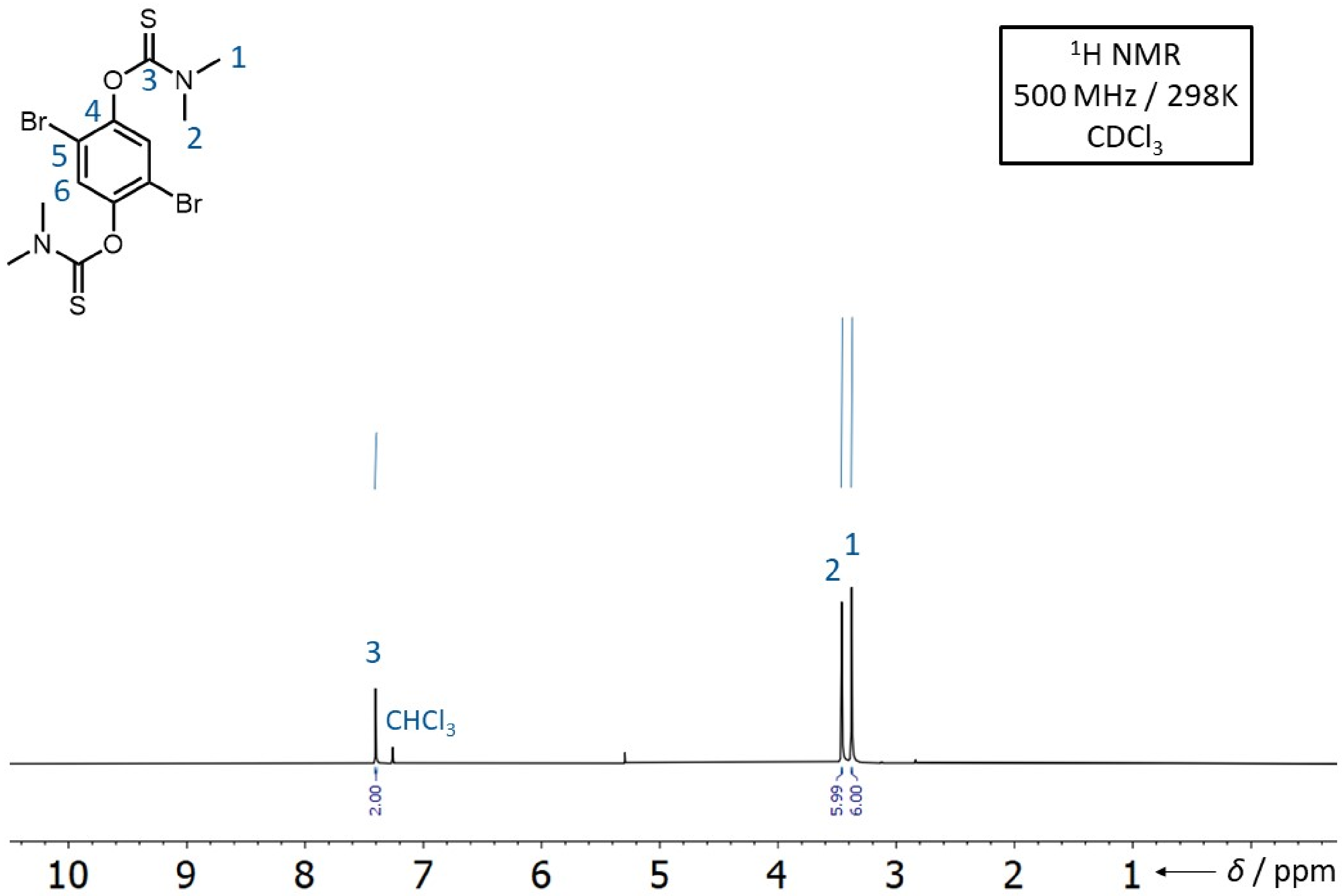

Figure S20. ^{1}H NMR Spectrum of ***O,O'*-(2,5-dibromo-1,4-phenlene)*bis*(dimethylcarbamothioate)**.

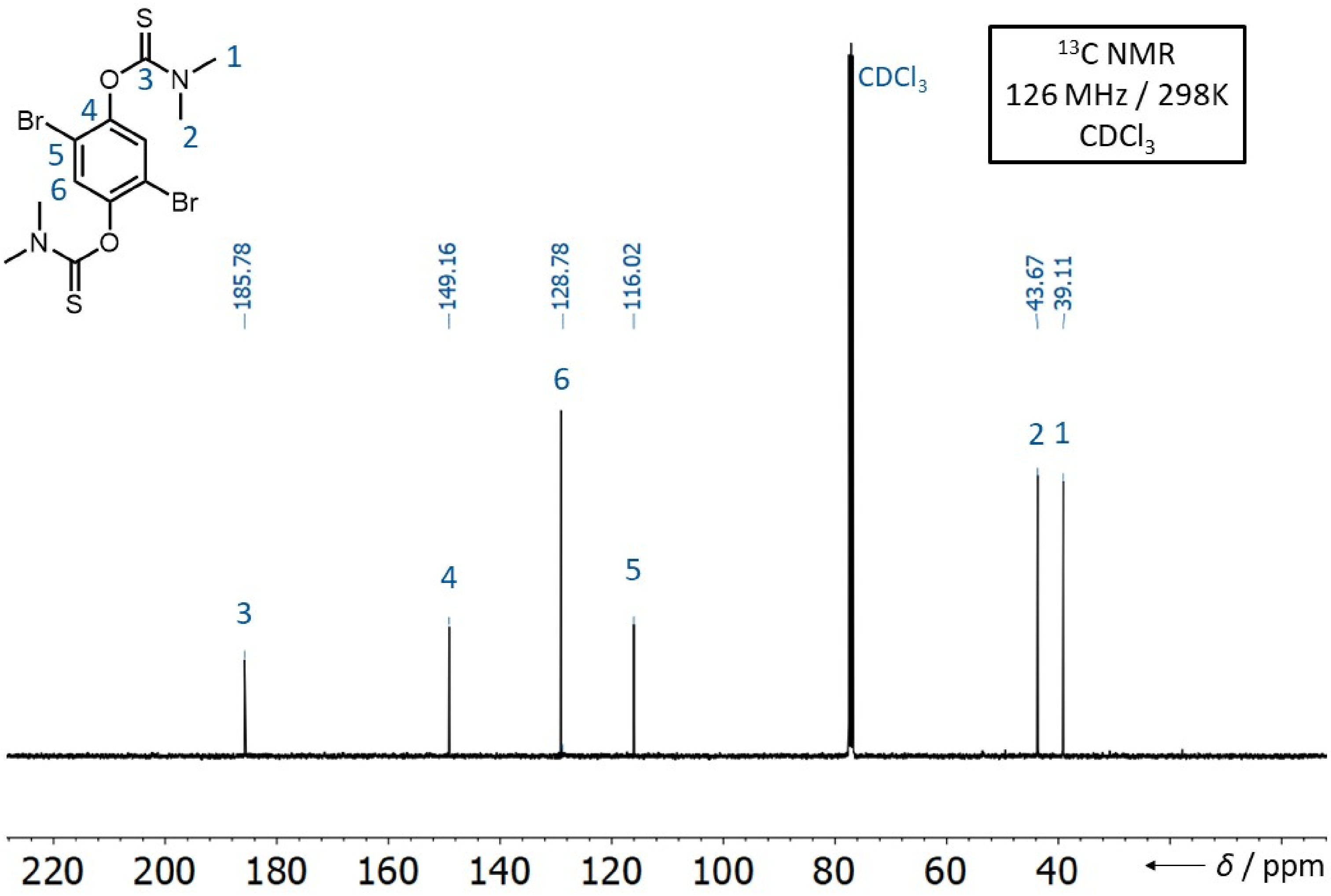

Figure S21. ^{13}C NMR Spectrum of ***O,O'*-(2,5-dibromo-1,4-phenylene)*bis*(dimethylcarbamothioate)**.

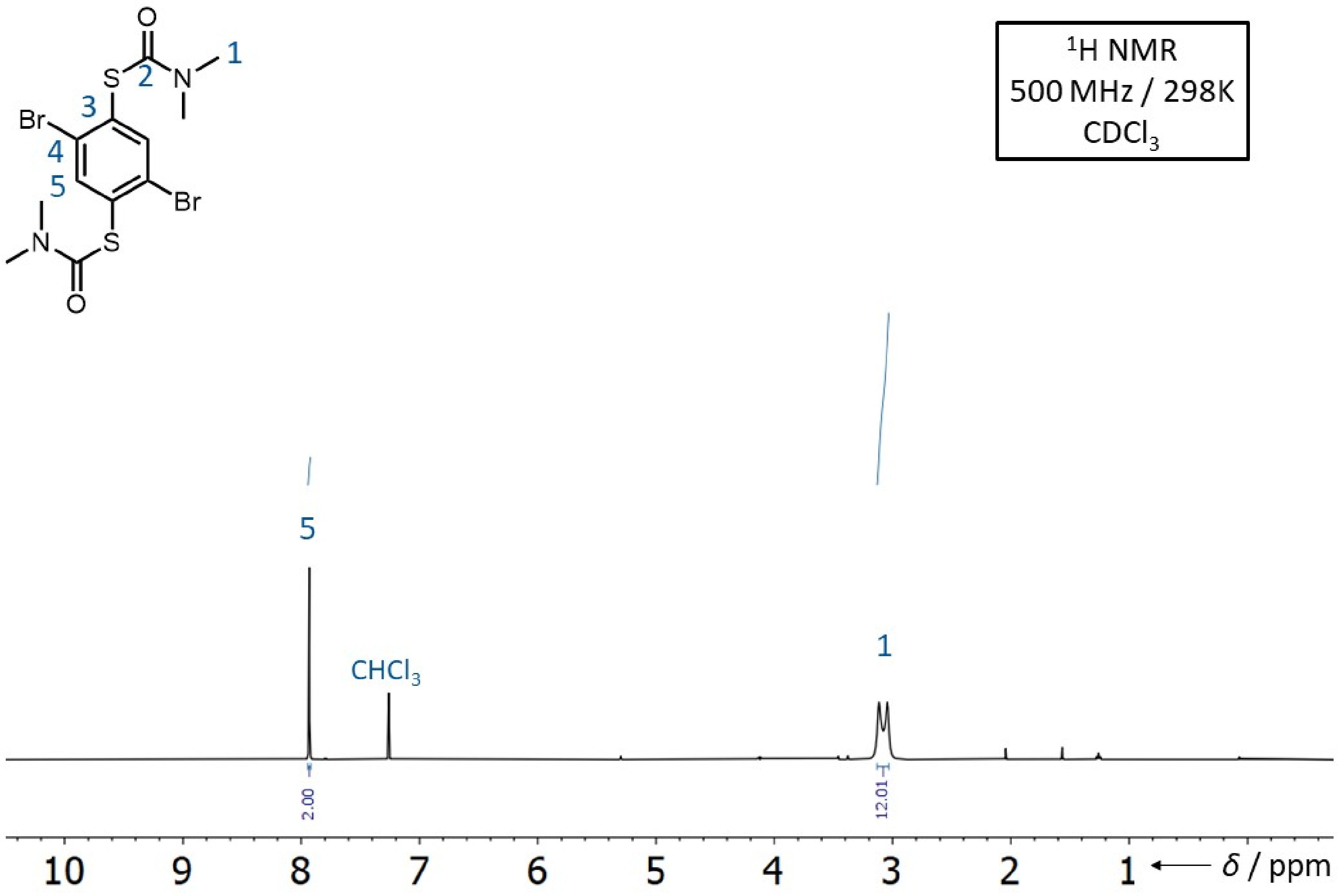

Figure S22. ^{1}H NMR Spectrum of ***S,S'*-(2,5-dibromo-1,4-phenylene)*bis*(dimethylcarbamothioate)**.

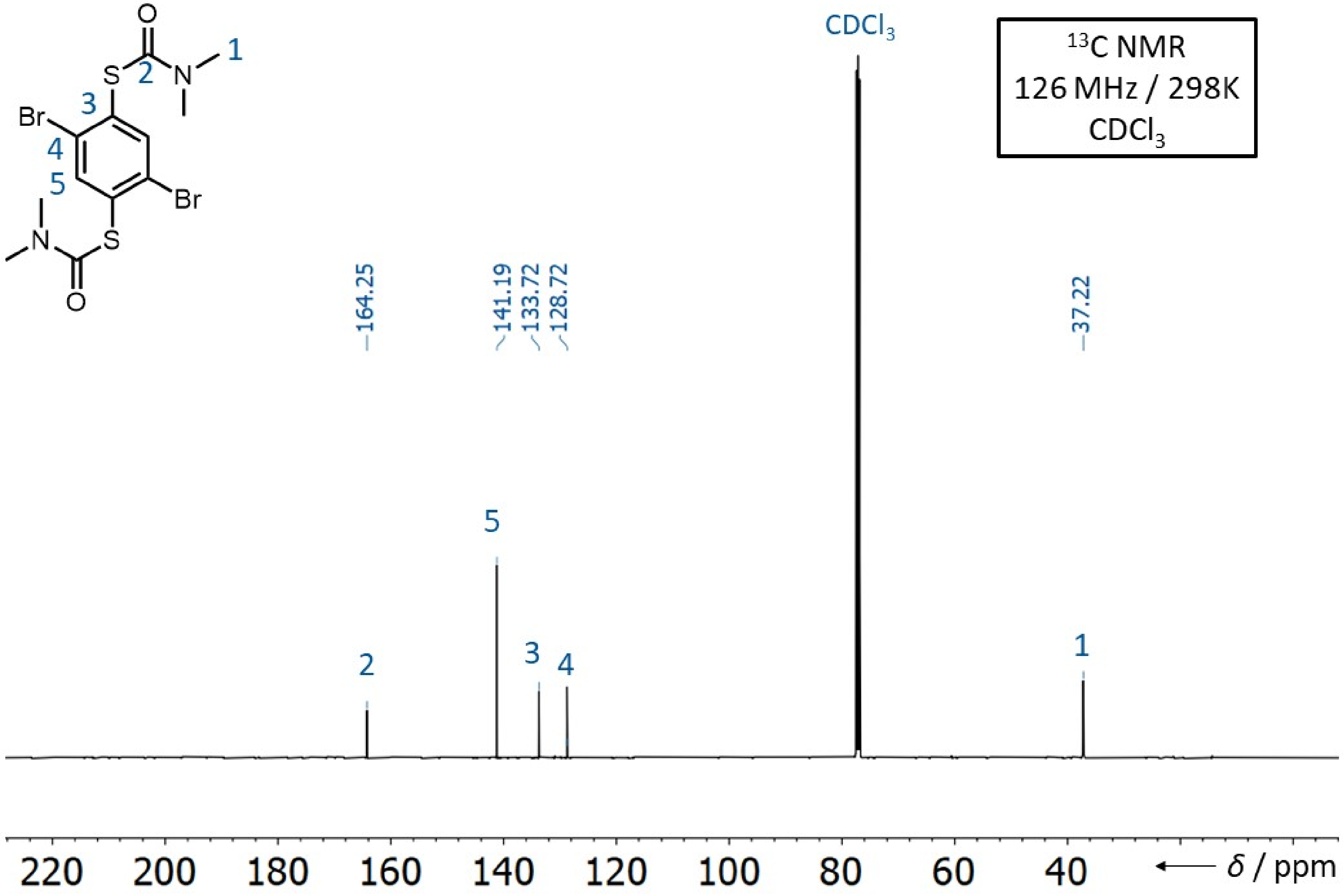

Figure S23. ^{13}C NMR Spectrum of ***S,S'*-(2,5-dibromo-1,4-phenylene)*bis*(dimethylcarbamothioate)**.

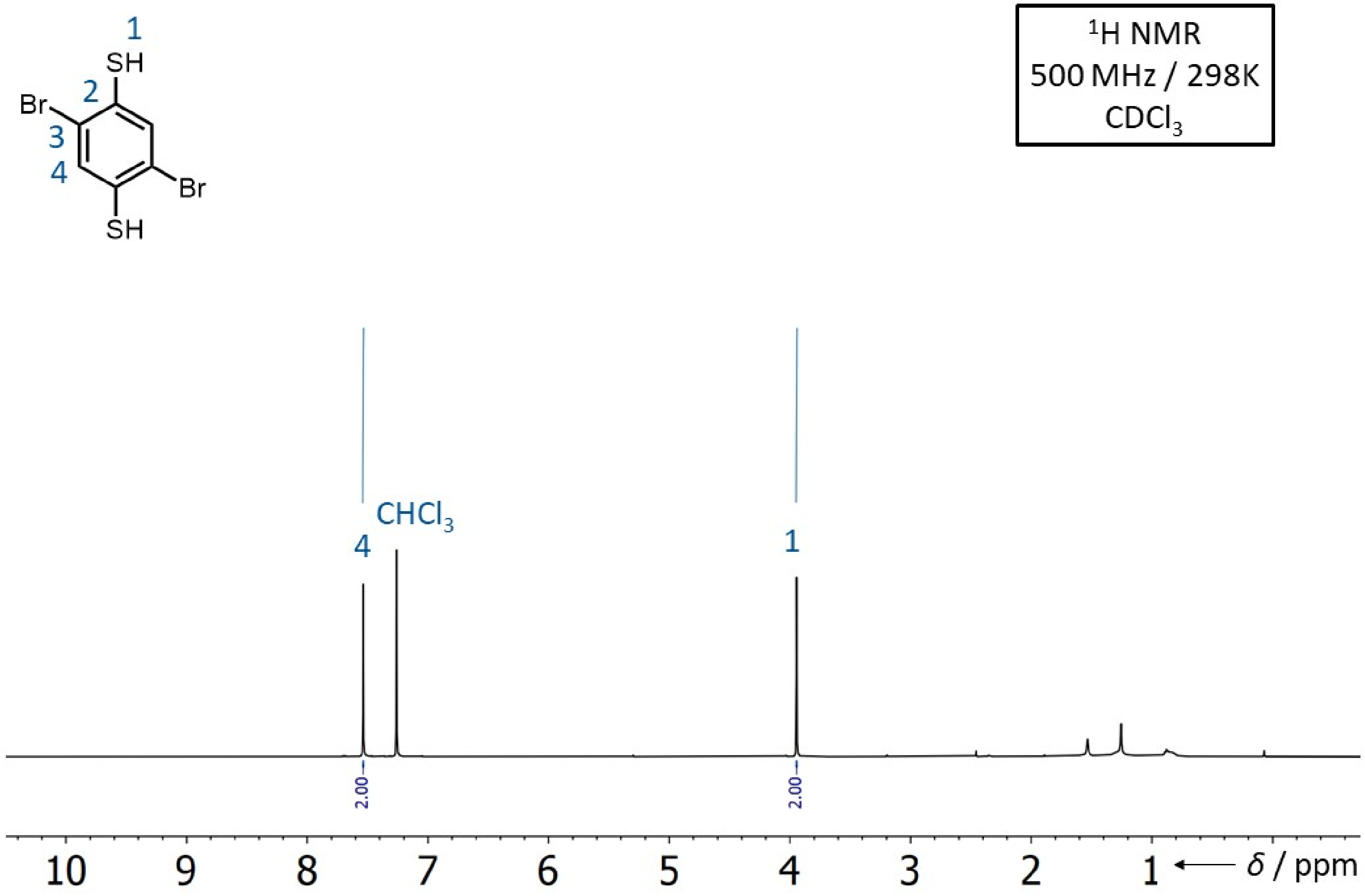

Figure S24. ^{1}H NMR Spectrum of **2,5-dibromobenzene-1,4-dithiol (2b)**.

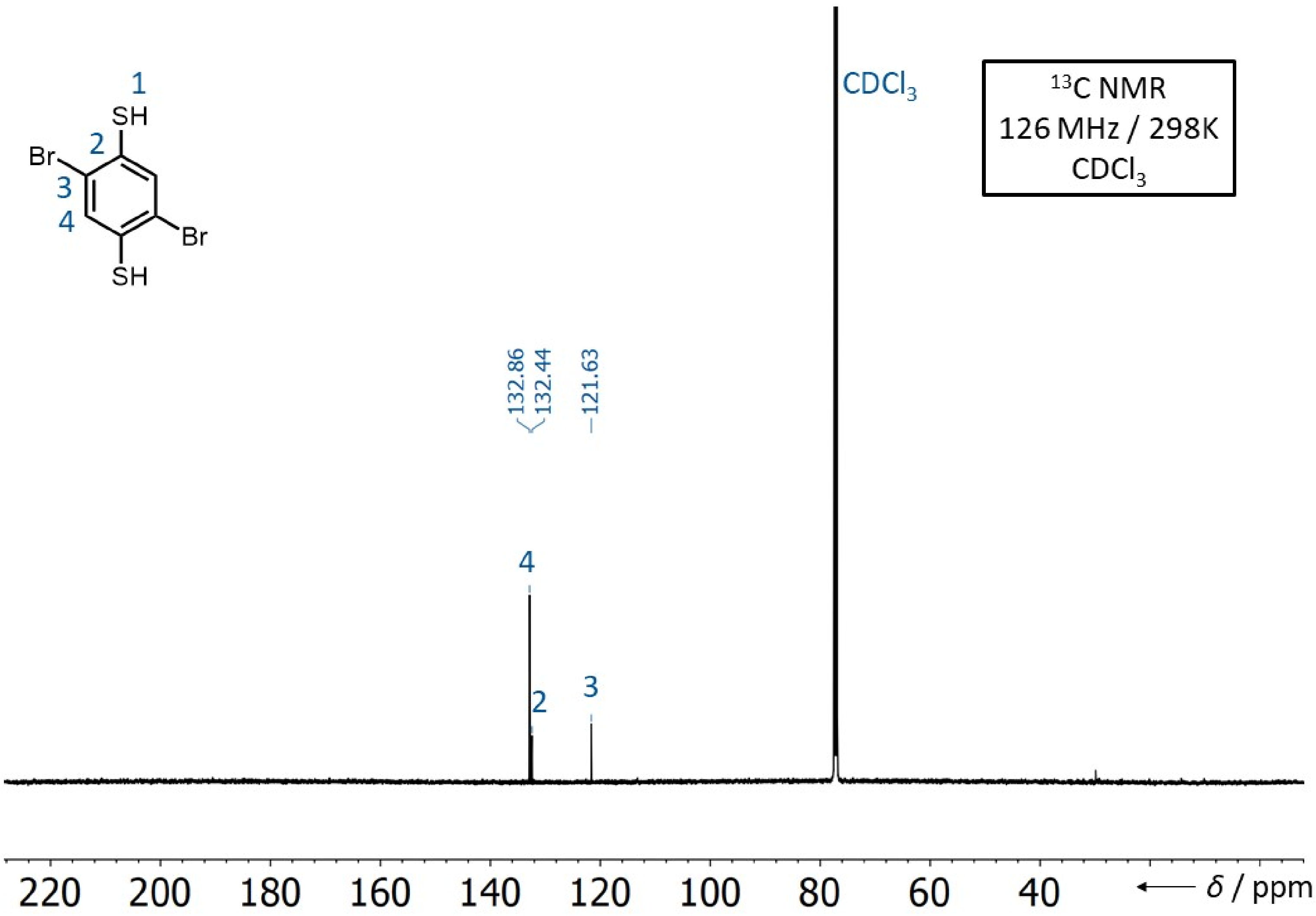

.

Figure S25. ^{13}C NMR Spectrum of **2,5-dibromobenzene-1,4-dithiol (2b)**.

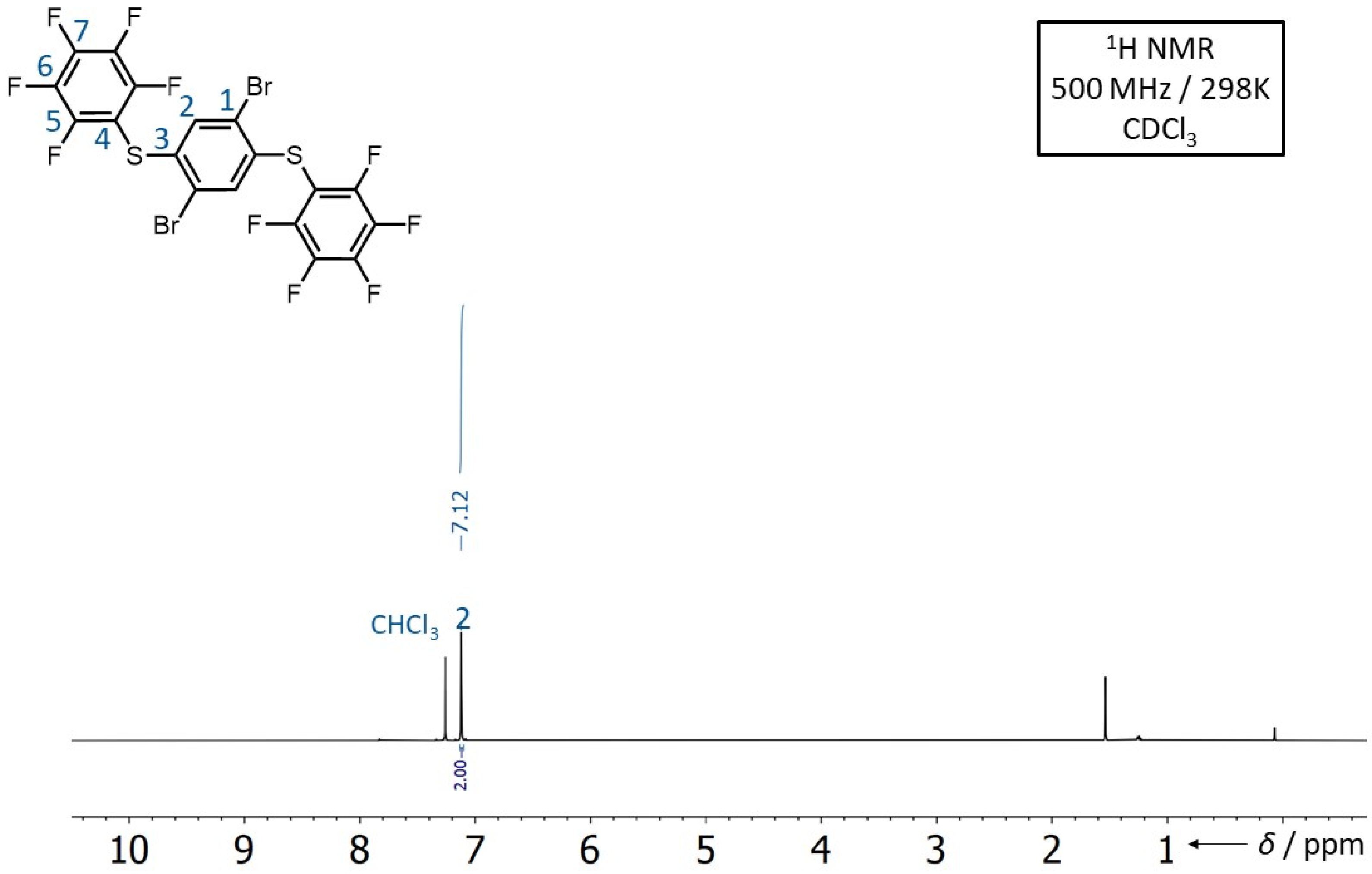

Figure S26. ^{1}H NMR Spectrum of **3c**.

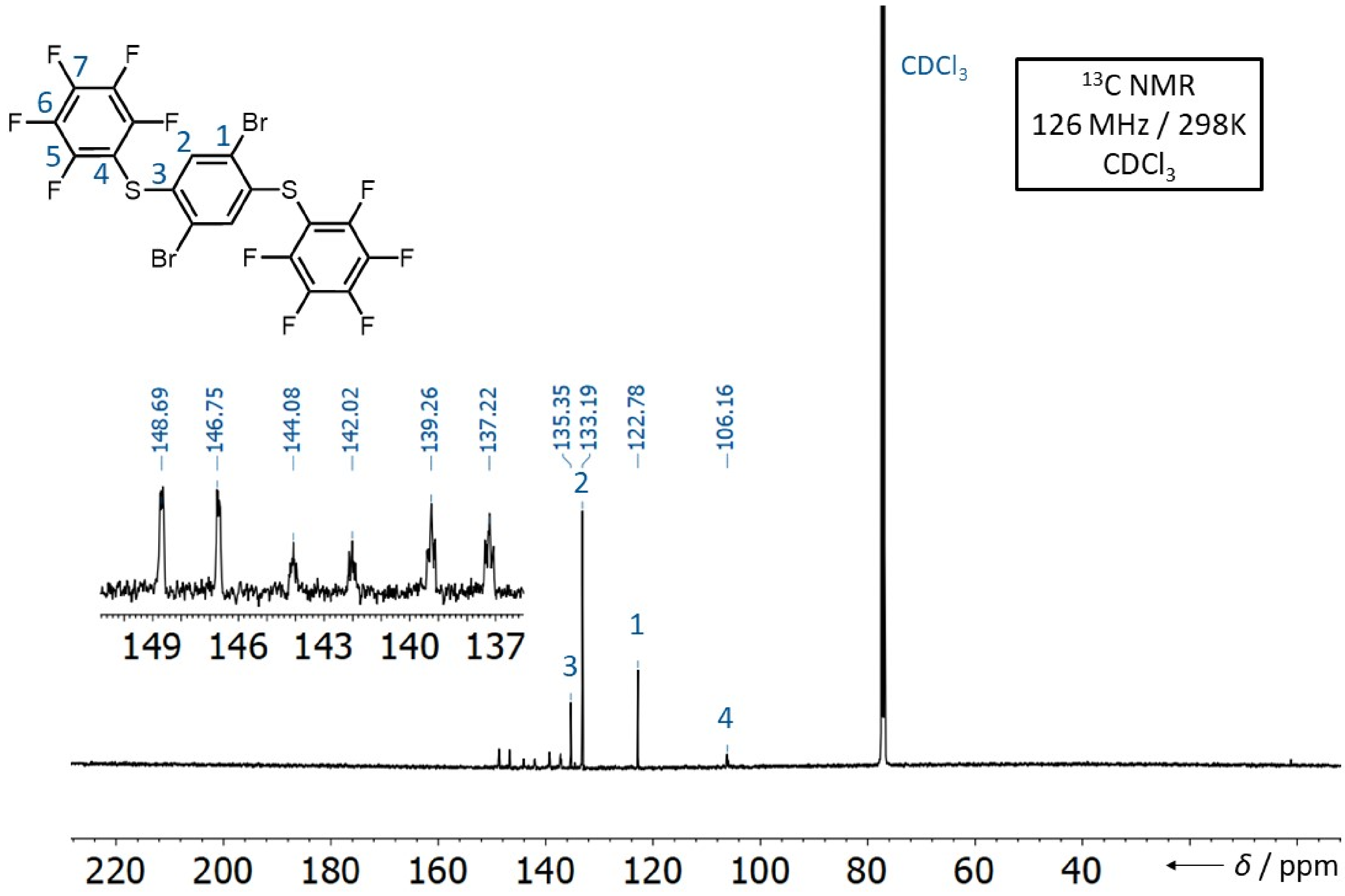

Figure S27. ^{13}C NMR Spectrum of **3c**.

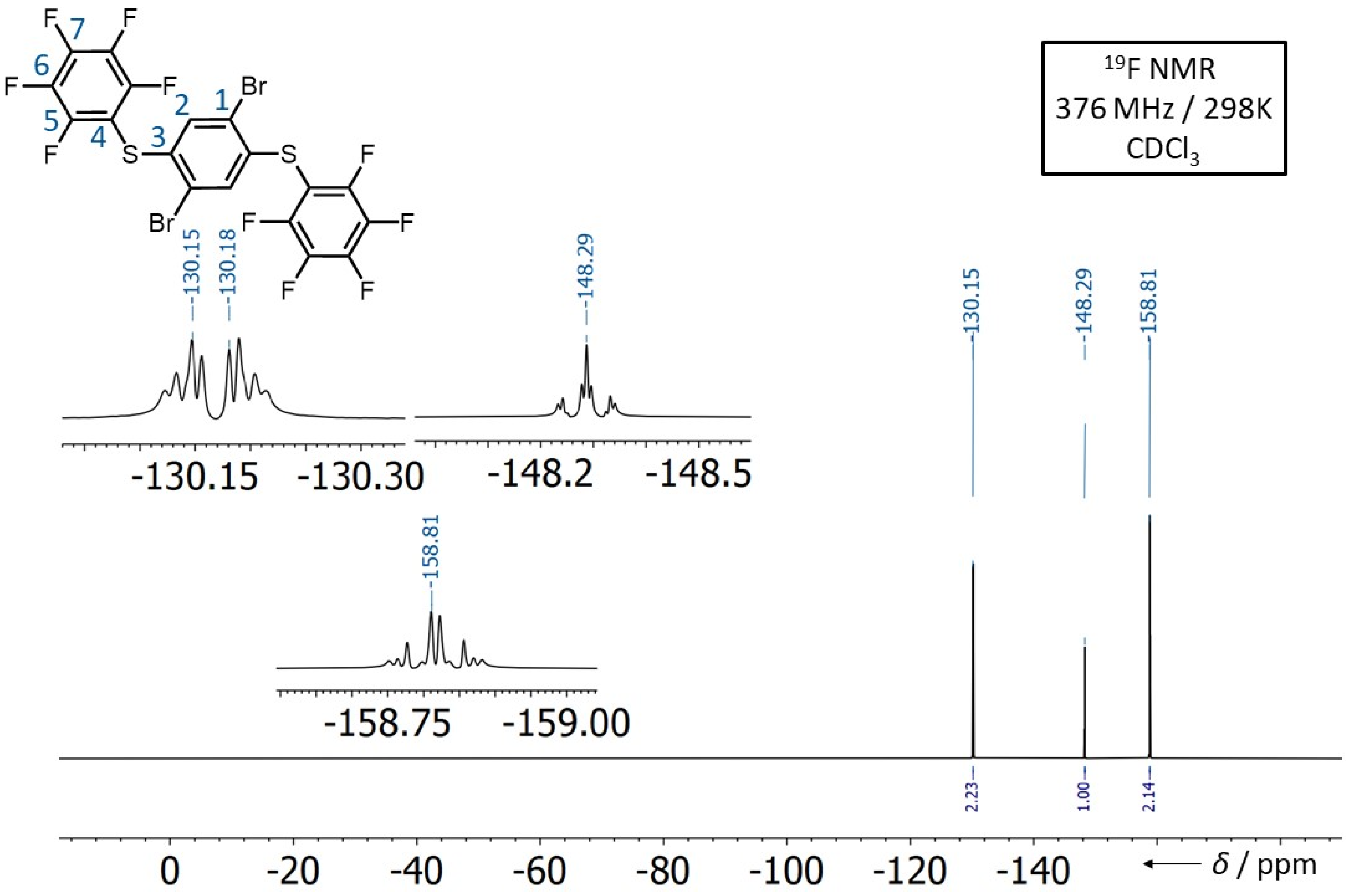

Figure S28. ^{19}F NMR Spectrum of **3c**.

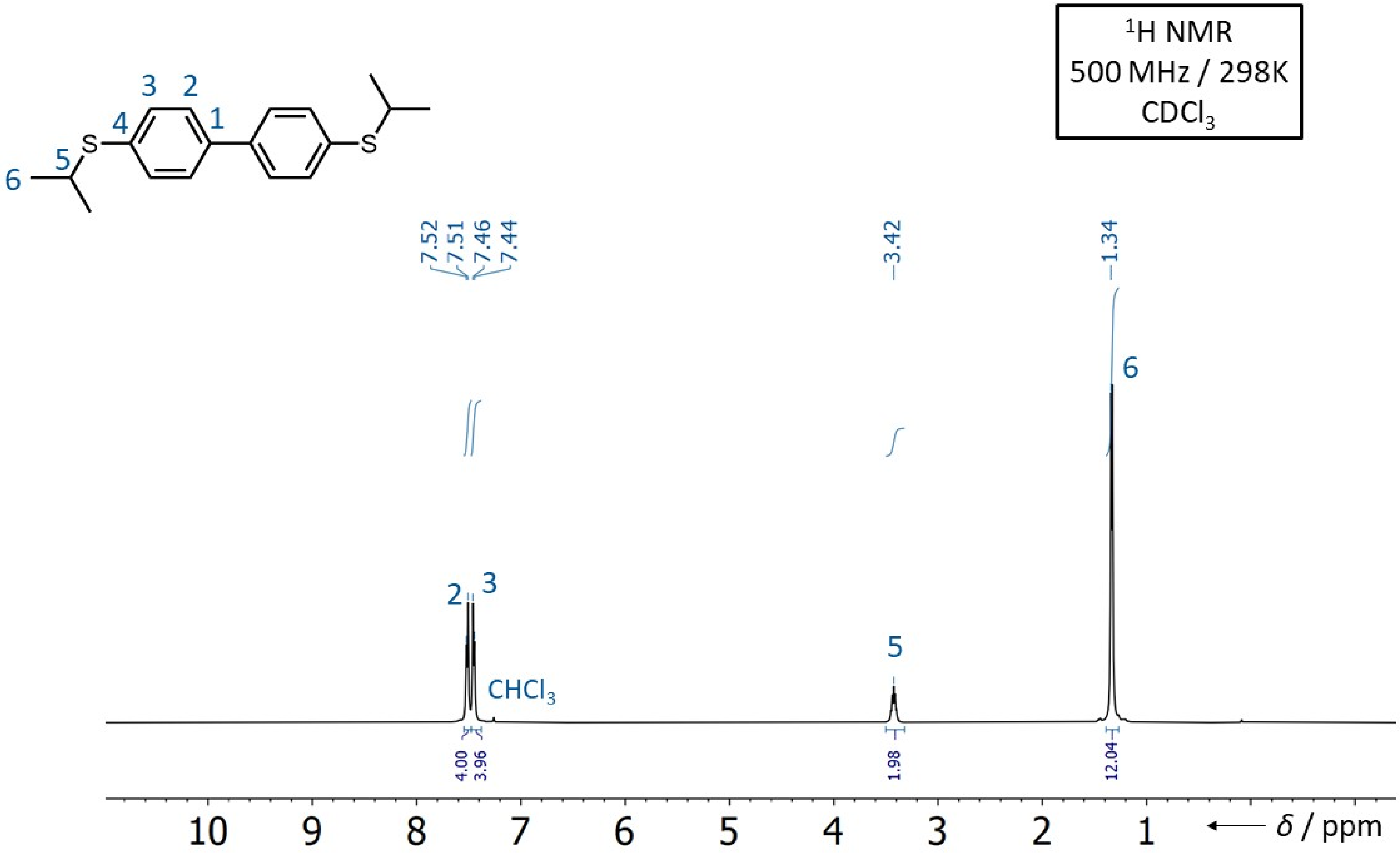

Figure S29. ^{1}H NMR Spectrum of **4,4'-*bis*(isopropylthio)-1,1'-biphenyl**.

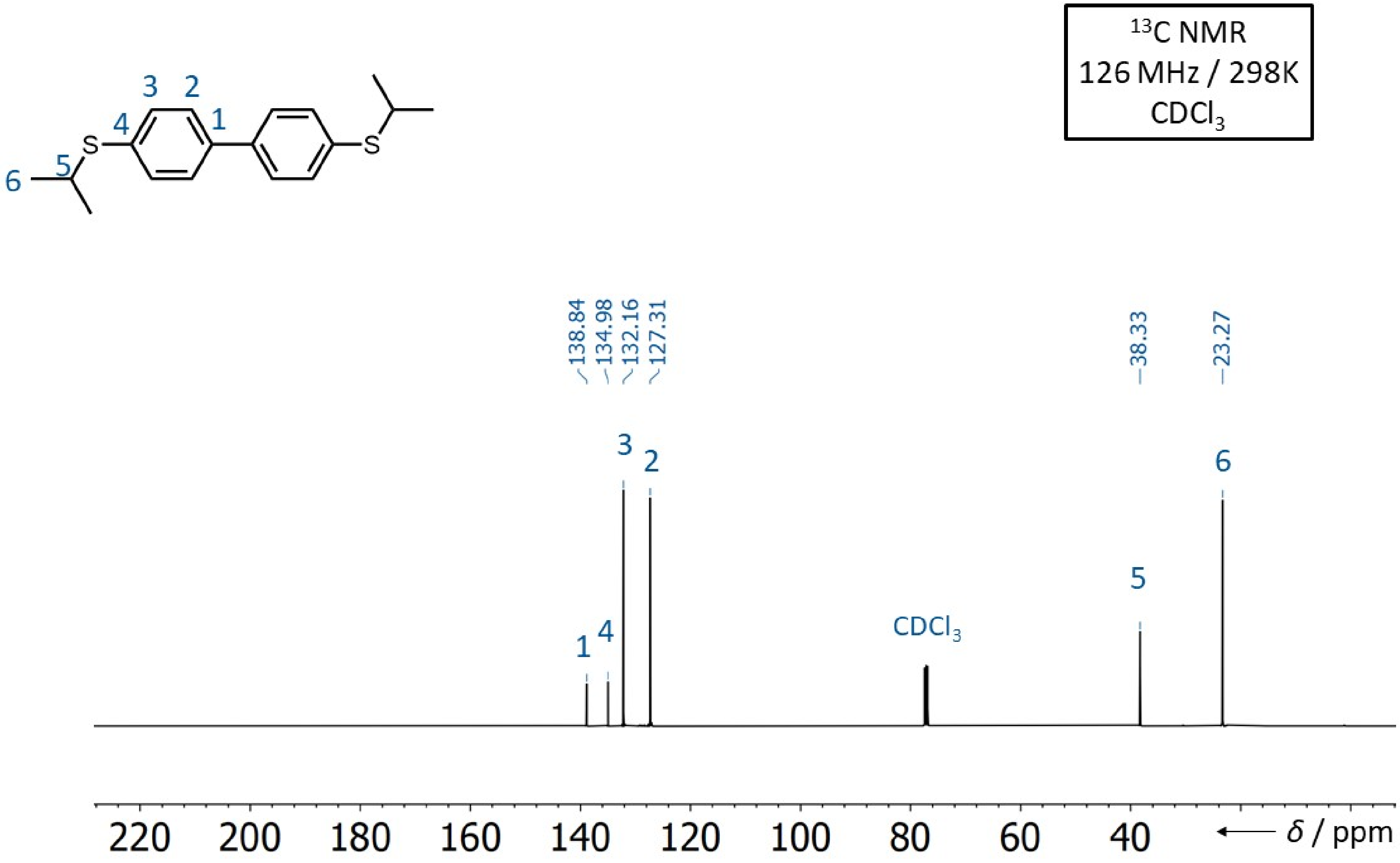

Figure S30. ^{13}C NMR Spectrum of **4,4'-*bis*(isopropylthio)-1,1'-biphenyl**.

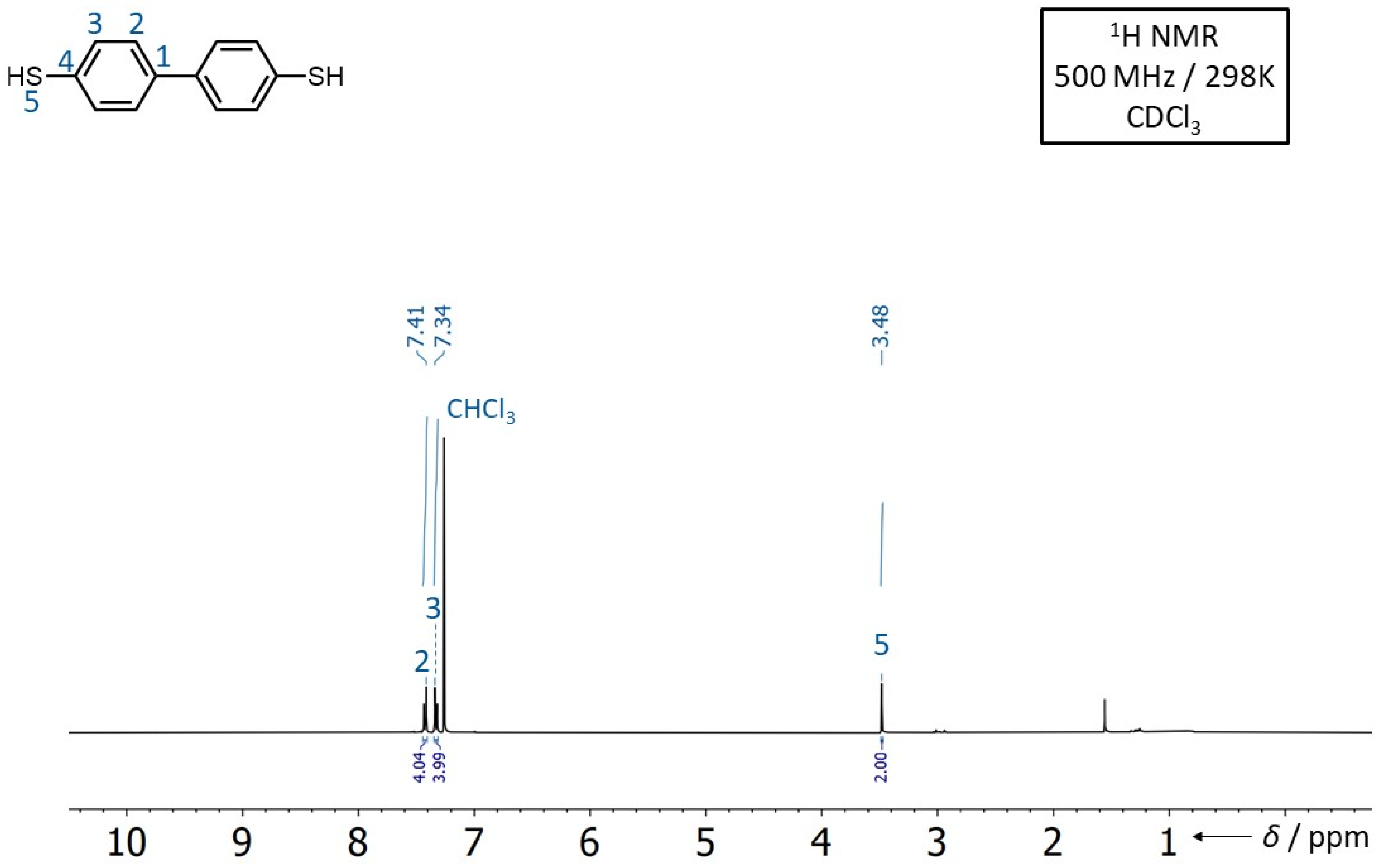

Figure S31. ^{1}H NMR Spectrum of **biphenyl-4,4′-dithiol** (**2d**).

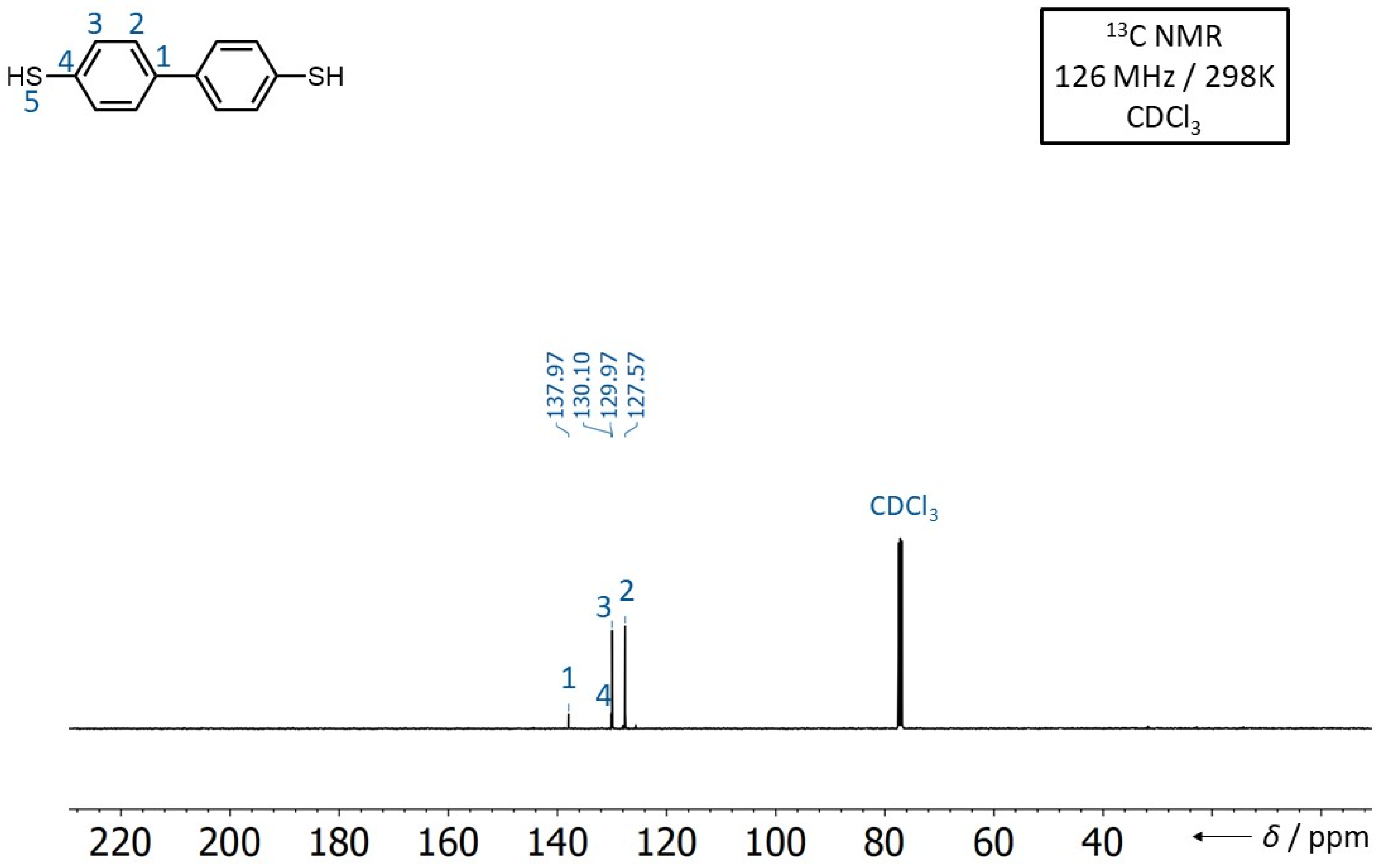

Figure S32. ^{13}C NMR Spectrum of **biphenyl-4,4′-dithiol** (**2d**).

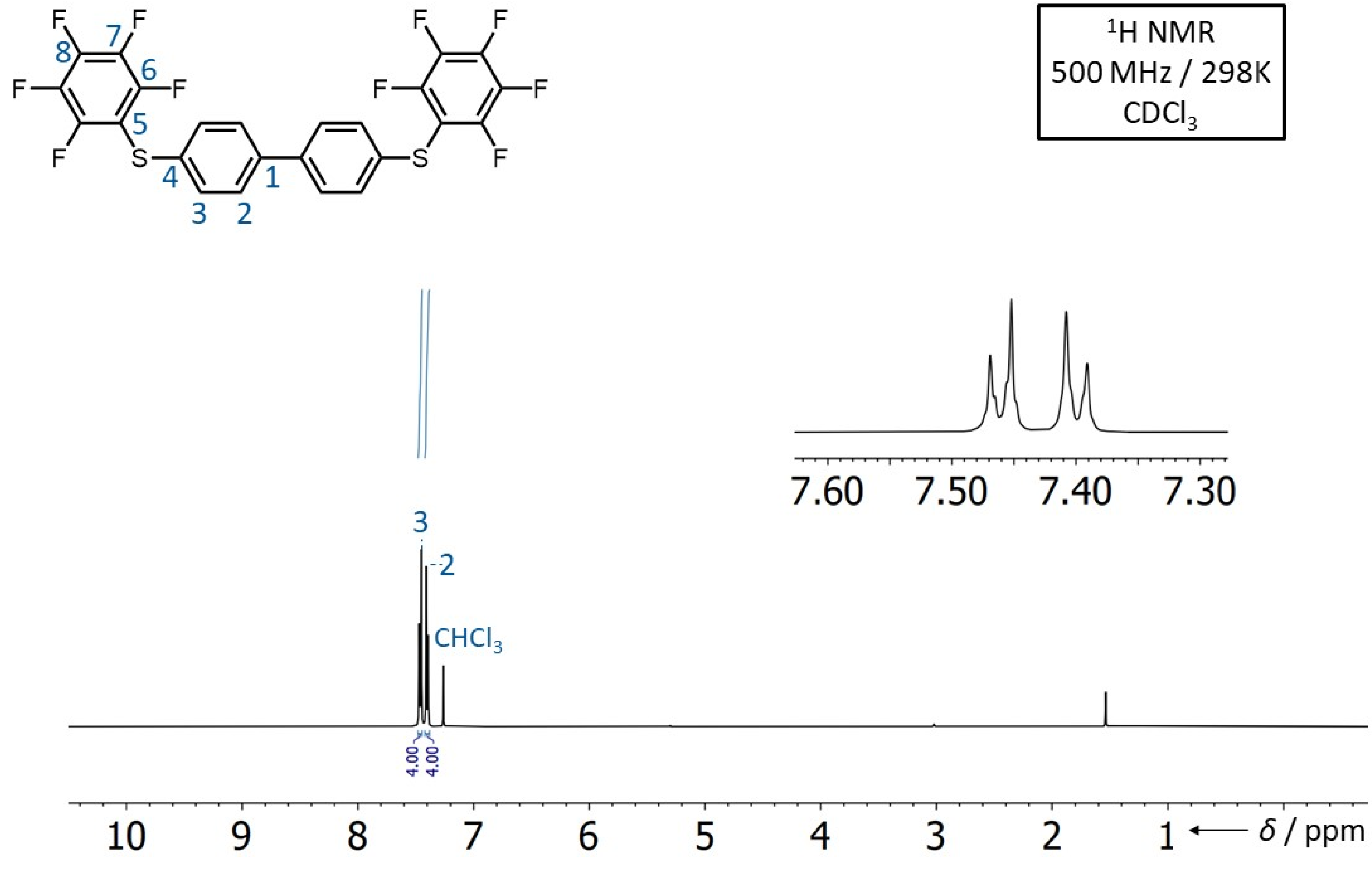

Figure S33. ^{1}H NMR Spectrum of **3d**.

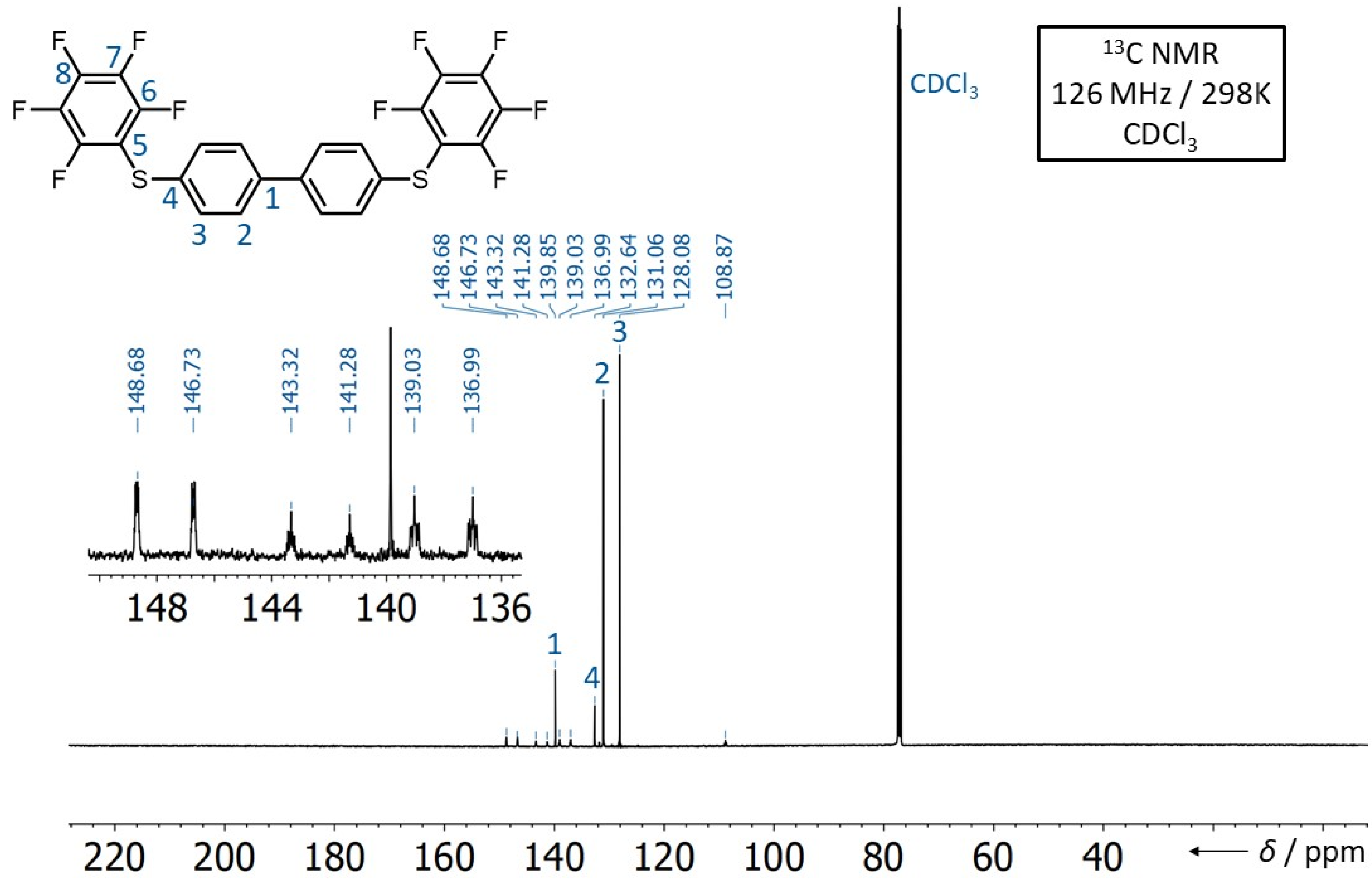

Figure S34. ^{13}C NMR Spectrum of **3d**.

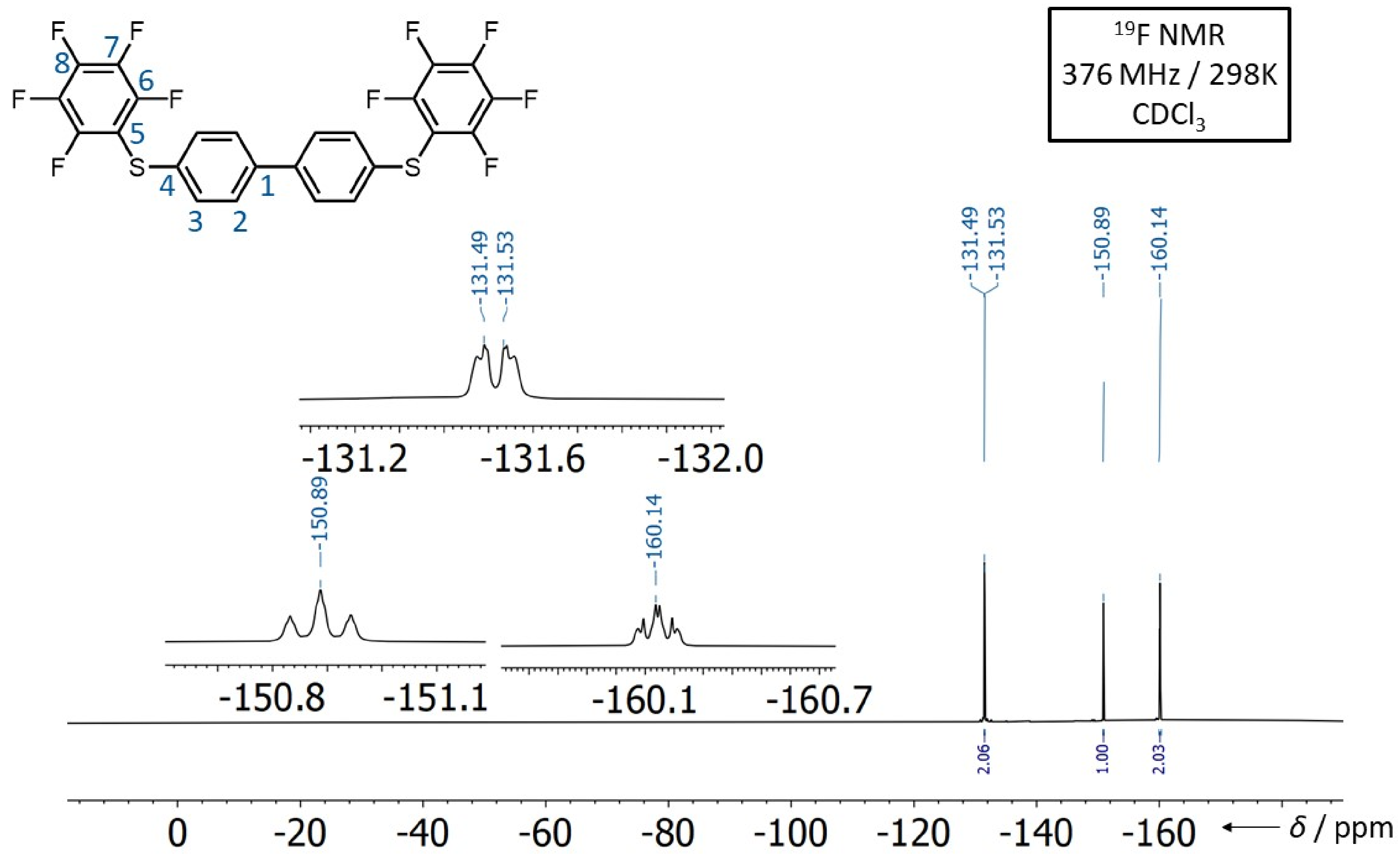

Figure S35. ^{19}F NMR Spectrum of **3d**.

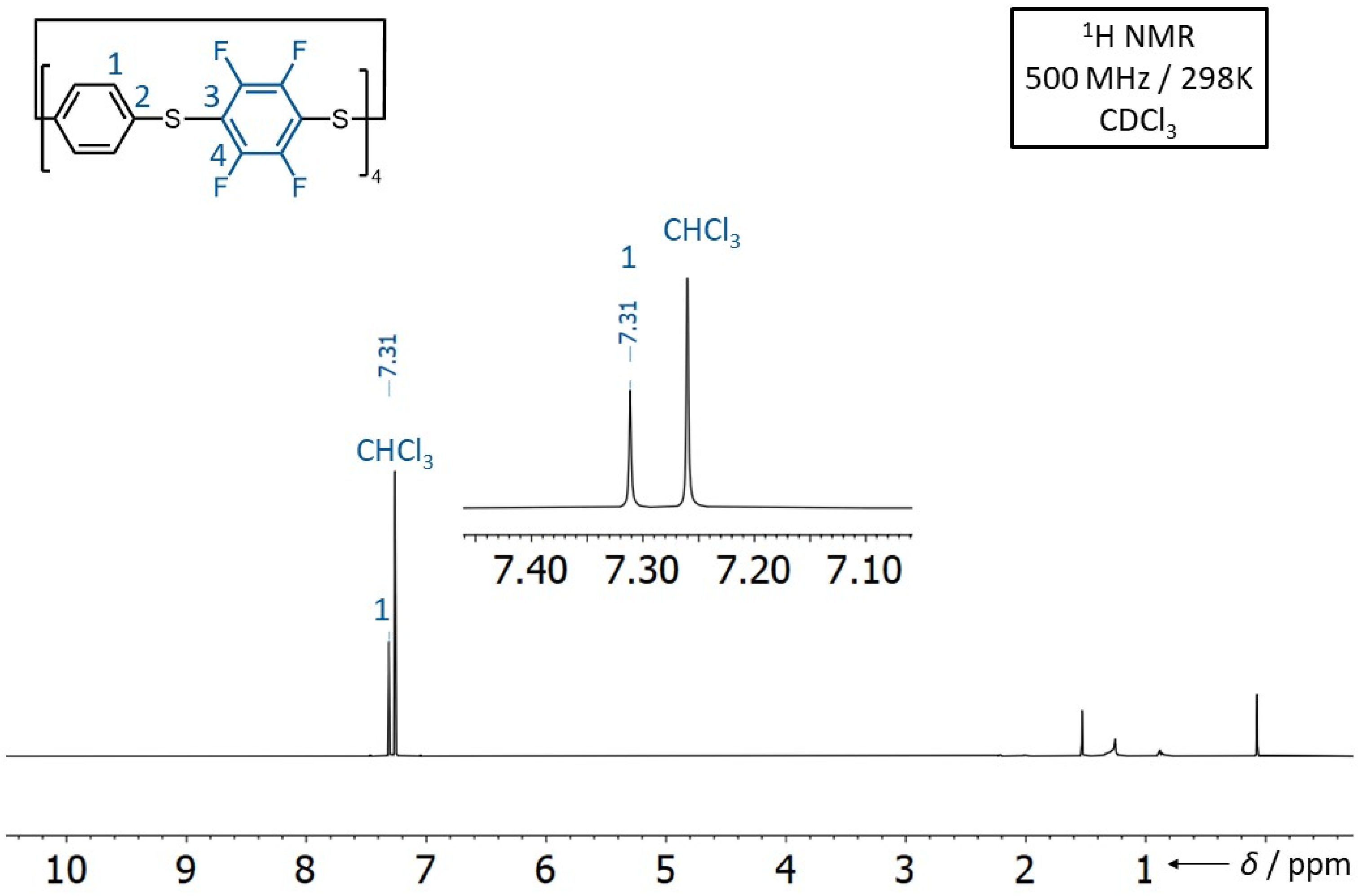

Figure S36. ^{1}H NMR Spectrum of **1a**.

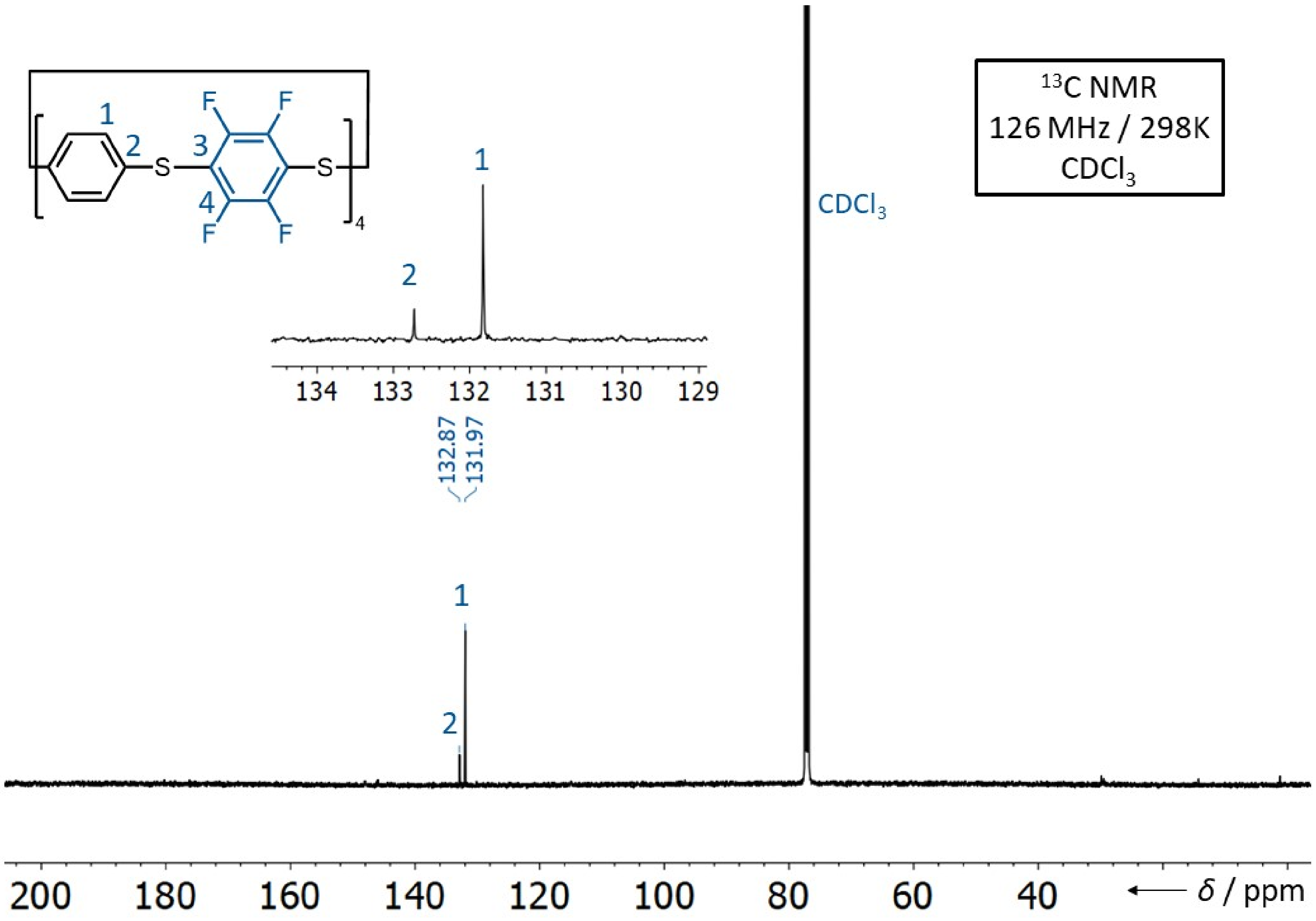

Figure S37. ^{13}C NMR Spectrum of **1a**.

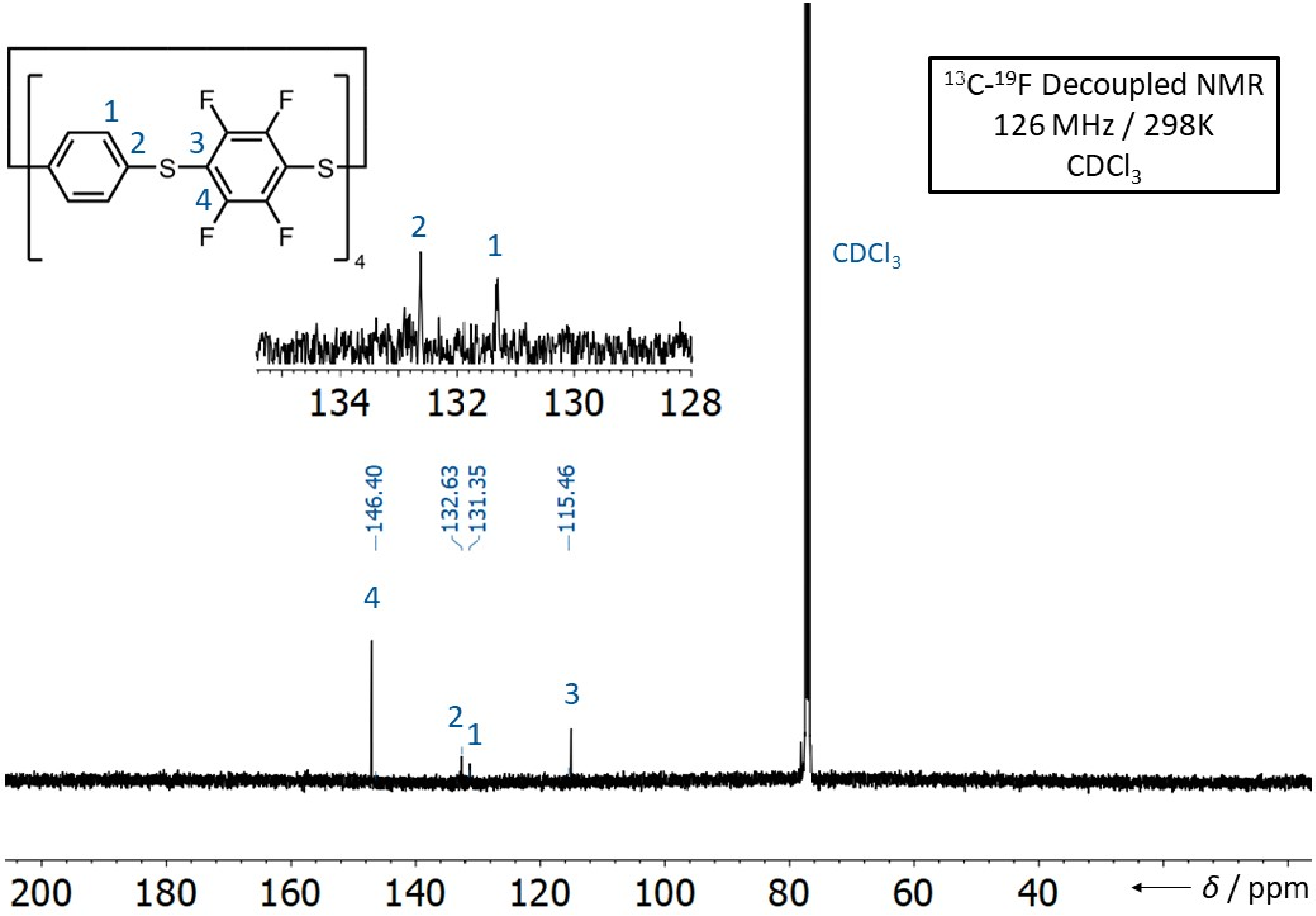

Figure S38. ^{13}C-^{19}F Decoupled NMR Spectrum of **1a**.

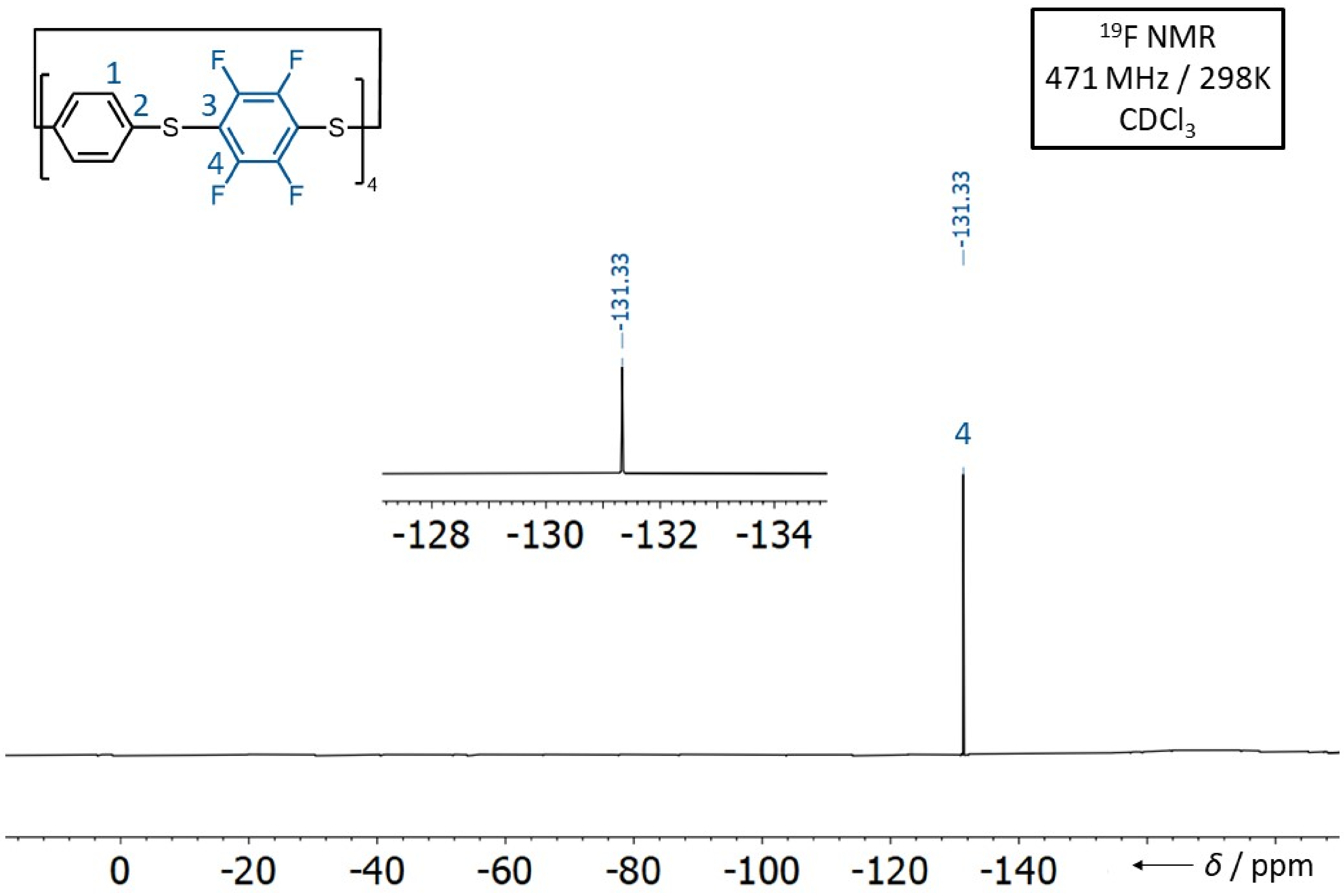

Figure S39. ^{19}F NMR Spectrum of **1a**.

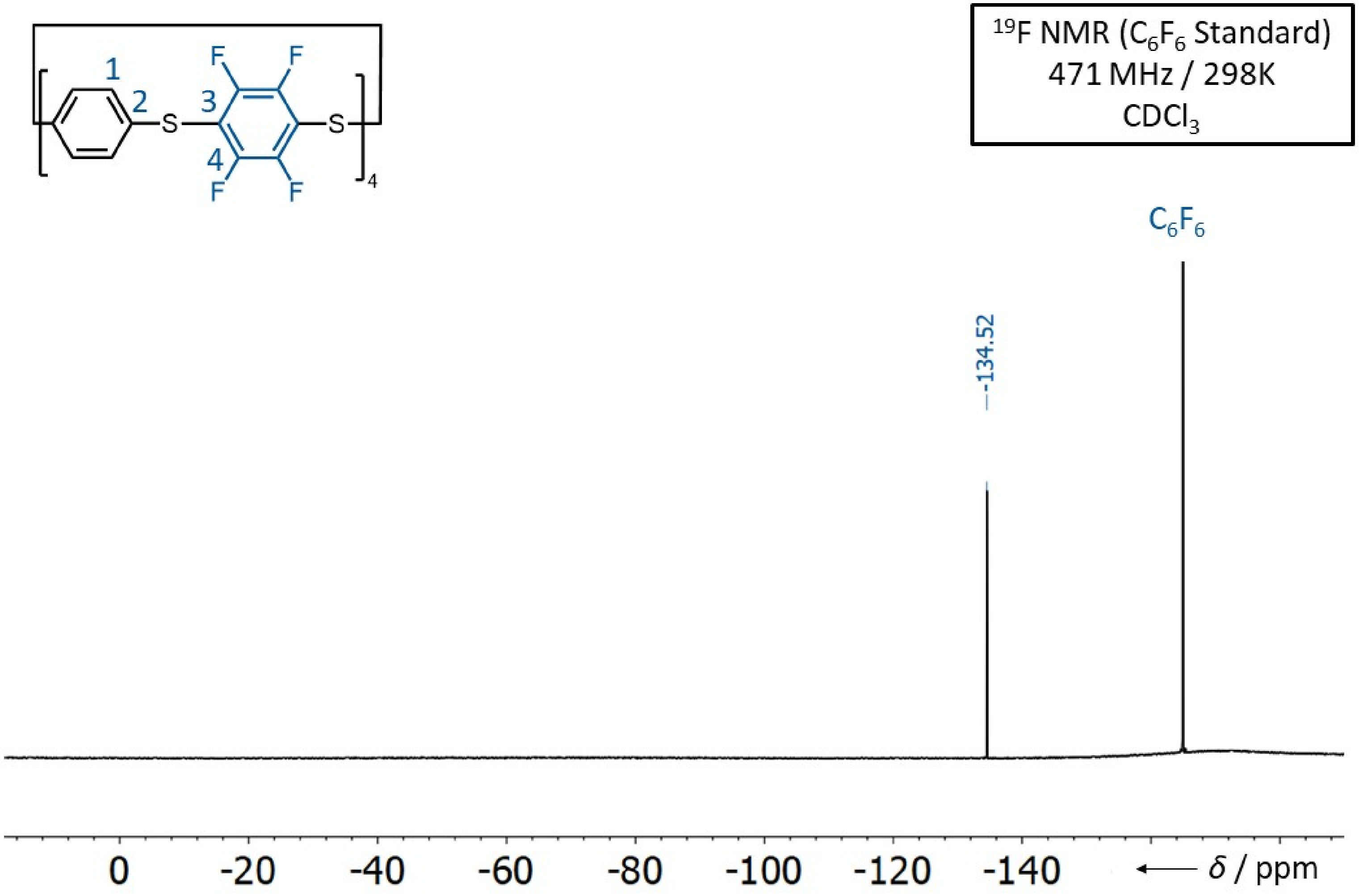

Figure S40. Referenced ^{19}F NMR Spectrum of **1a**.

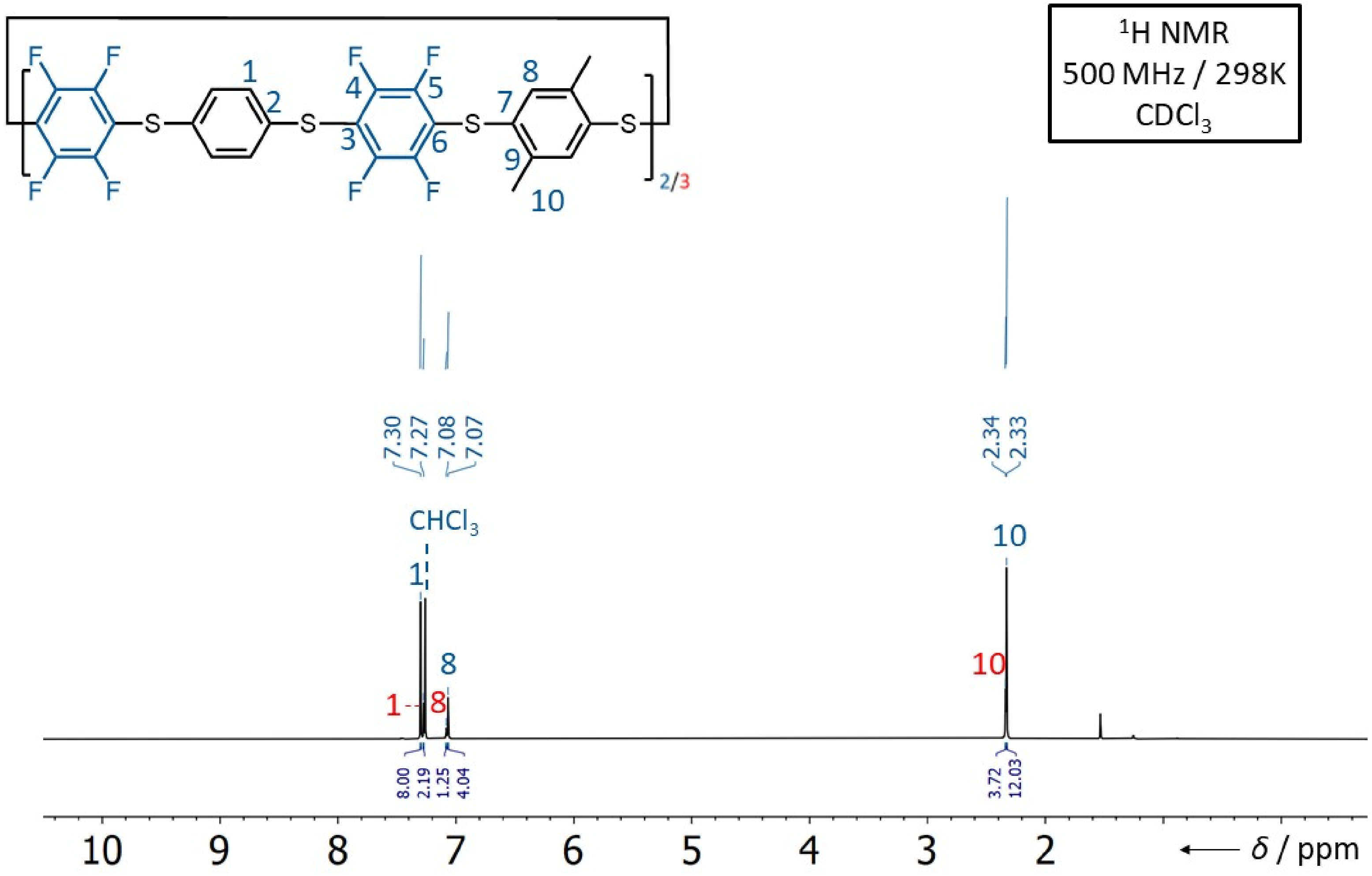

Figure S41. ^{1}H NMR Spectrum of **1ab**.

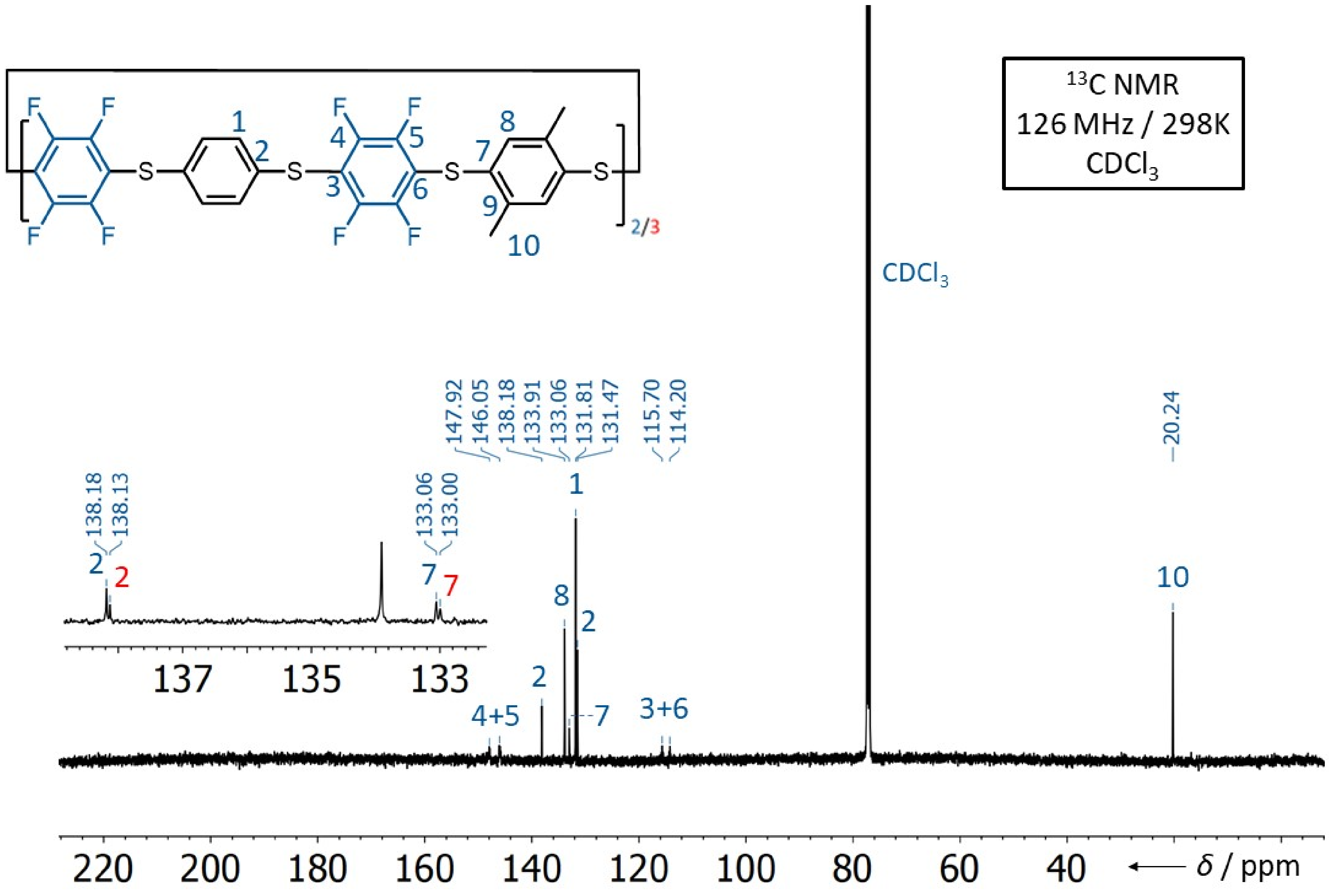

Figure S42. ^{13}C NMR Spectrum of **1ab**.

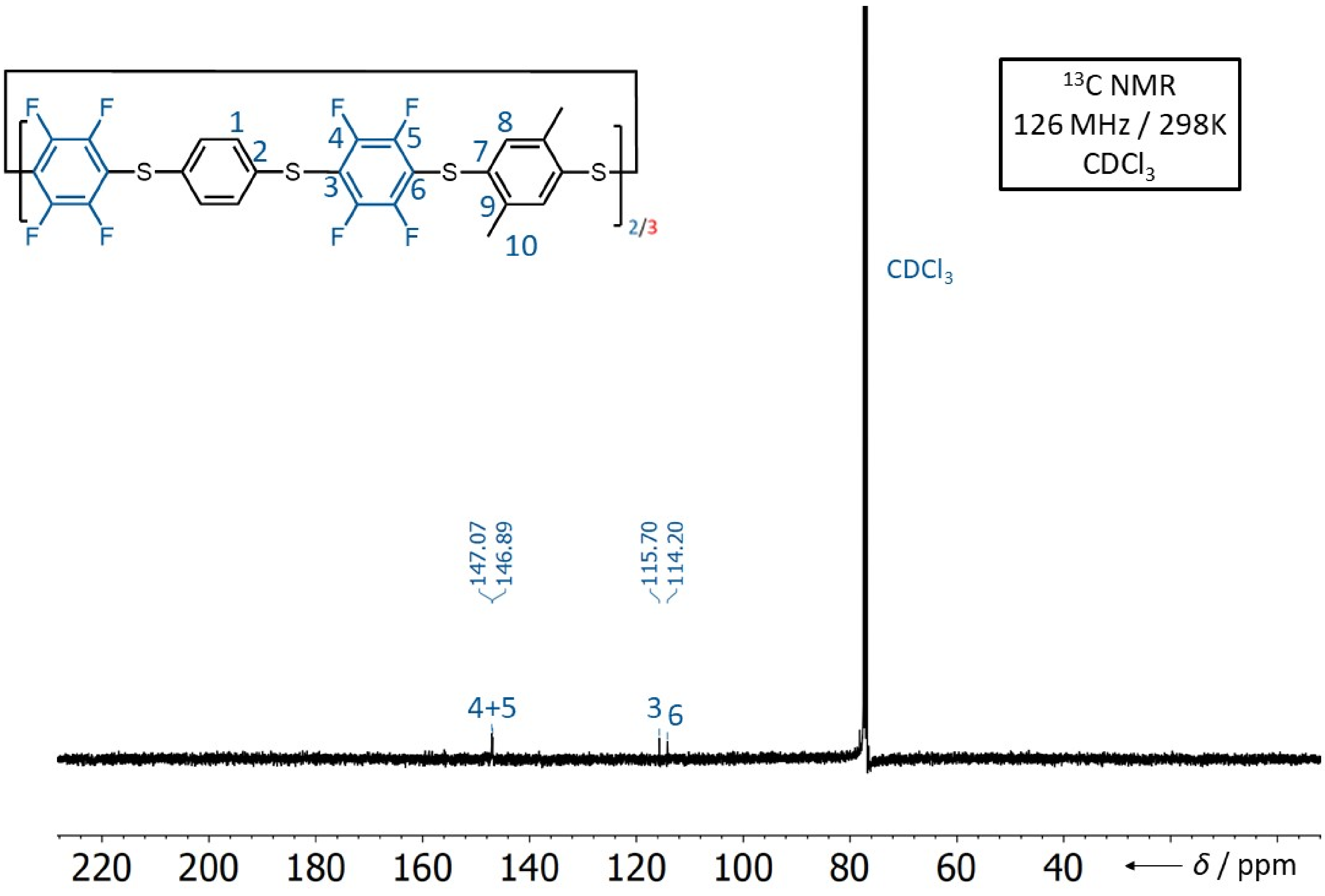

Figure S43. ^{13}C-^{19}F Decoupled NMR Spectrum of **1ab**.

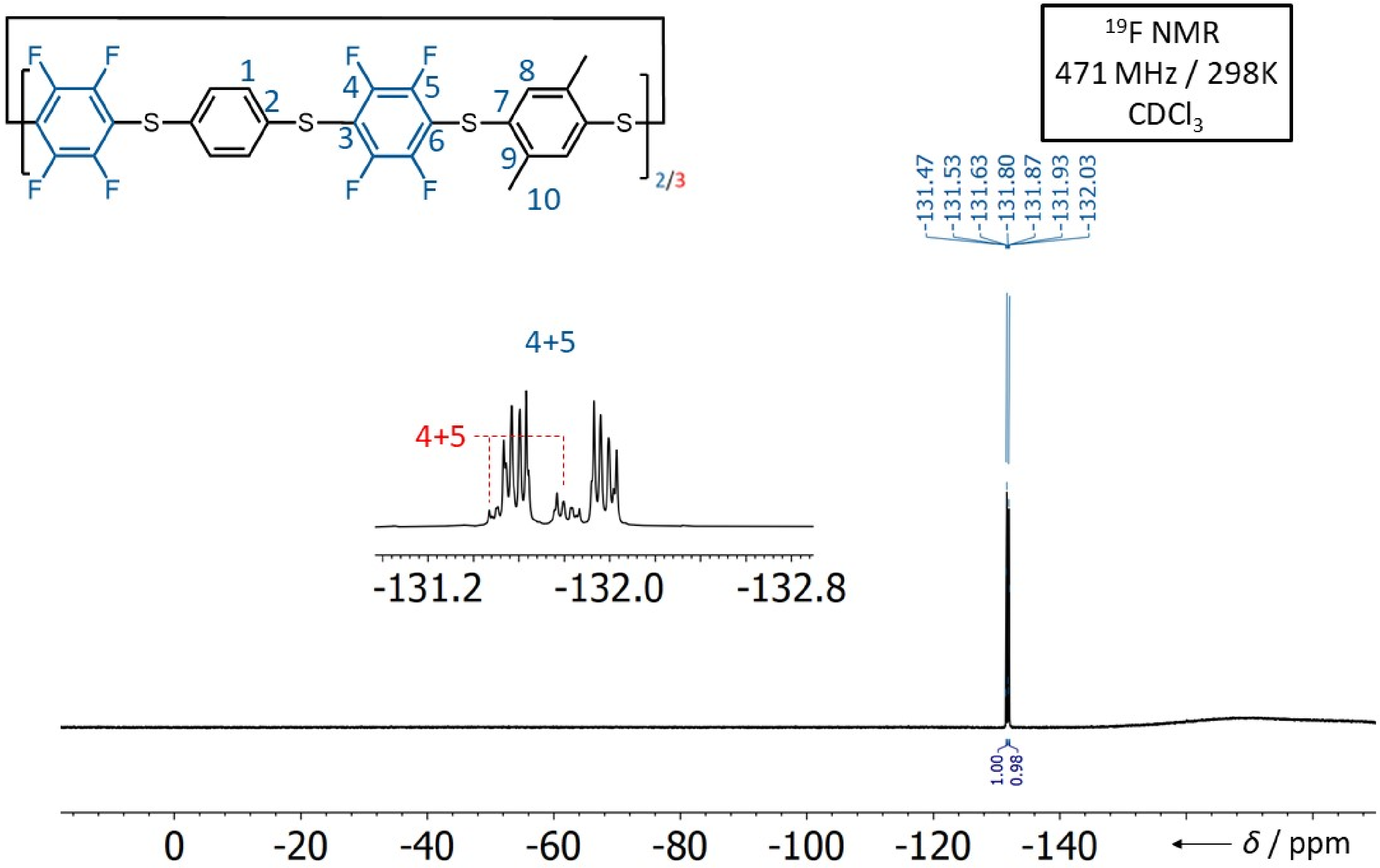

Figure S44. ^{19}F NMR Spectrum of **1ab**.

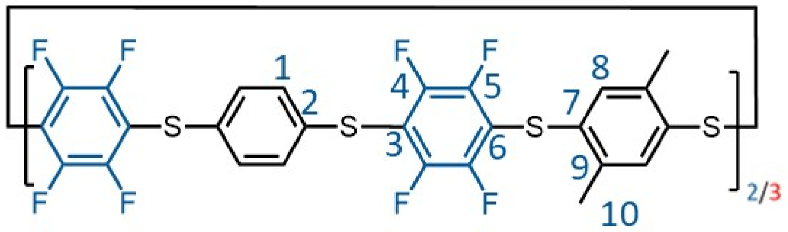

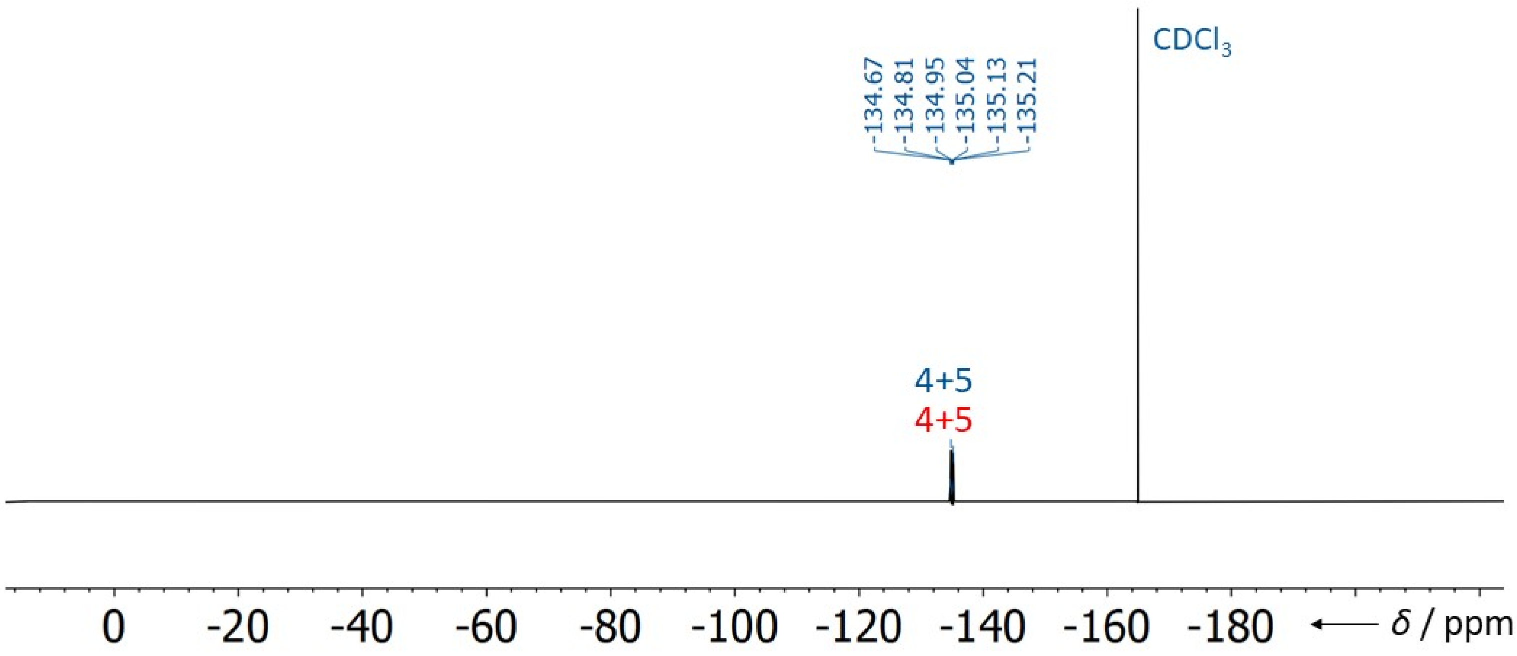

Figure S45. Referenced ^{19}F NMR Spectrum of **1ab**.

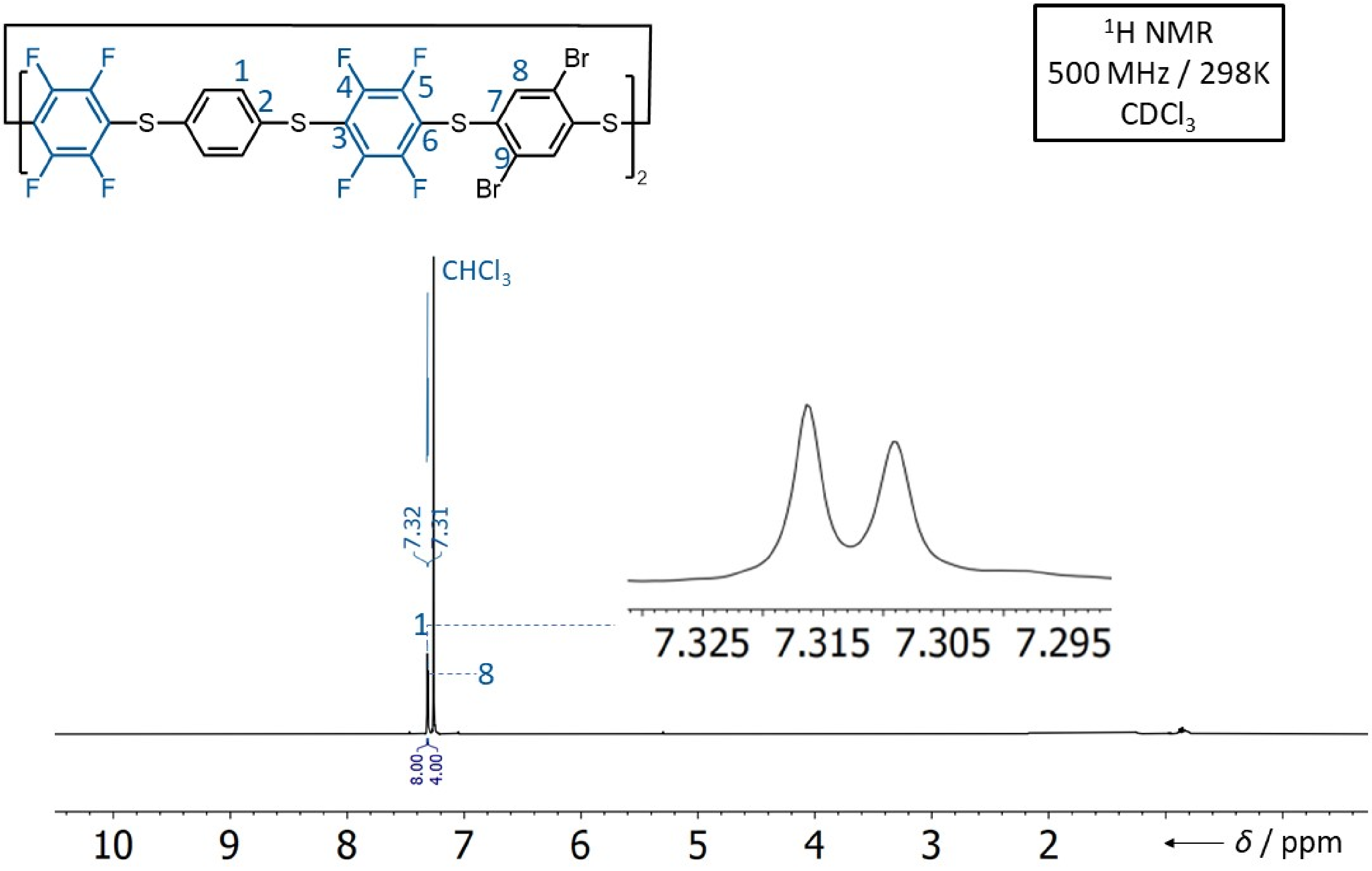

Figure S46. ^{1}H NMR Spectrum of **1ac**.

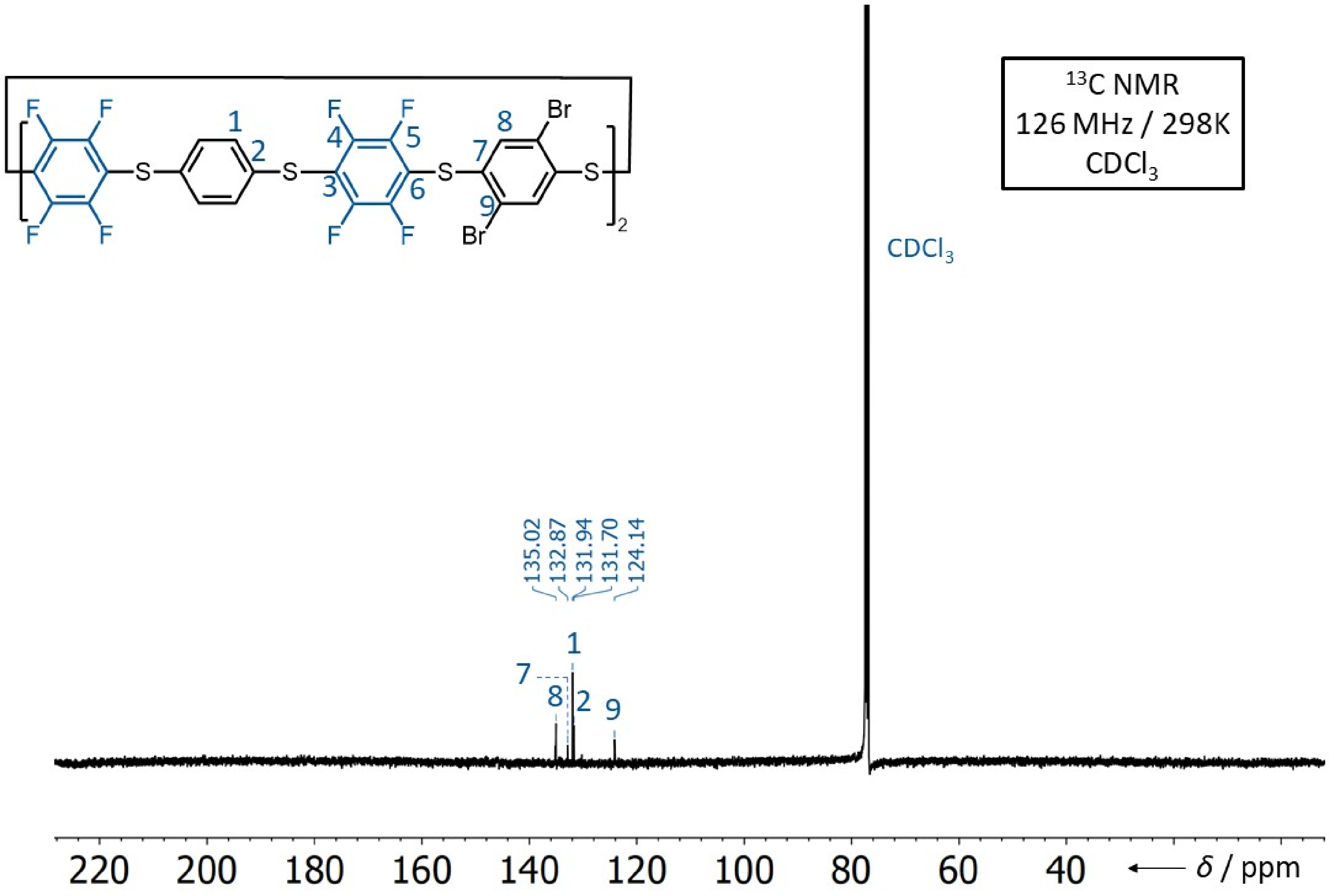

F
F
1
2
S
S
F
F
4
5
3
6
F
F
F
F
8
7
Br
S
S
9
Br
2
^{13}C NMR
126 MHz / 298K
$CDCl_3$
$CDCl_3$
135.02
132.87
131.94
131.70
124.14
7
8
1
2
9
220
200
180
160
140
120
100
80
60
40
δ / ppm

Figure S47. ^{13}C NMR Spectrum of **1ac**.

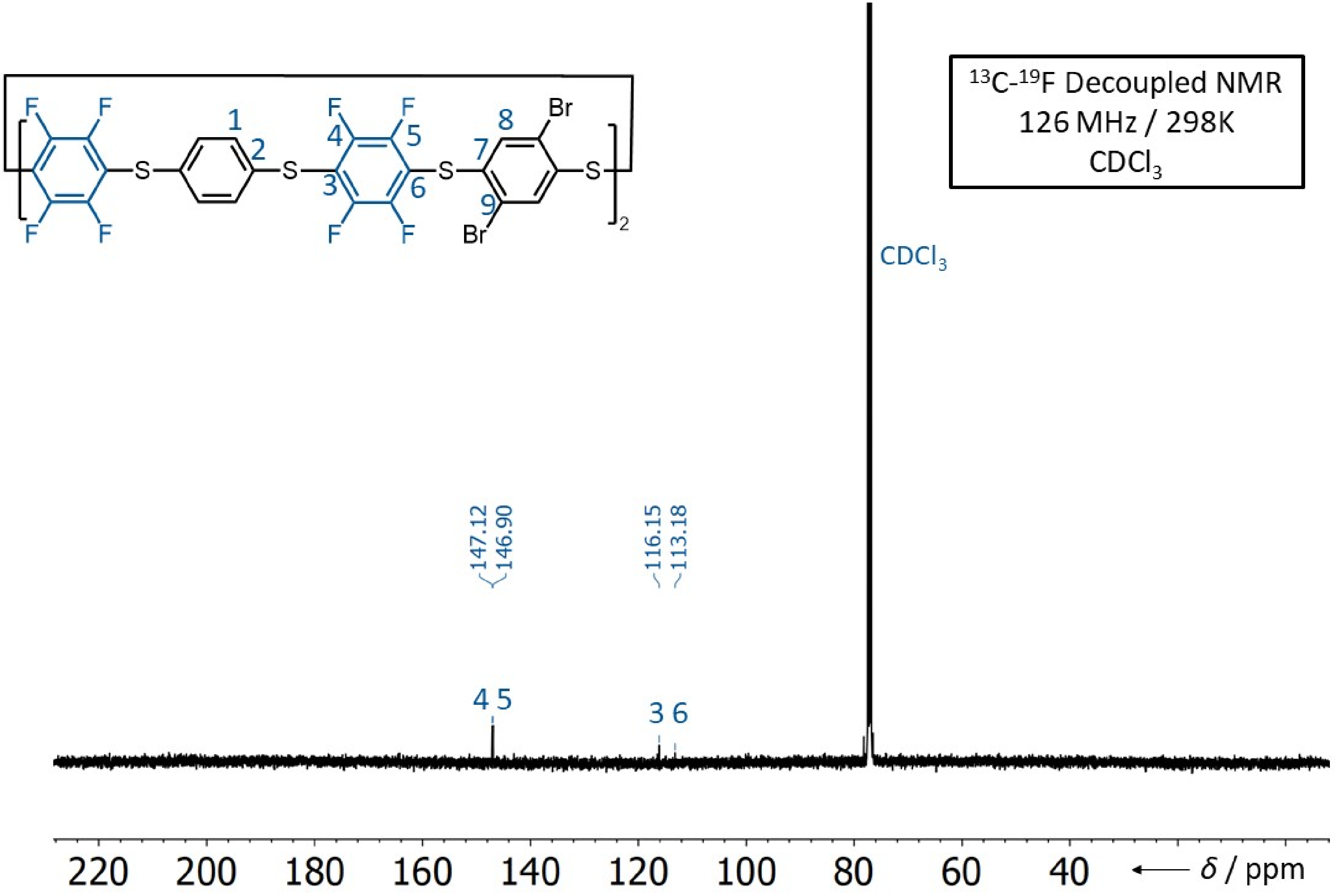

Figure S48. ^{13}C-^{19}F Decoupled NMR Spectrum of **1ac**.

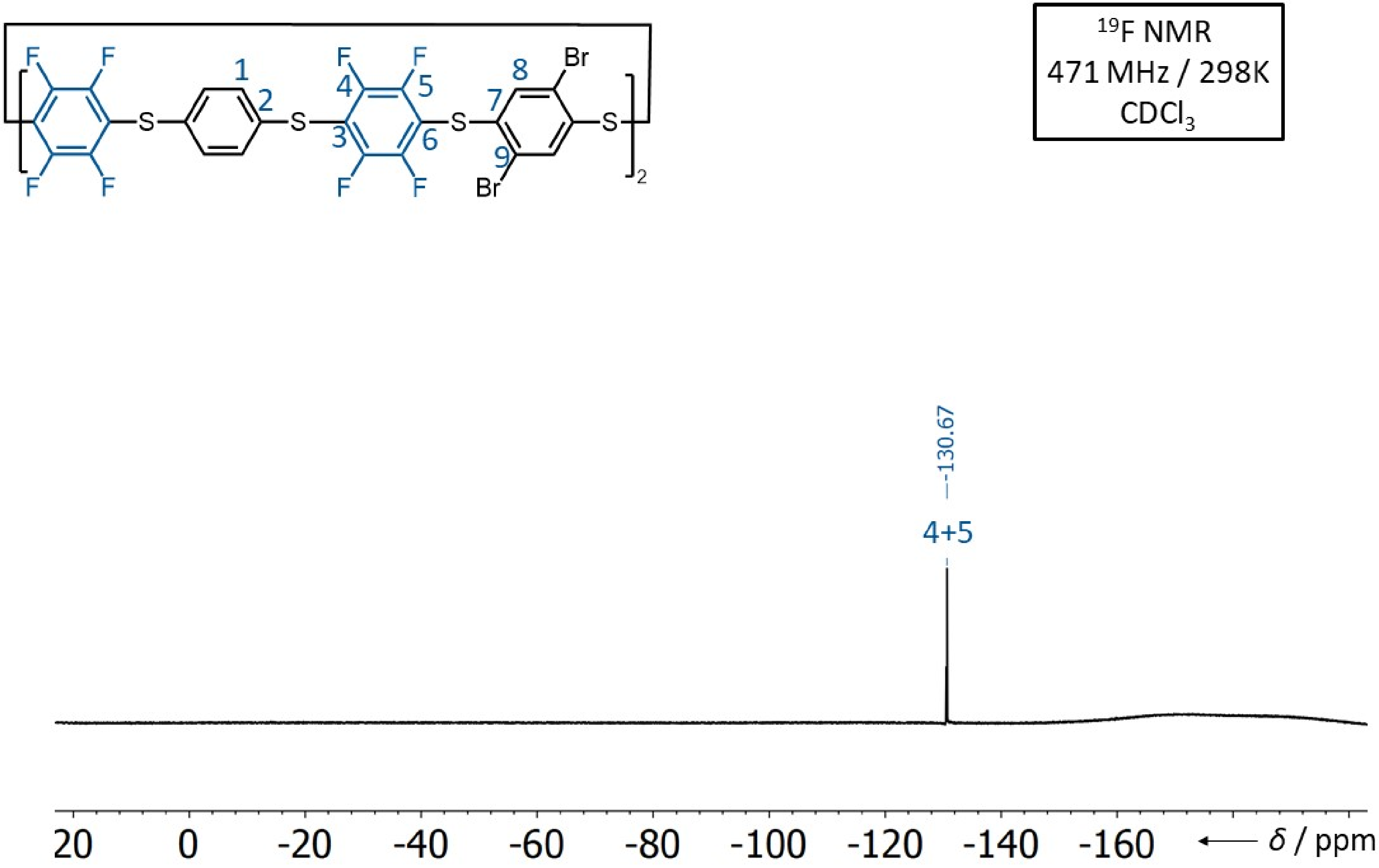

Figure S49. ^{19}F NMR Spectrum of **1ac**.

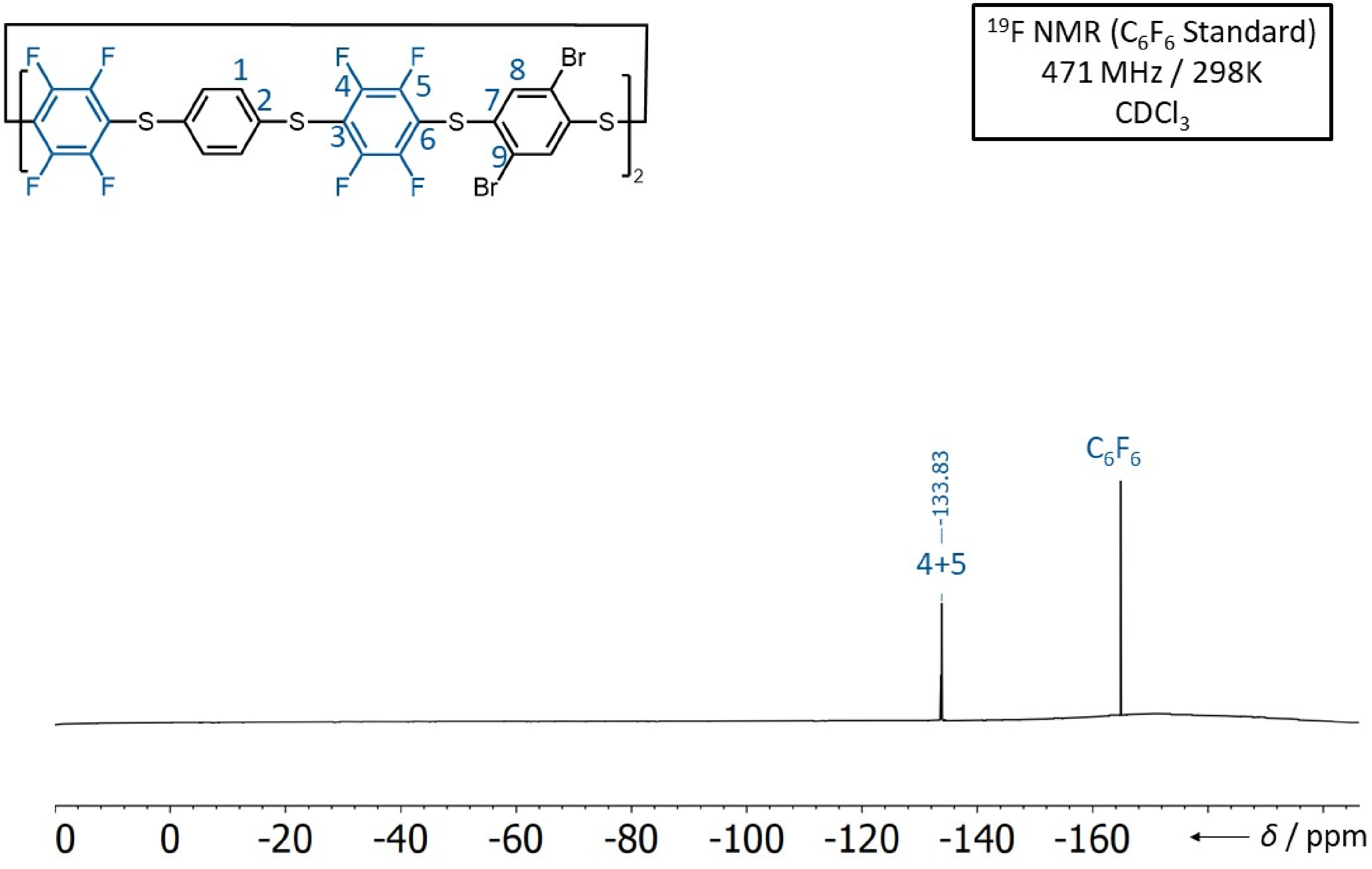

Figure S50. Referenced ^{19}F NMR Spectrum of **1ac**.

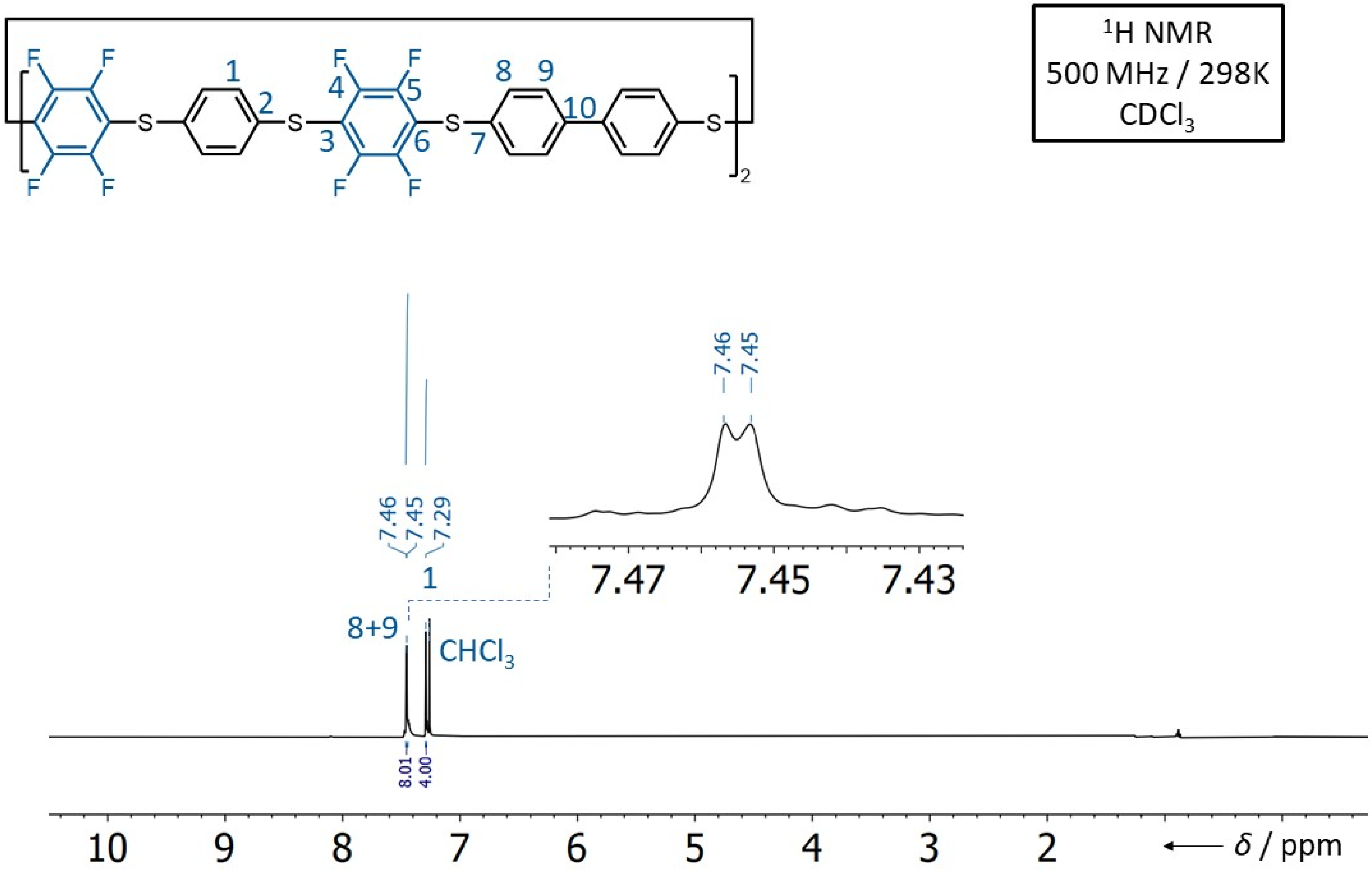

Figure S51. ^{1}H NMR Spectrum of **1ad**.

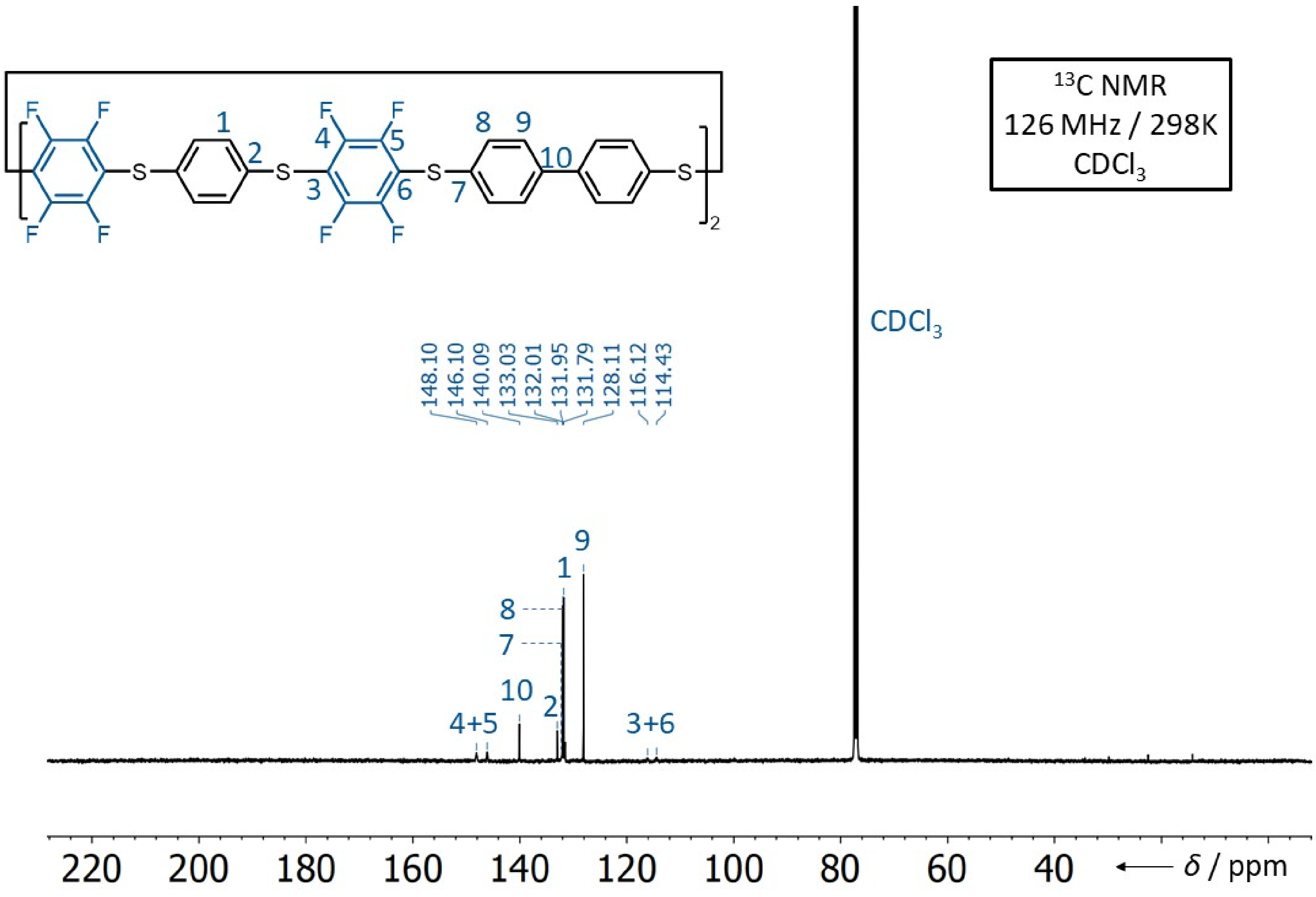

Figure S52. ^{13}C NMR Spectrum of **1ad**.

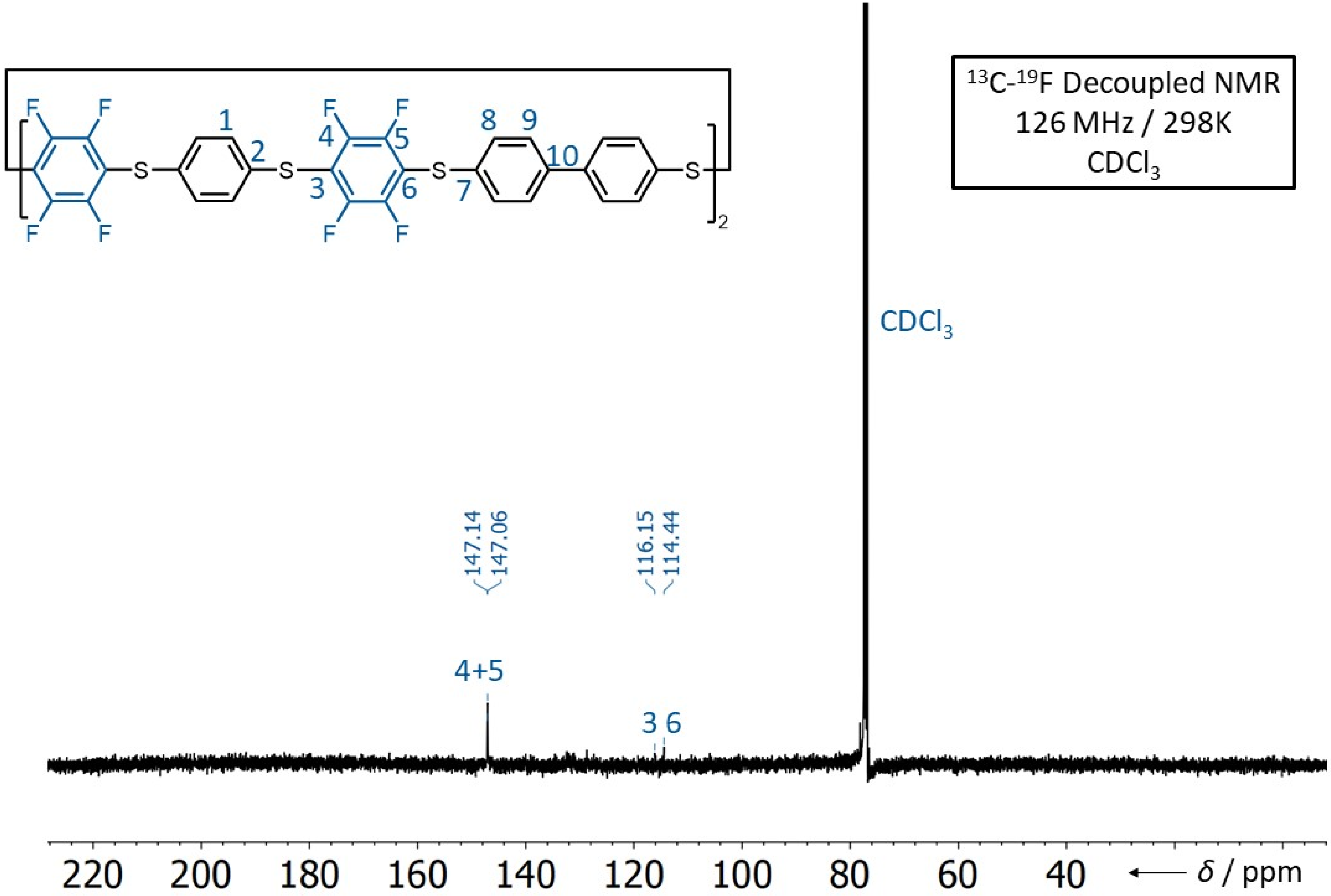

Figure S53. ^{13}C-^{19}F Decoupled NMR Spectrum of **1ad**.

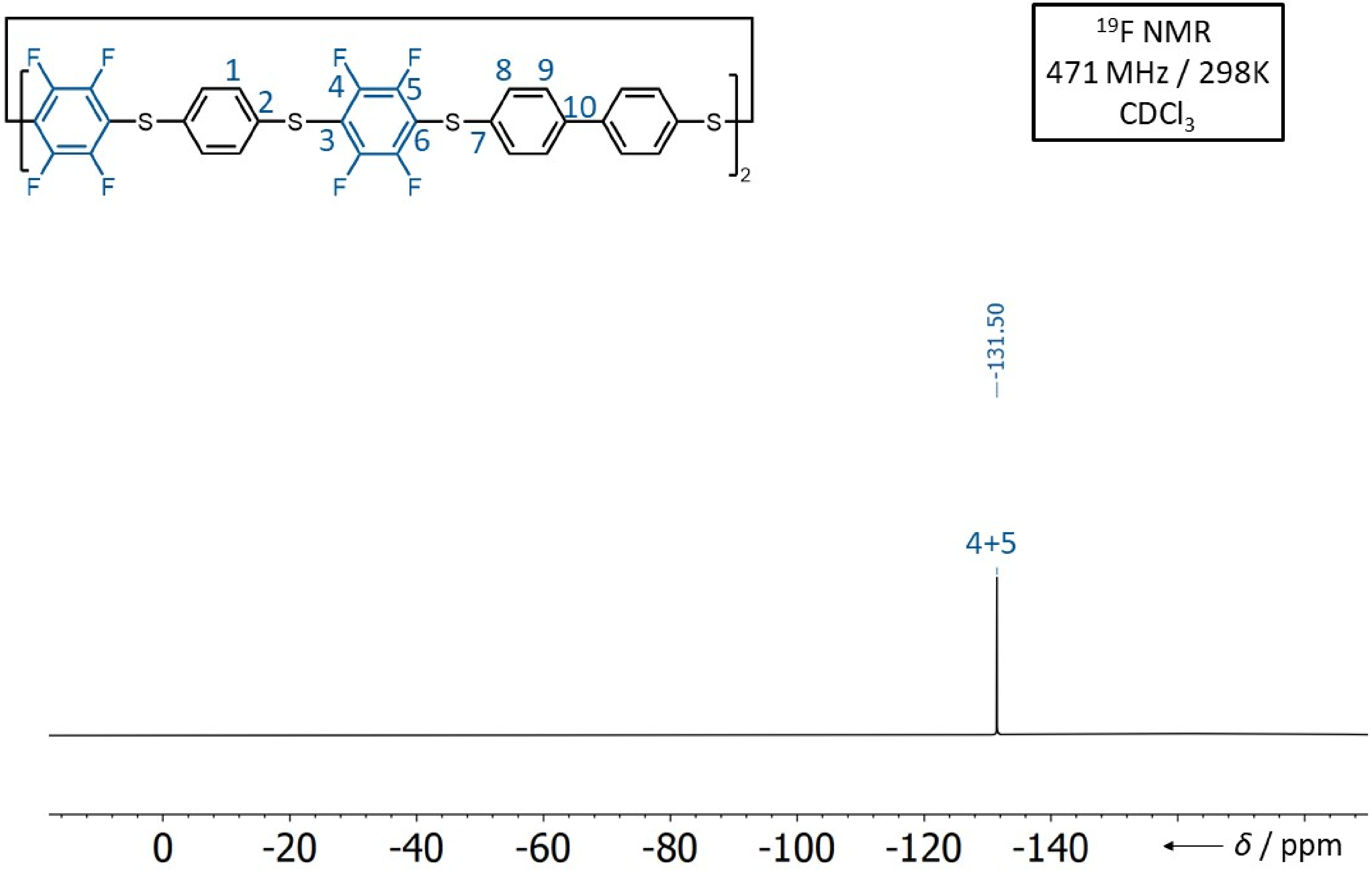

Figure S54. ^{19}F NMR Spectrum of **1ad**.

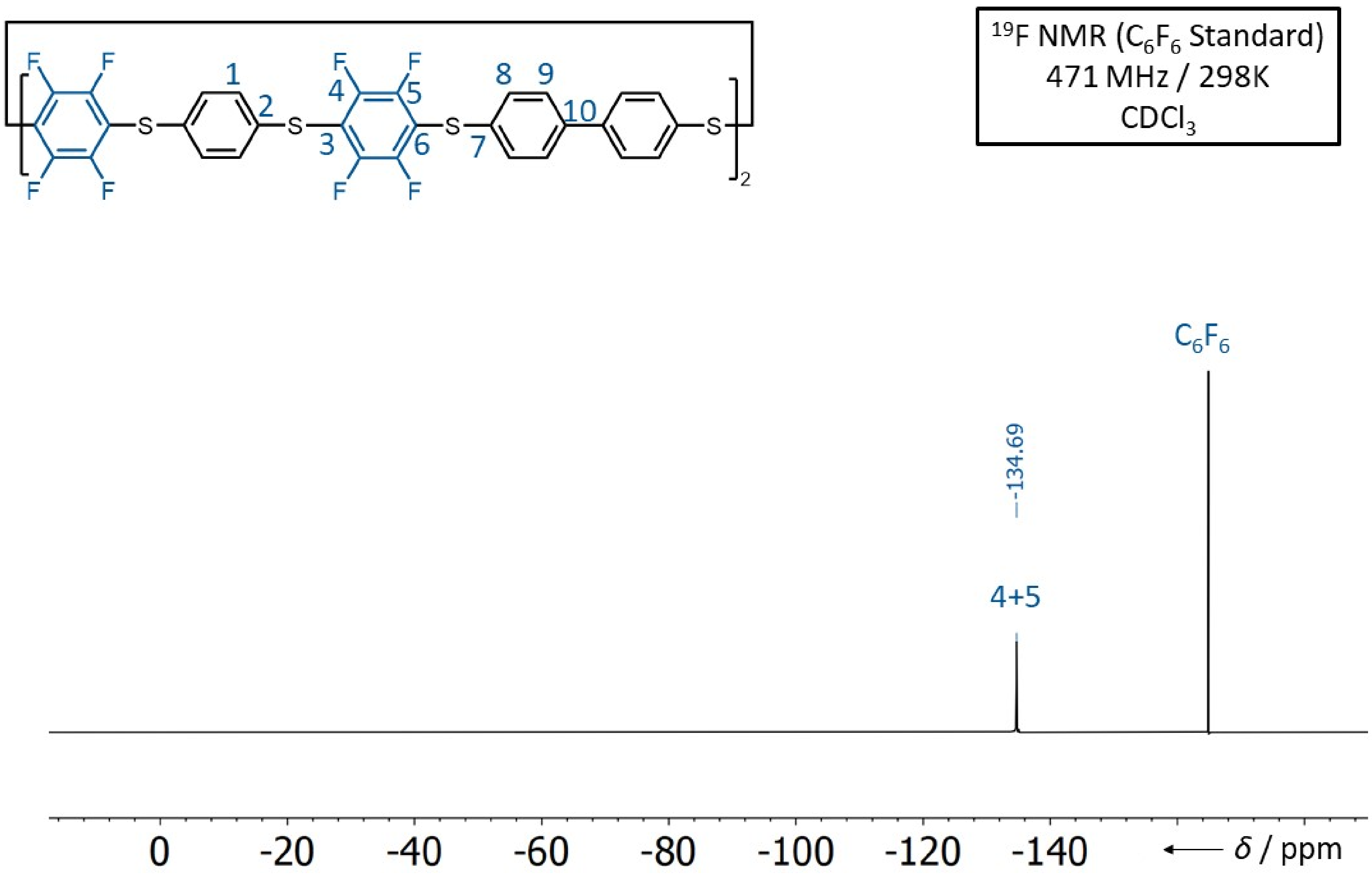

Figure S55. Referenced ^{19}F NMR Spectrum of **1ad**.

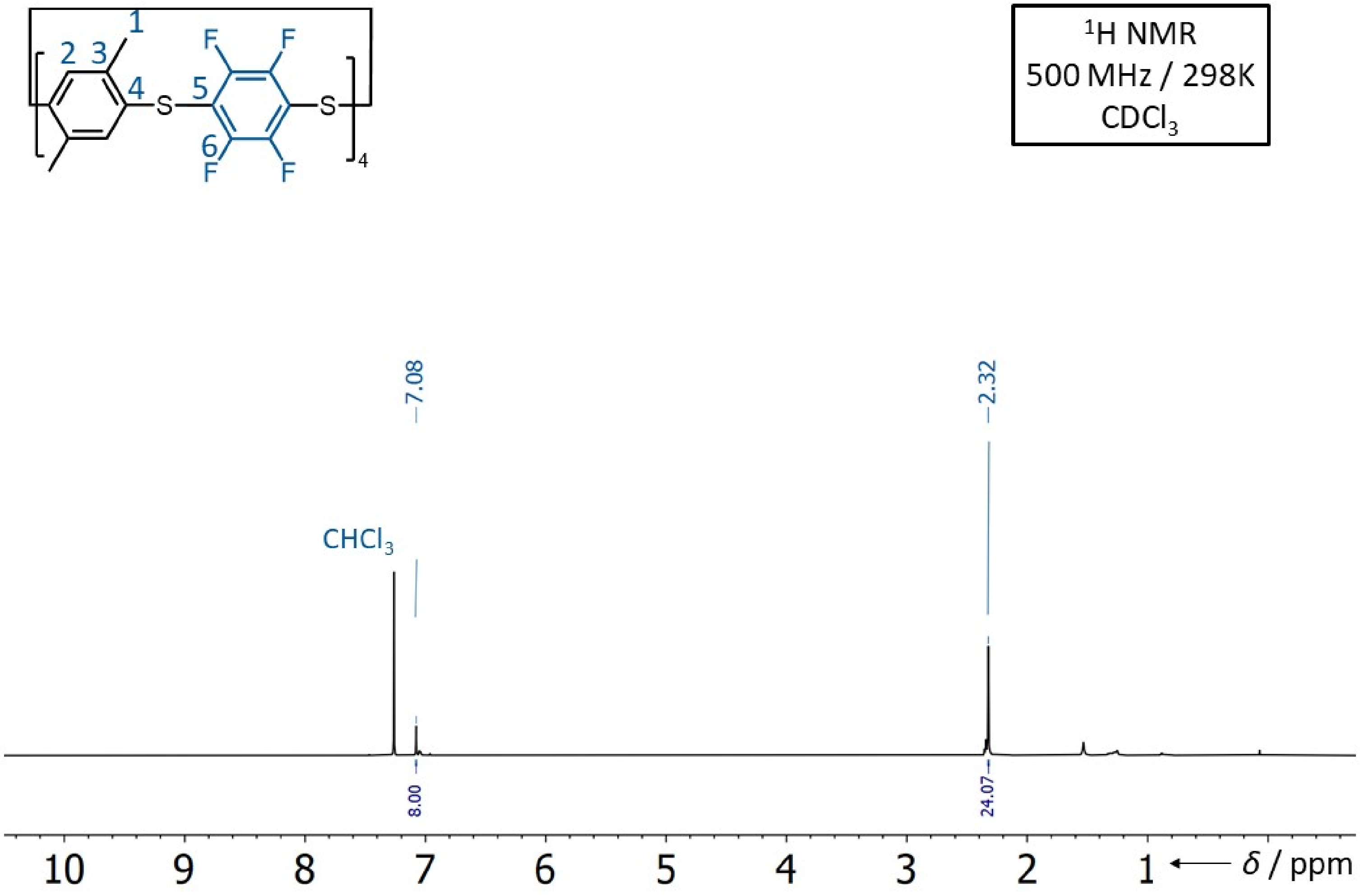

Figure S56. ^{1}H NMR Spectrum of **1b**.

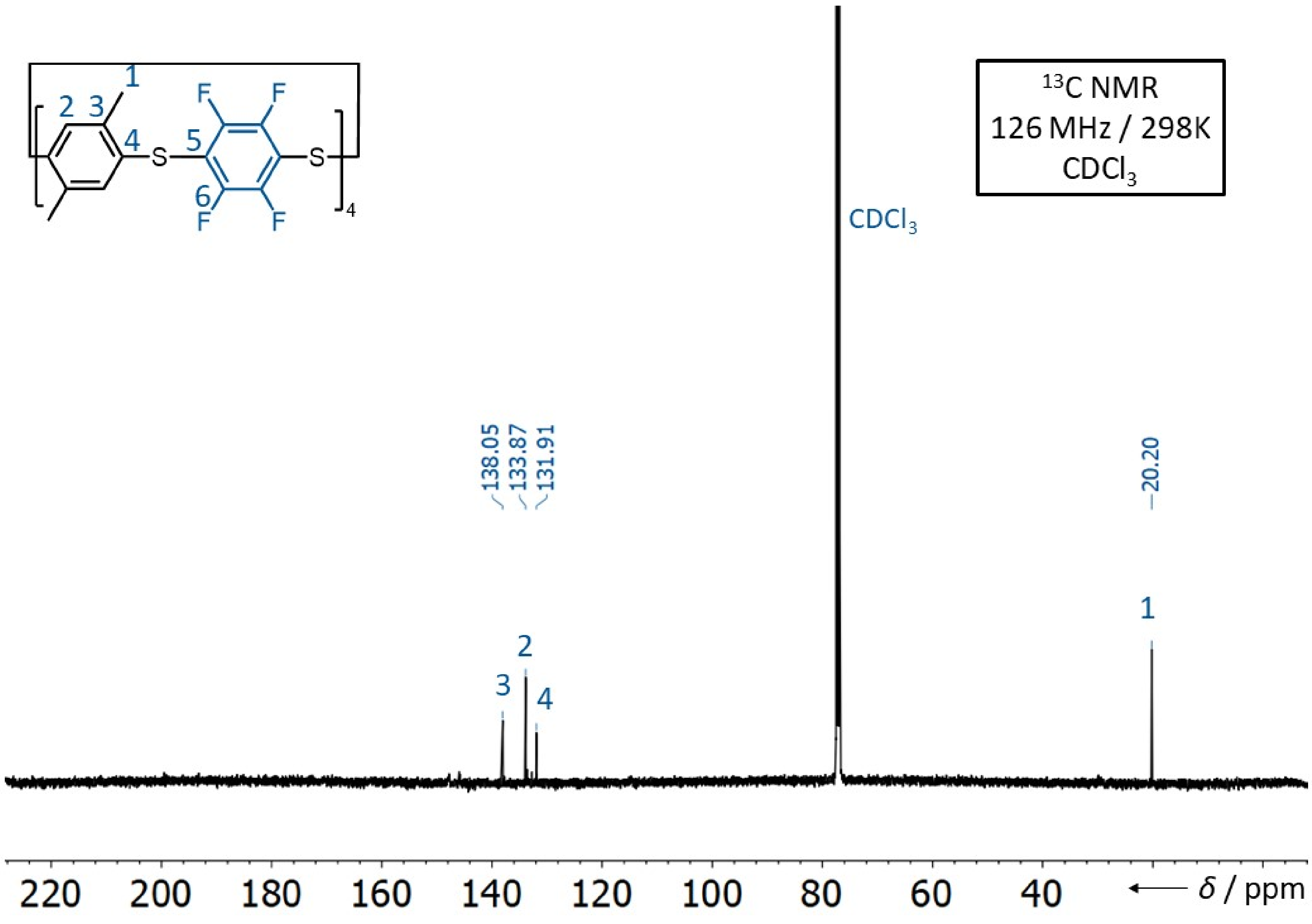

Figure S57. ^{13}C NMR Spectrum of **1b**.

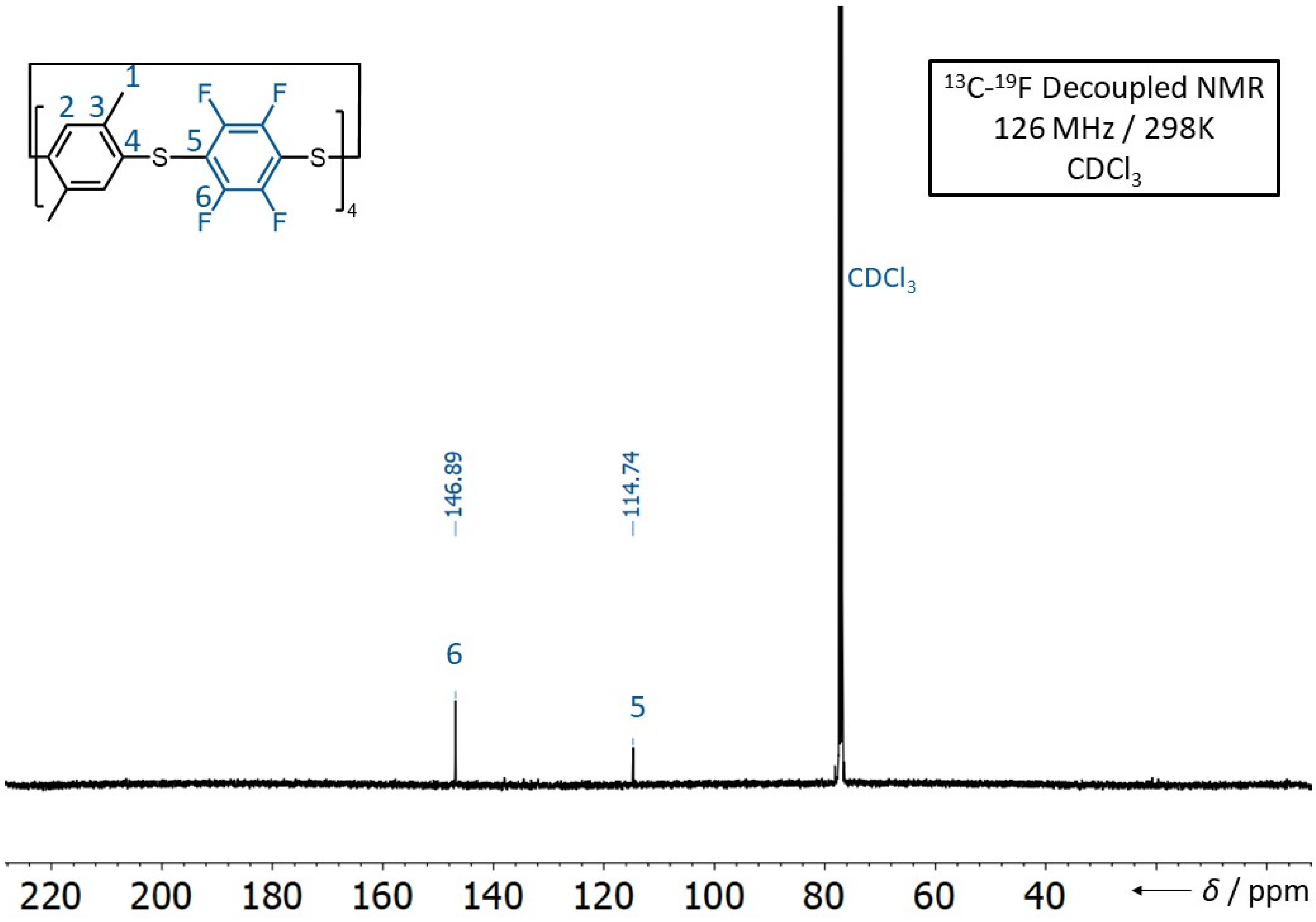

Figure S58. ^{13}C-^{19}F Decoupled NMR Spectrum of **1b**.

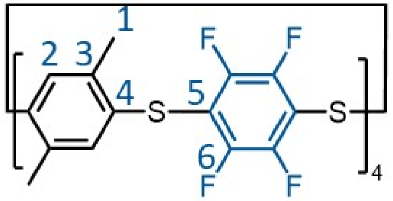

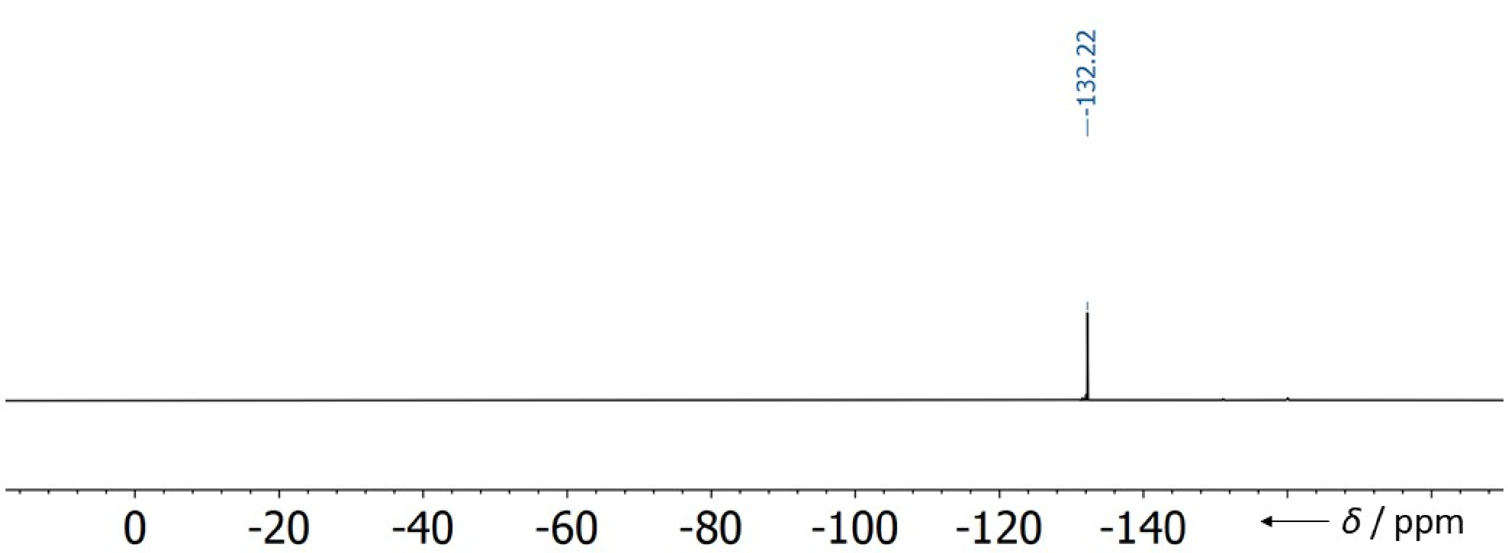

Figure S59. ^{19}F NMR Spectrum of **1b**.

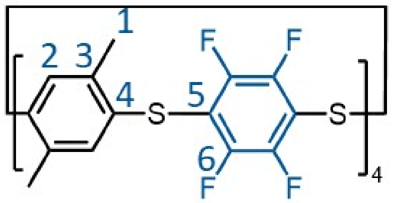

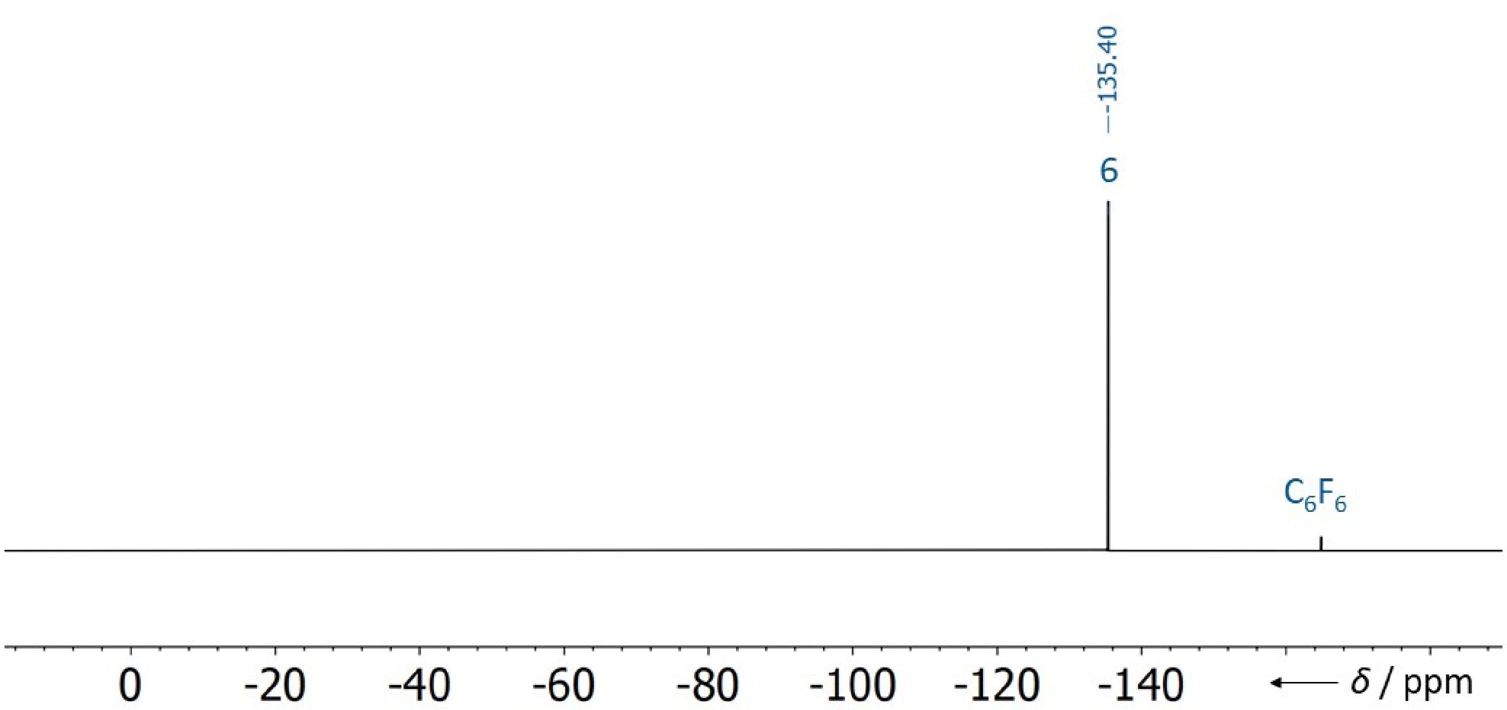

Figure S60. Referenced ^{19}F NMR Spectrum of **1b**.

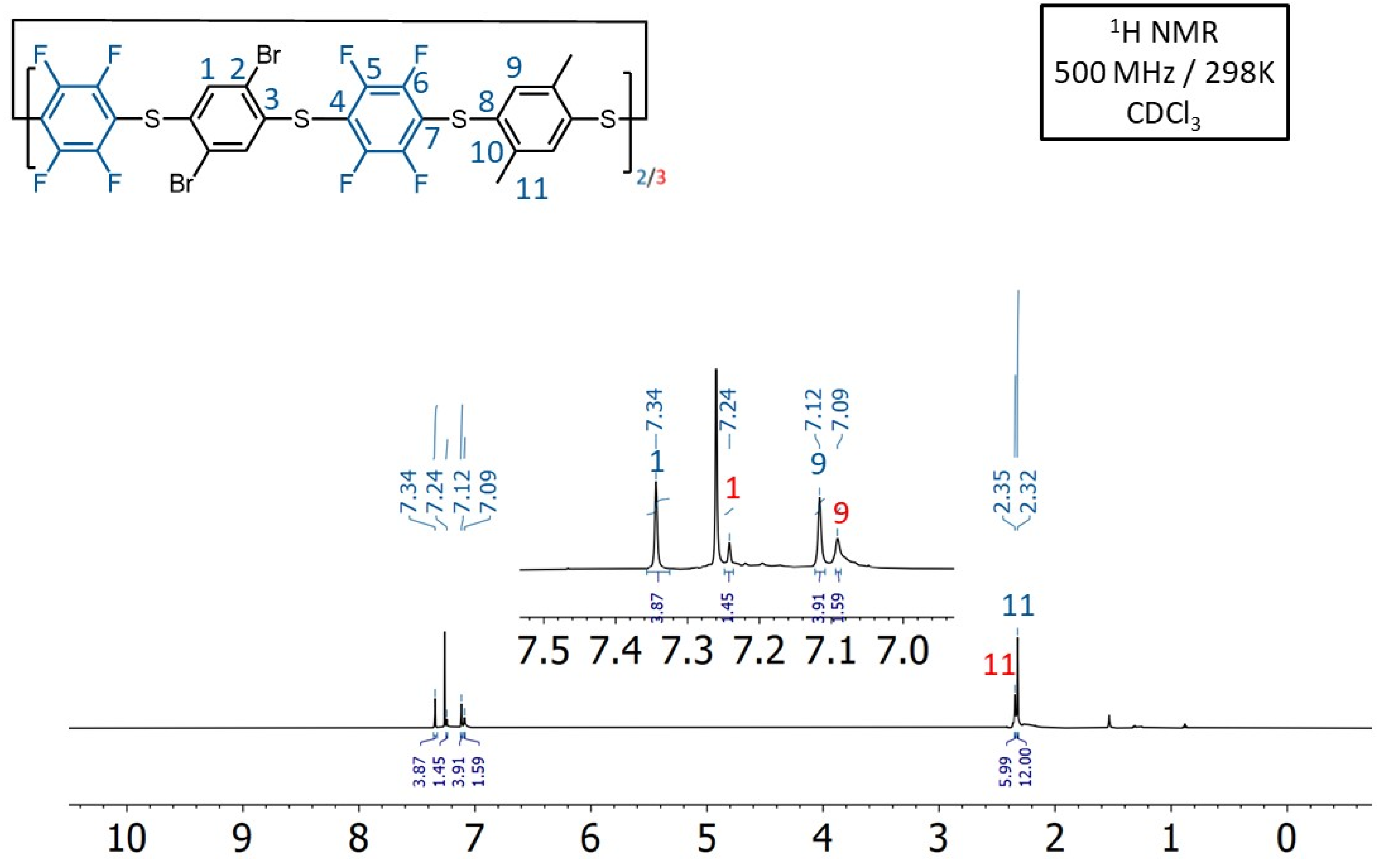

Figure S61. ^{1}H NMR Spectrum of **1bc**.

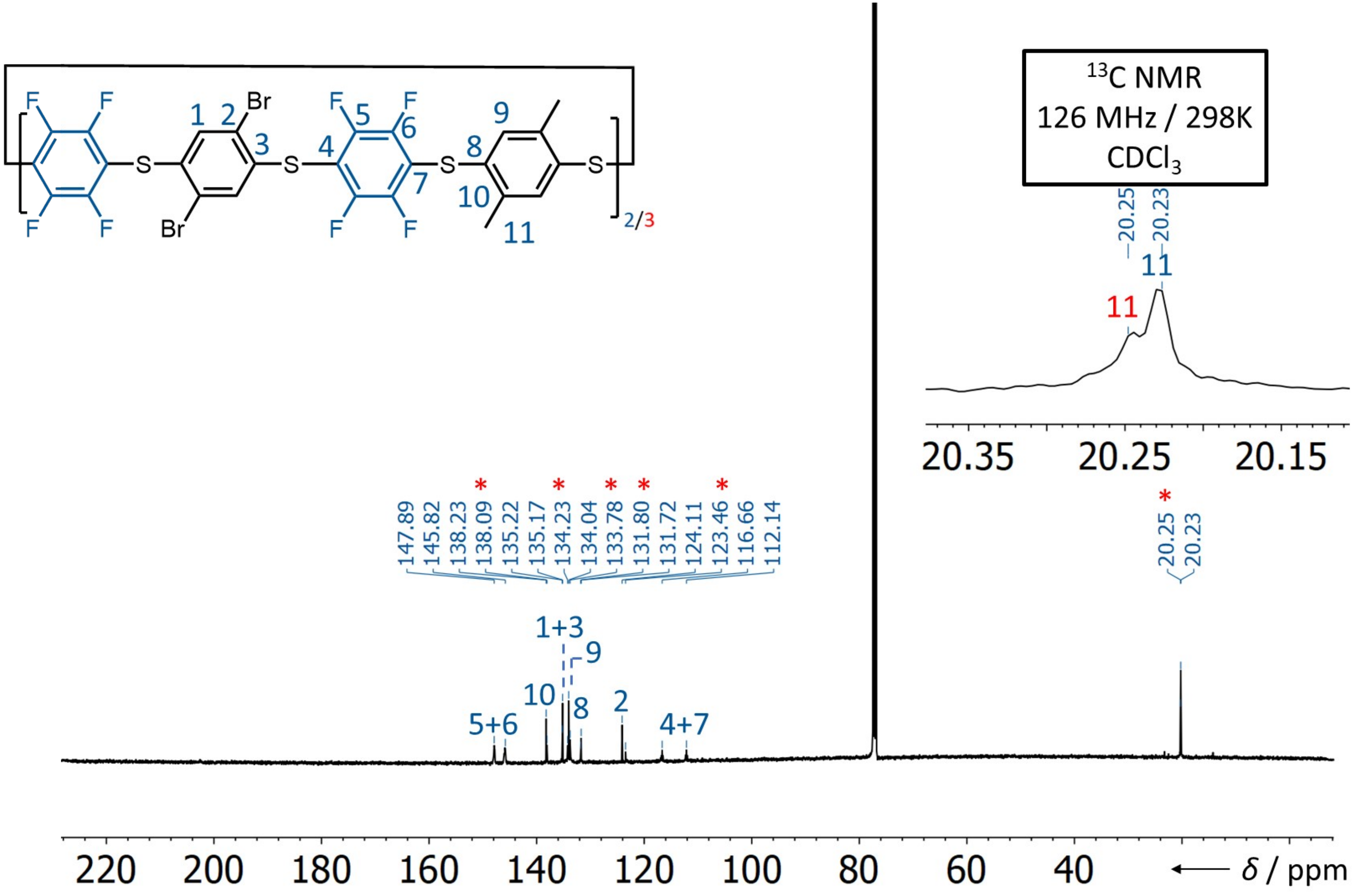

Figure S62. ^{13}C NMR Spectrum of **1bc**.

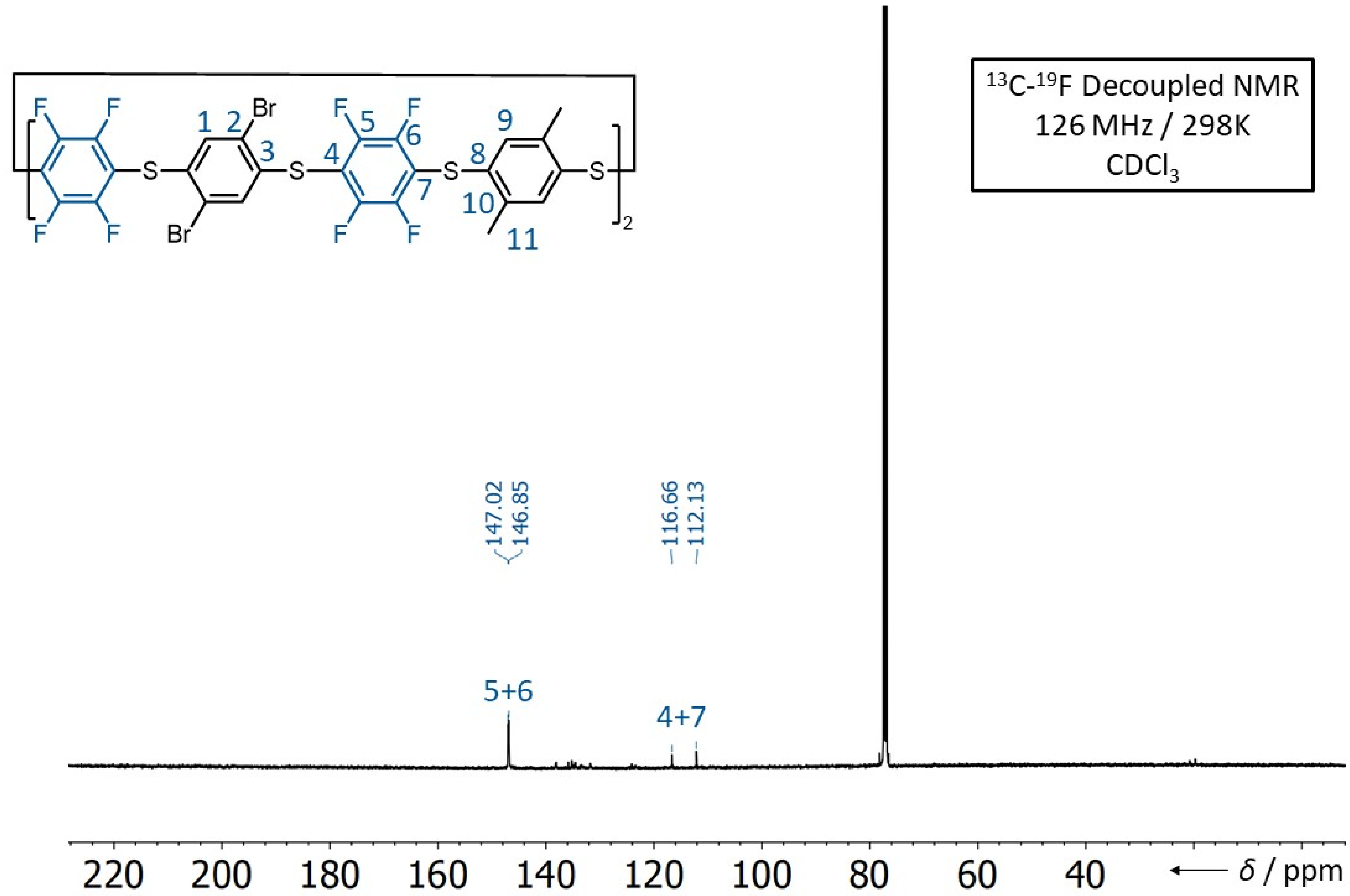

Figure S63. ^{13}C-^{19}F Decoupled NMR Spectrum of **1bc**.

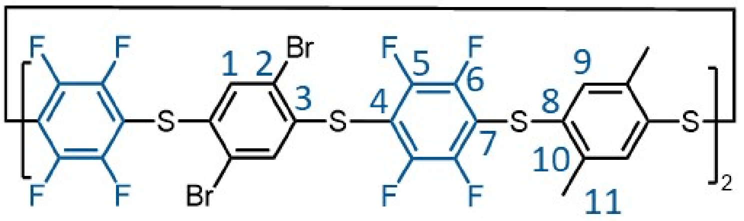

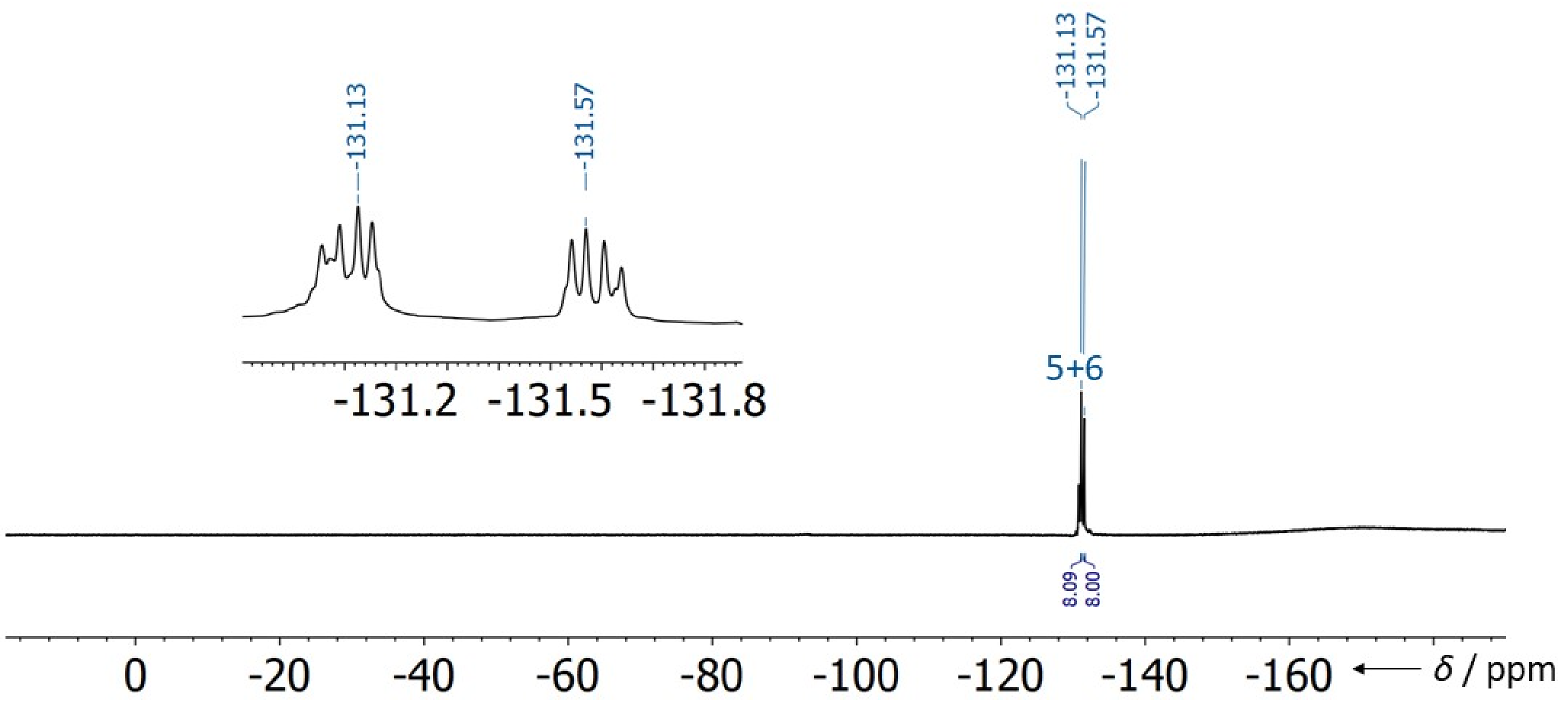

Figure S64. ^{19}F NMR Spectrum of **1bc**.

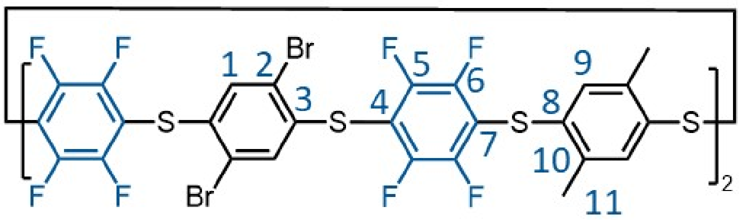

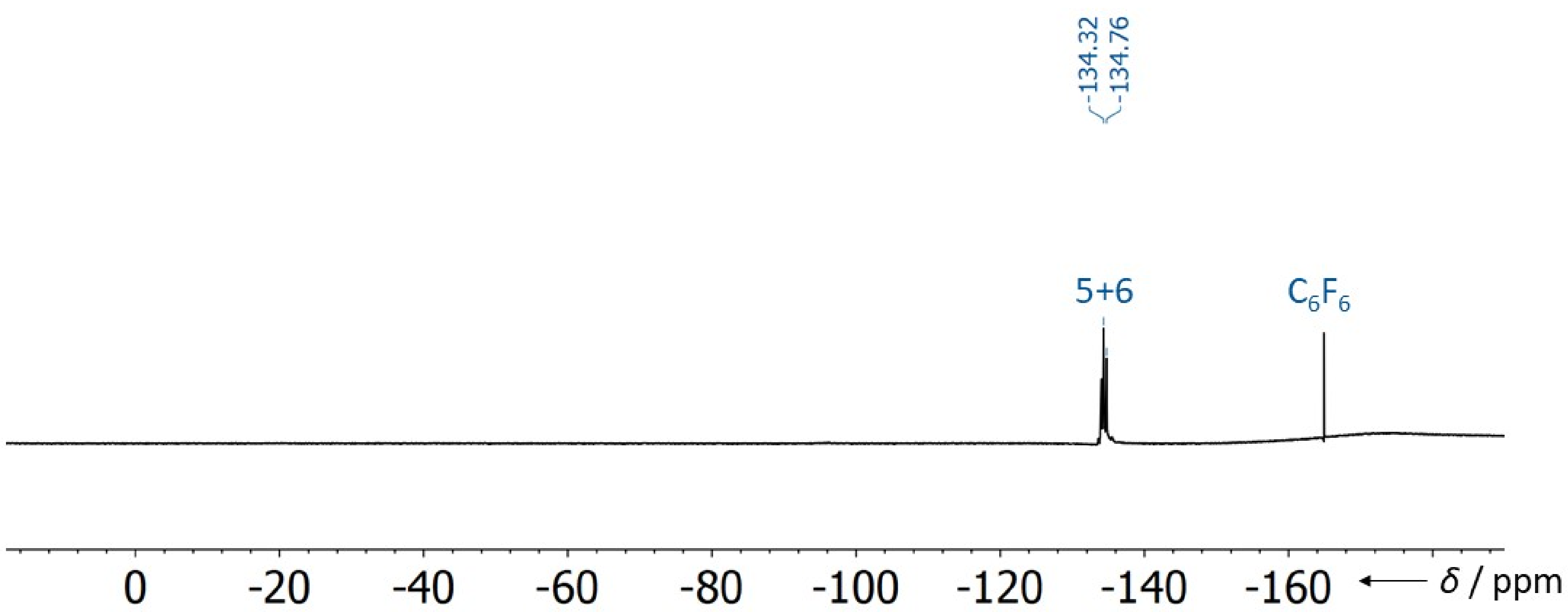

Figure S65. Referenced ^{19}F NMR Spectrum of **1bc**.

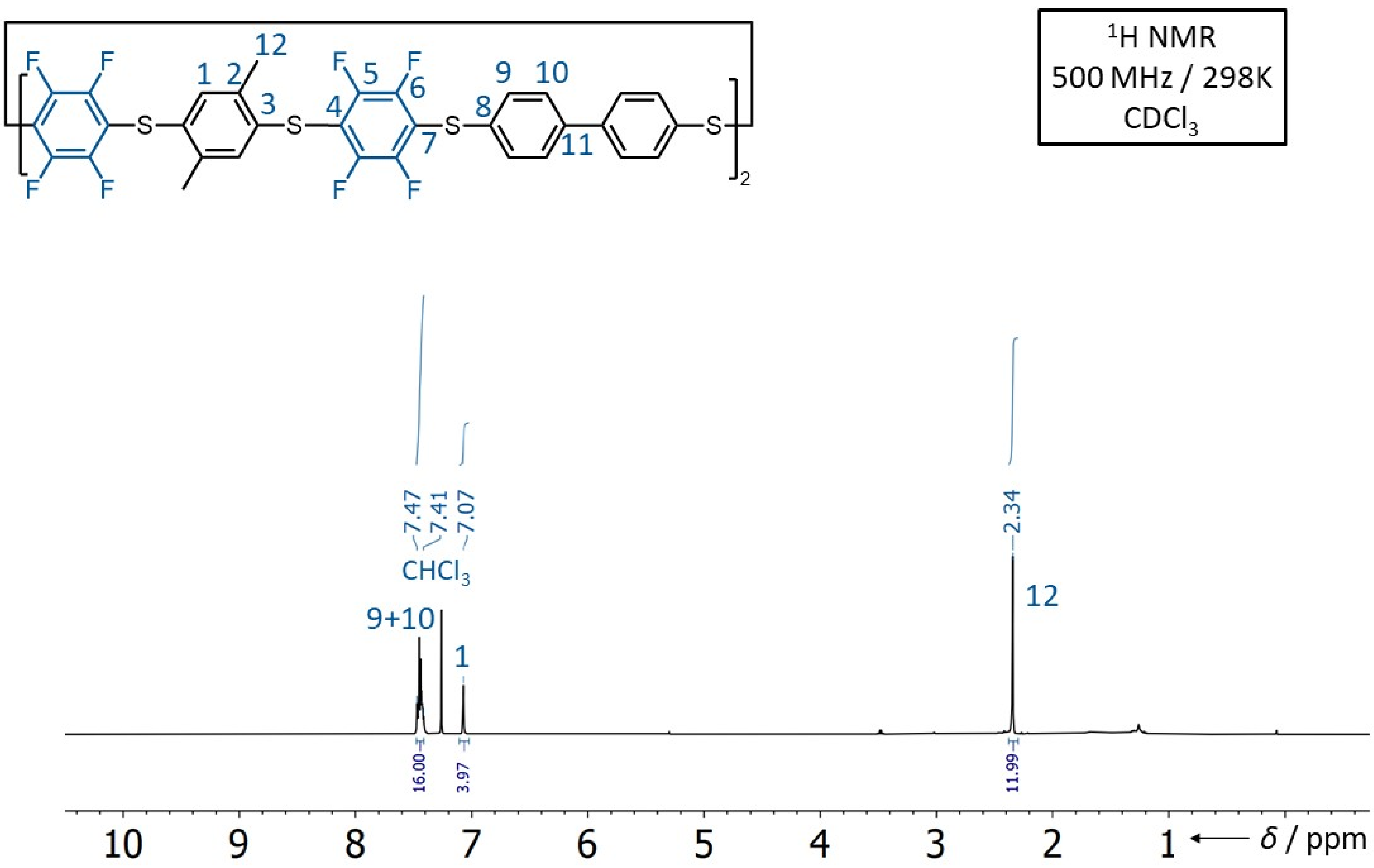

Figure S66. ^{1}H NMR Spectrum of **1bd**.

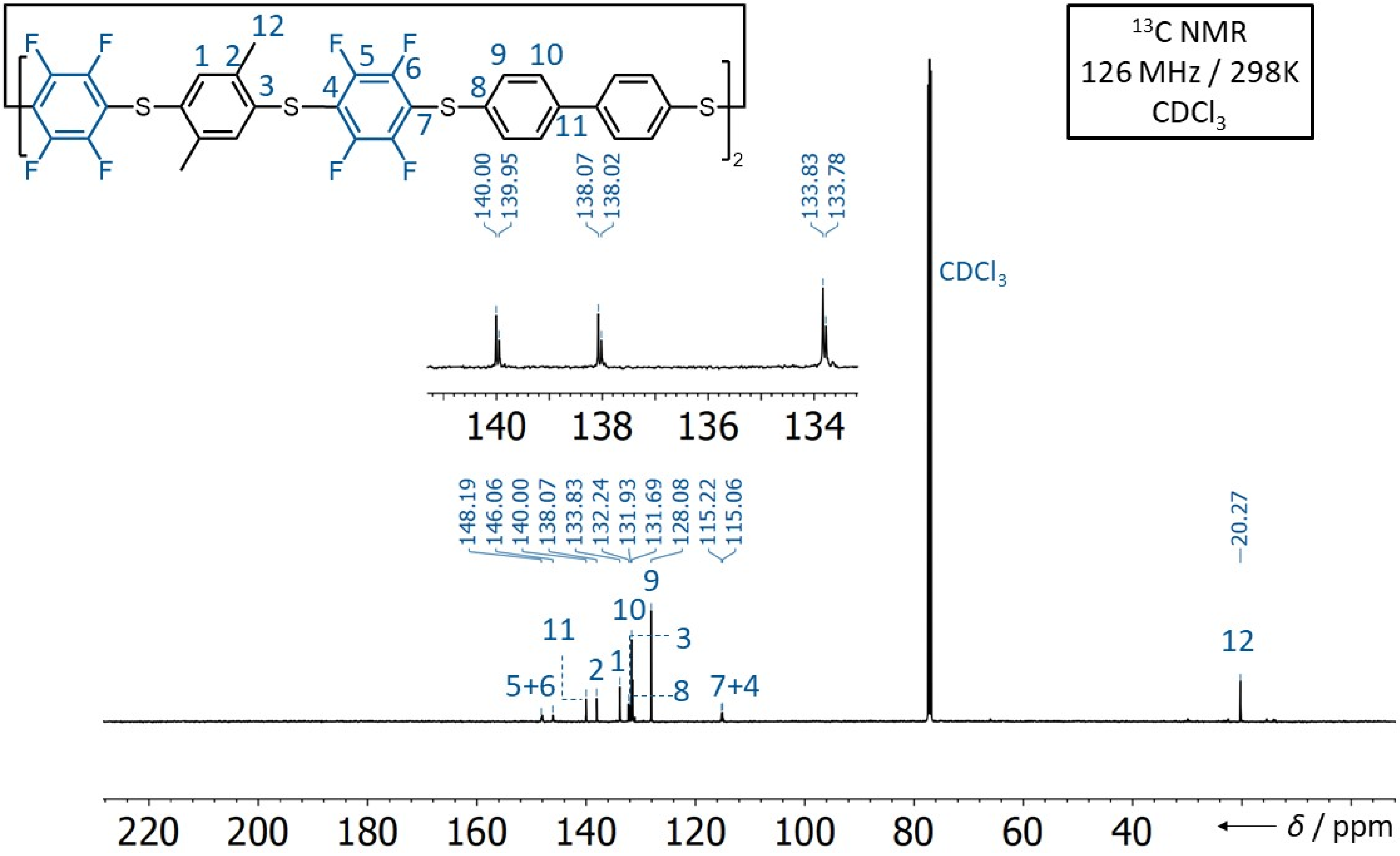

Figure S67. ^{13}C NMR Spectrum of **1bd**.

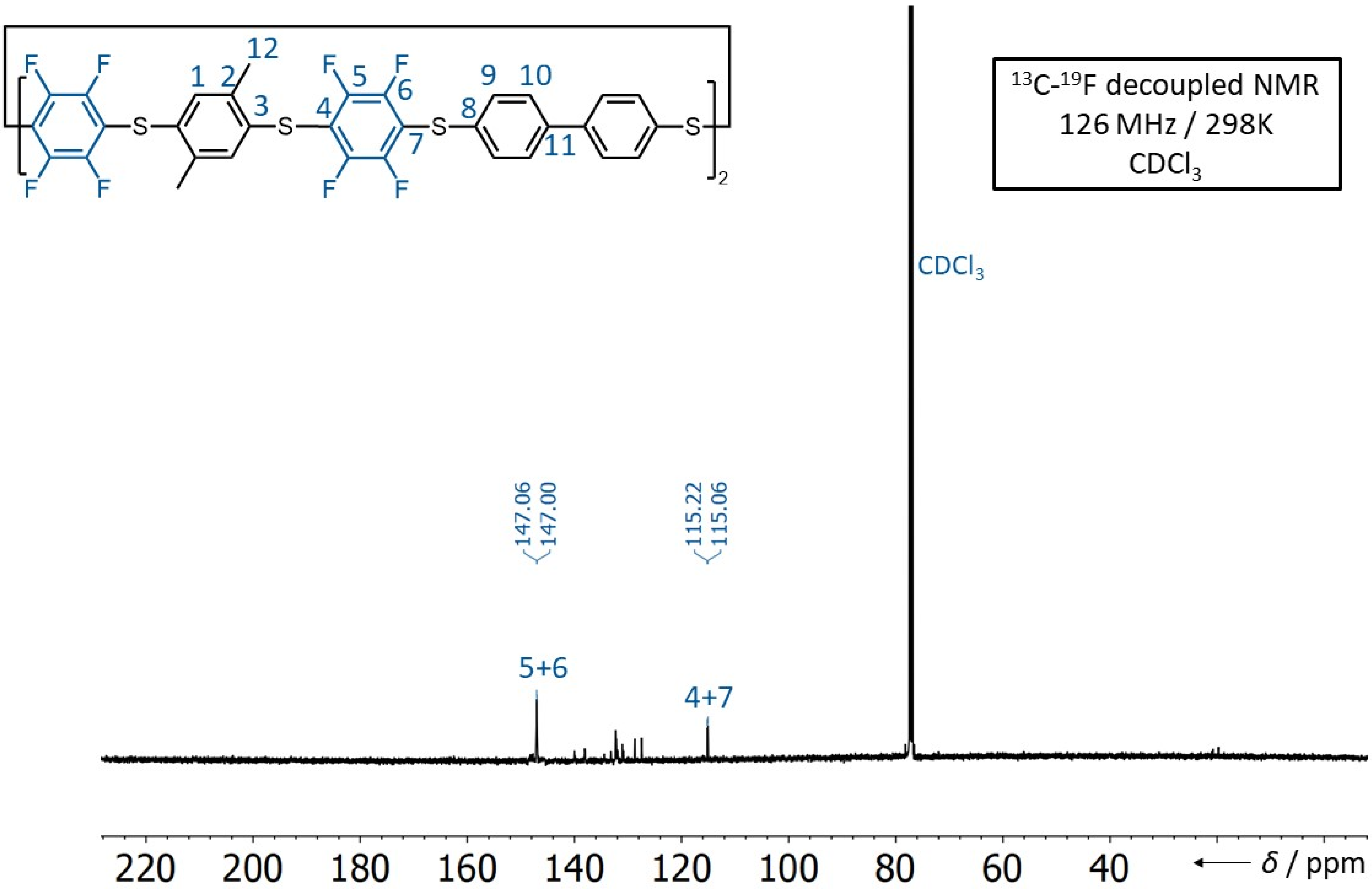

Figure S68. ^{13}C-^{19}F Decoupled NMR Spectrum of **1bd**.

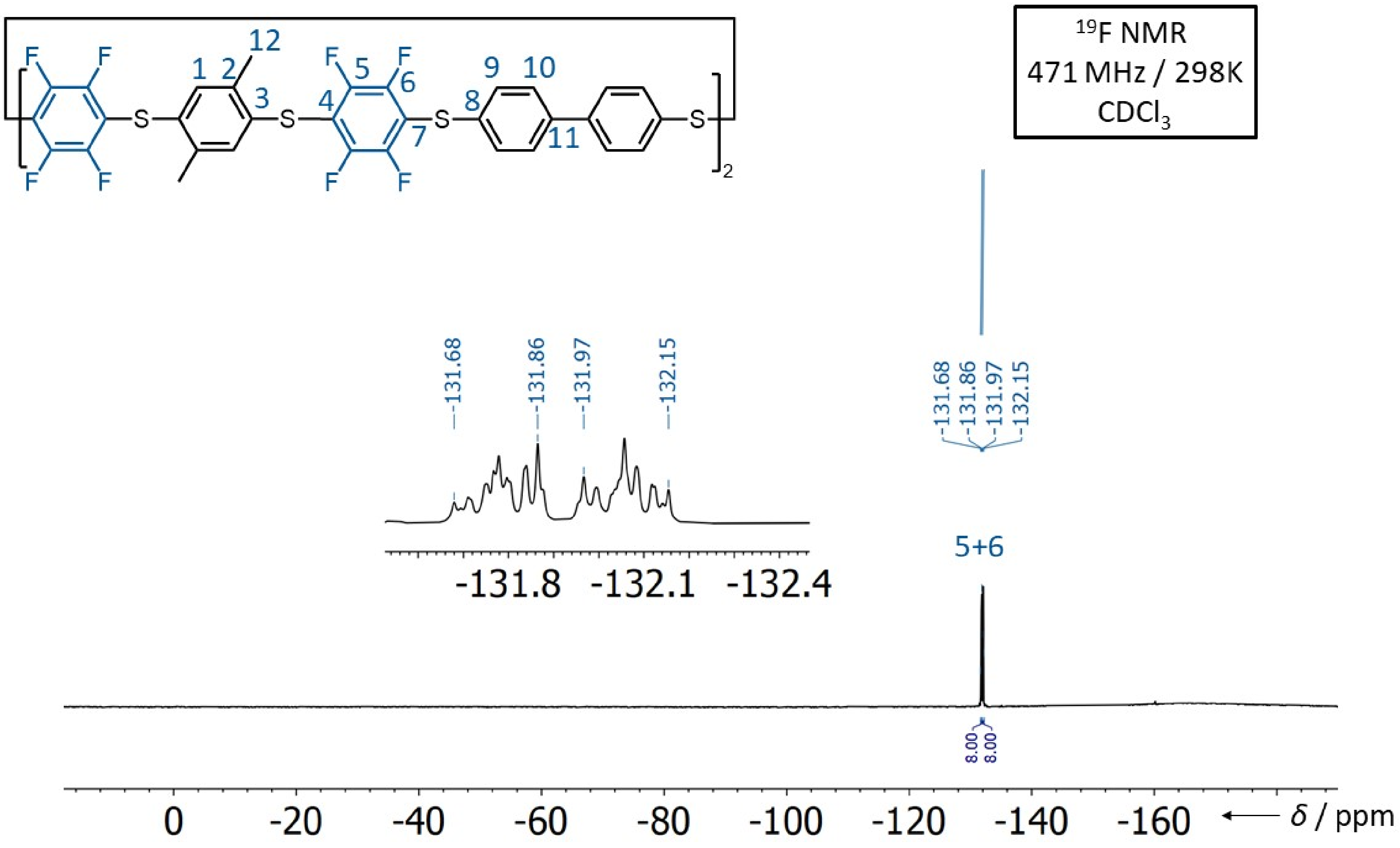

Figure S69. ^{19}F NMR Spectrum of **1bd**.

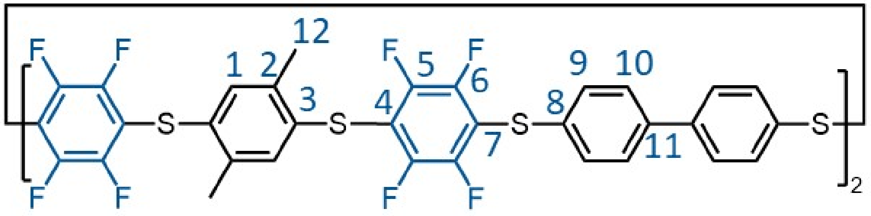

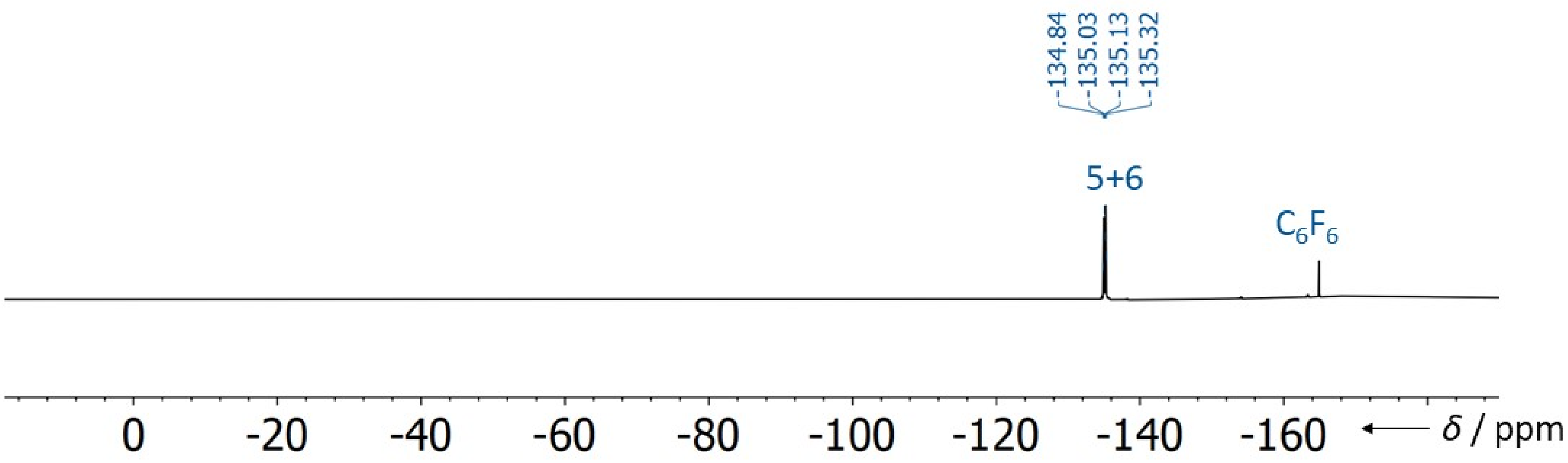

Figure S70. Referenced ^{19}F NMR Spectrum of **1bd**.

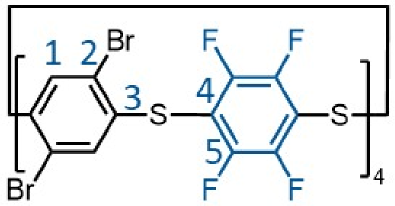

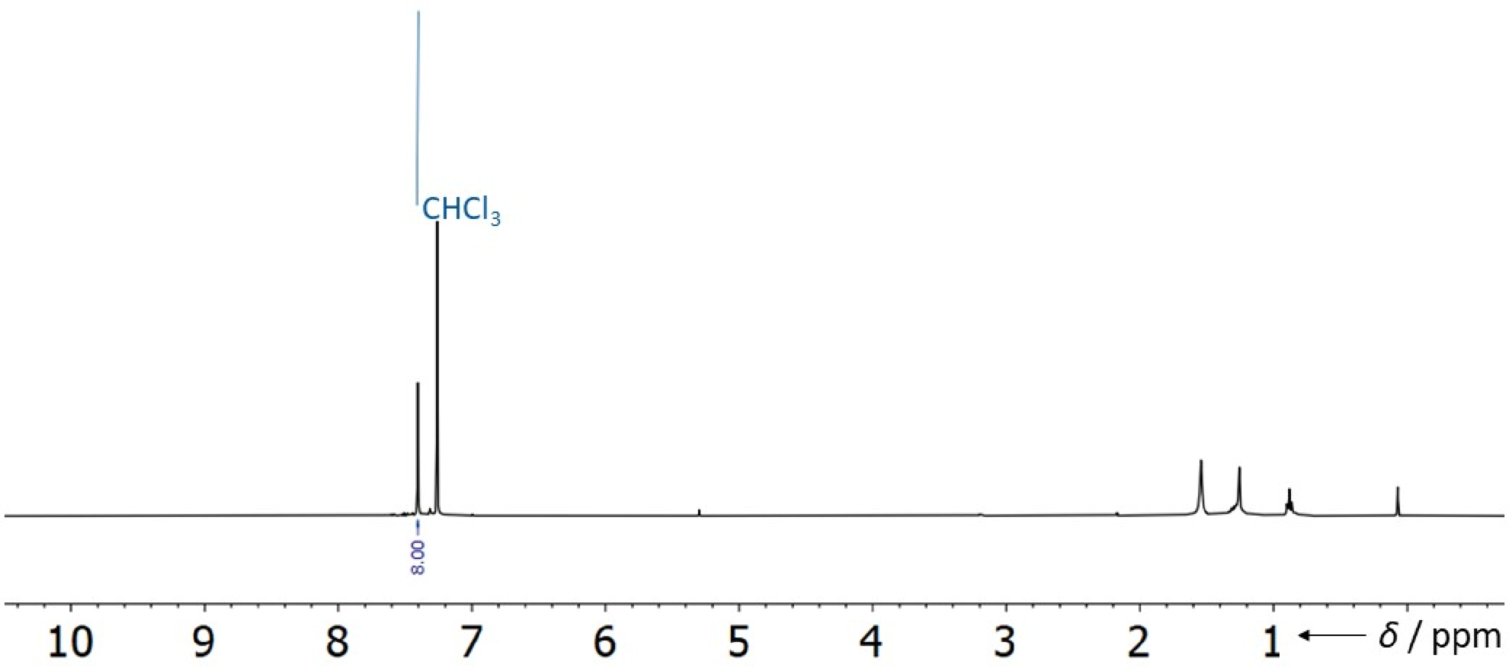

Figure S71. ^{1}H NMR Spectrum of **1c**.

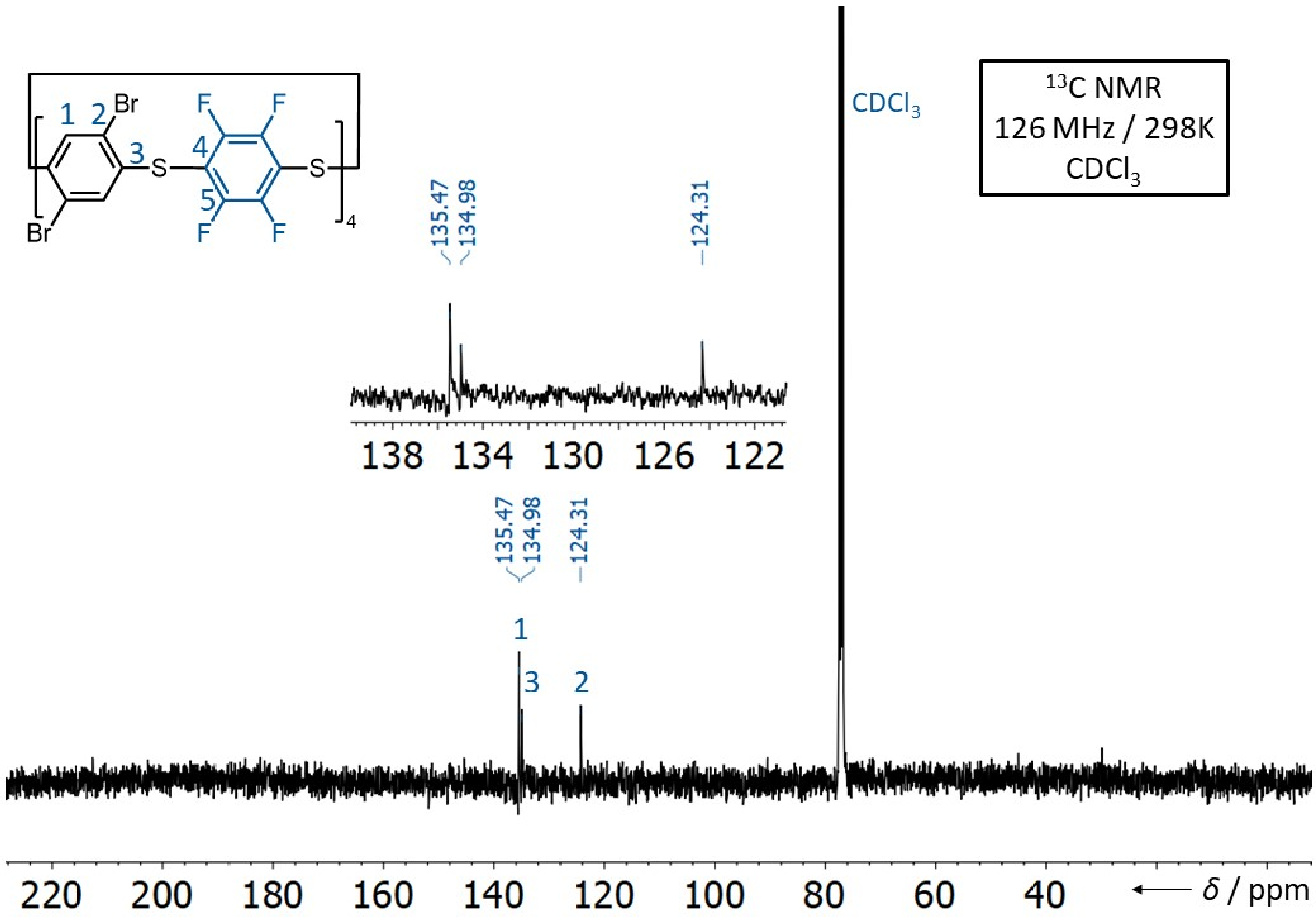

Figure S72. ^{13}C NMR Spectrum of **1c**.

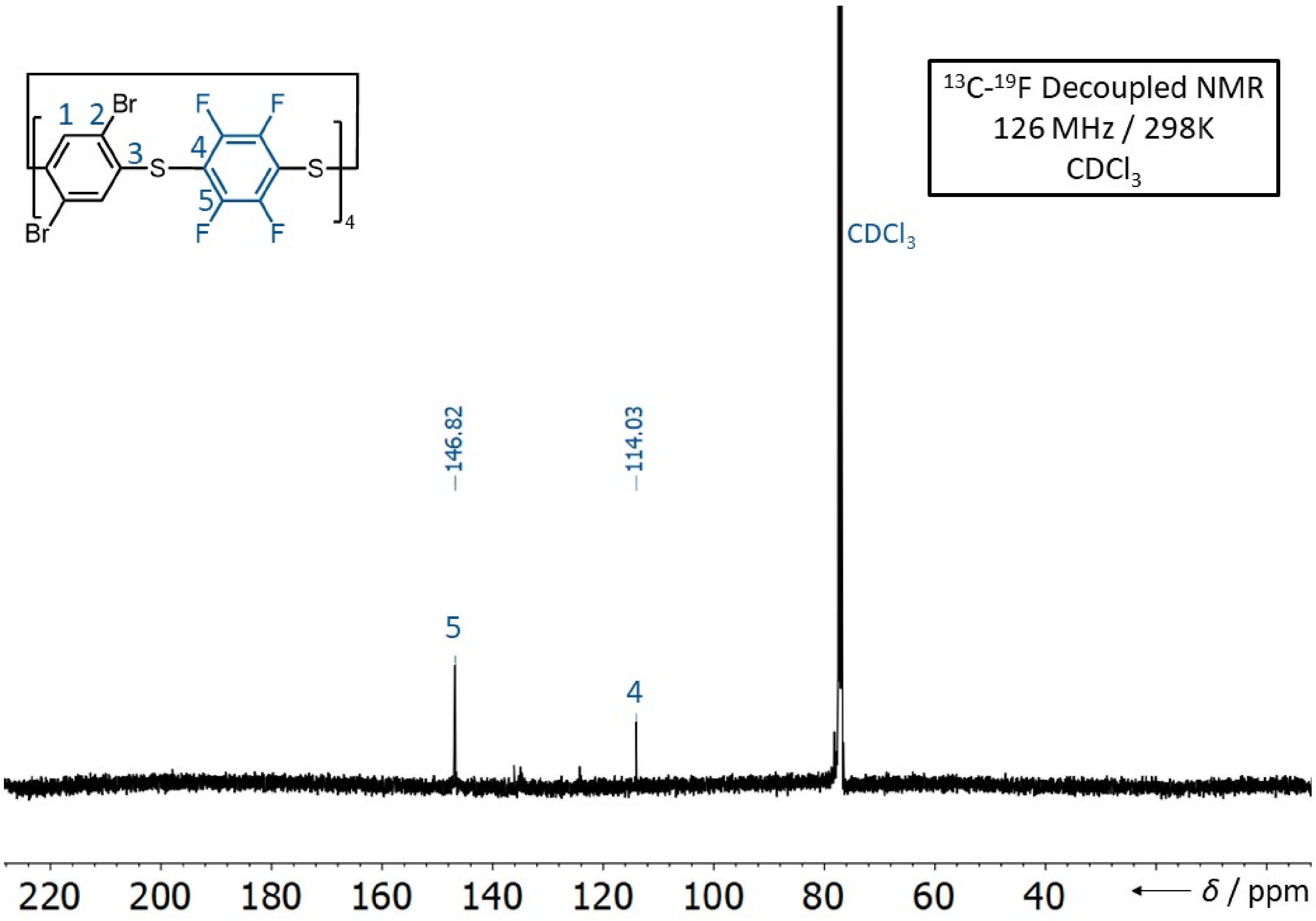

Figure S73. ^{13}C-^{19}F Decoupled NMR Spectrum of **1c**.

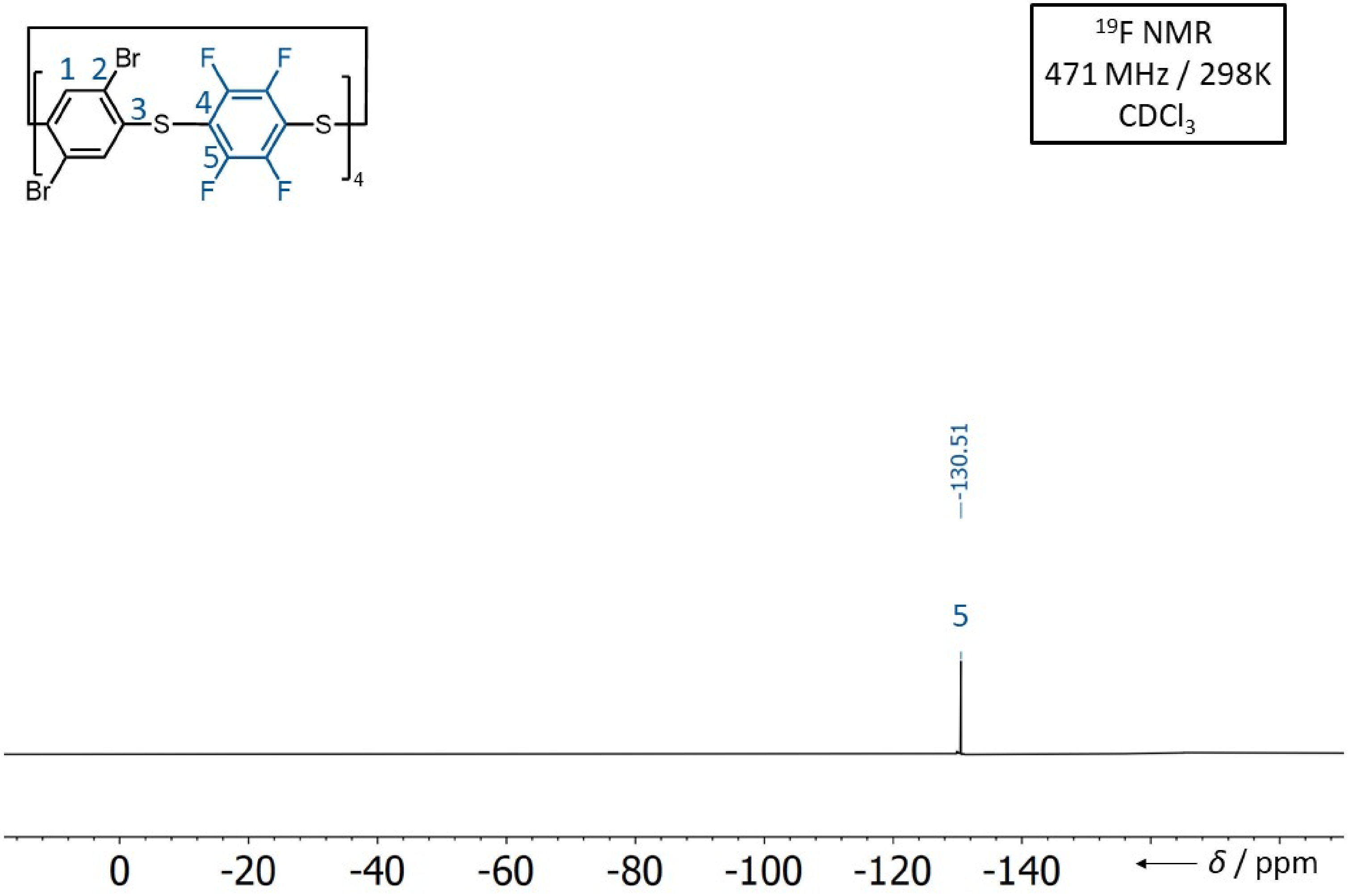

Figure S74. ^{19}F NMR Spectrum of **1c**.

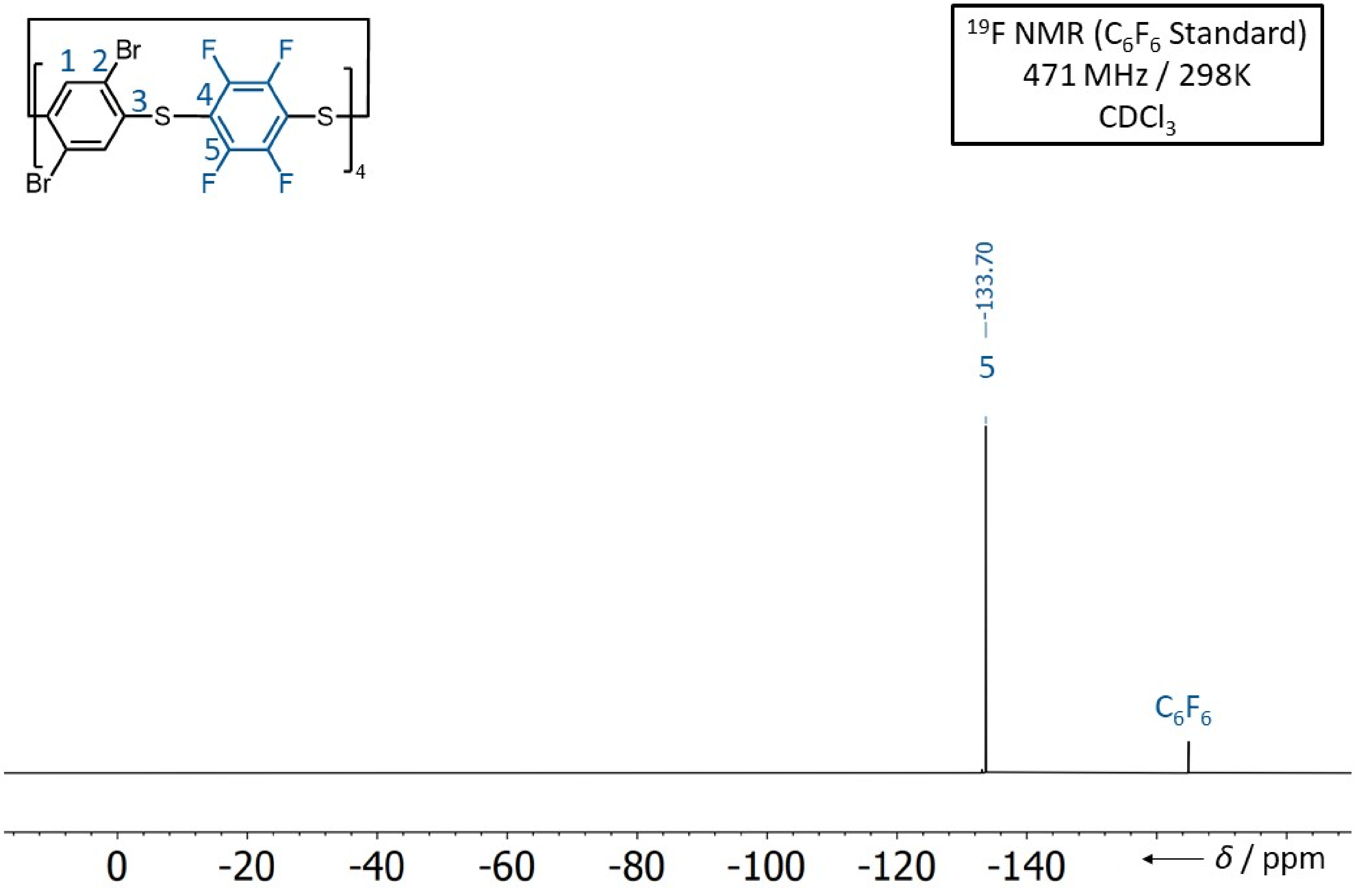

Figure S75. ^{19}F NMR Spectrum of **1c**.

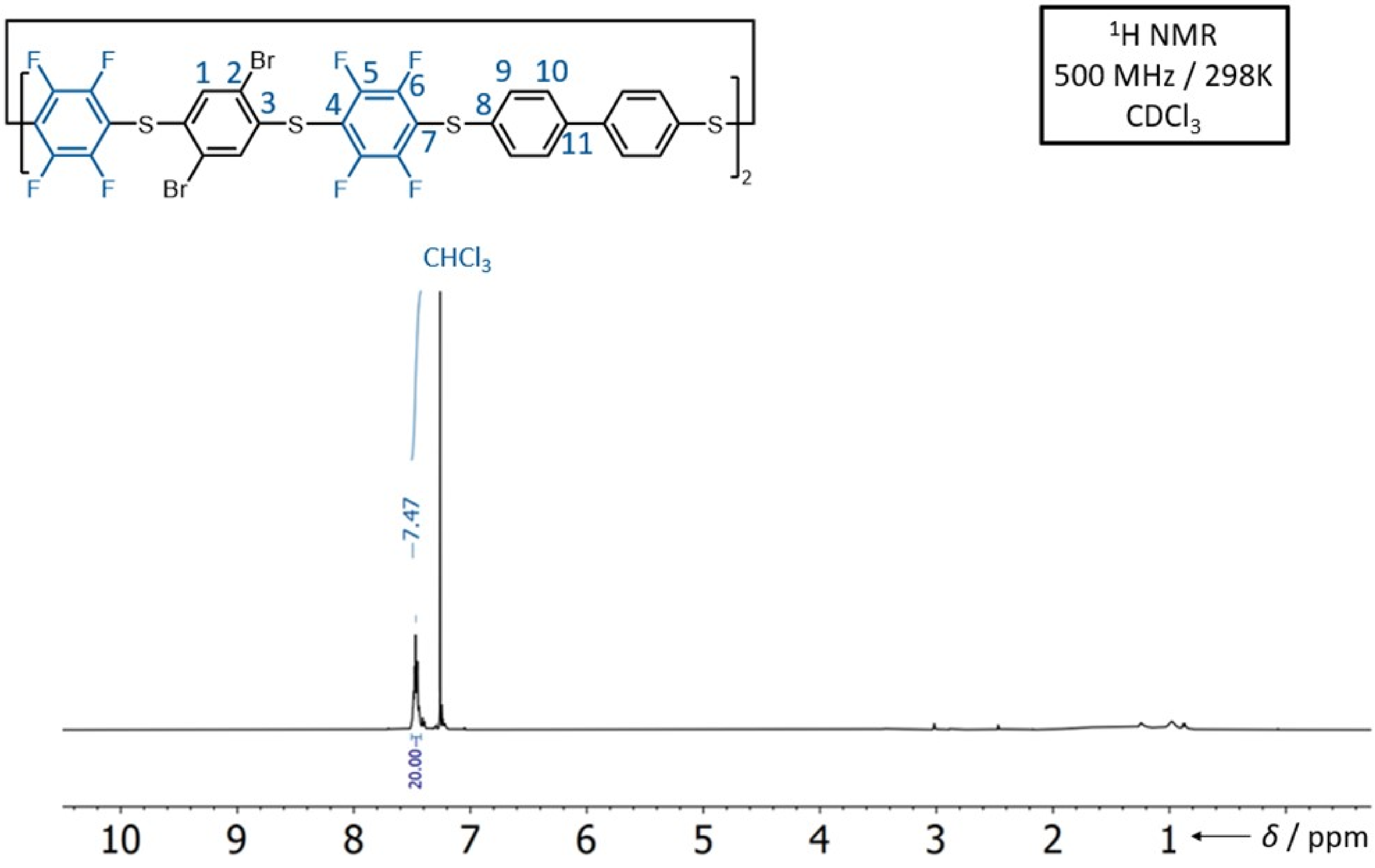

Figure S76. ^{1}H NMR Spectrum of **1cd**.

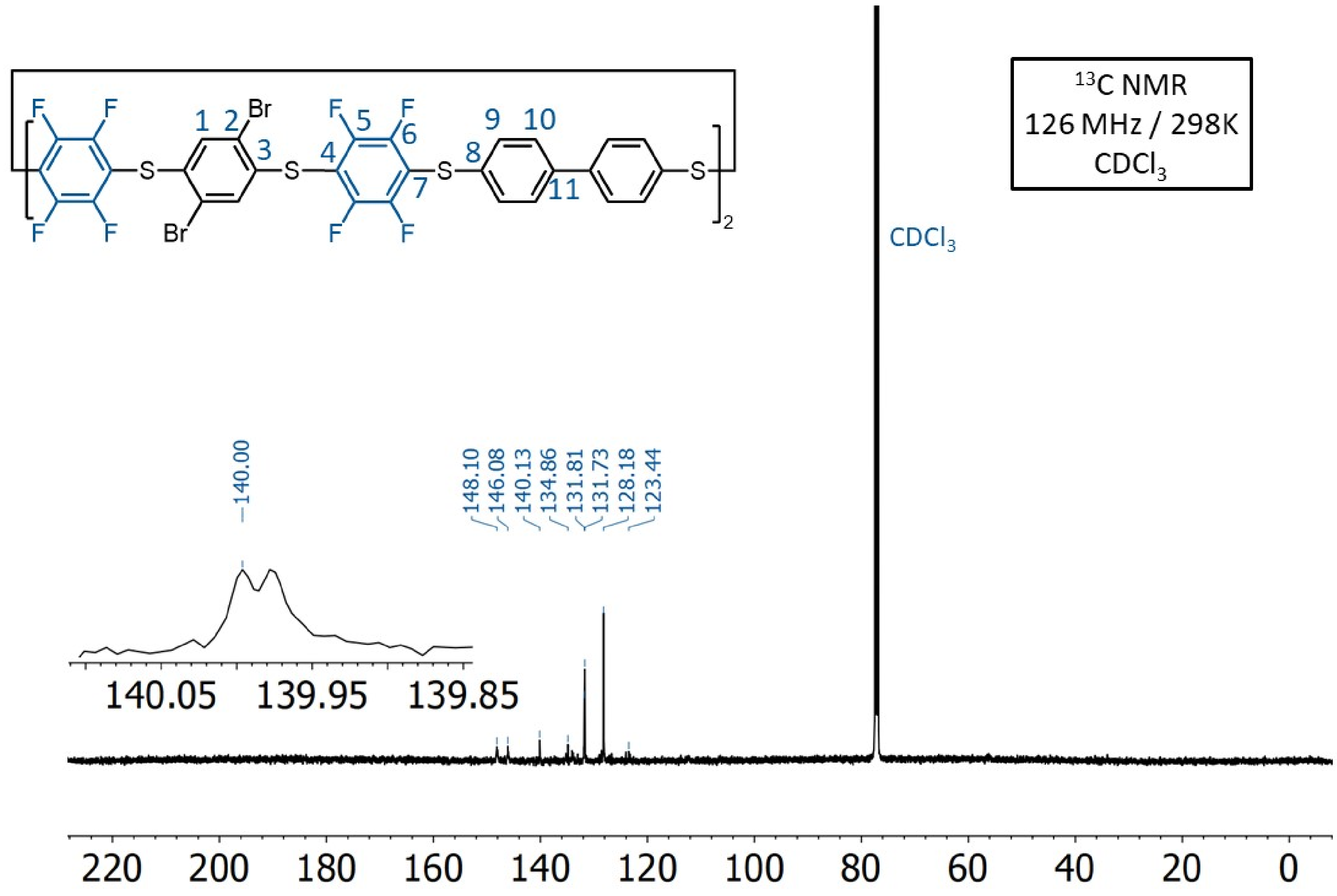

Figure S77. ^{13}C NMR Spectrum of **1cd**.

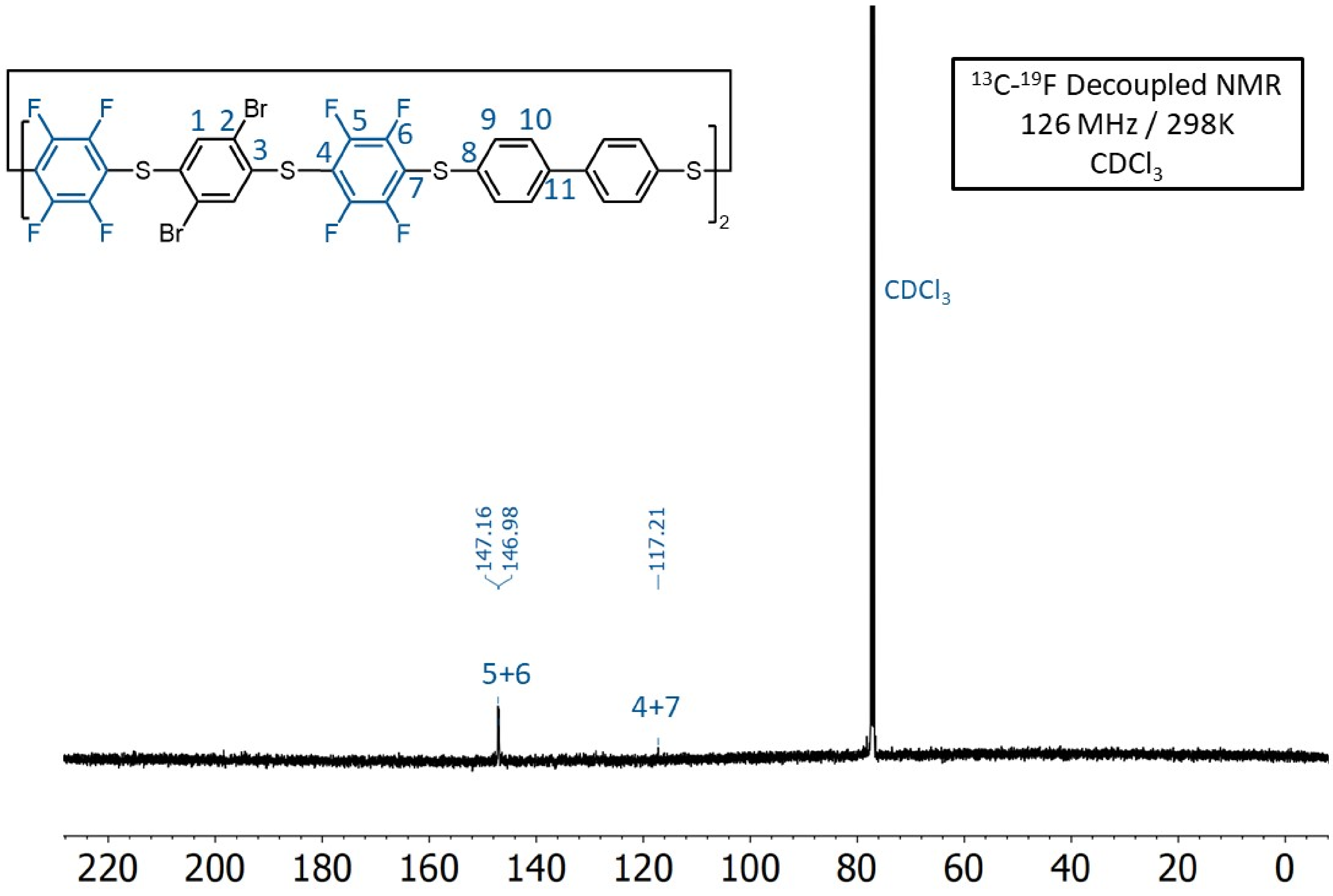

Figure S78. ^{13}C-^{19}F Decoupled NMR Spectrum of **1cd**.

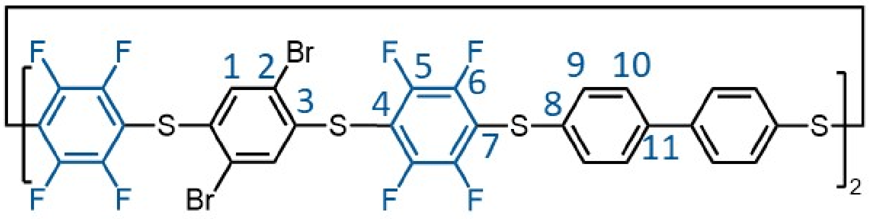

^{19}F NMR
471 MHz / 298K
$CDCl_3$

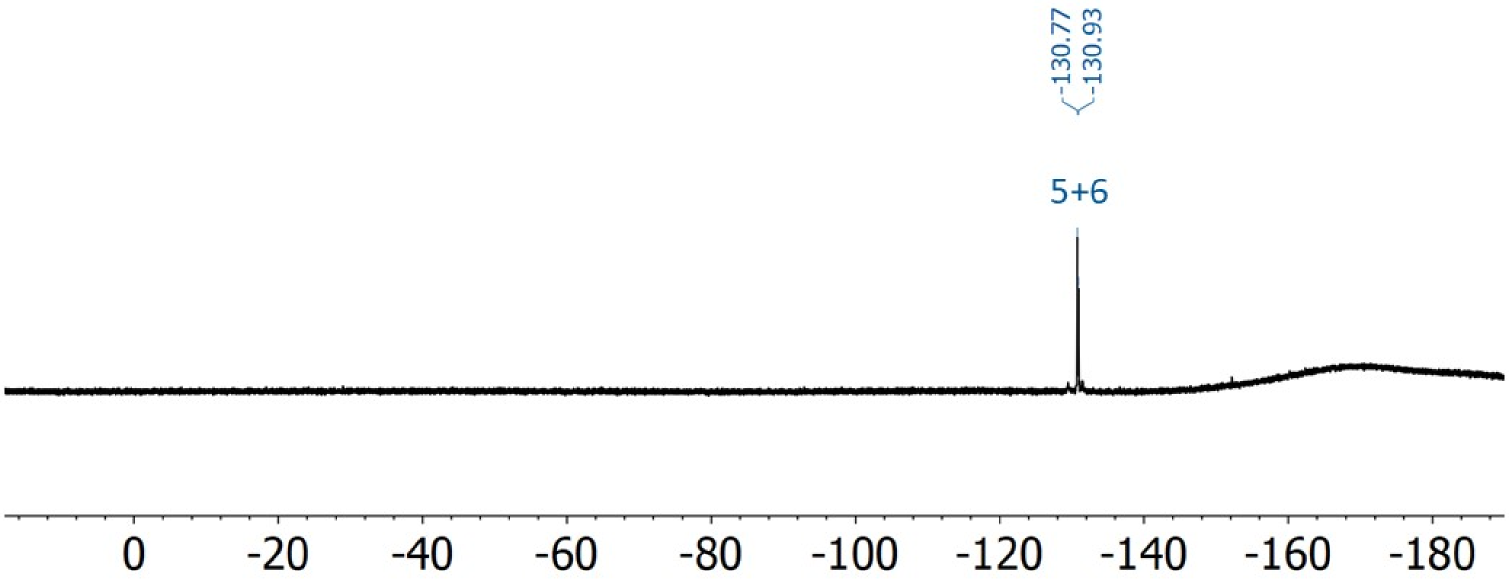

Figure S79. ^{19}F NMR Spectrum of **1cd**.

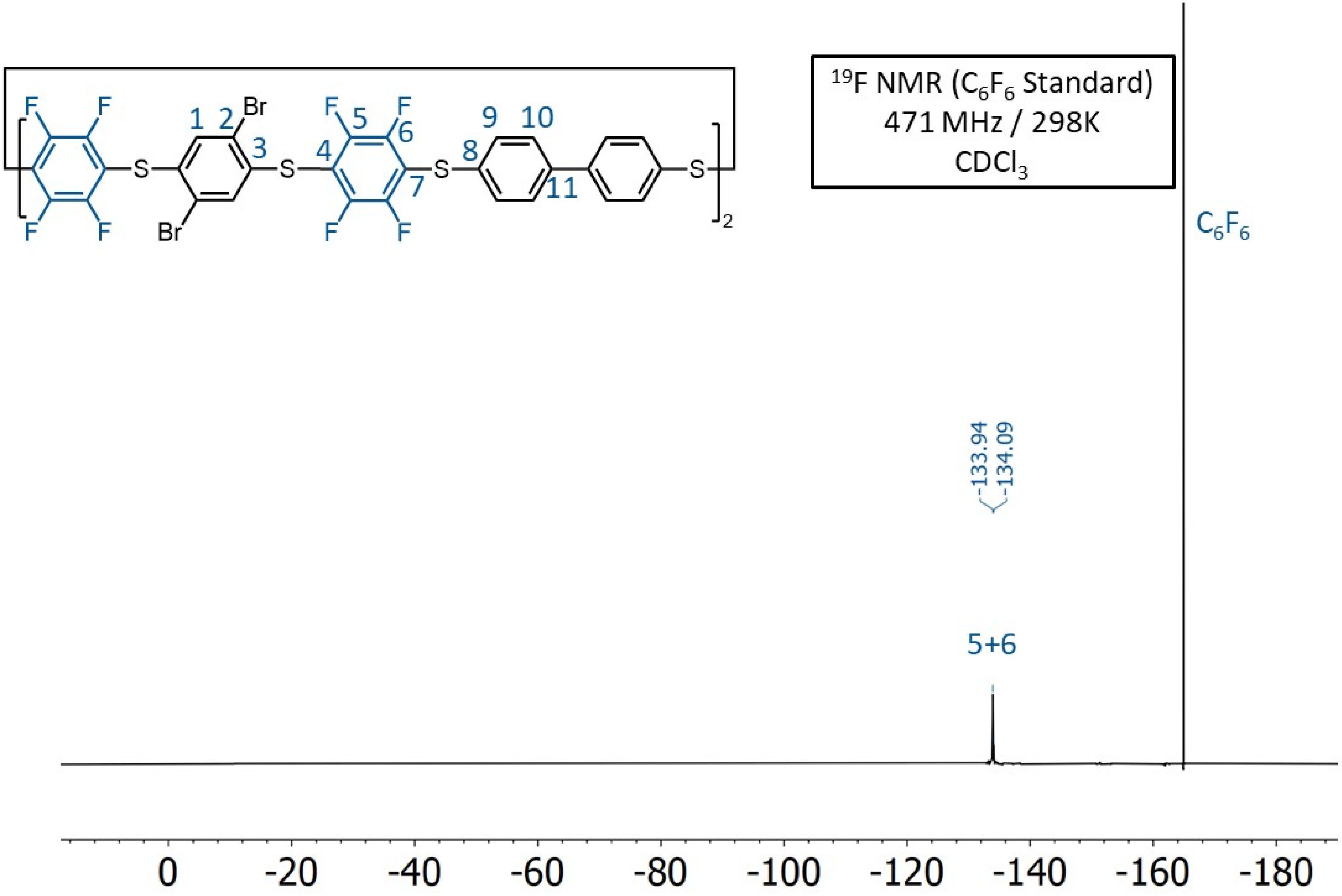

Figure S80. Referenced ^{19}F NMR Spectrum of **1cd**.

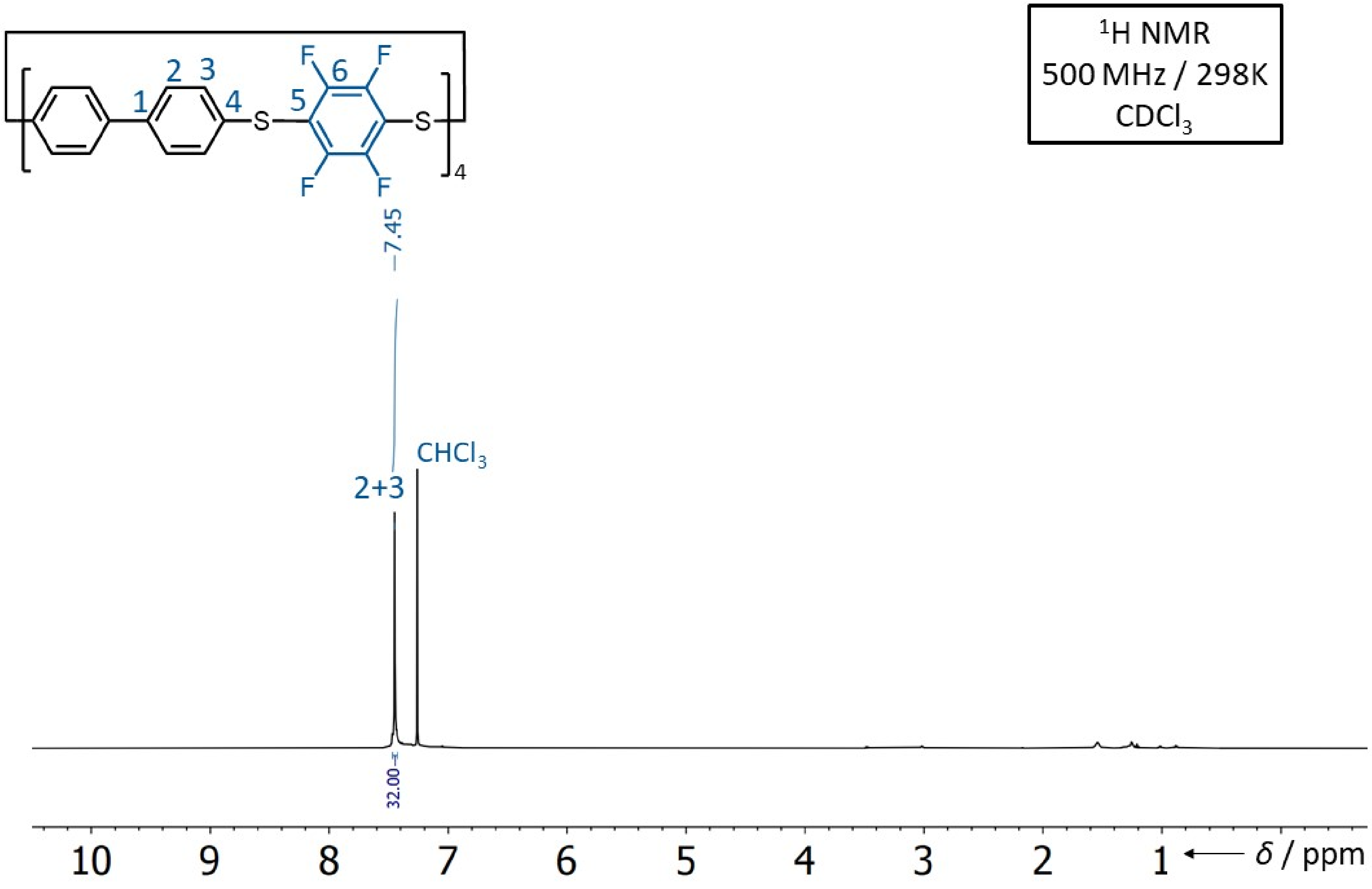

Figure S81. ^{1}H NMR Spectrum of **1d**.

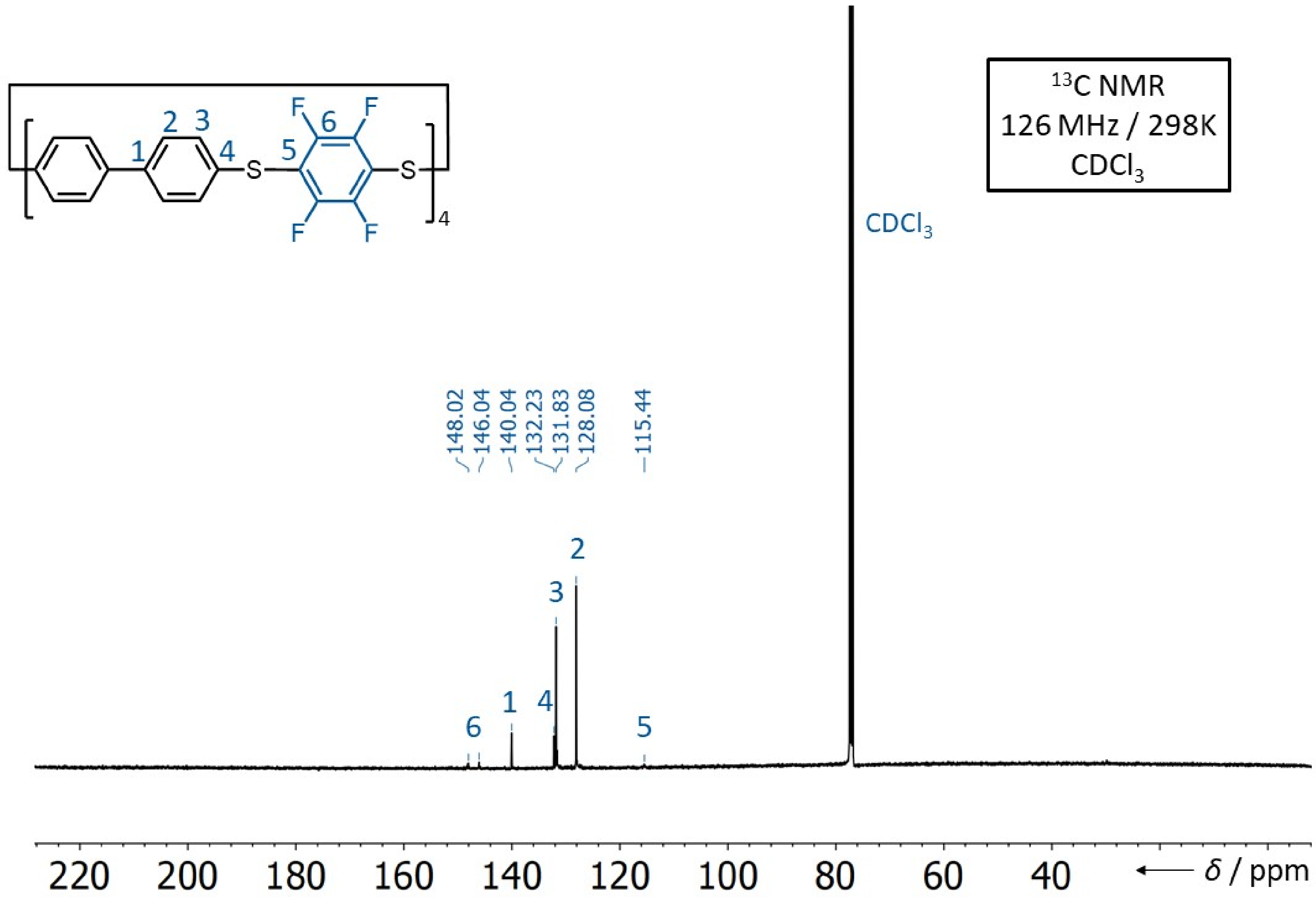

Figure S82. ^{13}C NMR Spectrum of **1d**.

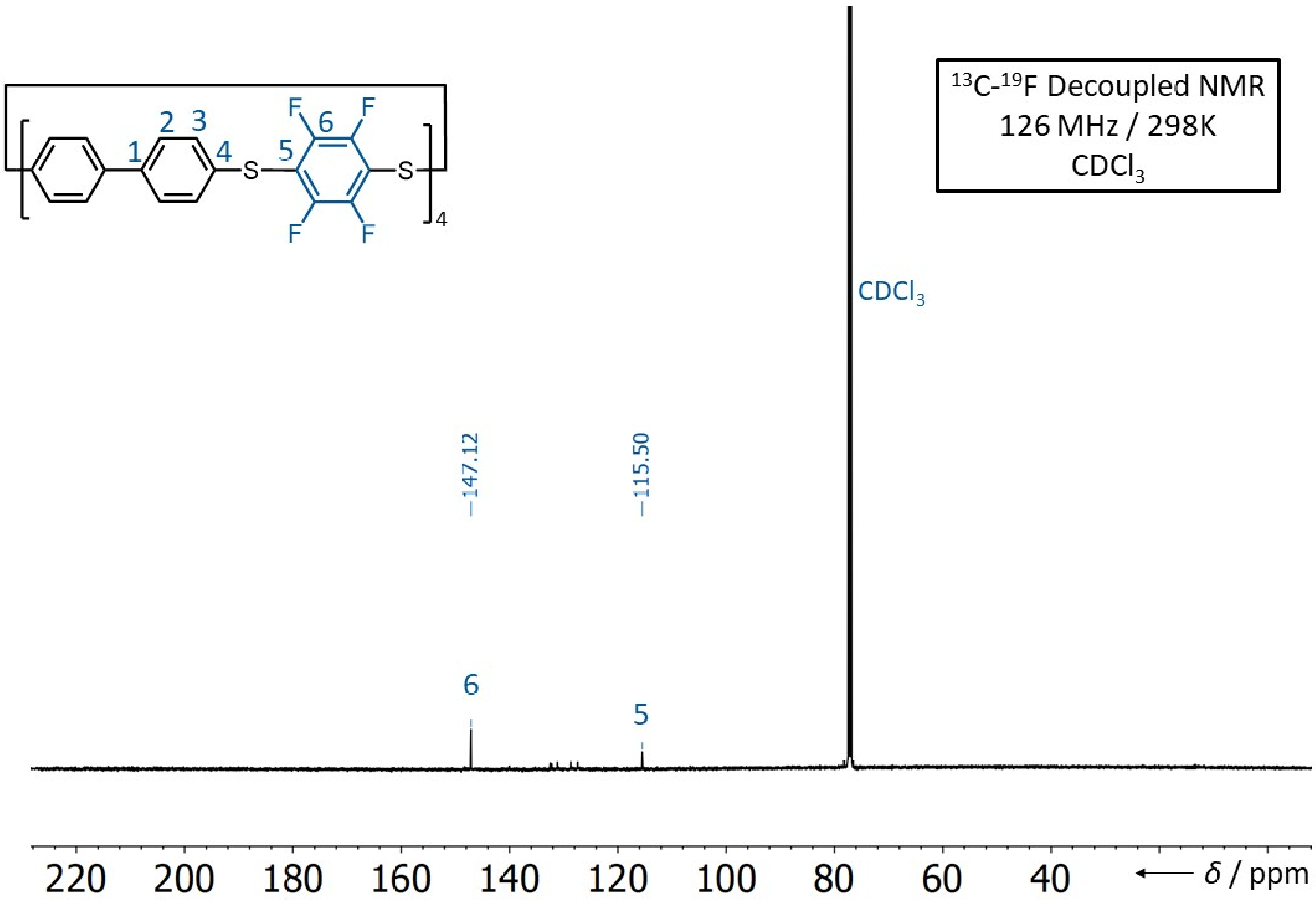

Figure S83. ^{13}C-^{19}F NMR Spectrum of **1d**.

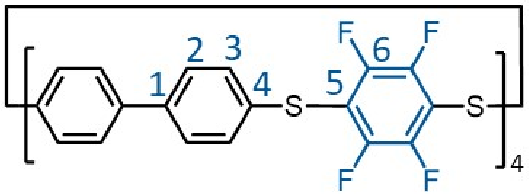

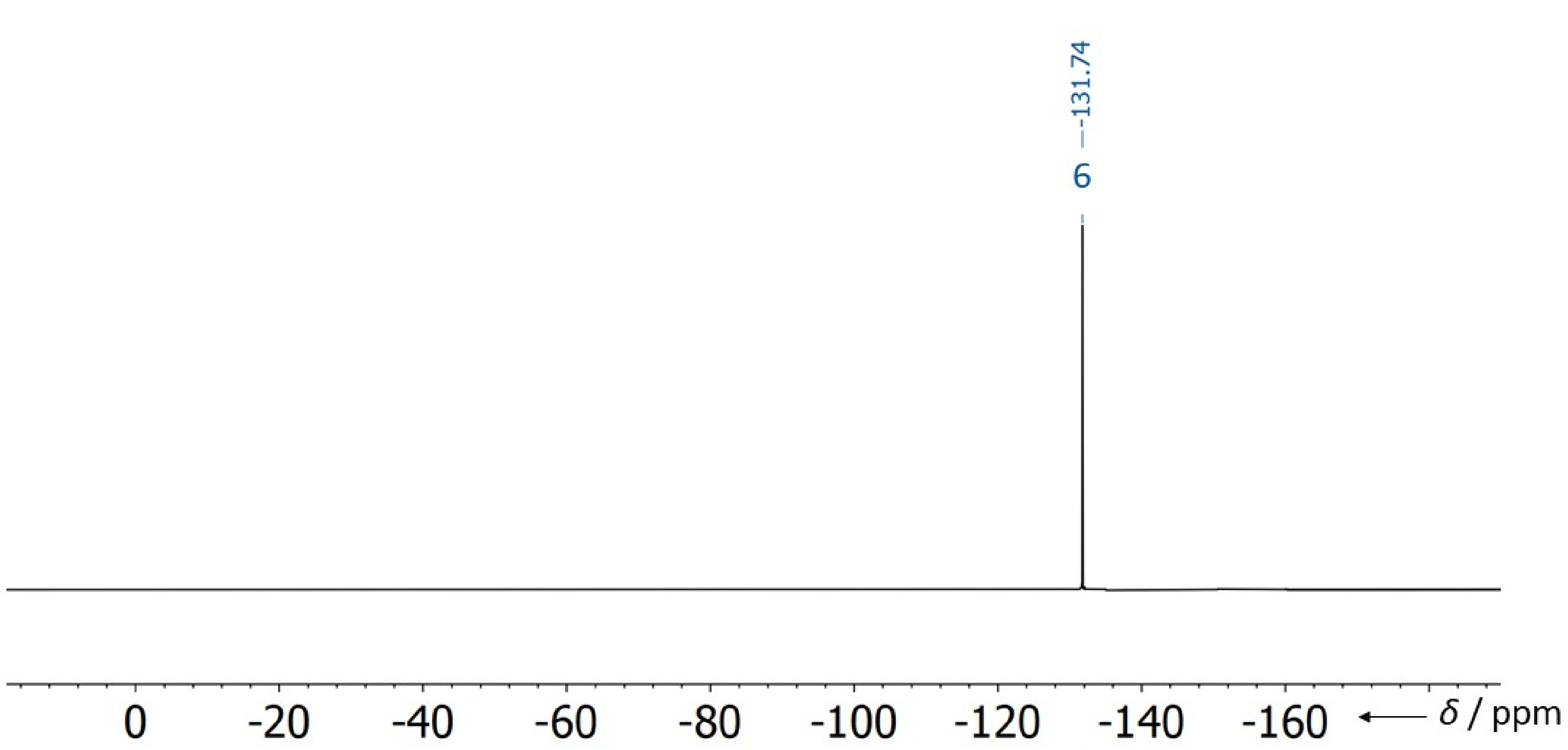

Figure S84. ^{19}F NMR Spectrum of **1d**.

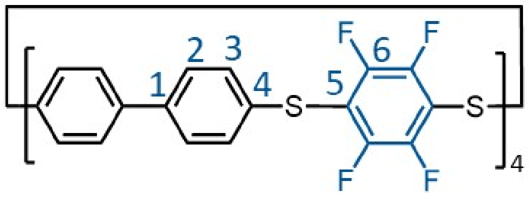

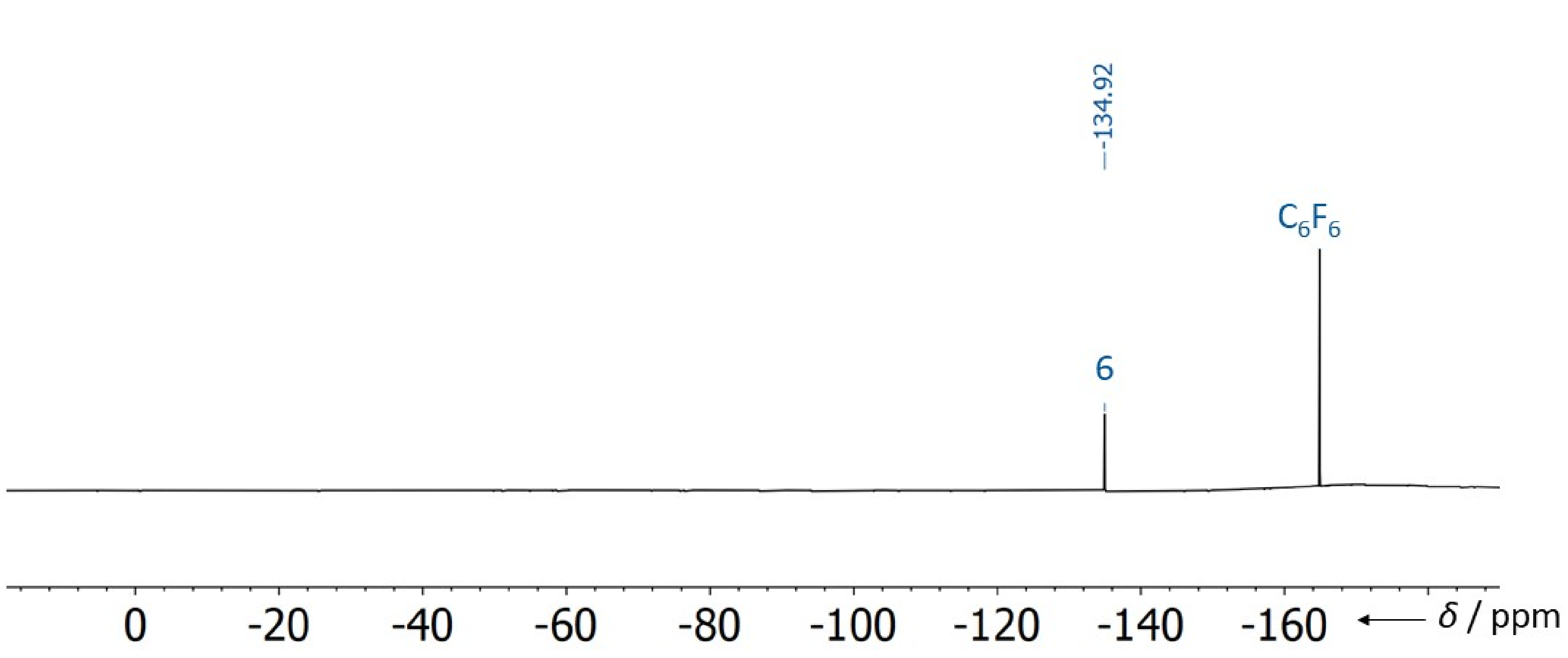

Figure S85. ^{19}F NMR Spectrum of **1d**.

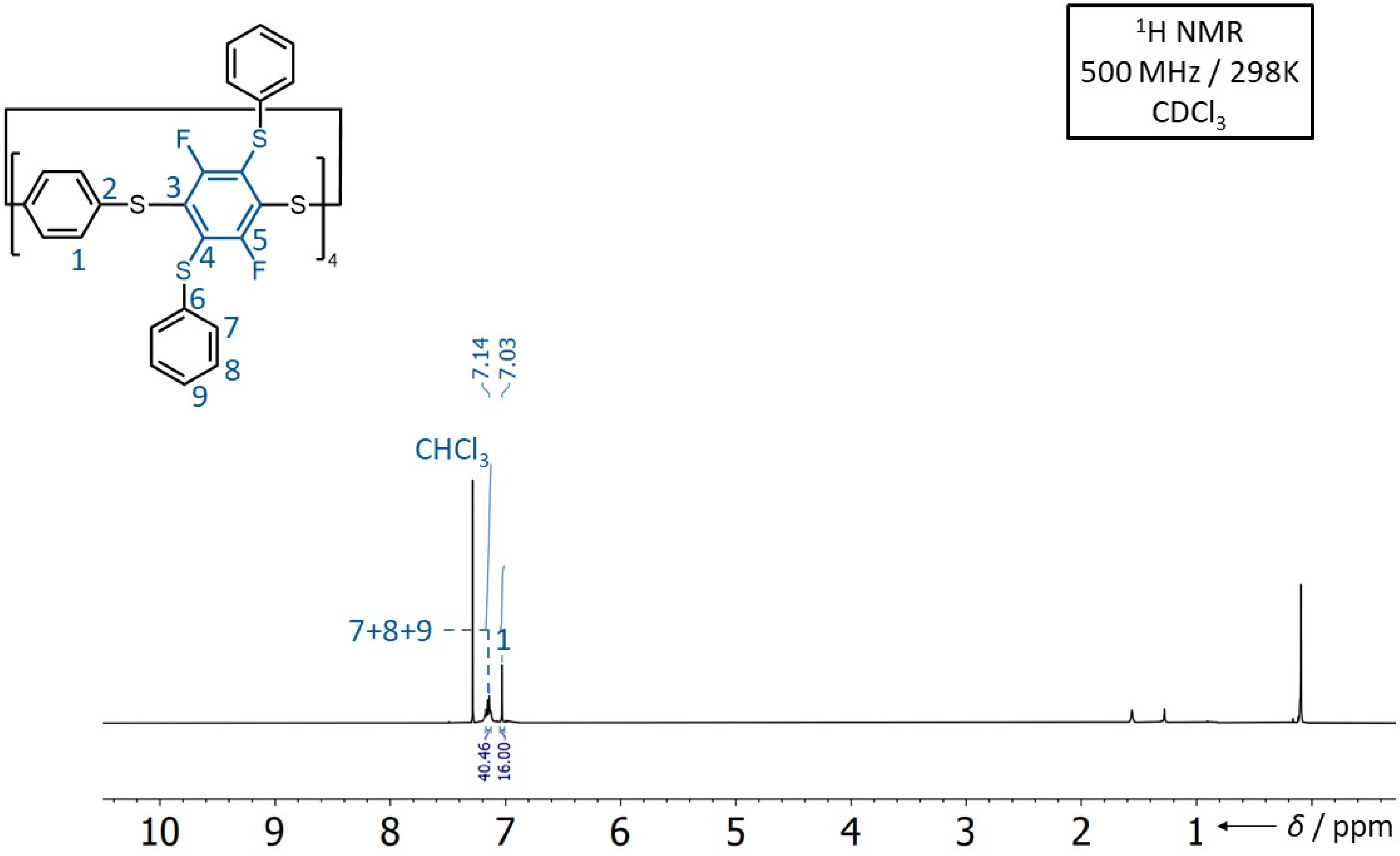

Figure S86. ^{1}H NMR Spectrum of **4a**.

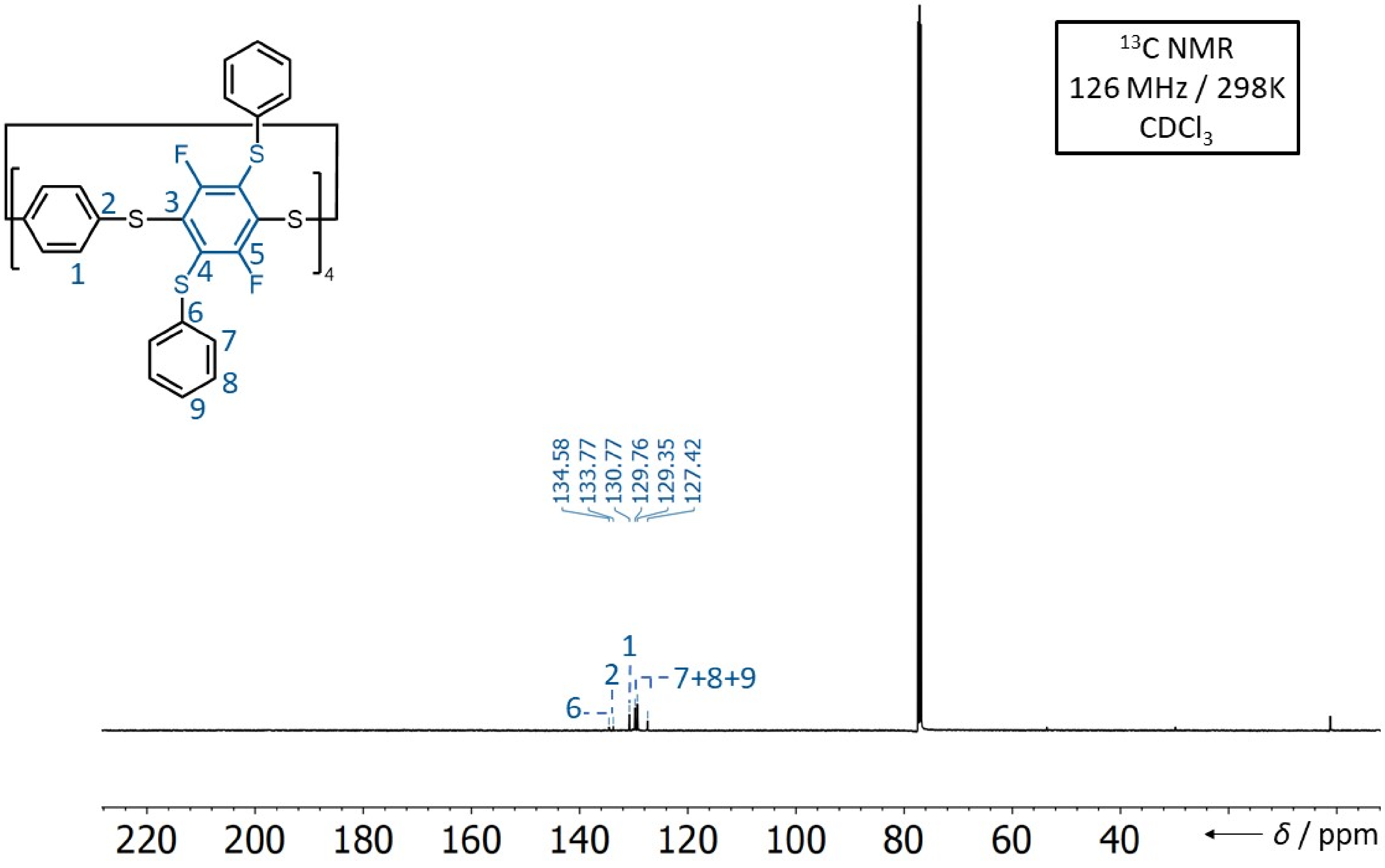

Figure S87. ^{13}C NMR Spectrum of **4a**.

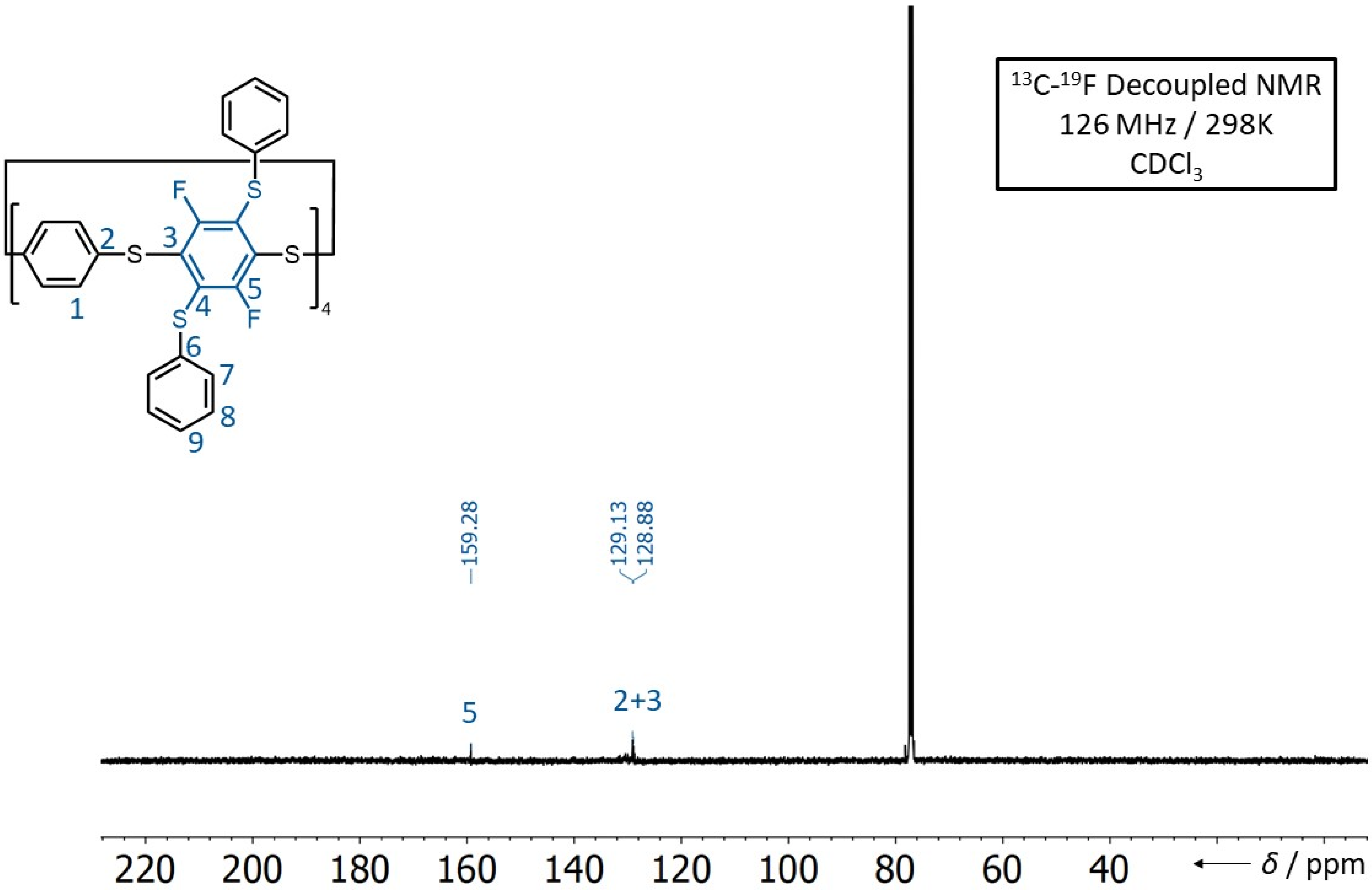

Figure S88. ^{13}C-^{19}F Decoupled NMR Spectrum of **4a**.

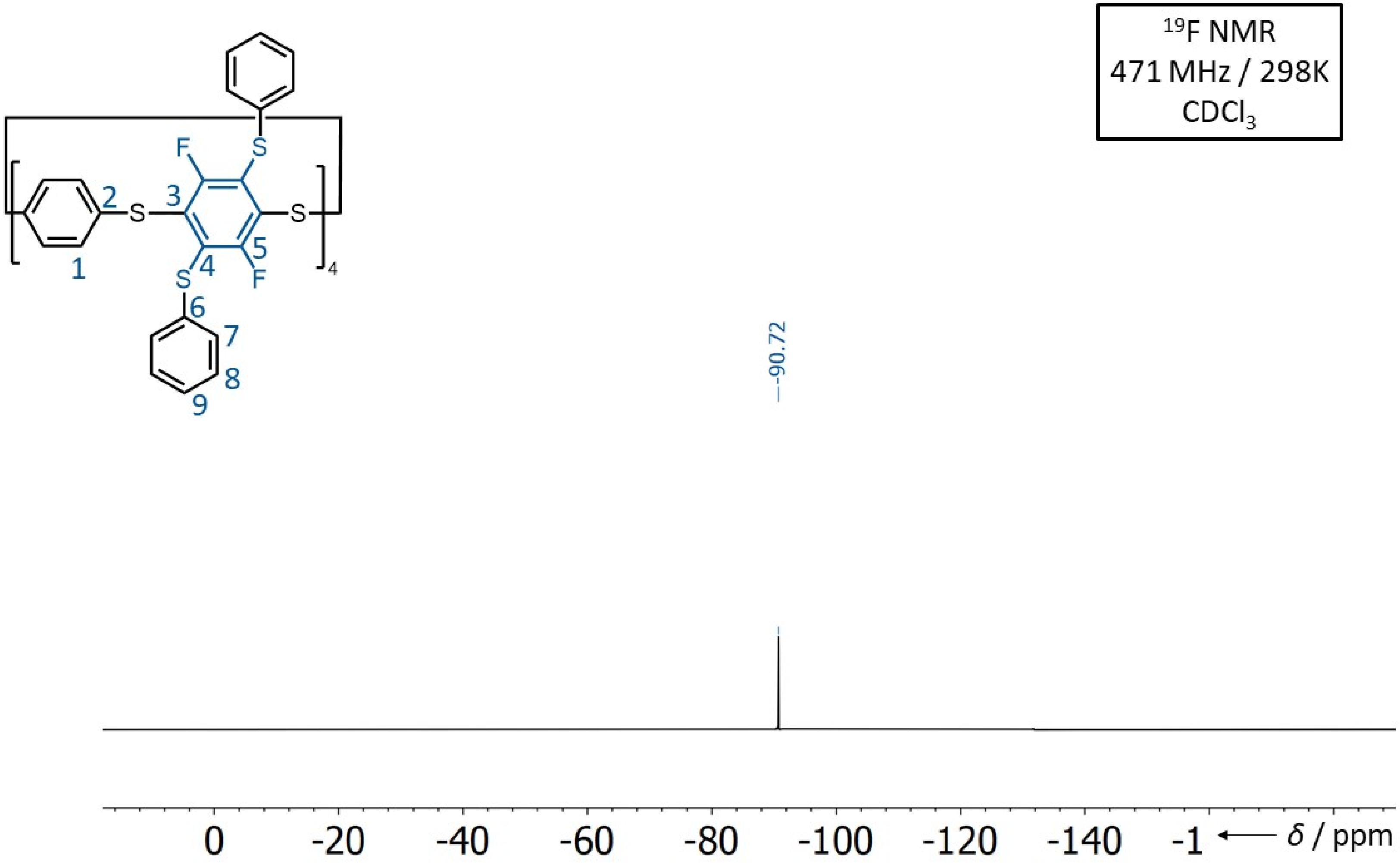

Figure S89. ^{19}F NMR Spectrum of **4a**.

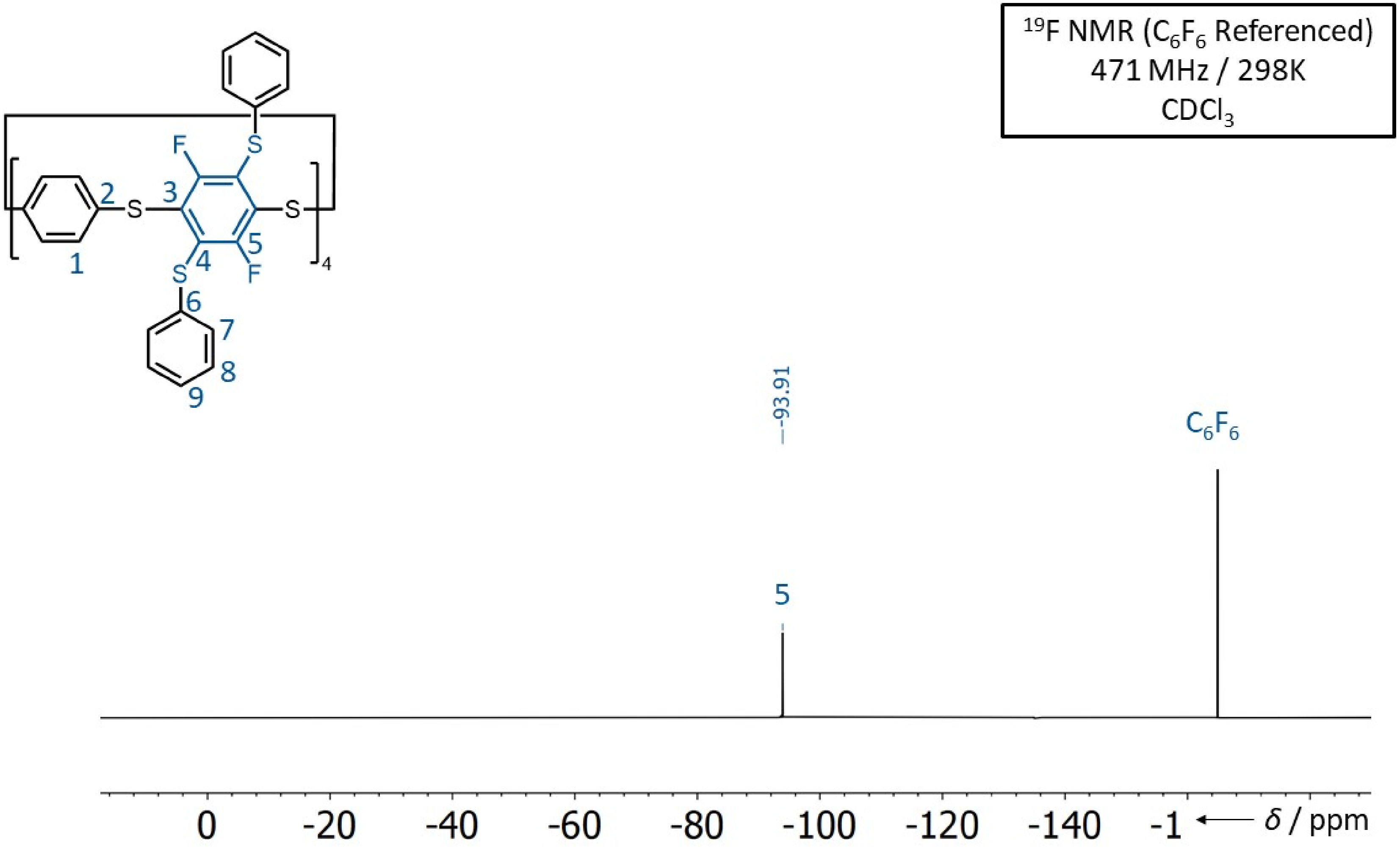

Figure S90. Referenced ^{19}F NMR Spectrum of **4a**.

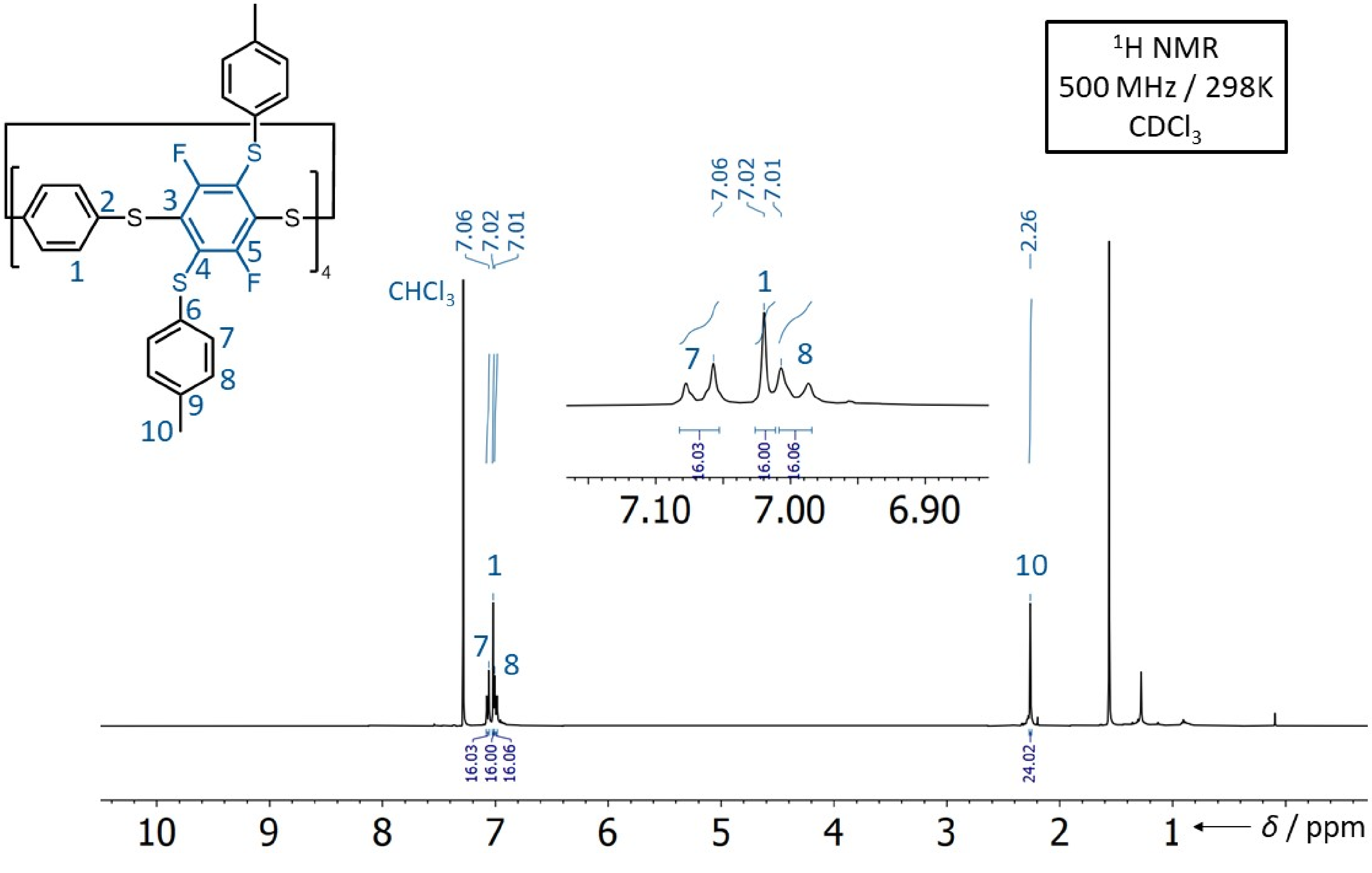

Figure S91. ^{1}H NMR Spectrum of **5a**.

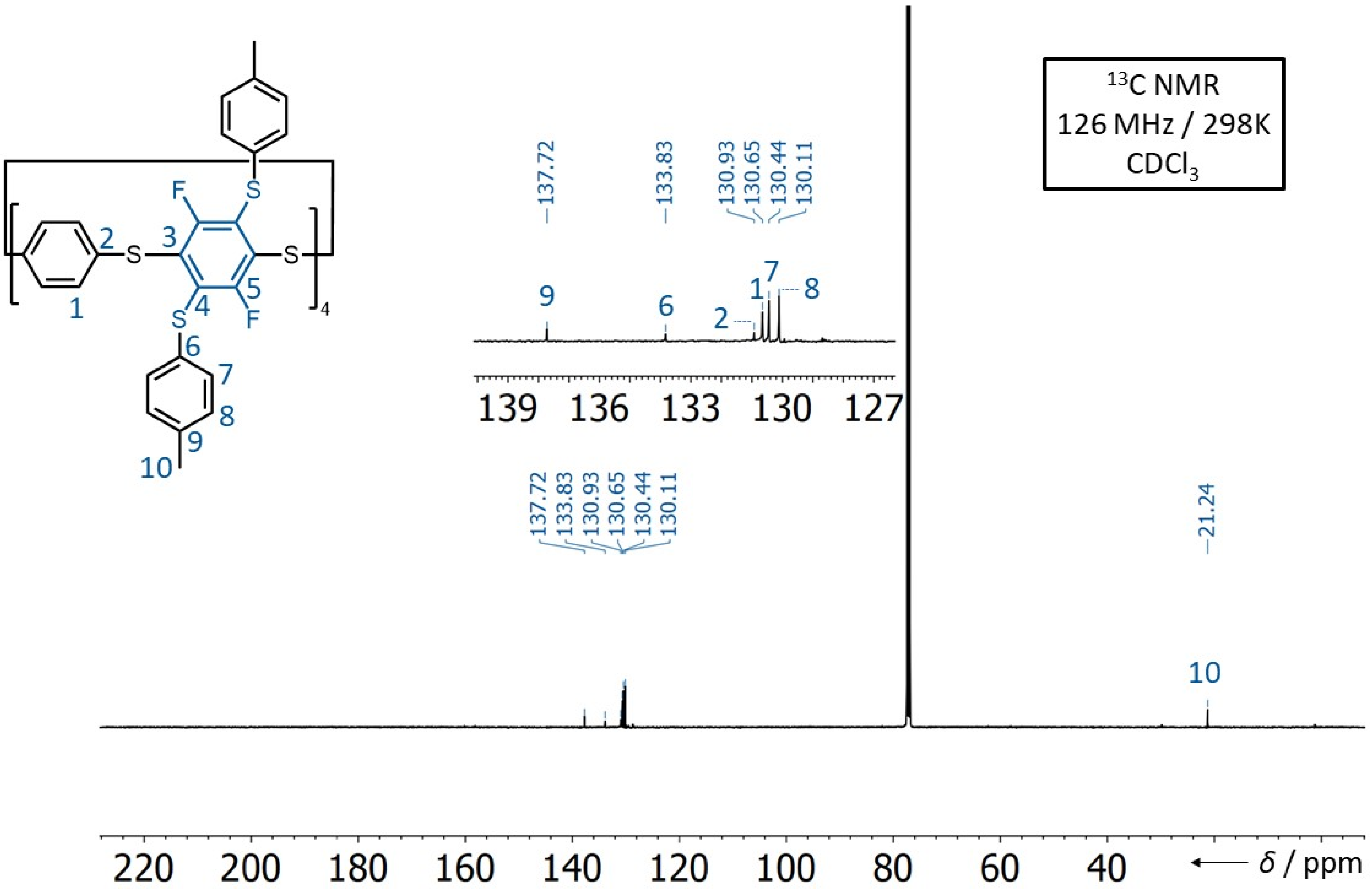

Figure S92. ^{13}C NMR Spectrum of **5a**.

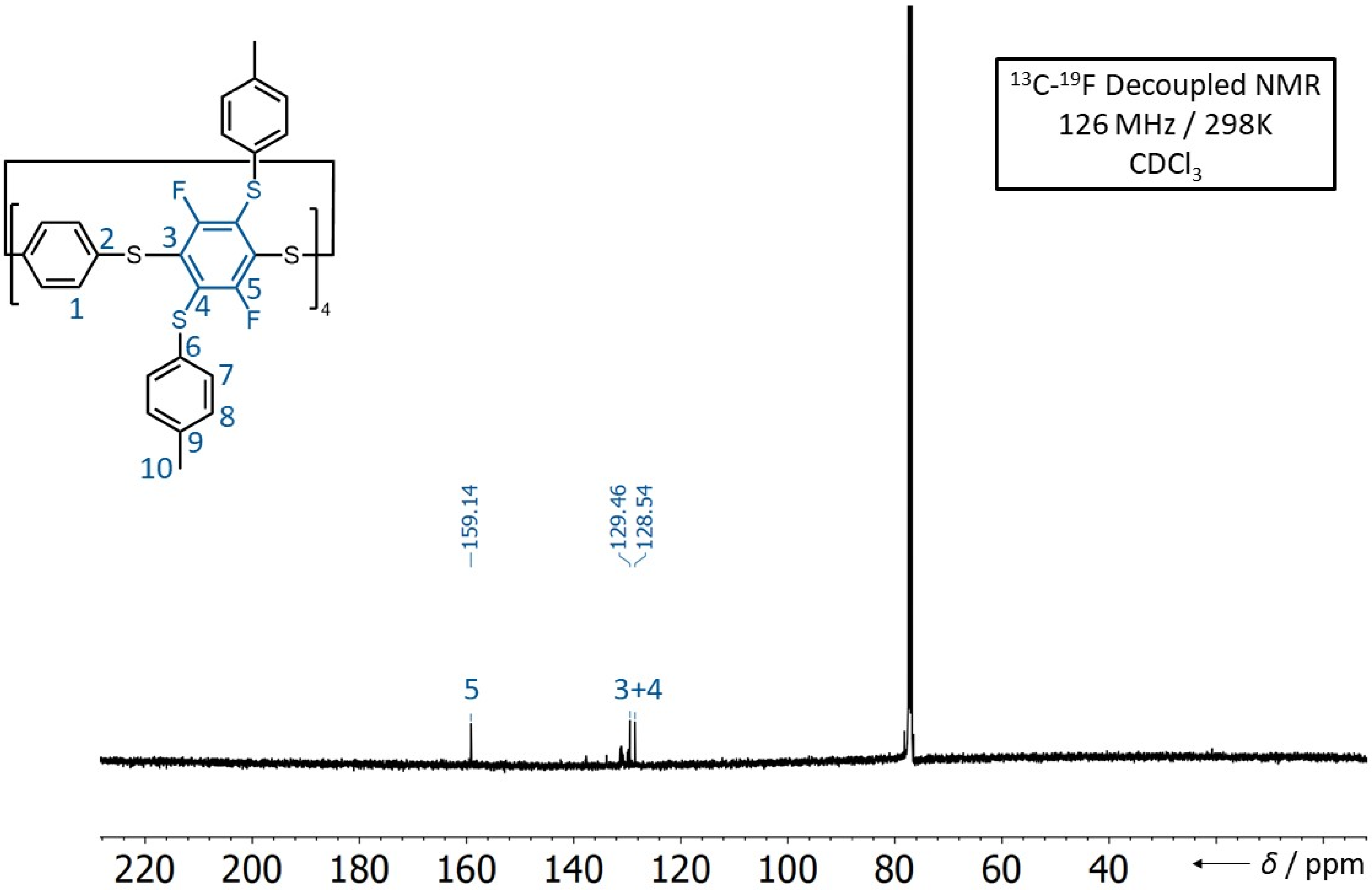

Figure S93. ^{13}C-^{19}F Decoupled NMR Spectrum of **5a**.

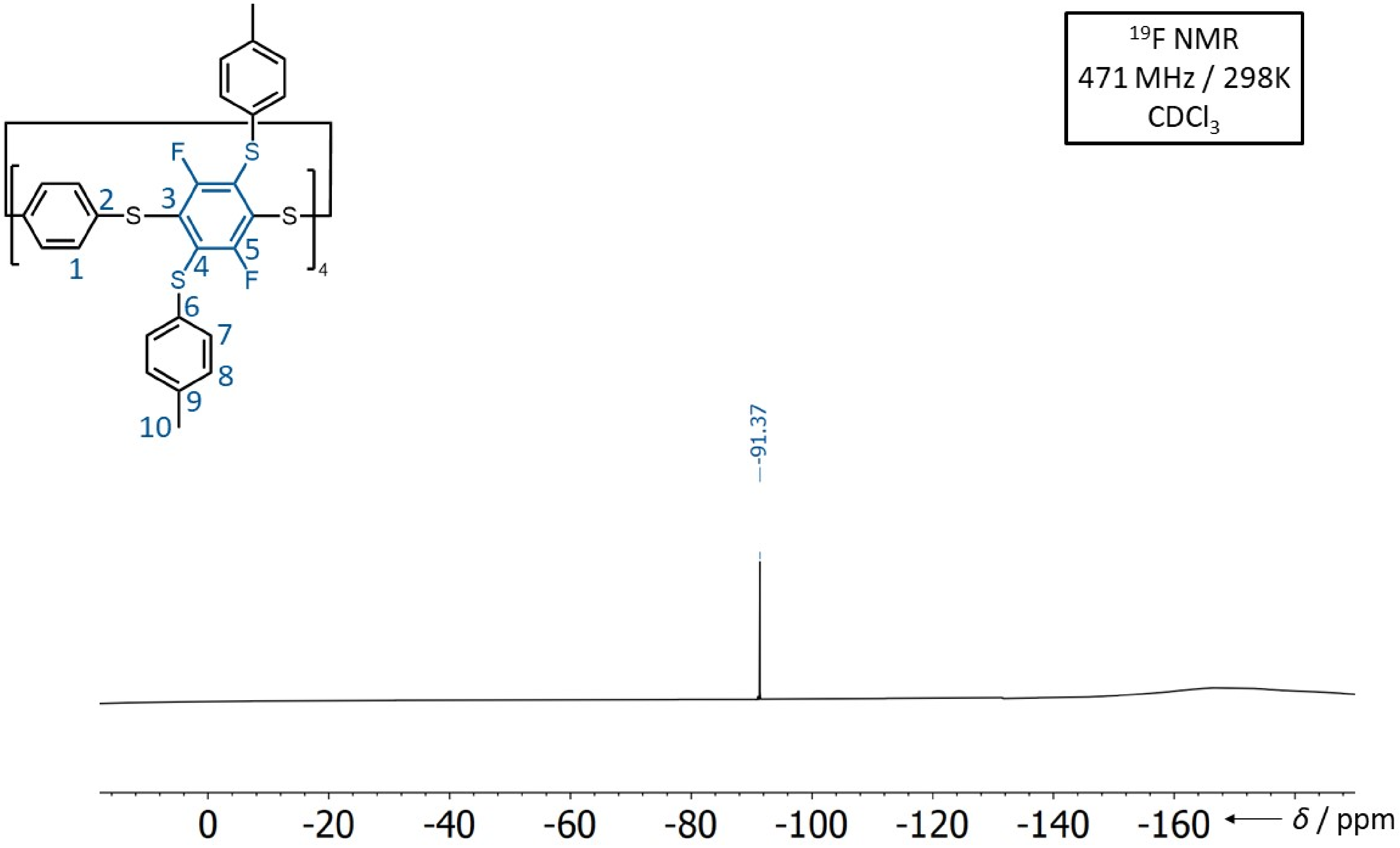

Figure S94. ^{19}F NMR Spectrum of **5a**.

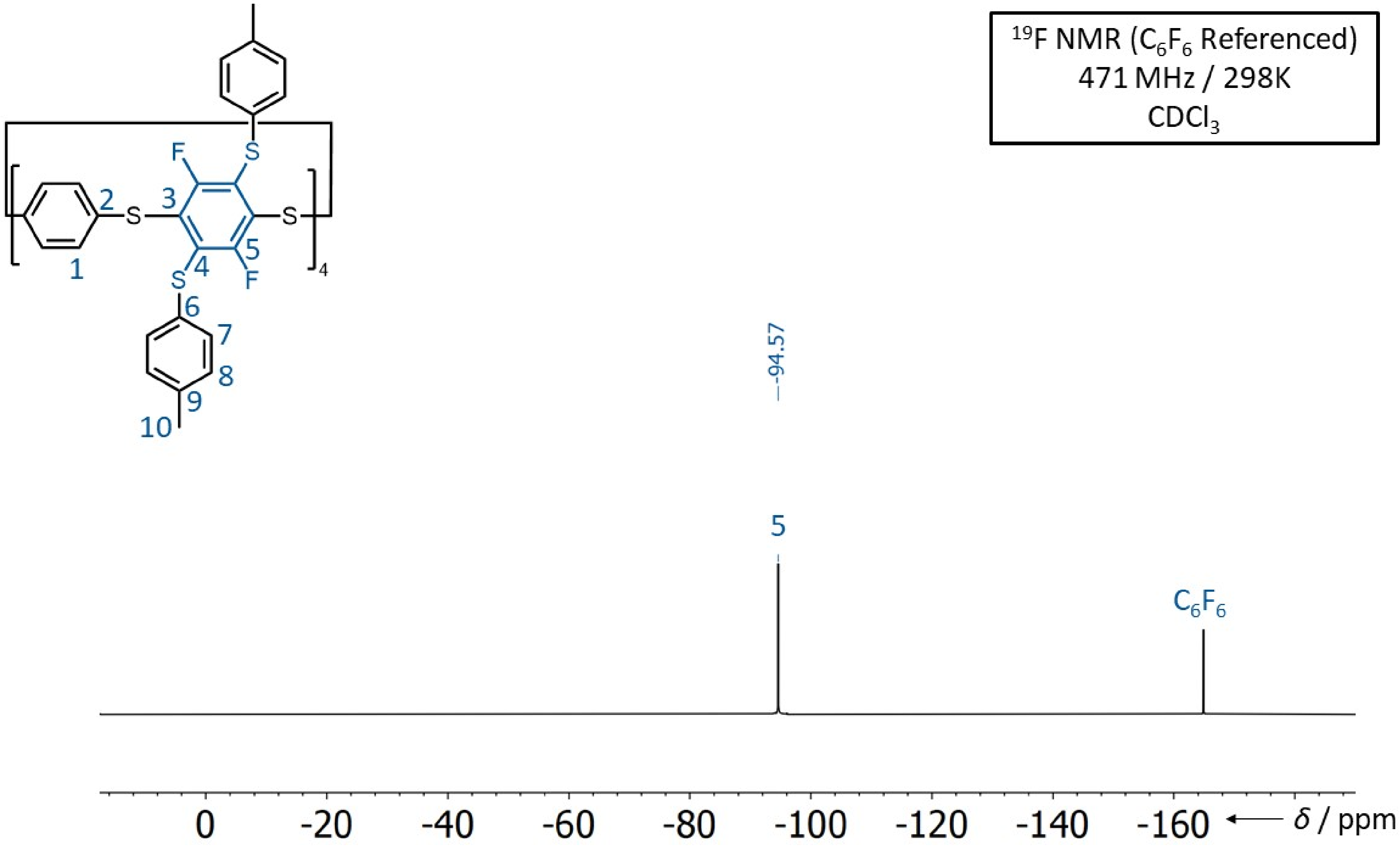

Figure S95. Referenced ^{19}F NMR Spectrum of **5a**.

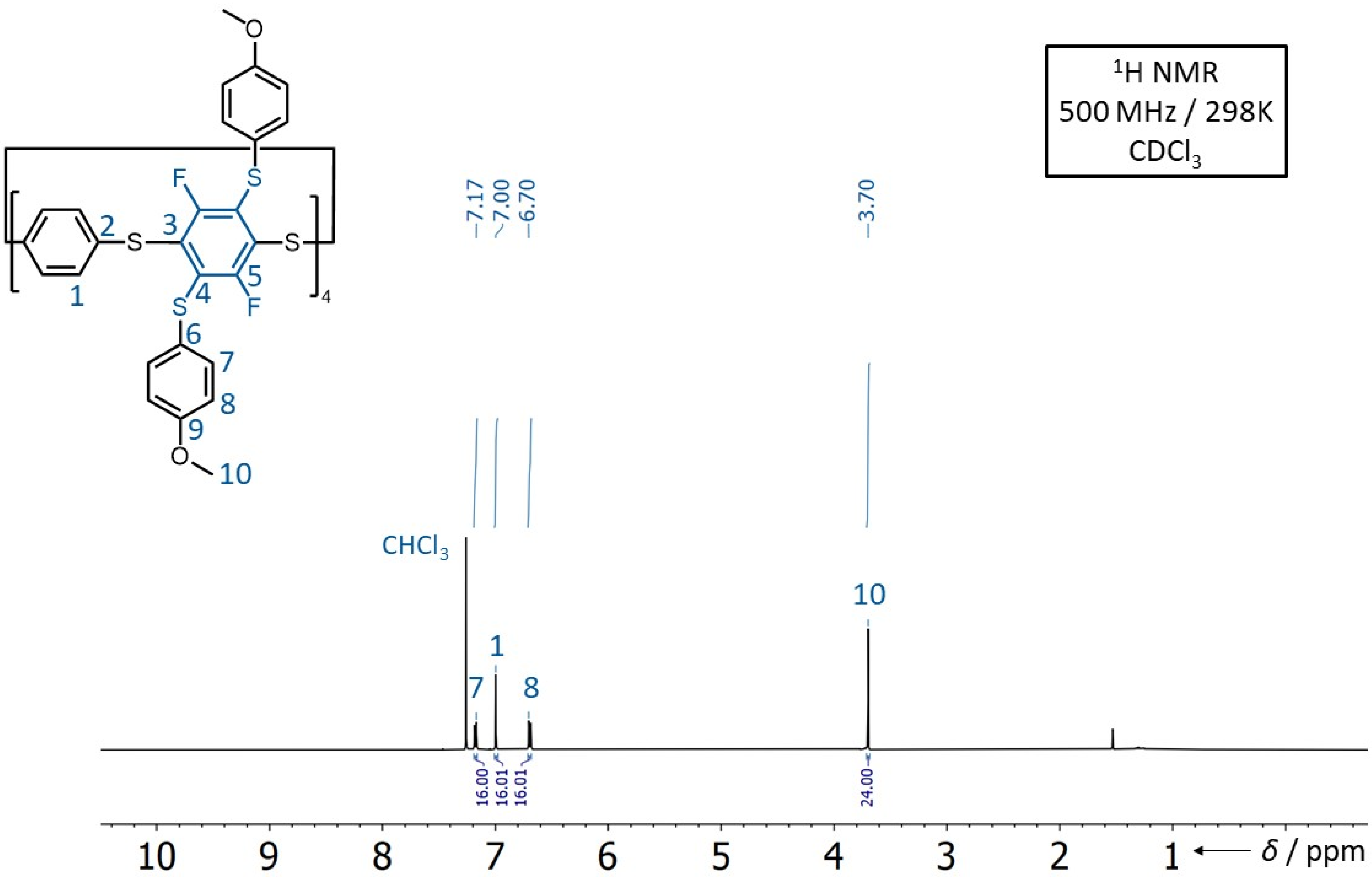

Figure S96. ¹H NMR Spectrum of **5b**.

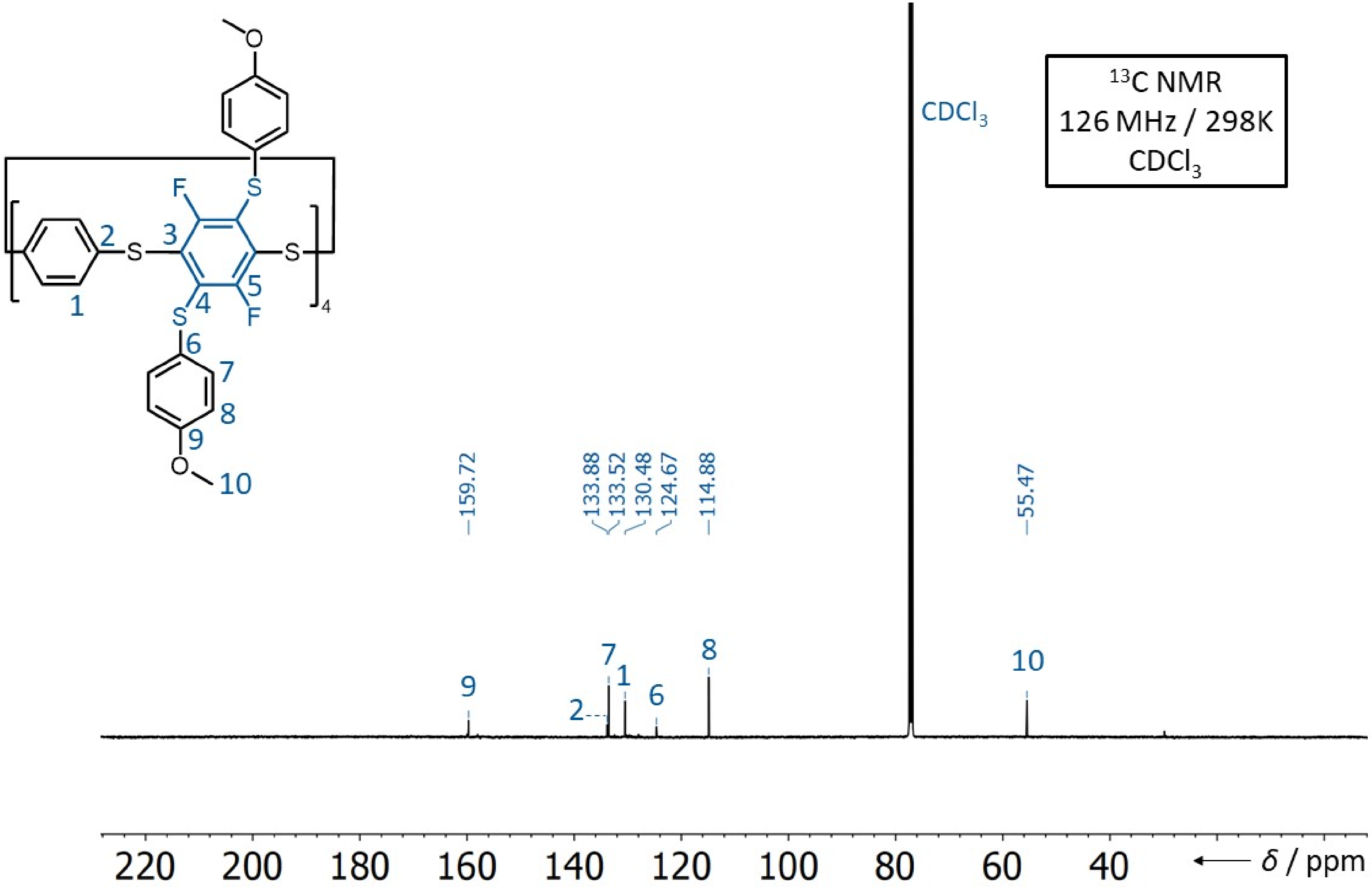

Figure S97. ^{13}C NMR Spectrum of **5b**.

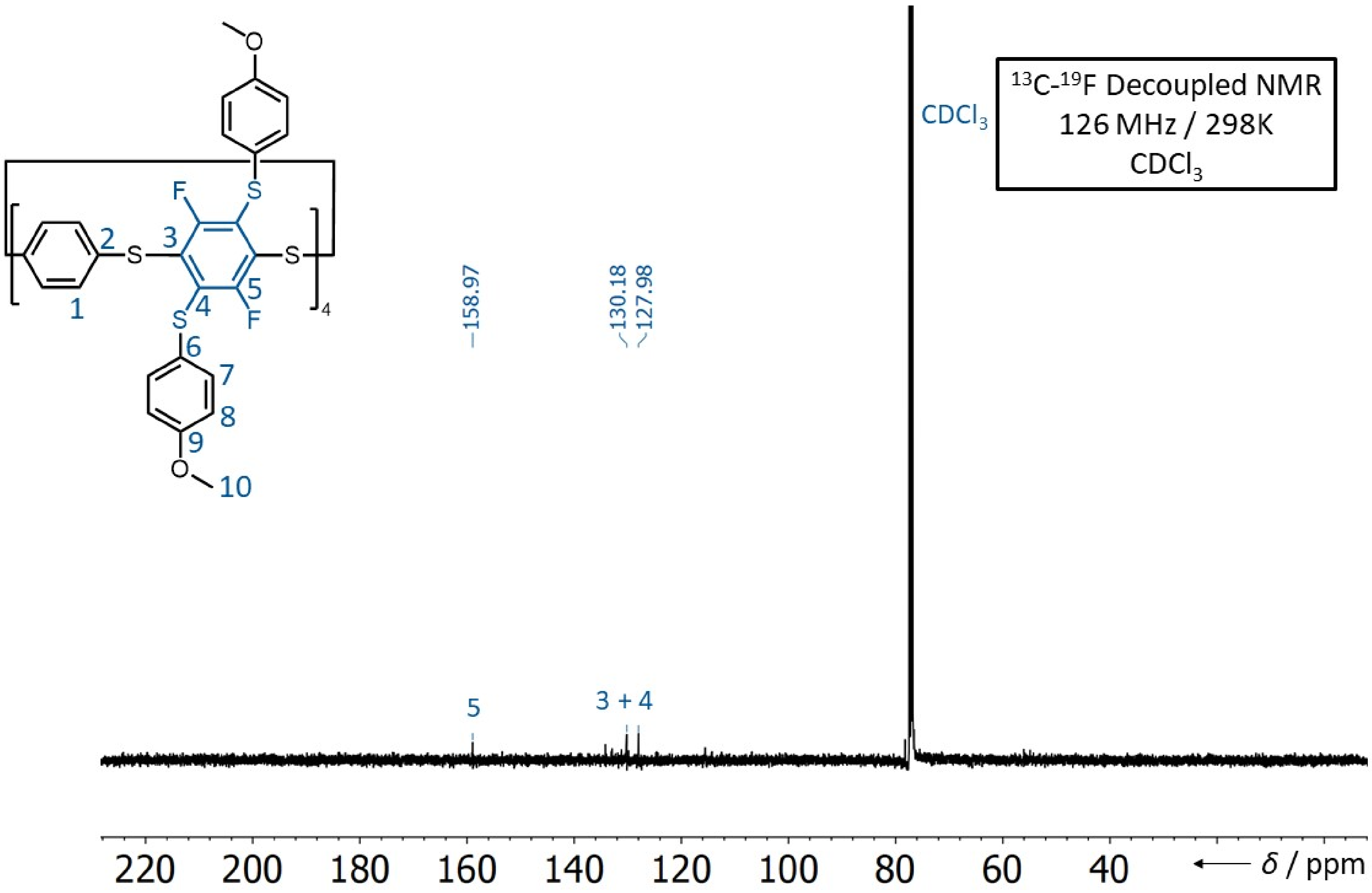

Figure S98. ^{13}C-^{19}F Decoupled NMR Spectrum of **5b**.

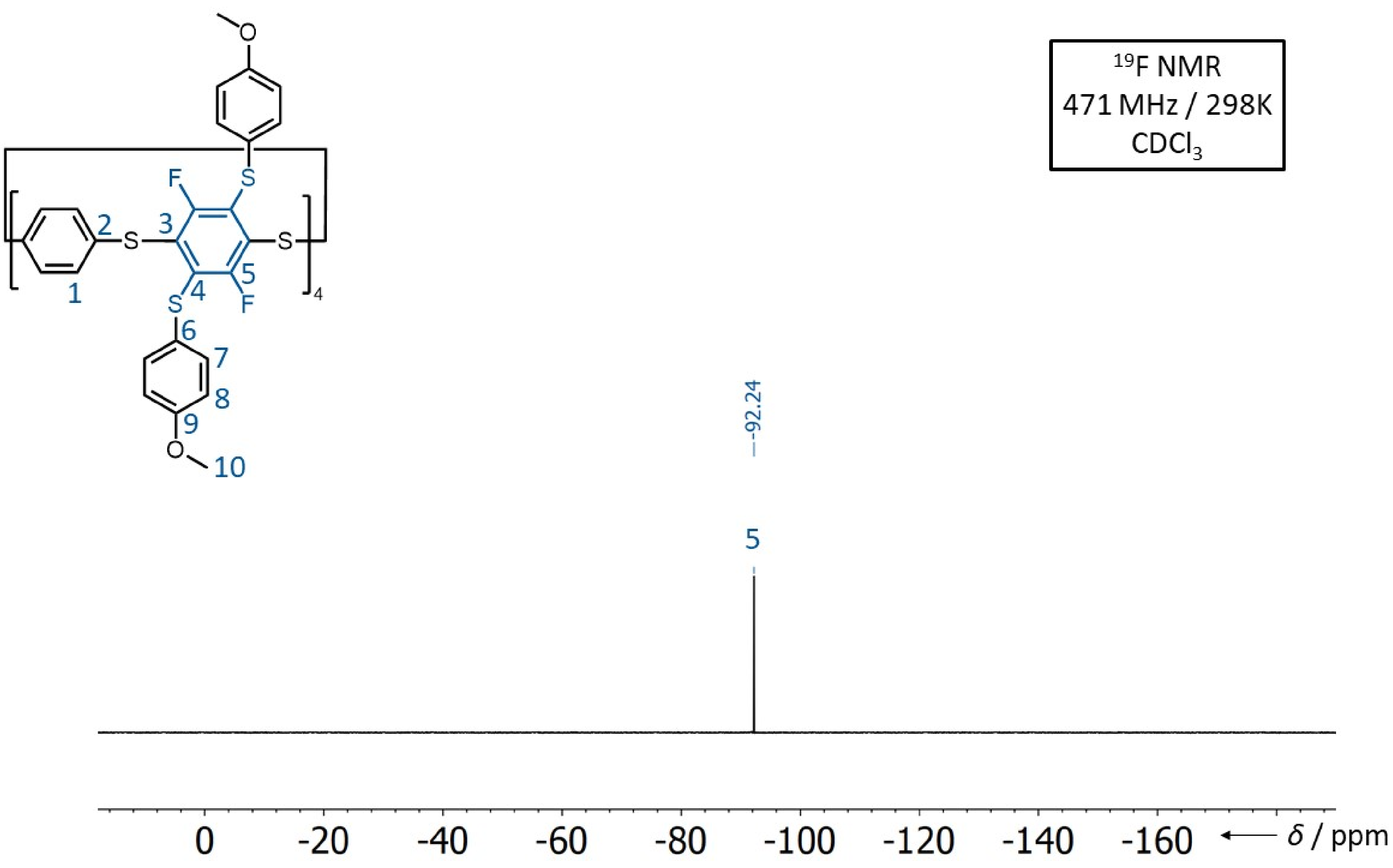

Figure S99. ^{19}F NMR Spectrum of **5b**.

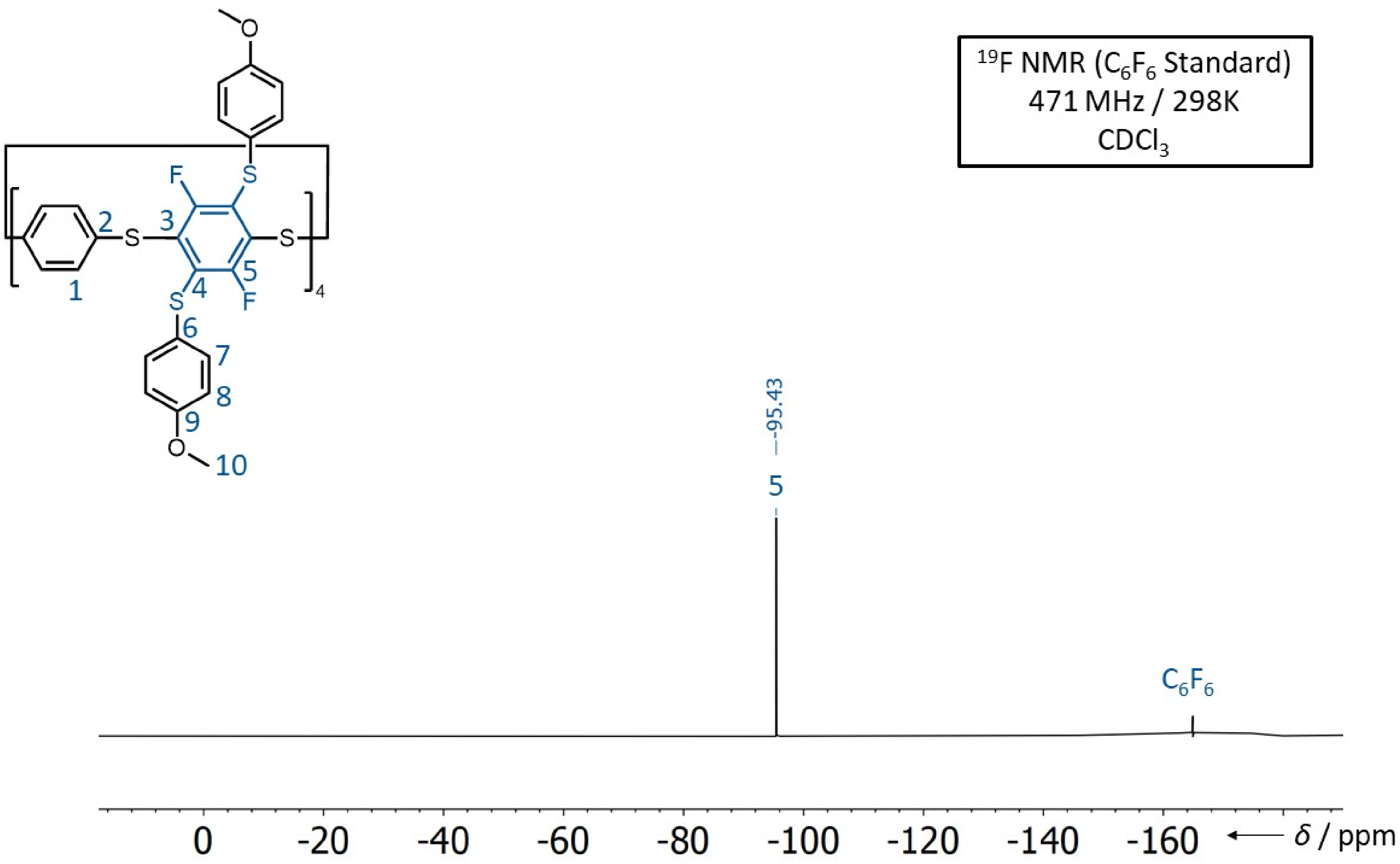

Figure S100. Referenced ^{19}F NMR Spectrum of **5b**.

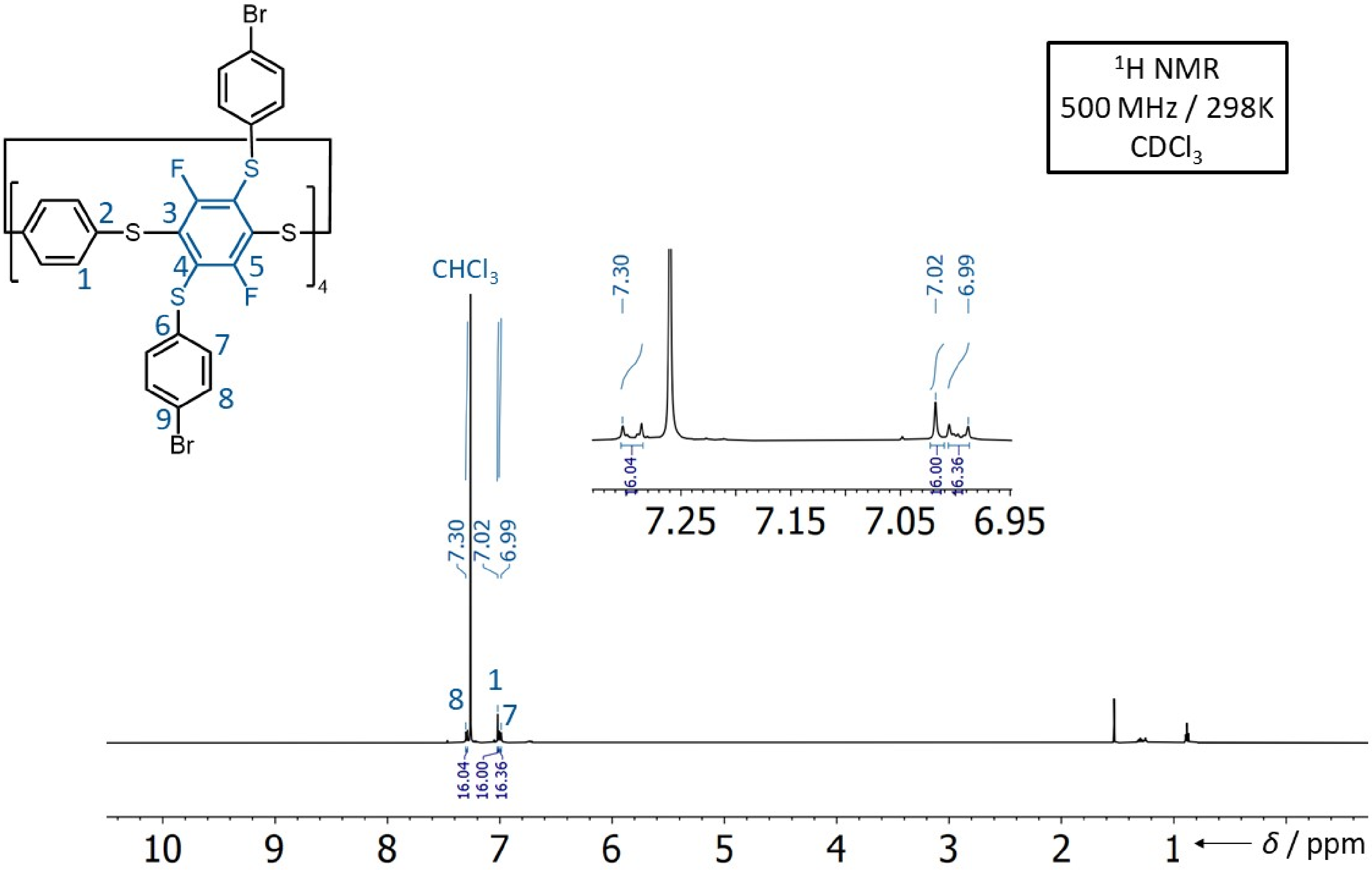
Br
F
S
2
3
S
S
1
4
5
4
S
F
6
7
8
9
Br
1H NMR
500 MHz / 298K
CDCl3
CHCl3
7.30
7.02
6.99
16.04
16.00
16.36
7.25
7.15
7.05
6.95
8
1
7
10
9
8
7
6
5
4
3
2
1
δ / ppm

Figure S101. ^{1}H NMR Spectrum of **5c**.

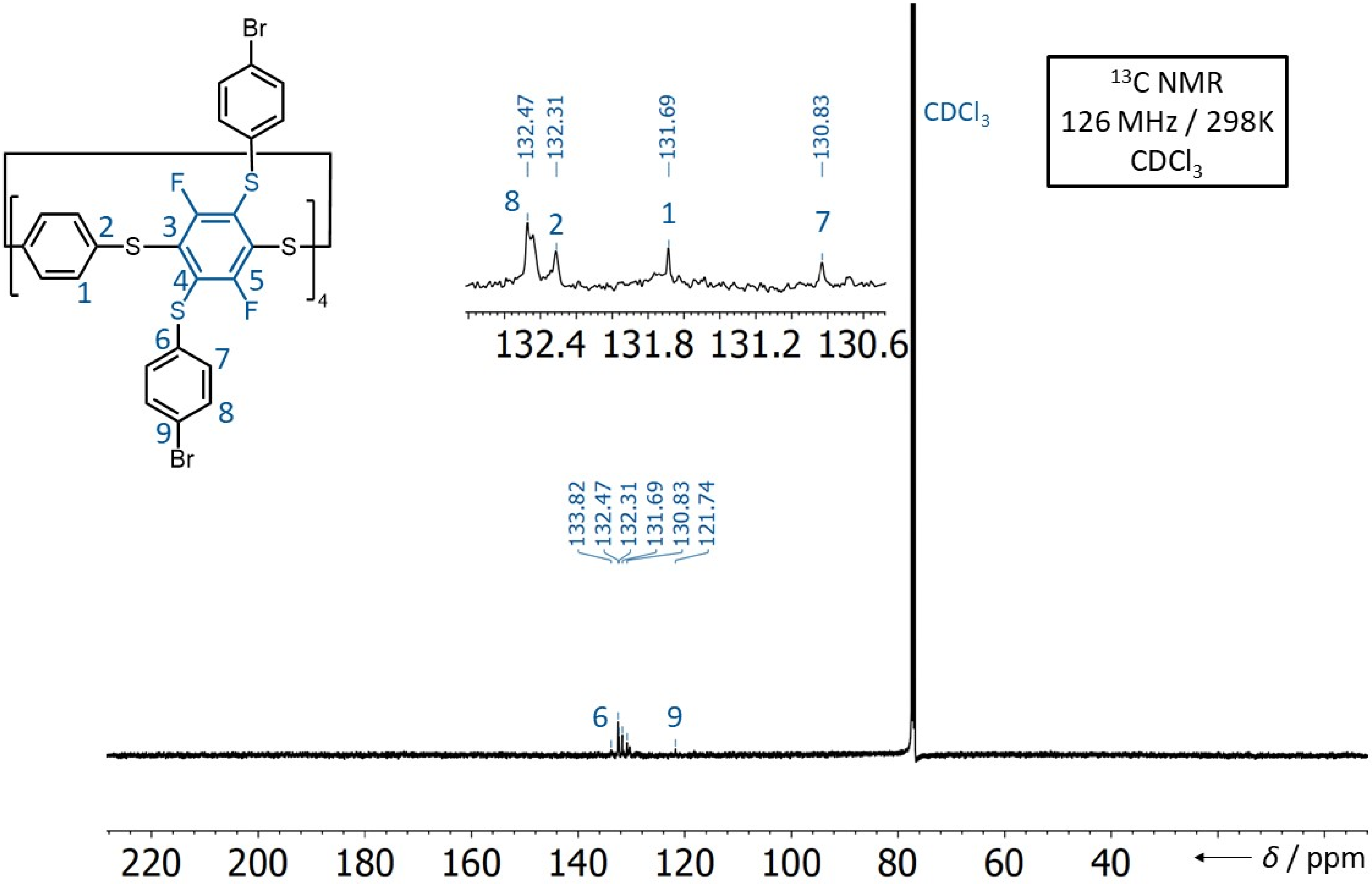

Figure S102. ^{13}C NMR Spectrum of **5c**.

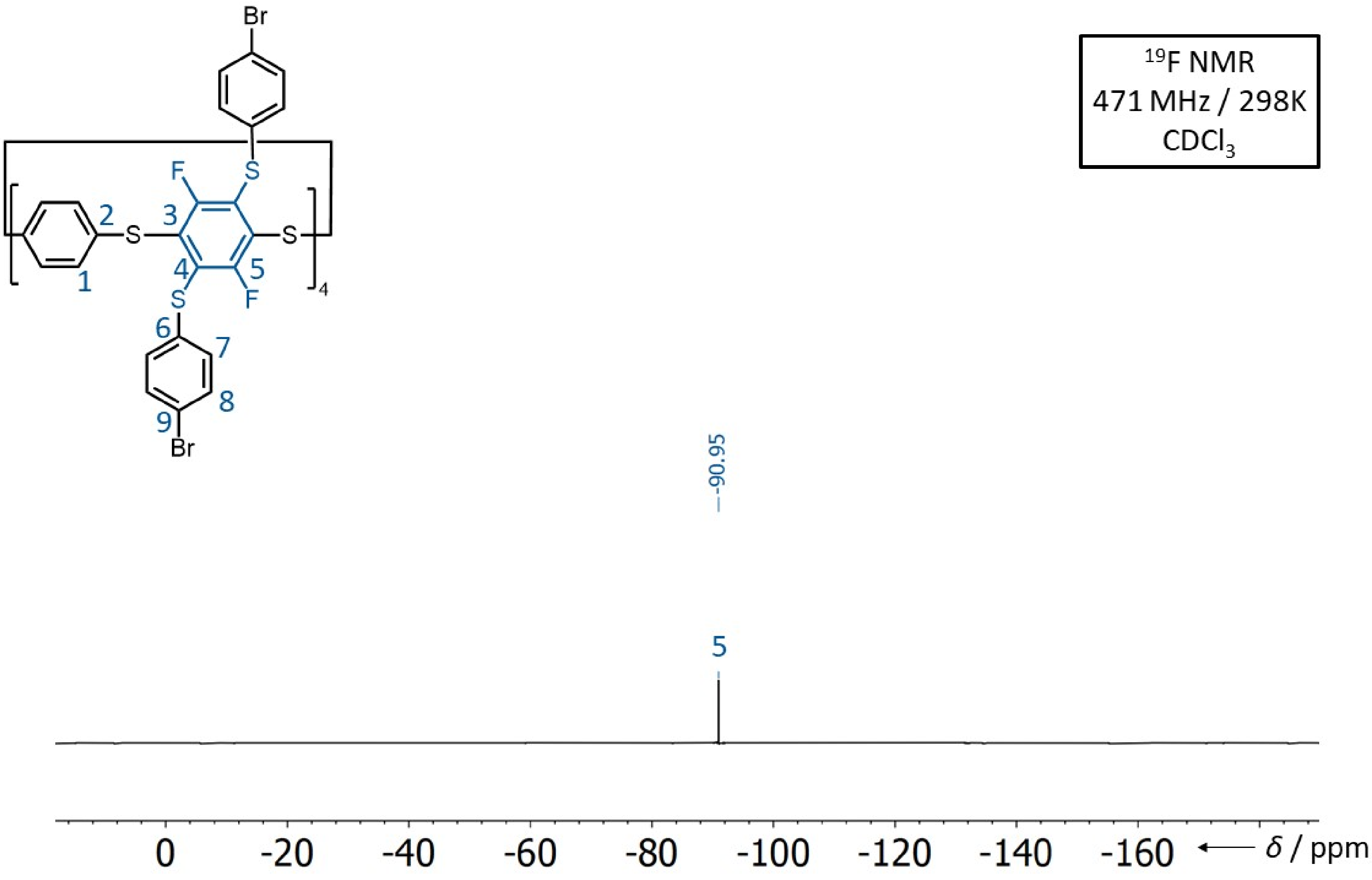

Figure S103. ^{19}F NMR Spectrum of **5c**.

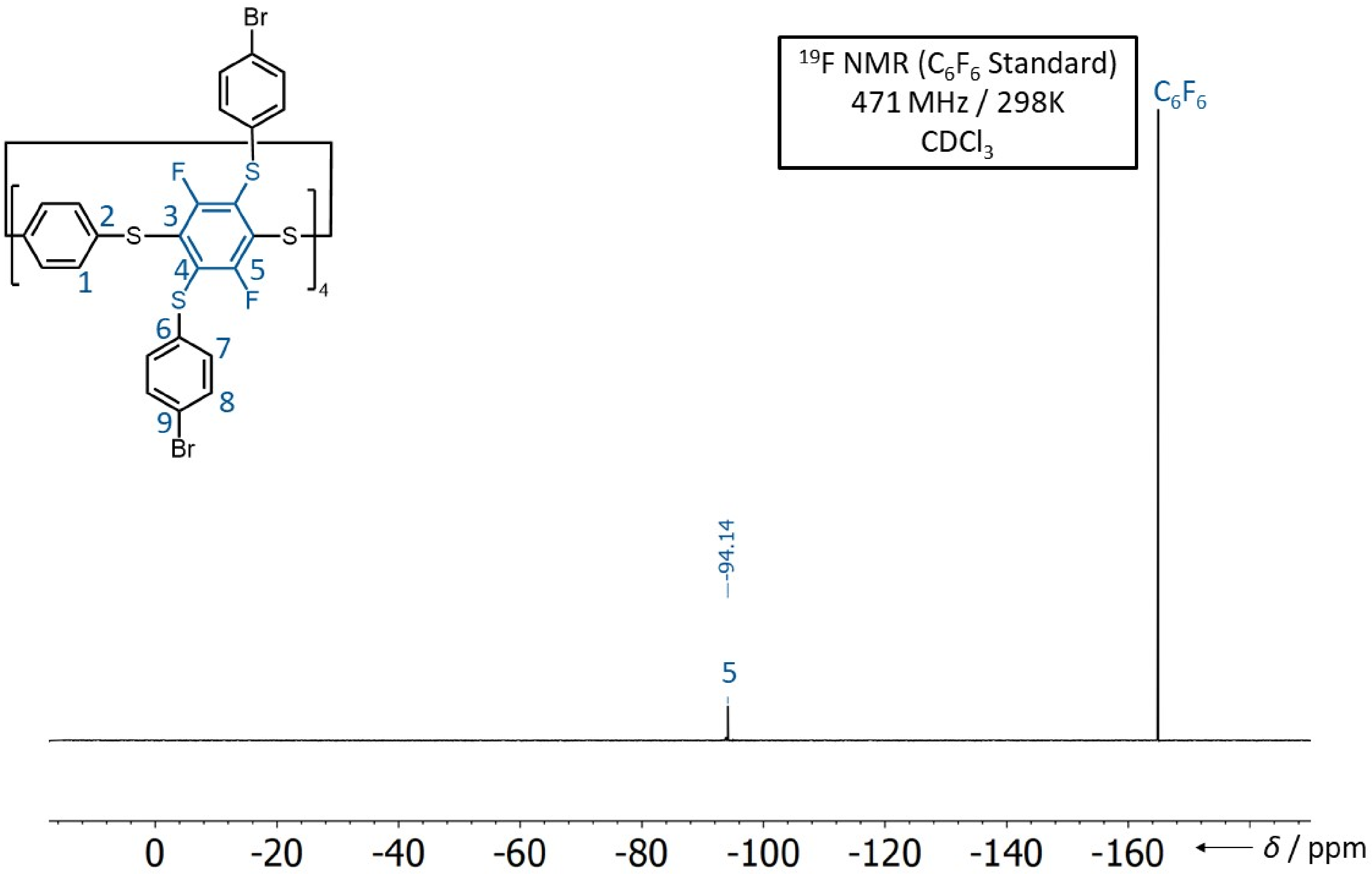

Figure S104. Referenced ^{19}F NMR Spectrum of **5c**.

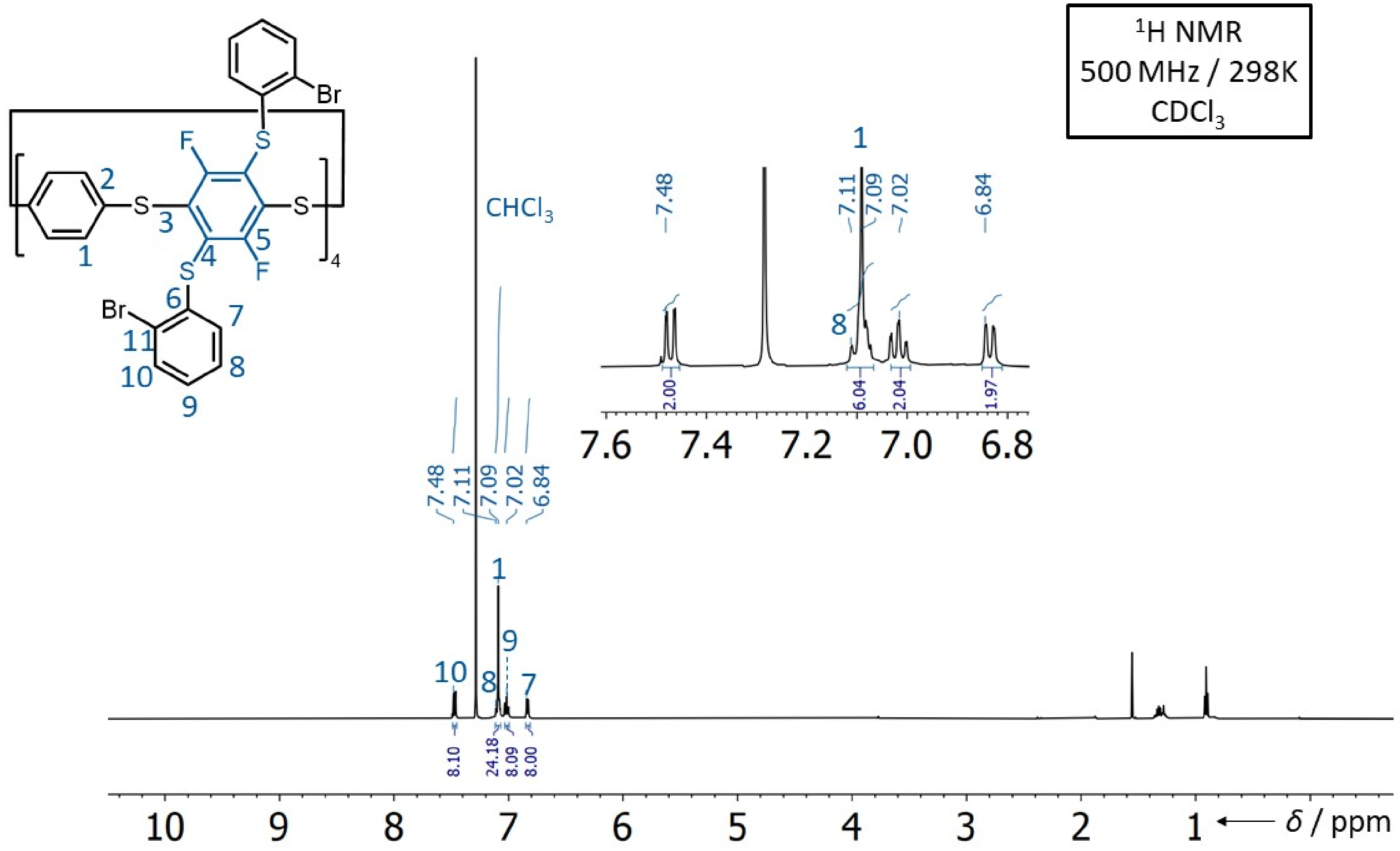

Figure S105. ^{1}H NMR Spectrum of **5d**.

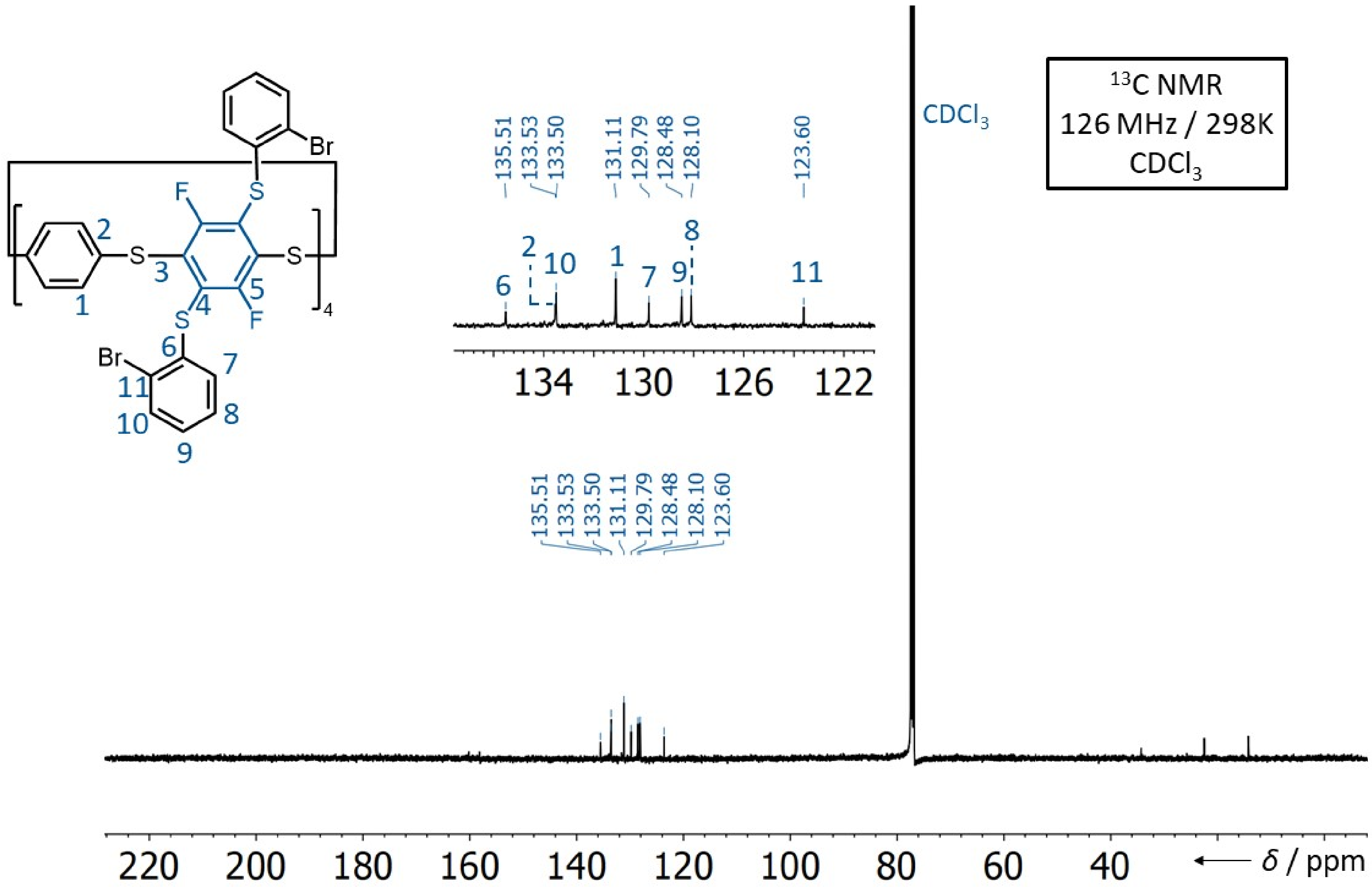

Figure S106. ^{13}C NMR Spectrum of **5d**.

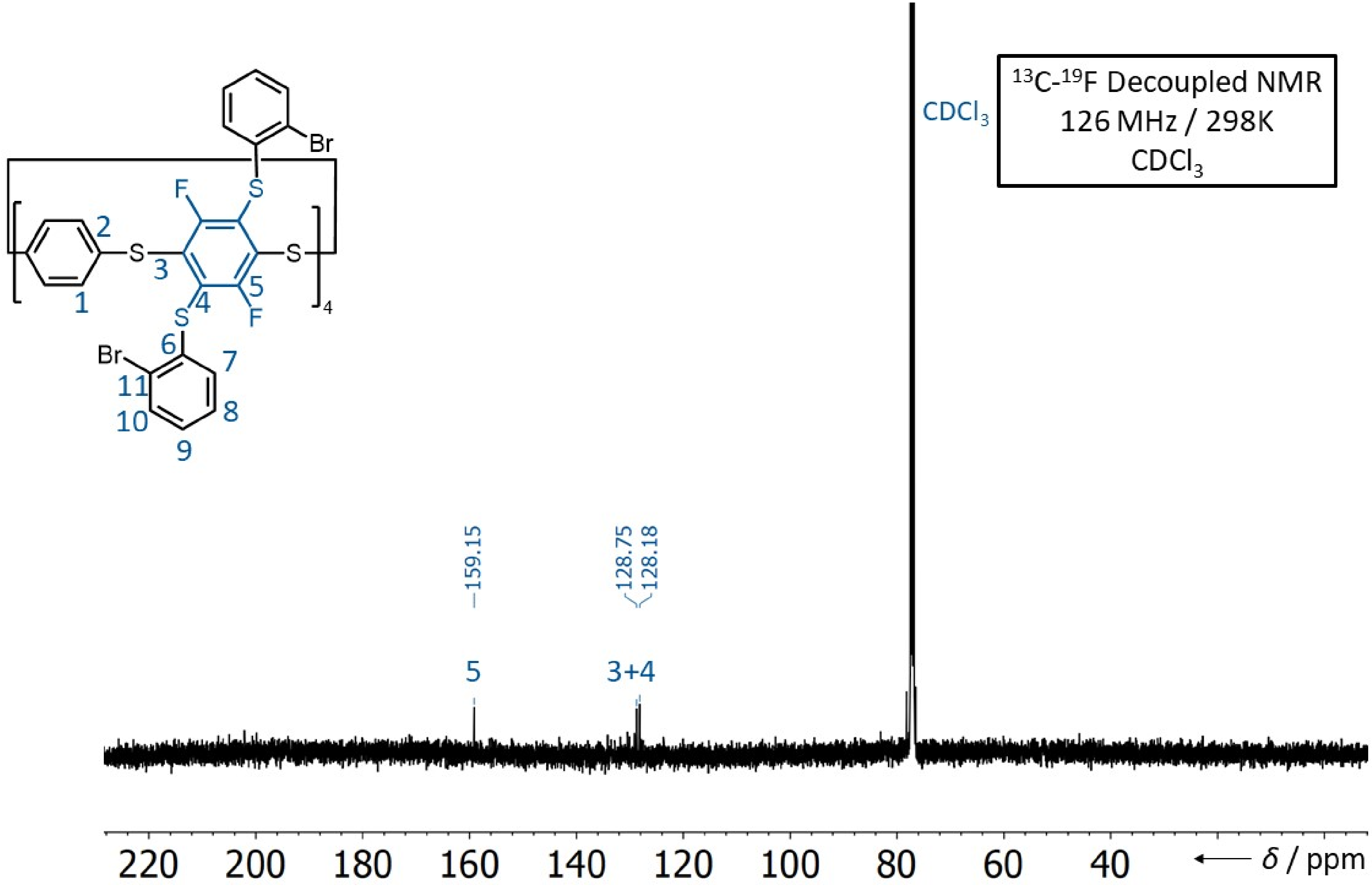

Figure S107. ^{13}C-^{19}F Decoupled NMR Spectrum of **5d**.

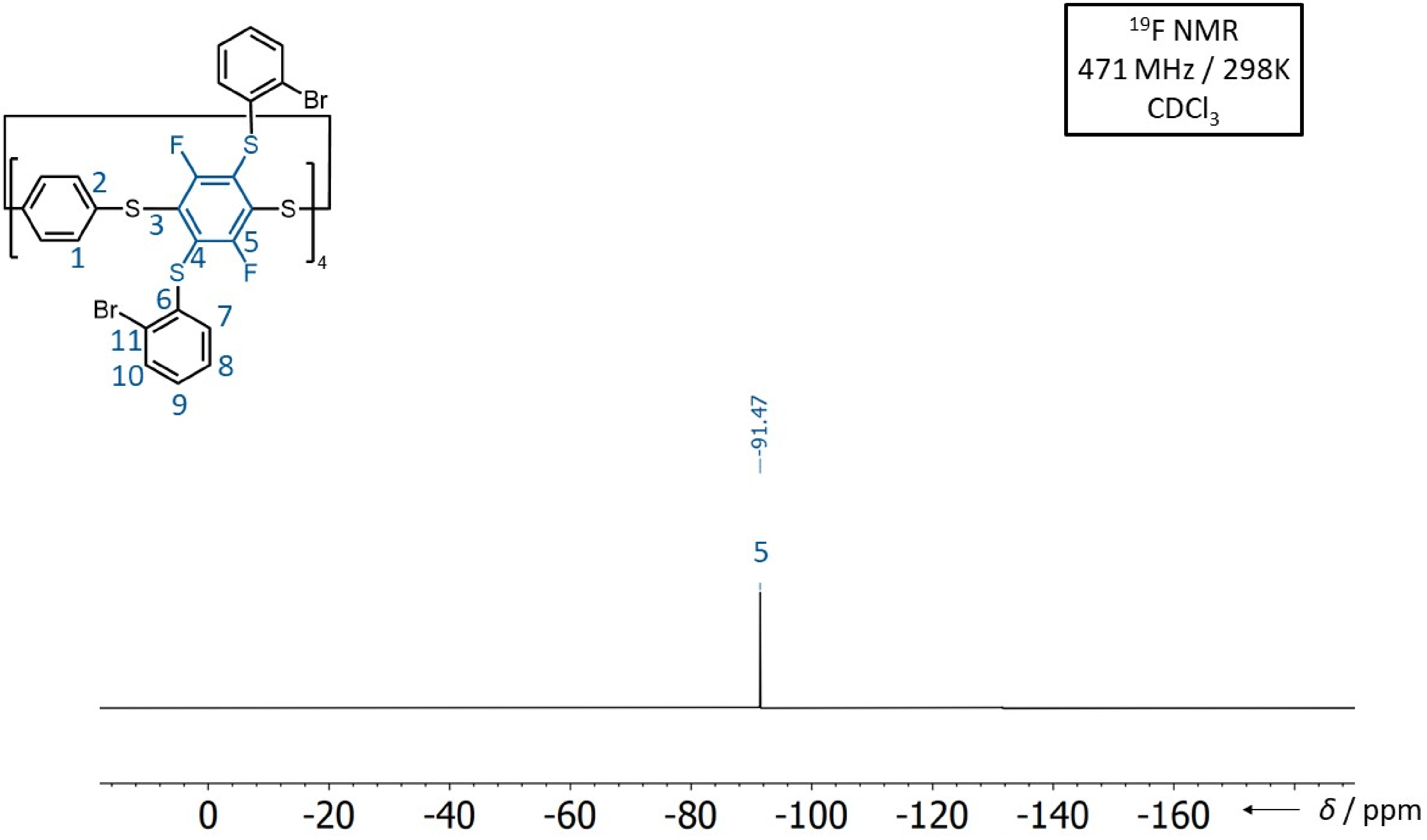

Figure S108. ^{19}F NMR Spectrum of **5d**.

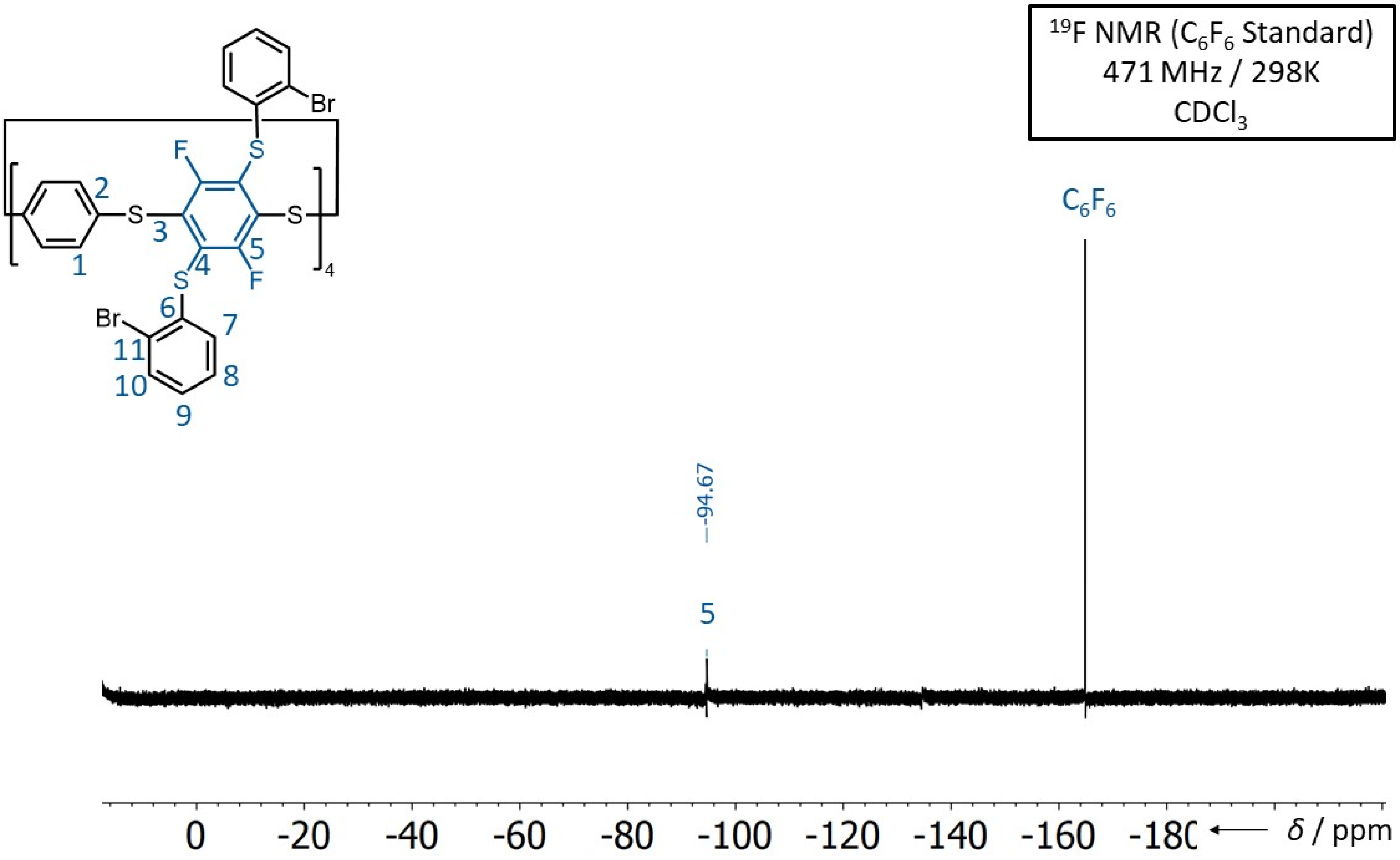

Figure S109. Referenced ^{19}F NMR Spectrum of **5d**.

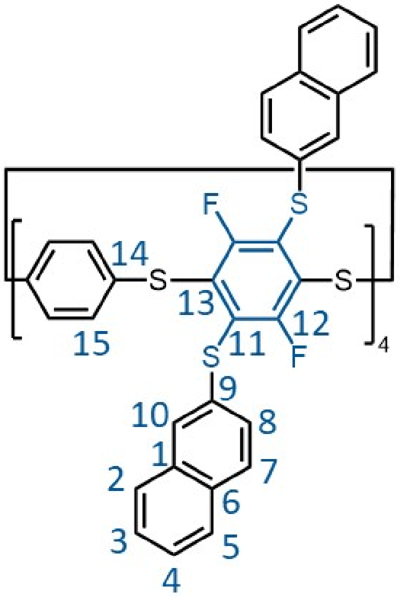

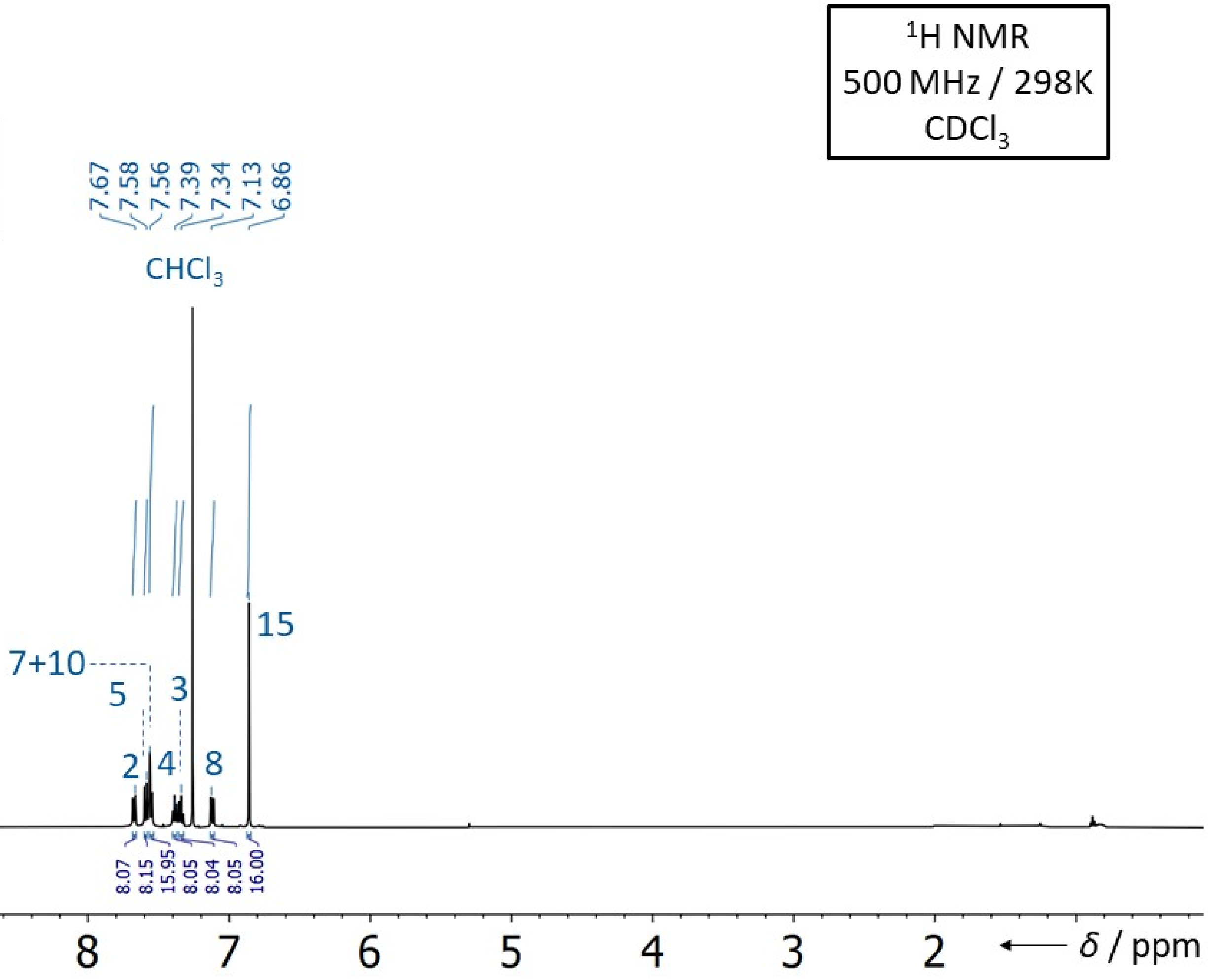

Figure S110. ^{1}H NMR Spectrum of **5e**.

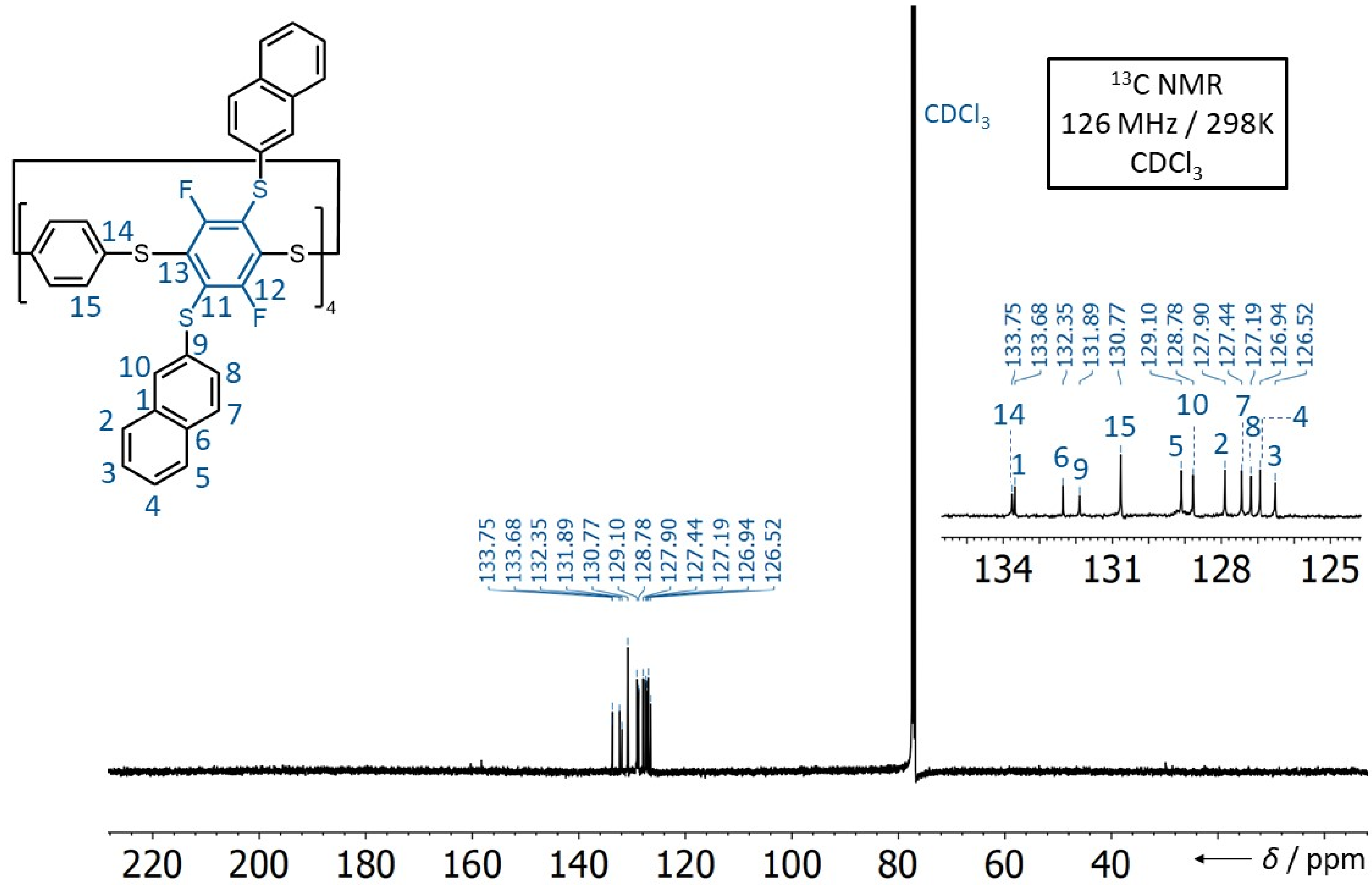

Figure S111. ^{13}C NMR Spectrum of **5e**.

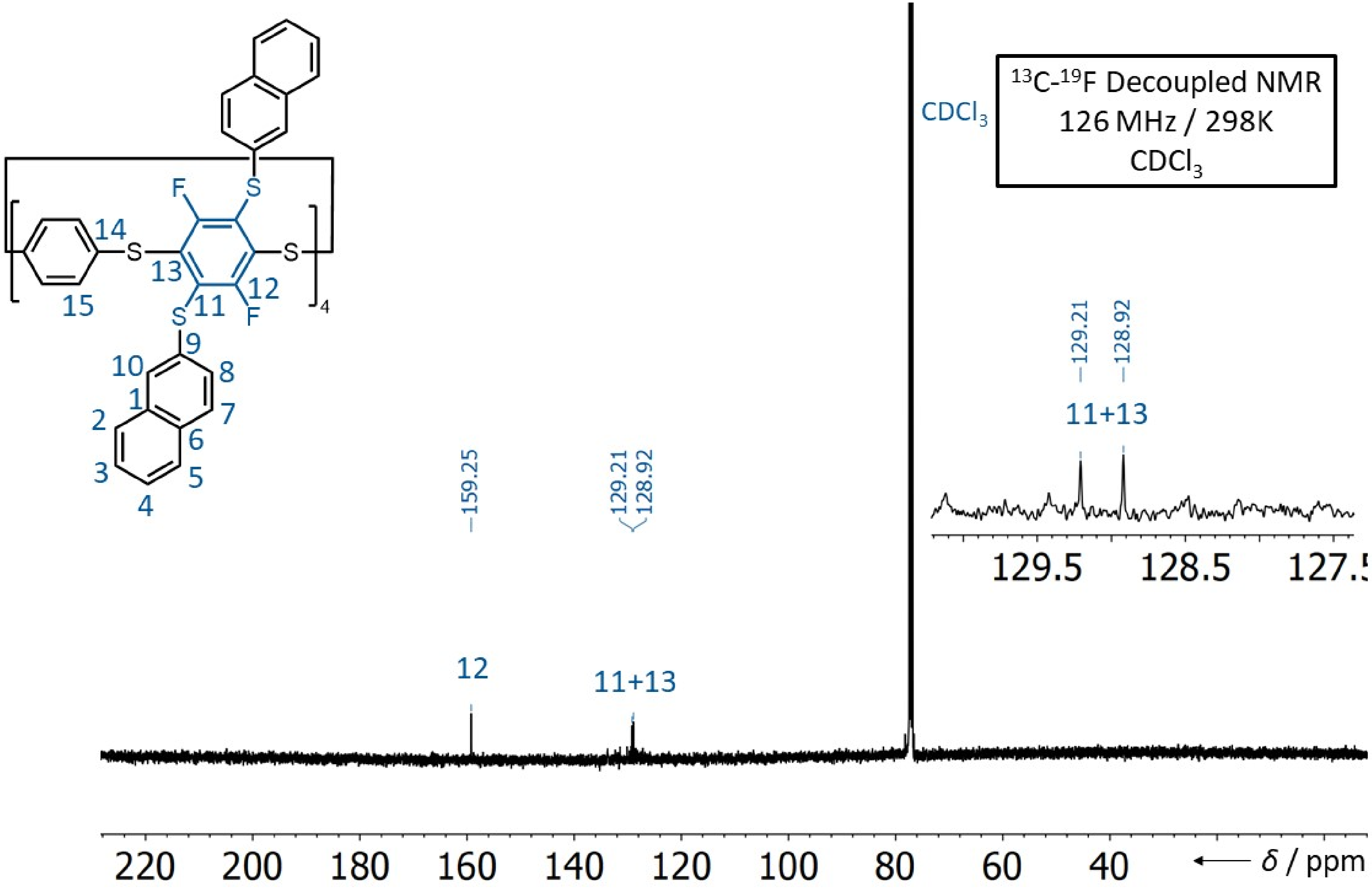

Figure S112. ^{13}C-^{19}F NMR Spectrum of **5e**.

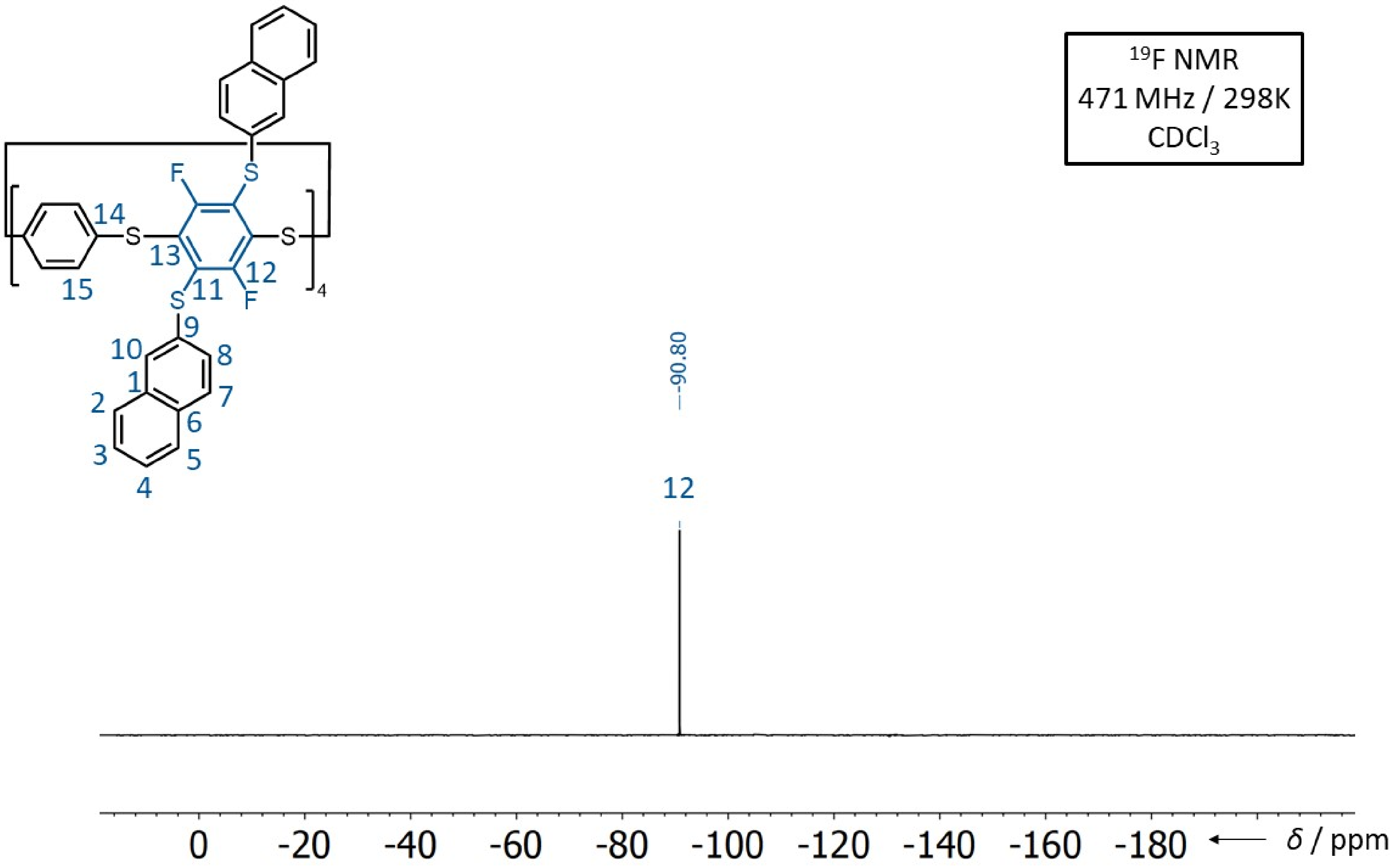

Figure S113. ^{19}F NMR Spectrum of **5e**.

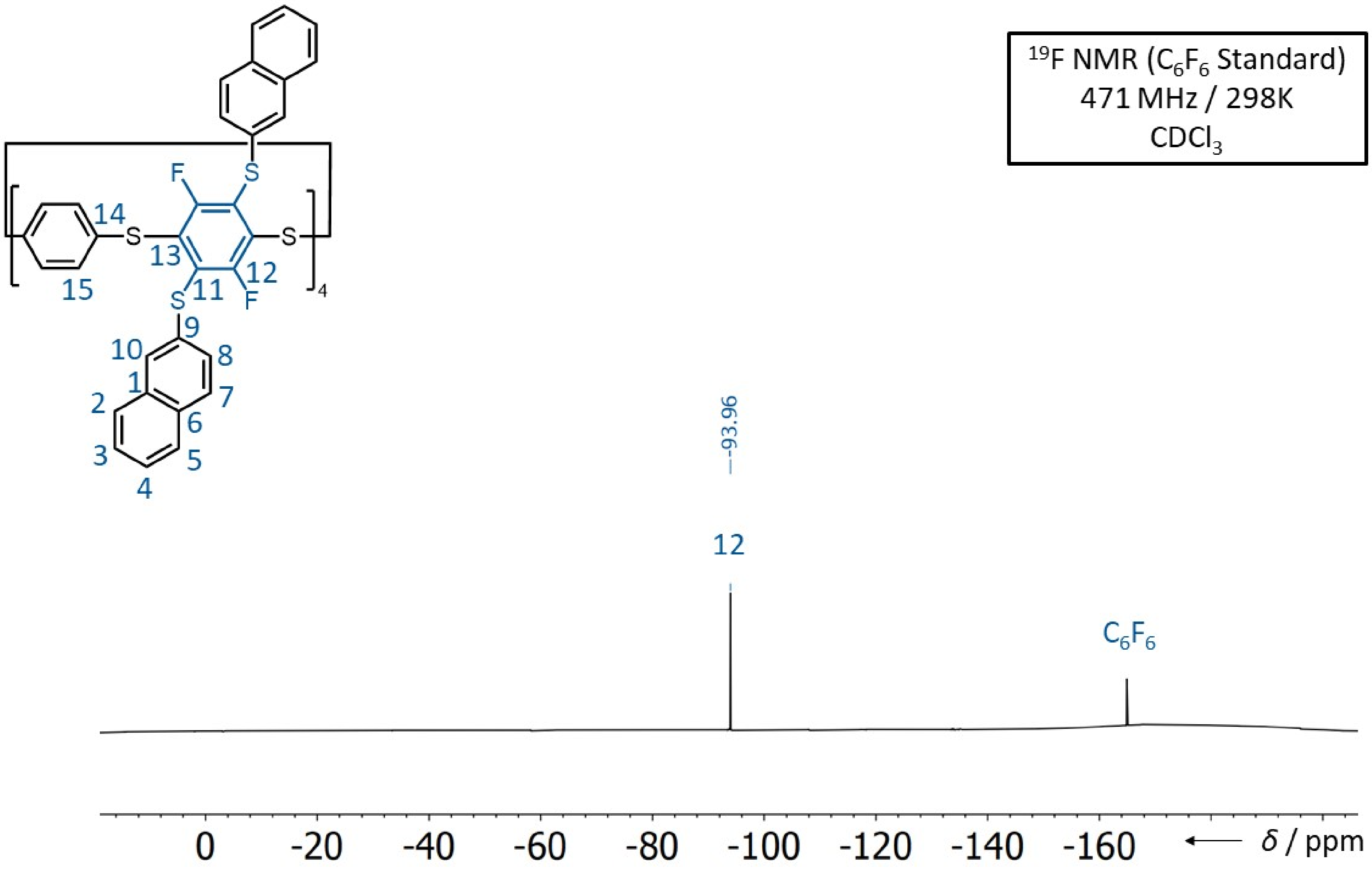

Figure S114. Referenced ^{19}F NMR Spectrum of **5e**.

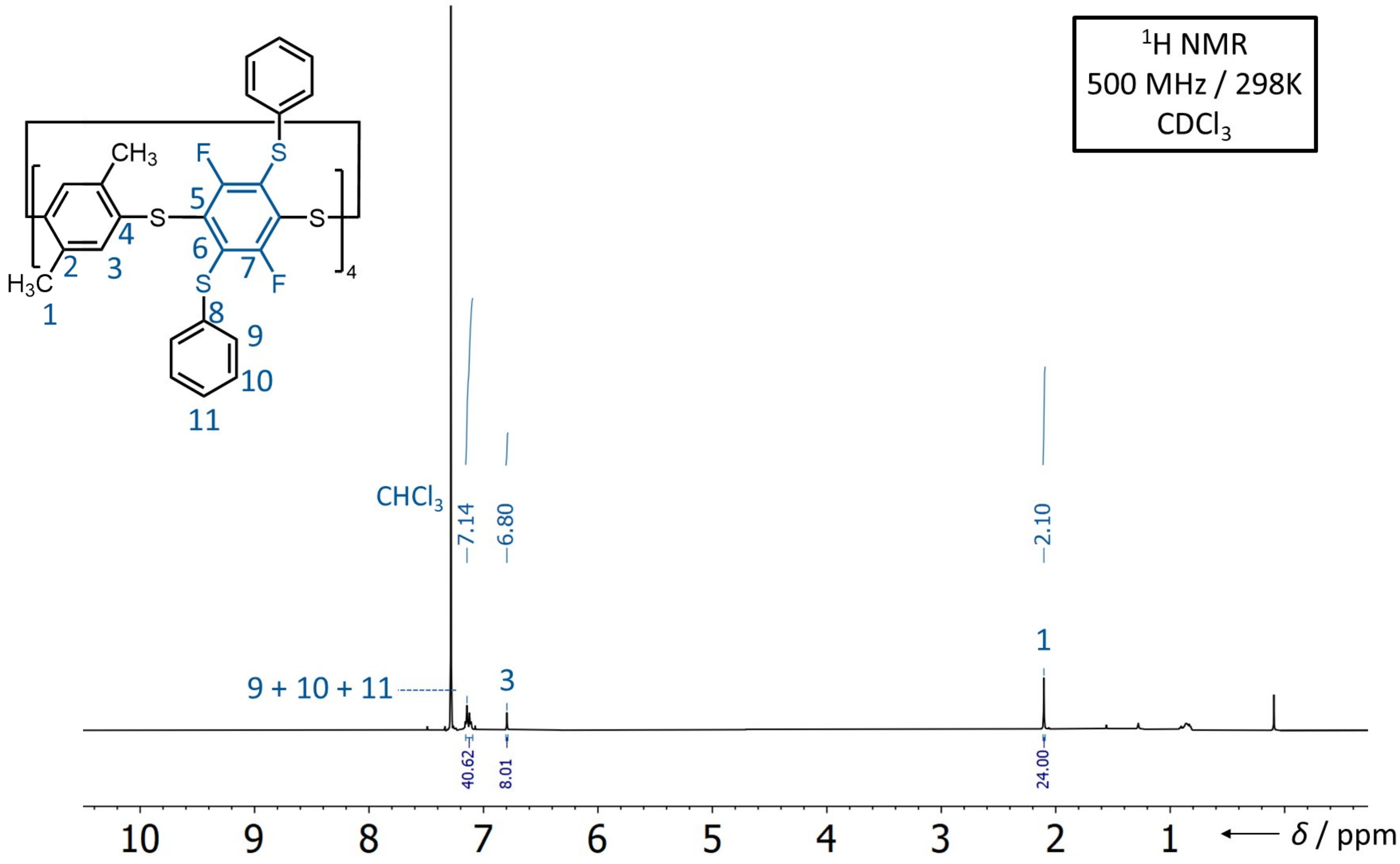

Figure S115. ^{1}H NMR Spectrum of **4b**.

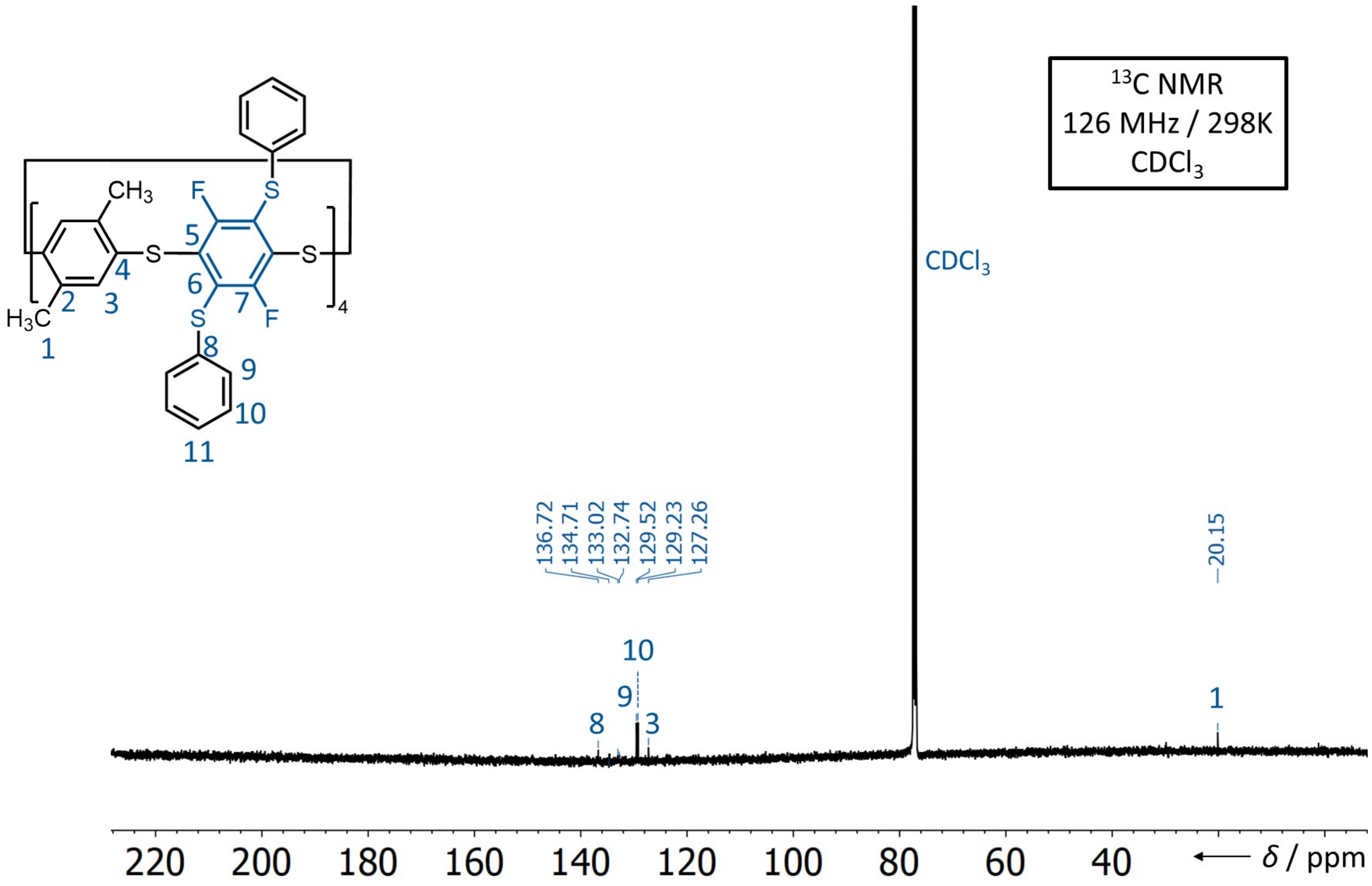

Figure S116. ^{13}C NMR Spectrum of **4b**.

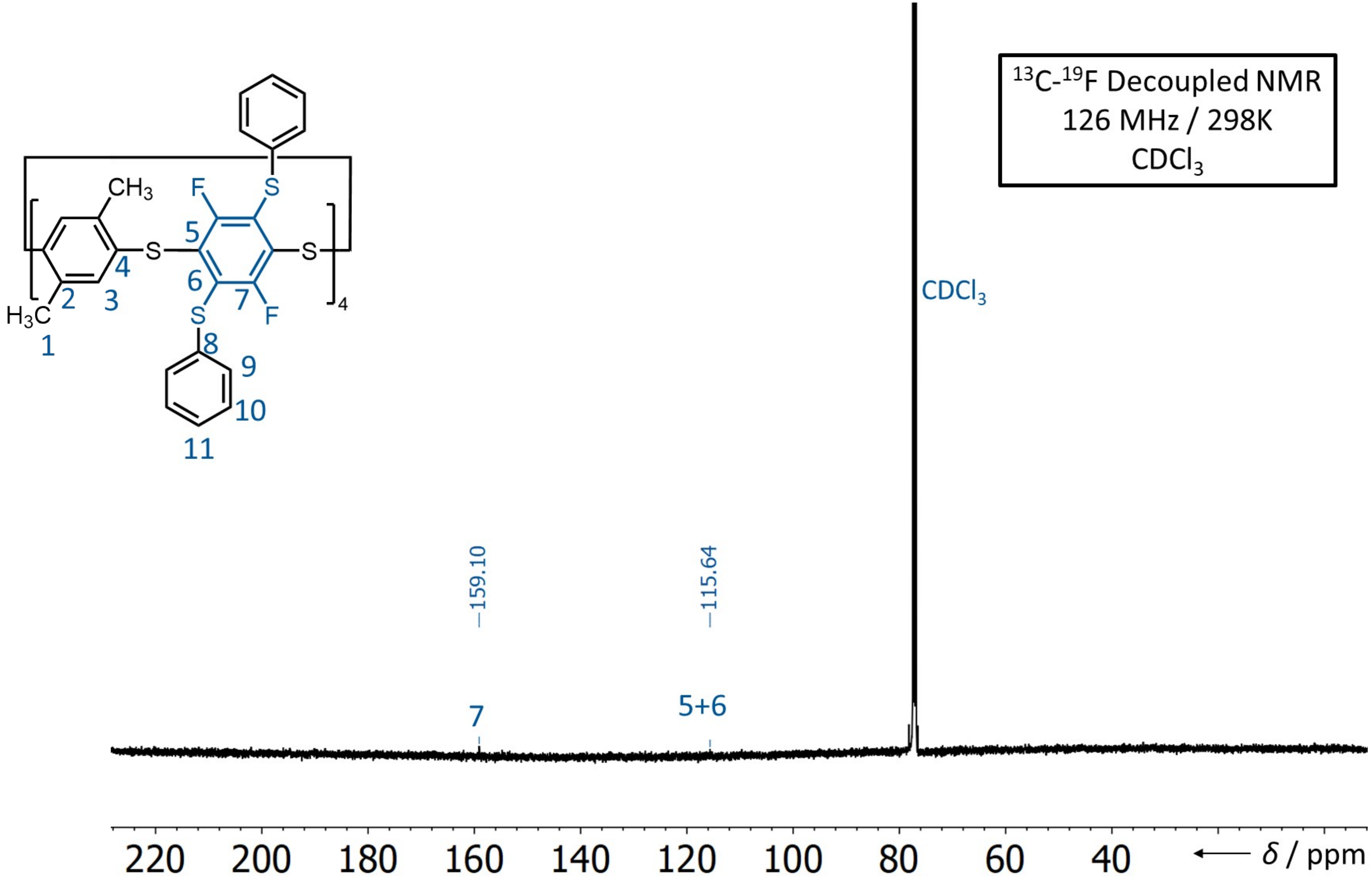

Figure S117. ^{13}C-^{19}F decoupled NMR Spectrum of **4b**.

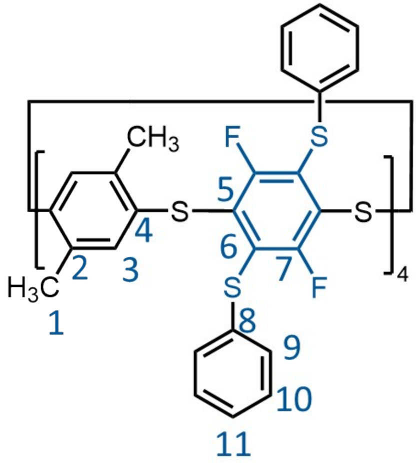

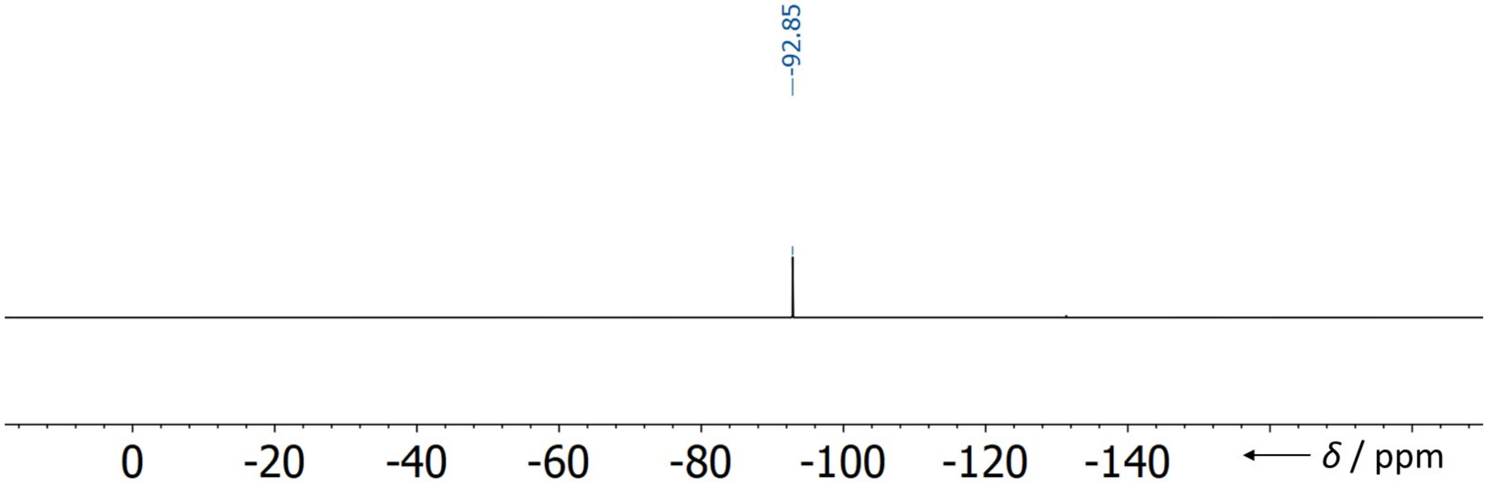

Figure S118. ^{19}F NMR Spectrum of **4b**.

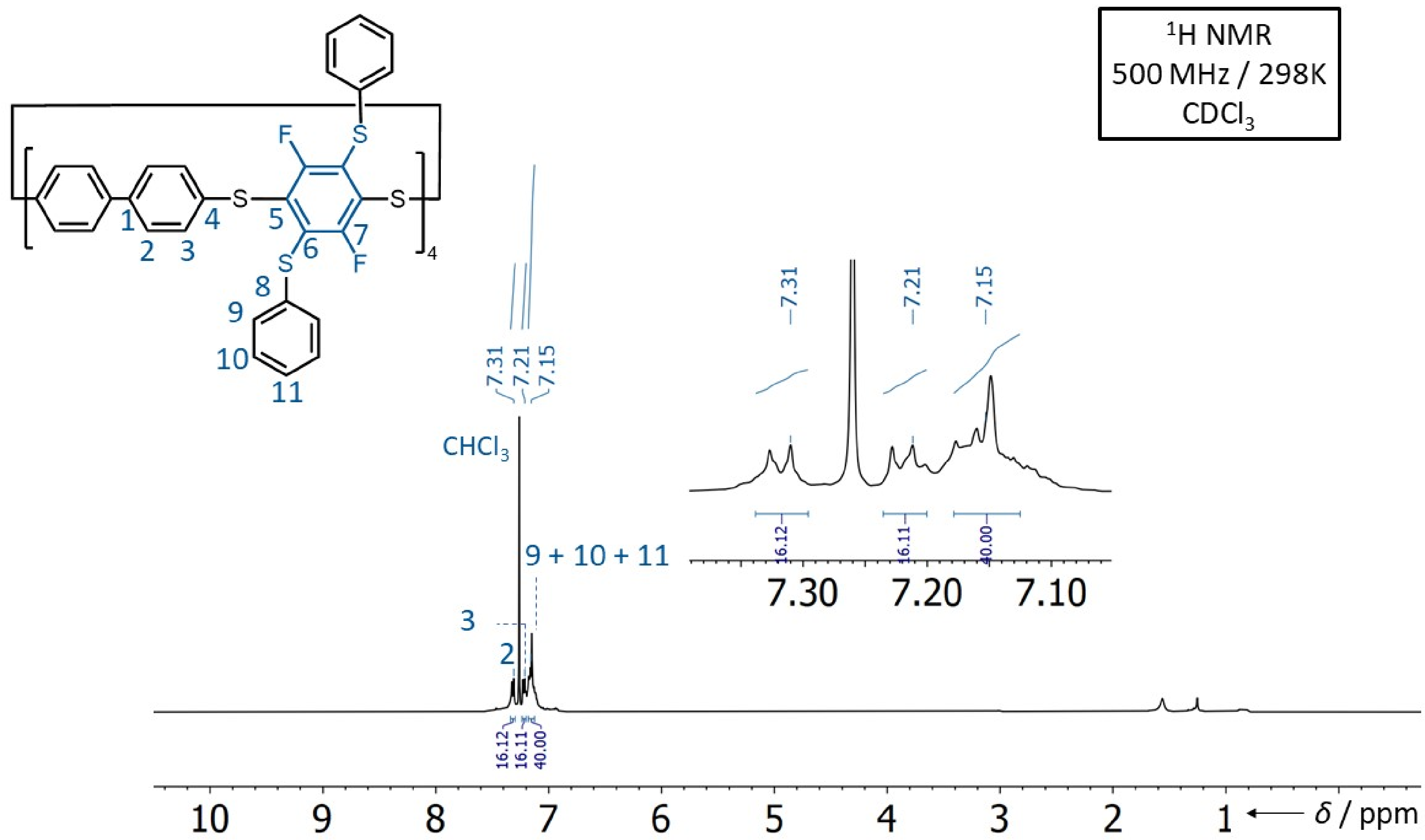

Figure S119. ^{1}H NMR Spectrum of **4d**.

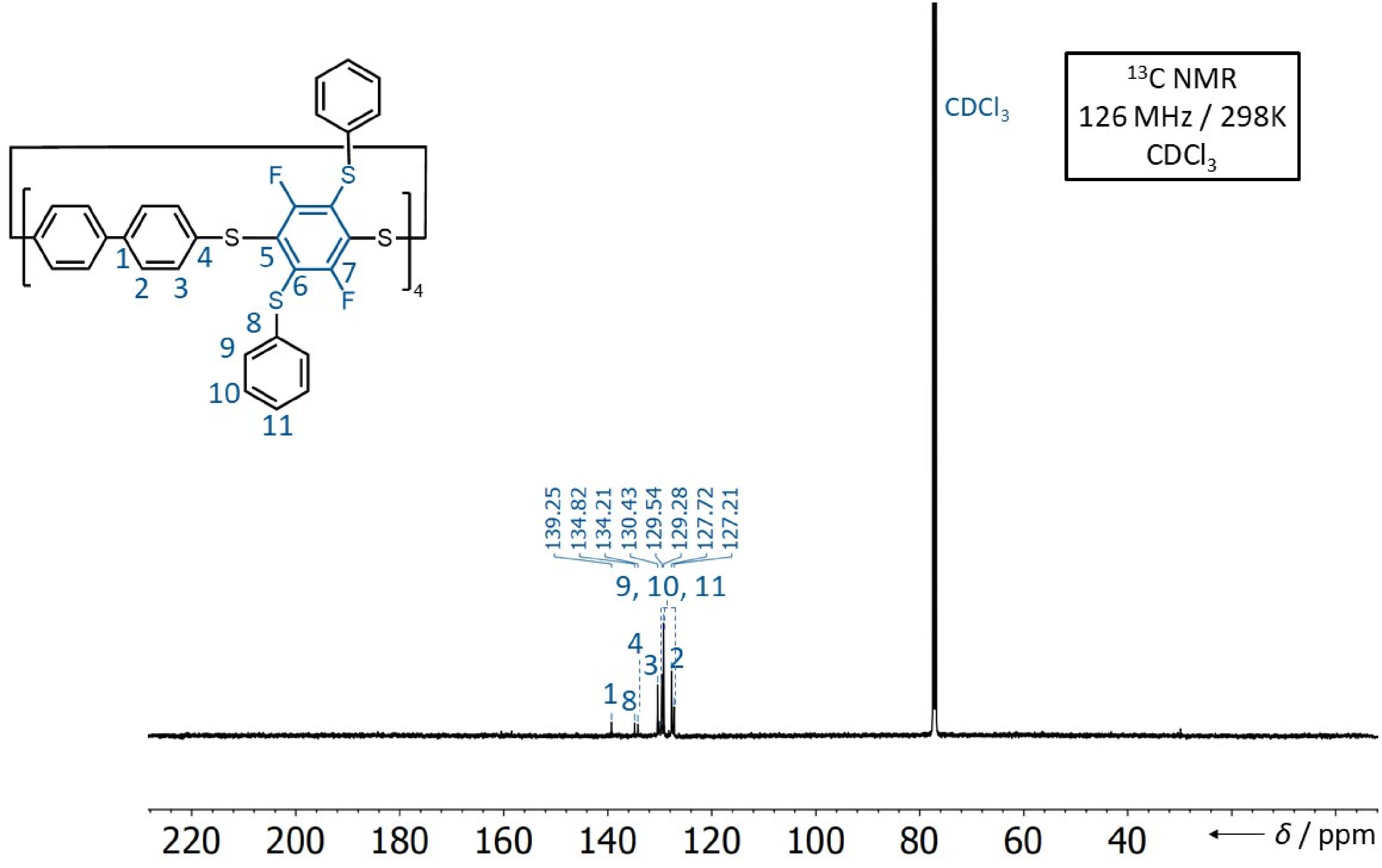

Figure S120. ^{13}C NMR Spectrum of **4d**.

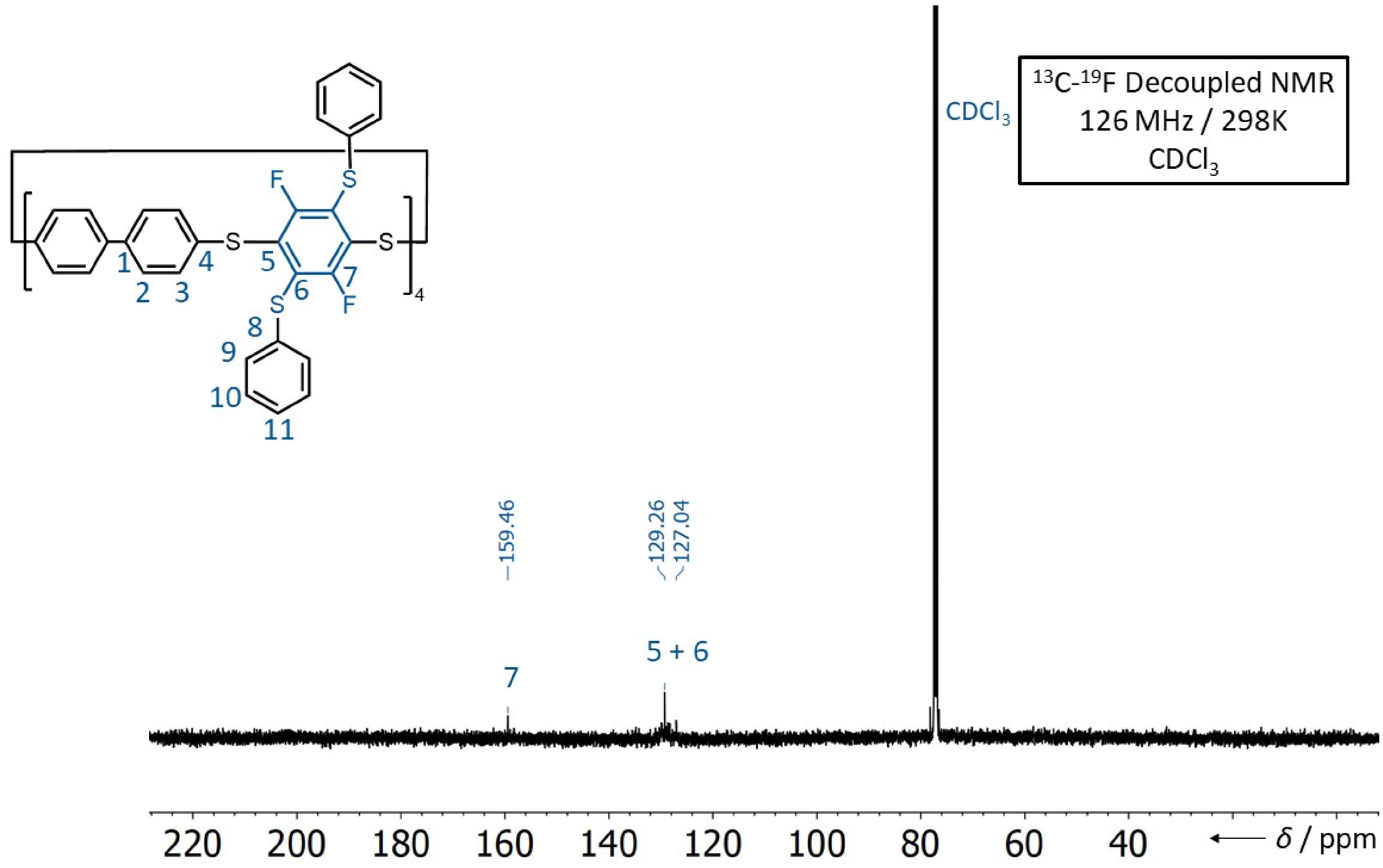

Figure S121. ^{13}C-^{19}F Decoupled NMR Spectrum of **4d**.

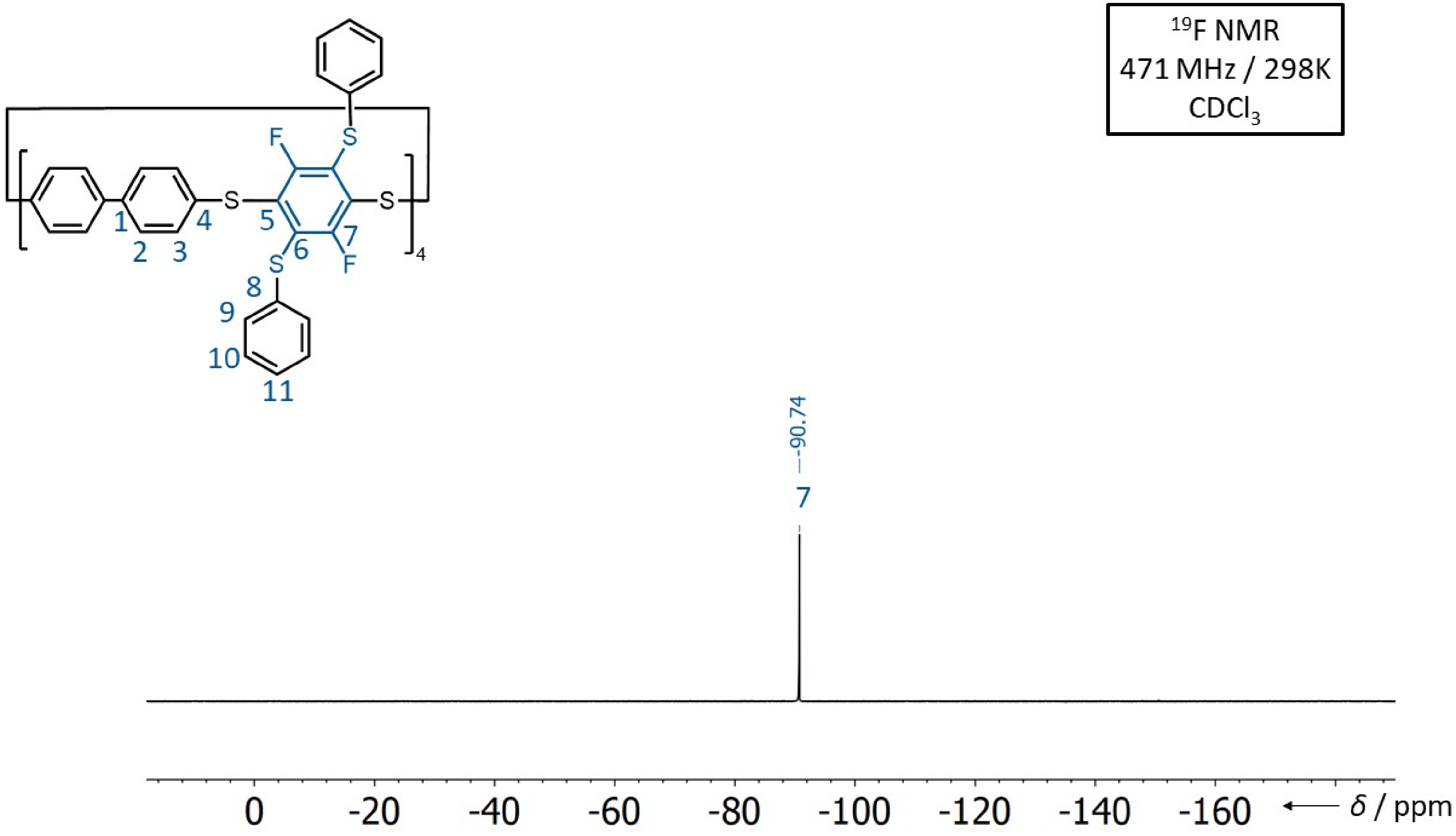

Figure S122. ^{19}F NMR Spectrum of **4d**.

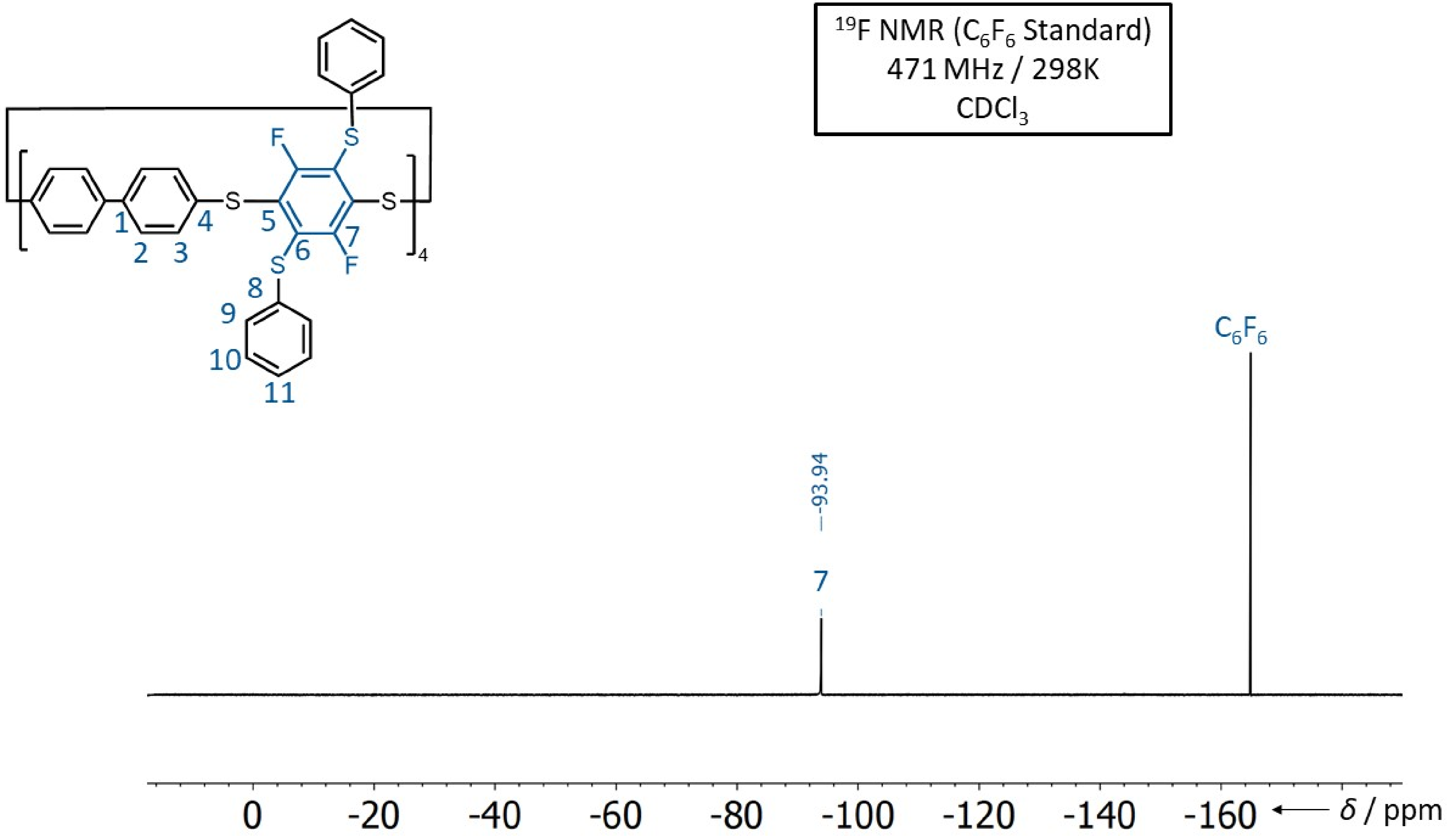

Figure S123. Referenced ^{19}F NMR Spectrum of **4d**.

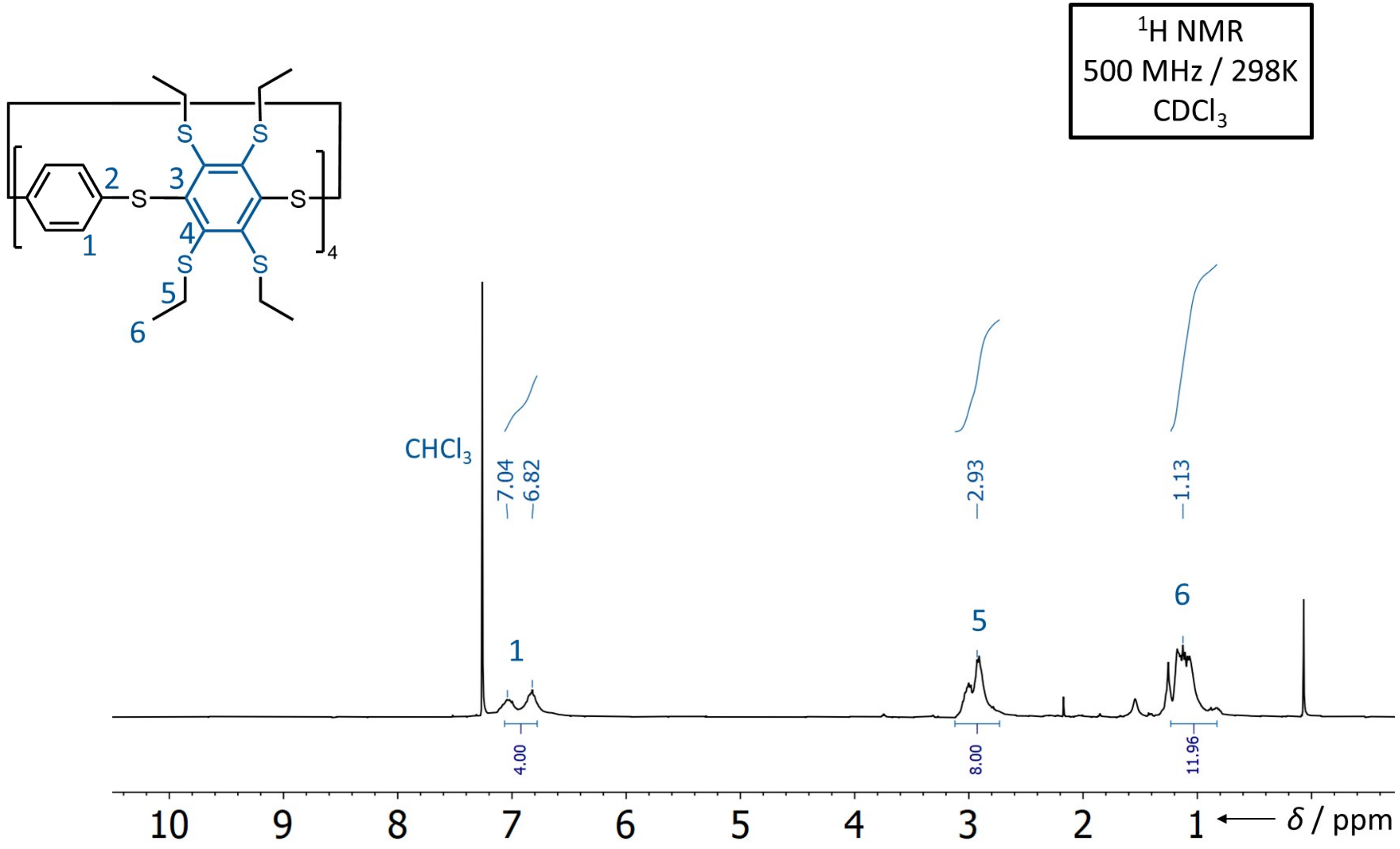

Figure S124. ^{1}H NMR Spectrum of **6a**.

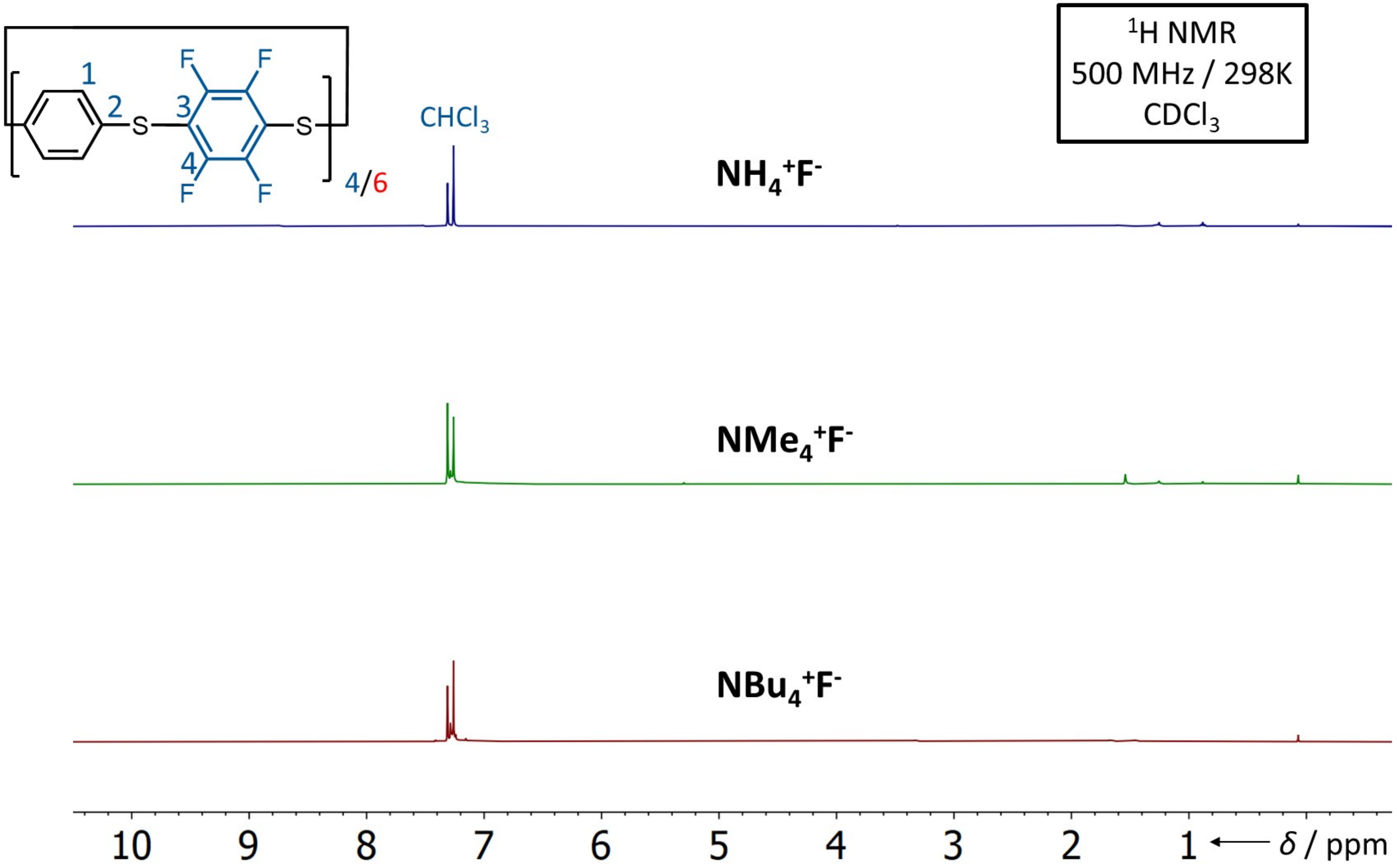

3.1. ^{1}H and ^{19}F NMR Characterisation of 1a and 1a' from Various Templates

Figure S125. ^{1}H NMR Spectra of **1a** synthesised from various ammonium fluoride templates: $NH_4{+}F^-$, $NMe_4^+F^-$ and $NBu_4^+F^-$. Blue numbering = S_8-corona[8]arene **1a** and Red numbering = S_{12}-corona[12]arene **1a'**.

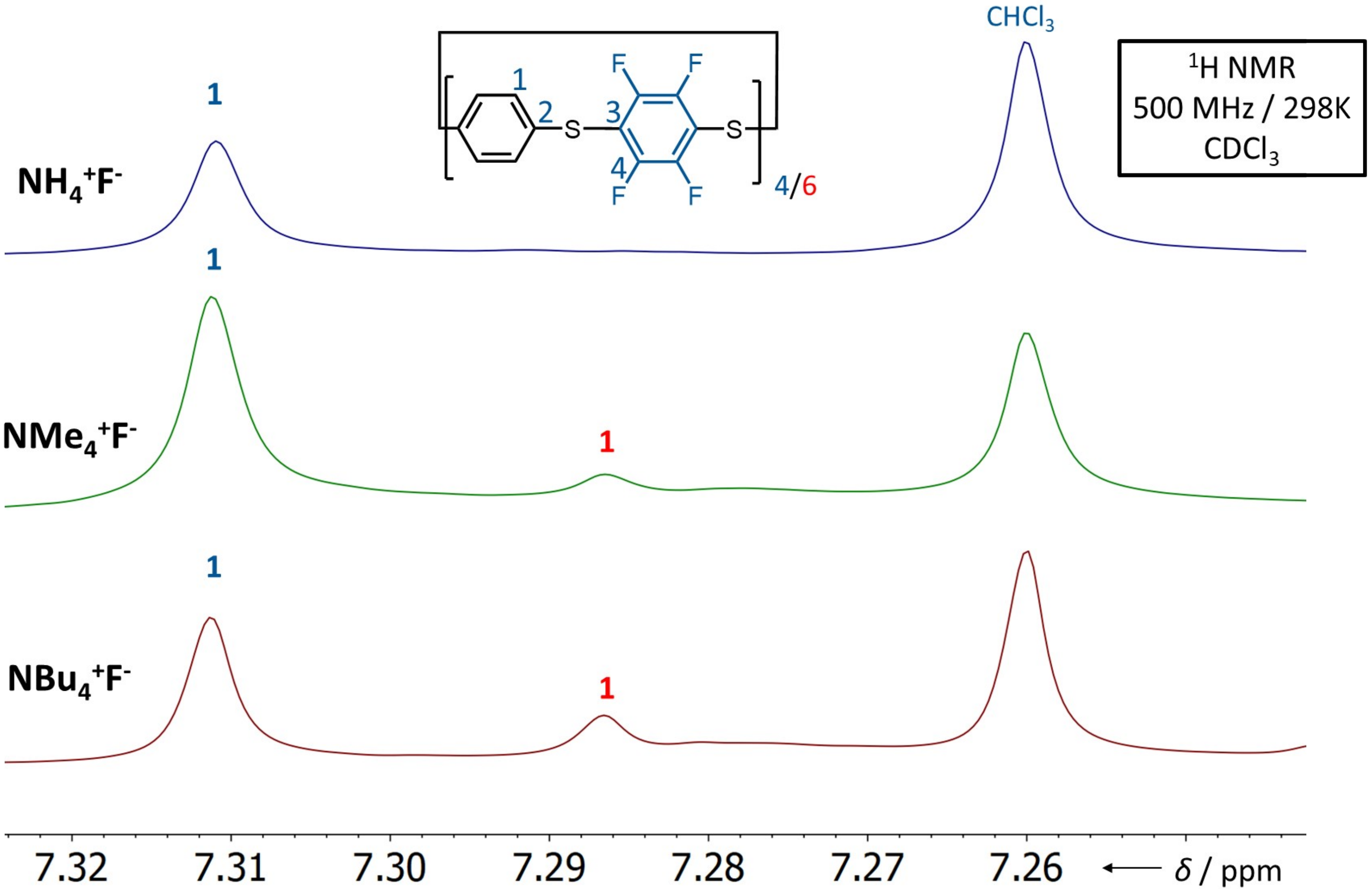

Figure S126. Partial ^{1}H NMR Spectra of **1a** synthesised from various ammonium fluoride templates: $NH_4^+F^-$, $NMe_4^+F^-$ and $NBu_4^+F^-$. Blue numbering = S_8-corona[8]arene **1a** and Red numbering = S_{12}-corona[12]arene **1a'**.

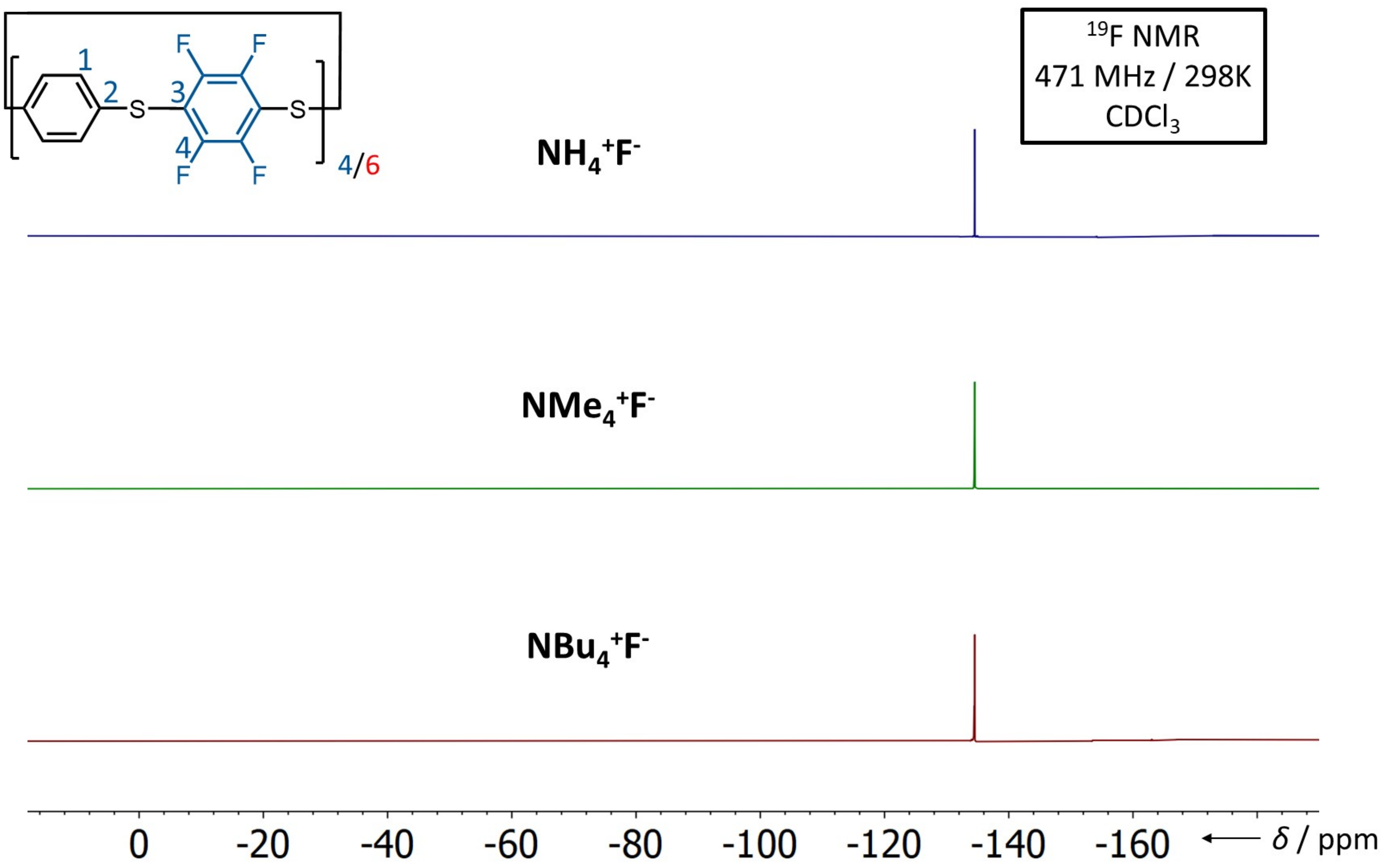

Figure S127. ^{19}F NMR Spectra of **1a** synthesised from various ammonium fluoride templates: $NH_4^+F^-$, $NMe_4^+F^-$ and $NBu_4^+F^-$. Blue numbering = S_8-corona[8]arene **1a** and Red numbering = S_{12}-corona[12]arene **1a’**.

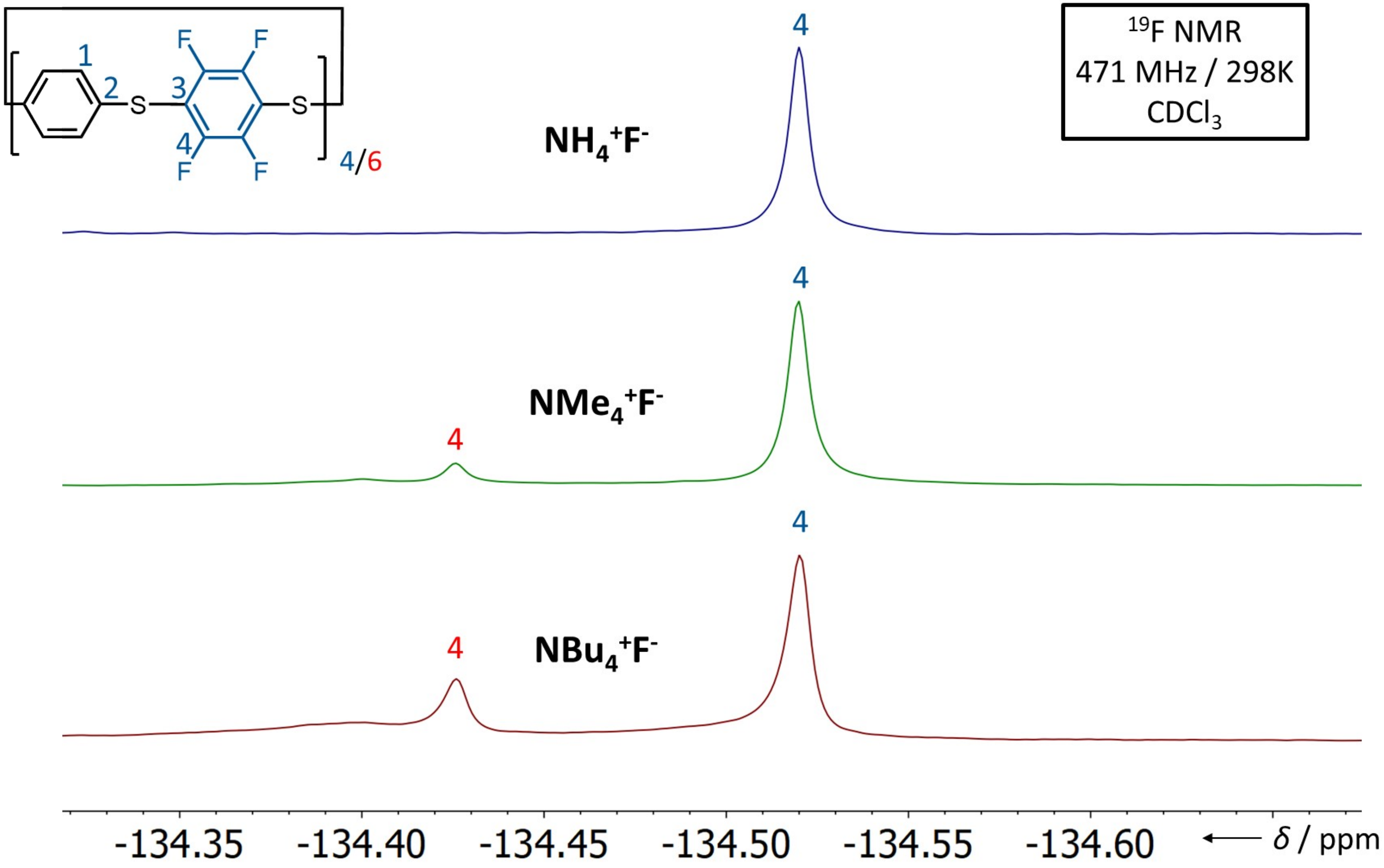

Figure S128. ^{19}F NMR Spectra of **1a** synthesised from various ammonium fluoride templates: $NH_4^+F^-$, $NMe_4^+F^-$ and $NBu_4^+F^-$. Blue numbering = S_8-corona[8]arene **1a** and Red numbering = S_{12}-corona[12]arene **1a'**.

3.2. ^{1}H NMR Reaction Monitoring

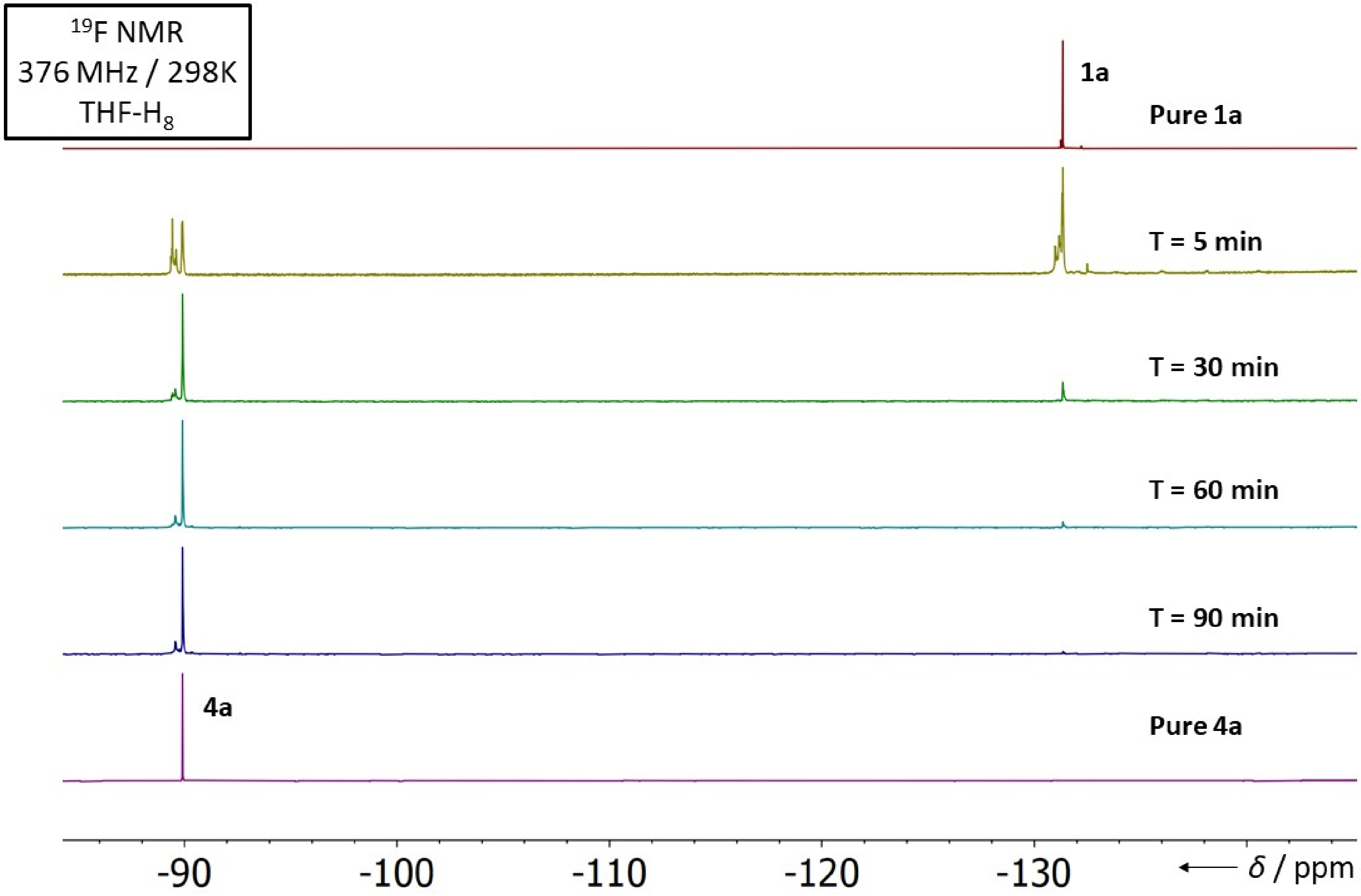

Figure S129. ^{1}H NMR stack of the formation of **4a** from **1a** over time.

3.3. ¹H / DOSY NMR

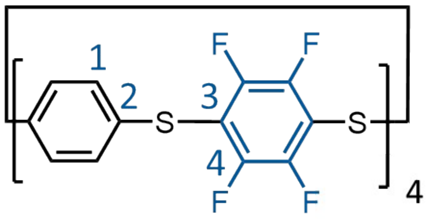

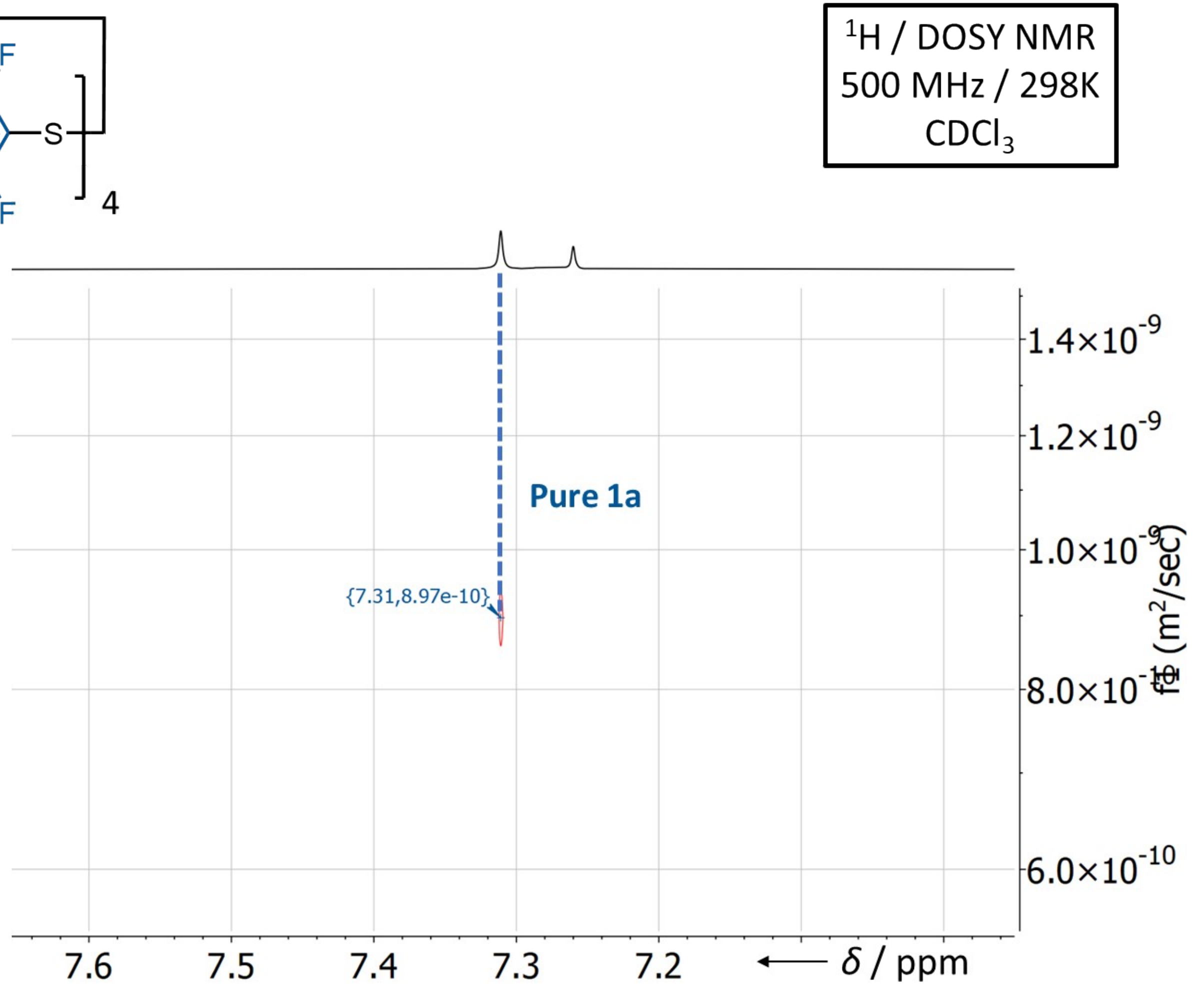

Figure S130. DOSY NMR Plot of **1a**. The y-axis indicates diffusion coefficient in m²/sec.

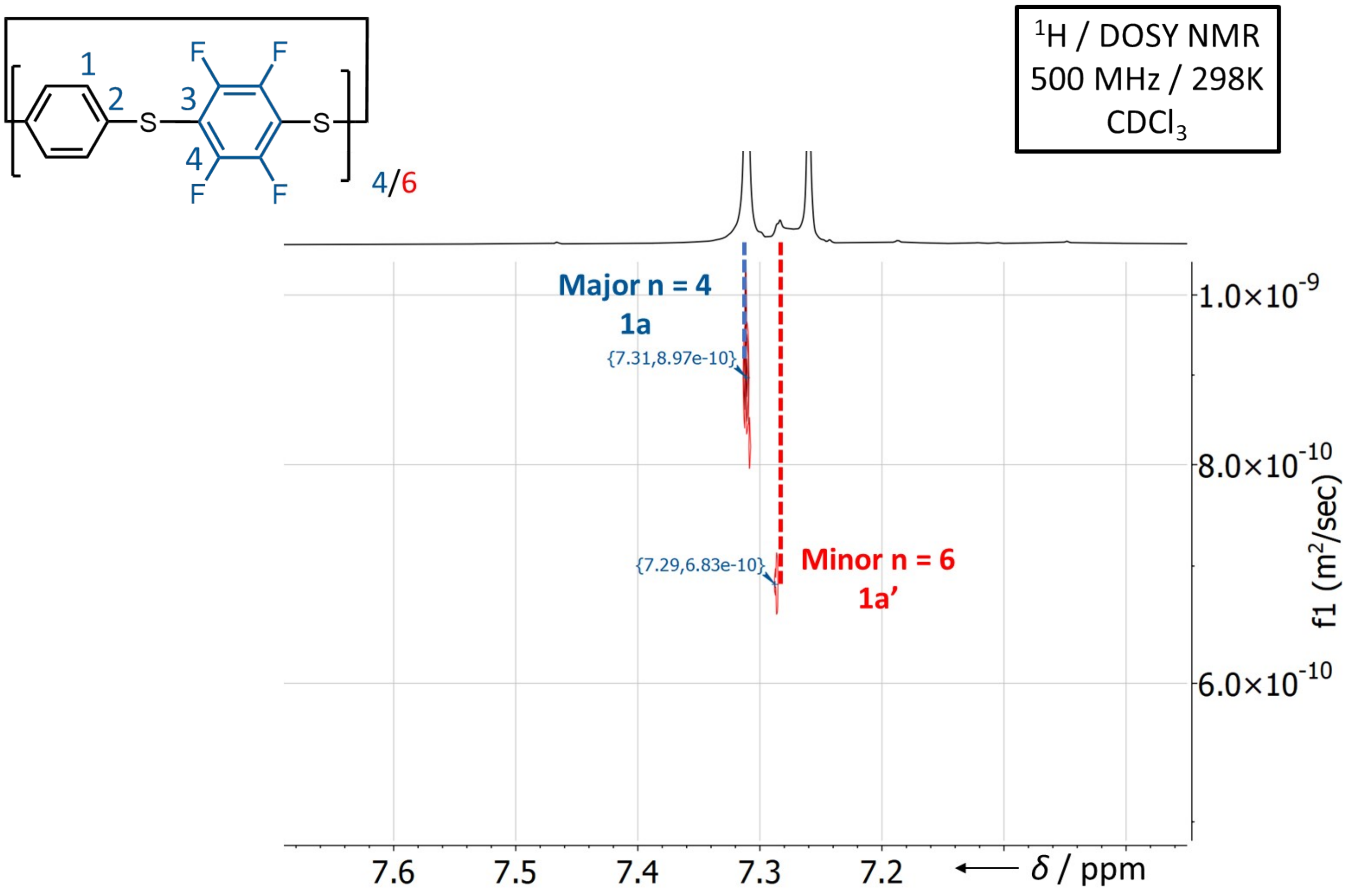

Figure S131. DOSY NMR Plot of **1a** and **1a’**. The y-axis indicates diffusion coefficient in m^2/sec.

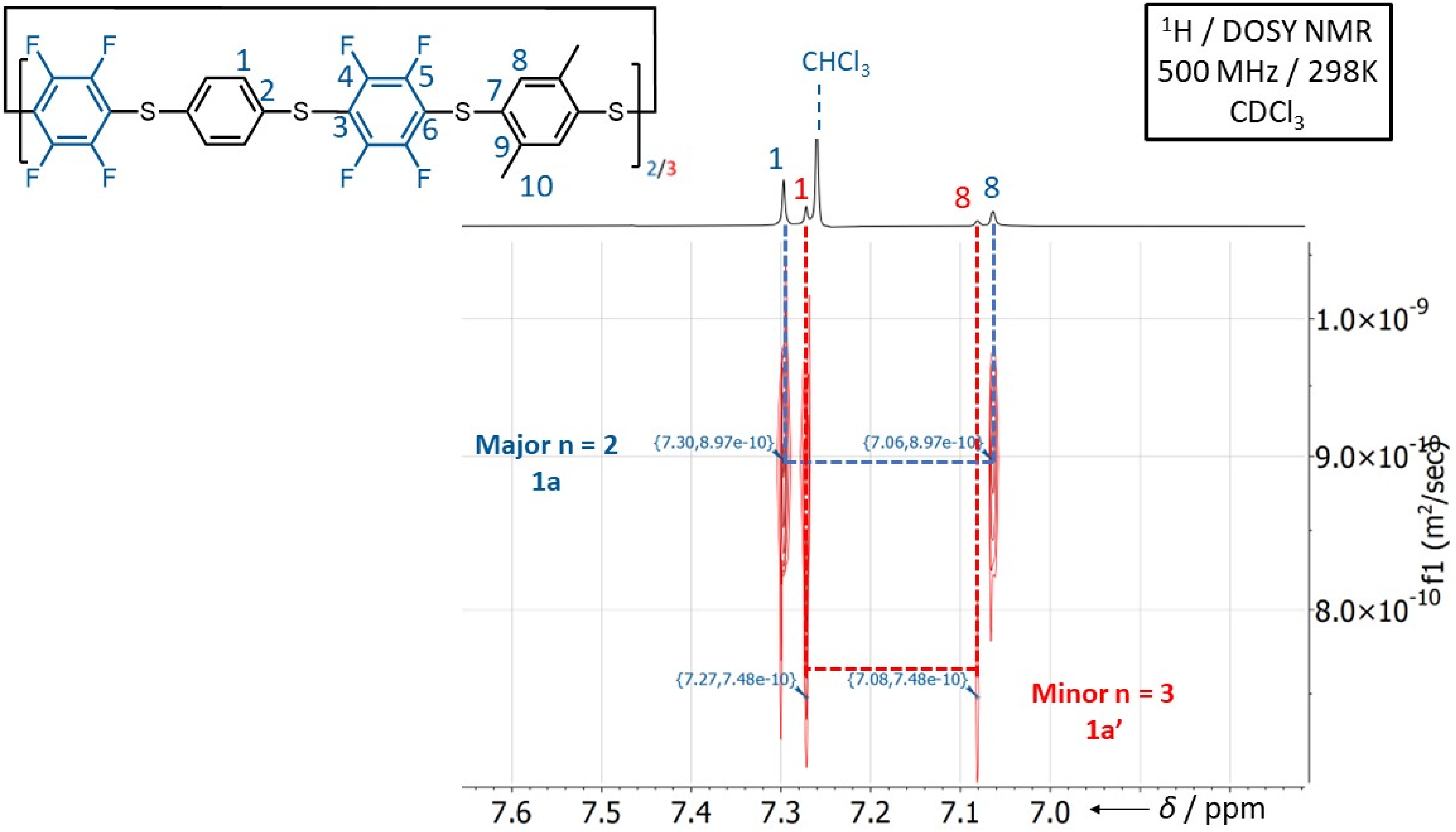

Figure S132. DOSY NMR Plot of **1ab** (**n = 2**) and **1ab** (**n = 3**). The y-axis indicates diffusion coefficient in m^2/sec.

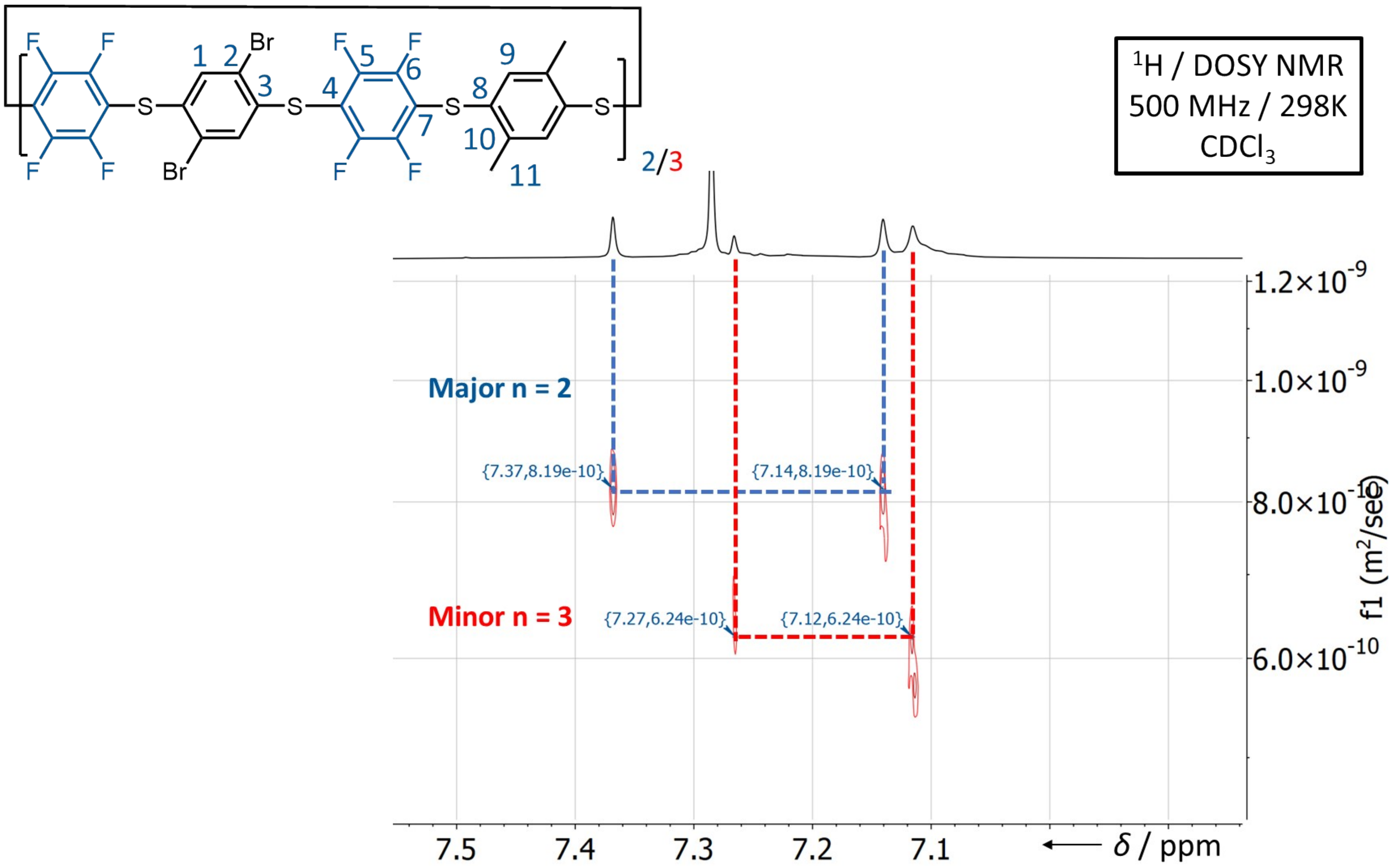

Figure S133. DOSY NMR Plot of **1bc** (**n = 2**) and **1bc'** (**n = 3**). The y-axis indicates diffusion coefficient in m^2/sec.

4. Matrix-assisted Laser Desorption/Ionisation TOF Mass Spectrometry

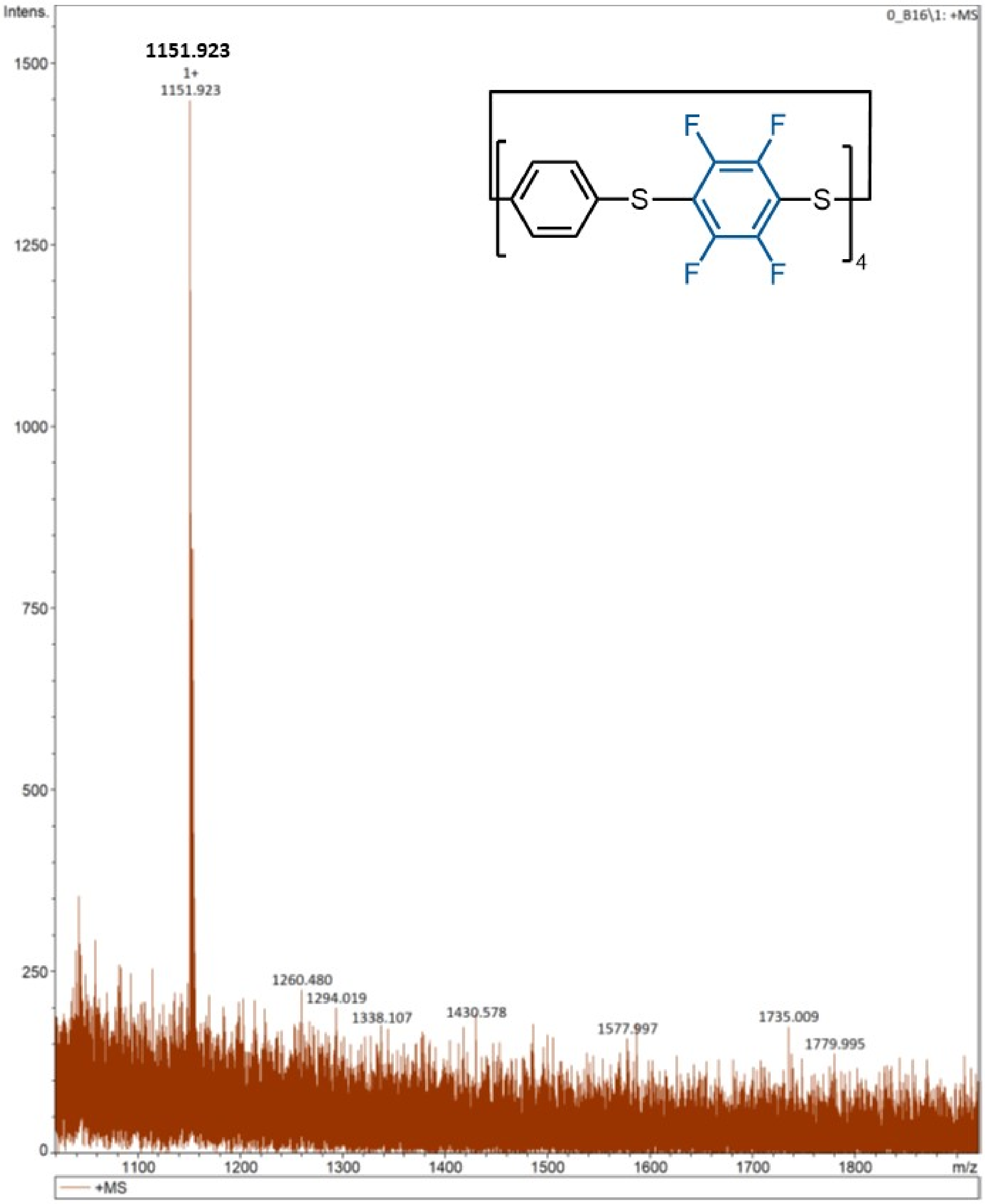

Figure S134. MALDI spectra of **1a**.

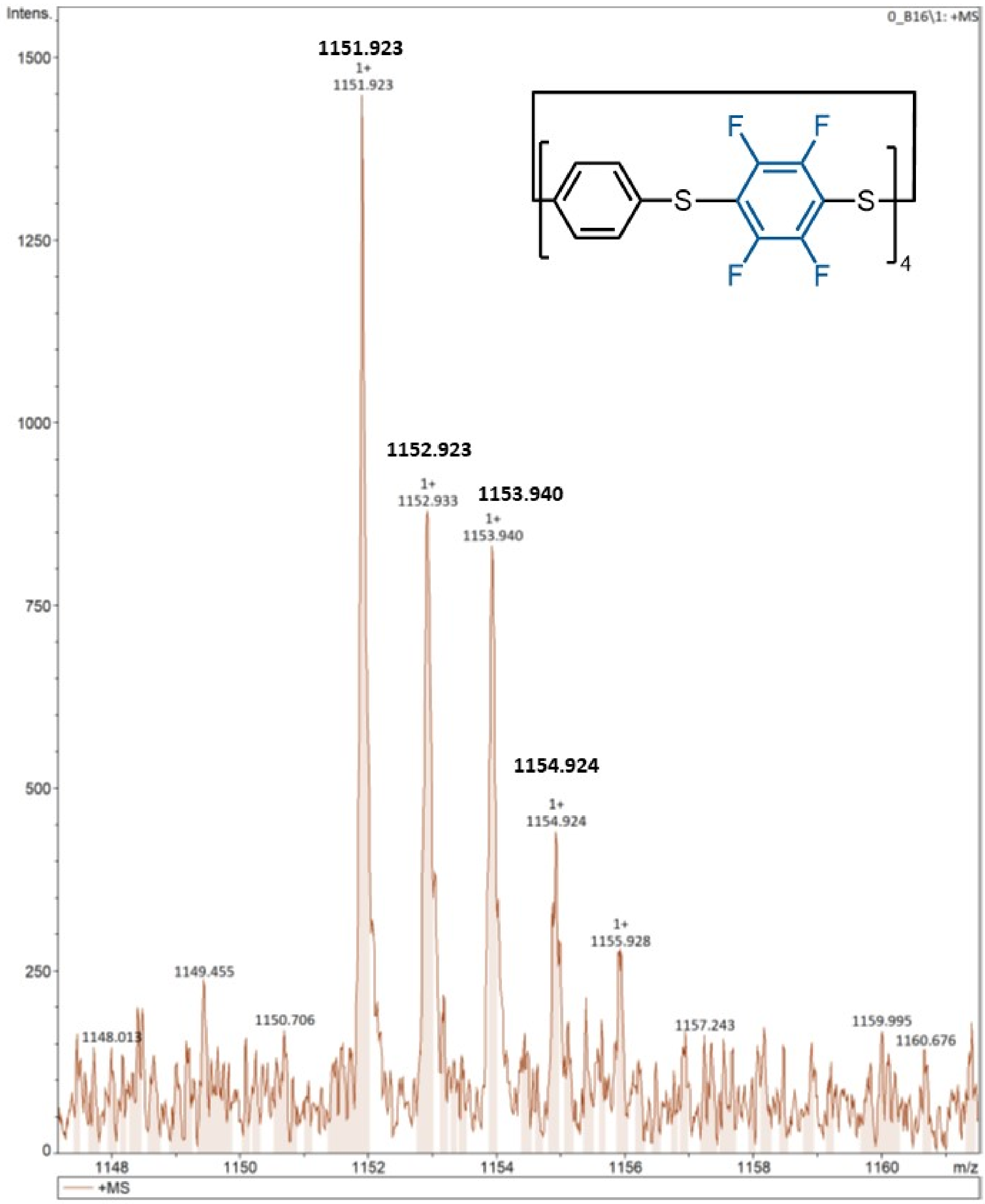

Figure S135. Zoomed in MALDI spectra of **1a**, showing the expected isotope pattern.

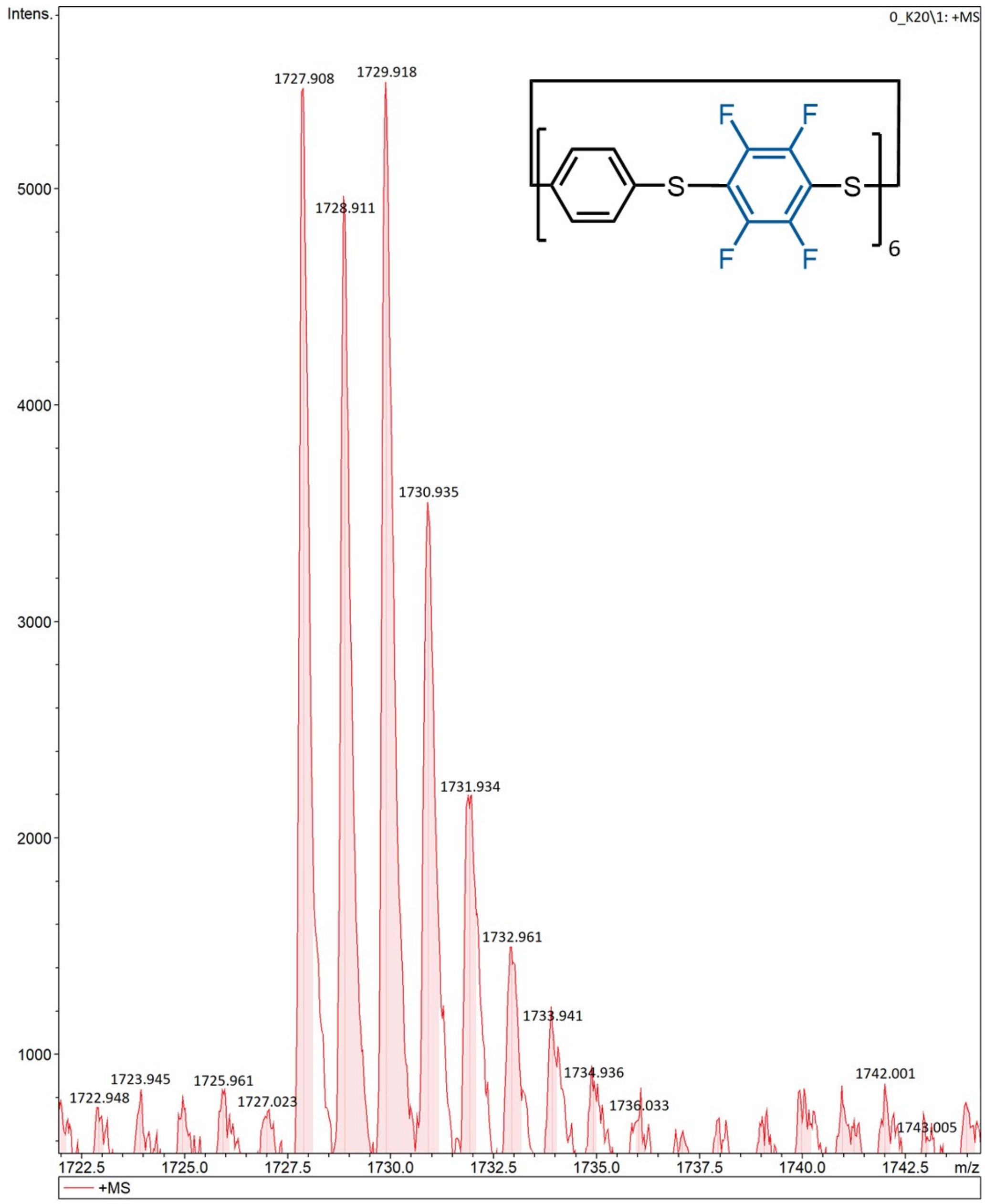

Figure S136. Zoomed in MALDI spectra of **1a'**, showing the expected isotope pattern.

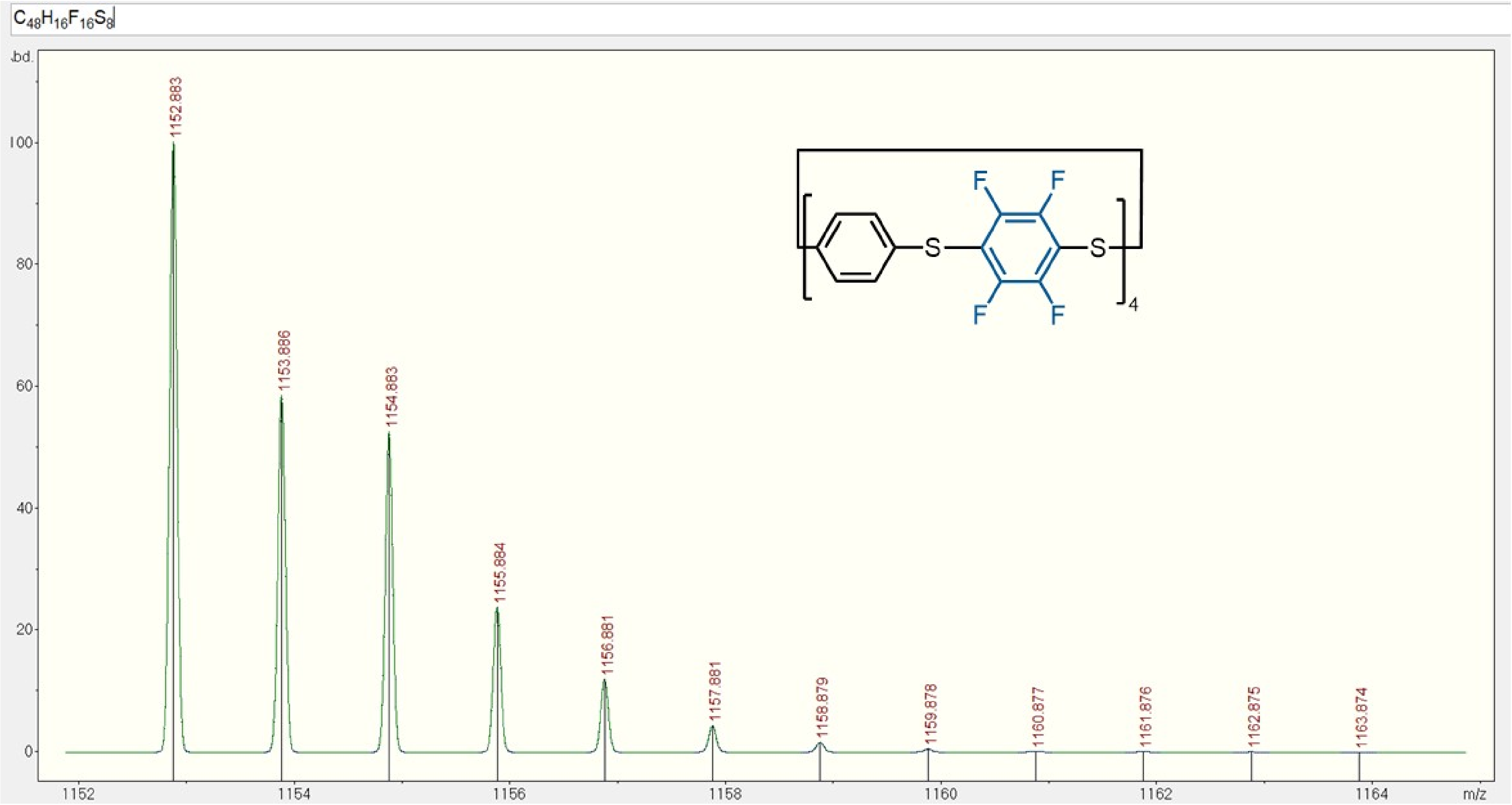

Figure S137. Simulated MALDI spectra of **1a.**

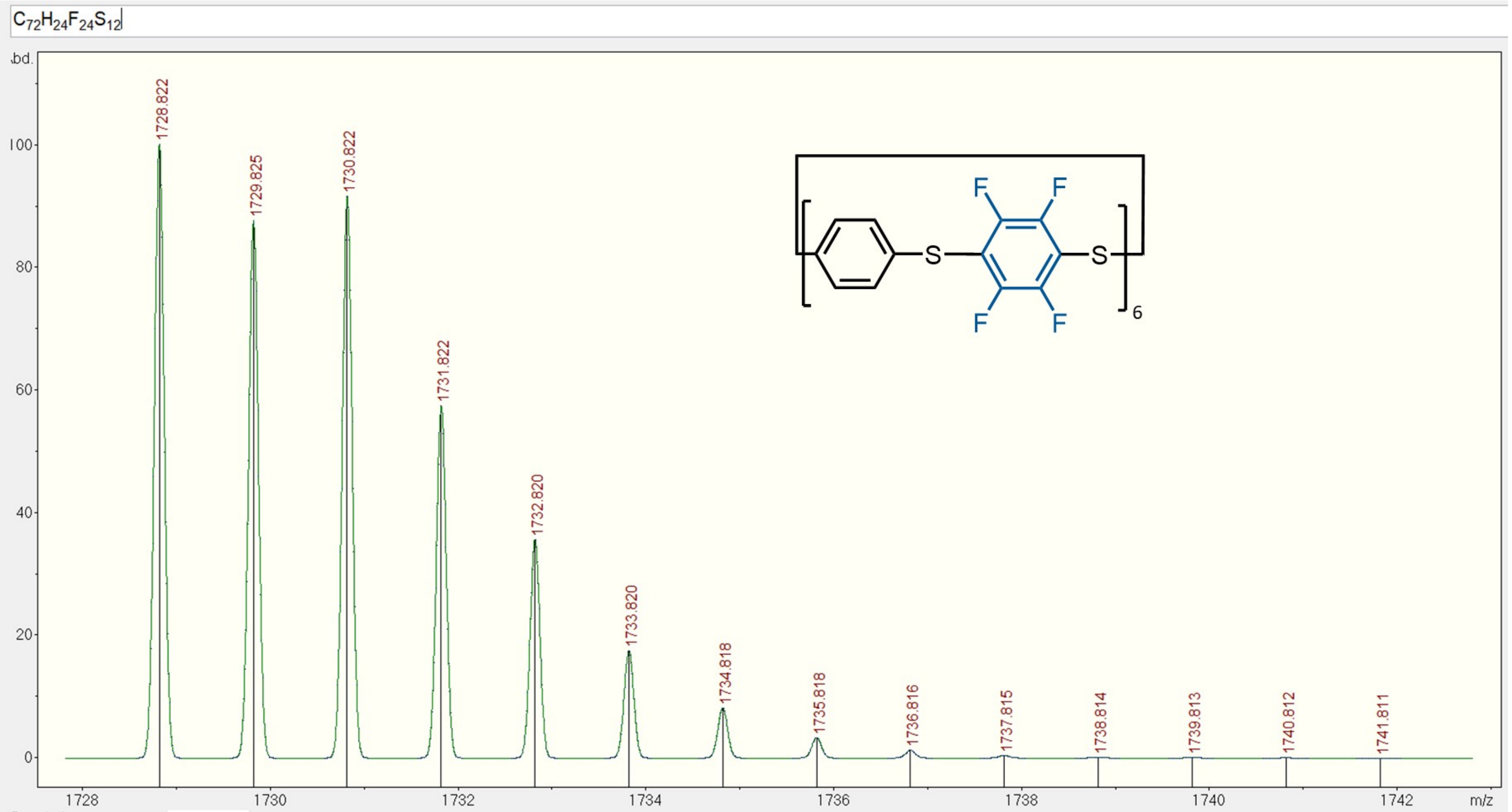

Figure S138. Simulated MALDI spectra of **1a'**.

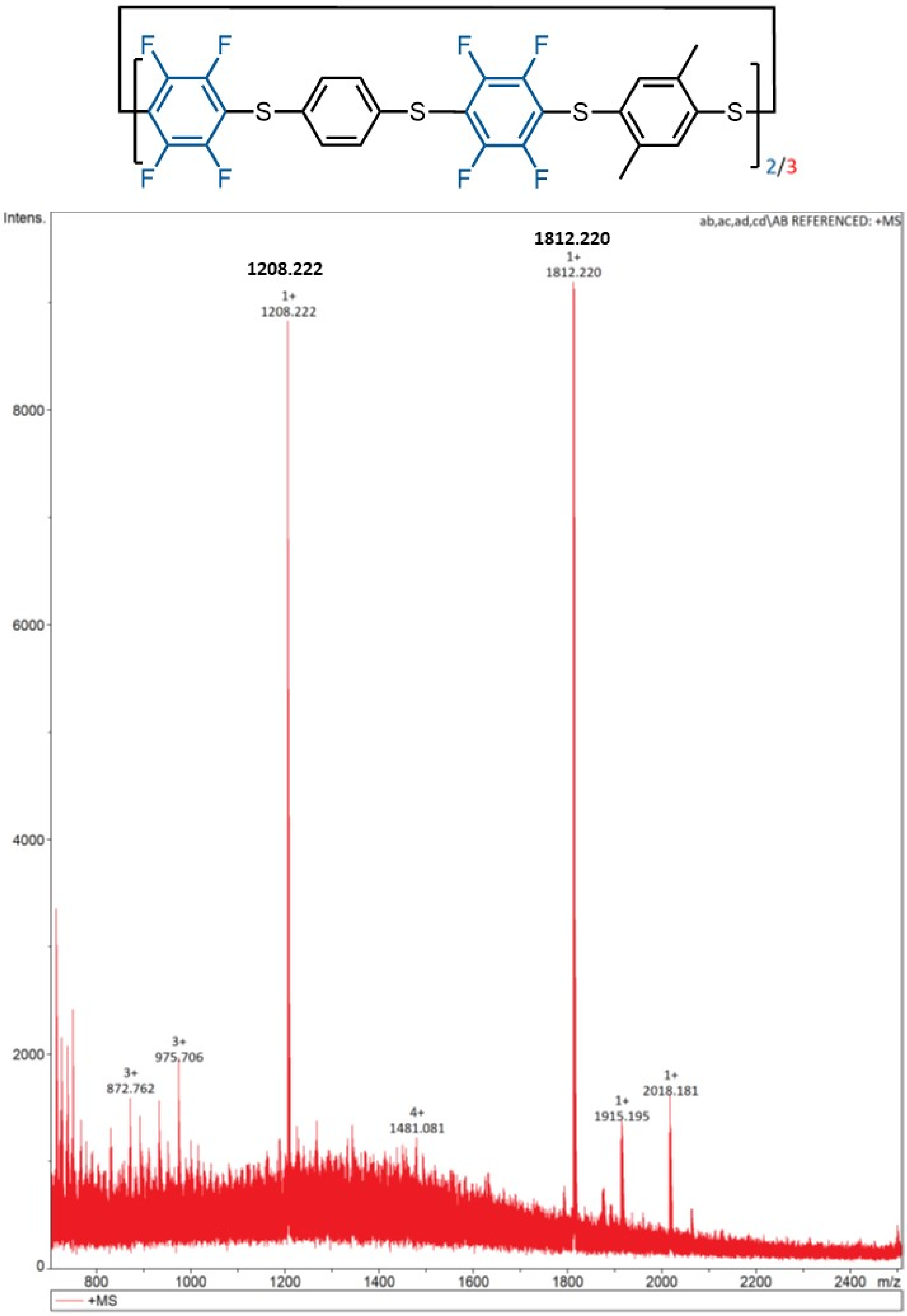

Figure S139. MALDI spectra of **1ab**, showing both macrocycle sizes.

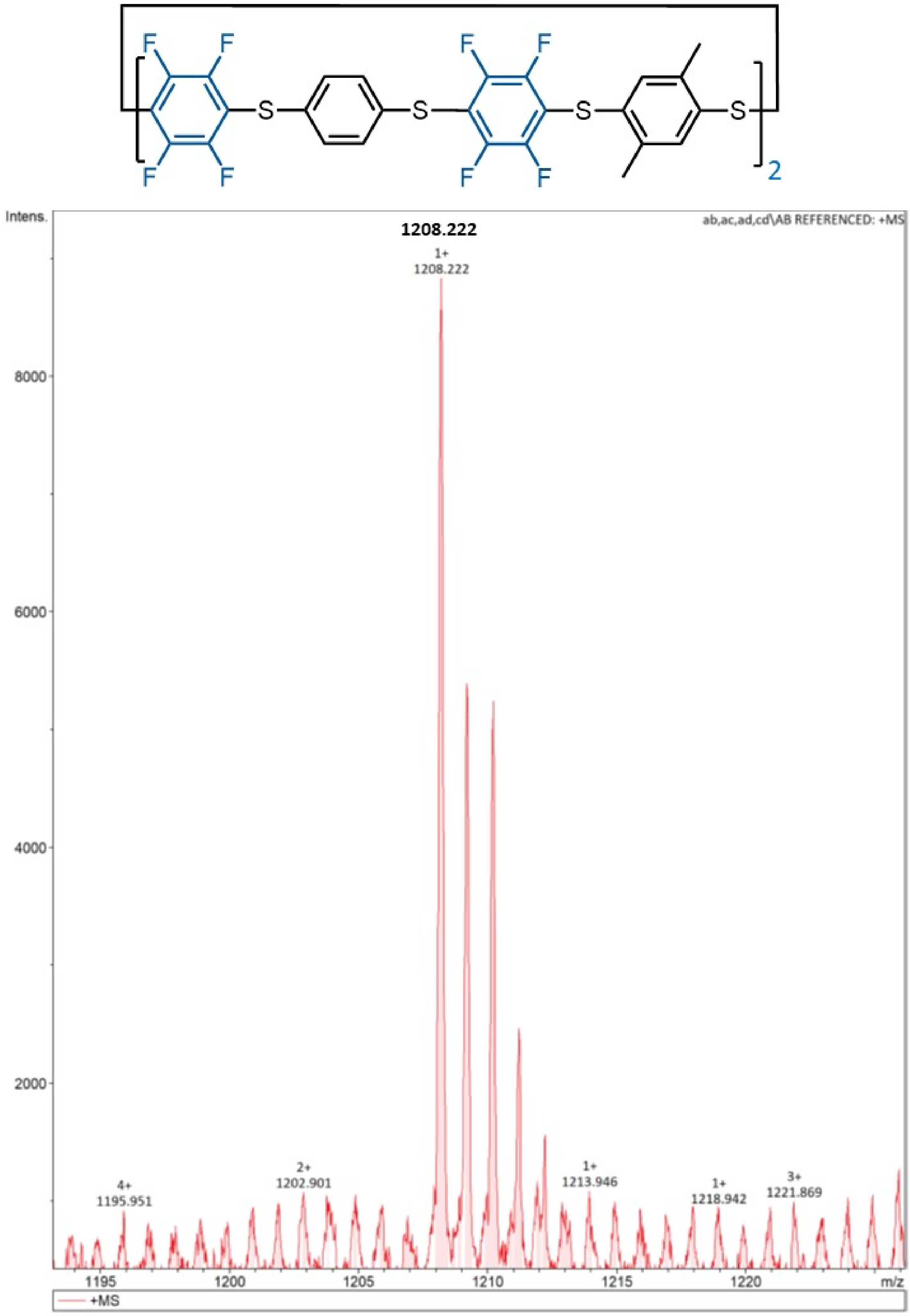

Figure S140. Zoomed in MALDI spectra of **1ab** (n = 2), showing the expected isotope pattern.

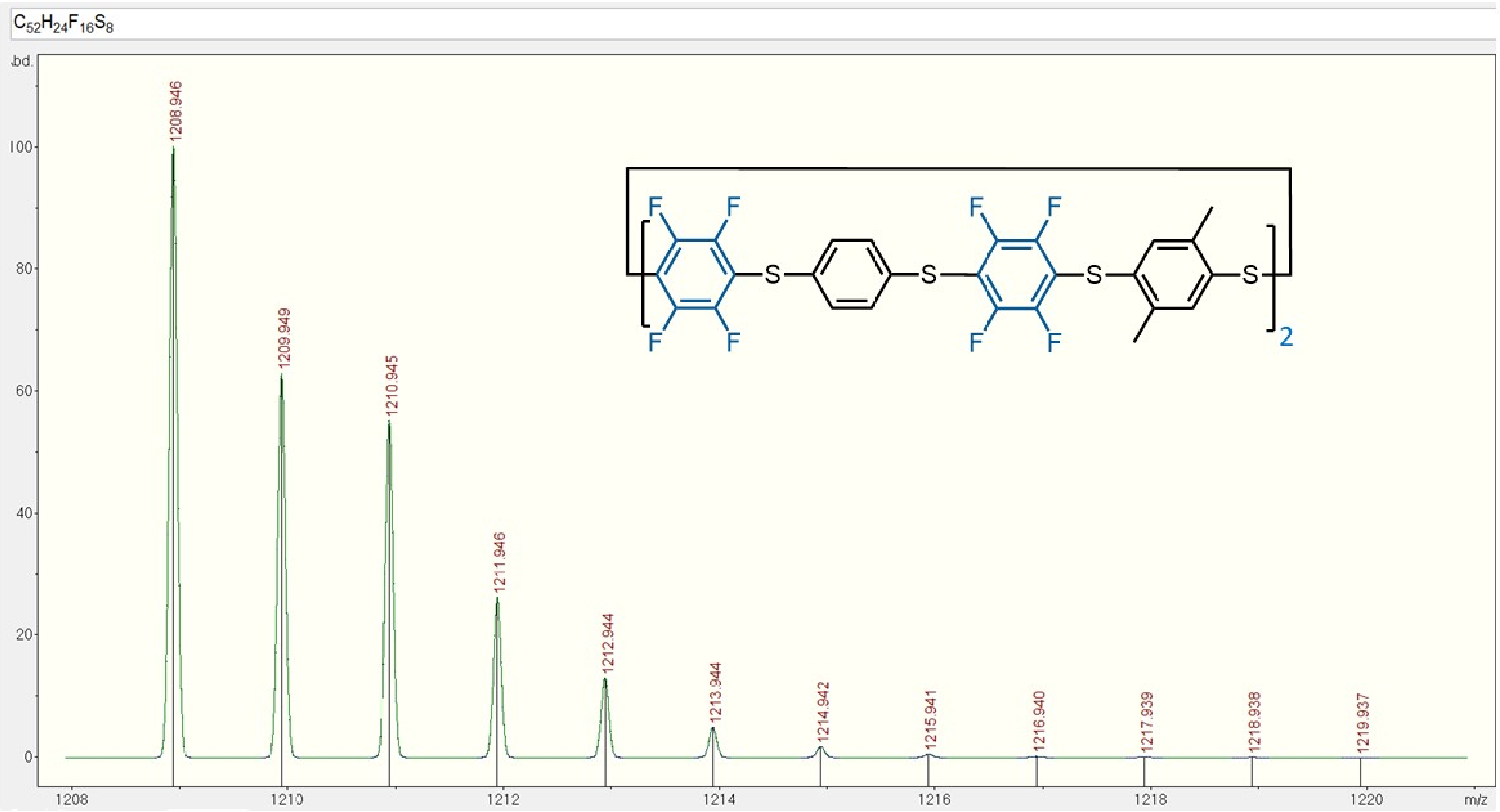

Figure S141. Simulated Zoomed in MALDI spectra of **1ab** (n = 2).

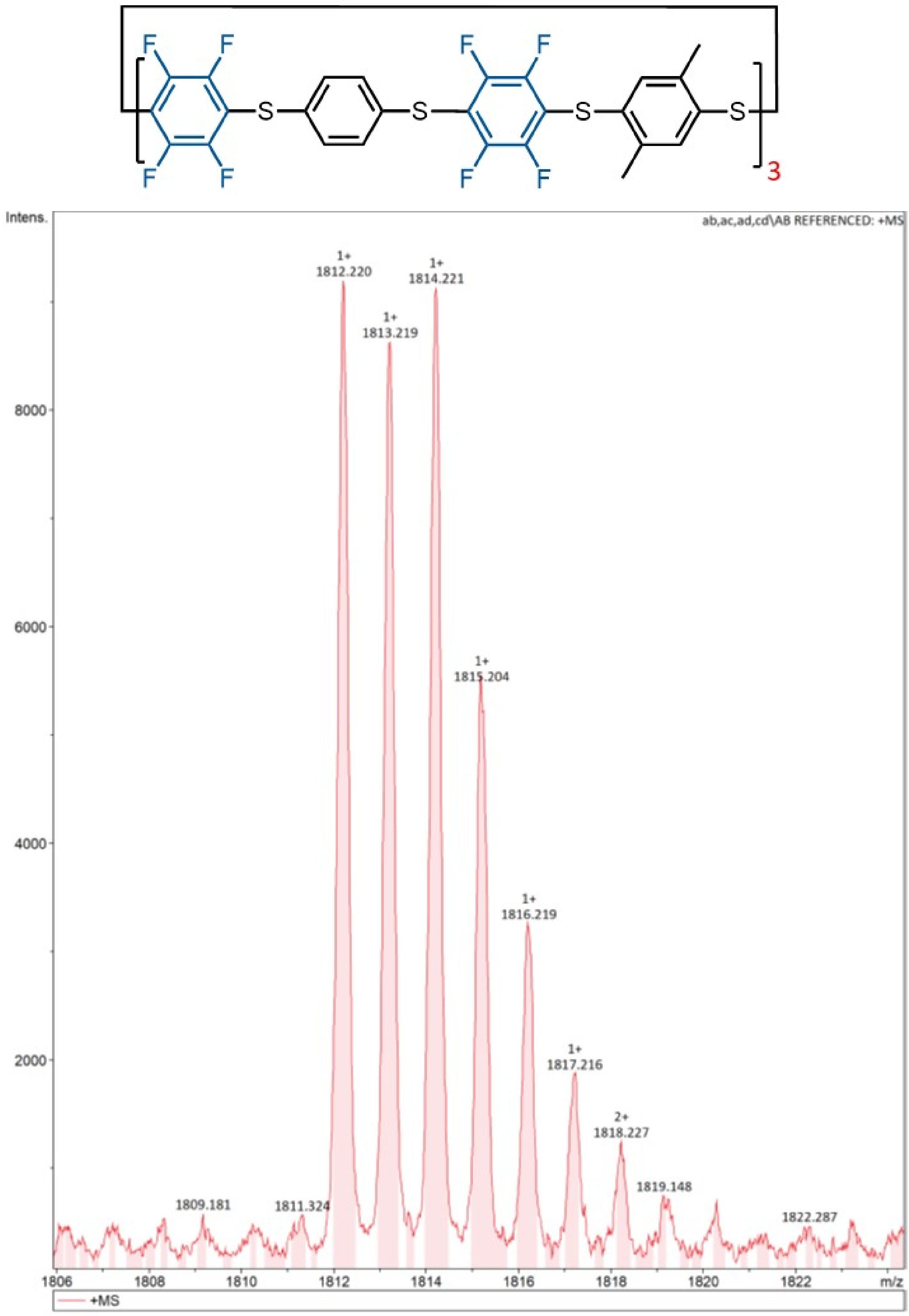

Figure S142. MALDI spectra of **1ab** (n = 3).

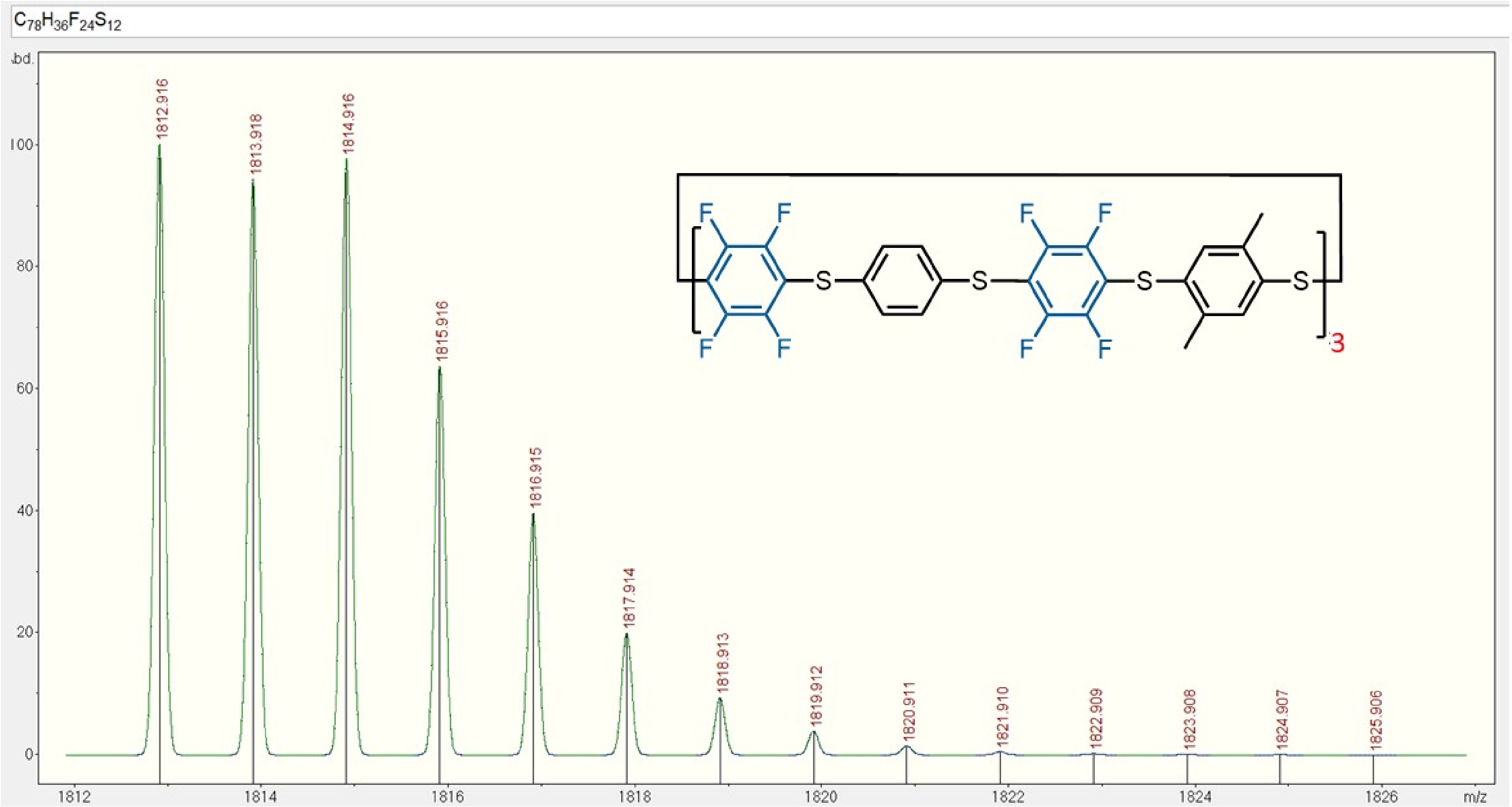

Figure S143. Simulated MALDI spectra of **1ab** (n = 3) showing the expected isotope pattern.

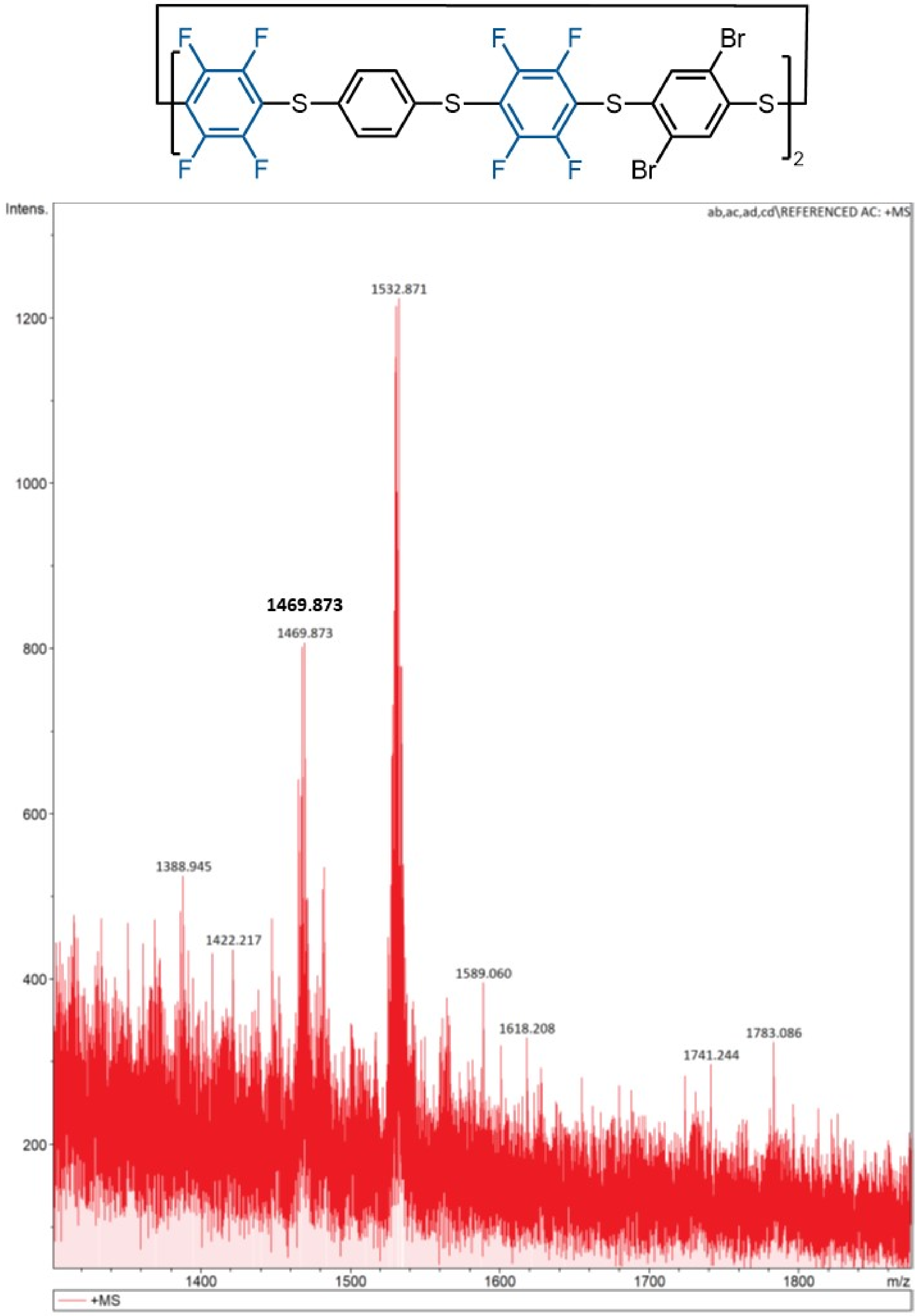

Figure S144. MALDI spectra of **1ac**.

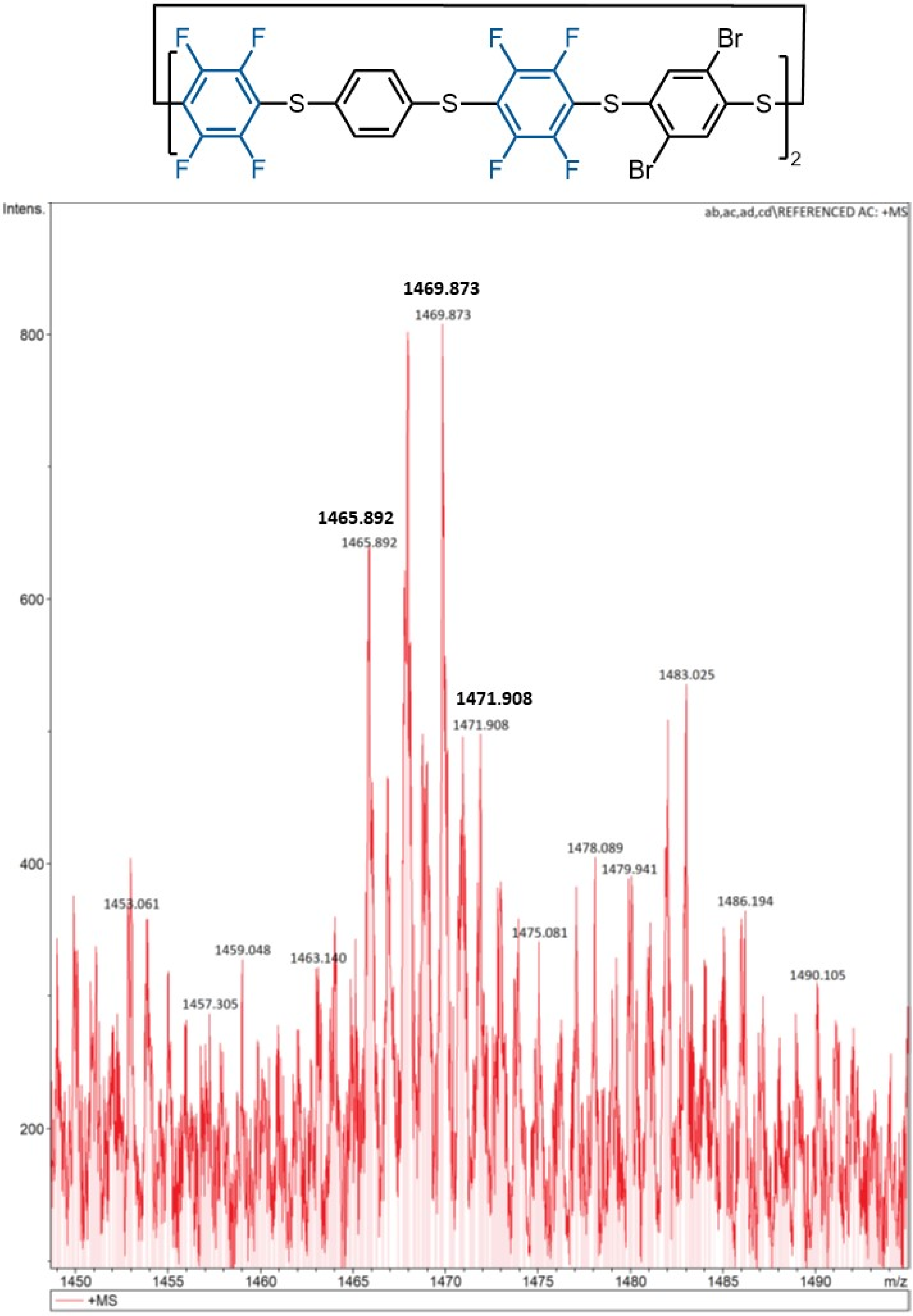

Figure S145. Zoomed in MALDI spectra of **1ac**.

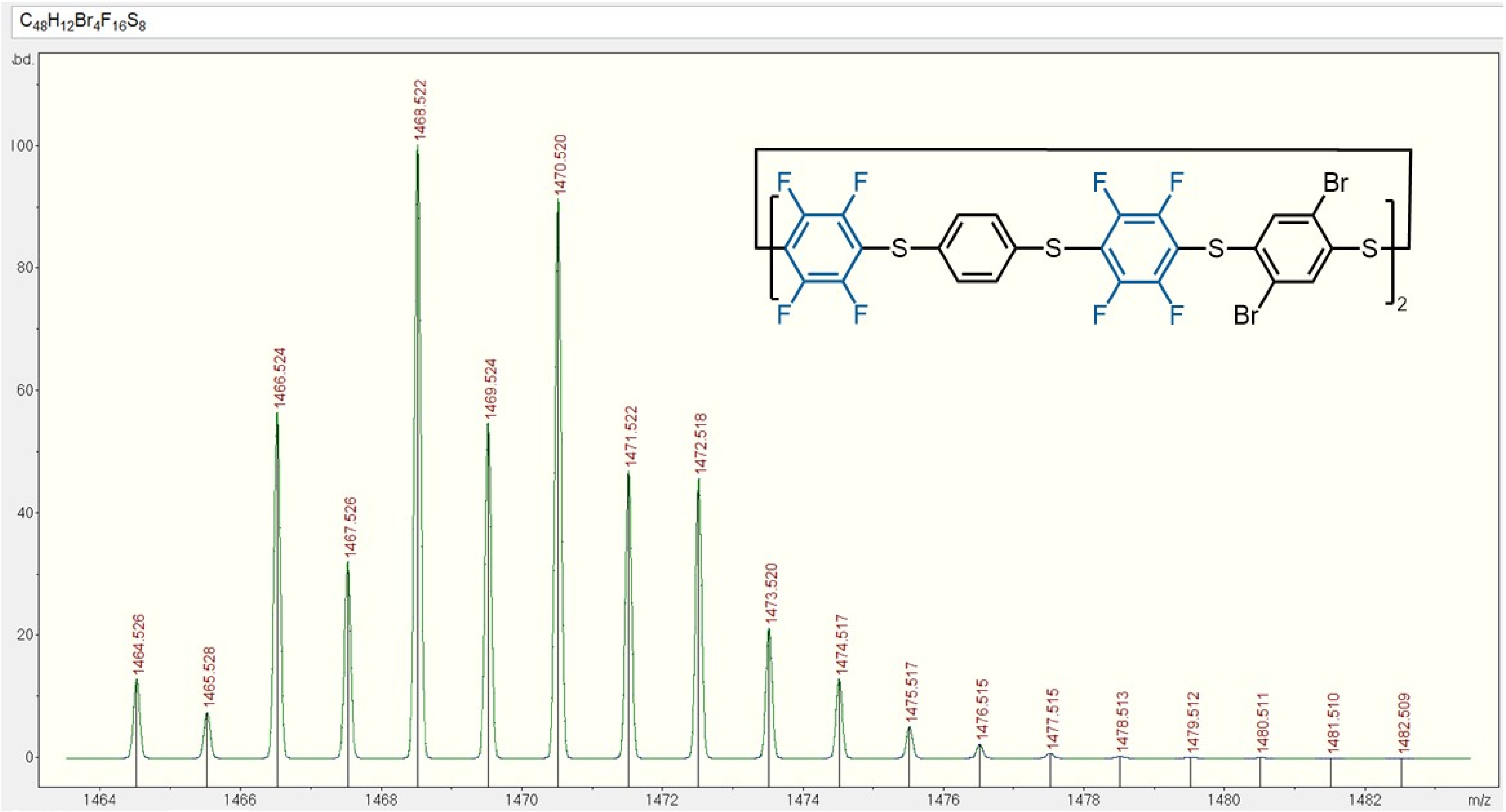

Figure S146. Simulated MALDI spectra of **1ac** showing the expected isotope pattern.

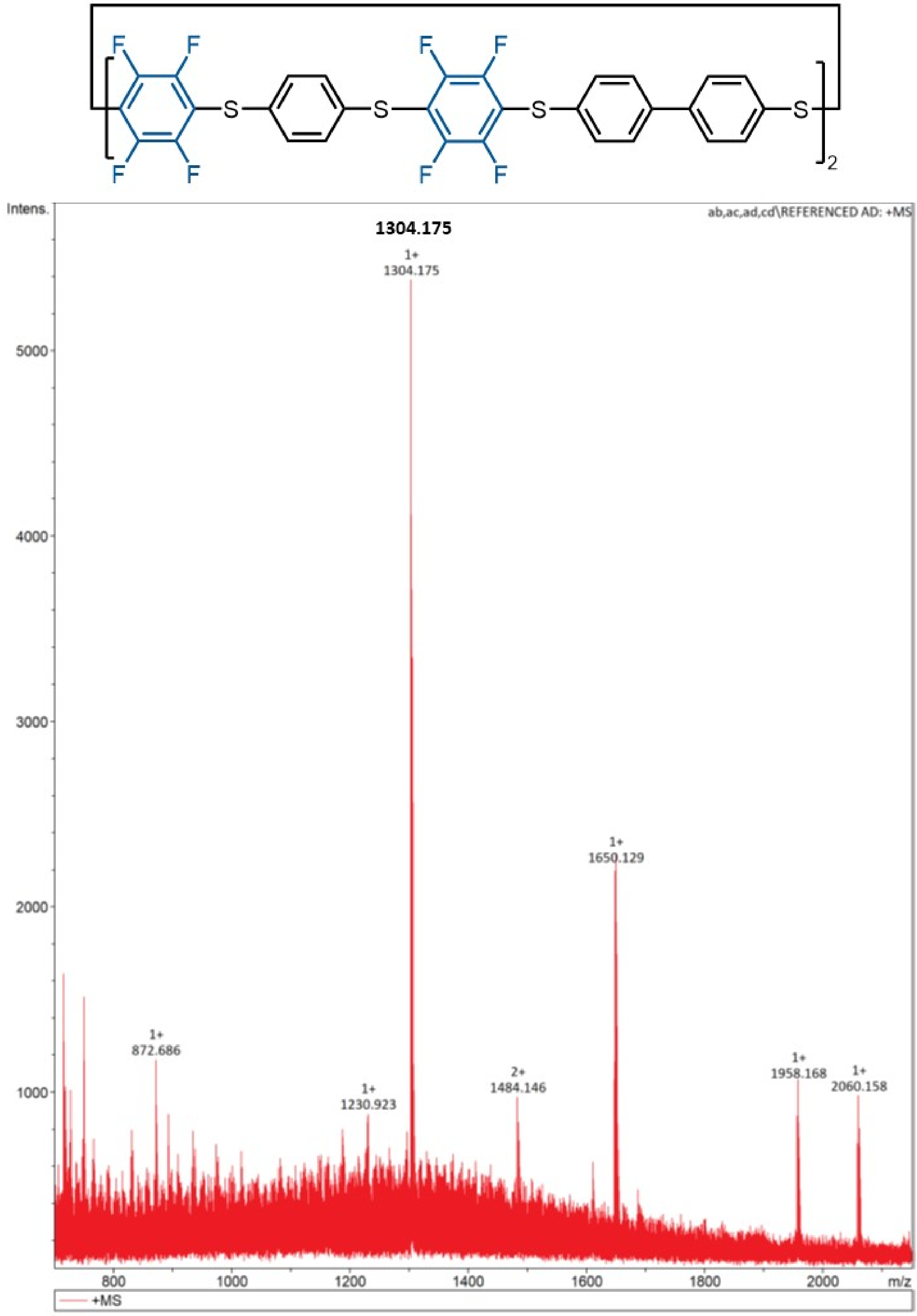

Figure S147. MALDI spectra of **1ad**.

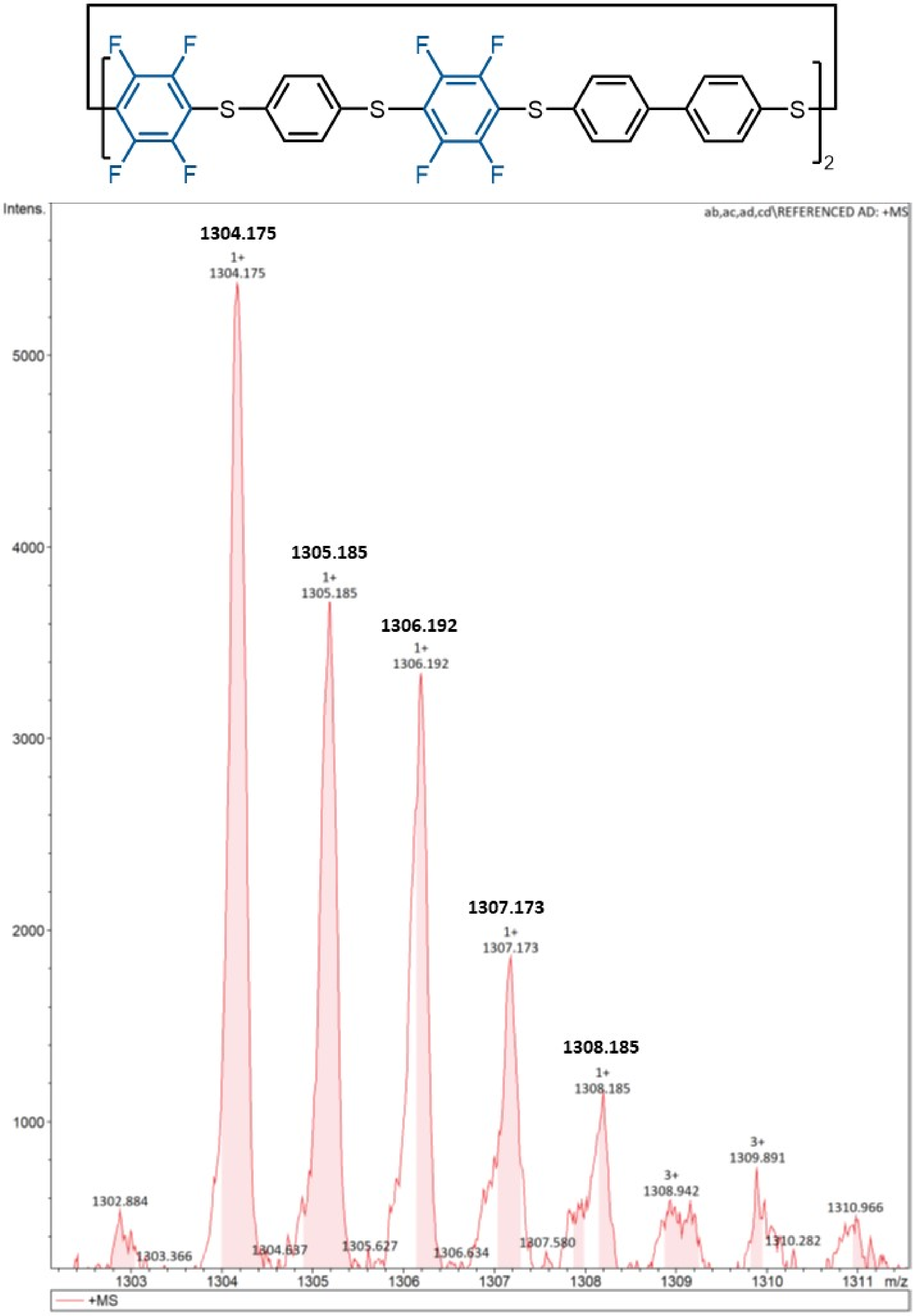

Figure S148. Zoomed in MALDI spectra of **1ad**.

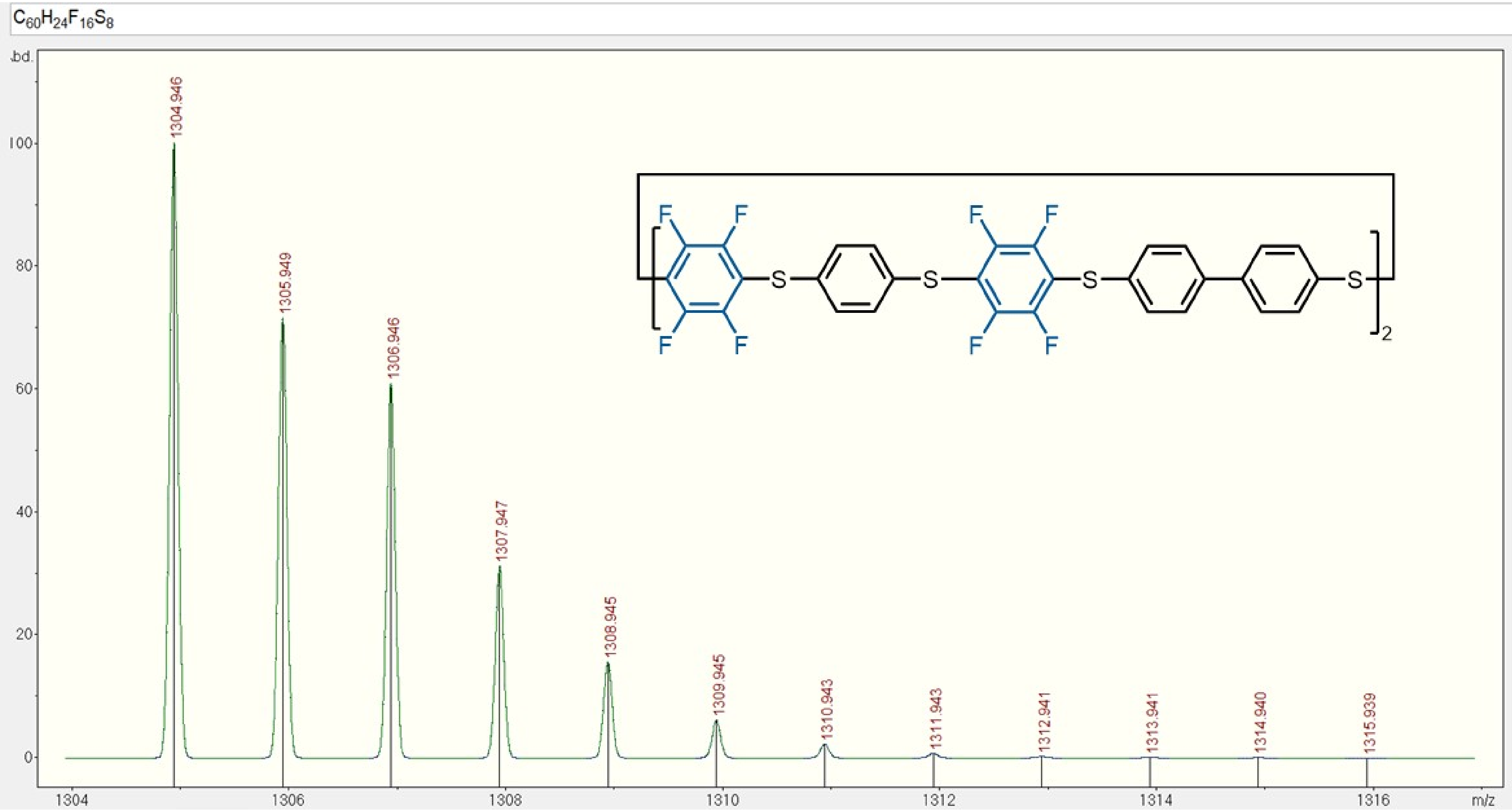

Figure S149. Simulated MALDI spectra of **1ac** showing the expected isotope pattern.

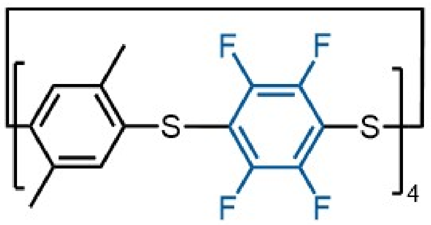

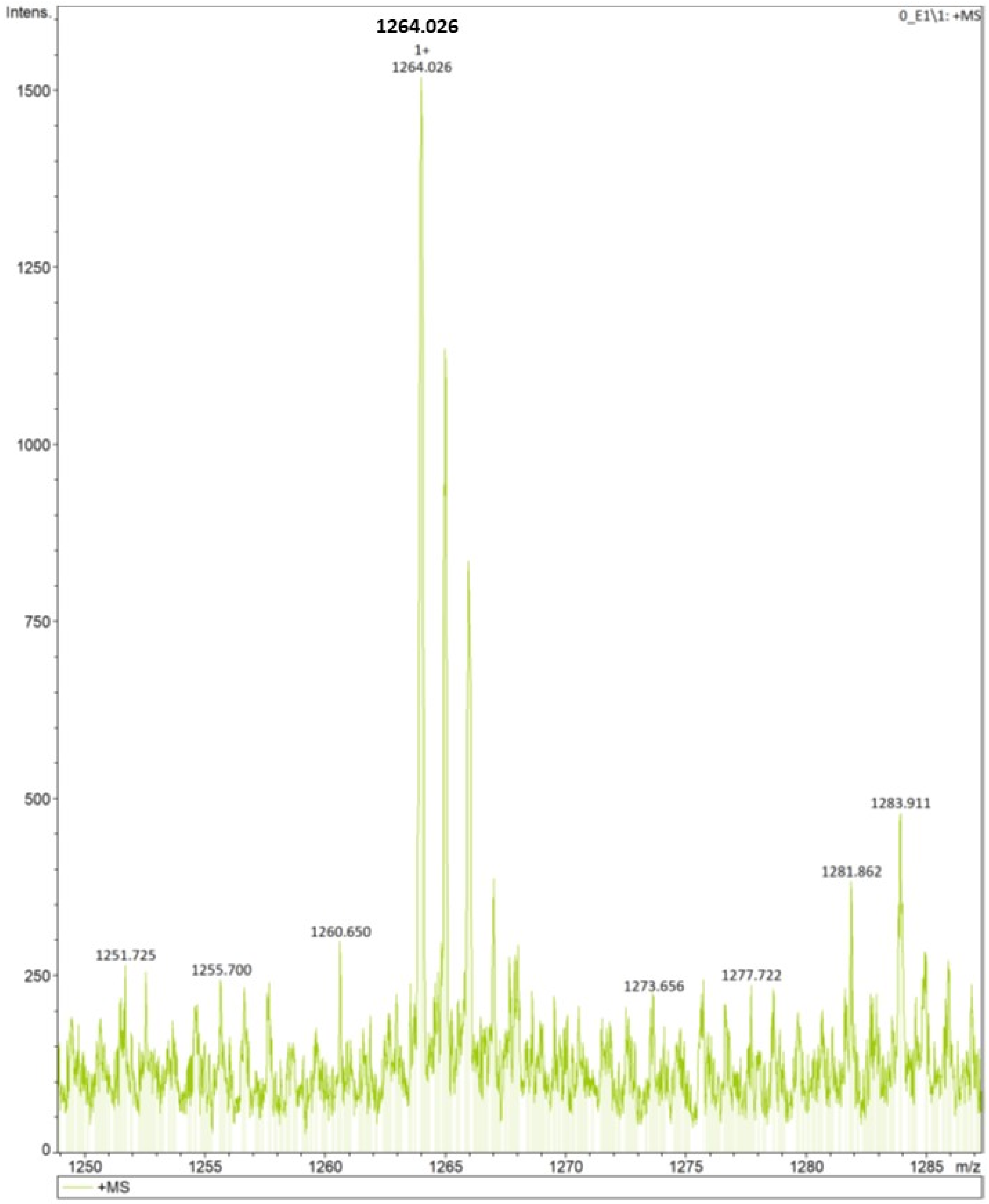

Figure S150. MALDI spectra of **1b**.

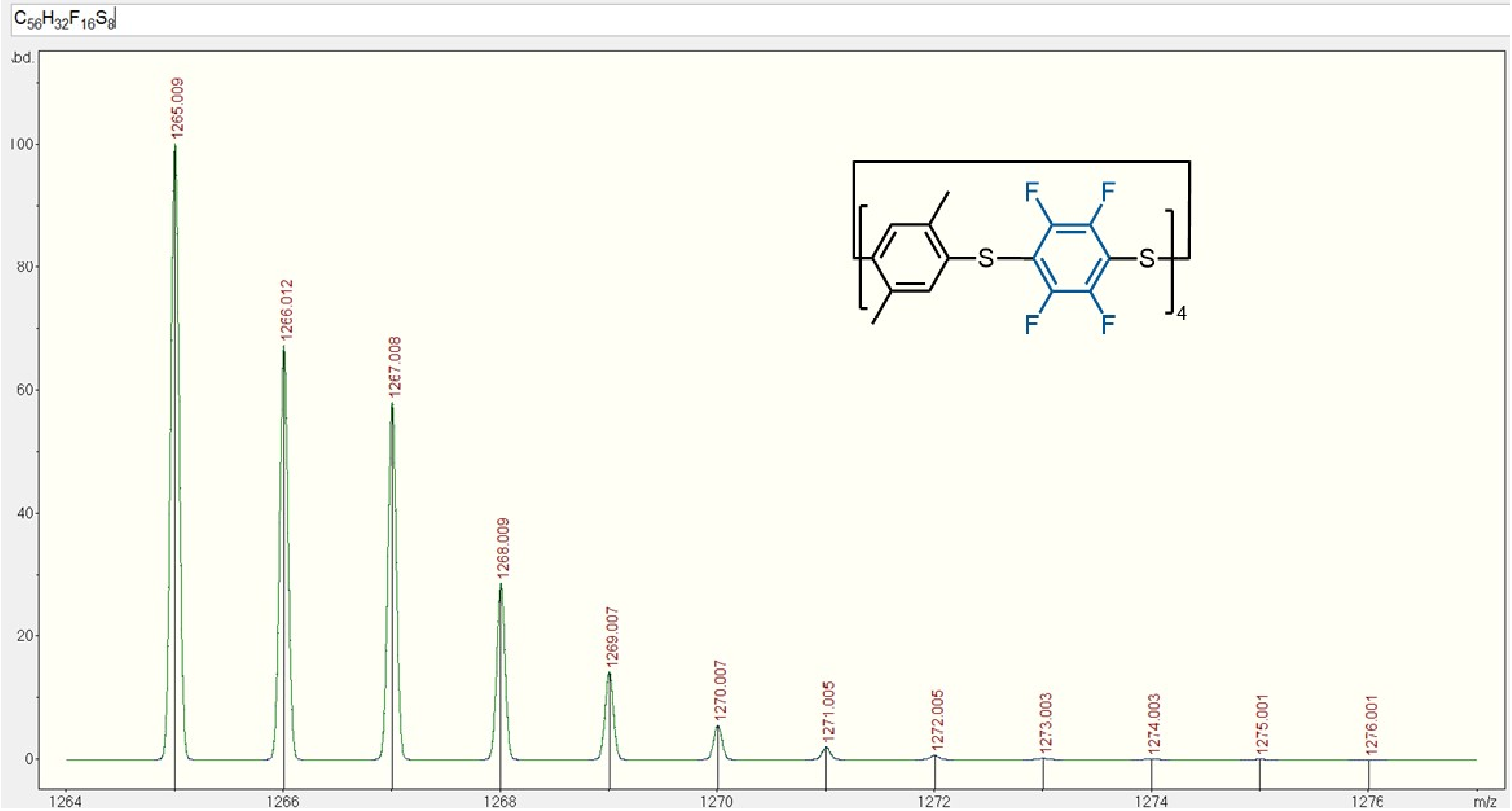

Figure S151. Simulated MALDI spectra of **1b** showing the expected isotope pattern.

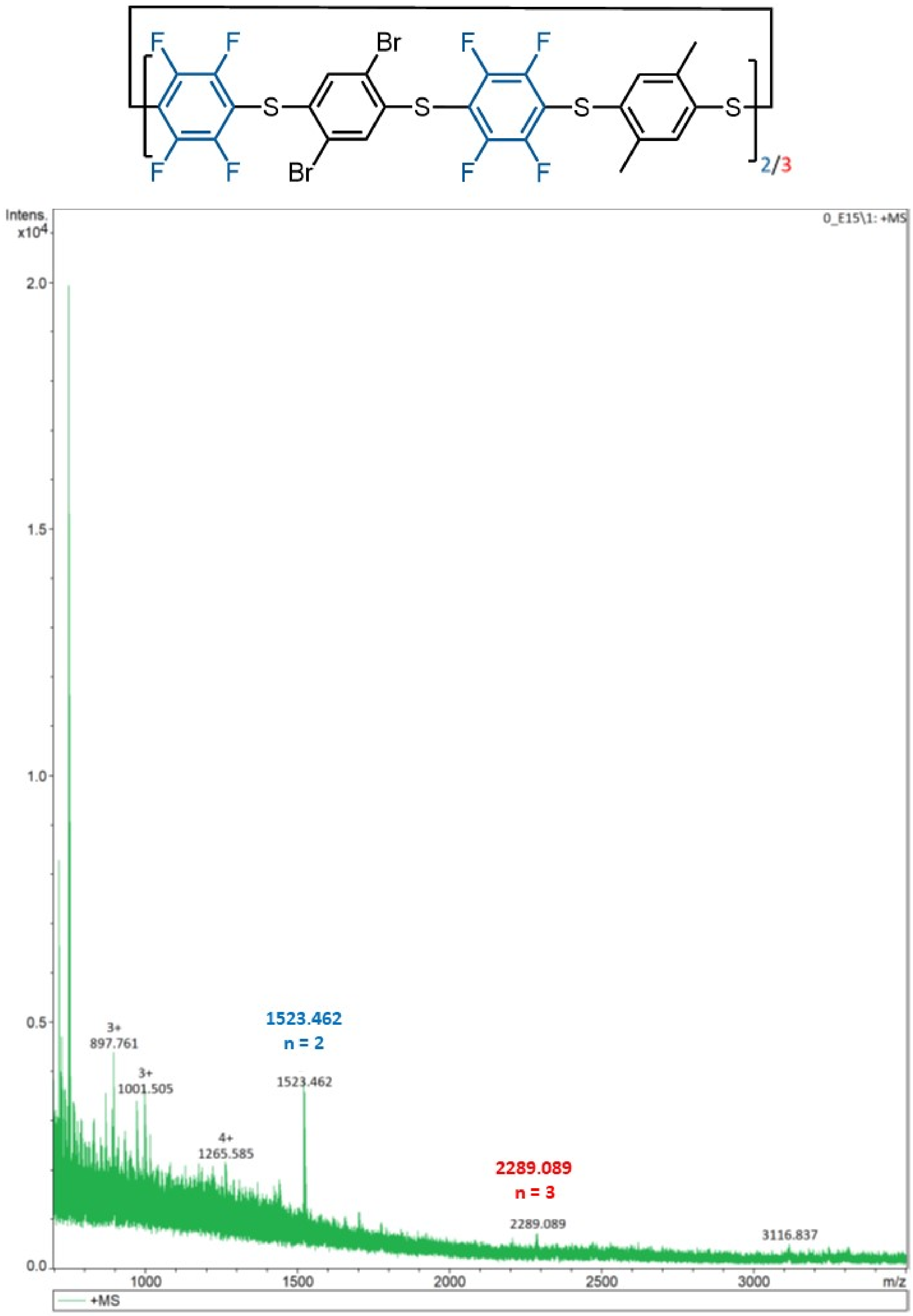

Figure S152. MALDI spectra of **1bc** showing both isomers (n = 2) and (n = 3).

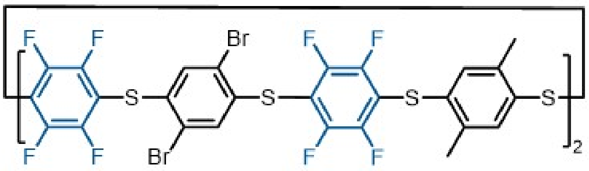

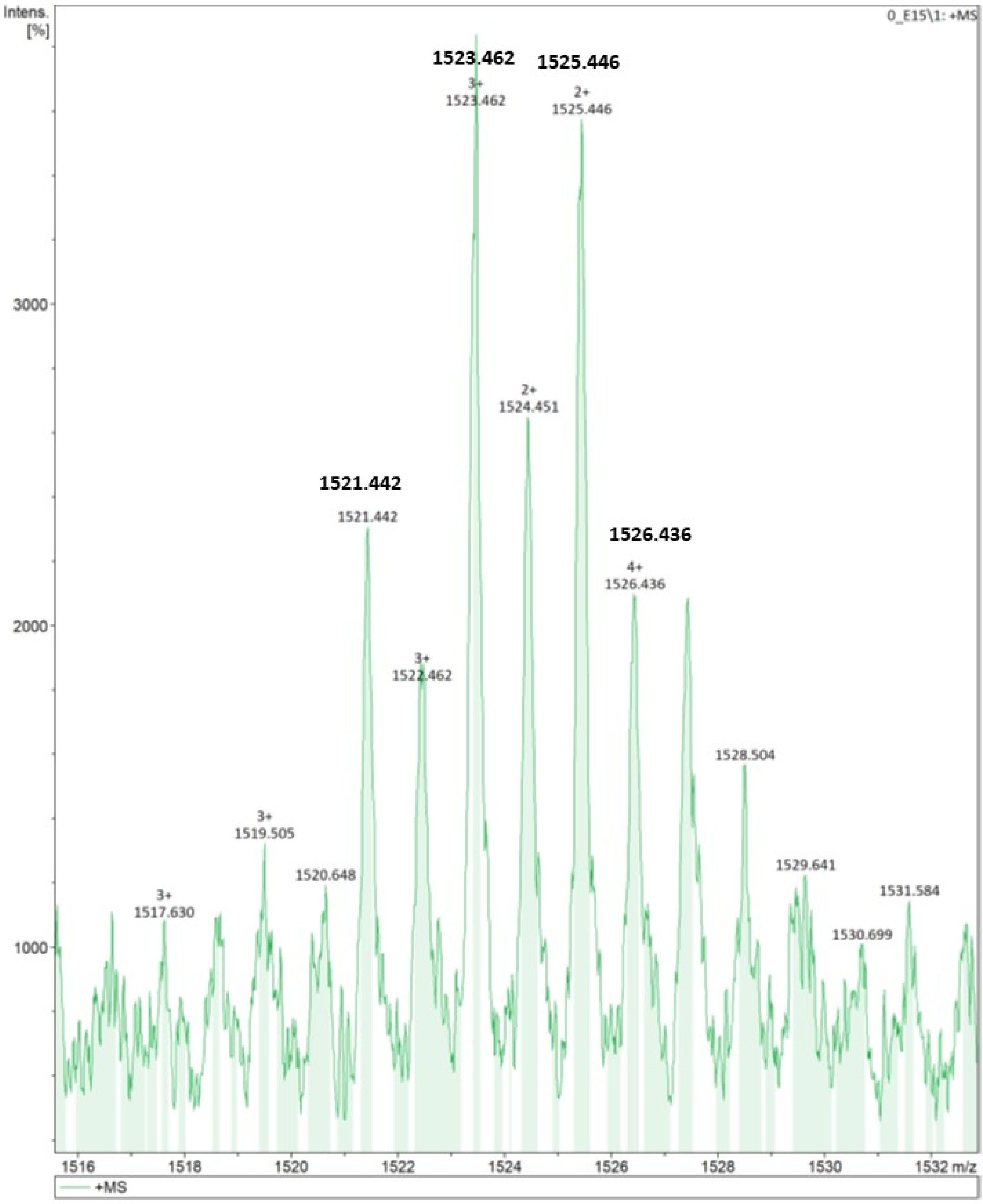

Figure S153. Zoomed in MALDI spectra of **1bc** (n = 2) showing the expected isotope pattern.

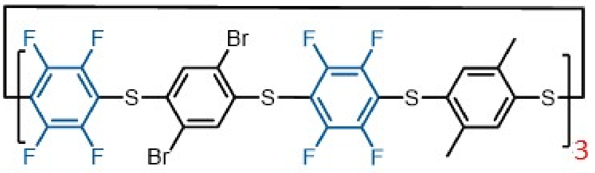

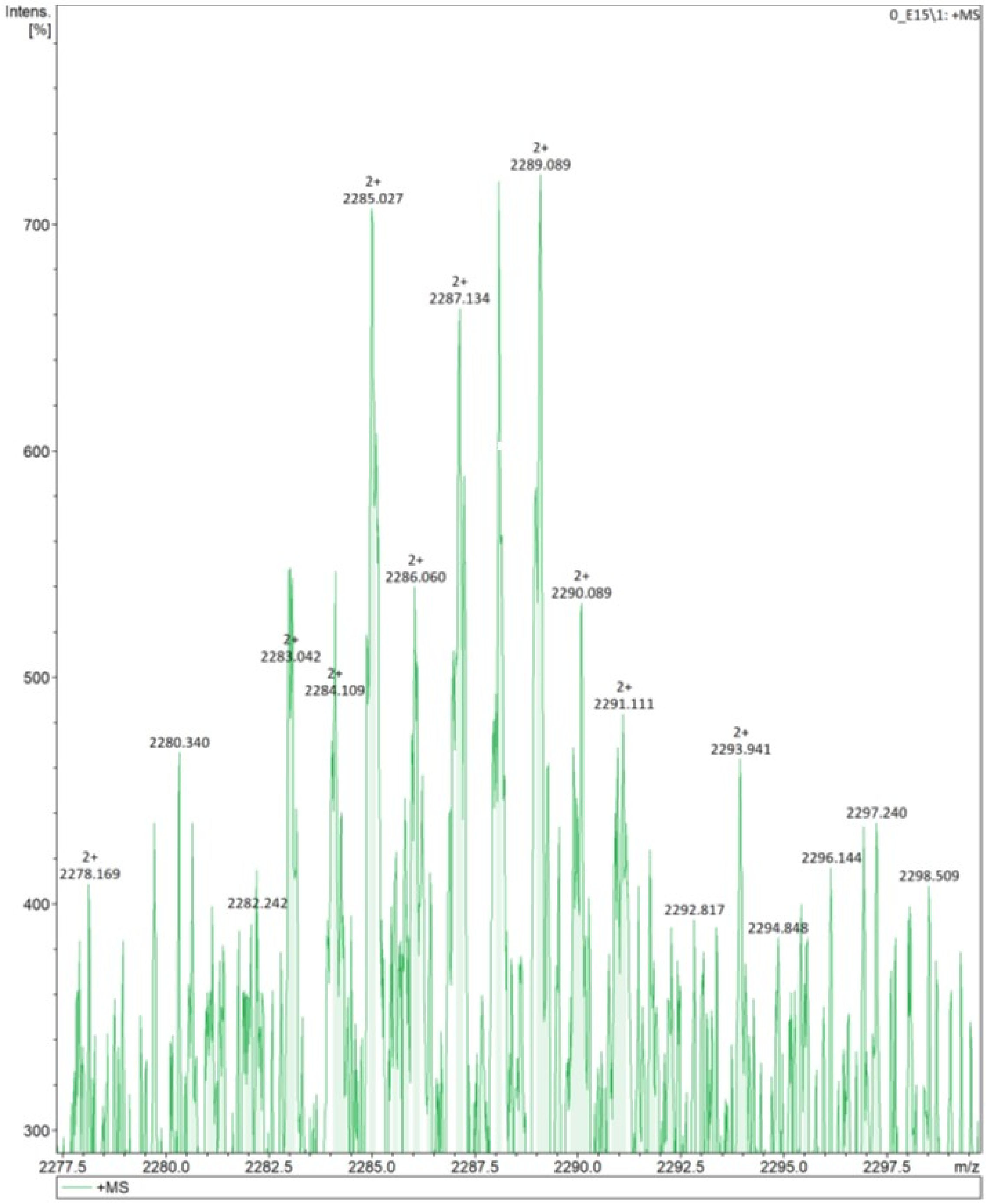

Figure S154. Zoomed in MALDI spectra of **1bc** (n = 3) showing the expected isotope pattern.

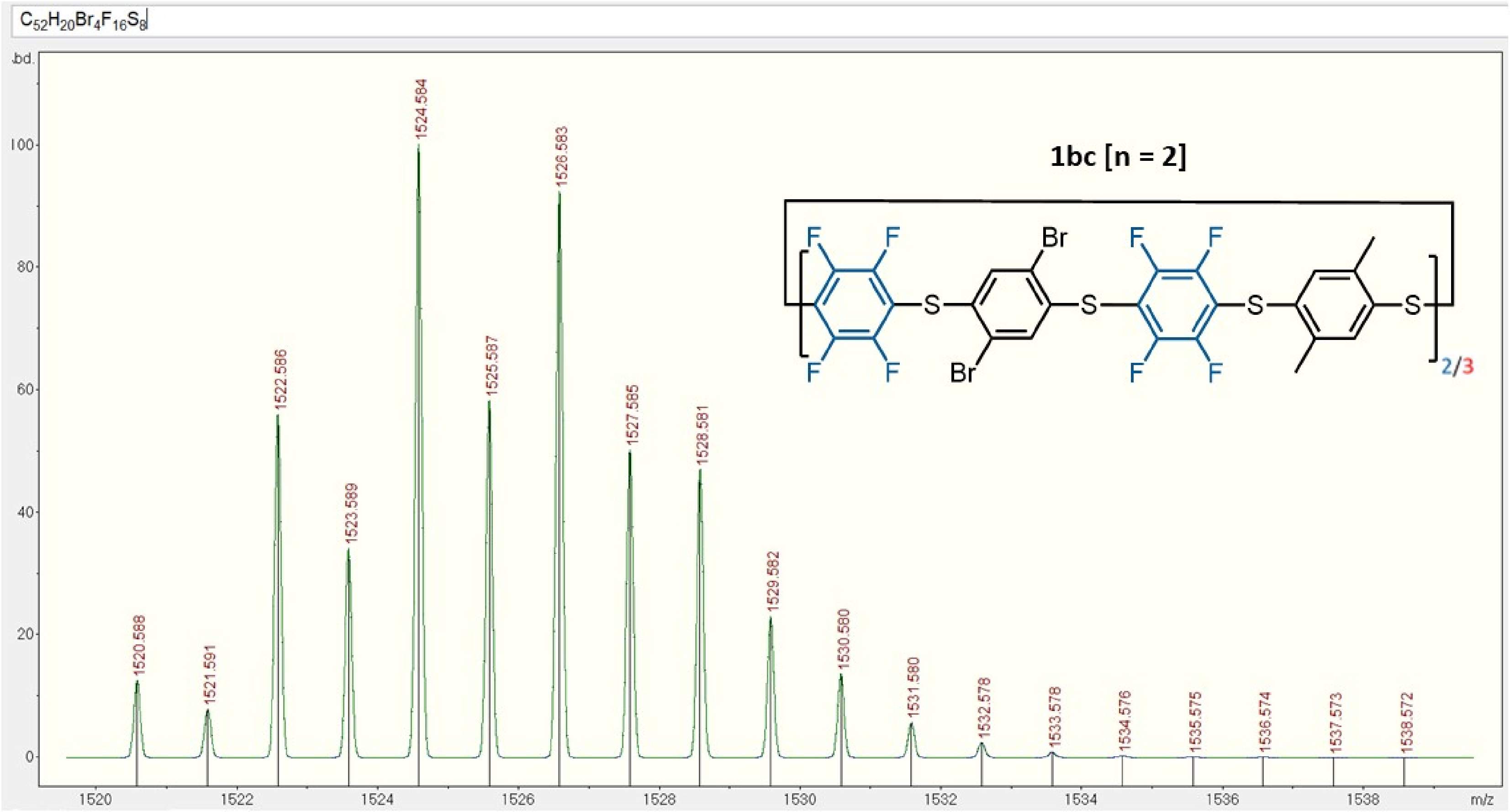

Figure S155. Simulated MALDI spectra of **1bc** (n = 2).

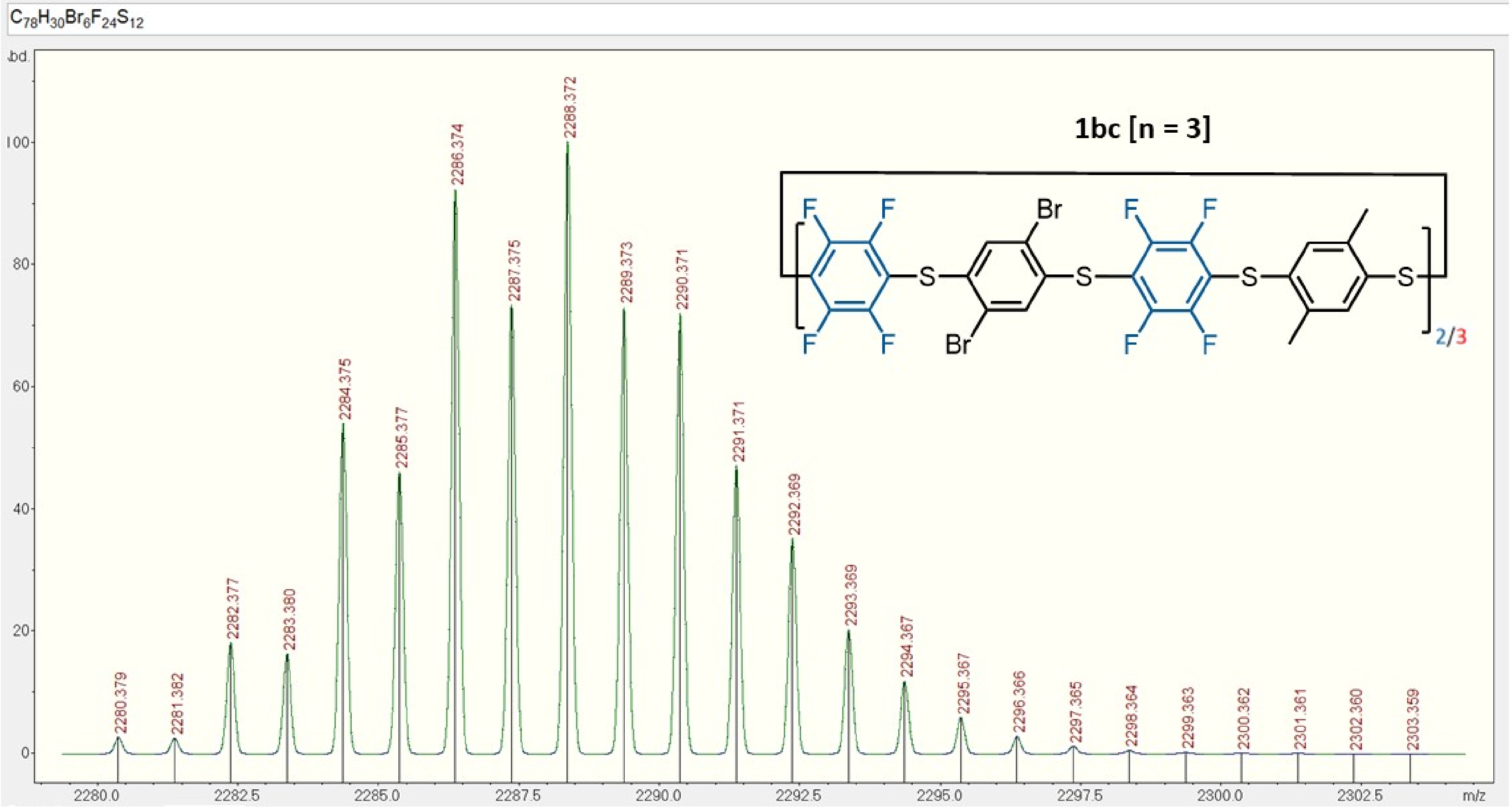

Figure S156. Simulated MALDI spectra of **1bc** (n = 3).

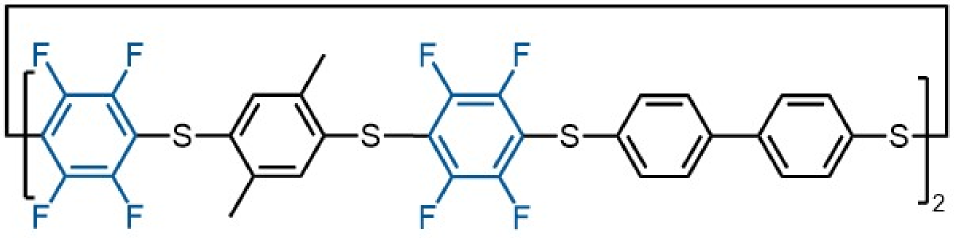

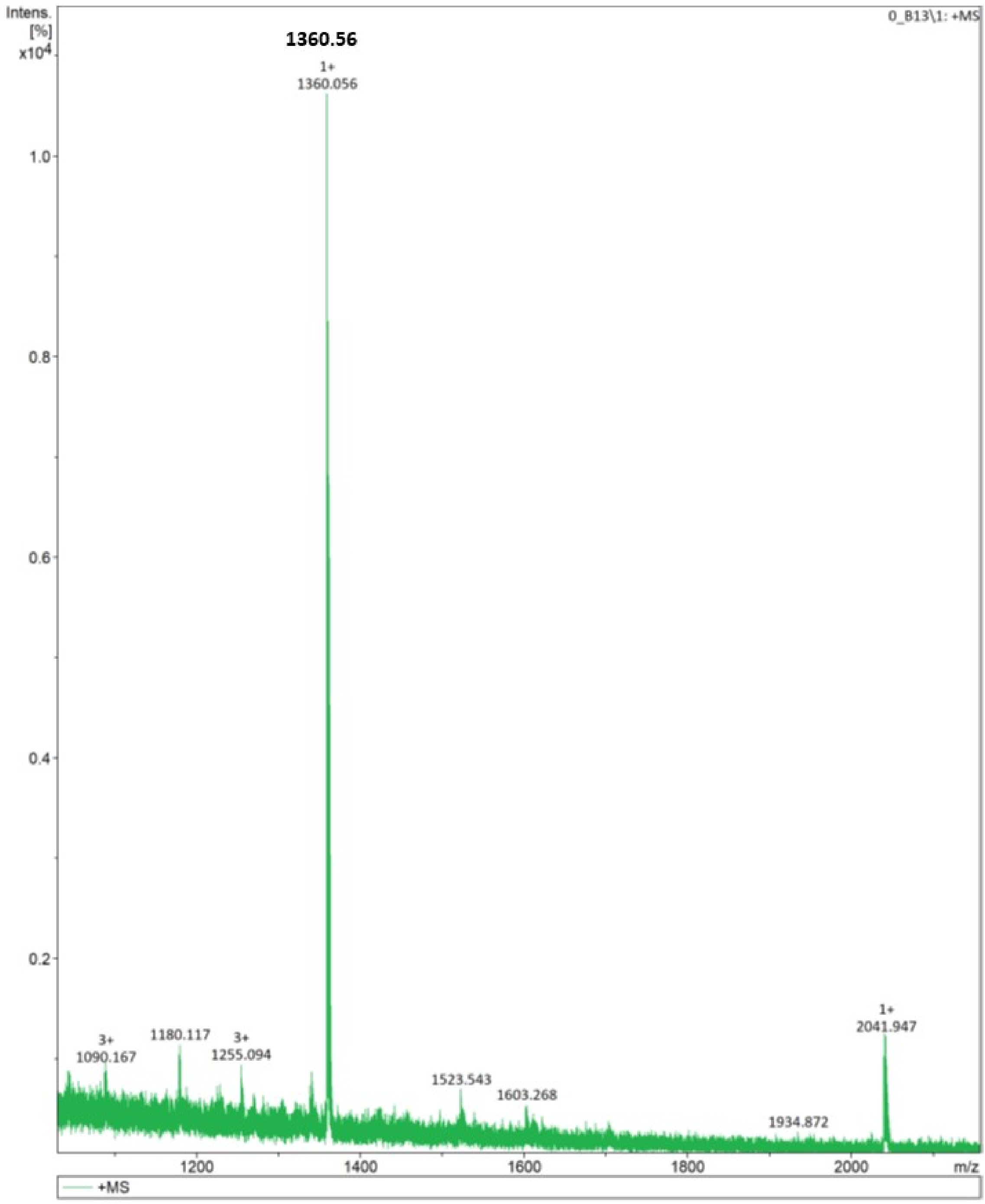

Figure S157. MALDI spectra of **1bd**.

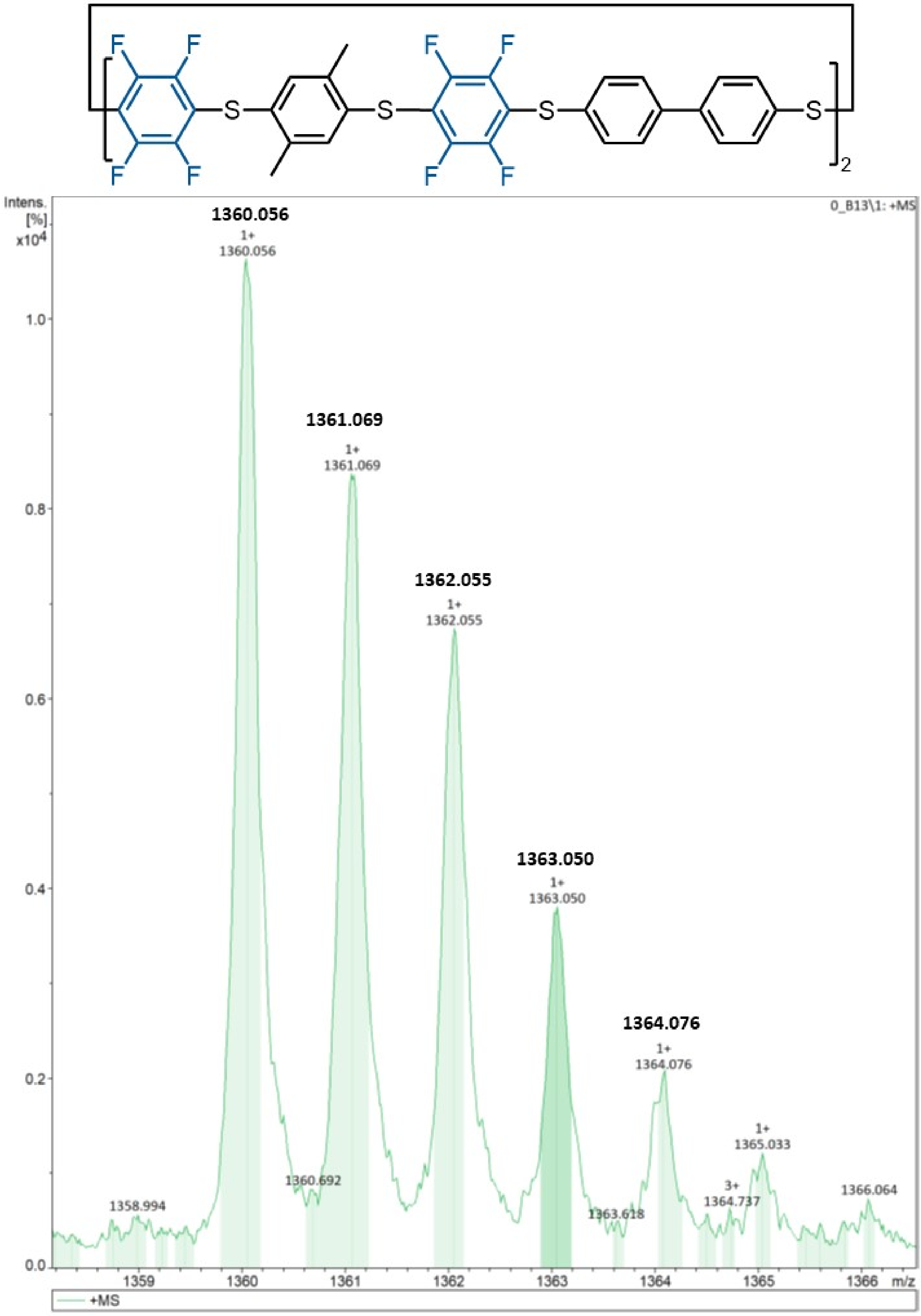

Figure S158. Zoomed in MALDI spectra of **1bd** showing the expected isotope pattern.

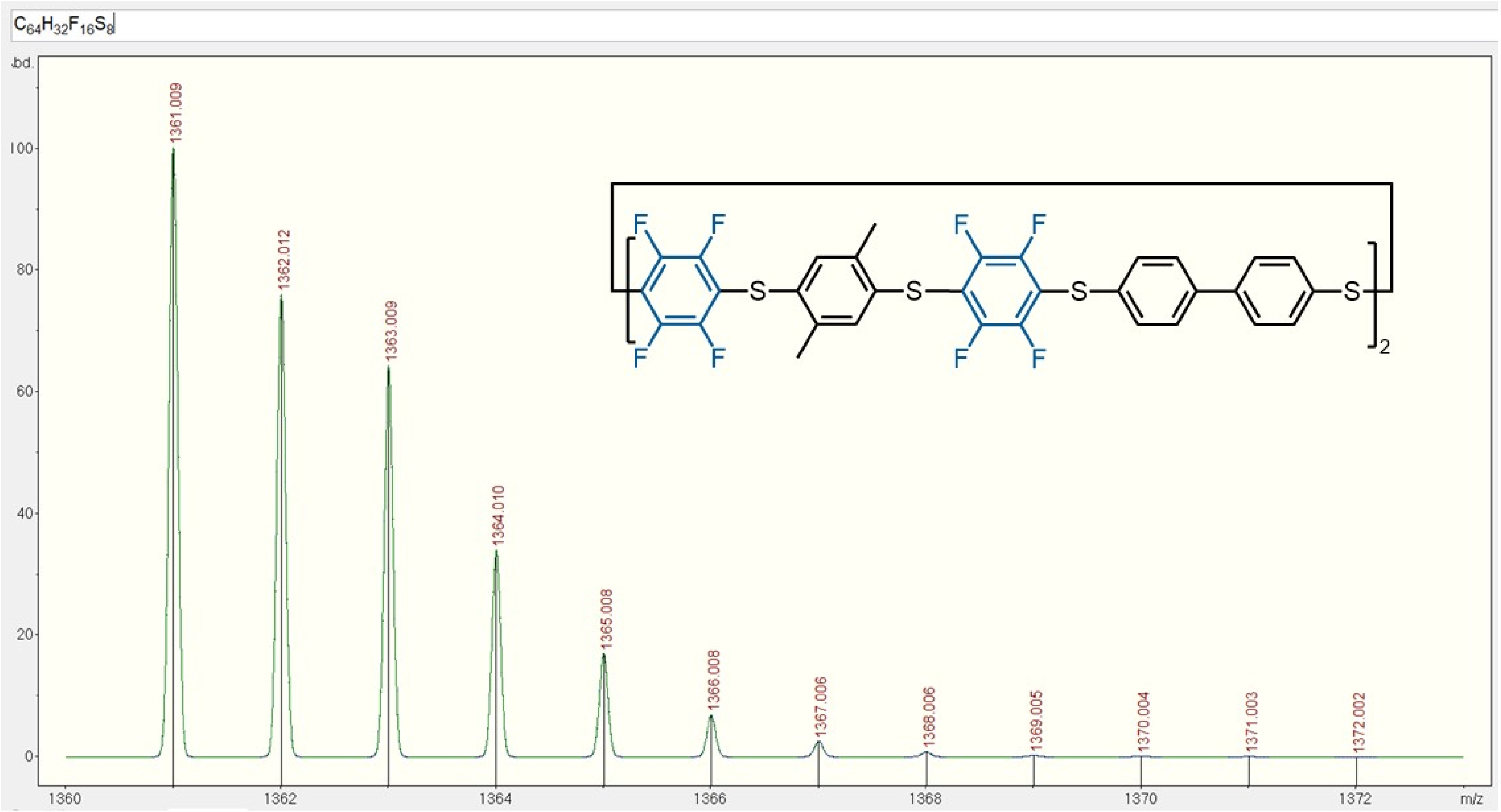

Figure S159. Simulated MALDI spectra of **1bd**.

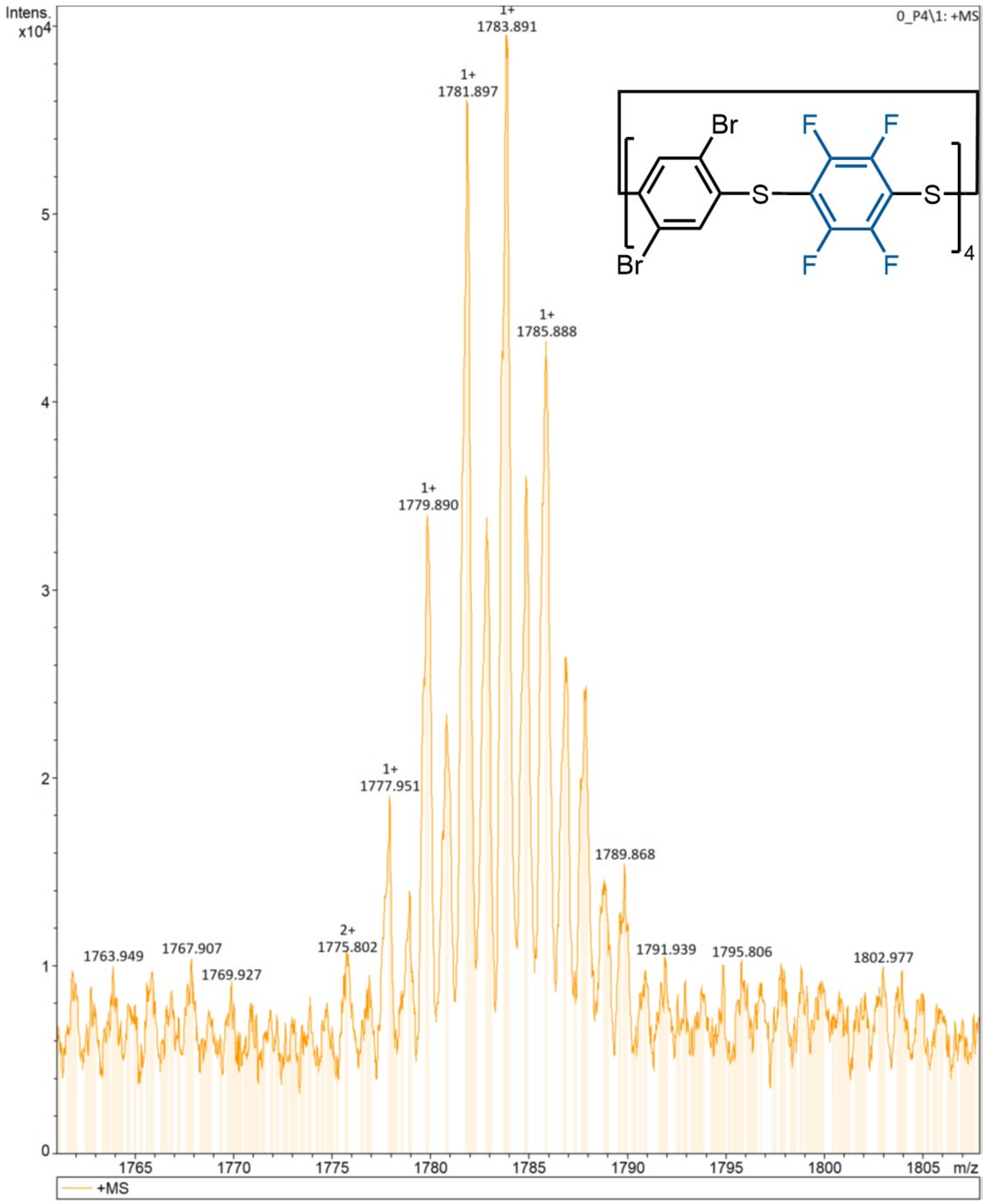

Figure S160. Zoomed in MALDI spectra of **1c** showing the expected isotope pattern.

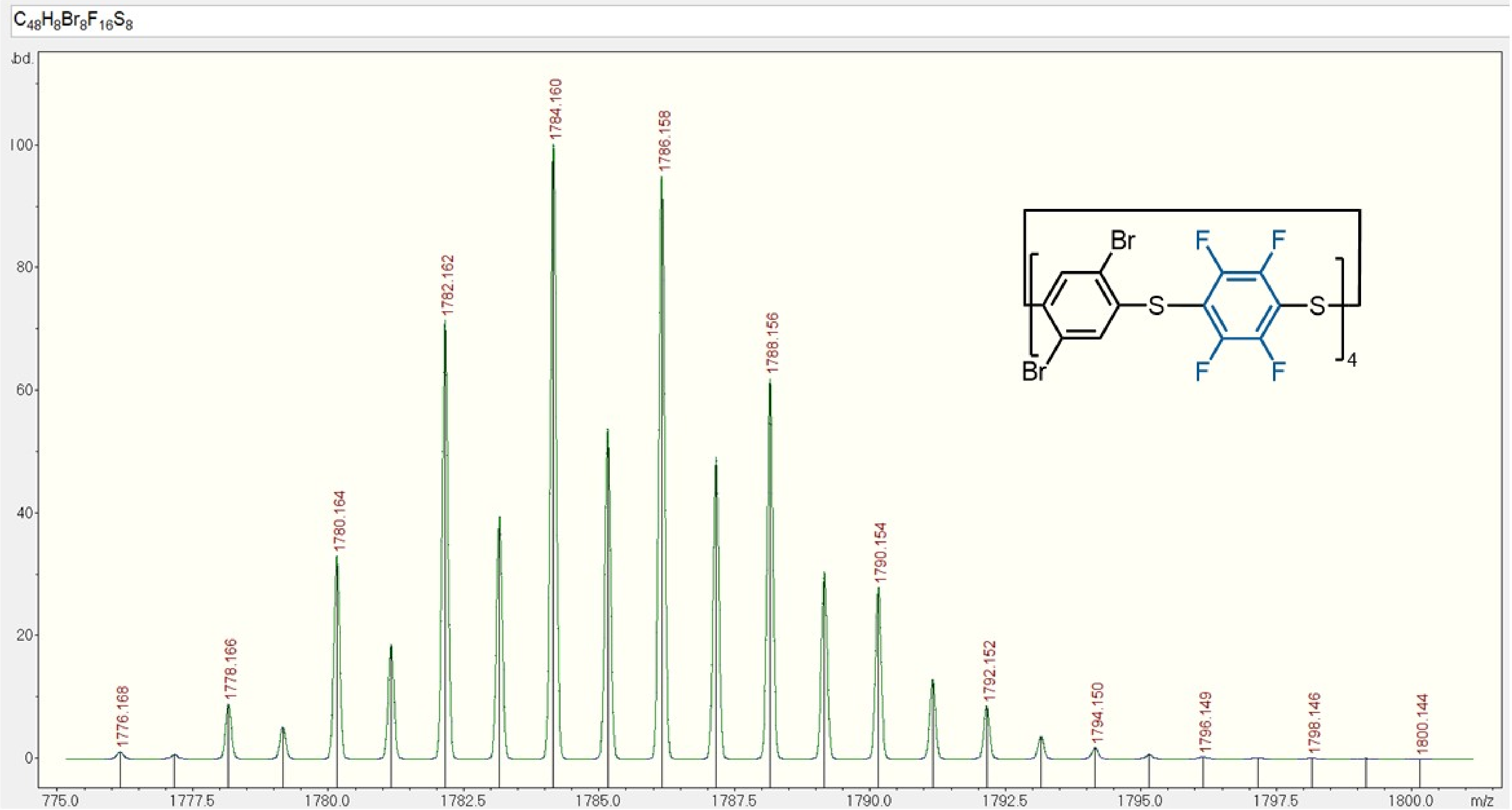

Figure S161. Simulated MALDI spectra of **1c**.

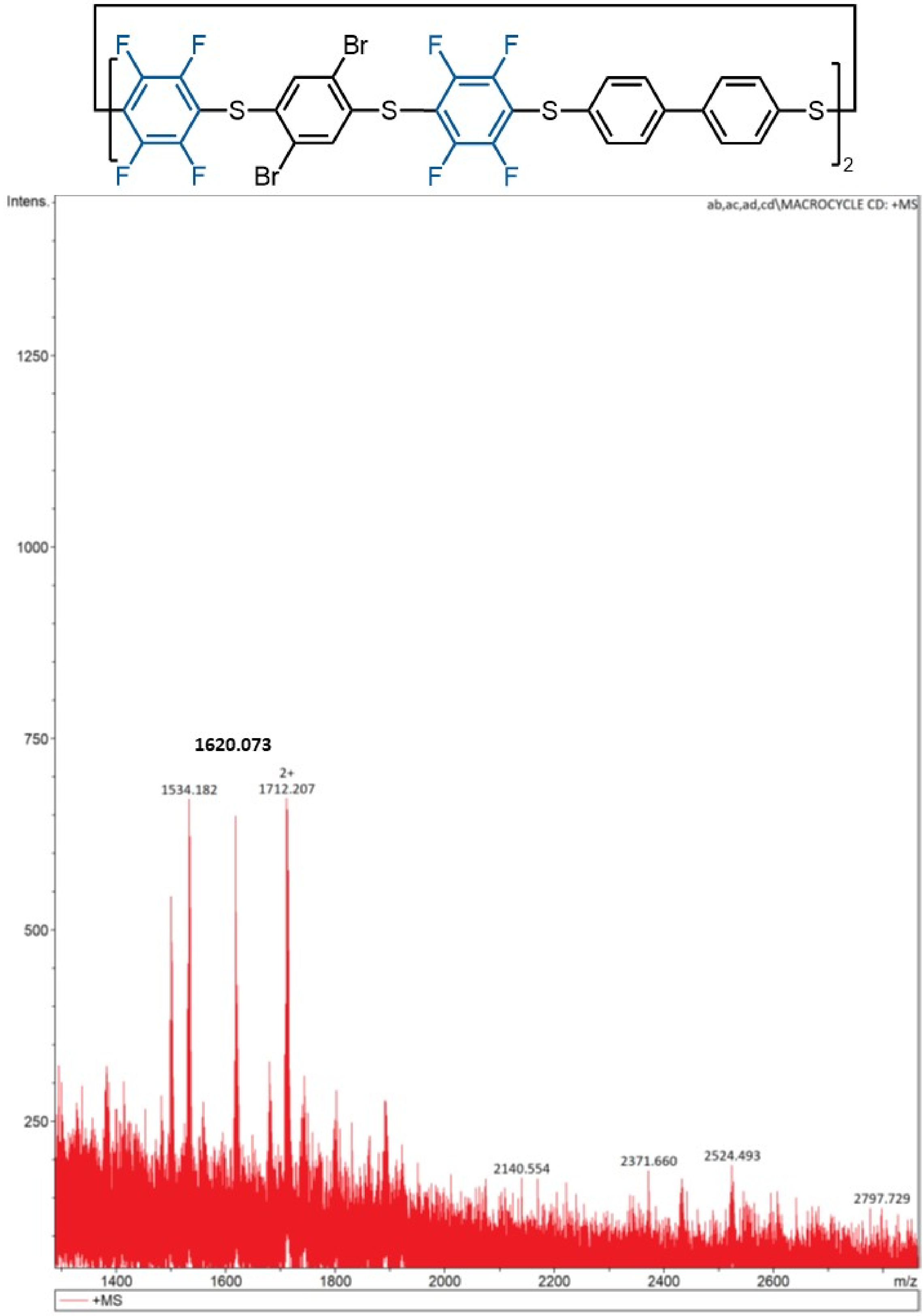

Figure S162. MALDI spectra of **1cd**.

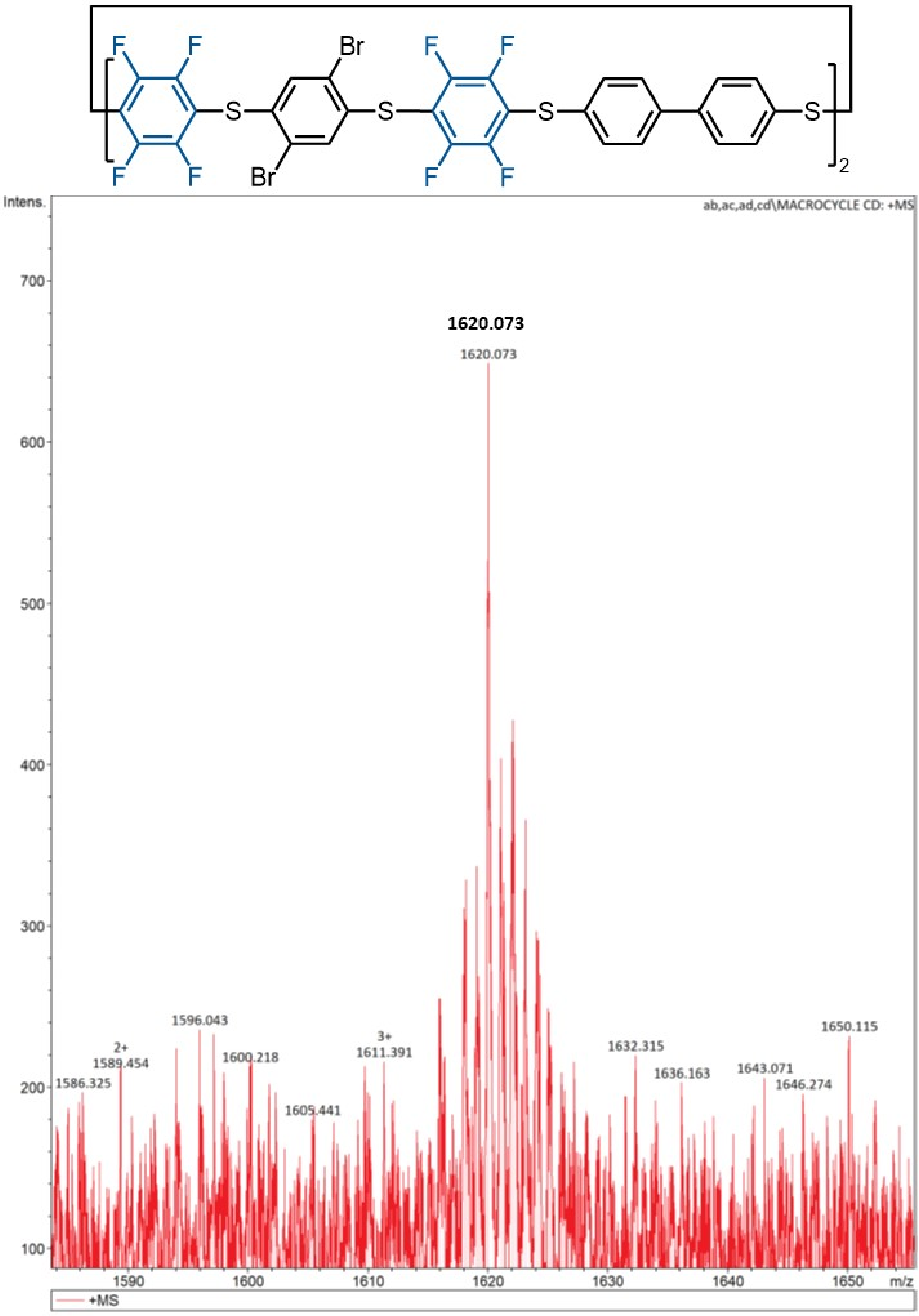

Figure S163. Zoomed in MALDI spectra of **1cd**, showing the expected isotope pattern.

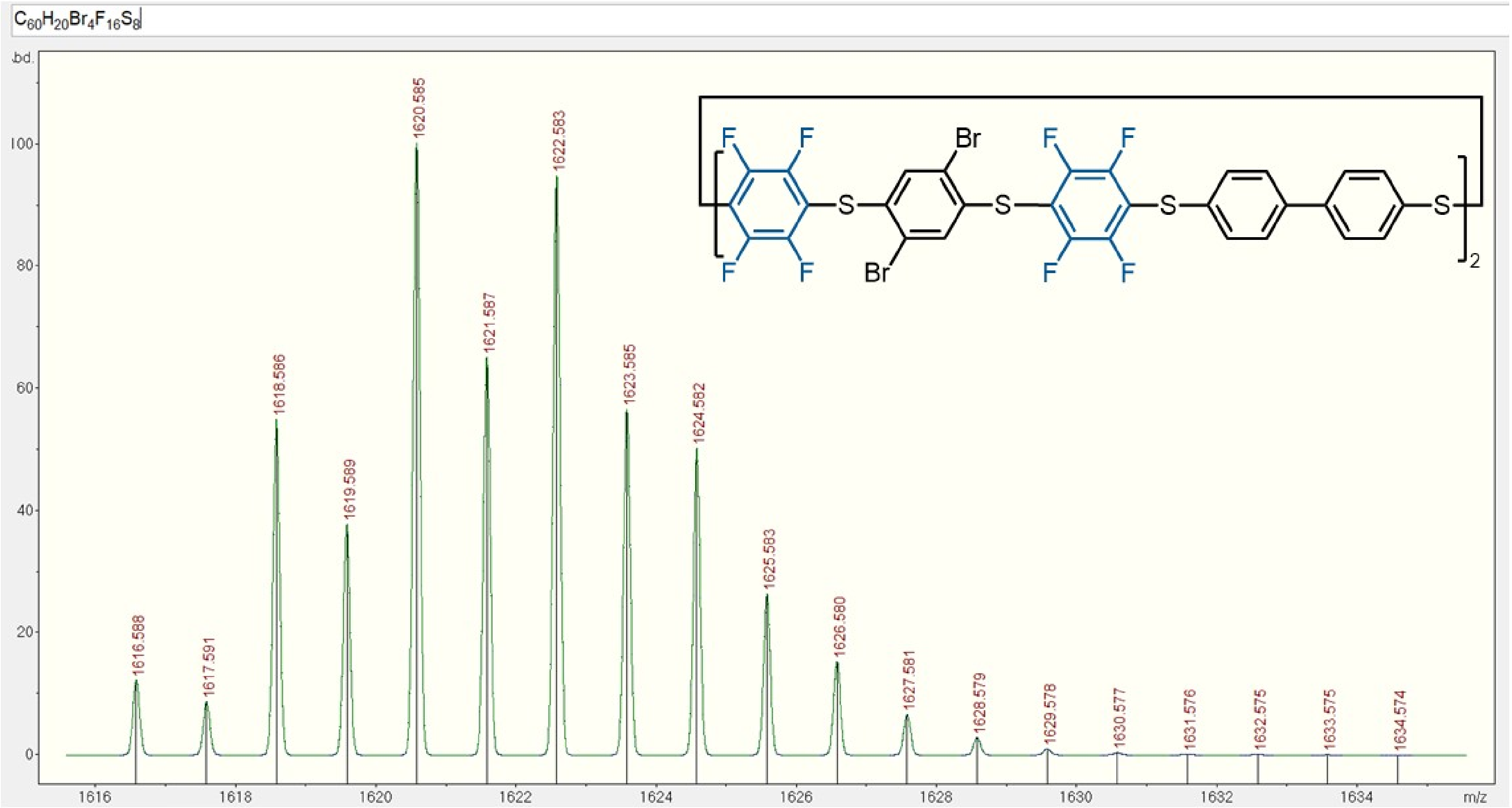

Figure S164. Simulated MALDI spectra of **1cd**.

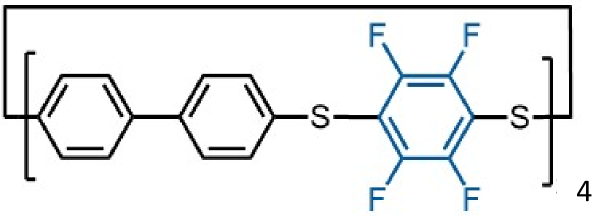

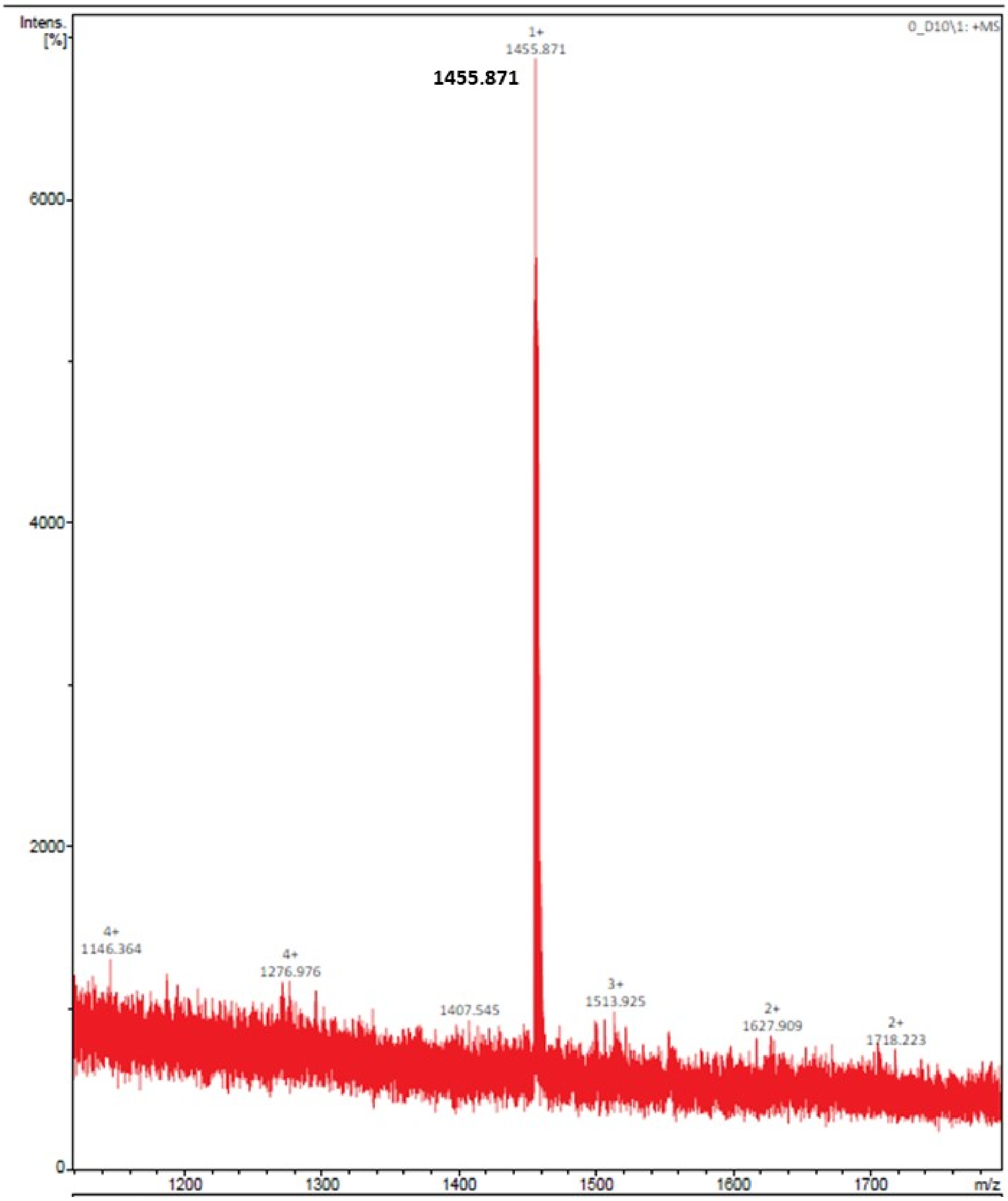

Figure S165. MALDI spectra of **1d**.

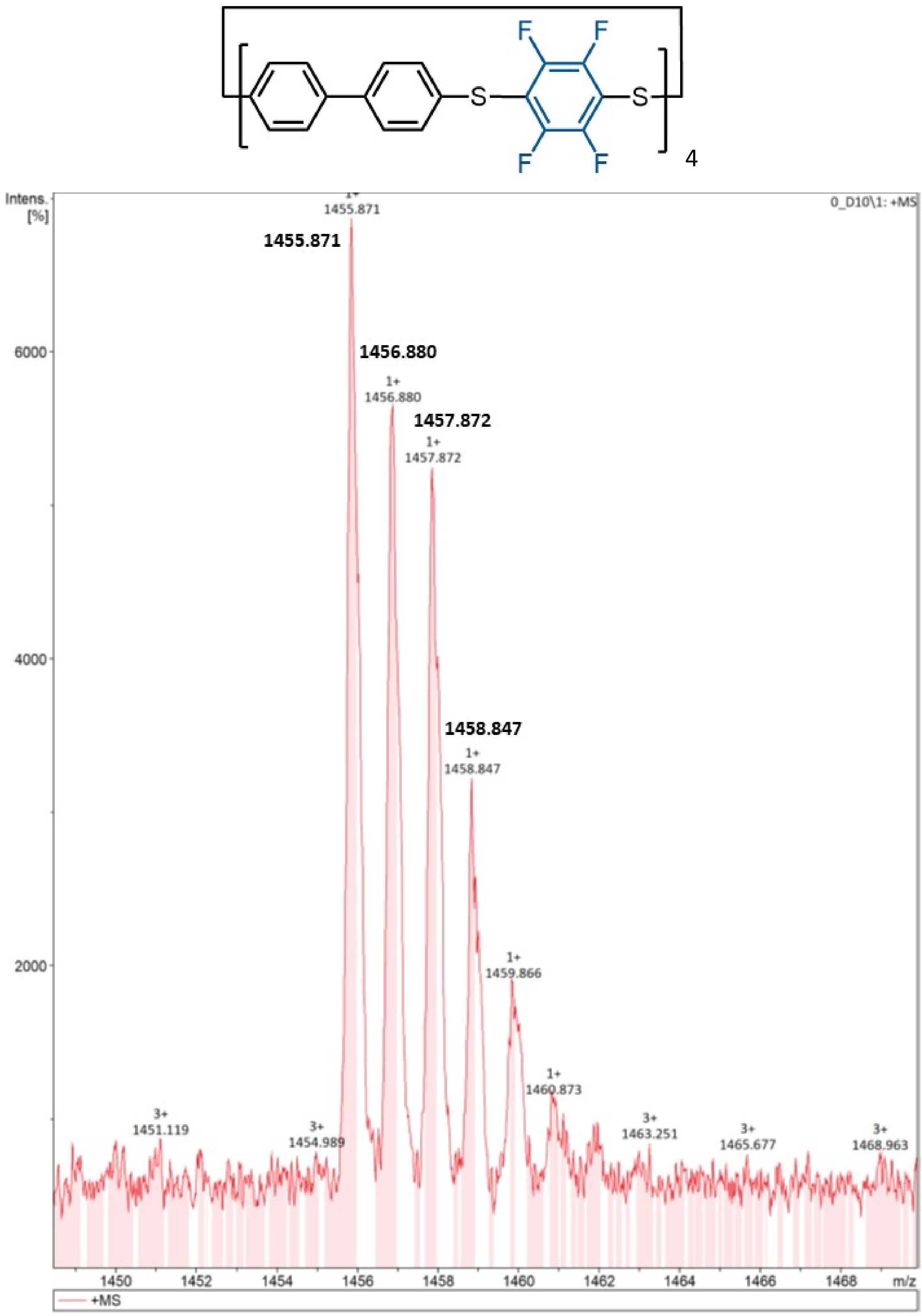

Figure S166. Zoomed in MALDI spectra of **1d**, showing the expected isotope pattern.

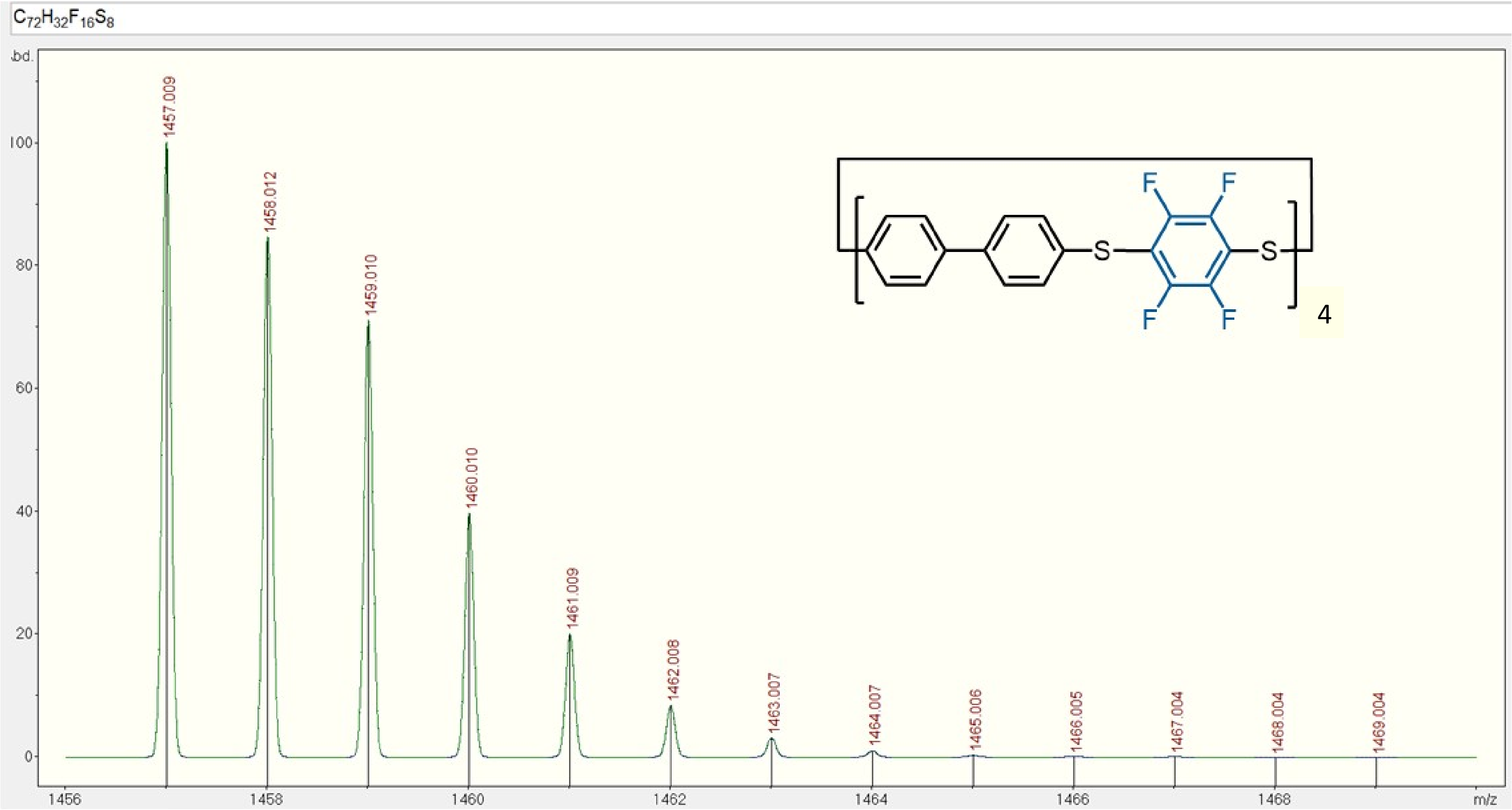

Figure S167. Simulated MALDI spectra of **1d**.

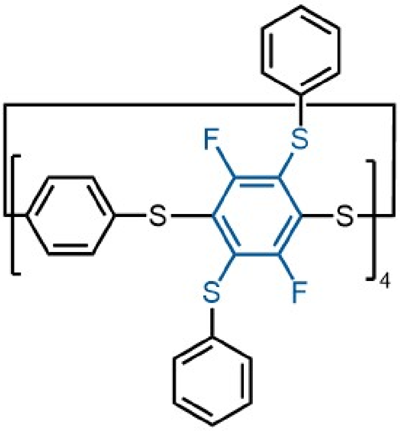

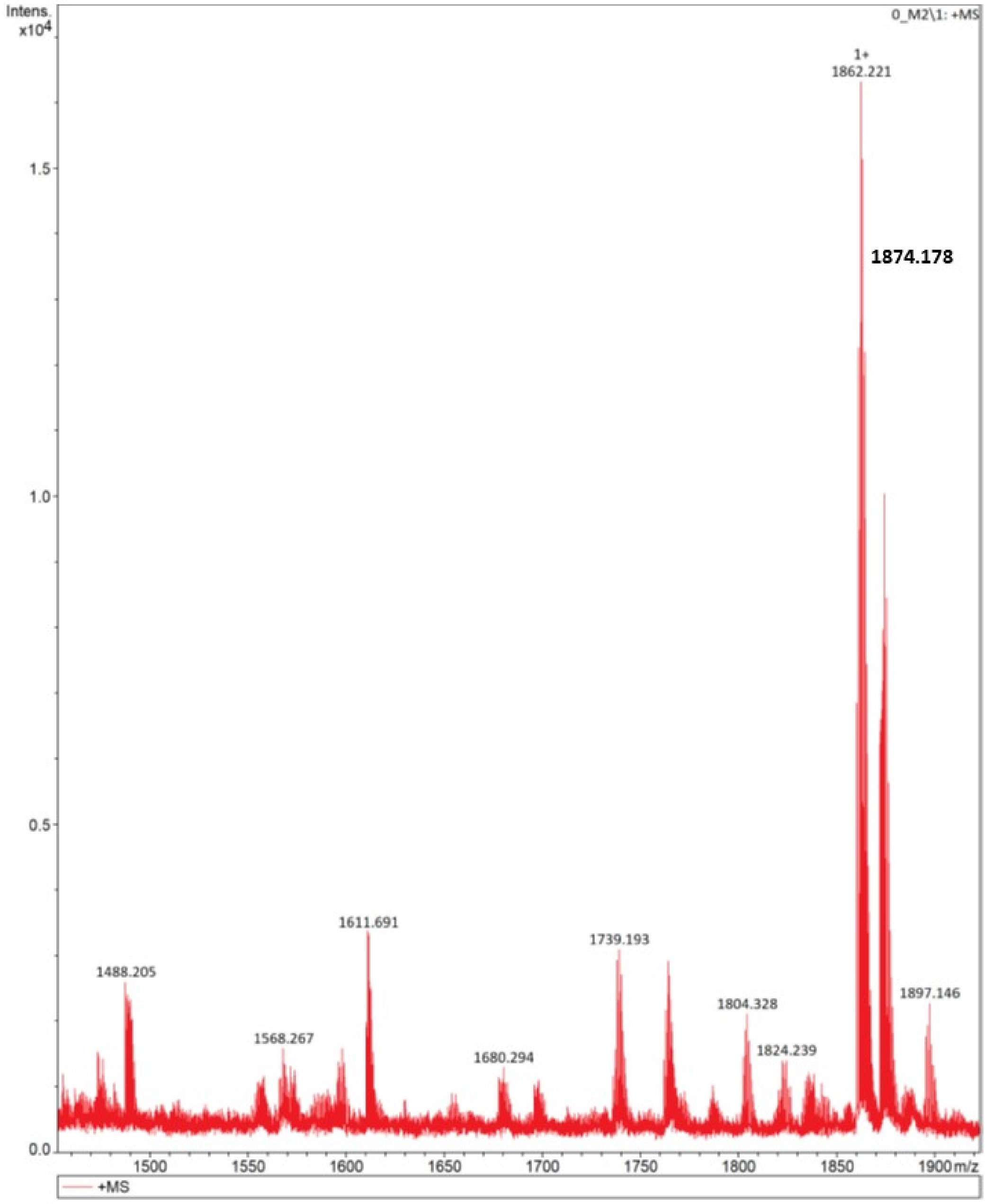

Figure S168. MALDI spectra of **4a**.

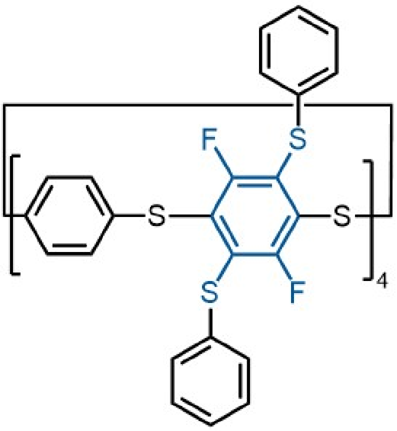

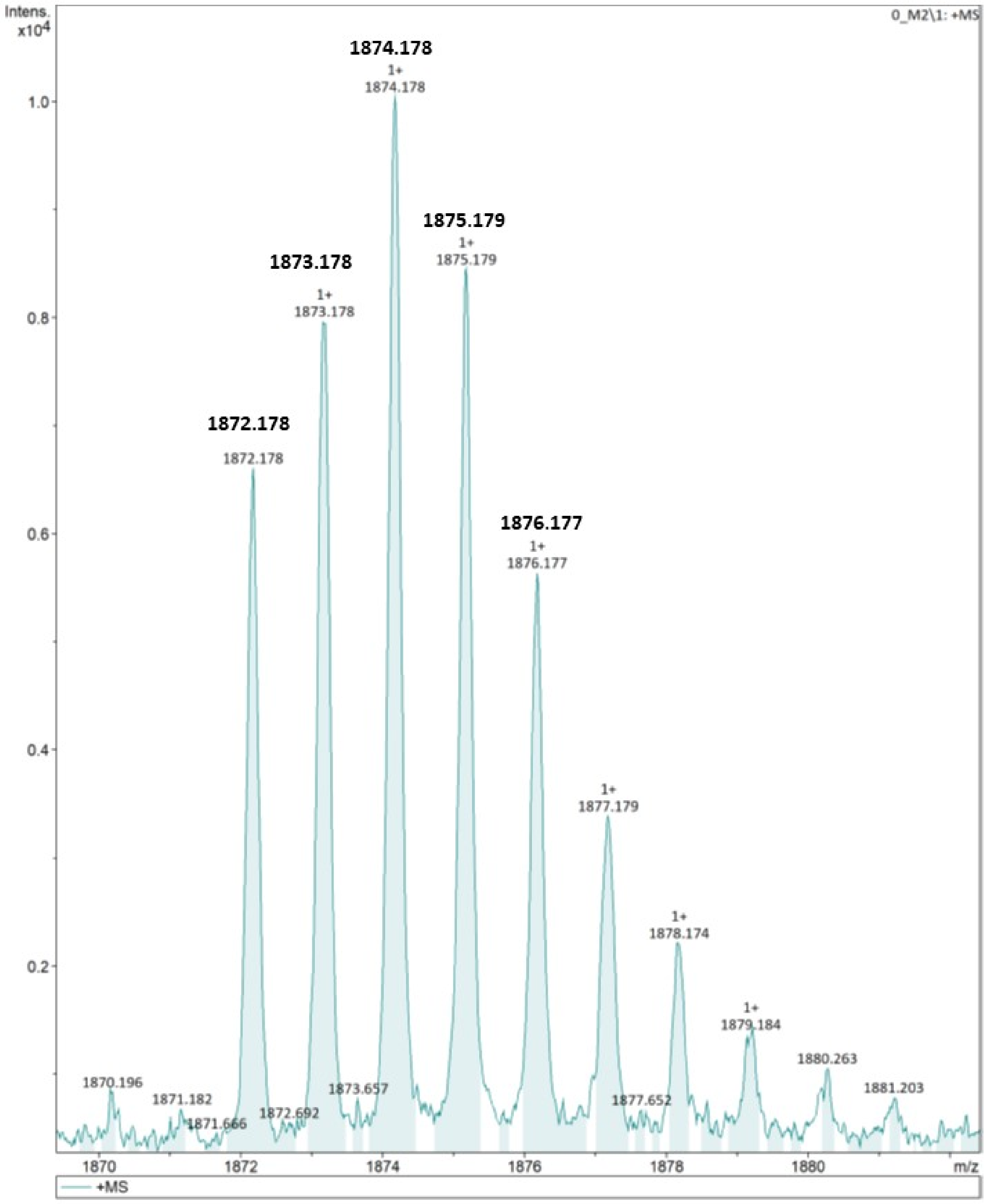

Figure S169. Zoomed in MALDI spectra of **4a**, showing the expected isotope pattern.

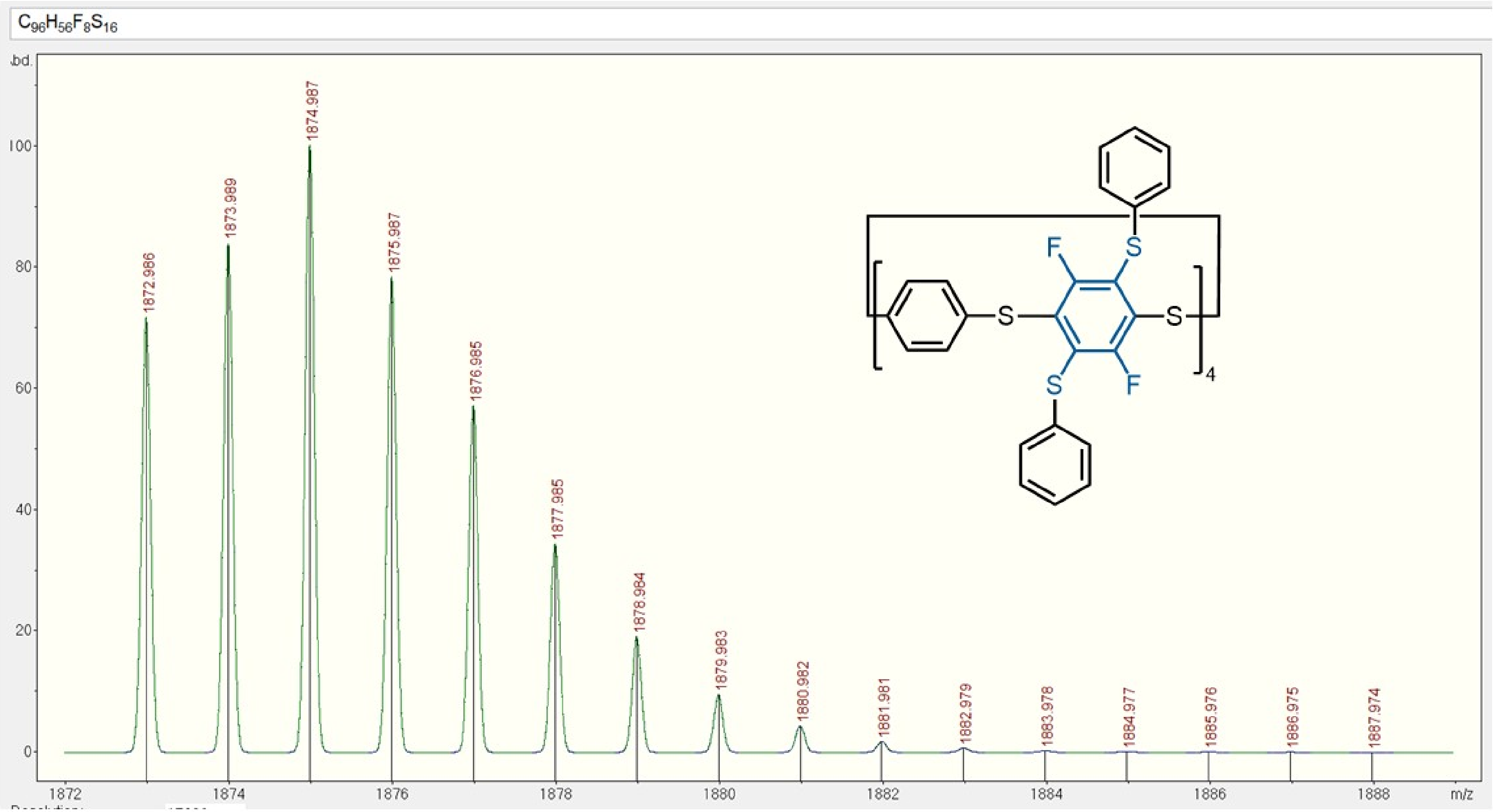

Figure S170. Simulated MALDI spectra of **4a**.

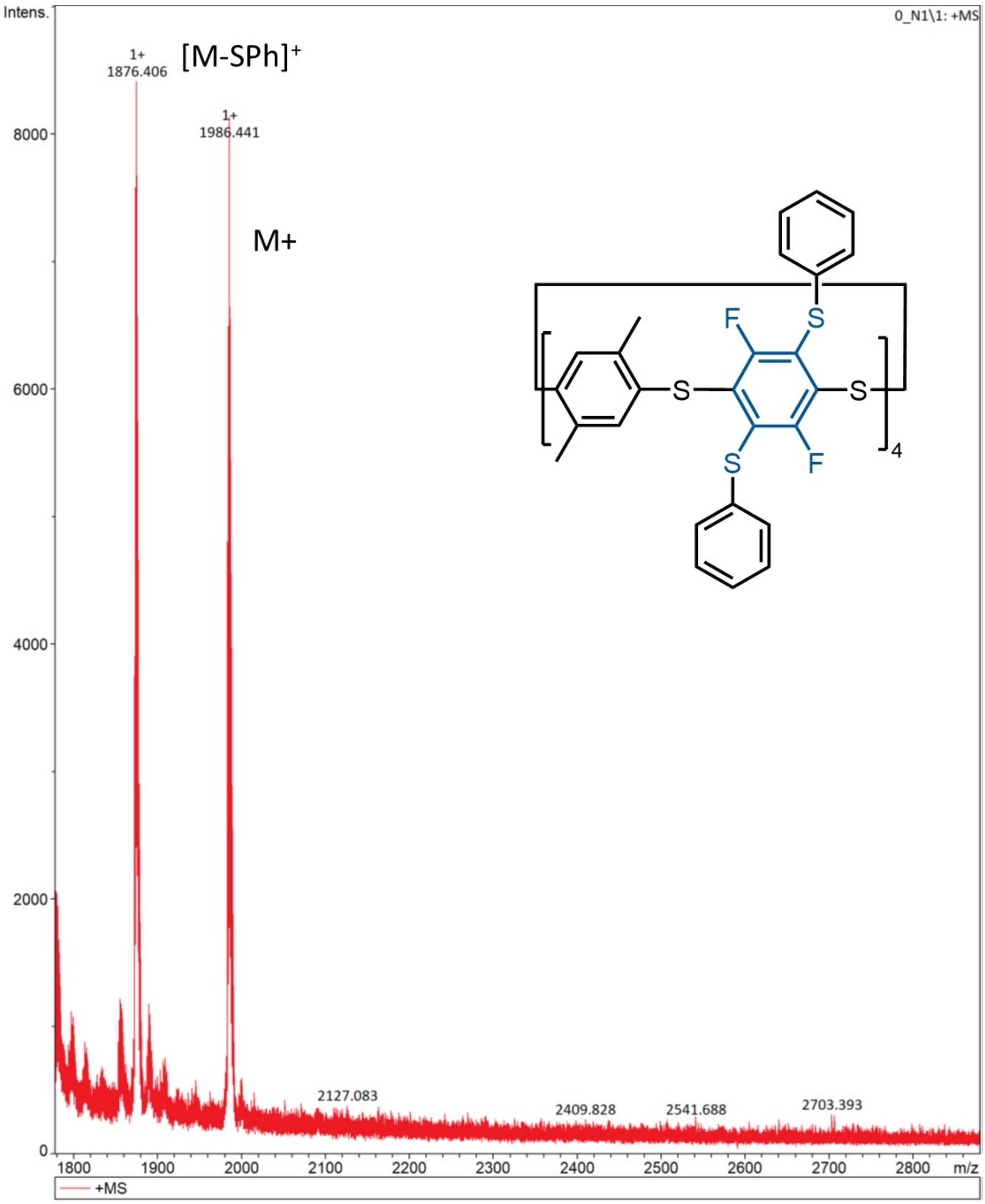

Figure S171. MALDI spectra of **4b**.

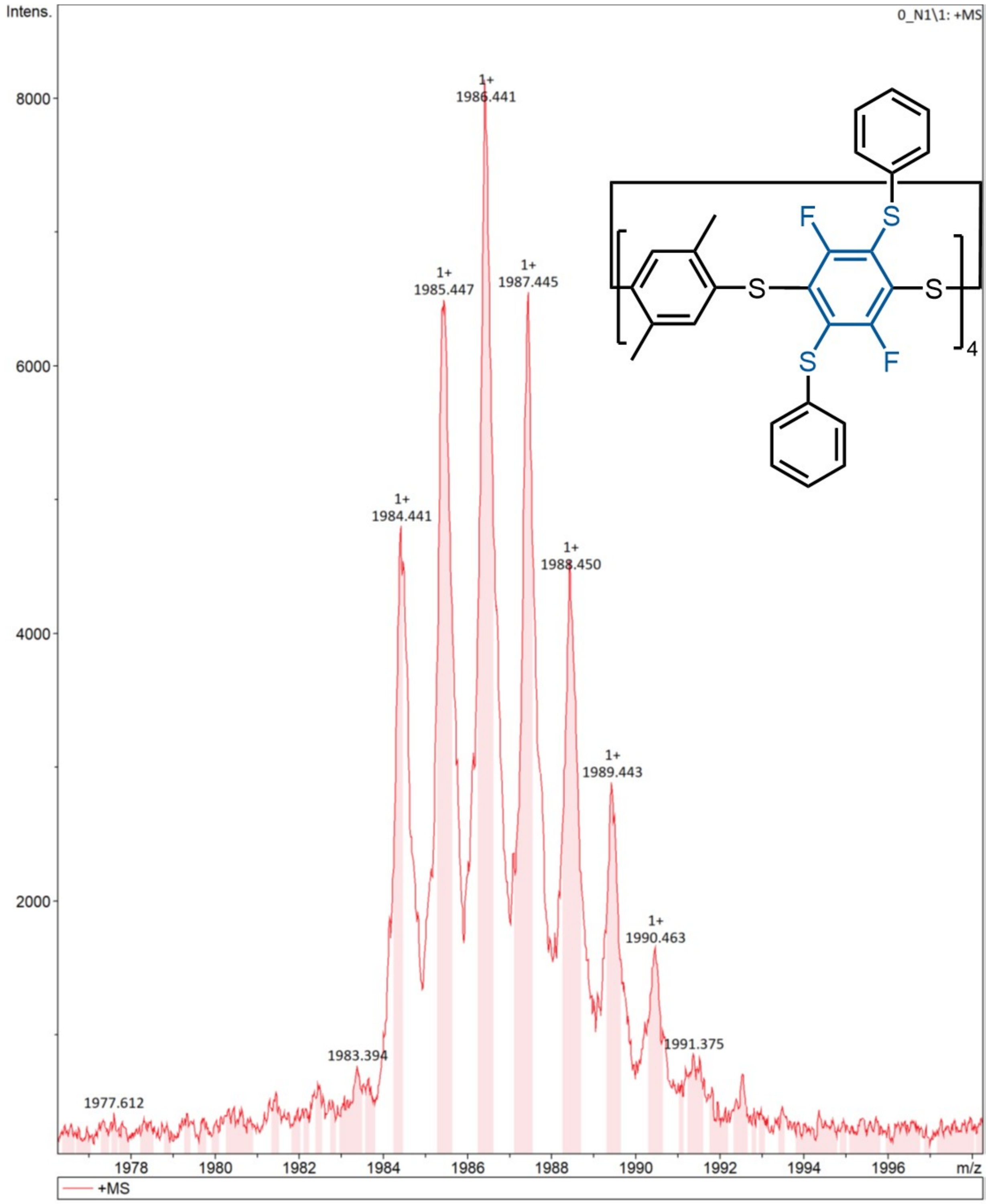

Figure S172. Zoomed in MALDI spectra of **4b** showing the expected splitting pattern.

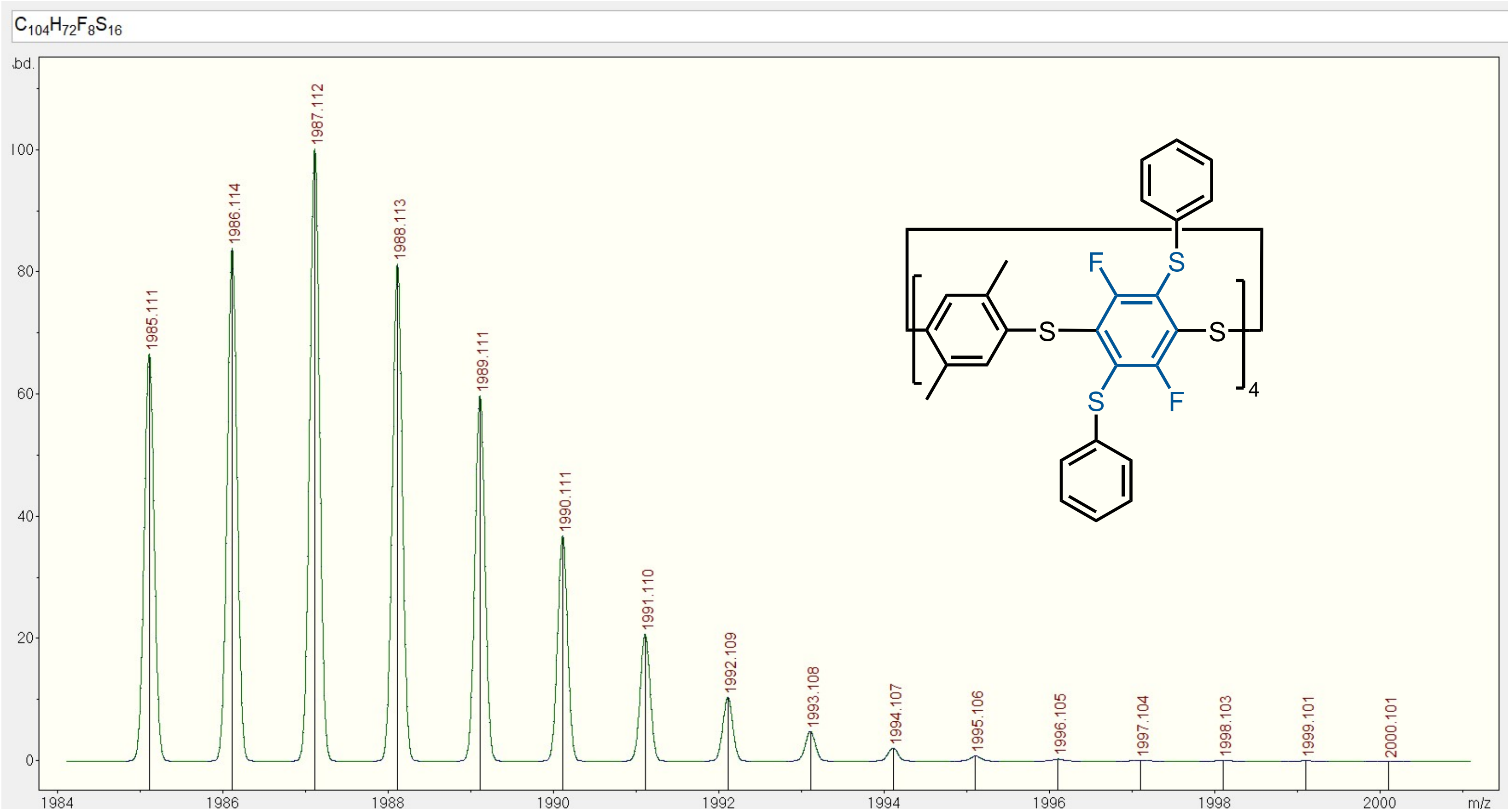

Figure S173. Simulated MALDI spectra of **4b**.

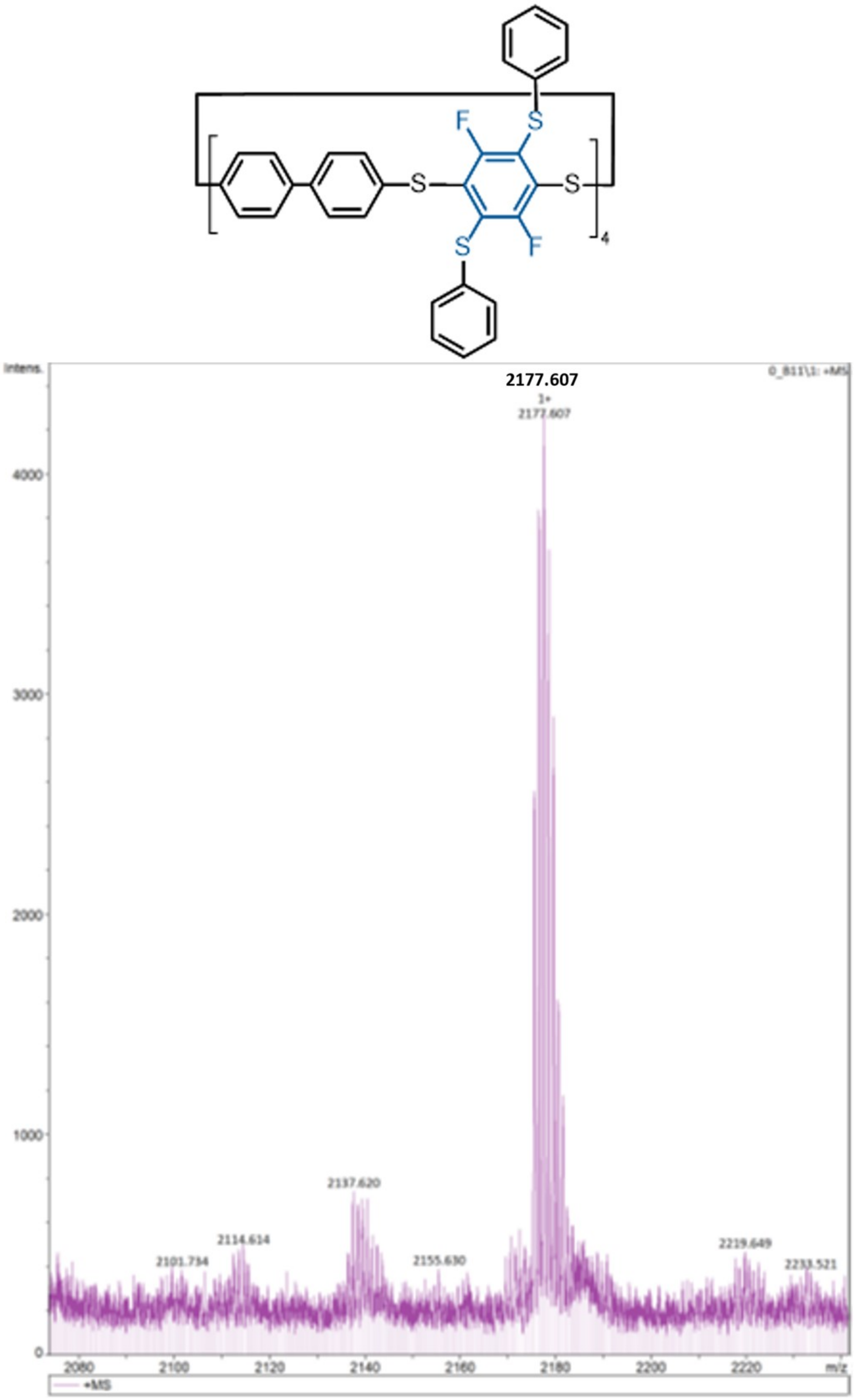

Figure S174. MALDI spectra of **4d**, showing the expected isotope pattern.

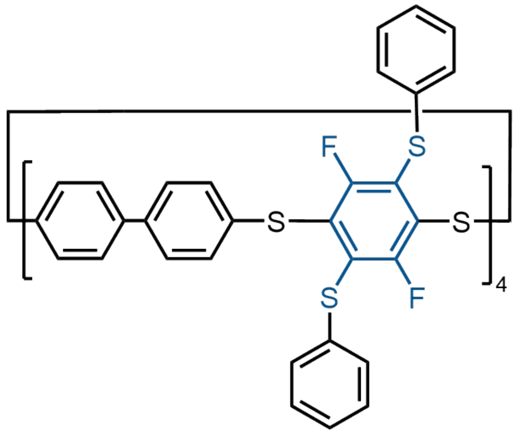

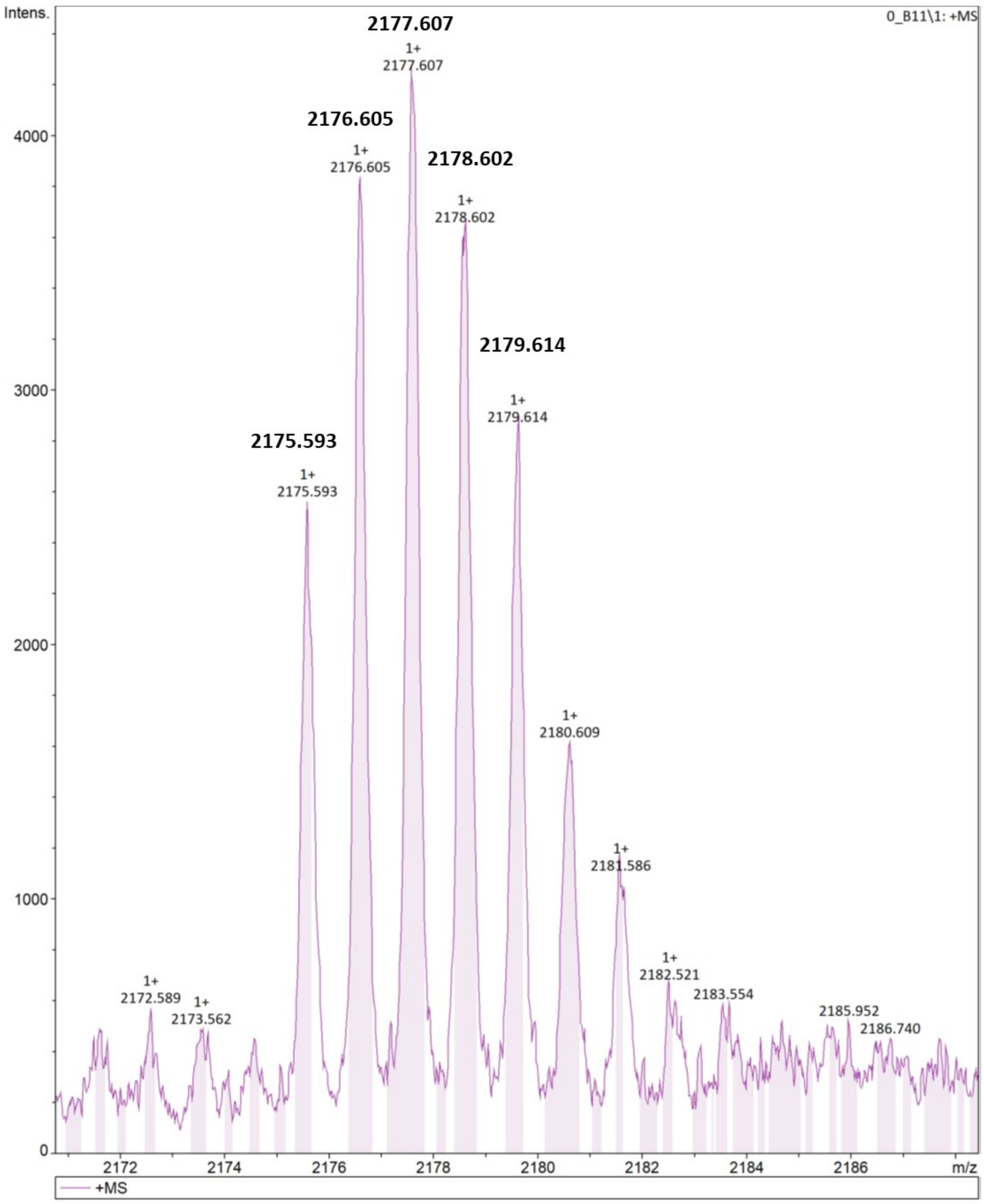

Figure S175. Zoomed in MALDI spectra of **4d**, showing the expected isotope pattern.

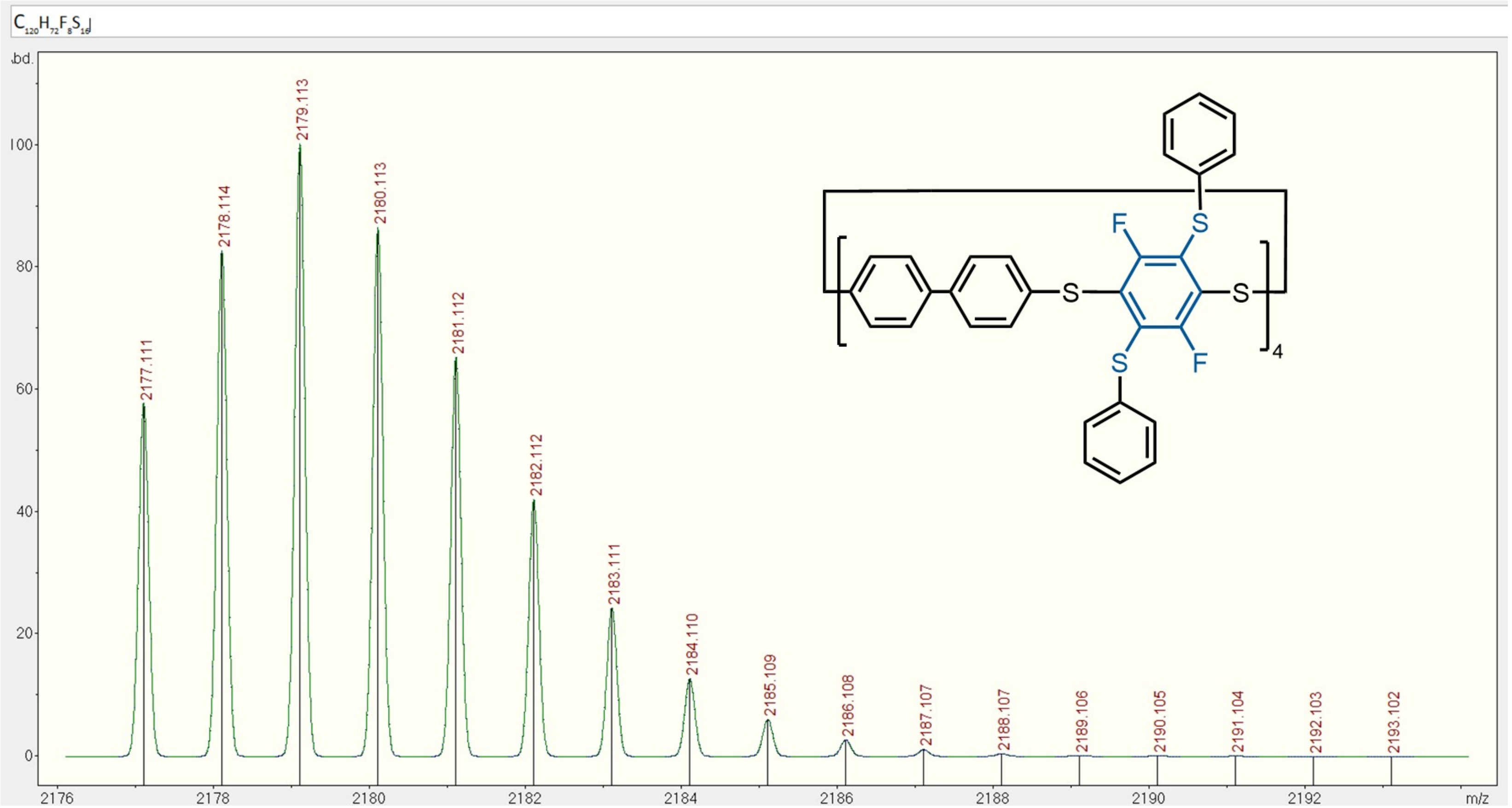

Figure S176. Simulated MALDI spectra of **4d**.

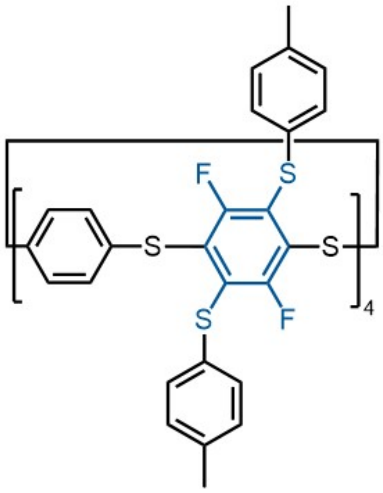

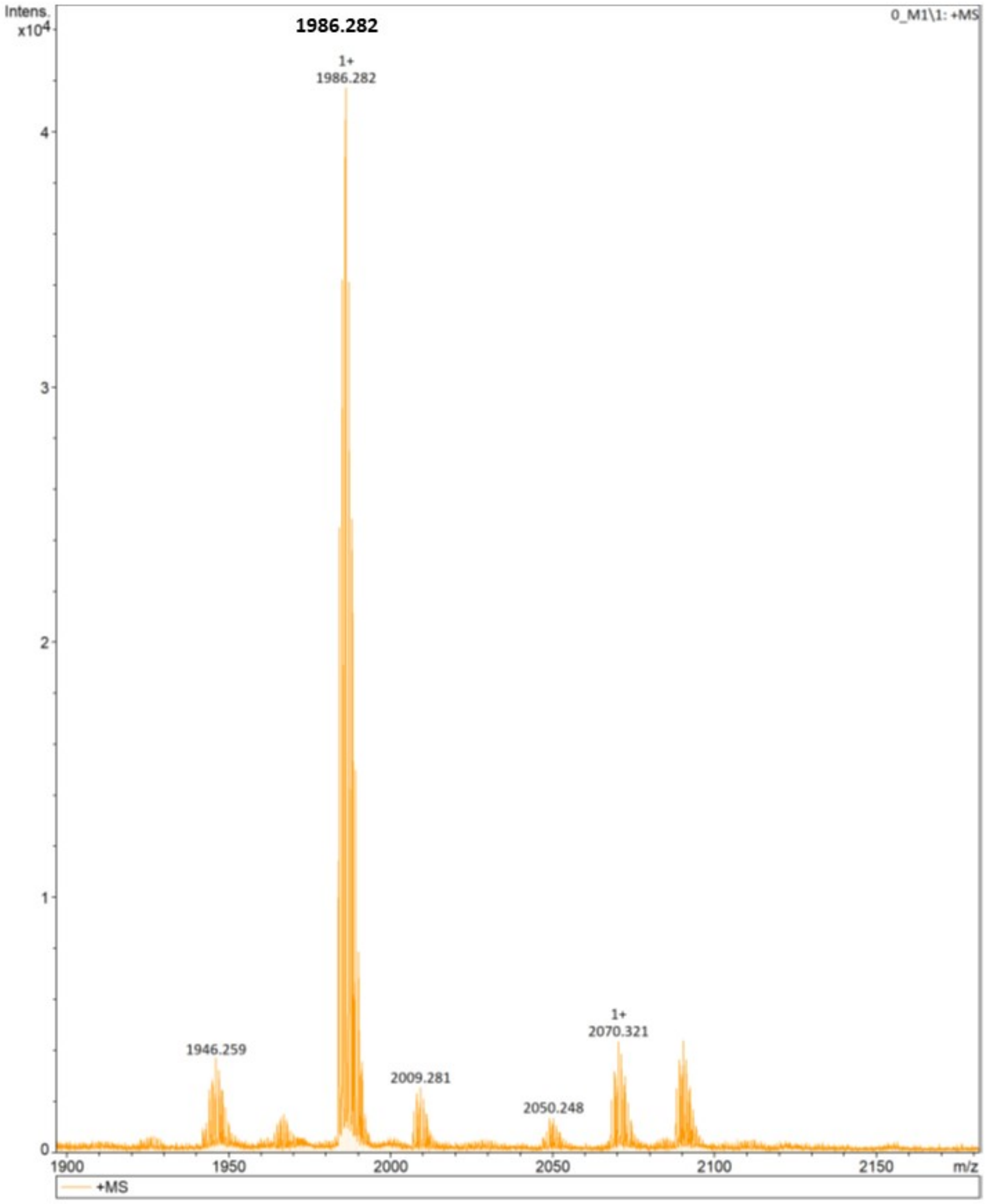

Figure S177. MALDI spectra of **5a**.

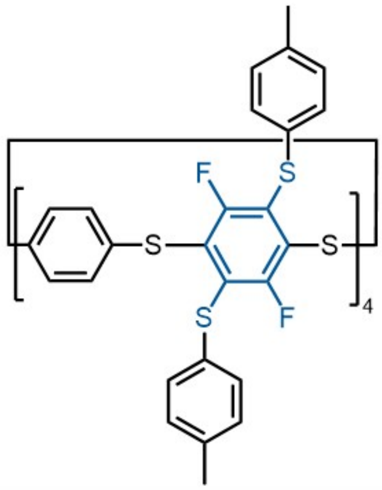

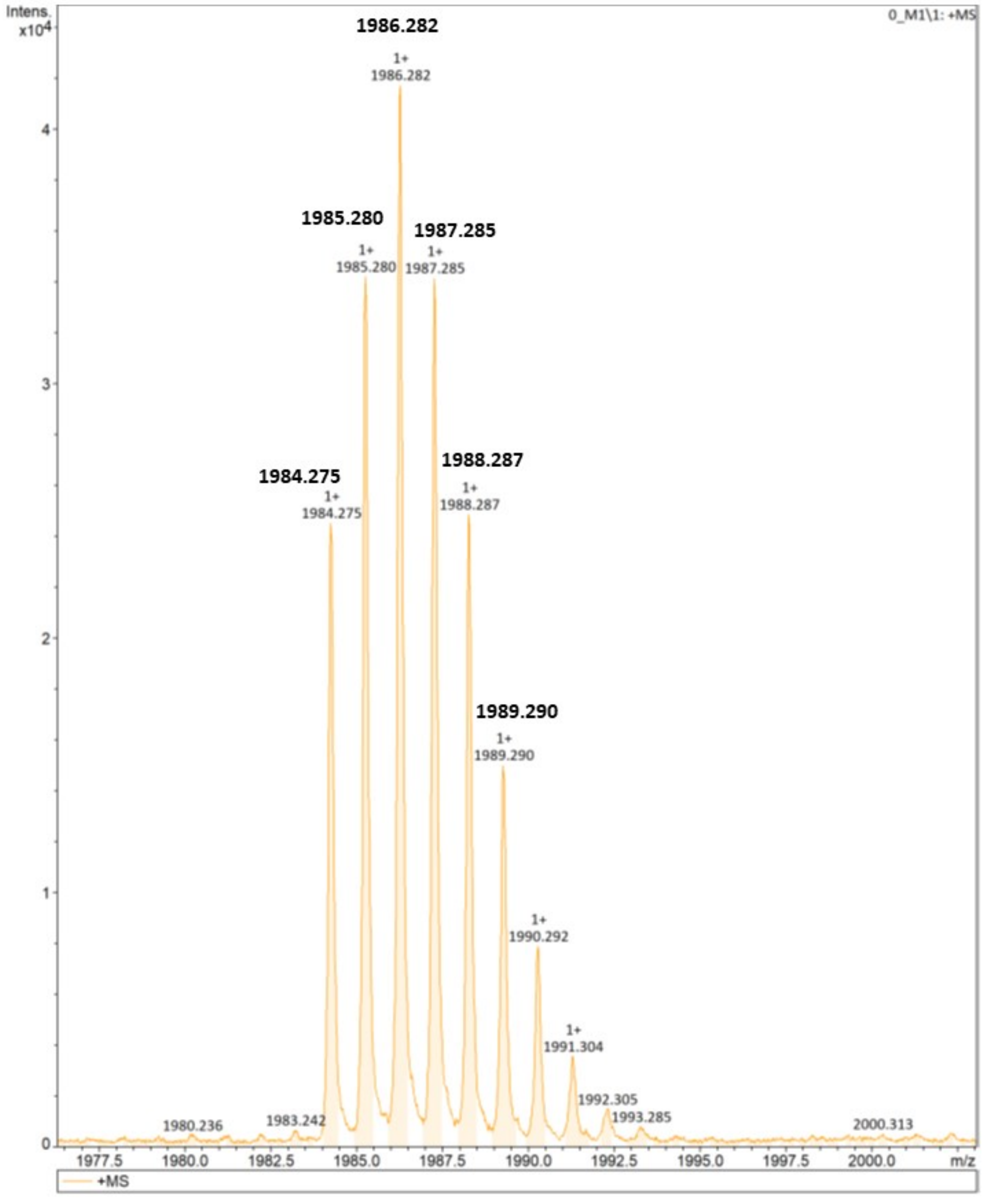

Figure S178. Zoomed in MALDI spectra of **5a**, showing the expected isotope pattern.

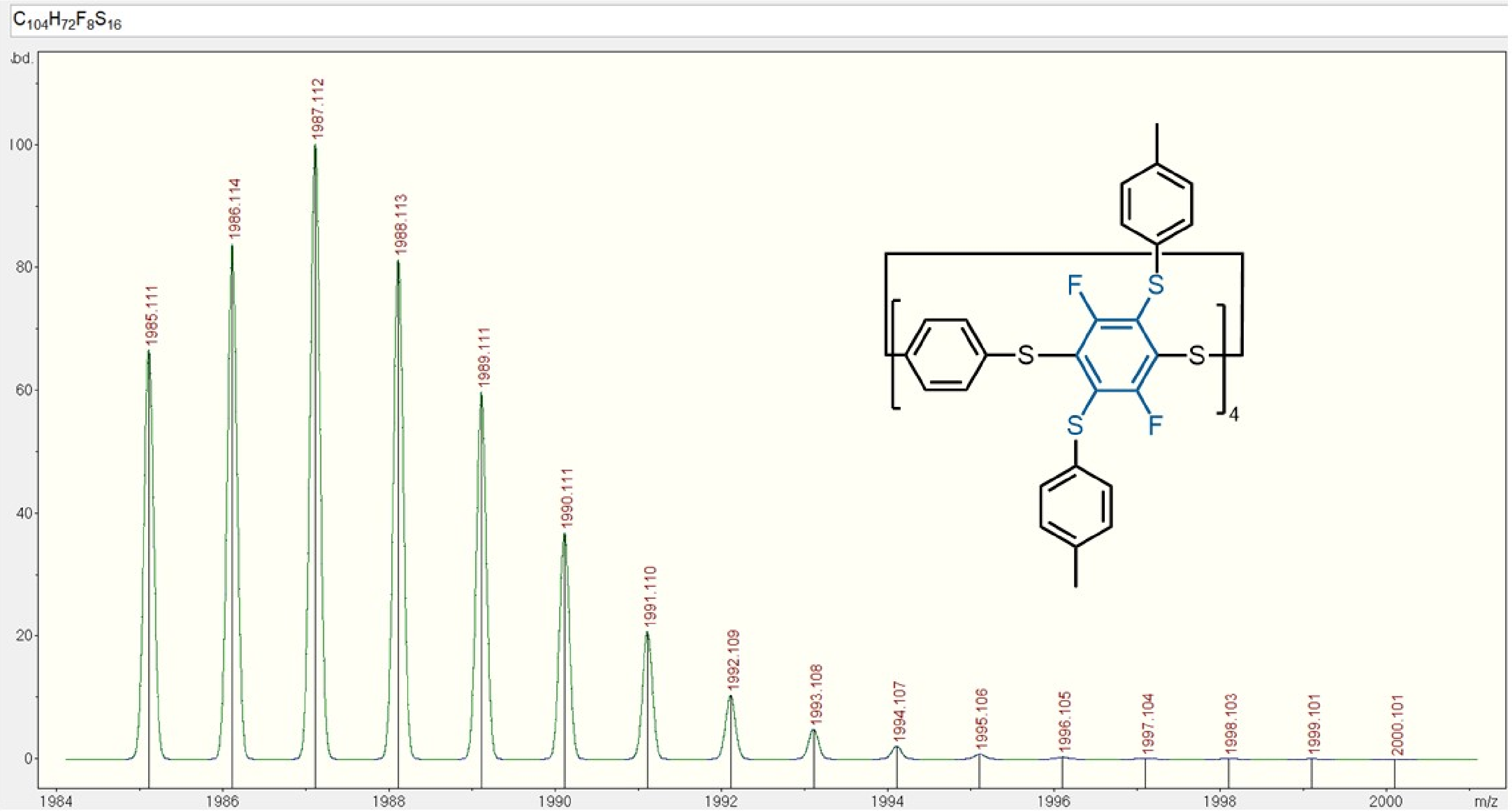

Figure S179. Simulated MALDI spectra of **5a**.

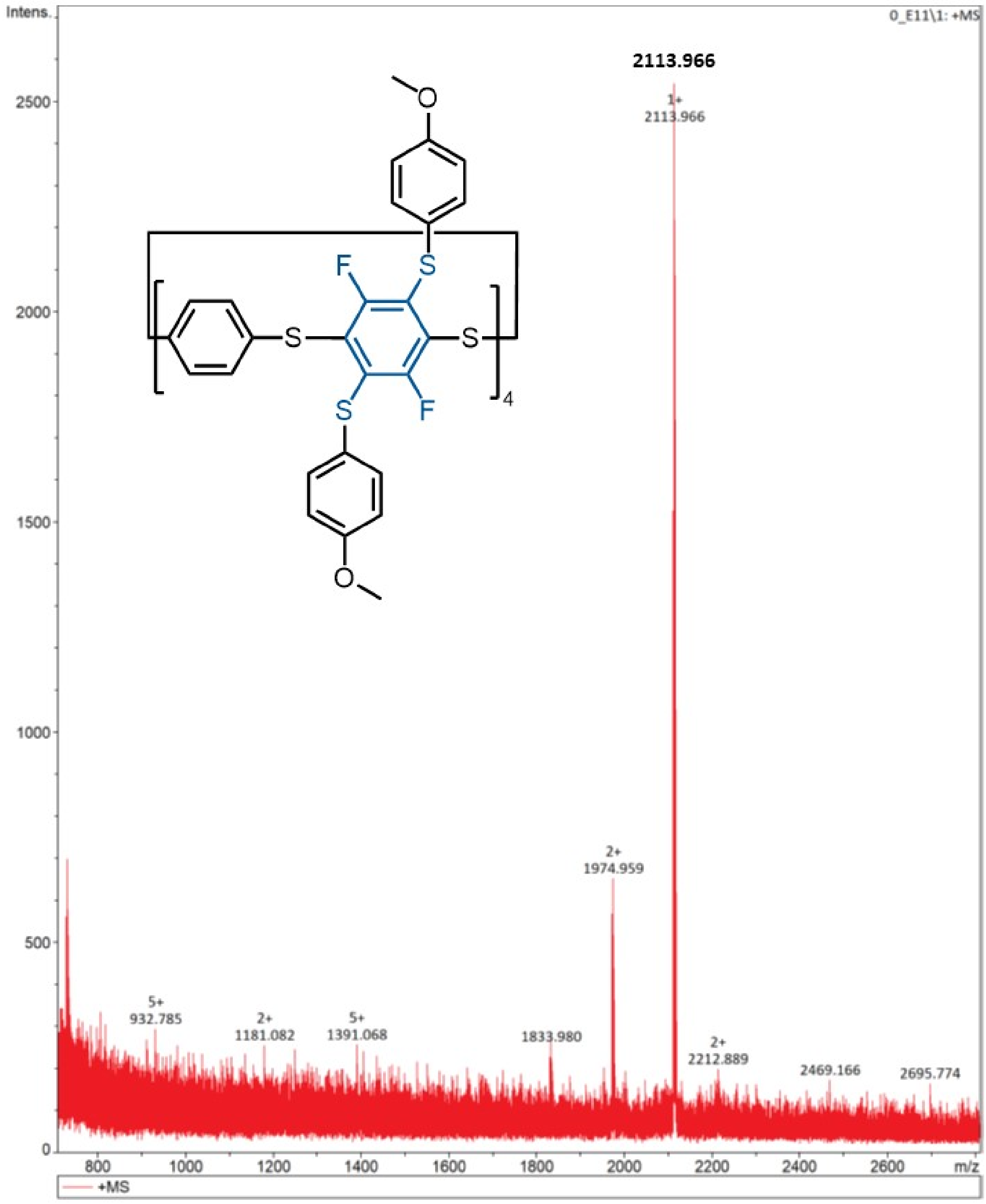

Figure S180. MALDI spectra of **5b**.

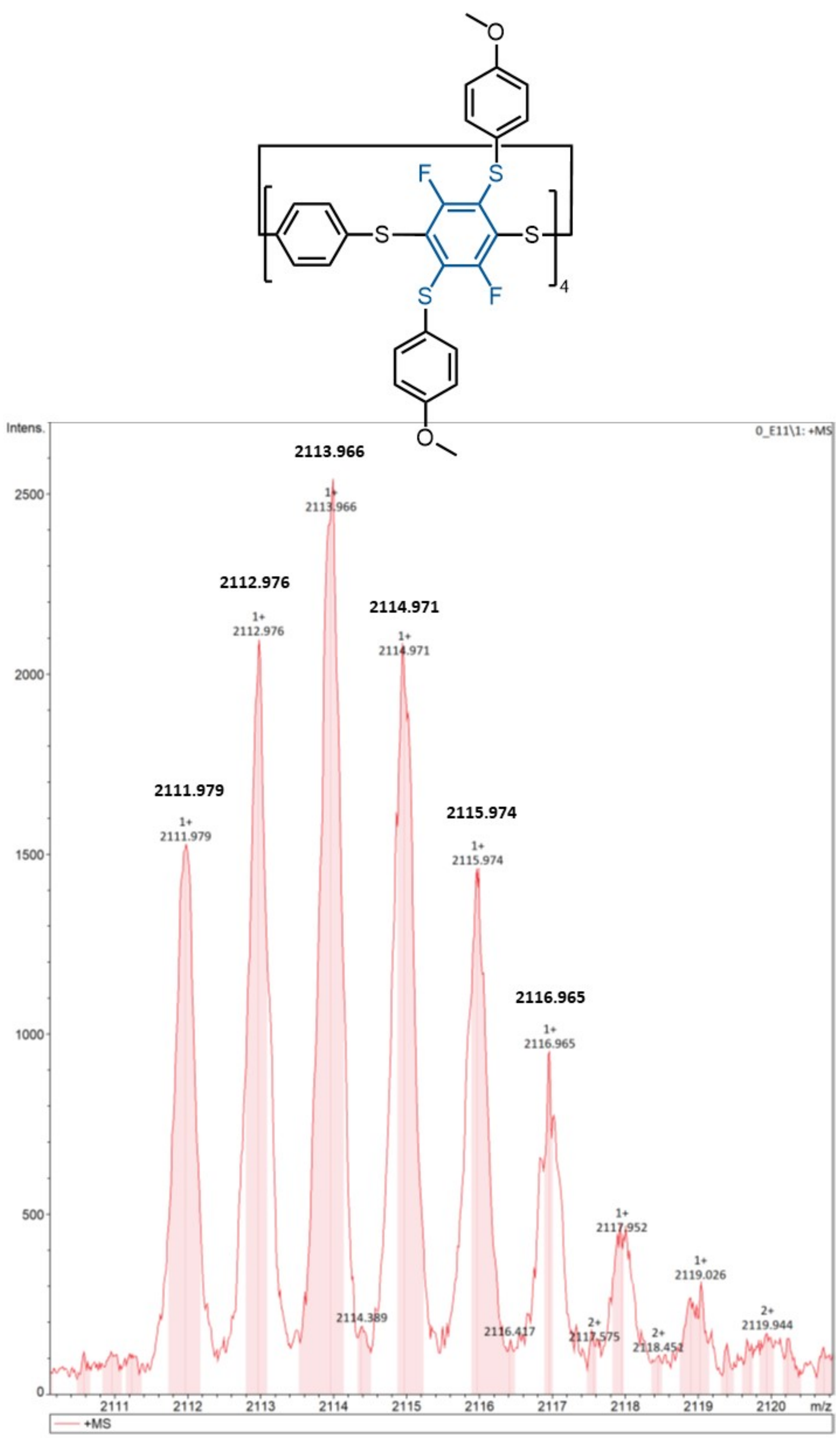

Figure S181. Zoomed in MALDI spectra of **5b** showing the expected isotope pattern.

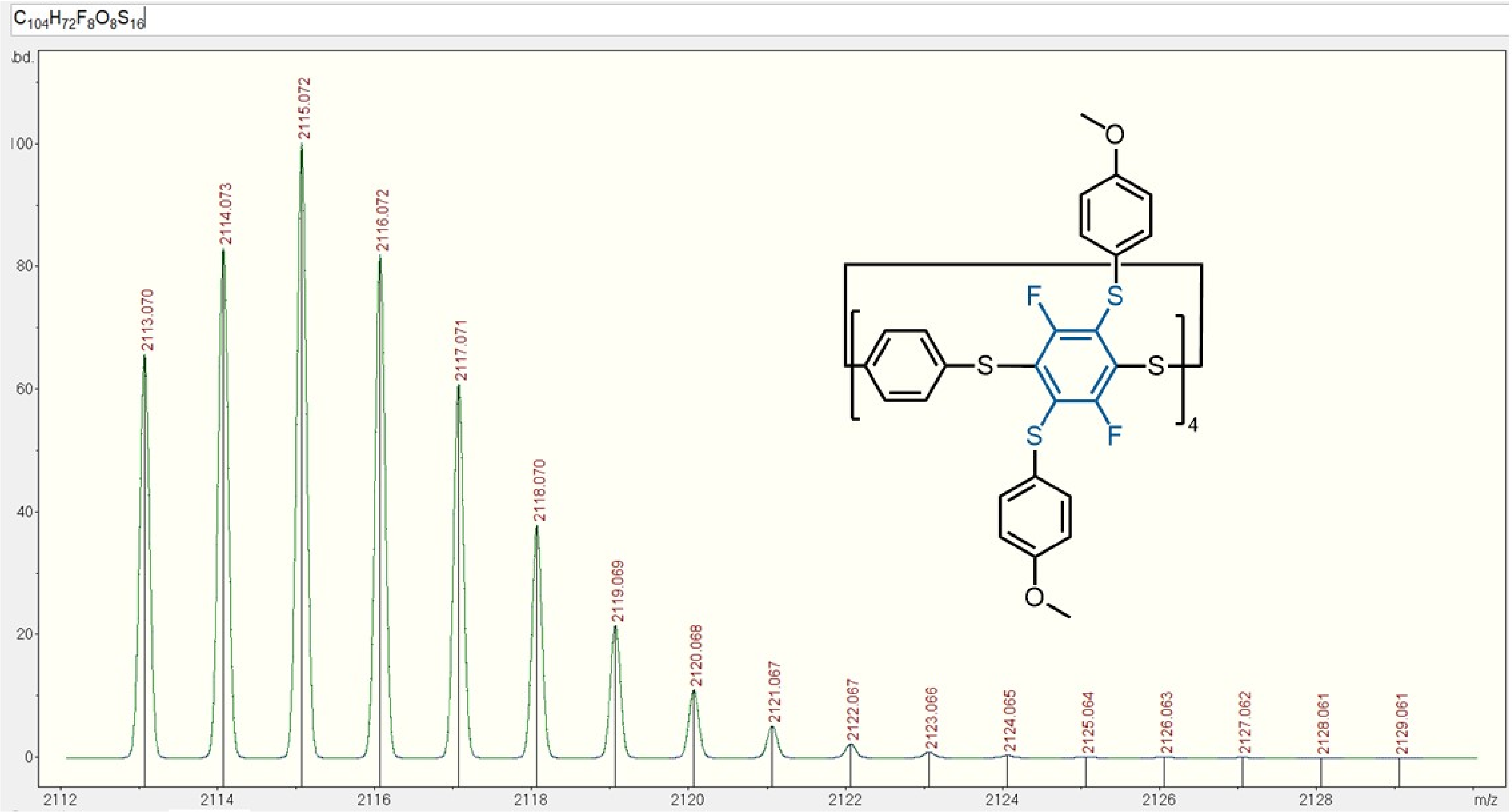

Figure S182. Simulated MALDI spectra of **5b**.

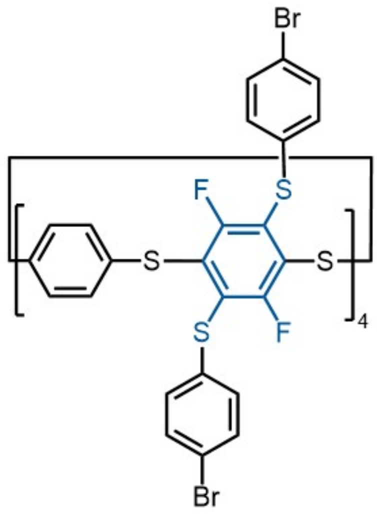

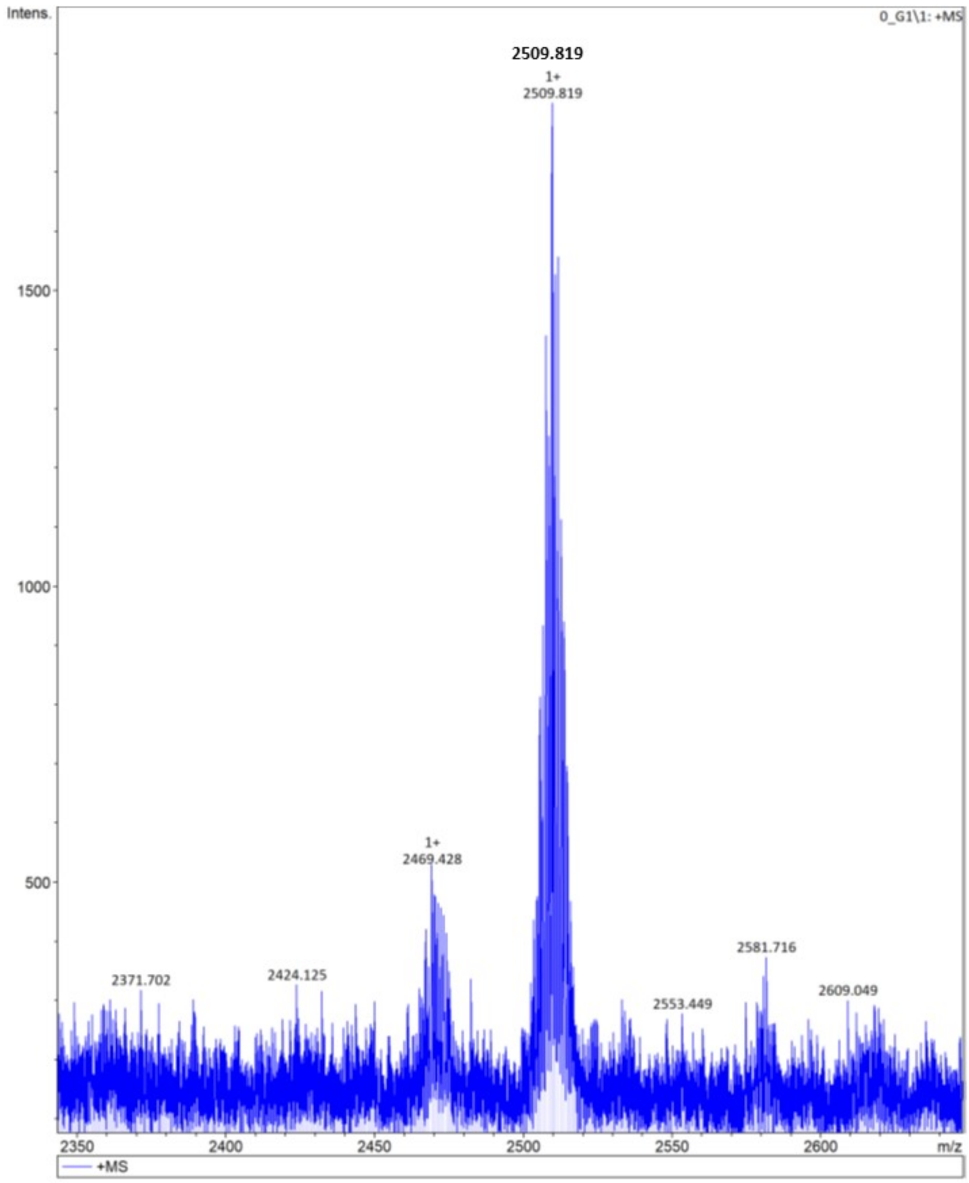

Figure S183. MALDI spectra of **5c**.

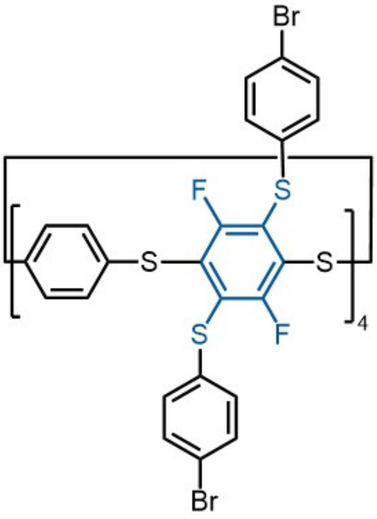

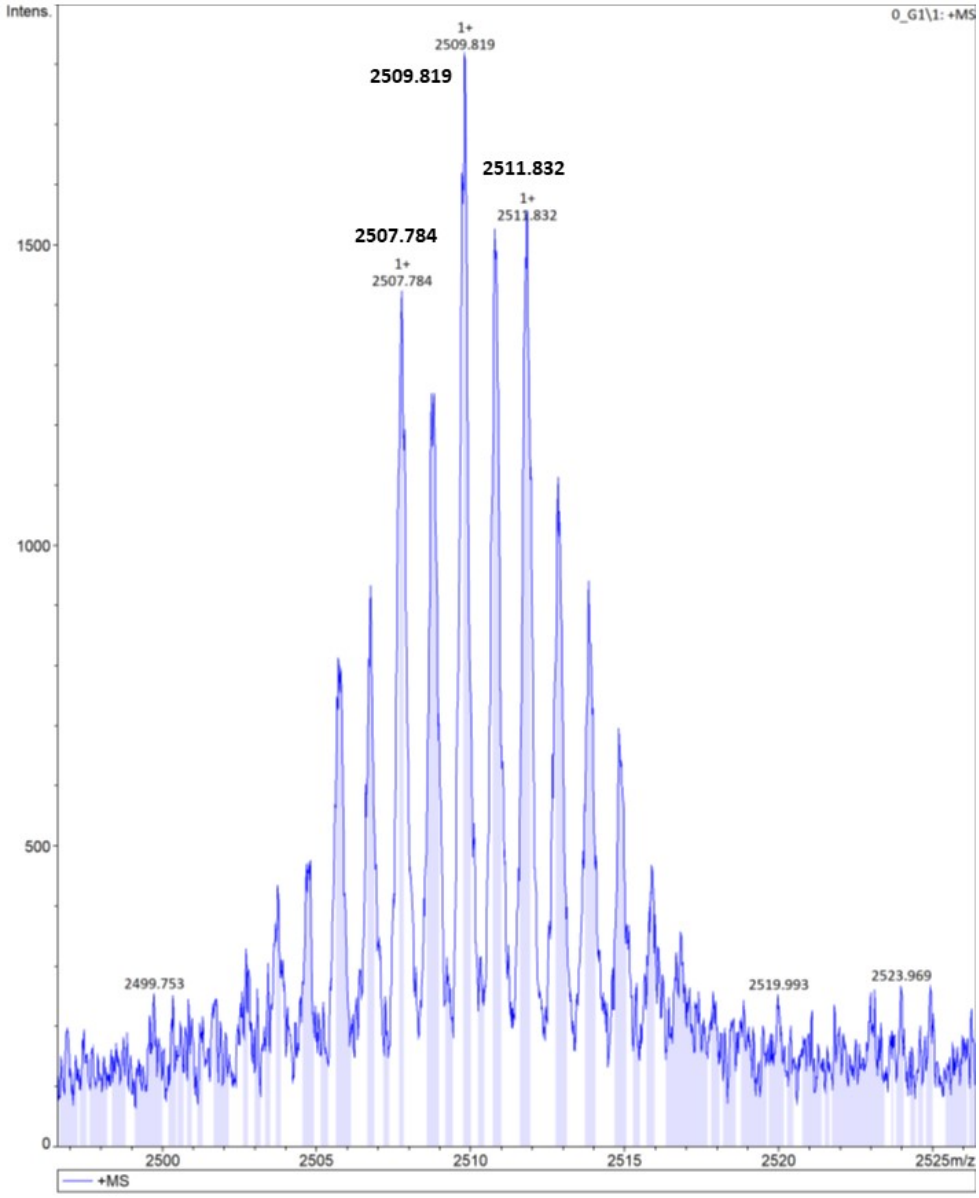

Figure S184. Zoomed in MALDI spectra of **5c**, showing the expected isotope pattern.

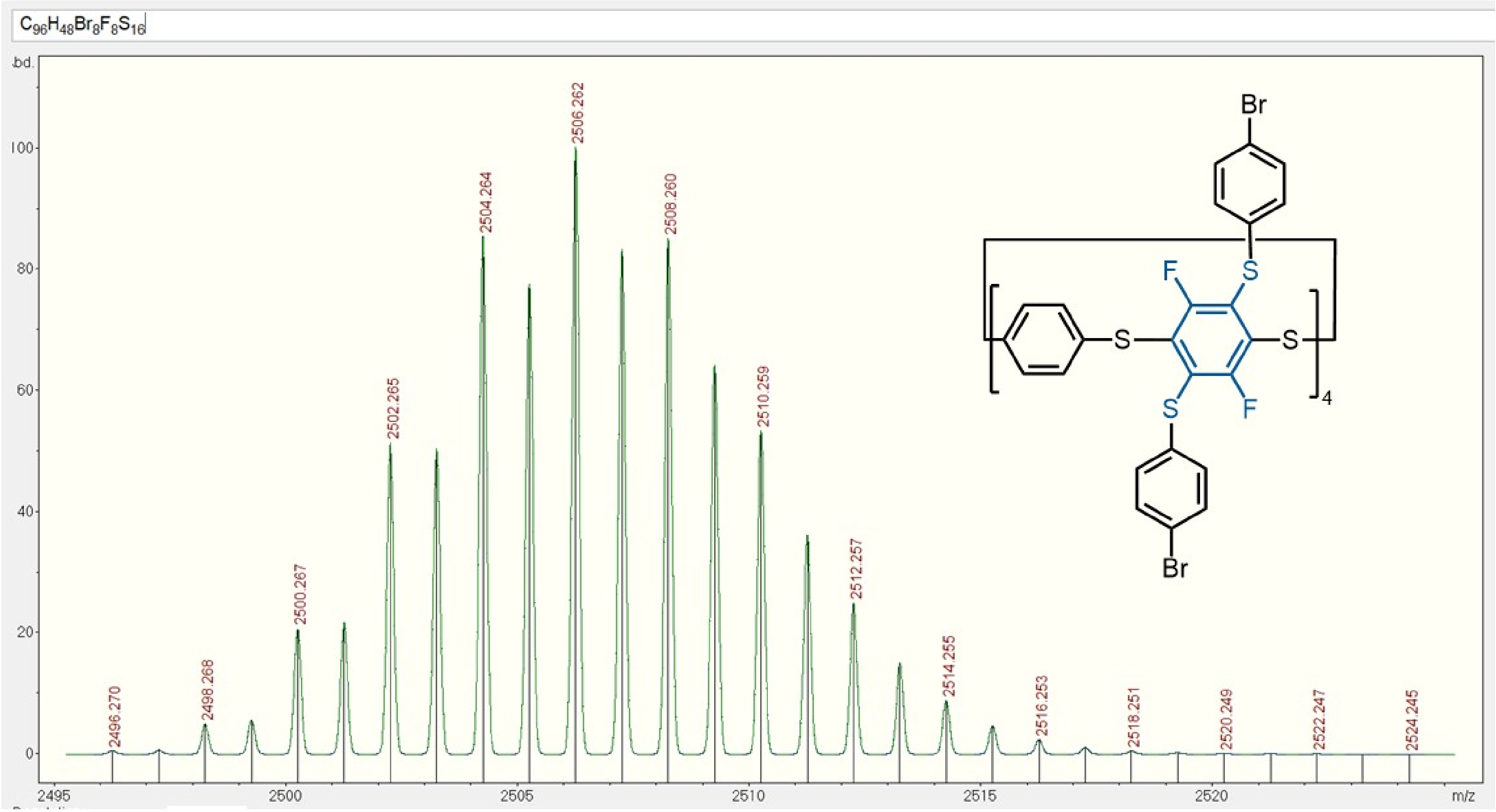

Figure S185. Simulated MALDI spectra of **5c**.

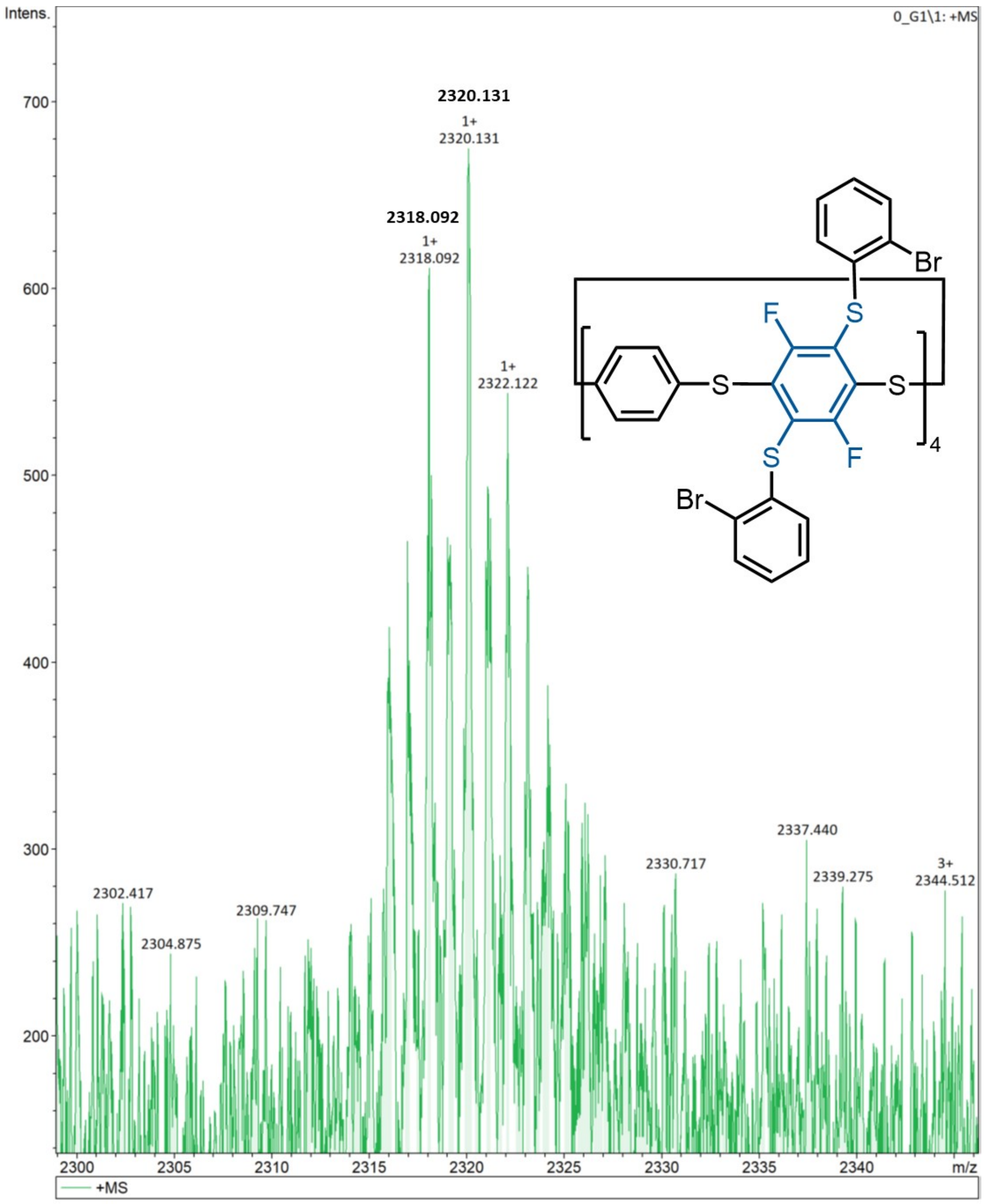

Figure S186. Zoomed in MALDI spectra of **5d**, showing the expected isotope pattern.

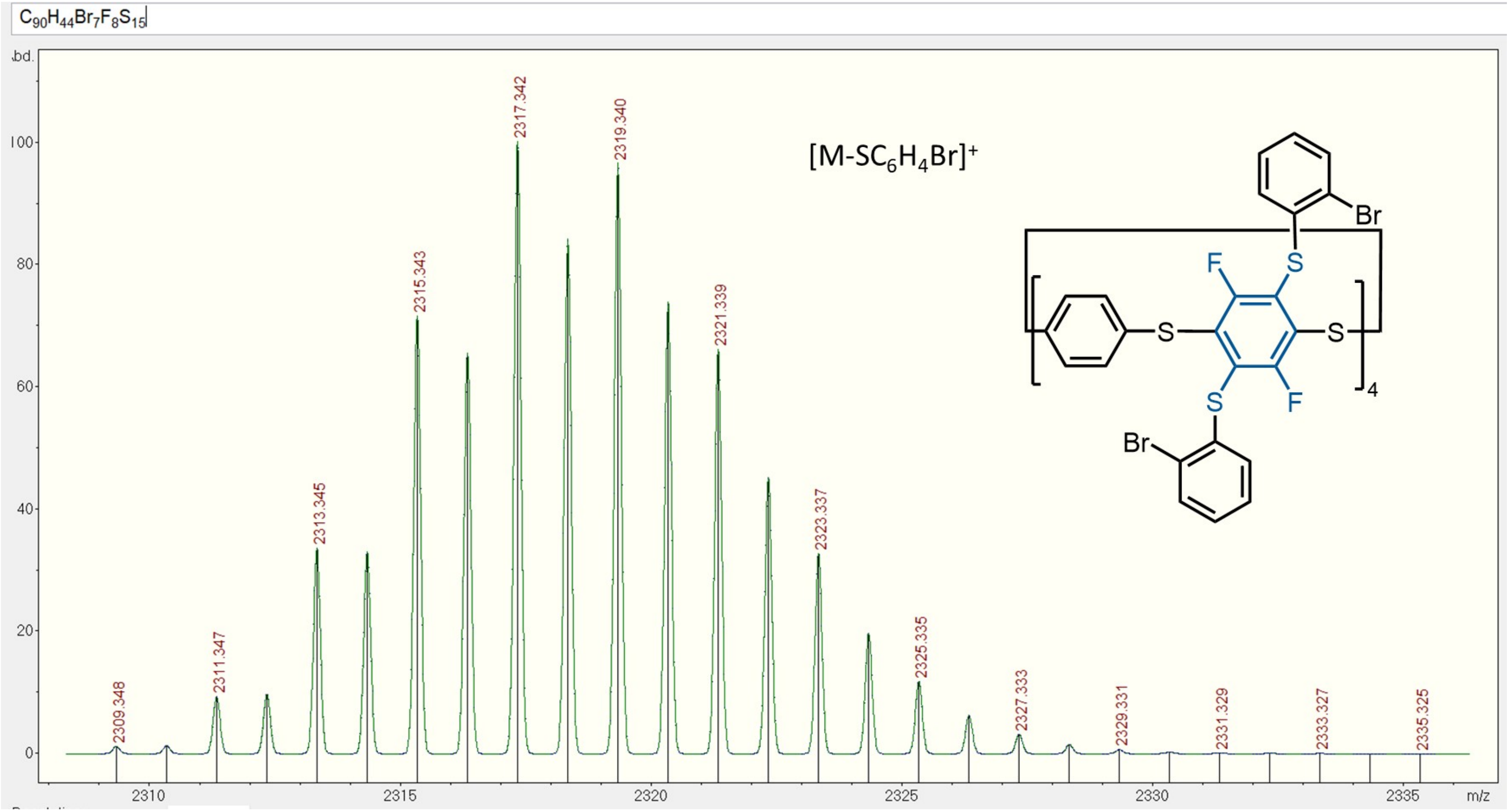

Figure S187. Simulated MALDI spectra of **5d**.

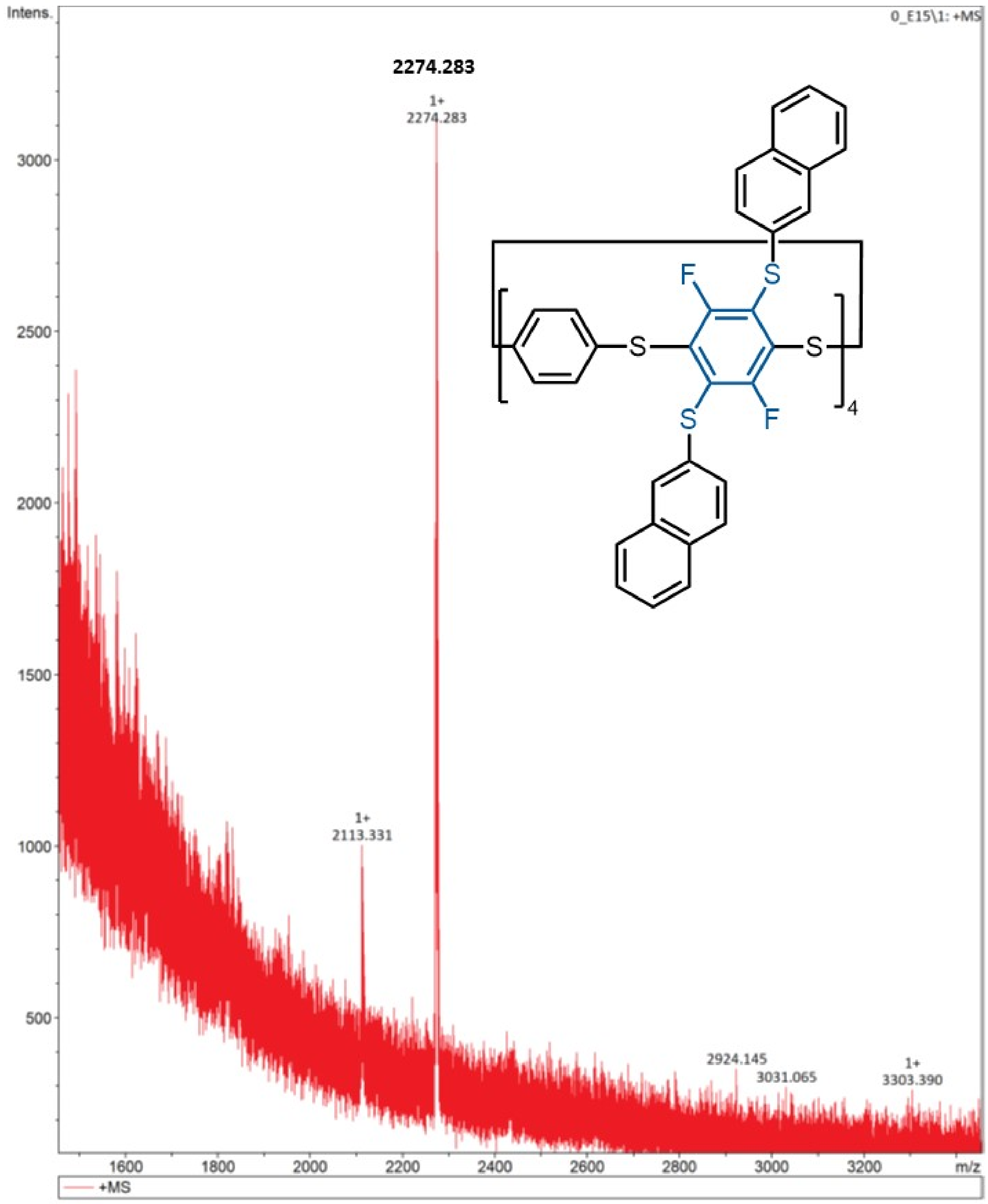

Figure S188. MALDI spectra of **5e**.

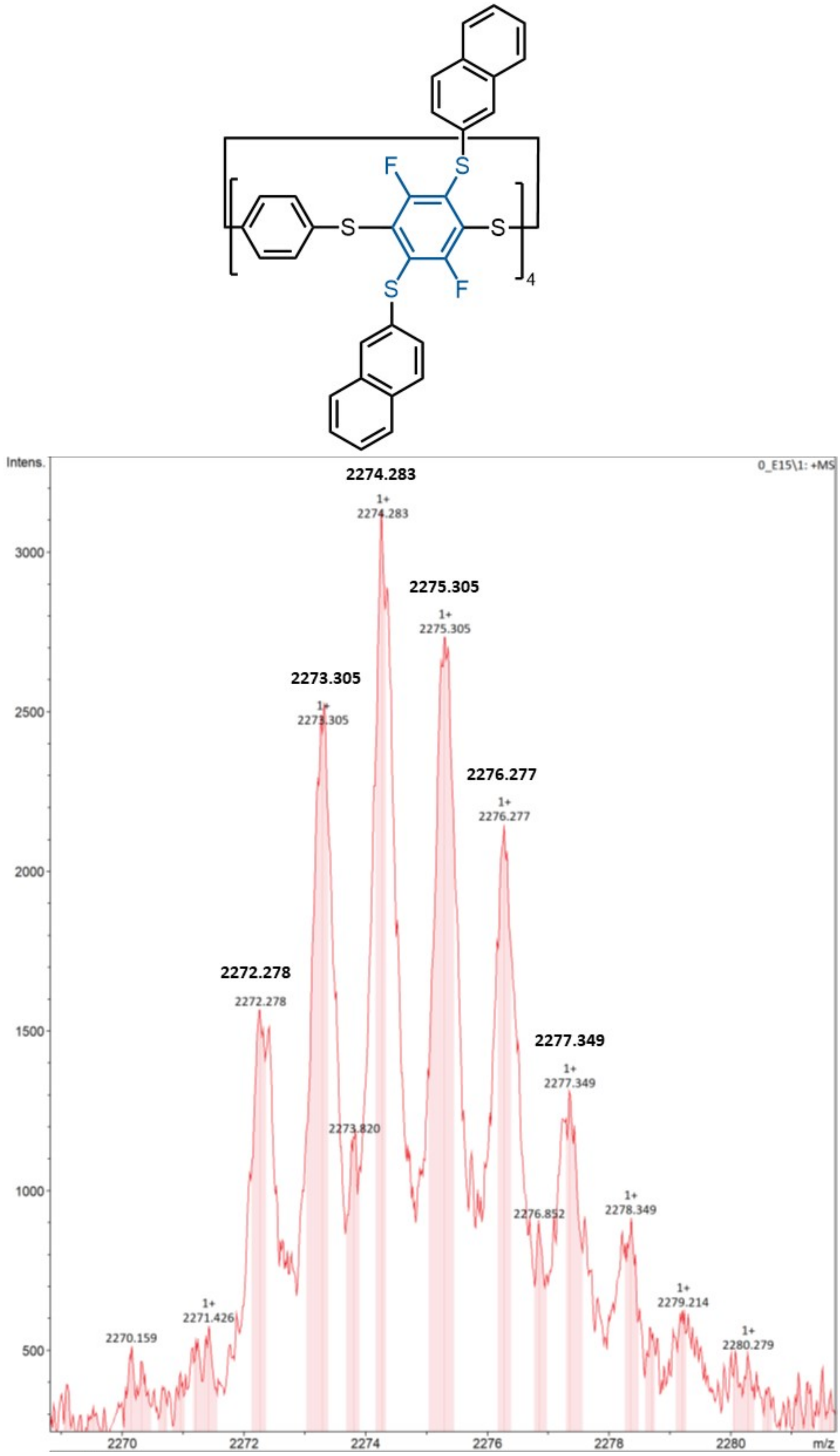

Figure S189. Zoomed in MALDI spectra of **5e**, showing the expected isotope pattern.

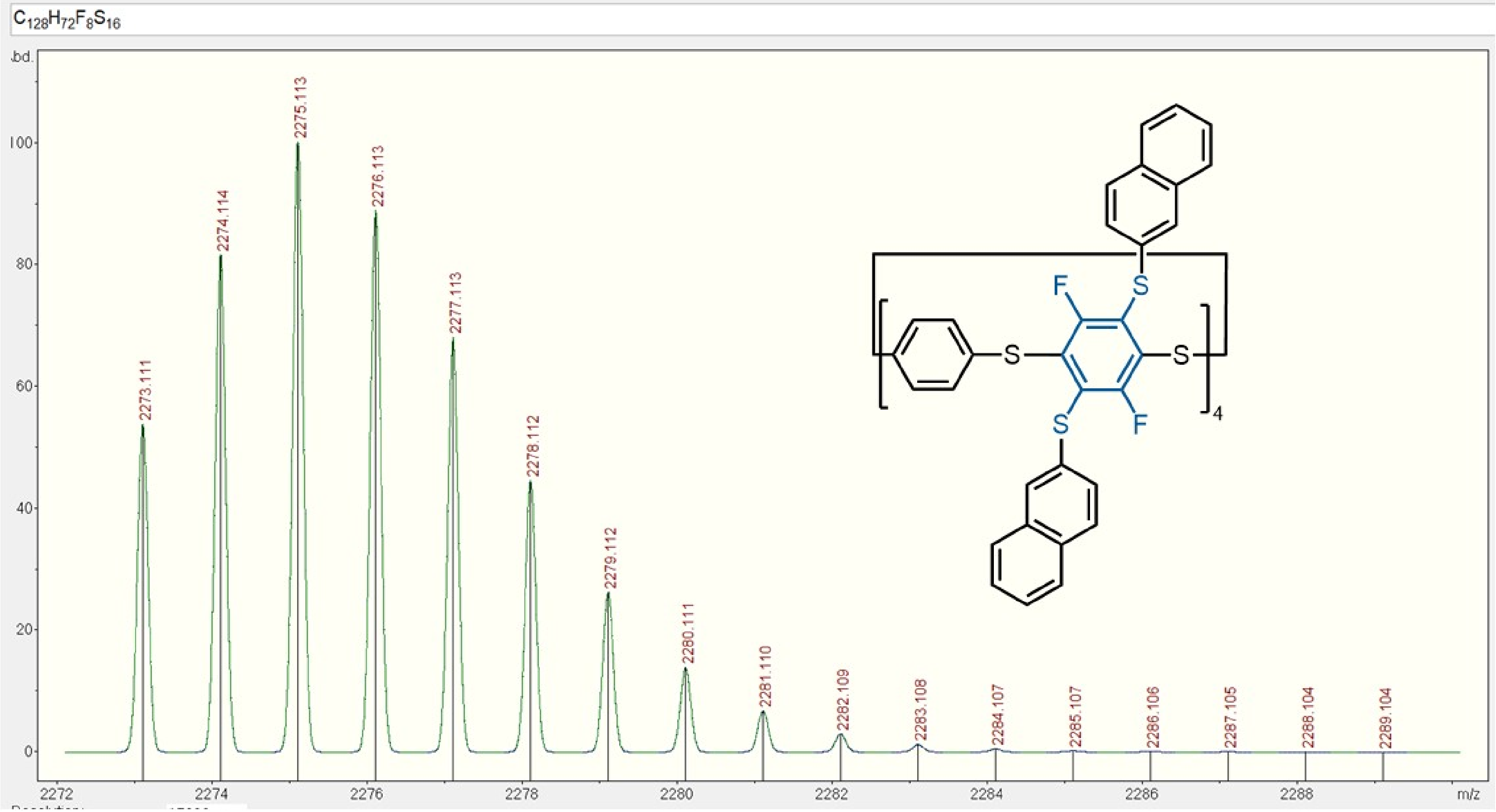

Figure S190. Simulated MALDI spectra of **5e**.

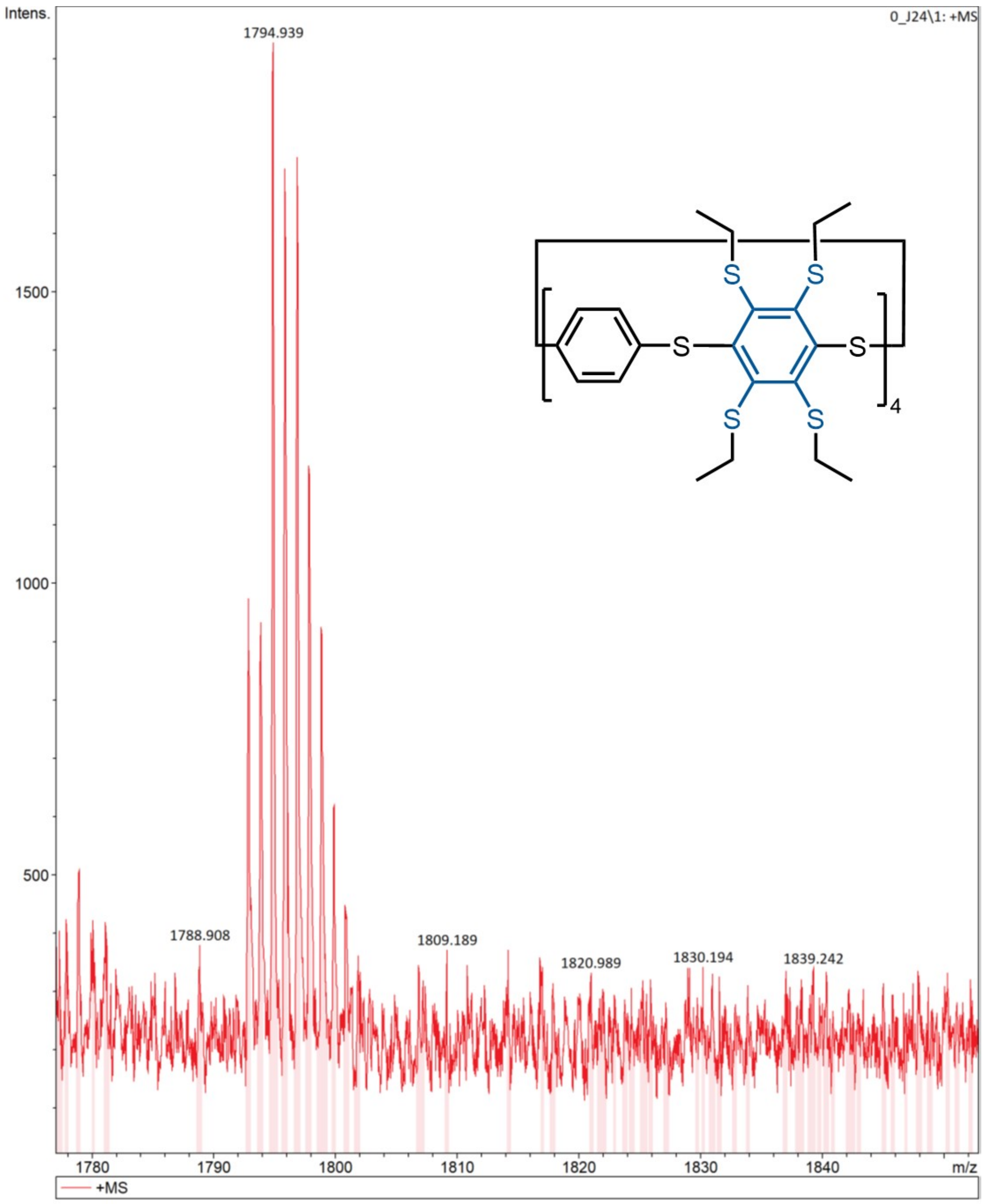

Figure S191. MALDI spectra of **6a**.

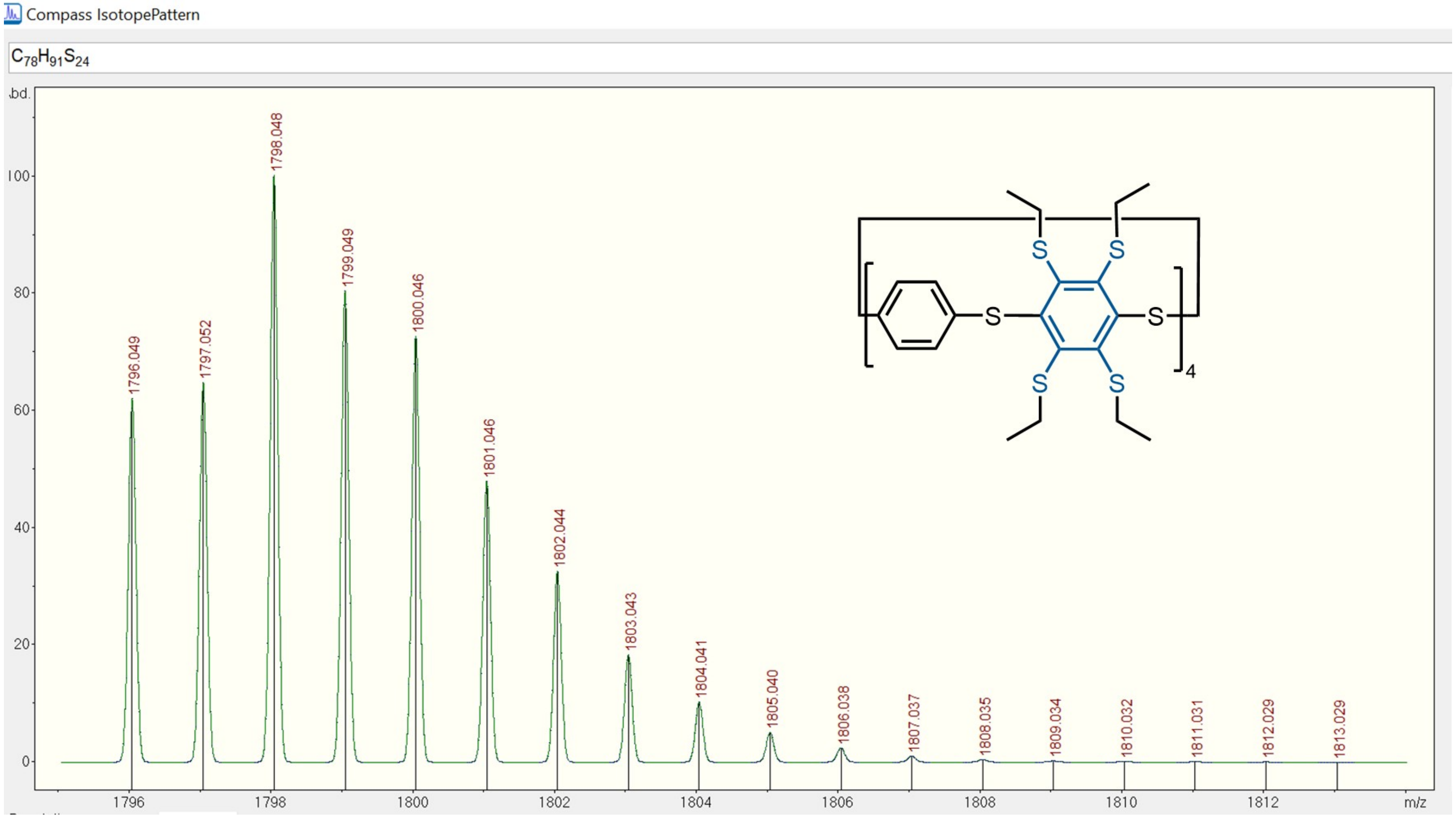

Figure S192. Simulated MALDI spectra of **6a**.

5. X-ray Crystallographic Analysis

Analysis of all crystal structures, their packing and their Hirshfeld surface plots are shown (Figures S193-211) with the crystal system, space group and unit cell parameters reported below.

Collection data are summarised in Table S1. Selected hydrogen-bond parameters for each crystal structure are also outlined (Table S2). Full data are in the cif files CCDC-2175101-2175106.

Single Crystal X-ray Diffraction (SCXRD) Refinement Special Details

1a–Hexane

The crystals of the macrocycle diffracted weakly with resolution limit of 1.0 Å despite the use of synchrotron radiation. The data used in the refinement was truncated to a resolution of 1.0 Å resulting in a low data to parameter ratio. Restraints were applied to the geometries and anisotropic displacement parameters of all atoms in the structure to aid the refinement.

All C-S bonds C-F bonds in the structure were respectively restrained to have similar distances (SADI). The geometries of all phenyls and tetrafluorophenly rings were respectively restrained to have similar geometries reflecting their symmetry (SAME). All tetrafluorophenly moieties were restrained to have planar geometries (FLAT). Rigid bond and similarity restraints were applied to the anisotropic displacement parameters of all atoms in the structure (RIGU, SIMU).

Electron density peaks indicative of hexane residues were observed in 1D channels running through the stacked macrocycles in the direction of the *c*-axis. The electron density peaks could not be developed into a sensible model for discreet ordered hexane residues and hence were accounted for with a solvent mask.

A solvent mask was calculated, and 600 electrons were found in a volume of 1140 Å^3 in 1 void per unit cell. This is consistent with the presence of 3[C_6H_{14}] per Asymmetric Unit which accounts for 600 electrons per unit cell.

1a–$CHCl_3$

Crystals of the macrocycle diffracted weakly with a resolution limit of 1.35 Å, despite the use of an intense rotating anode copper source. The data used in the refinement was truncated to a resolution of 1.35 Å consequently resulting in a very low data-parameter ratio of 2.65. To aid refinement of the

structure with such a low data to parameter ratio restraints were applied to the geometries and anisotropic displacement parameters of all atoms in the structure.

Additional weak diffraction peaks were observed in between the main peaks along both the *a* and *b* axes. The peaks were too weak to extract any information or use for indexing a large unit cell of a superstructure. The origin of the potential superstructure is likely to be either disorder in the chloroform solvent residues or the disorder of the phenyl and tetrafluorophenyl rings.

Occupational disorder is observed for the phenyl and tetrafluorophenyl rings of the macrocycle: each ring is modelled with as having half occupancy of each such moiety. The chloroform residue is modelled with its occupancy fixed at a value of 0.5.

Geometric similarity restraints were applied the geometries of all chemically similar 1,2 and 1,3 distances in the structure (SADI, SAME). The phenyl and tetrafluorophenyl moieties were restrained to have planar geometries (FLAT). The three C-Cl bond lengths of the chloroform residue were restrained to have target distances taken from the Olex2 FragmentDB (DFIX).

Rigid bond and similarity restraints were applied to the anisotropic displacement parameters of all atoms in the structure (RIGU, SIMU).

1a–THF

The crystal was integrated as a two-component twin and the final structure refined against an HKLF5 reflection file resulting in a batch scale factor of 0.408(1).

A large residual electron density peak of 2.69 Å^{-3} is observed 0.23 Å from sulphur atom S2B. This peak is likely caused by a combination of deficiencies in the handling of the twinning and a minor unmodelled disorder component in the ring conformation.

A solvent mask was calculated, and 196 electrons were found in a volume of Å^3 in 1 void per unit cell. This is consistent with the presence of 2[C_4H_8O] per Asymmetric Unit which account for 160 electrons per unit cell.

1a–DMF

A solvent mask was calculated, and 240 electrons were found in a volume of 1102 Å^3 in 2 voids per unit cell. This is consistent with the presence of 2[C_3H_7NO] per Asymmetric Unit which account for 320 electrons per unit cell.

Table S1. Summarised X-ray collection data for **1a** solvates and **1b**.

	**1a-Hexane**	**1a-CHCl3**	**1a-THF**	**1a-Pyridine**	**1a-DMF**	**1b**
Chemical formula	$C_{48}H_{16}F_{16}S_8 \cdot 3[C6H_{14}]$	$C_{48}H_{16}F_{16}S_8 \cdot CHCl_3$	$2(C_{24}H_8F_8S_4)$	$C_{48}H_{16}F_{16}S_8 \cdot 4(C_5H_5N)$	$C_{48}H_{16}F_{16}S_8$	$C_{56}H_{32}F_{16}S_8$
M_r	1411.60	1272.45	1297.29	1469.49	1299.28	1265.29
Crystal system, space group	Orthorhombic, $Pna2_1$	Triclinic, *P*-1	Triclinic, *P*-1	Monoclinic, $P2_1/c$	Tetragonal, $P4_2/n$	Triclinic, *P*-1
Temperature (K)	100	120	120	100	100	100
a, *b*, *c* (Å)	34.3683 (7), 28.4432 (5), 5.4447 (1)	6.7916 (16), 13.818 (4), 15.064 (4)	13.6071 (3), 14.6029 (3), 15.3344 (3)	5.89701 (12), 32.2682 (3), 16.4767 (3)	19.8473 (2), 19.8473 (2), 13.1934 (1)	9.5286 (2), 10.3554 (2), 14.2500 (4)
α, β, γ (°)	90, 90, 90	78.57 (2), 81.39 (2), 82.79 (2)	97.914 (2), 98.988 (2), 110.147 (2)	90, 95.0947 (16), 90	90, 90, 90	75.540 (2), 89.177 (2) 73.340 (2)
V (Å^3)	5322.44 (17)	1363.3 (7)	2764.52 (11)	3122.89 (7)	5197.08 (11)	1301.90 (5)
Z, *(Z')*	4, (1)	1, (0.5)	2, (1)	2, (0.5)	4, (0.5)	1, (0.5)
Radiation type	Synchrotron, λ = 0.6889 Å	Cu *K*α	Cu *K*α	Synchrotron, λ = 0.6889 Å	Synchrotron, λ = 0.6889 Å	Synchrotron, λ = 0.6889 Å
μ (mm^{-1})	0.41	5.20	3.86	0.35	0.41	0.41
Crystal size (mm)	0.2 × 0.02 × 0.01	0.36 × 0.03 × 0.03	0.30 × 0.06 × 0.03	0.06 × 0.03 × 0.02	0.1 × 0.02 × 0.02	0.04 × 0.02 × 0.01
Diffractometer	DLS Beamline I19-EH1, Fluid Film Devices, Dectris PILATUS 2M	Rigaku XtalLAB PRO MM007, Dectris PILATUS3 R 200K	Rigaku XtalLAB PRO MM007, PILATUS3 R 200K	DLS Beamline I19-EH1, Fluid Film Devices, Dectris PILATUS 2M	DLS Beamline I19-EH1, Fluid Film Devices, Dectris PILATUS 2M	DLS Beamline I19-EH1, Fluid Film Devices, Dectris PILATUS 2M
T_{min}, T_{max}	–	0.574, 1.000	0.513, 1.000	0.980, 1.0	0.985, 1.0	0.992, 1.0
No. of measured, independent and observed [$I > 2\sigma(I)$] reflections	42986, 5593, 4168	5179, 1148, 812	11514, 11514, 9518	62385, 14951, 9446	73694, 6454, 5194	26997, 11650, 7278
R_{int}	0.073	0.134	Twin	0.051	0.114	0.046
θ_{max} (°)	20.2	34.8	74.5	36.0	27.3	36.0
(sin θ/λ)$_{max}$ (Å^{-1})	0.500	0.370	0.625	0.853	0.667	0.853
$R[F^2 > 2\sigma(F^2)]$, $wR(F^2)$, S	0.061, 0.193, 1.03	0.176, 0.477, 2.29	0.125, 0.366, 1.63	0.046, 0.139, 1.08	0.053, 0.149, 1.05	0.049, 0.137, 1.02
No. of reflections, parameters, and restraints	5593, 649,1893	1148, 433, 1164	11514, 650, 534	14951, 433, 0	6454, 325, 0	11650, 365, 0
$\Delta\rangle_{max}$, $\Delta\rangle_{min}$ (e Å^{-3})	0.23, -0.24	0.73, -0.43	2.69, -0.74	0.71, -0.64	0.91, -0.31	0.69, -0.68
Absolute structure parameter (Flack)	-0.01 (6)	–	–	–	–	–
CCDC	2175101	2175102	2175103	2175104	2175105	2175106

5.1. 1a–Hexane

Method of Growth: liquid-liquid diffusion: hexanes into CH_2Cl_2

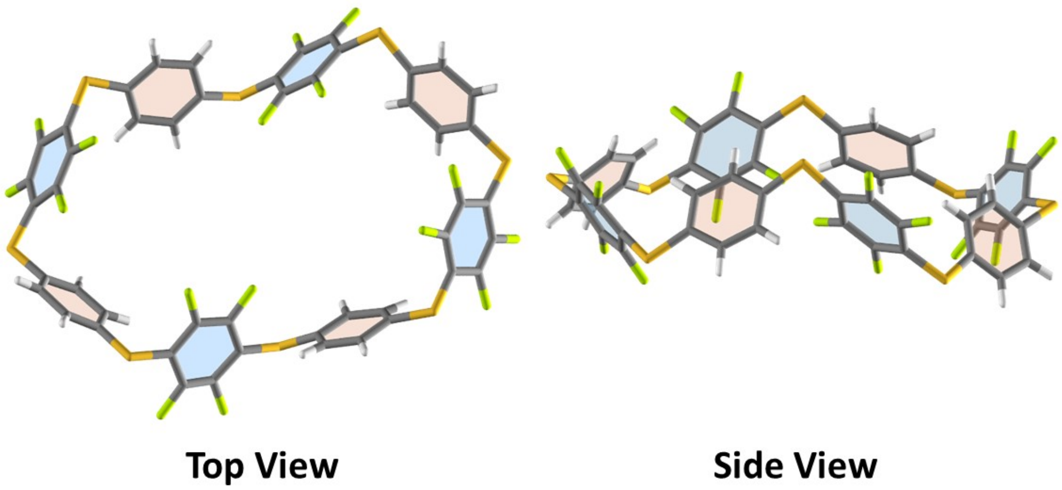

Figure S193. Solid-state structure of **1a**–**Hexane** viewed from above and side-on, with guests excluded for clarity.

Crystal System: Orthorhombic

Space group: *Pna2₁*

Unit Cell Parameters: *a* = 34.537(3) Å, *b* = 28.132(3) Å, *c* = 5.5150(7) Å, β = 90°, V = 5358.35 Å3, Z = 4, Z’ = 0

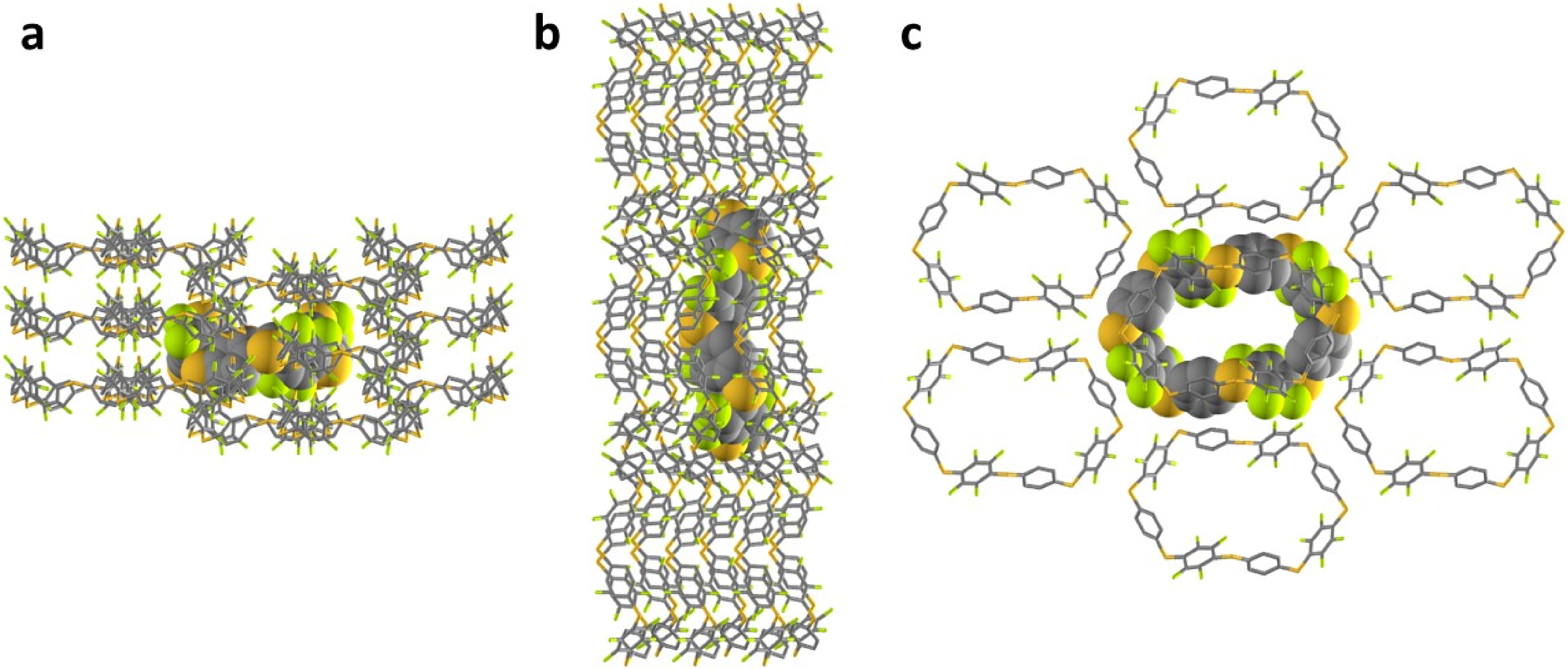

Figure S194. Solid-state superstructure **1a**–**Hexane**. A central molecule (space filling representation) is shown embedded in a section of the lattice in order to illustrate the crystal packing. Projections are viewed along the crystallographic (a) *a*-, (b) *b*-, and (c) *c*-axes.

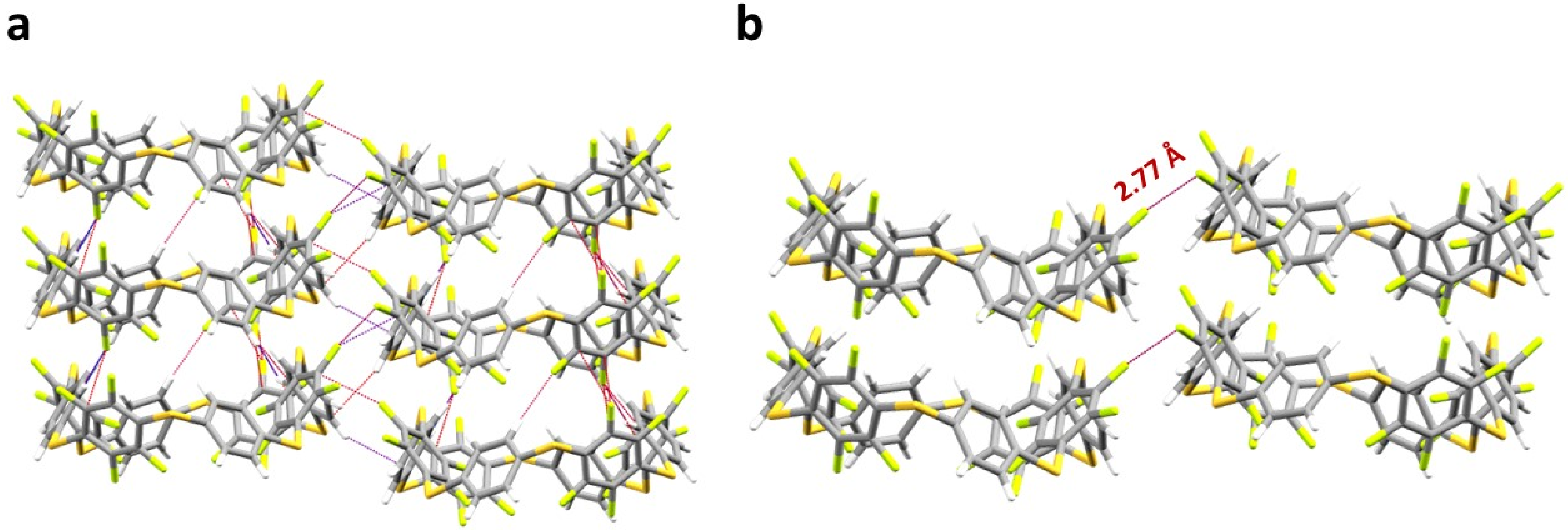

Figure S195. Solid-state superstructure **1a**–**Hexane**: (a) Intermolecular close contacts within the sum of the Van der Waals radius. (Short (Green), Medium (Blue), Long (Red). (b) Highlighting type-1 F-F interaction between adjacent units with all other close contacts omitted for clarity.

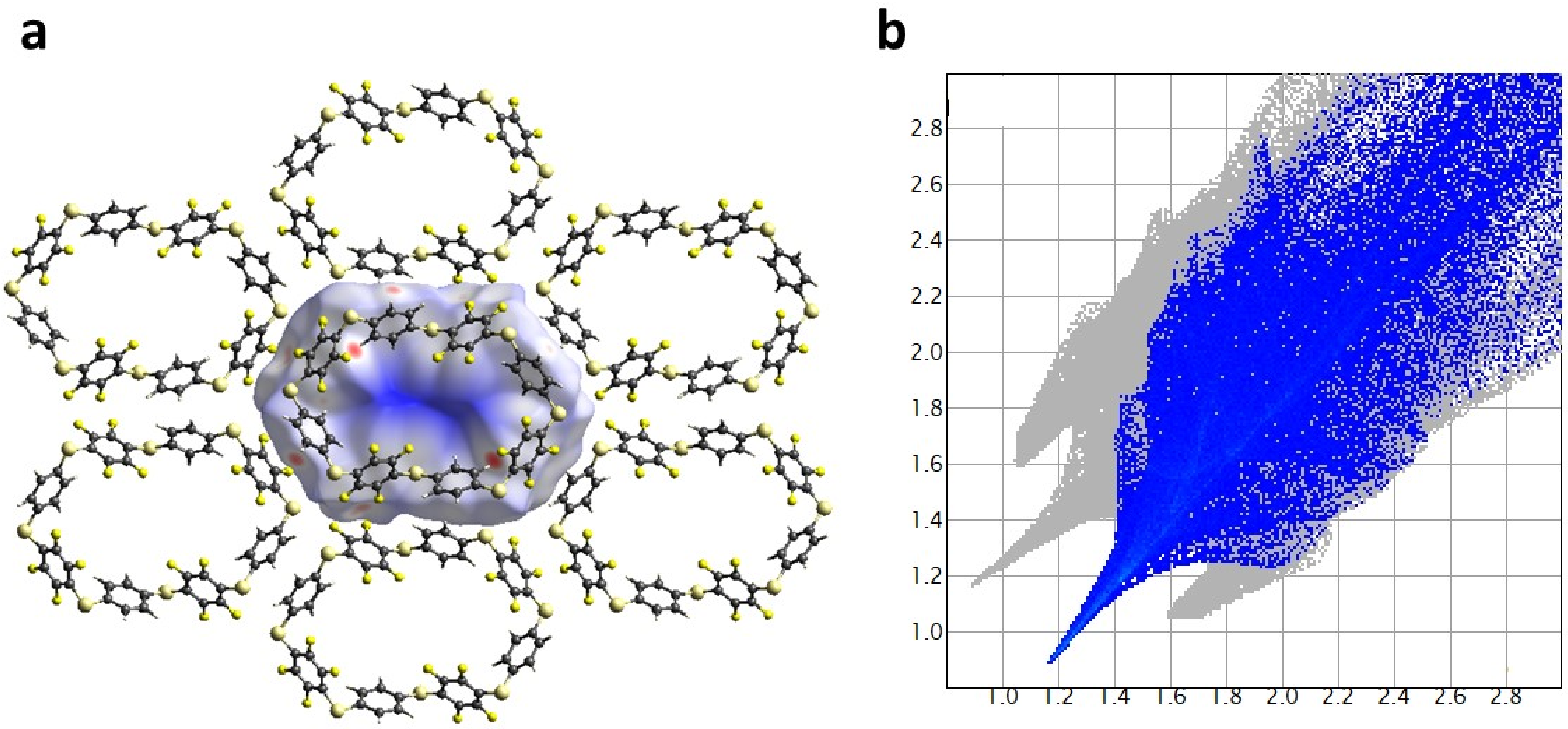

Figure S196. Solid-state superstructure open **1a–Hexane** polymorph: (a) Hirshfield surface plots of **1a–Hexane** with generated close contacts. (b) Fingerprint plot of **1a–Hexane**.

5.2. 1a–$CHCl_3$

Method of Growth: Slow evaporation of a solution of **1a** in $CHCl_3$

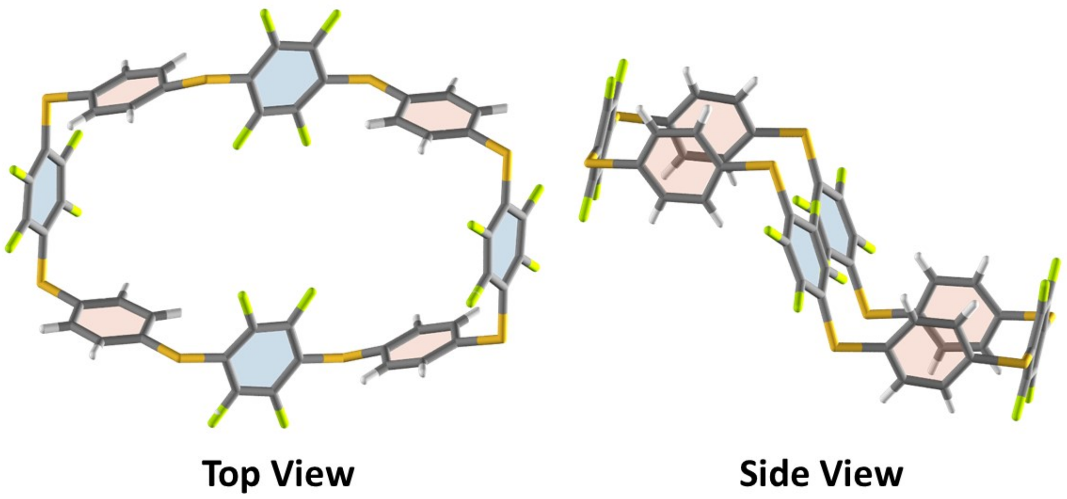

Figure S197. Solid-state structure of **1a**–**$CHCl_3$** viewed from above and side-on, with guests excluded for clarity.

Crystal System: Triclinic

Space group: *P1*

Unit Cell Parameters: *a* = 6.7916(16) Å, *b* = 13.818(4) Å, *c* = 15.064(4) Å, β = 81.39(2)°, V = 1363.33 Å3, Z = 1, Z′ = 0

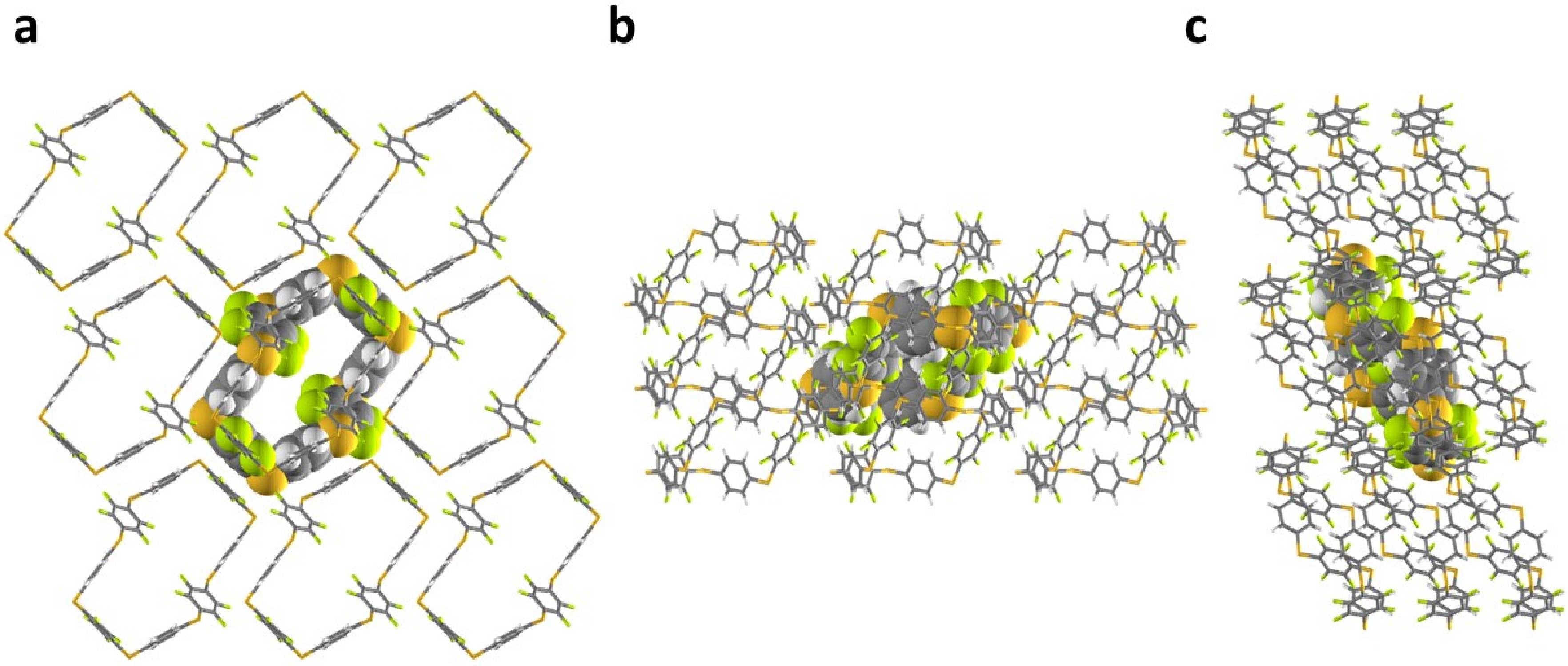

Figure S198. Solid-state superstructure **1a–$CHCl_3$**. A central molecule (space filling representation) is shown embedded in a section of the lattice in order to illustrate the crystal packing. Projections are viewed along the crystallographic (a) *a*-, (b) *b*-, and (c) *c*-axes.

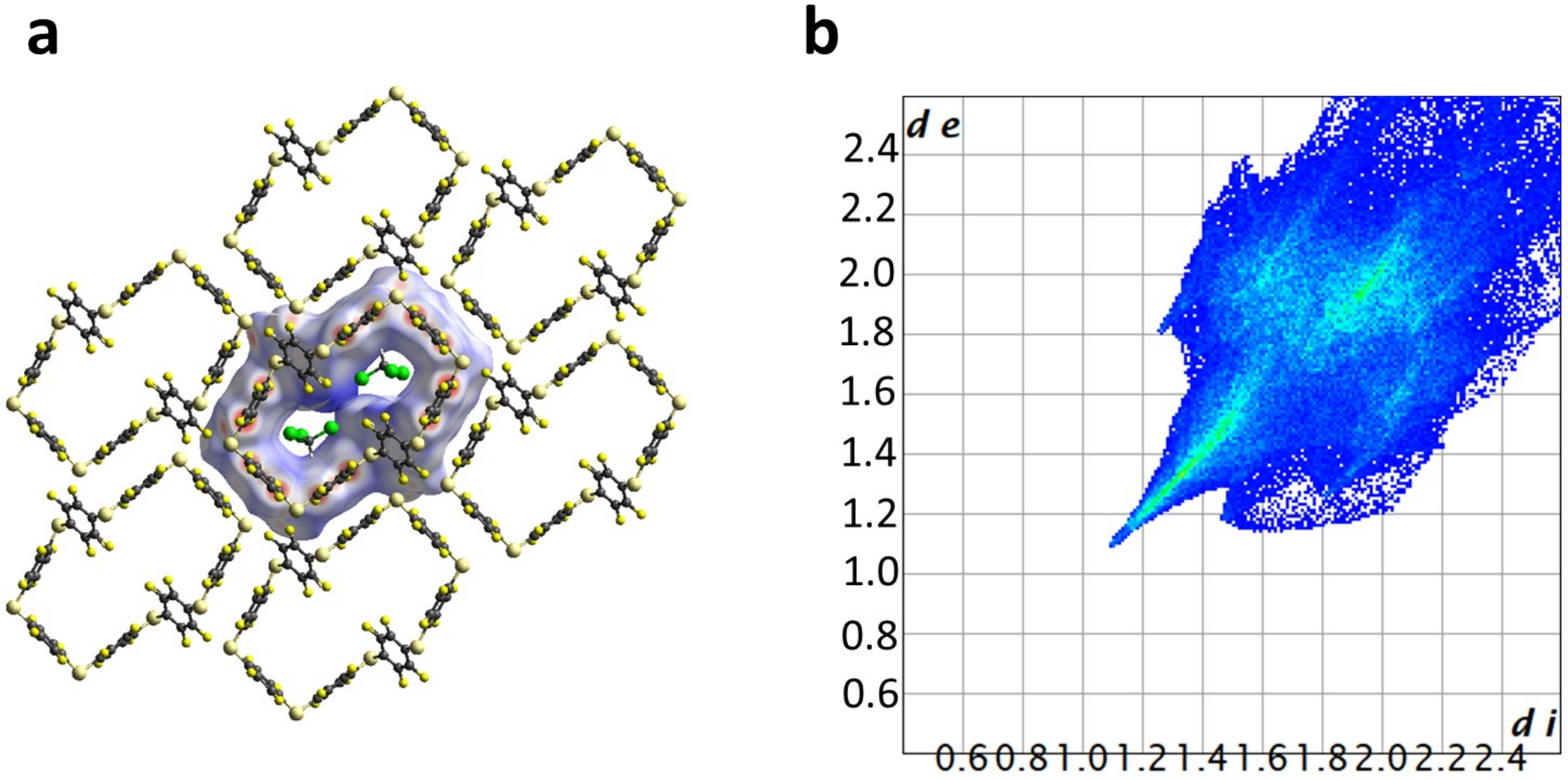

Figure S199. Solid-state superstructure of **1a–CHCl$_3$**: (a) Hirshfield surface plots of **1a–CHCl$_3$** with generated close contacts within the van der Waals Radii and guest excluded. **(**b) Fingerprint plot of **1a–CHCl$_3$** staircase polymorph.

5.3. 1a–THF

Method of Growth: Slow evaporation of a solution of **1a** in THF

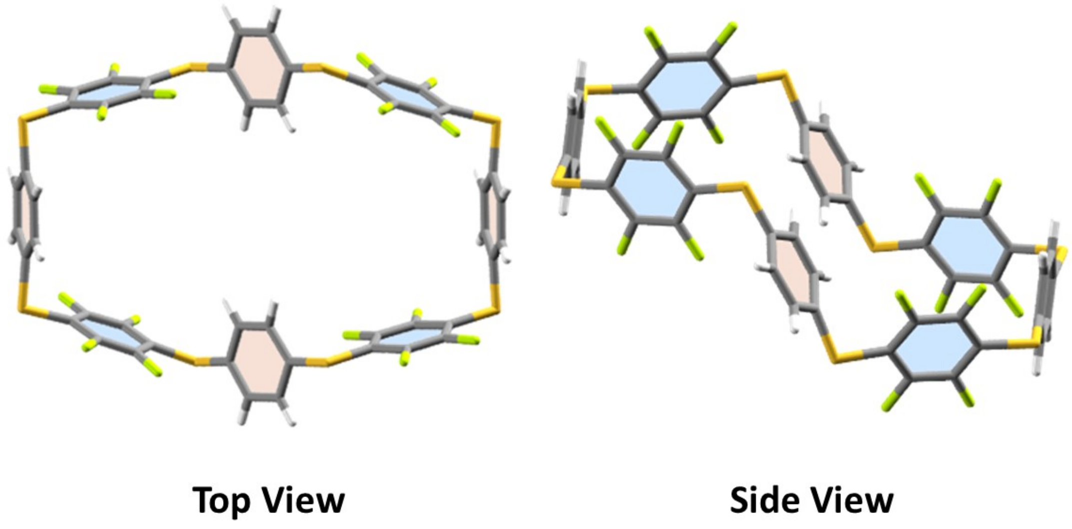

Figure S200. Solid-state structure of **1a**–**THF** viewed from above and side-on, with guests excluded for clarity.

Crystal System: Triclinic

Space group: *P1*

Unit Cell Parameters: *a* = 13.6071(3) Å, *b* = 14.6029(3) Å, *c* = 15.3344(3) Å, β = 98.988(2)°, V = 2764.52 Å3, Z = 2, Z' = 1

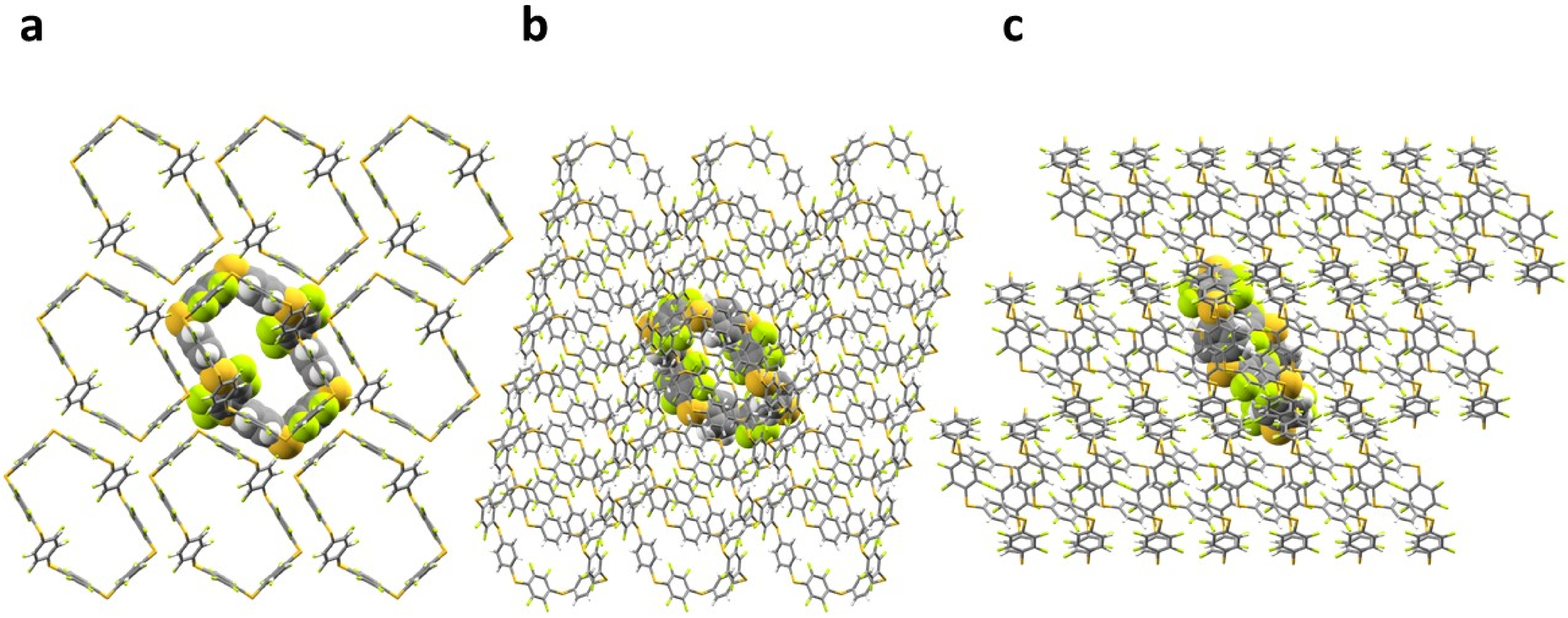

Figure S201. Solid-state superstructure of **1a**–**THF**. A central molecule (space filling representation) is shown embedded in a section of the lattice in order to illustrate the crystal packing. Projections are viewed along the crystallographic (a) *a*-, (b) *b*-, and (c) *c*-axes.

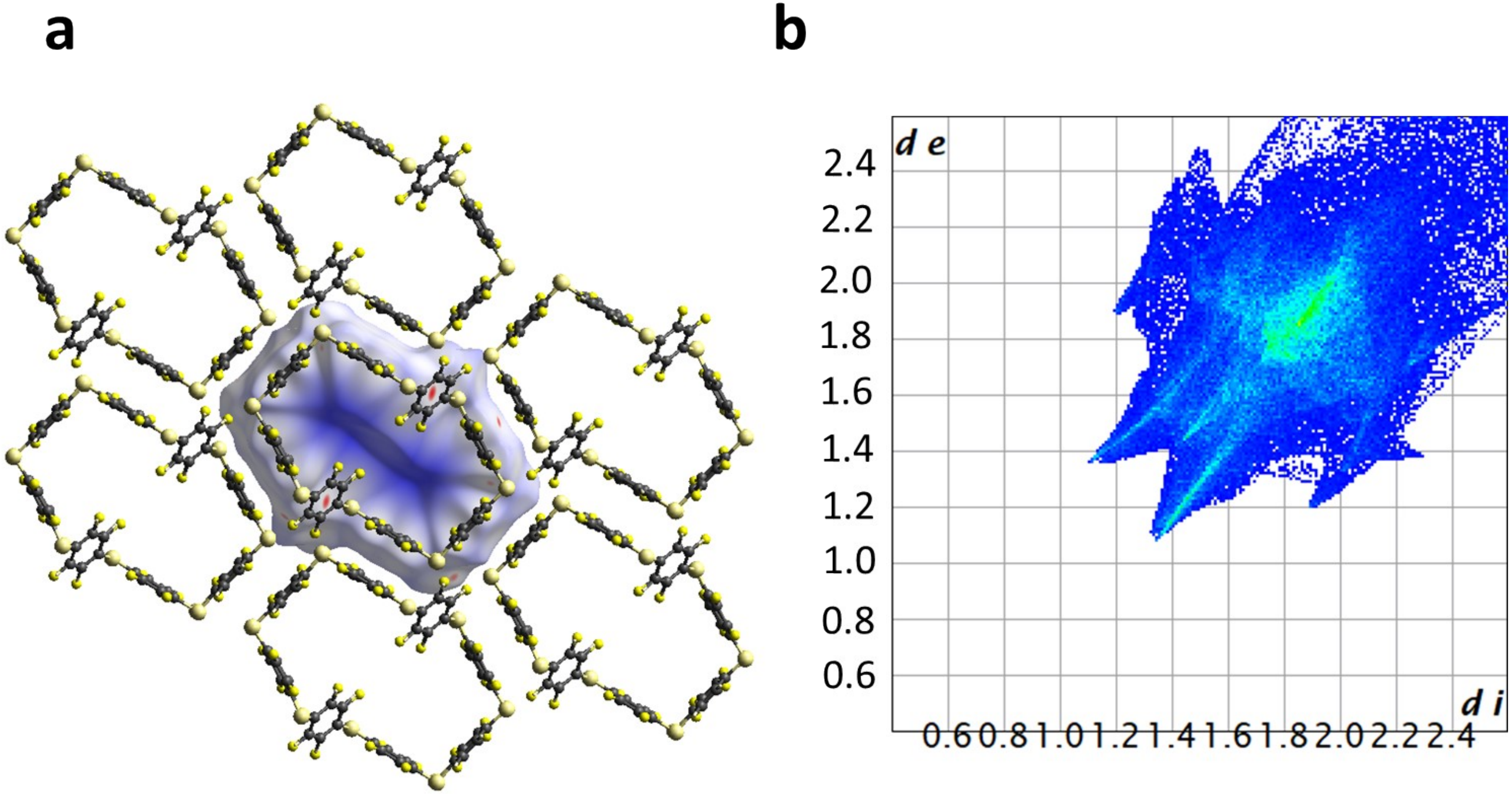

Figure S202. Solid-state superstructure of **1a**–**THF** staircase polymorph: (a) Hirshfield surface plots of **1a**–**THF** with generated close contacts within van der Waals Radii and guest excluded. (b) Fingerprint plot of **1a**–**THF**.

5.4. 1a–Pyridine

Method of Growth: Slow evaporation of a solution of **1a** in pyridine

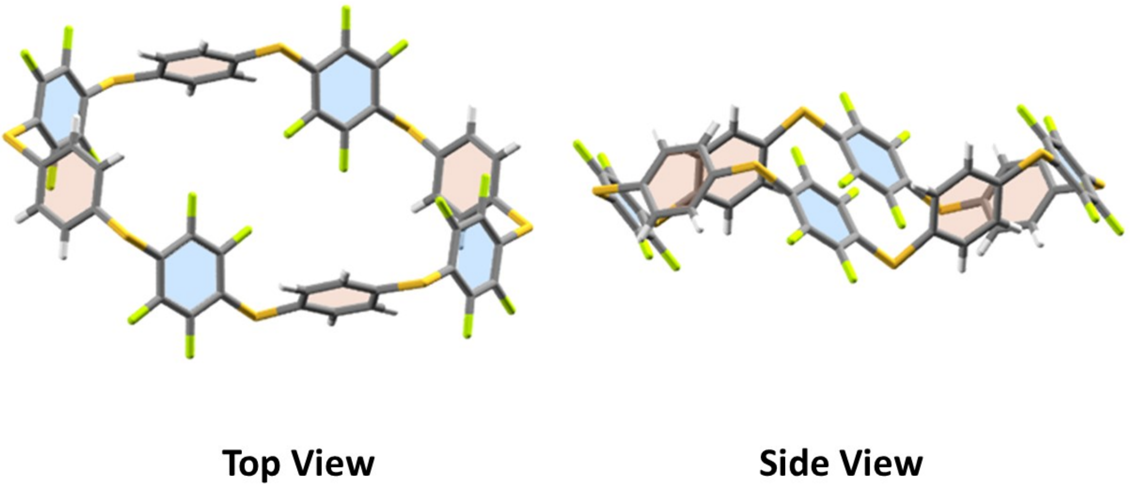

Figure S203. Solid-state structure of **1a**–**Pyridine** viewed from above and side-on, with guests excluded for clarity.

Crystal System: Monoclinic

Space group: $P2_1/c$

Unit Cell Parameters: *a* = 5.89701(12) Å, *b* = 32.2682(3) Å, *c* = 16.4767(3) Å, β = 95.0947(16)°, V = 3122.9 Å3, Z = 2, Z’ = 0.5

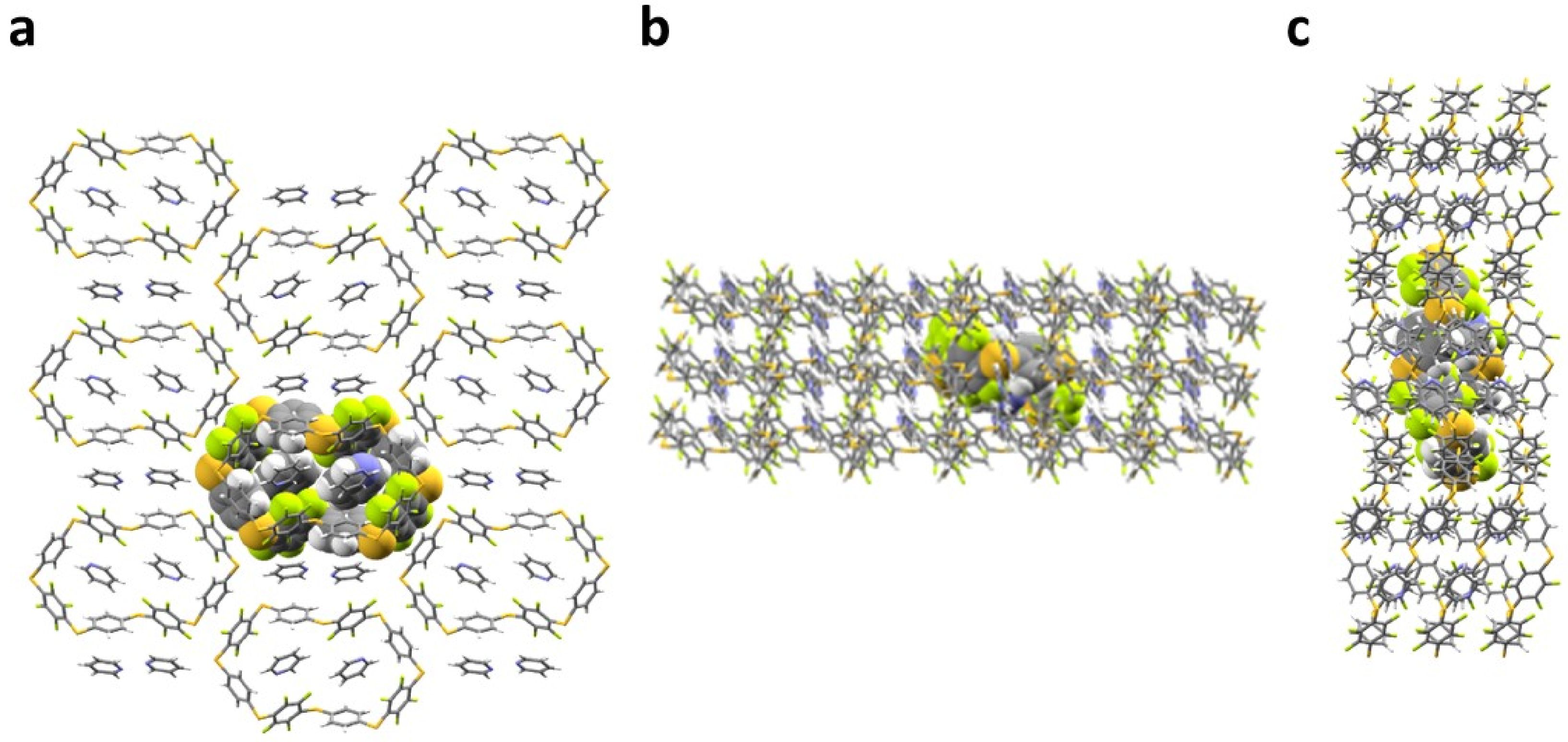

Figure 204. Solid-state superstructure of **1a**–**Pyridine** with guest included. A central molecule (space filling representation) is shown embedded in a section of the lattice in order to illustrate the crystal packing. Projections are viewed along the crystallographic (a) *a*-, (b) *b*-, and (c) *c*-axes.

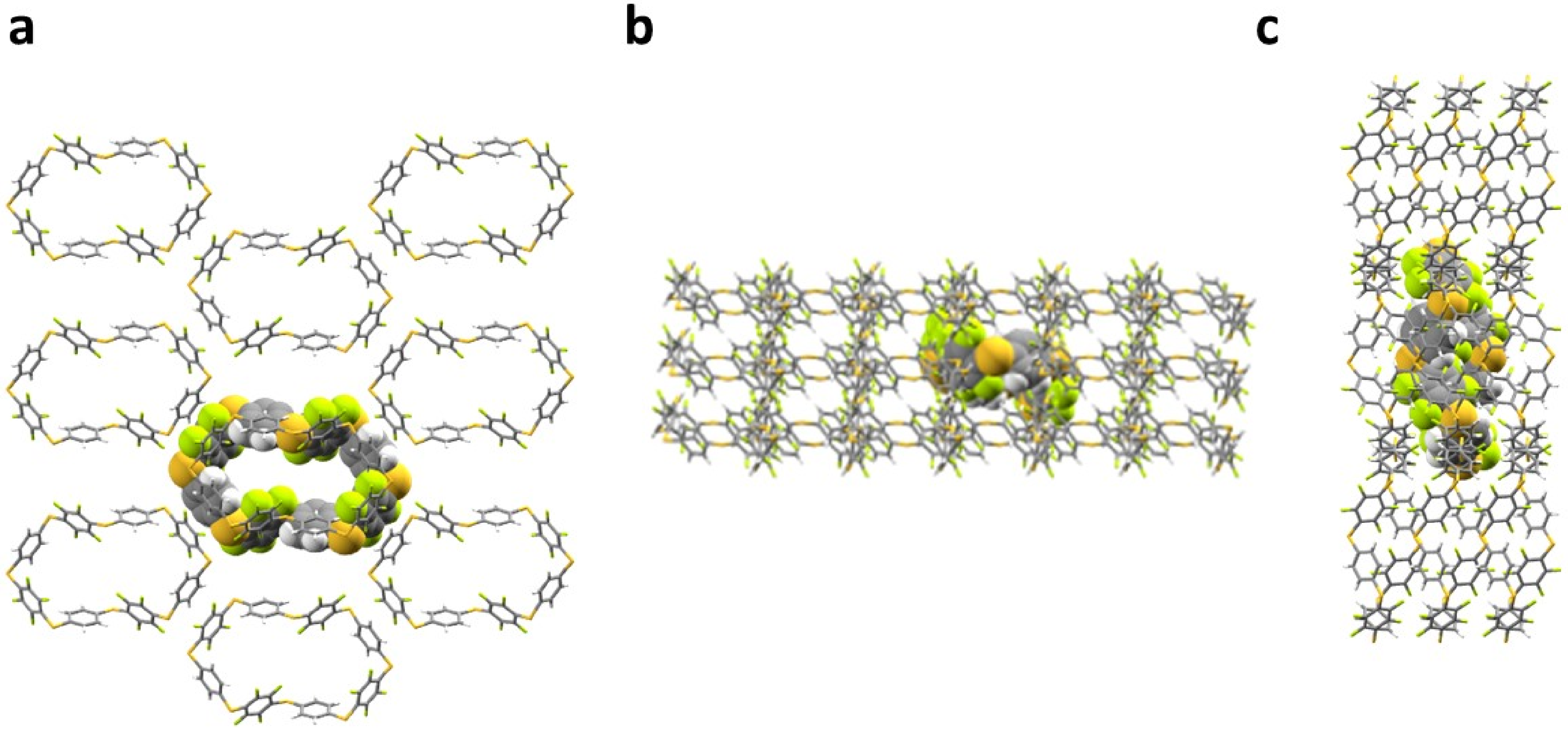

Figure S205. Solid-state superstructure of **1a**–**Pyridine** with guest excluded. A central molecule (space filling representation) is shown embedded in a section of the lattice in order to illustrate the crystal packing. Projections are viewed along the crystallographic (a) *a*-, (b) *b*-, and (c) *c*-axes.

5.5. 1a–DMF

Method of Growth: Slow evaporation of a solution of **1a** in DMF

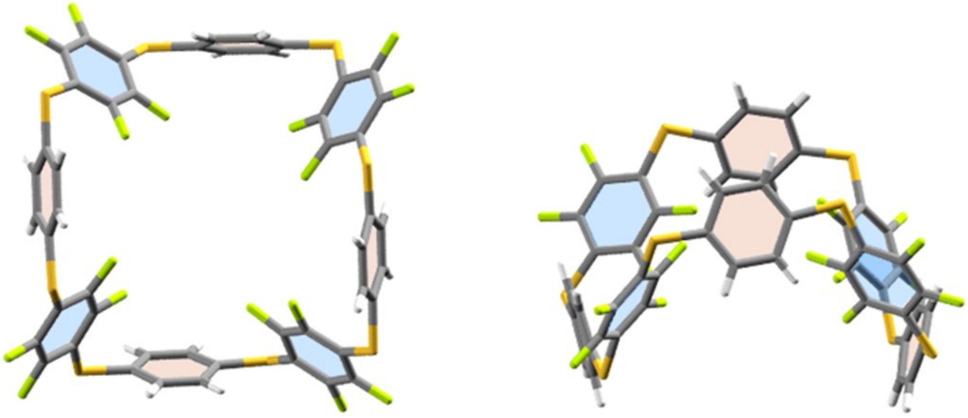

Top View **Side View**

Figure S206. Solid-state structure of **1a**–**DMF** viewed from above and side-on, with guests excluded for clarity.

Crystal System: Tetragonal

Space group: *$P4_2/c$*

Unit Cell Parameters: *a* = 19.8473(2) Å, *b* = 19.8473(2) Å, *c* = 13.19340(10) Å, β = 90°, V = 5197.08 Å3, Z = 4, Z' = 0.5

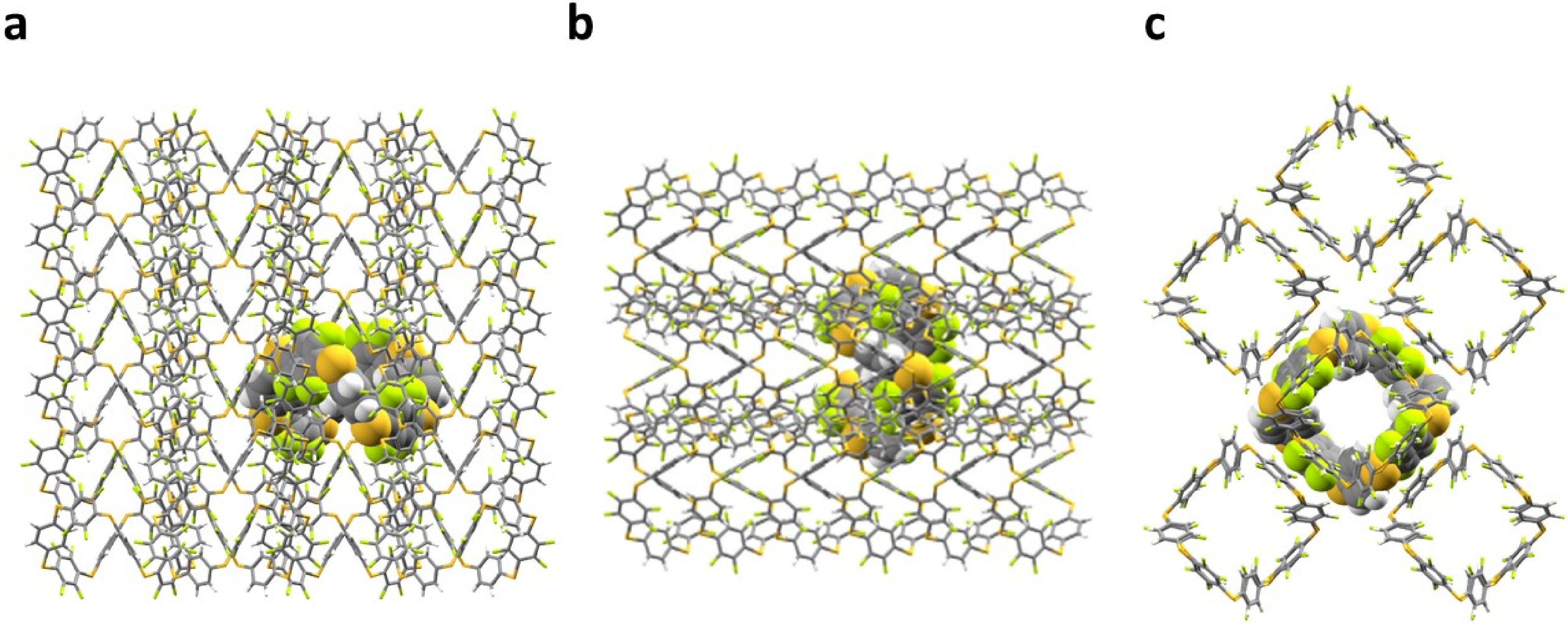

Figure S207. Solid-state superstructure of **1a**–**DMF** with guest excluded. A central molecule (space filling representation) is shown embedded in a section of the lattice in order to illustrate the crystal packing. Projections are viewed along the crystallographic (a) *a*-, (b) *b*-, and (c) *c*-axes.

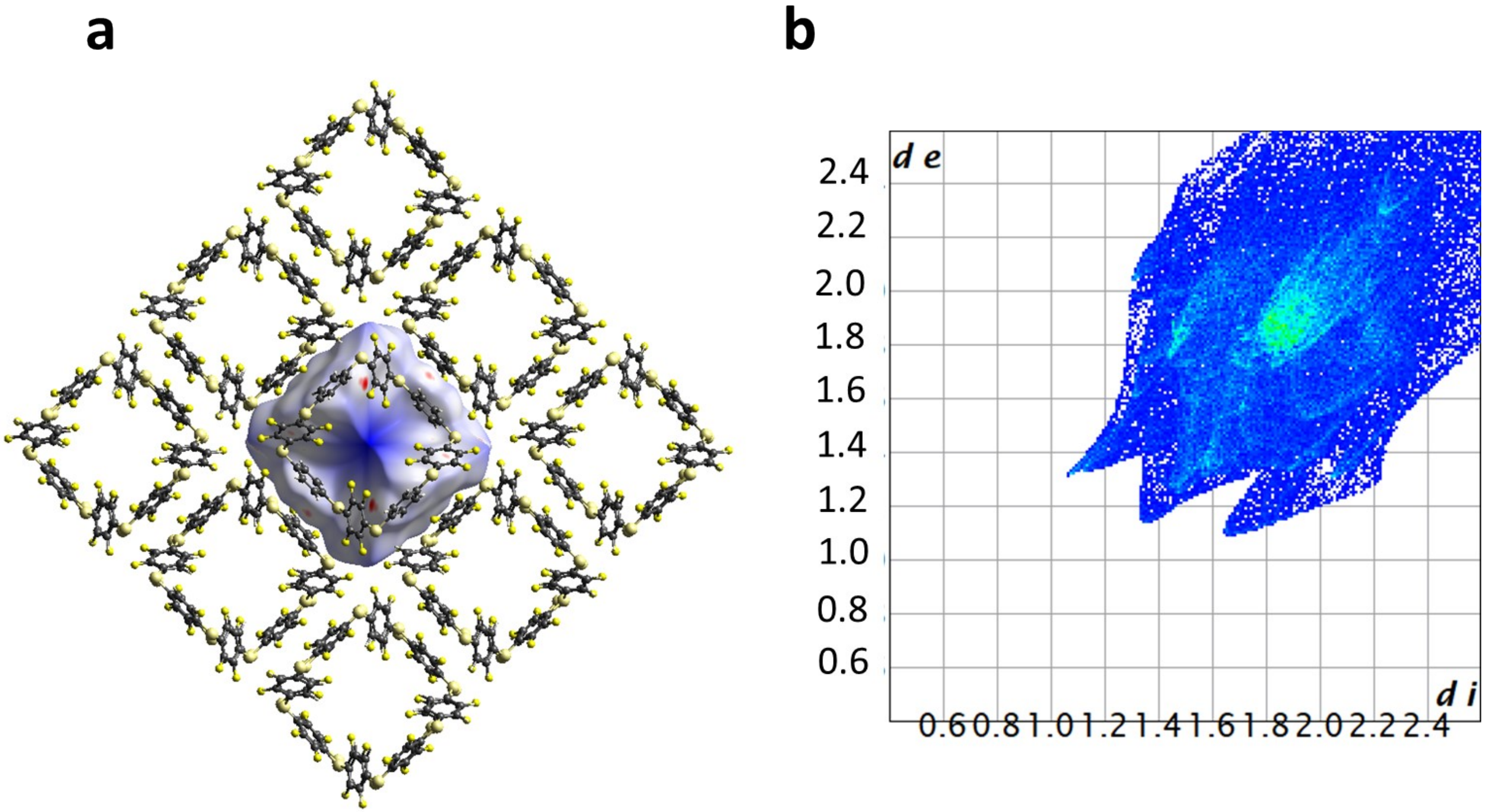

Figure S208. Solid-state superstructure of **1a**–**DMF** staircase polymorph: (a) Hirshfield surface plots of **1a**–**DMF** with generated close contacts within van der Waals Radii and guest excluded. (b) Fingerprint plot of **1a**–**DMF**.

5.6. 1b–$CHCl_3$

Method of Growth: Slow evaporation of a solution of **1b** in $CHCl_3$

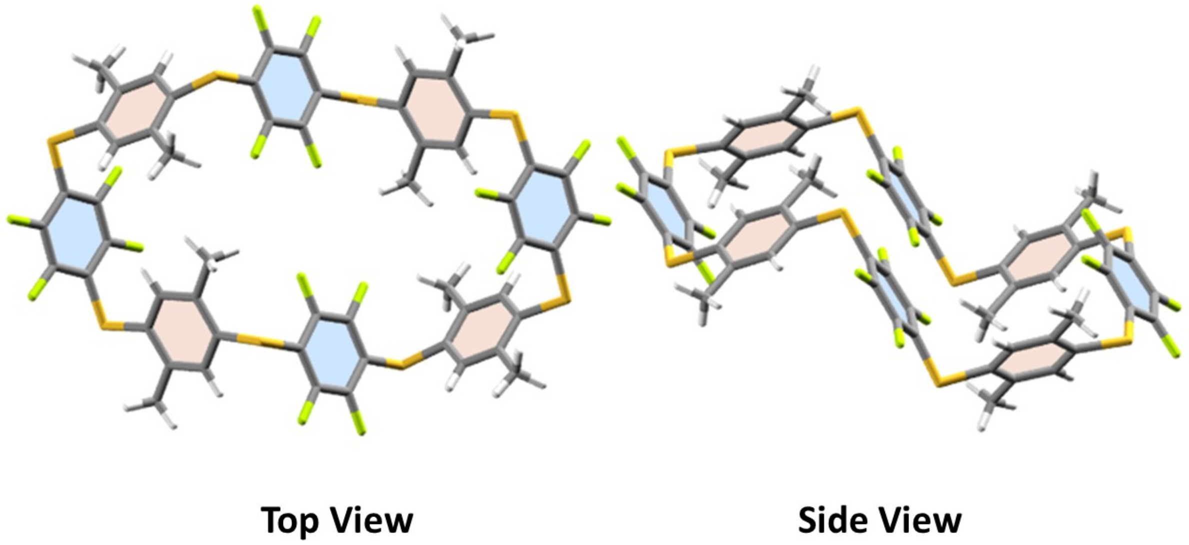

Figure S209. Solid-state structure of **1b–$CHCl_3$** viewed from above and side-on, with guests excluded for clarity.

Crystal System: Triclinic

Space group: *P1*

Unit Cell Parameters: a = 9.5286(2) Å, b = 10.3554(2) Å, c = 14.2500(4) Å, β = 89.177°, V = 1301.9 Å3, Z = 1, Z' = 0.5

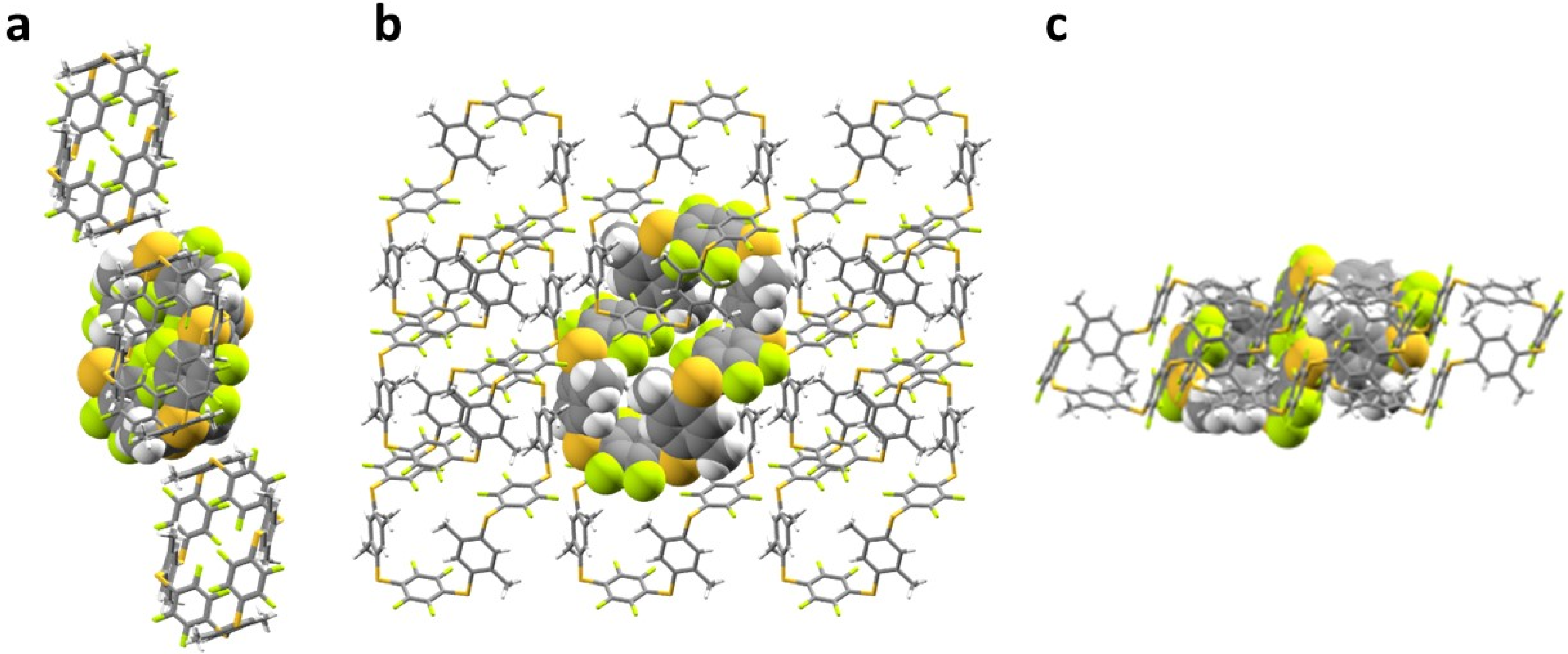

Figure S210. Solid-state superstructure of **1b** with guest excluded. A central molecule (space filling representation) is shown embedded in a section of the lattice in order to illustrate the crystal packing. Projections are viewed along the crystallographic (a) *a*-, (b) *b*-, and (c) *c*-axes.

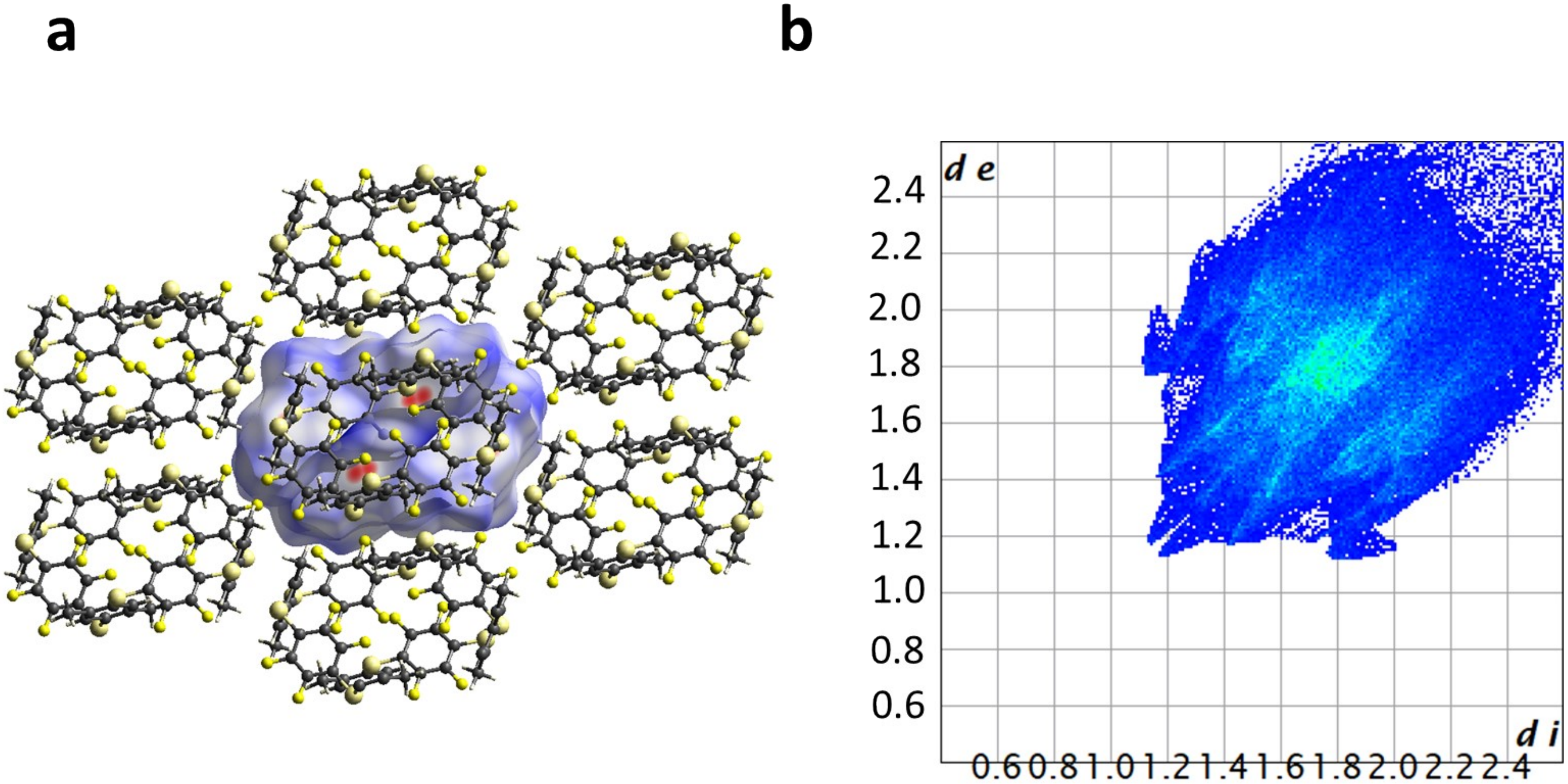

Figure S211. Solid-state superstructure of **1b–$CHCl_3$** staircase polymorph: (a) Hirshfield surface plots of **1b–$CHCl_3$** with generated close contacts within van der Waals Radii and guest excluded. (b) Fingerprint plot of **1b–$CHCl_3$**.

Table S2. Selected hydrogen-bond parameters.

D*—H···*A	***D*—H (Å)**	**H···*A* (Å)**	***D*···*A* (Å)**	***D*—H···*A* (°)**
**1a-Hexane**				
C13—H13···F27[i]	0.95	2.56	3.452 (14)	157.0
C15—H15···F69[ii]	0.95	3.15	3.806 (16)	127.8
C16—H16···F69[ii]	0.95	3.20	3.844 (17)	127.1
C16—H16···F70[ii]	0.95	2.26	3.194 (16)	167.1
C32—H32···F28[i]	0.95	2.25	3.071 (15)	144.7
C32—H32···F29	0.95	2.99	3.528 (15)	117.0
C33—H33···F28[i]	0.95	3.18	3.529 (14)	103.9
C35—H35···S6[iii]	0.95	3.48	3.748 (15)	98.8
C35—H35···F90[iv]	0.95	2.94	3.864 (16)	166.3
C52—H52···F48	0.95	3.29	3.533 (12)	97.2
C53—H53···F67[i]	0.95	2.74	3.654 (13)	162.5
C55—H55···F49[v]	0.95	2.68	3.477 (13)	141.4
C72—H72···F68[i]	0.95	2.09	2.949 (17)	149.9
C72—H72···F69	0.95	2.97	3.664 (18)	131.0
C73—H73···F68[i]	0.95	3.09	3.426 (18)	102.5
C75—H75···S2[iii]	0.95	3.30	3.696 (17)	107.7
**1a-THF**				
C22B—H22B···F18B	0.95	2.69	3.143 (6)	110.0
C22B—H22B···F37B[vi]	0.95	2.93	3.669 (5)	135.6
C22B—H22B···F20A[vii]	0.95	2.61	3.302 (5)	129.6
C23B—H23B···F19A[vii]	0.95	2.60	3.536 (5)	169.0
C23B—H23B···F20A[vii]	0.95	2.86	3.417 (5)	118.4
C25B—H25B···F40B	0.95	3.12	3.563 (6)	110.2
C25B—H25B···F18A	0.95	2.55	3.216 (5)	127.2
C25B—H25B···F20A[viii]	0.95	3.21	3.433 (6)	95.2
C26B—H26B···F17A	0.95	2.60	3.545 (5)	172.5
C26B—H26B···F18A	0.95	2.73	3.306 (5)	119.9
C26B—H26B···F19A[viii]	0.95	3.19	3.624 (7)	110.1
C42B—H42B···F39A[vii]	0.95	3.01	3.502 (6)	113.9
C42B—H42B···F40A[vii]	0.95	2.65	3.567 (5)	163.8
C43B—H43B···F38B[ix]	0.95	2.81	3.646 (5)	147.2
C43B—H43B···F39A[vii]	0.95	2.58	3.296 (6)	132.6
C45B—H45B···F17B[x]	0.95	2.75	3.432 (5)	129.6
C45B—H45B···F37A	0.95	2.94	3.414 (6)	112.3
C45B—H45B···F38A	0.95	2.55	3.466 (5)	162.7

C46B—H46B···F37A	0.95	2.52	3.199 (6)	128.8
C22A—H22A···F19B^{viii}	0.95	2.94	3.471 (6)	116.6
C22A—H22A···F17A^{viii}	0.95	2.93	3.530 (5)	122.3
C23A—H23A···S2B^{viii}	0.95	3.13	3.390 (5)	97.4
C23A—H23A···F19B^{viii}	0.95	2.96	3.492 (6)	116.5
C23A—H23A···F38A^{xi}	0.95	2.69	3.275 (5)	120.1
C42A—H42A···F20B^{xii}	0.95	2.60	3.266 (5)	127.9
C42A—H42A···F39A	0.95	2.71	3.265 (6)	117.9
C43A—H43A···F19B^{xii}	0.95	2.60	3.520 (5)	163.6
C43A—H43A···F20B^{xii}	0.95	2.87	3.397 (6)	116.2
C43A—H43A···F17A^{xii}	0.95	2.93	3.607 (5)	129.1
C45A—H45A···F18B^{x}	0.95	2.65	3.311 (6)	126.8
C45A—H45A···F38B^{xiii}	0.95	2.96	3.400 (5)	109.5
C45A—H45A···F20A^{xii}	0.95	3.16	3.590 (6)	109.2
C46A—H46A···F17B^{x}	0.95	2.62	3.558 (5)	168.0
C46A—H46A···F18B^{x}	0.95	2.85	3.408 (6)	118.8
C46A—H46A···F20B^{xiv}	0.95	2.92	3.305 (6)	105.9
**1a-Pyridine**				
C22—H22···F37^{vii}	0.95	3.24	3.6370 (15)	107.0
C23—H23···F37^{vii}	0.95	2.37	3.2148 (15)	147.3
C23—H23···F40	0.95	2.66	3.3979 (15)	134.7
C26—H26···F18	0.95	2.97	3.1647 (17)	92.7
C26—H26···N1B^{xv}	0.95	3.09	3.743 (2)	127.4
C42—H42···F38^{vii}	0.95	2.30	3.2348 (15)	168.3
C43—H43···F17^{x}	0.95	2.89	3.2943 (15)	106.8
C43—H43···N1A^{vii}	0.95	2.76	3.510 (2)	136.2
C45—H45···F39^{xvi}	0.95	2.46	3.3941 (14)	167.9
C45—H45···F40^{xvi}	0.95	3.04	3.6103 (15)	119.6
C46—H46···S3^{xvii}	0.95	3.28	3.5178 (12)	96.9
C46—H46···F40^{xvi}	0.95	2.78	3.4783 (15)	131.1
C2B—H2B···N1B^{xviii}	0.95	2.75	3.570 (2)	144.9
C4B—H4B···S3	0.95	3.21	3.4860 (15)	98.7
C5B—H5B···F19^{xix}	0.95	2.61	3.1428 (17)	116.2
C6B—H6B···F19^{xix}	0.95	2.60	3.1533 (18)	117.2
C6B—H6B···N1B^{xv}	0.95	2.92	3.489 (2)	120.1
C2A—H2A···F17^{xii}	0.95	2.78	3.427 (2)	126.3
C3A—H3A···F18	0.95	2.97	3.528 (2)	118.9
C4A—H4A···F18	0.95	2.72	3.4075 (18)	129.5

C6A—H6A···F38	0.95	2.80	3.3272 (18)	115.6
**1a-DMF**				
C22A—H22A···F18B	0.95	2.78	3.385 (3)	122.1
C22A—H22A···F19B^{x}	0.95	2.54	3.066 (3)	115.1
C22A—H22A···F20B^{x}	0.95	3.03	3.858 (3)	145.9
C23A—H23A···F17B	0.95	2.81	3.556 (3)	135.9
C23A—H23A···F18B	0.95	3.30	3.649 (3)	104.1
C23A—H23A···F19B^{x}	0.95	2.74	3.167 (3)	108.5
C25A—H25A···F20B^{i}	0.95	2.89	3.379 (3)	112.9
C26A—H26A···F19B^{i}	0.95	2.98	3.752 (3)	139.2
C26A—H26A···F20B^{i}	0.95	2.96	3.418 (3)	110.7
C25B—H25B···S1A^{xx}	0.95	3.35	3.860 (3)	116.1
C25B—H25B···F20A^{xxi}	0.95	2.52	3.136 (3)	122.4
C25B—H25B···F20A^{xx}	0.95	2.49	3.274 (3)	140.3
C26B—H26B···F20A^{xx}	0.95	3.14	3.590 (3)	111.1
C26B—H26B···F17B^{xx}	0.95	3.26	3.964 (3)	132.2
**(1b)**				
C23—H23···F37^{xxii}	0.95	2.71	3.6229 (19)	160.9
C23—H23···F38^{xxii}	0.95	2.70	3.2455 (18)	117.4
C27—H27B···F18	0.98	2.71	3.407 (2)	128.3
C27—H27C···F38^{xxii}	0.98	2.88	3.403 (2)	114.1
C28—H28A···F19^{xxiii}	0.98	2.95	3.5979 (19)	124.3
C28—H28A···F40	0.98	2.58	3.390 (2)	139.6
C43—H43···S4^{xxiv}	0.95	3.31	3.5872 (15)	99.5
C43—H43···F17^{x}	0.95	3.09	3.4331 (17)	103.2
C47—H47B···S4	0.98	2.93	3.1432 (16)	93.1
C47—H47B···F20^{xxv}	0.98	3.02	3.6472 (17)	123.3
C47—H47C···F17^{xxiii}	0.98	3.14	3.5415 (18)	106.0
C47—H47C···F39	0.98	2.56	3.3628 (19)	139.7
C48—H48B···F37^{xxvi}	0.98	2.98	3.6479 (19)	126.6
C48—H48C···S1^{x}	0.98	2.78	2.9766 (17)	91.7
C48—H48C···F19^{xxvii}	0.98	2.71	3.532 (2)	141.5

Symmetry code(s): (i) *x*, *y*, *z*-1; (ii) *x*-1/2, -*y*+1/2, *z*+1; (iii) *x*, *y*, *z*+1; (iv) -*x*+1/2, *y*+1/2, *z*+1/2; (v) -*x*+1, -*y*+1, *z*+1/2; (vi) -*x*+2, -*y*+2, -*z*+1; (vii) *x*+1, *y*, *z*; (viii) -*x*+1, -*y*+2, -*z*+1; (ix) -*x*+1, -*y*+1, -*z*; (x) -*x*+1, -*y*+1, -*z*+1; (xi) -*x*, -*y*+1, -*z*; (xii) -*x*, -*y*+1, -*z*+1; (xiii) *x*-1, *y*-1, *z*; (xiv) *x*-1, *y*-1, *z*-1; (xv) -*x*+1, -*y*+1, -*z*+2; (xvi) *x*-1, -*y*+3/2, *z*-1/2; (xvii) *x*, -*y*+3/2, *z*-1/2; (xviii) -*x*, -*y*+1, -*z*+2; (xix) -*x*+2, -*y*+1, -*z*+2; (xx) -*y*+1, *x*+1/2, *z*+1/2; (xxi) *y*-1/2, -*x*+1, *z*+1/2; (xxii) -*x*+1, -*y*+2, -*z*; (xxiii) *x*-1, *y*, *z*; (xxiv) -*x*, -*y*+2, -*z*+1; (xxv) -*x*+2, -*y*+1, -*z*+1; (xxvi) -*x*, -*y*+2, -*z*; (xxvii) *x*-2, *y*+1, *z*.

6. UV-Vis Titrations and Binding Calculations

Experimental. Dichloromethane and methanol were of analytical grade and used as supplied. THF was distilled (66°C) under nitrogen which removed any residual BHT stabiliser and reproducibly lowered water contents to 190±20 ppm for samples stored under nitrogen; $CHCl_3$ was distilled (61°C) from activity I basic alumina under nitrogen; DMF was distilled from CaH_2 under reduced pressure (146°C, 20 mbar) then stored under nitrogen. Solid reagents were weighed under nitrogen. Dilutions, solvent handling, and UV-vis titration samples were carried out under cushions of argon. Karl-Fischer titrations confirmed residual water levels of: 85±2 ppm (CH_2Cl_2), 110±2 ppm ($CHCl_3$), 225±2 ppm (MeOH), 190±20 ppm (THF), 710-1150 ppm (DMF) for solvents handled in this way both before and after UV-vis titrations. All solids were weighed to an accuracy of 0.001 mg on a Sartorius CPA26P balance under nitrogen. The water of crystallisation content of $NR_4X{\bullet}nH_2O$ salts depends on their drying, handling and batch (they appreciably deliquescent). The value of n was determined by quantified ^1H NMR spectroscopy in DMSO-d_6 containing mesitylene as an internal standard on the same day/batch as for the UV-vis titration. A correction was made for the residual water present in the DMSO-d_6 NMR solvent in each assay and provided the following compositions for the material titrated (which were used in the isotherms binding calculations): $NBu_4F{\bullet}2.46H_2O$, $NBu_4Cl{\bullet}1.69H_2O$ and $NMe_4F{\bullet}2.47H_2O$. The butyl ammonium salts were easily soluble in THF to at least 10 mM. The methyl derivative $NMe_4F{\bullet}2.47H_2O$ was insoluble in THF. Concentrations of up to 6–7 mM could be realised in 4:4:1 CH_2Cl_2/THF/MeOH (containing 200 ppm water) or in DMF (if it contained at least 1000 ppm water) after prolonged sonication/warming for the latter.

UV-vis Studies: General Procedure. Studies were conducted on an Agilant-Cary 5000 UV-vis spectrometer. Nominal 10 μM solutions of macrocycle were prepared by weighing ca. 1.2 mg amounts (accurate to 0.001 mg) of pure 8-ring macrocycle **1a** into a 100 mL volumetric flask and adding the required solvent under argon. The sample and reference cuvettes were filled with the same solvent also under an argon, sealed, zeroed and an initial background spectrum established. Under a cushion of argon, macrocycle solution **1a** (3.00 mL) was transferred by graduated syringe to the cleaned and dried UV-vis sample cuvette, which was then sealed and transferred to the spectrometer. Aliquots of potential guests were added by microlitre syringes under argon cushions, the cell contents agitated with a fine glass rod, the cuvette re-sealed under argon and spectra acquired after each aliquot (for details below see individual runs). Post titration water contents were assayed by Karl-Fisher titrations and found to be in the ranges quoted above.

Control studies indicated no significant background deviation from linearity in the THF or DMF solvent background absorbance (0±0.005) in the window 290—500 nm. Guest binding resulted in increased absorbance at 290–300 nm (plus an extra band at ca. 370 nm for NR_4F guests, R = Bu, Me). Primary guest binding absorbance data at 295 nm, and for the ammonium fluorides also the absorbance at 370 nm were fitted. All isotherm fitted primary data are given in the ESI file: 'UV-vis PRIMARY DATA and BINDING FITS' (Excel). Data were fitted using the established non-linear least squares regression approaches already described.[19,20,21] Figure S212 summarises only.

Titration of macrocycle 1a and $CHCl_3$. Concentration of corona macrocycle **1a** = 10.4 μM in THF (200 ppm water, 3.00 mL total volume); neat $CHCl_3$ used (112 ppm water). A total of 155 μL of chloroform was added (62000 equivalents w.r.t. **1a**). The spectra and primary data are shown in the file 'UV-vis PRIMARY DATA and BINDING FITS' (Excel). A fit to a 1:1 binding isotherm (A_{295} data) provided K = 3.0(7) M^{-1} (R^2 = 0.98), with ε_{HG} = 5.8(1) ×10^4 M^{-1} cm^{-1}. A replicate analysis, using 10.8 μM **1a**, provided K = 4.2(4) M^{-1} (R^2 = 0.99) with ε_{HG} = 5.92(4) ×10^4 M^{-1} cm^{-1}. These two sets of data were averaged for the main paper.

Titration of macrocycle 1a and DMF. Concentration of corona macrocycle **1a** = 10.4 μM in THF (200 ppm water, 3.00 mL total volume); neat DMF used (1960 ppm water). A total of 155 μL of DMF was added (64400 equivalents w.r.t. **1a**). The spectra and primary data are shown in the file 'UV-vis PRIMARY DATA and BINDING FITS' (Excel). A fit to a 1:1 binding isotherm (A_{295} data) provided K = 3(1) M^{-1} (R^2 = 0.97), with ε_{HG} = 5.8(1) ×10^4 M^{-1} cm^{-1}. A replicate analysis provided K = 3.6(5) M^{-1} (R^2 = 0.98) with ε_{HG} = 6.04(5) ×10^4 M^{-1} cm^{-1}. Further replicates with using 10.8 μM **1a** and drier DMF (870 ppm) provided K = 7.0(8) M^{-1} (R^2 = 0.98), with ε_{HG} = 5.50(2) ×10^4 M^{-1} cm^{-1} and K = 7.8(7) M^{-1} (R^2 = 0.99) with ε_{HG} = 5.62(4) ×10^4 M^{-1} cm^{-1}. The latter two data sets were averaged for the main paper, but comment is made on the effect of water on the value in the text (first two replicates). The spectra and primary data are shown in the file 'UV-vis PRIMARY DATA and BINDING FITS' (Excel).

Titration of macrocycle 1a and pyridine. Concentration of corona macrocycle **1a** = 10.4 μM in THF (200 ppm water, 3.00 mL total volume); concentration of pyridine guest solution = 7.45 mM in THF (200 ppm water). A total of 205 μL of pyridine guest solution was added (49.0 equivalents w.r.t. **1a**). The spectra and primary data are shown in the file 'UV-vis PRIMARY DATA and BINDING FITS' (Excel). A fit to a 1:1 binding isotherm (A_{295} data) provided K = 7(1) × 10^3 M^{-1} (R^2 = 0.95), with ε_{HG} = 5.86(8) ×10^4 M^{-1} cm^{-1}. A replicate analysis, using 10.8 μM **1a**, provided K = 9(1) × 10^3 M^{-1} (R^2 = 0.98) with ε_{HG} = 5.6(3) ×10^4 M^{-1} cm^{-1}. These two values were averaged for the main paper.

Titration of macrocycle 1a and NBu_4Cl•1⅔H_2O. Concentration of corona macrocycle **1a** = 10.4 µM in THF (170 ppm water content, 3.00 mL total volume); concentration of NBu_4Cl•1.69H_2O guest solution = 3.58 mM in THF (200 ppm water). A total of 105 µL of NBu_4Cl•1.69H_2O guest solution was added (12.0 equivalents w.r.t. **1a**). The spectra and primary data are shown in the file 'UV-vis PRIMARY DATA and BINDING FITS' (Excel). A fit to a 1:1 binding isotherm (A_{295} data) provided K = 2.7(4) × 10^4 M^{-1} (R^2 = 0.97), with ε_{HG} = 6.08(5) ×10^4 M^{-1} cm^{-1}. A replicate analysis provided K = 2.6(2) × 10^4 M^{-1} (R^2 >0.99) with ε_{HG} = 6.15(2) ×10^4 M^{-1} cm^{-1}. These two values were averaged for the main paper.

Titration of macrocycle 1a and NBu_4F•2½H_2O. Concentration of corona macrocycle **1a** = 10.4 µM in THF (170 ppm water content, 3.00 mL total volume); concentration of NBu_4F•2.07H_2O guest solution = 3.64 mM in THF (200 ppm water). A total of 515 µL of NBu_4F•2.46H_2O guest solution was added (60.1 equivalents w.r.t. **1a**). The spectra and primary data are shown in the file 'UV-vis PRIMARY DATA and BINDING FITS' (Excel). A fit to a 1:1 for the initial binding isotherm (A_{295} data) provided K = 1.6(2) × 10^4 M^{-1} (R^2 = 0.97), with ε_{HG} = 7.1(1) ×10^4 M^{-1} cm^{-1}. A replicate analysis provided K = 1.5(2) × 10^4 M^{-1} (R^2 = 0.99) with ε_{HG} = 7.1(1) ×10^4 M^{-1} cm^{-1}. The 1:1 fit for the second binding isotherm (A_{370} data) provided K = 9(4) × 10^2 M^{-1} (R^2 = 0.94) with ε_{HG} = 6(2) ×10^4 M^{-1} cm^{-1}. A replicate analysis of this provided K = 1.1(3) ×10^3 M^{-1} (R^2 = 0.98) with ε_{HG} = 6(1) ×10^4 M^{-1} cm^{-1}. These values were averaged for the main paper.

Titration of macrocycle 1a and NMe_4F•2½H_2O: Concentration of macrocycle **1a** = 10.8 µM in DMF (1150 ppm water content, 3.00 mL total volume); concentration of NMe_4F•2.47H_2O guest solution = 6.08 mM in THF (1150 ppm water, 63.9 mM). A total of 595 µL of NMe_4F•2.47H_2O guest solution was added (112 equivalents w.r.t. **1a**). A replicate was conducted using concentration of macrocycle **1a** = 10.8 µM in DMF (1150 ppm water content (63.9 mM), 2.70 mL total volume); concentration of NMe_4F•2.47H_2O guest solution = 6.08 mM in THF (1150 ppm water). A total of 950 µL of NMe4F•2.47H_2O guest solution was added. The spectra and primary data are shown in the file 'UV-vis PRIMARY DATA and BINDING FITS' (Excel). While the UV-vis spectra clearly showed the formation of a new species associated with absorption at 370 nm but the presence of water appreciably interferes with the data analysis and accurate K values could not be determined. Attempts to use drier DMF (710 ppm water) limited the solubility of NMe_4F•2.47H_2O and useful spectra could not be attained. Both the corona macrocycle **1a** and NMe_4F•2.47H_2O were soluble in a 4:4:1 mixture of THF/CH_2Cl_2/MeOH (190 ppm water) but no binding of any kind (as determined by the absorbance at 295 and 370 nm) resulted in this solvent mixture.

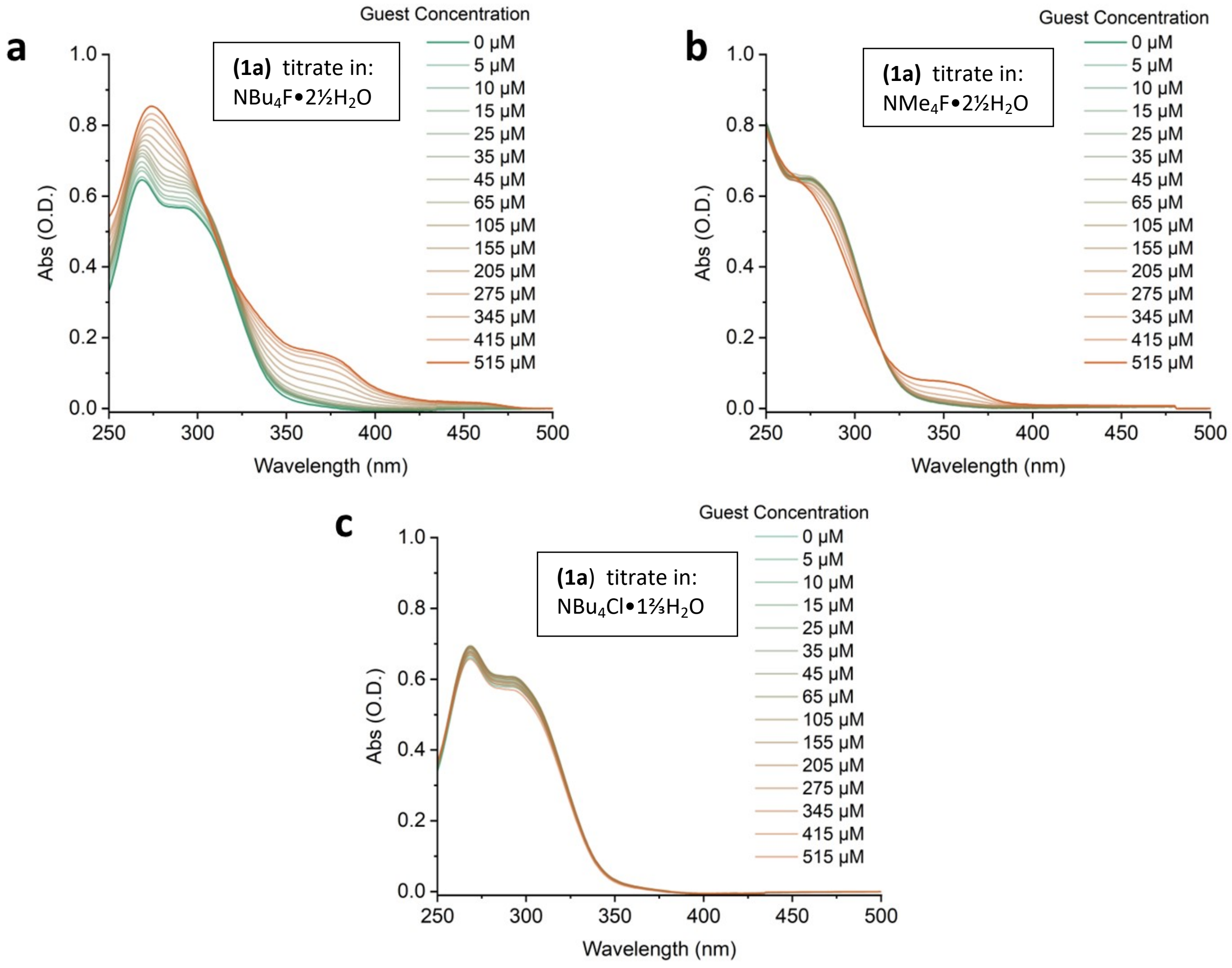

Figure S212. UV-Vis titrations of **1a** with ammonium halide guests (see 'UV-vis PRIMARY DATA and BINDING FITS' (Excel) for full data).

7. NMR Kinetic Analysis

7.1. ^{19}F NMR Kinetic investigation of one-pot macrocycle formation

Experimental. A solution of $\mathbf{C_6F_6}$ (1.6 mg, 8.6 µmol) in pyridine-d$_5$ (1.0 mL) was added to a solid mixture of 1,4-dithiobenzene (**2a**) (12.0 mg, 86 µmol, 10-fold excess) solid NMe_4F•$4H_2O$ (20.0 mg, 120 µmol, 14-fold excess) at 22 °C. The NMR tube was shaken, the kinetics clock started, and the sample was transferred to the NMR spectrometer. Quantitative ^{19}F NMR spectra (376.5 MHz) were recorded every 6-12 mins (raw data not referenced to C_6F_6 shift). Data to 85 mins were used, by which time 81% of all $\mathbf{C_6F_6}$ had been consumed; after this time competing oligomerisation reactions were observed (data thus not used).

Data handling. Data were fitted to a variety of rate law types using non-least squares regression, as described by Billo,[21] using time (min)/^{19}F integration data until best fits were attained (as judged by R^2 and the kinetic parameter standard deviations).

Analysis. The 10 spectra (Figure S213a, showing signals labelled **D**, **F** and **H** corresponding to the intermediates in Scheme S2 later) acquired show relatively simple behaviour: (i) C_6F_6 (s, δ –162.6) smoothly disappears (ii) Initially, a dominant single species grows in (br s, δ –132.8, w½ ~40 Hz). (iii) This is followed by a pair of signals (br s, δ –132.4 and br, δ –132.2, both with w½ ~45 Hz), who grow in at the same rate (and therefore assigned to the same specie). (iv) Finally, a sharp signal (s, δ –131.8) (**H**) becomes apparent whose ^{19}F NMR shift, in pyridine-*d*$_5$, is reminiscent both free macrocycle **1a** (s, δ –131.9) and the macrocycle **1a** in the presence of 10 equivalents of NMe_4F•$4H_2O$ (s, δ –131.9). Aside from this only two minor impurity species (s, δ –132.0 and broader s, δ –131.8) are present (marked m, they account for ≤ 1.5% of the total fluoride integration and were not modelled, but see text later). The template NMe_4F•$4H_2O$ is not appreciably soluble in pyridine and its (particulate) presence causes some line broadening; the concentrations of the modelled entities are not affected though.

The disappearance of $\mathbf{C_6F_6}$ (signal **A**) has best fit to the rate law (1) (R^2 >0.99).[22,23] This indicates an undetected reactive intermediate (**B**),[24] derived from $\mathbf{C_6F_6}$, that reacts by two distinct onward processes, one involving the dithiol (k_{1obs}) and the other a second molecule of $\mathbf{C_6F_6}$ (k_{2obs}).

$$\text{Rate} = k_{1obs}[\mathbf{A}] + k_{2obs}[\mathbf{A}]^2 \qquad (1)$$

where $k_{1obs} = k_1[HSC_6H_4SH]$ with the later approximately constant (in excess)

Fitting consumption of $\mathbf{C_6F_6}$ (**A**) over 0-85 min provided: k_{1obs} = 4.7(28) × 10^{-3} min^{-1} and k_{2obs} = 2.0(3) × 10^{-3} $(\mu M)^{-1}$ min^{-1}, where the numbers in parentheses are the standard deviation in the last digit (Figure S213b). Signal **D** is clearly derived from **A** (via undetected **B**). While **D** is also itself reactive (complicating the analysis), during the first 30 mins it is the major specie (>75% of all $\mathbf{C_6F_6}$ derived products). The appearance of **D**, in this period, gives a good fit to first-order kinetics (R^2 >0.99) with k_{3obs} = 1.4(2) × 10^{-1} min^{-1} (Figure S213c).

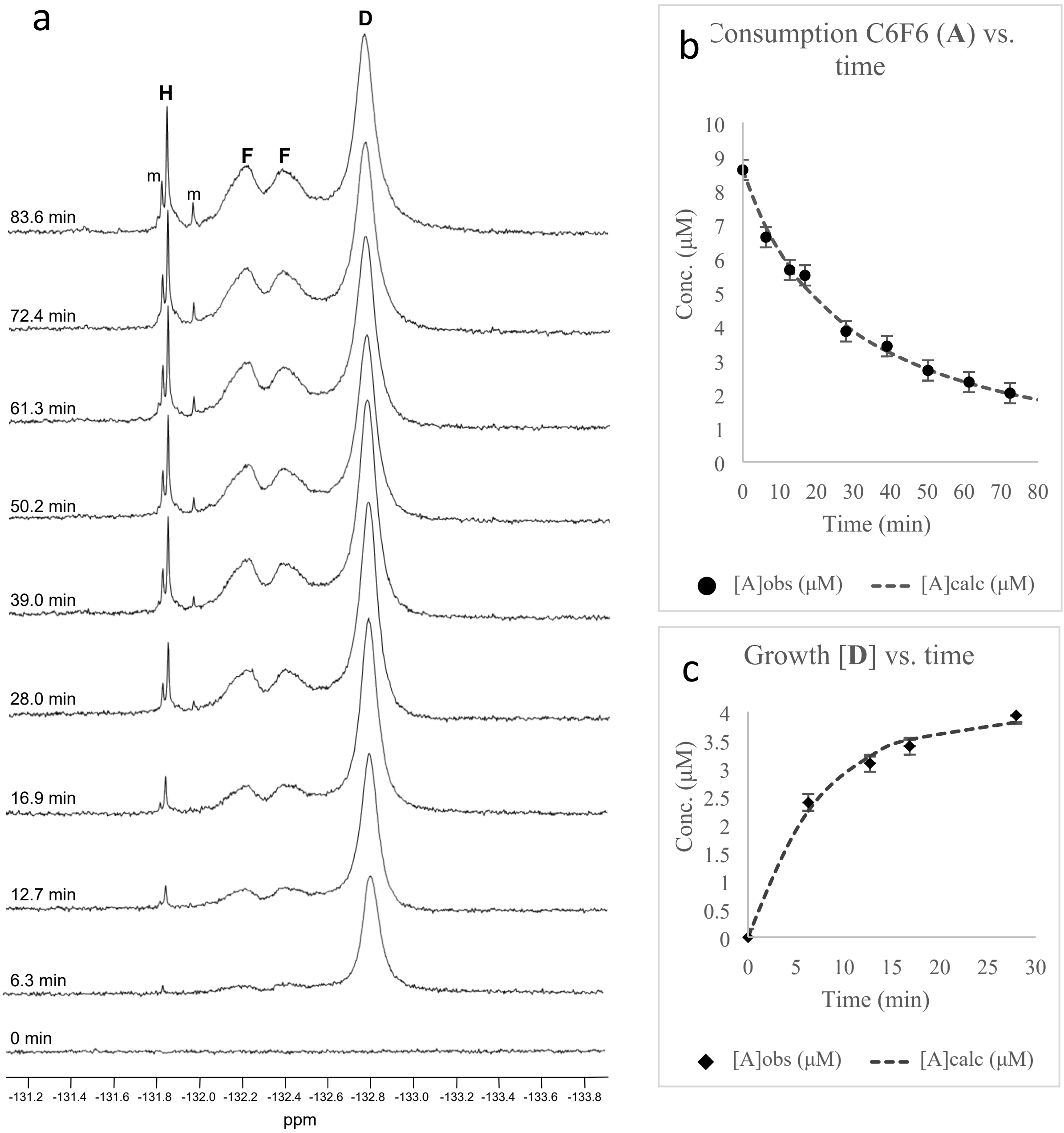

Figure S213. Reaction monitoring of the one-pot formation of **1a**: a) Partial ^{19}F NMR spectrum of the reaction mixture (–131 to –134 ppm); b) second-order kinetic consumption of C_6F_6 (signal **A**, not shown); c) first-order growth of signal **D**. The identity of the intermediates (**A-H**) is shown in Scheme S2. See also file 'Kinetic PRIMARY DATA and RATE FITS' (Excel).

As exact kinetic forms are not available for the complete cascade leading to the macrocycle (signal **H**), we sought an approximate numerical simulation.[15] To avoid over parameterising the simulation we used just three generic rate constants for the processes shown in Scheme S1. Several simulations were trialled, with that of Scheme S2, providing the best fit to the observed ^{19}F NMR behaviour.

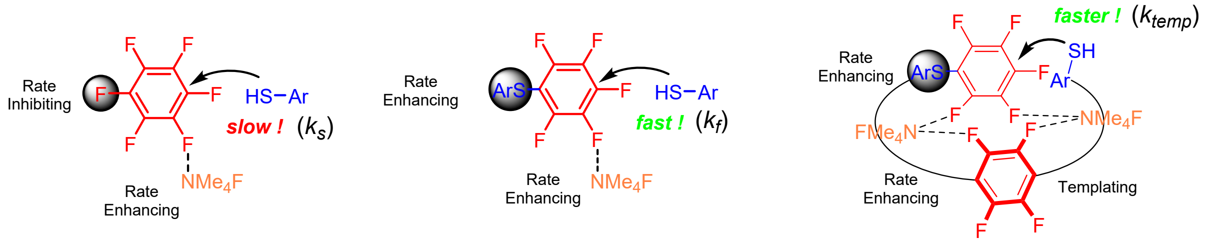

Scheme S1. Generic rate constants used in the simulation model (Scheme S2). See also file 'Kinetic PRIMARY DATA and RATE FITS' (Excel).

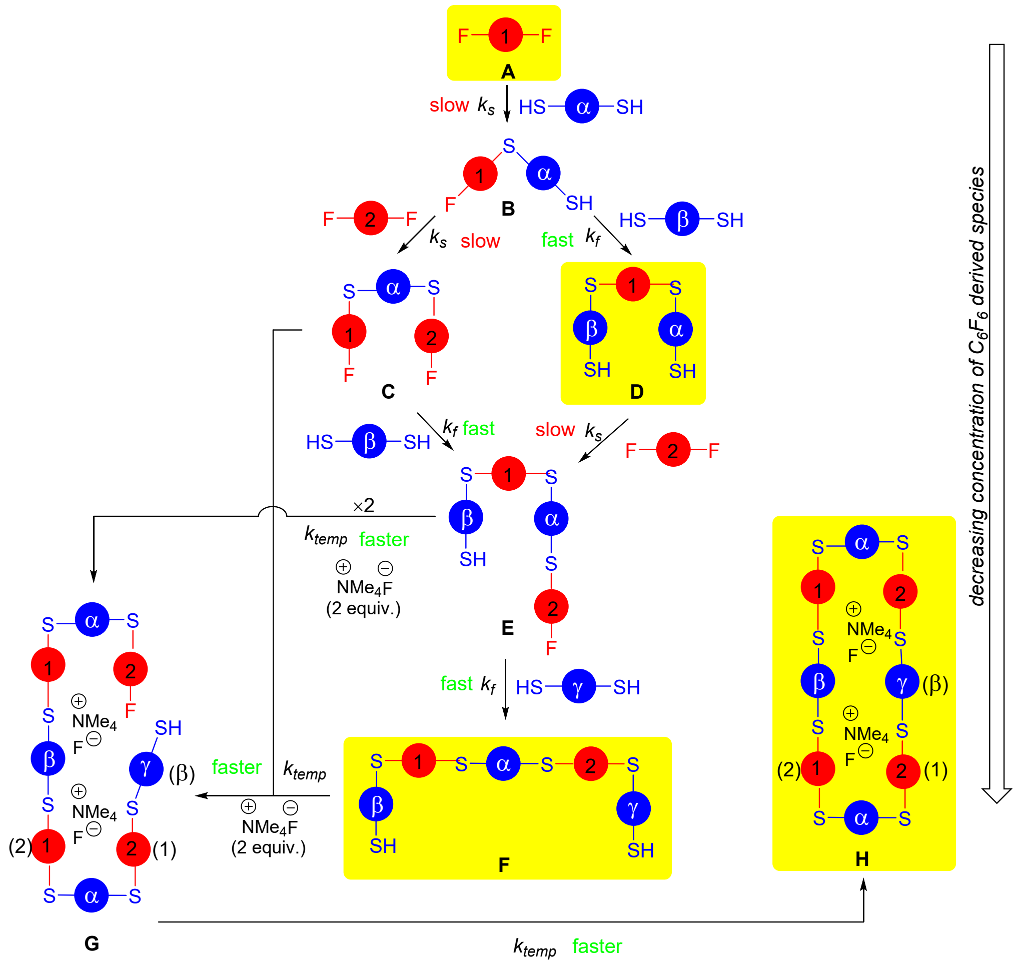

Scheme S2. Numerical simulation model, only the species in yellow boxes have detectable concentrations. The 1,4-C_6F_4 and 1,4-C_6H_4 units are shown schematically: the numbers (1-4) and Greek

characters (α-γ) show the origins of the fragments in macrocycle assembly. Note undetected **C** is equivalent to isolated fragment **3a**, used in synthetic studies (Section 2).

The model of Scheme S2 was simulated by numerical integration of the individual fundamental kinetic steps using a time interval of 1 min. The results of the non-linear least squares regression of this process are summarised in Figure S214, where it is compared to the NMR observed concentrations.

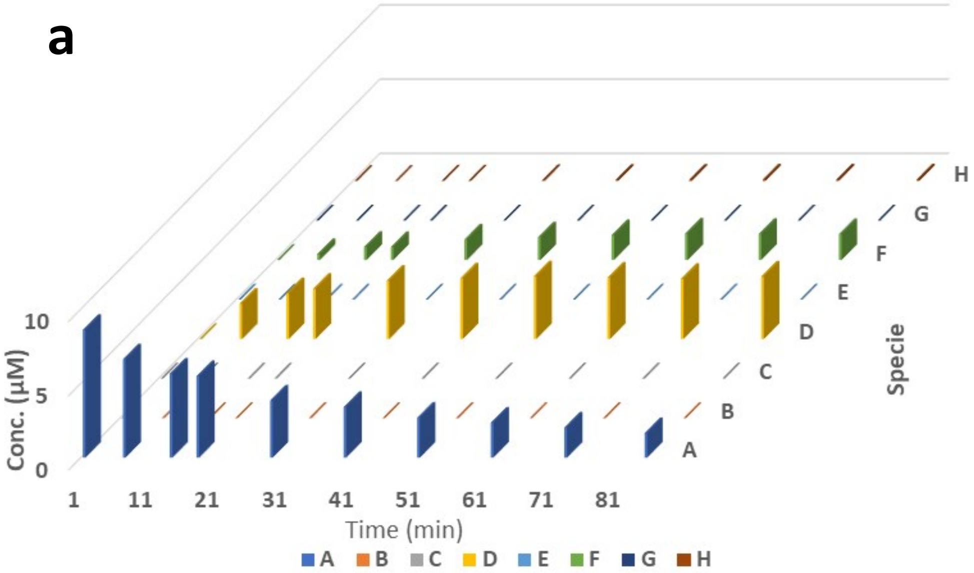

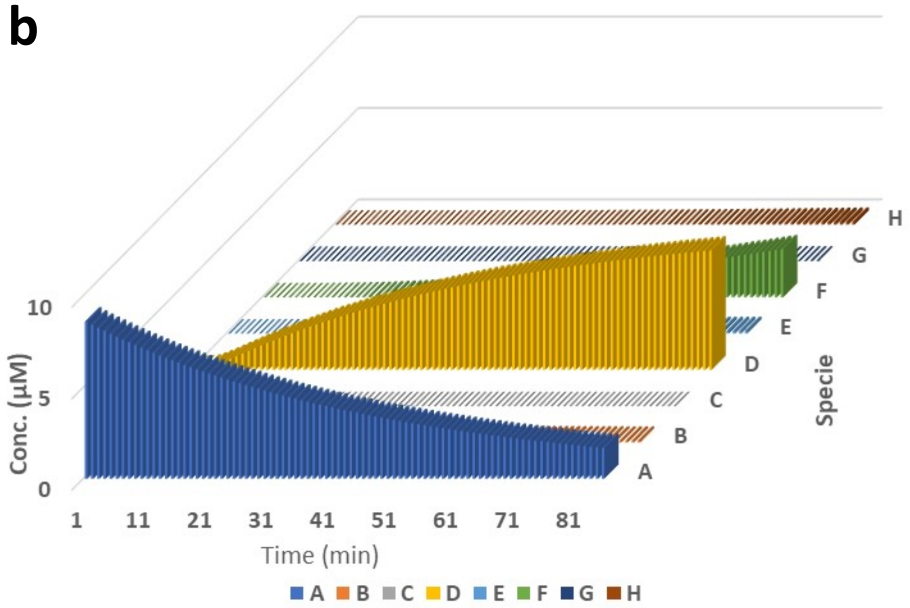

Figure S214. (a) ^{19}F NMR spectroscopically observed concentrations. (b) Numerical simulation of the behaviour of the model of Scheme S2. See also file 'Kinetic PRIMARY DATA and RATE FITS' (Excel).

The very simple model of Schemes S213-S214 predicts the observed experimental behaviour quite well. Importantly, all the non-observed intermediates of Scheme S2 are predicted to be present at less than ca. 0.1 µM in the reaction mixture (0.3 µM for **B** in the early stages of the reaction). The fit of Figure S214 provided: k_s = 2.4 ×10^{-4} (µM)$^{-1}$ min^{-1}, k_f = 4.6 ×10^{-3} (µM)$^{-1}$ min^{-1} and k_{temp} = 2.2 ×10^{-1} (µM)$^{-1}$ min^{-1} (min^{-1} for **G** to **H**). Attempts to replace k_{temp} with k_f led to a significantly worse fit with significantly slower macrocycle production, reinforcing the proposed kinetic catalytic template synthesis. Allowing for reagent molecularity, at the outset of the reaction both numerical and exact approaches gave the same values for the absolute values of k_1 ~5.4 x 10^{-5} (µM)$^{-1}$ min^{-1} and k_2 ~2.5 x 10^{-4} (µM)$^{-1}$ min^{-1} determined from exact rate laws, within experimental error. This is also in line with the experimental need to add **C_6F_6** to an excess of the dithiol in the one-pot preparation of the macrocycle.

The template NMe_4F•$4H_2O$ is poorly soluble in pyridine alone; we cannot detect it in control samples. There is a small excess initial increase in the reaction mixture signal at δ –170.8 (assigned to HF•pyridine),[25] this value is beyond that expected for HF generation by the reaction kinetics at the start of the reaction, indicating that the conditions do facilitate some NMe_4F•$4H_2O$ dissolution. This is no more than 2 µM [i.e. K_{SP}(NMe_4F•$4H_2O$) ~4 ×10^{-12} M^2 in pyridine]. However, this is still sufficient NMe_4F flux to provide the required kinetic templating catalytic effects observed. Finally, although their very low concentrations prevent their explicit characterisation and modelling, we propose the two impurities (m) we see in Figure S213a to also be corona macrocycles. The reaction of **F** with **C_6F_6** would provide the next chain growth homolog, which could either dimerise or react with **E** to provide traces of the isolated S_{12}-corona[4H,4F]arene (**1a'**) or other higher aromatic ring containing corona macrocycles.

7.2. Studies of Rate Acceleration by NMe_4F•$4H_2O$

To understand the intrinsic electronic rate acceleration provided by NMe_4F catalysis in S_NAr reactions (in the absence of any macrocyclic templating effects), the reaction of $PhSC_6F_5$ (**7**) in pyridine in the presence and absence of $NMe_4F.4H_2O$, was investigated. We used the model system of Figure S215, as no macrocyclisation effects are possible here.

Experimental. A solution of known $PhSC_6F_5$ (**7**)[26] (2.7 mg, 9.8 µmol) in pyridine-d_5 (1.0 mL) at 22 °C was prepared and its ^{19}F NMR spectrum was recorded. Thiophenol (10 µL, 10.8 mg, 98 µmol, 10-fold excess) was placed in the tube containing **7**. The NMR tube was shaken, the kinetics clock

started, and the sample was promptly transferred to the NMR spectrometer. Quantitative ^{19}F NMR spectra (376.5 MHz) were recorded every 6 min between 3.5 and 45 mins of reaction. Similar runs were repeated in the presence of suspended $NMe_4F•4H_2O$ (22.6 mg, 137 µmol, 14-fold excess).

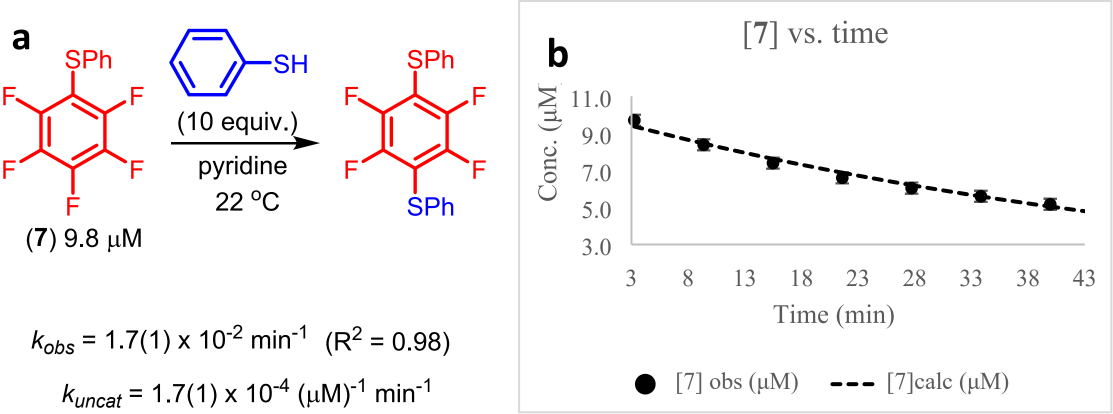

Figure S215. Rate constants for the uncatalysed displacement of 1,4-related fluorides to ArS groups using model compound **7**: (a) schematic of reaction, $[\mathbf{7}]_0$ = 9.8 µM and $[PhSH]_0$ = 98 µM; b) Fit of [**7**] (mM) over time. See also file 'Kinetic PRIMARY DATA and RATE FITS' (Excel).

Analysis. In the absence of any catalytic kinetic promoter, conversion of **7** was slow (50% conversion after 45 min). A first-order fit to these data gives k_{obs} = 1.7(1) × 10^{-2} min^{-1} (R^2 = 0.98) providing a derived second-order rate constant of k_{uncat} = 1.7(1) × 10^{-4} $(µM)^{-1}$ min^{-1} (Figure S215). In the presence of $NMe_4F•4H_2O$, thiophenol substitution of **7** was extremely accelerated and the equivalent S_NAr reaction achieved >98% conversion within 3.5 min. The rapidity of the reaction, in the presence of the excess PhSH (necessary to enforce first order behaviour), prevented the attainment of accurate rate data, but the acceleration (≥25 × k_{uncat} based on initial rate) is of similar magnitude to the acceleration observed in the one-pot study (k_f in Section 7.1). These data are consistent with a significant electronic effect, as calculated (Section 8, S_NAr acceleration through increased sp^3 character/decreased aromaticity). These effects are *in addition* to any templating effects also provided by the catalyst. Conversely, in the absence of any kinetic catalytic promoter, we found the reaction of $\mathbf{C_6F_6}$ (**A**) with PhSH to be immeasurably slow at room temperature, but we could estimate its maximum uncatalyzed rate constant to be ~1 ×10^{-5} $(µM)^{-1}$ min^{-1} at most.

Using the rate constants from Schemes S1-S2 and Figure S215 allowed us to quantify the relative rates of the S_NAr reactions associated with the macrocycle formation cascade and associated processes. The relative rates are summarised in Scheme S3.

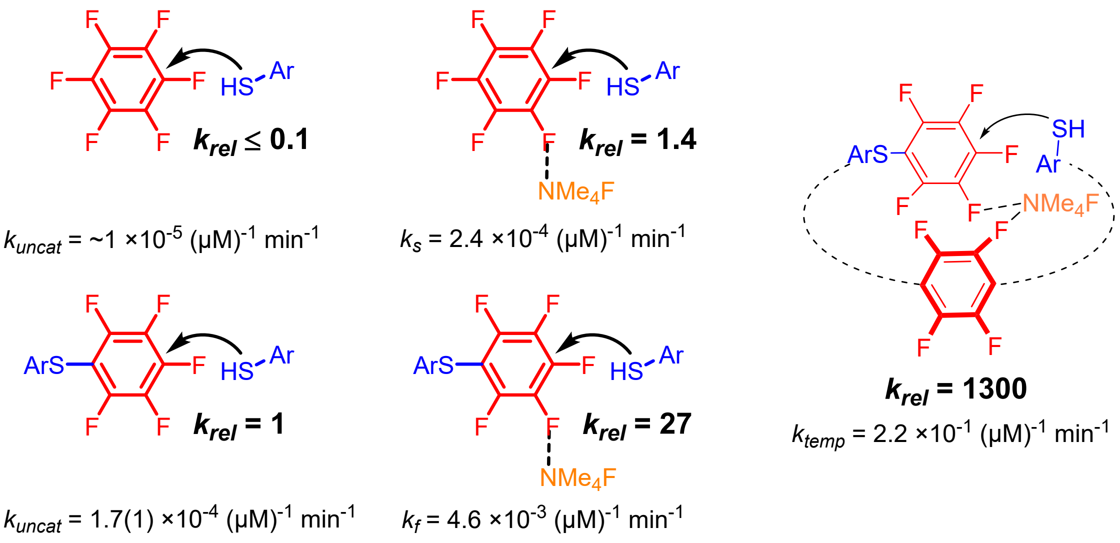

Scheme S3. Relative rates of important processes in the kinetic catalytic cascade forming macrocycle **H** (equivalent to **1a** formation) derived from the kinetic studies.

8. Computational Analysis

General Comments

All of the density functional theory (DFT) calculations carried out in this study were performed using the Q-Chem quantum chemical software package.[27]

C_6F_6…NMe_4F Binding Study

The geometry of the C_6F_6-NMe_4F interaction was optimised at the M06-2X/6-31++G(d,p) level of theory as shown in Figure S216. Two of the ammonium methyl hydrogens interact most strongly with just three fluorines of the C_6F_6 (Figure S216b). The fluoride ion is bound in a contact ion pair with three ammonium methyl hydrogens, but also has a π-interaction with the C_6F_6 unit (Figure 216a). The calculated interaction energy between NMe_4F and C_6F_6 is ca. 12 kcal mol^{-1} (*in vacuo*, 0 K) and is significant, based on the energies calculated for the adduct and its components. Natural population analysis (NPA) indicates that the fluorine atoms of the C_6F_6 unit polarise, and each lose about 1/9th of an electron when in the presence of NMe_4F (Figure 216c).[28]

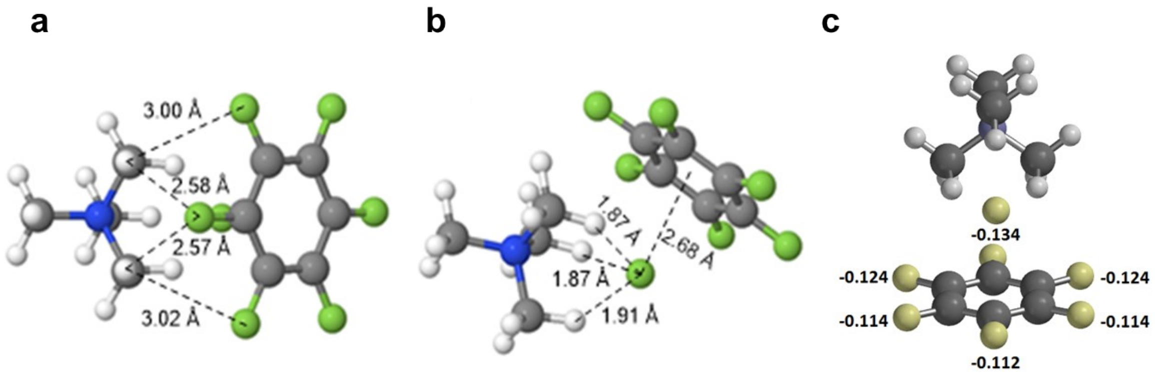

Figure S216. Calculated interaction geometry for $\mathbf{C_6F_6}$…NMe_4F viewed from above the $\mathbf{C_6F_6}$ unit (a), from its side (b), and shown with selected NPA deviations from isolated $\mathbf{C_6F_6}$ (c); 2.68 Å is the distance to the centre of the $\mathbf{C_6F_6}$ benzenoid (the F…C_{aryl} distances are all 3.02 ± 0.12 Å). Calculated Cartesian coordinates are given in Table S17.

Conformations of corona macrocycle S_8-[4H,4F]-arene (1a)

Stochastic conformational sampling of the free **1a** was performed using the Merck Molecular Force Field (MMFF94)[29] as implemented within the Chem3D 20.0 software package. The crystal structure coordinates of **1a**-*conformer1* (as attained from CH_2Cl_2-hexanes) were imported and one hundred initial conformations were generated by displacing the atoms by a maximum random off-set of 20 nm from their initial positions and then relaxing these geometries using the default tolerances. The resulting geometries were then optimised to minimum energy structures at the DFT M06-2X/6-31++G(d,p) level of theory.[30] Structures with energies within 5 kcal mol^{-1} of the minimum were retained, and single-point energies were calculated at the M06-2X/6-311++G(2df,2p) level with and without a conductor-like polarizable continuum model (C-PCM) solvent dielectric of 36.7.[31] Vibrational zero-point energy corrections were then added to conformations with relative energies within 1 kcal mol^{-1} based on harmonic frequency calculations at the lower level of theory, identifying **1a**-*conformer2* and **1a**-*conformer3* as the two lowest energy conformers (Figure S217). For comparison, the conformation of the CH_2Cl_2/hexanes derived X-ray solid-state structure **1a**-*conformer1* (excluding the disordered partial hexanes present in the macrocyclic cavity) had a relative energy of +8.9 and +5.3 kcal mol^{-1} *in vacuo* and in solvent, respectively, when calculated at the same level of theory without zero-point energy corrections. We detect only one species in pure (sublimed or prepared using chemospecific NH_4F catalysis) samples of **1a** by ^{1}H, ^{19}F and ^{13}C NMR spectroscopy at room temperature, consistent with interconversion of *conformers2/3* on the NMR timescale. The final coordinated for **1a**-*conformer1* and *conformer2* are given in Tables S18-S19.

Electronic UV-vis spectra were calculated using adiabatic linear-response time-dependent density functional theory (TDDFT) at the M06-2X/6-311++G(d,p) level.[32] The resultant spectra and associated major transitions are shown in Figures S218-220.

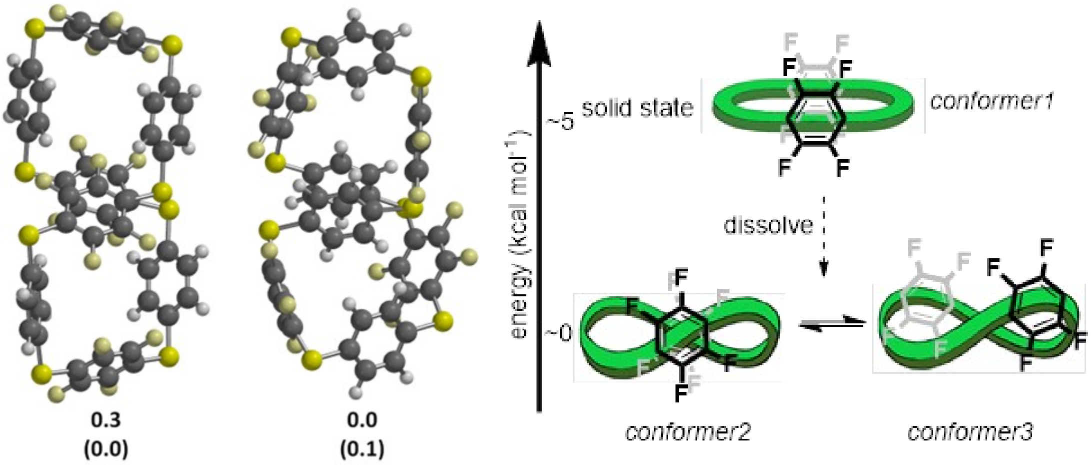

Figure S217. The left shows the DFT calculated minimum energy structures for **1a**-*conformer2* and **1a**-*conformer 3* with calculated relative energies in kcal mol^{-1} both *in vacuo* and with solvent (C-PCM, approximating to *N,N*-dimethylformamide, in parentheses). The right shows a schematic representation of the relationship between the solid state and solution conformers of **1a** (n = 4). Listings of the Cartesian coordinates of the calculated **1a**-*conformers 2, and 3* are given in Tables S18-19.

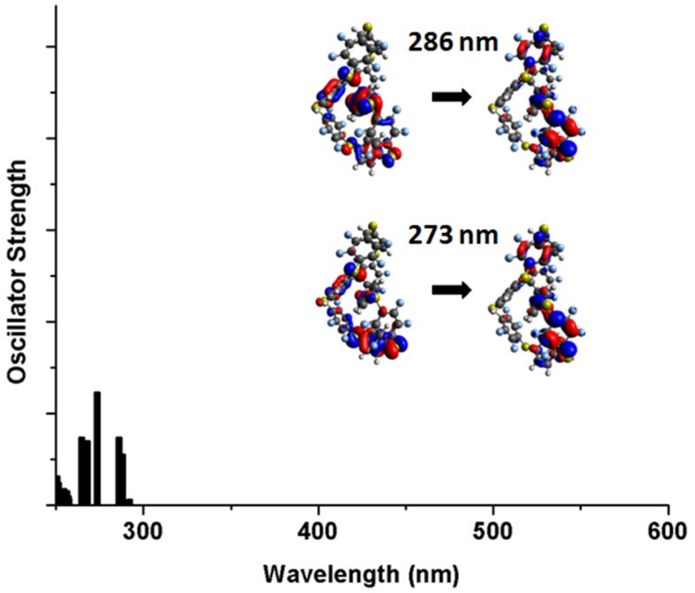

Figure S218. The calculated UV-vis spectrum of the **1a**-*conformer1* with the dominant donor and acceptor oritals shown for the two highest intensity transitions.

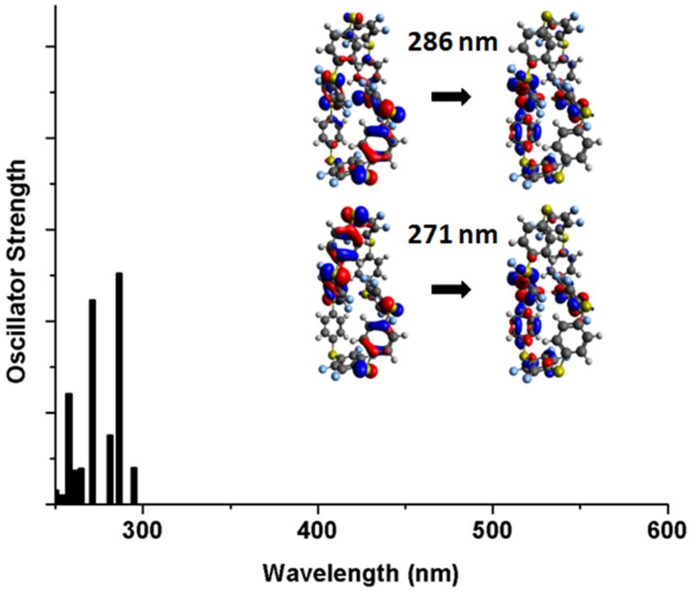

Figure S219. The calculated UV-vis spectrum of the **1a**-*conformer2* with the dominant donor and acceptor orbitals is shown for the thighest-intensity transitions.

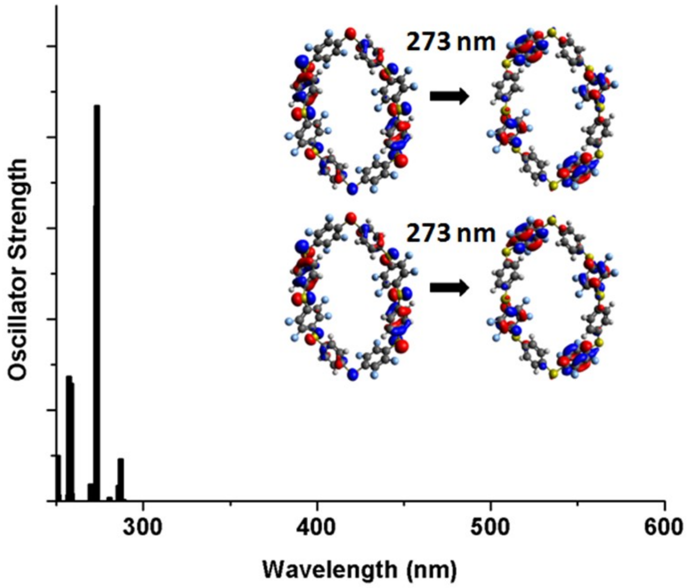

Figure S220. The calculated UV-vis spectrum of **1a**-*conformer3* with the dominant donor and acceptor orbitals shown for the two highest intensity transitions.

The TDDFT calculations of **1a**-*conformer2* and **1a**-*conformer3* indicate that these conformational isomers have similar spectral band positions in the UV-Vis region. The experimentally observed UV/Vis transitions are therefore assigned to interconverting **1a**-*conformer2/3* in solution in the absence of strongly binding guests.

8.1 Steric vs. Electronic Effects on Macrocyclisation

To understand steric and electronic effects in the fragments **3a-d** rotational barriers and charge distributions were calculated at the M06-2X/6-31++G(d,p)//M06-2X/6-311++G(2df,2p) level of theory from a transition state search starting from initial structures generated from the application of the freezing string method with 15 nodes between the optimised *trans* and *cis* terminal fluorobenzenoid isomers[33] of species **3a** to **3d** (Scheme S4). Outputs from the calculations are given in Table S4, while the calculated coordinates of all the intermediates used are given in Tables S5-S16.

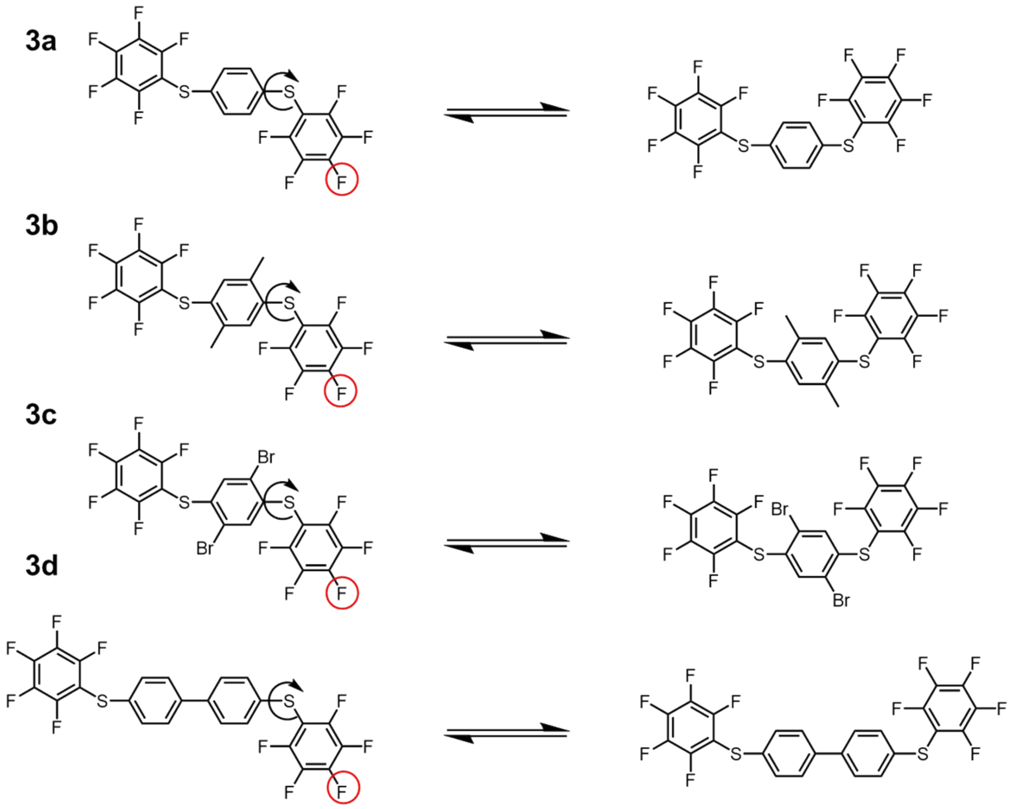

Scheme S4. The red circle shows the C-F bond selected for NPA charge calculations (to compare leaving group ability), while the arrow indicates the C-S bond whose rotational barrier was determined.

Table S4. Selected fluorine atom NPA charges and transition state energy barriers for rotation in **3**.

Species	Fluorine Charge (a.u.)	C-S Rotational Barrier (kcal mol^{-1})
**3a**	-0.317	1.1
**3b**	-0.317	5.9
**3c**	-0.316	4.8
**3d**	-0.317	0.2

Table S5. Calculated Cartesian coordinates (Å) for the DFT minimum energy structure of **3a** with the terminal fluorobenzenoids on opposite sides of the central aryl unit.

Atom	x-coordinate	y-coordinate	z-coordinate
C	4.7221202432	-1.3422683812	-0.9296580508
C	5.1585768083	0.8941378562	0.6446183756
C	3.7954199346	-0.3011149612	-0.9773675801
C	5.8519198838	-1.2782015840	-0.1222005691
C	6.0673589085	-0.1582110965	0.6688788269
C	4.0459510171	0.8190372902	-0.1803875005
S	2.3936424663	-0.3844547106	-2.0567543005
C	1.0734655295	-0.1595069010	-0.8733292234
C	-1.0725795799	0.1652532095	0.8710041479
C	0.0664498627	0.7600347016	-1.1708213305
C	1.0050357635	-0.9187335716	0.2950977528
C	-0.0656467334	-0.7543909933	1.1684041643
C	-1.0042419946	0.9243410530	-0.2975230903
H	0.1344051712	1.3651535604	-2.0692274903
H	1.7766144074	-1.6508208106	0.5149000330
H	-0.1335951004	-1.3594443658	2.0668522759
H	-1.7758204315	1.6564468938	-0.5172671276
S	-2.3923182388	0.3910933551	2.0547675696
C	-3.7947871592	0.3029045101	0.9766254920
C	-6.0686151532	0.1516425126	-0.6662683947
C	-4.0448525798	-0.8201670467	0.1836323201
C	-4.7228121988	1.3427647594	0.9265330377
C	-5.8535521946	1.2745835722	0.1207208317
C	-5.1584509418	-0.8994597302	-0.6396680360
F	-3.2003452873	-1.8485530135	0.1992166663
F	-5.3726167295	-1.9781154789	-1.3881347502
F	-7.1417299071	0.0799001010	-1.4441487235
F	-6.7235504692	2.2806499336	0.0936572752
F	-4.5439505546	2.4429125445	1.6551318486
F	5.3730651104	1.9699414165	1.3970770861
F	3.2026911549	1.8484731370	-0.1934915300
F	7.1394164982	-0.0905873431	1.4485840318
F	6.7205889212	-2.2854690132	-0.0973573753
F	4.5427435701	-2.4397714049	-1.6620966748

Table S6. Calculated Cartesian coordinates (Å) for the DFT minimum energy structure of **3a** with the terminal fluorobenzenoids on same sides of the central aryl unit.

Atom	x-coordinate	y-coordinate	z-coordinate
C	4.7187496544	-0.5753275115	-0.0067473971
C	3.4765684652	1.8527222895	-0.4880964616
C	3.5172529598	-0.5696888780	-0.7147728801
C	5.3029128885	0.6002263402	0.4508573868

C	4.6779366608	1.8163531683	0.2112889484
C	2.9183164280	0.6703566286	-0.9512635651
S	2.8222919319	-2.0750518826	-1.3387736103
C	1.2184846306	-2.0253799282	-0.5486136566
C	-1.2999739320	-2.0408626669	0.6325855235
C	1.0949956403	-1.8233004998	0.8267779982
C	0.0852923265	-2.2470187399	-1.3328052620
C	-1.1728272289	-2.2733525343	-0.7376735398
C	-0.1666071435	-1.8119851821	1.4143294424
H	1.9808190681	-1.6668835777	1.4353534623
H	0.1879242307	-2.3906477996	-2.4040038057
H	-2.0585599997	-2.4581961068	-1.3383314969
H	-0.2754415861	-1.6269048530	2.4783040971
S	-2.9173965533	-2.0686109263	1.4055078665
C	-3.5594708106	-0.5507849987	0.7440324958
C	-4.5826092594	1.8539817430	-0.2837829837
C	-4.6136325364	-0.5447869448	-0.1688257486
C	-3.0372673774	0.6853559766	1.1302662857
C	-3.5294745511	1.8792342318	0.6233285050
C	-5.1291554650	0.6406837233	-0.6795991997
F	-5.1569037768	-1.6873649806	-0.5849497618
F	-6.1325780783	0.6199443593	-1.5532021708
F	-5.0649193725	2.9901004891	-0.7716026351
F	-3.0068002518	3.0431772644	0.9991017140
F	-2.0339576886	0.7425729315	2.0040688908
F	2.8837255855	3.0193910093	-0.7252722952
F	1.7782481721	0.7409799768	-1.6337017464
F	5.2280427227	2.9437237906	0.6445645411
F	6.4511809721	0.5646472258	1.1211186095
F	5.3426805732	-1.7219254094	0.2553667754

Table S7. Calculated Cartesian coordinates (Å) for the DFT transition state structure of **3a**.

Atom	x-coordinate	y-coordinate	z-coordinate
C	4.2137548499	-1.0069584258	-0.2023752658
C	5.4808896151	1.4366627408	0.0780151249
C	3.7512817610	0.0746642859	-0.9547520214
C	5.2759516163	-0.8838905132	0.6814844244
C	5.9121457643	0.3443675934	0.8189049484
C	4.4090013857	1.2942091707	-0.7955475248
S	2.3940787749	-0.1073873852	-2.0864293107
C	1.0640487282	-0.2782056816	-0.8941611472
C	-1.0334320830	-0.5400678625	0.9117762284
C	0.6102644344	0.8402031558	-0.1901053920
C	0.4700386174	-1.5247409774	-0.6985561525
C	-0.5814438772	-1.6564000145	0.2075805173
C	-0.4332689052	0.7074966455	0.7207186375
H	1.0717554889	1.8089910703	-0.3579786820
H	0.8381552309	-2.3889853819	-1.2415334644

H	-1.0478990786	-2.6224698244	0.3703130996
H	-0.7904166509	1.5686432589	1.2768431747
S	-2.3678738513	-0.7088754952	2.1012656500
C	-3.7449975214	-0.3732601291	1.0275055955
C	-5.9522053081	0.1370709619	-0.6331602457
C	-4.2701927213	-1.3606902338	0.1924157755
C	-4.3602748968	0.8785743736	1.0045263172
C	-5.4537986126	1.1401540212	0.1879686071
C	-5.3585802964	-1.1193610471	-0.6341889353
F	-3.7255901269	-2.5757854156	0.1629968201
F	-5.8378035646	-2.0783533298	-1.4219874454
F	-6.9945357544	0.3782428903	-1.4182662677
F	-6.0217509850	2.3431114130	0.1819597024
F	-3.9080866443	1.8700424656	1.7690624504
F	6.0865632082	2.6128180036	0.2179227326
F	4.0183314172	2.3695048882	-1.4768385021
F	6.9301958918	0.4718813010	1.6603638730
F	5.6932586470	-1.9290026943	1.3908349964
F	3.6308045702	-2.1981996215	-0.3163915100

Table S8. Calculated Cartesian coordinates (Å) for the DFT minimum energy structure of **3b** with the terminal fluorobenzenoids on opposite sides of the central aryl unit.

Atom	x-coordinate	y-coordinate	z-coordinate
C	3.9843704839	-1.0048615956	-0.7088265300
C	5.9280129596	0.6930444341	0.2903677482
C	3.8237415948	0.3708118504	-0.8867018159
C	5.0771876958	-1.5334667531	-0.0354826603
C	6.0532483426	-0.6790256802	0.4640741376
C	4.8200312050	1.2023848997	-0.3753981369
S	2.4316538704	1.0410410557	-1.7586293322
C	1.0941678031	0.4523879828	-0.7212282530
C	-1.0941678031	-0.4523879828	0.7212282530
C	0.9348031431	0.8995661126	0.6000680016
C	0.1754189142	-0.4233984141	-1.2998260431
C	-0.9348031431	-0.8995661126	-0.6000680016
C	-0.1754189142	0.4233984141	1.2998260431
H	0.3462917812	-0.7636772249	-2.3175506104
H	-0.3462917812	0.7636772249	2.3175506104
S	-2.4316538704	-1.0410410557	1.7586293322
C	-3.8237415948	-0.3708118504	0.8867018159
C	-6.0532483426	0.6790256802	-0.4640741376
C	-4.8200312050	-1.2023848997	0.3753981369
C	-3.9843704839	1.0048615956	0.7088265300
C	-5.0771876958	1.5334667531	0.0354826603
C	-5.9280129596	-0.6930444341	-0.2903677482
F	-4.7232698836	-2.5250660803	0.4986335956
F	-6.8589910264	-1.5102101826	-0.7762415750
F	-7.1052418201	1.1733007140	-1.1053375038

F	-5.2055900620	2.8483775439	-0.1205531625
F	-3.0770292515	1.8512064499	1.1908004912
F	6.8589910264	1.5102101826	0.7762415750
F	4.7232698836	2.5250660803	-0.4986335956
F	7.1052418201	-1.1733007140	1.1053375038
F	5.2055900620	-2.8483775439	0.1205531625
F	3.0770292515	-1.8512064499	-1.1908004912
C	-1.8912843655	-1.8548610262	-1.2613857120
H	-2.1784845049	-2.6612298921	-0.5810967210
H	-1.4389106803	-2.2912441273	-2.1538656637
H	-2.8102434599	-1.3433380984	-1.5701856040
C	1.8912843655	1.8548610262	1.2613857120
H	1.4389106803	2.2912441273	2.1538656637
H	2.1784845049	2.6612298921	0.5810967210
H	2.8102434599	1.3433380984	1.5701856040

Table S9. Calculated Cartesian coordinates (Å) for the DFT minimum energy structure of **3b** with the terminal fluorobenzenoids on same sides of the central aryl unit.

Atom	x-coordinate	y-coordinate	z-coordinate
C	-4.6959480937	0.3610896781	0.9054450053
C	-3.4314939082	-2.0149472414	0.2618305592
C	-3.6491838117	0.3925003167	-0.0159847444
C	-5.1146317111	-0.8231977576	1.4991550908
C	-4.4750295287	-2.0137616957	1.1798752150
C	-3.0389254723	-0.8231444013	-0.3318027398
S	-3.1343721866	1.9202310836	-0.7556766408
C	-1.4038479684	1.9223037035	-0.2917725479
C	1.3122177810	1.9904104621	0.2630532071
C	-0.9980636998	1.9686618945	1.0514307044
C	-0.4676889966	1.9350888082	-1.3253999788
C	0.9055644904	1.9877888399	-1.0806375764
C	0.3759307304	1.9844143695	1.2966204721
H	-0.8212004808	1.8948328219	-2.3522639066
H	0.7303955081	1.9830976846	2.3239263460
S	3.0410430556	2.0726487269	0.7268167669
C	3.6219528253	0.5559840954	0.0142773241
C	4.5489823269	-1.8317807584	-1.1436671471
C	4.6790130045	0.5528387530	-0.8958003278
C	3.0543097978	-0.6777501819	0.3390455548
C	3.4963123435	-1.8610041451	-0.2366477031
C	5.1475695323	-0.6220425767	-1.4704544759
F	5.2606166333	1.6964789445	-1.2553984882
F	6.1505011792	-0.5933489085	-2.3445680187
F	4.9812423316	-2.9589629432	-1.6958472281
F	2.9301262449	-3.0214936134	0.0826519809
F	2.0602499517	-0.7466178209	1.2218572477
F	-2.8276939540	-3.1580540099	-0.0513450959
F	-2.0511904341	-0.8658341702	-1.2232847507

F	-4.8582324532	-3.1495722032	1.7502206859
F	-6.1088636820	-0.8204528472	2.3836623461
F	-5.3154795535	1.4865615274	1.2590006384
C	1.8742582697	2.0092757816	-2.2328791569
H	2.7140090034	2.6810039327	-2.0343755626
H	1.3730969521	2.3346406852	-3.1465807278
H	2.2869945688	1.0106320481	-2.4183787695
C	-1.9674355223	1.9718160307	2.2032107891
H	-1.4836191727	2.3442005661	3.1082849635
H	-2.3307124307	0.9590226172	2.4140931368
H	-2.8390411434	2.5962083793	1.9883045677

Table S10. Calculated Cartesian coordinates (Å) for the DFT transition state structure of **3b**.

Atom	x-coordinate	y-coordinate	z-coordinate
C	4.7733855101	-1.0018759083	-0.2399322940
C	5.2593155123	1.7210409544	-0.0763558492
C	3.8473215212	-0.1214659426	-0.8017513012
C	5.9154635909	-0.5460839664	0.4044034737
C	6.1566473902	0.8203603312	0.4850007458
C	4.1228015263	1.2441527520	-0.7161624583
S	2.4678903690	-0.7189330581	-1.7312281757
C	1.0538593817	-0.8906779923	-0.6401812769
C	-1.4035569762	-1.3376234540	0.6023313956
C	0.9486770886	-0.6526077425	0.7389533229
C	-0.0568373825	-1.3460529749	-1.3630499032
C	-1.2940294956	-1.5888712443	-0.7744575852
C	-0.3032332408	-0.8815654137	1.3211635776
H	0.0506145950	-1.5310875079	-2.4301769120
H	-0.4245004527	-0.6854317317	2.3831852051
S	-2.9141595553	-1.6693562335	1.5023672593
C	-3.9703754117	-0.4180504101	0.8166687934
C	-5.6773068679	1.5538406025	-0.2327055777
C	-5.1432399178	-0.7569169138	0.1419040690
C	-3.6835864899	0.9416447618	0.9556576816
C	-4.5144282309	1.9231911000	0.4332329174
C	-5.9954324543	0.2101111199	-0.3772839357
F	-5.4744095601	-2.0349468445	-0.0359936328
F	-7.1049331226	-0.1427451395	-1.0220341846
F	-6.4845974684	2.4849531752	-0.7268639378
F	-4.2166088882	3.2116894033	0.5797726370
F	-2.5901762264	1.3310885379	1.6074456210
F	5.4948893397	3.0274824229	0.0037526616
F	3.2752387900	2.1348887860	-1.2207152485
F	7.2440633029	1.2638843865	1.1003744176
F	6.7783136121	-1.4026609823	0.9438061353
F	4.5621836812	-2.3139177489	-0.2752111184
C	-2.4453528933	-2.0862267503	-1.6057434347
H	-2.9786321492	-2.8944263251	-1.0980943203

H	-2.0933665113	-2.4485485835	-2.5735560649
H	-3.1689779266	-1.2846466132	-1.7922300572
C	2.0616689029	-0.1661755772	1.6340381947
H	1.6908588246	-0.0730579687	2.6565977897
H	2.4329689459	0.8157763043	1.3282515760
H	2.9053604452	-0.8611407855	1.6518298392

Table S11. Calculated Cartesian coordinates (Å) for the DFT minimum energy structure of **3c** with the terminal fluorobenzenoids on opposite sides of the central aryl unit.

Atom	x-coordinate	y-coordinate	z-coordinate
C	3.9999067056	-1.0137557364	-0.2962417649
C	5.9618110448	0.8714247369	0.2068492827
C	3.8573325186	0.2614838208	-0.8446474227
C	5.0831578884	-1.3483909691	0.5039071195
C	6.0699858426	-0.4016786904	0.7509833647
C	4.8604861839	1.1930082701	-0.5759529031
S	2.4826135646	0.6864339993	-1.8819932834
C	1.1278630793	0.3112666006	-0.7829122726
C	-1.1283022982	-0.3131896704	0.7831763658
C	0.8759680546	1.0005921574	0.4098023096
C	0.2379747443	-0.6886283444	-1.1805282178
C	-0.8764162103	-1.0024817979	-0.4095522209
C	-0.2383802567	0.6866535180	1.1808348740
H	0.4387577341	-1.2450659226	-2.0896294824
H	-0.4391172426	1.2430632395	2.0899645716
S	-2.4833064222	-0.6883267414	1.8820007846
C	-3.8576014141	-0.2619562225	0.8446443631
C	-6.0693303213	0.4037427883	-0.7511839802
C	-4.8607812688	-1.1929482445	0.5742248772
C	-3.9996953526	1.0140528450	0.2978923539
C	-5.0824897403	1.3499793858	-0.5023245097
C	-5.9616443662	-0.8701110608	-0.2087193298
F	-4.7825433462	-2.4289533023	1.0588610025
F	-6.9055793514	-1.7751709527	-0.4504608255
F	-7.1172029224	0.7210355143	-1.5007660304
F	-5.1928114454	2.5727861170	-1.0139046656
F	-3.0822164546	1.9496430337	0.5360343548
F	6.9057286581	1.7769804512	0.4468204487
F	4.7817186063	2.4283670243	-1.0621652171
F	7.1182935082	-0.7178182482	1.5004493472
F	5.1939262091	-2.5704951124	1.0170522127
F	3.0824666932	-1.9498324843	-0.5326124185
Br	-1.9948195368	-2.4026959299	-1.0054736335
Br	1.9942469144	2.4009859282	1.0055705454

Table S12. Calculated Cartesian coordinates (Å) for the DFT minimum energy structure of **3c** with the terminal fluorobenzenoids on same sides of the central aryl unit.

Atom	x-coordinate	y-coordinate	z-coordinate
C	-4.7988165801	0.2712648396	-0.2493374569
C	-3.4558778317	-1.9251864287	-1.2718006106
C	-3.5254957546	0.4565114549	-0.7873042228
C	-5.4020696308	-0.9791023930	-0.2210813814
C	-4.7249375475	-2.0810595076	-0.7275027358
C	-2.8781192559	-0.6637673344	-1.3095063616
S	-2.7854841271	2.0679310128	-0.8103089070
C	-1.2197240478	1.7042004145	-0.0354269773
C	1.3330670987	1.2412045617	1.0597820365
C	-1.0825582691	1.2443247718	1.2800723362
C	-0.0664145891	1.9489616552	-0.7837593822
C	1.1971417979	1.7471014859	-0.2388899633
C	0.1801053579	0.9925904967	1.8064114340
H	-0.1635867923	2.2933910607	-1.8078022427
H	0.2762150779	0.5912283681	2.8094335648
S	2.9050178574	0.9456703392	1.8517274246
C	3.5200908315	-0.3574356465	0.8167344101
C	4.5253369296	-2.4100282973	-0.8148185644
C	4.7648960939	-0.2454369921	0.1979335880
C	2.8008215908	-1.5347343430	0.6053056366
C	3.2824348163	-2.5496798425	-0.2092189822
C	5.2722700238	-1.2578805011	-0.6063672481
F	5.5028221743	0.8487828974	0.3625782686
F	6.4622191047	-1.1254914180	-1.1856396946
F	5.0002192942	-3.3806841935	-1.5849969021
F	2.5734561388	-3.6591601882	-0.3969146257
F	1.6165204096	-1.7115082236	1.1894308534
F	-2.8110061418	-2.9780587942	-1.7654797261
F	-1.6697444053	-0.5435427449	-1.8584252094
F	-5.2914139199	-3.2806348211	-0.6960512019
F	-6.6157894505	-1.1302240720	0.3014875823
F	-5.4688662623	1.2996475170	0.2640816835
Br	2.6964493894	2.1599835931	-1.3113338719
Br	-2.5770326870	0.9288819663	2.3918373787

Table S13. Calculated Cartesian coordinates (Å) for the DFT transition state structure of **3c**.

Atom	x-coordinate	y-coordinate	z-coordinate
C	4.7334179776	-1.1051326284	-0.3563115160
C	5.3307340320	1.5448418142	-0.9144692226
C	3.8132919735	-0.3415182480	-1.0752682607
C	5.9278527063	-0.5627675566	0.0947309037
C	6.2226851568	0.7665526165	-0.1853843142
C	4.1410482939	0.9853064407	-1.3568461330
S	2.3376958912	-1.0648578129	-1.7134800020
C	1.0247249754	-0.8614233298	-0.5188400973
C	-1.3842769427	-0.8030888855	0.9778450652
C	0.9799942543	-0.2556838456	0.7405110828

C	-0.1595260693	-1.4404662076	-1.0047331429
C	-1.3364794454	-1.4284737379	-0.2752584946
C	-0.2149315796	-0.2240461245	1.4603410225
H	-0.1522826779	-1.9229614761	-1.9775380249
H	-0.2367650588	0.2804025920	2.4201193826
S	-2.8266490020	-0.7642681545	2.0239640068
C	-3.8983074586	0.2424123146	1.0304184241
C	-5.6194346819	1.8166514046	-0.5333430475
C	-5.1593832329	-0.2194458094	0.6545671048
C	-3.5312251091	1.5217909794	0.6110281150
C	-4.3689439008	2.3032121072	-0.1722620714
C	-6.0191970713	0.5544220215	-0.1142604999
F	-5.5713414672	-1.4293869912	1.0230752846
F	-7.2160540637	0.0888573781	-0.4605186107
F	-6.4325586474	2.5602775834	-1.2729185718
F	-3.9910508161	3.5174453395	-0.5624032913
F	-2.3476738956	2.0265333068	0.9561714539
F	5.6239028297	2.8148320932	-1.1765267334
F	3.2942306334	1.7616149565	-2.0230556337
F	7.3620005734	1.2936369967	0.2395262042
F	6.7893316843	-1.2998606953	0.7892333503
F	4.4668489502	-2.3697823462	-0.0509074512
Br	-2.8486854410	-2.2777825866	-1.0245538681
Br	2.4475148567	0.5871788484	1.6016720203

Table S14. Calculated Cartesian coordinates (Å) for the DFT minimum energy structure of **3d** with the terminal fluorobenzenoids on opposite sides of the central aryl unit.

Atom	x-coordinate	y-coordinate	z-coordinate
C	6.8242260640	-0.8008353482	0.3729861418
C	6.3813508099	1.6520295177	-0.8370508942
C	5.8272319557	-0.7053168726	-0.5972466432
C	7.5961522529	0.2966916451	0.7365956416
C	7.3699807967	1.5257970698	0.1322984392
C	5.6314412428	0.5415026216	-1.1956709508
S	4.8598753636	-2.1164027400	-1.0677329537
C	-0.6436613311	-0.1686426241	0.6266089556
C	-3.1656937744	0.7624743394	1.4209072657
C	-1.3528851191	0.7416635641	-0.1694015915
C	-1.2217738113	-0.6046311969	1.8243092546
C	-2.4767070615	-0.1494553012	2.2193227249
C	-2.5993502342	1.2127949253	0.2263339248
H	-0.9072023420	1.1133559456	-1.0874079116
H	-0.7010414887	-1.3336763716	2.4379415495
H	-2.9322824156	-0.5133633136	3.1345586847
H	-3.1286714380	1.9426416289	-0.3794291262
S	-4.7490835850	1.3971543416	1.9677731687
C	-5.7988102586	0.6301577880	0.7590158695
C	-7.4987706761	-0.5810020099	-1.1219505875

C	-5.9555270628	-0.7564951439	0.7065775785
C	-6.5235734540	1.3908989284	-0.1578993962
C	-7.3708378924	0.8008883121	-1.0886570669
C	-6.7849856760	-1.3658846258	-0.2236267710
F	-5.2980732583	-1.5352227021	1.5631239954
F	-6.9114314983	-2.6902018032	-0.2550112469
F	-8.3031219774	-1.1532621744	-2.0098492068
F	-8.0505342343	1.5504160128	-1.9529859650
F	-6.4184259557	2.7185890697	-0.1705891811
F	6.1690718454	2.8307786111	-1.4166681894
F	4.6982678452	0.6905076371	-2.1329302148
F	8.0952134827	2.5815101013	0.4817909683
F	8.5391277291	0.1809986630	1.6684048485
F	7.0589010822	-1.9572174159	0.9904936687
C	0.6975303618	-0.6502458086	0.2135361379
C	3.2447545711	-1.5283414475	-0.5632726813
C	0.9735605861	-0.9478195581	-1.1256056695
C	1.7195119580	-0.8085591103	1.1599000340
C	2.9795963760	-1.2540945462	0.7801626106
C	2.2395048582	-1.3759691193	-1.5170111667
H	0.1889772191	-0.8515594731	-1.8700740322
H	1.5297303869	-0.5650607163	2.2013588310
H	3.7622674207	-1.3807756968	1.5225784563
H	2.4510393841	-1.5833267376	-2.5615047471

Table S15. Calculated Cartesian coordinates (Å) for the DFT minimum energy structure of **3d** with the terminal fluorobenzenoids on same sides of the central aryl unit.

Atom	x-coordinate	y-coordinate	z-coordinate
C	-6.1887184409	-0.5614648467	1.0568530659
C	-5.7387998617	-1.3783624124	-1.5511099491
C	-5.6493057607	0.3546372206	0.1543675153
C	-6.5075974640	-1.8603211733	0.6770814114
C	-6.2771435092	-2.2689850359	-0.6293233119
C	-5.4405399198	-0.0826593873	-1.1555110268
S	-5.2649501437	2.0167457942	0.6443619228
C	0.7713315440	1.9429654900	-0.0689093388
C	3.5552256203	1.8934088974	-0.3961598700
C	1.6208528352	1.8766334579	1.0442408760
C	1.3416607434	1.9910894260	-1.3460921845
C	2.7236857939	1.9617196333	-1.5134427928
C	3.0015715449	1.8604245665	0.8857487100
H	1.1968783718	1.8719743678	2.0442323071
H	0.6989772519	2.0143008030	-2.2210702179
H	3.1576234993	1.9688617820	-2.5082464769
H	3.6535826200	1.8350823268	1.7539959317
S	5.3349947988	1.8924057328	-0.6072199674
C	5.6655376548	0.2075244893	-0.1532587291
C	6.2142212697	-2.4582790878	0.5429090477

C	5.2387148622	-0.8553326339	-0.9520503218
C	6.3781306461	-0.1070719524	1.0032104832
C	6.6588106246	-1.4227614911	1.3535513485
C	5.4979210118	-2.1758152104	-0.6145329068
F	4.5569045589	-0.6173778376	-2.0705270609
F	5.0748074194	-3.1707238739	-1.3901540069
F	6.4698123551	-3.7185231886	0.8732191721
F	7.3381825493	-1.6959591962	2.4648579944
F	6.8076124017	0.8553325907	1.8170744455
F	-5.5252426764	-1.7719173335	-2.8040765060
F	-4.9350565548	0.7490155409	-2.0637678924
F	-6.5688546116	-3.5100397875	-0.9995197369
F	-7.0195601001	-2.7156648988	1.5587916679
F	-6.4047589898	-0.2156906410	2.3246791587
C	-0.7022050088	1.9585349428	0.1042712531
C	-3.4875711316	1.9713767354	0.4233993243
C	-1.3149005313	1.1525538732	1.0738920181
C	-1.5095669188	2.7734558763	-0.6972036570
C	-2.8938414821	2.7769423308	-0.5471351886
C	-2.6943807629	1.1643184363	1.2420265721
H	-0.7061308132	0.4993575567	1.6919706169
H	-1.0515924959	3.4208348889	-1.4389613828
H	-3.5148301449	3.3980760116	-1.1844070424
H	-3.1592770229	0.5441663000	2.0031224262

Table S16. Calculated Cartesian coordinates (Å) for the DFT transition state structure of **3d**.

Atom	x-coordinate	y-coordinate	z-coordinate
C	-5.8620639344	1.1518743031	1.0885003080
C	-7.0430550596	0.2661180670	-1.2578670294
C	-5.7529527111	-0.1990242285	0.7511505719
C	-6.5472893438	2.0513270403	0.2811962044
C	-7.1379346349	1.6039383294	-0.8941661228
C	-6.3560836633	-0.6185360649	-0.4368320008
S	-4.9373752262	-1.3467383027	1.8193196735
C	0.8212888244	-1.5463476924	-0.1143201944
C	3.5037035318	-1.6213958496	-0.9347789812
C	1.1536837463	-1.5978037350	-1.4727523132
C	1.8601099404	-1.5423840882	0.8271635383
C	3.1891558355	-1.5929014763	0.4253636650
C	2.4835109626	-1.6235789763	-1.8845171135
H	0.3641356274	-1.6241969344	-2.2178140339
H	1.6247104062	-1.4828434912	1.8860060458
H	3.9840991424	-1.5984940607	1.1656174541
H	2.7303805413	-1.6371206998	-2.9416387603
S	5.2067552490	-1.7033197270	-1.4813458913
C	5.8284866077	-0.2430952495	-0.6904542670
C	6.8165893718	2.0661984935	0.5685479108
C	6.8760960790	-0.3105748151	0.2274729049
C	5.3001288037	1.0199059307	-0.9687349793

C	5.7727456387	2.1649088192	-0.3444064106
C	7.3746588841	0.8271804108	0.8516893621
F	7.4272183870	-1.4824203641	0.5392260506
F	8.3704904706	0.7347810616	1.7296394169
F	7.2767586941	3.1562167558	1.1712920276
F	5.2420374051	3.3542971313	-0.6170563160
F	4.3091515751	1.1483507889	-1.8476538765
F	-7.6091324954	-0.1543711495	-2.3855400985
F	-6.2737406150	-1.8875723034	-0.8224105996
F	-7.7900836826	2.4560554387	-1.6735128475
F	-6.6443931658	3.3328874868	0.6230262694
F	-5.3016311921	1.6177443578	2.1991659201
C	-0.5938527036	-1.4863669657	0.3246988239
C	-3.2721889886	-1.3446946013	1.1762188178
C	-1.5319377668	-0.7168376267	-0.3700417333
C	-1.0272386456	-2.1892441675	1.4565122233
C	-2.3473370931	-2.1215633823	1.8810892922
C	-2.8592828389	-0.6435110307	0.0444795767
H	-1.2189153577	-0.1424082960	-1.2370522562
H	-0.3269268592	-2.8148706866	2.0020449380
H	-2.6605605497	-2.6776161270	2.7601211873
H	-3.5571027107	-0.0284217381	-0.5139943193

Table S17. Calculated Cartesian coordinates (Å) for the DFT minimum energy structure of the C_6F_6…NMe_4F association complex.

Atom	x-coordinate	y-coordinate	z-coordinate
N	3.1690367801	-0.4398084878	-0.5759393342
C	2.4058010754	0.7540929345	-1.0759291458
H	1.7043325639	1.0086111246	-0.2665799524
H	1.8939923777	0.4705434911	-1.9982433805
H	3.1216859817	1.5548018911	-1.2719846931
C	2.2049205692	-1.5638747850	-0.3224929443
H	1.5169045492	-1.1881894057	0.4495347155
H	2.7762185860	-2.4298895987	0.0178786544
H	1.6939847199	-1.7933921280	-1.2600167708
C	3.8227169358	-0.0738629703	0.7256306453
H	3.0026047189	0.2149353989	1.3997356387
H	4.5140817330	0.7499524185	0.5378347951
H	4.3677935252	-0.9480698498	1.0872720412
C	4.1877414193	-0.8528331753	-1.5737895883
H	4.7265406832	-1.7219604133	-1.1929912527
H	4.8808926960	-0.0261829195	-1.7370838187
H	3.6845268713	-1.1070999084	-2.5081101518
F	1.1008407641	0.3824902717	1.3825307769
C	-1.0072841390	1.1786736176	-0.7742191195
C	-1.6431007646	-0.5884544058	1.2668851624
C	-1.4440971241	1.6624536529	0.4491777725
C	-0.8902035622	-0.1874200442	-0.9757409803

C	-1.2090565835	-1.0714452954	0.0423153626
C	-1.7602057579	0.7781789929	1.4696294757
F	-0.6607595487	2.0234211086	-1.7503691564
F	-1.5382904158	2.9746896898	0.6483615958
F	-2.1560302527	1.2418272985	2.6493736883
F	-1.9215826491	-1.4354375051	2.2531982248
F	-1.0533940808	-2.3849376259	-0.1477849539
F	-0.4264248694	-0.6517182332	-2.1465607468

Table S18. Calculated Cartesian coordinates (Å) for the DFT minimum energy structure of **1a**-*conformer2*.

Atom	x-coordinate	y-coordinate	z-coordinate
C	-5.7310010541	-3.3130045160	0.4392610715
C	-5.4821499101	-4.4216540163	-0.3668489657
C	-4.1680895035	-4.8026882052	-0.6371302904
C	-3.1111441047	-4.0717902385	-0.1001368117
C	-3.3615860048	-2.9832827116	0.7397270794
C	-4.6698099979	-2.6070909336	1.0119540407
C	-0.8716657231	-2.9852540207	-1.1393321980
C	0.2327851059	-2.3257804497	-0.6024572209
C	0.6474481609	-1.0910404996	-1.0801601345
C	-0.0091948465	-0.4598415389	-2.1367407958
C	-1.1043015232	-1.1276912476	-2.6870588562
C	-1.5204760436	-2.3600684331	-2.2050437383
C	2.1374779987	1.1562643268	-2.8271847006
C	2.8503164770	0.0474186606	-3.2892699392
C	4.2331894964	0.1218889997	-3.4126700395
C	4.9040665459	1.3071452038	-3.0971842627
C	4.1858742370	2.4238629812	-2.6721150870
C	2.8026563416	2.3450326552	-2.5239161830
C	7.0410259275	0.6315599857	-1.6373848764
C	6.7715153631	1.3114532860	-0.4480090150
C	6.9467008902	0.7078155324	0.7858251997
C	7.3985010489	-0.6097534756	0.8910296800
C	7.7147307888	-1.2731471671	-0.2919969998
C	7.5363632547	-0.6655565500	-1.5312724135
C	5.7230026967	-1.2952284810	2.8611334085
C	4.7540270308	-1.6401316512	1.9146195873
C	3.4065335171	-1.5040142027	2.2187421447
C	3.0238391665	-1.0296559080	3.4754937408
C	3.9867056074	-0.7169499934	4.4325164315
C	5.3409329406	-0.8475124283	4.1242129434
C	0.8423078378	0.4040407713	2.7057892530
C	1.4822798592	1.6433606189	2.6759743266
C	1.0890214784	2.6418282748	1.7981051704
C	0.0274452718	2.4676026577	0.9074822961
C	-0.5921260764	1.2157833917	0.9119460522
C	-0.2040968994	0.2237281452	1.8034930383
C	-2.1914786887	3.6623165413	-0.2031405157

C	-2.9508383077	3.5878209291	0.9677581168
C	-4.3351100071	3.5024805104	0.8852317480
C	-4.9635874211	3.5110194818	-0.3643690082
C	-4.2059424253	3.6136537940	-1.5290949539
C	-2.8152749881	3.6810190215	-1.4490603873
C	-6.9582237182	1.6954973367	-0.1426231105
C	-7.5848239760	1.2361468135	1.0132031142
C	-7.7327171614	-0.1238234264	1.2660711036
C	-7.2652904116	-1.0856328184	0.3728819108
C	-6.6699888008	-0.6232855523	-0.8045795139
C	-6.5112496840	0.7310082621	-1.0482952673
F	0.9002641265	-2.8556276128	0.4255529240
F	1.6853643982	-0.4960650533	-0.4776902820
F	-1.7784527143	-0.5805720891	-3.6991183239
F	-2.5674231252	-2.9441092901	-2.7834628592
F	6.3106423752	2.5624030749	-0.4816507435
F	6.6379971762	1.3971016501	1.8826543449
F	8.1476299005	-2.5340098498	-0.2627254188
F	7.8059275421	-1.3782708316	-2.6256680100
F	2.4806172574	1.8994012004	3.5192521346
F	1.7298299051	3.8118669515	1.8376968388
F	-1.5868893486	0.9396537509	0.0610240309
F	-0.8545825130	-0.9460571395	1.7587758711
F	-8.0383816409	2.0969329896	1.9252162923
F	-8.3162929252	-0.4934360613	2.4072391702
F	-6.2292203656	-1.4889463848	-1.7165145063
F	-5.9165414754	1.1051534103	-2.1814108661
H	4.7107239930	3.3402010969	-2.4246030531
H	5.0522149222	-2.0117329805	0.9384054495
H	4.7974922257	-0.7393068059	-3.7579550910
H	2.6517113652	-1.7699956584	1.4871192318
H	2.3305562856	-0.8700249976	-3.5494846636
H	-2.4611058254	3.6006977638	1.9372393041
H	-4.9348646547	3.4463797119	1.7888906881
H	-4.8654806635	-1.7599511123	1.6627792160
H	6.0951853806	-0.5615401565	4.8501352033
H	2.2448622631	3.2027739664	-2.1584948600
H	-2.2180457341	3.7228174734	-2.3544564280
H	3.6821262784	-0.3317752770	5.4004113637
H	-2.5347453700	-2.4313193911	1.1743627482
H	-3.9687294082	-5.6351390797	-1.3040285335
H	-6.3092820007	-4.9551150019	-0.8240866624
H	-4.6998264135	3.6081985587	-2.4951188609
S	-7.4235766894	-2.8134602799	0.7313913666
S	-6.7525566991	3.4305548533	-0.4634167898
S	-0.4141217034	3.8186755126	-0.1452505580
S	1.2853037362	-0.8589250643	3.8693861045
S	7.4652365618	-1.4266708821	2.4635768487
S	6.6942786682	1.3752867610	-3.2159840278
S	0.3615275693	1.1746905144	-2.6858676291

S	-1.4258317278	-4.5376058918	-0.4870189948

Table S19. Calculated Cartesian coordinates (Å) for the DFT minimum energy structure of **1a**-*conformer3*.

Atom	x-coordinate	y-coordinate	z-coordinate
C	-6.3053512042	-1.6752120598	-0.0229385780
C	-5.1251720810	-1.3993871860	-0.7225798031
C	-4.9380341029	-0.1536591828	-1.3069105003
C	-5.9336480146	0.8213530221	-1.1970746181
C	-7.1228858218	0.5375002980	-0.5293544793
C	-7.3084715831	-0.7124753985	0.0625368125
C	-4.2987835916	3.0017230879	-1.0456671253
C	-3.1777808742	3.4950571866	-1.7122919155
C	-2.0900890055	4.0211000390	-1.0192787377
C	-2.0698505302	4.0647708188	0.3733781219
C	-3.1911532525	3.5654248803	1.0406849259
C	-4.2810361388	3.0559448950	0.3516471670
C	0.0523731565	3.2963697943	1.9192116104
C	-0.2266742469	2.8861133594	3.2232080711
C	0.4289966351	1.7808089739	3.7572359374
C	1.3618376602	1.0820016907	2.9852556307
C	1.6294330977	1.4799446114	1.6754033471
C	0.9798394723	2.5925641941	1.1490093907
C	3.5000865005	-0.7078546071	2.7969399613
C	4.5219192989	0.2184065177	2.5761232764
C	5.6732554609	-0.1269205274	1.8807616866
C	5.8748338602	-1.4233550138	1.4030531291
C	4.8622104111	-2.3507020840	1.6396749961
C	3.6981574777	-2.0005852333	2.3112270690
C	6.7643212752	-1.5137672145	-1.1290135783
C	7.2125155816	-0.3713936926	-1.7901000369
C	6.6859116507	-0.0431967580	-3.0395174179
C	5.7133166952	-0.8582446038	-3.6139334932
C	5.3051685028	-2.0329396358	-2.9759600047
C	5.8329599165	-2.3637186604	-1.7335917781
C	3.3548772828	0.0503122158	-4.4450714613
C	2.1883635059	-0.6289163485	-4.7896050544
C	0.9737423292	-0.3403139749	-4.1736057356
C	0.8770347745	0.6371026695	-3.1840572653
C	2.0434015730	1.3302557109	-2.8552000912
C	3.2482922495	1.0540143717	-3.4796954653
C	-0.5282268882	0.1398770633	-0.8594216054
C	-1.4509395432	0.5202764912	0.1215307704
C	-1.4827745126	-0.1361475109	1.3425563246
C	-0.6107722537	-1.2020880969	1.5878832266
C	0.3224984840	-1.5688404848	0.6208768958
C	0.3703972243	-0.8938277664	-0.6025123343
C	-2.4147263745	-2.6980321610	2.7438661979

C	-3.5354588942	-2.0135486820	3.2104455152
C	-4.7977219891	-2.2644528413	2.6926783497
C	-4.9919728821	-3.2074753877	1.6830941406
C	-3.8875344663	-3.9571607294	1.2836192531
C	-2.6246673333	-3.7061045227	1.8046600380
F	-3.1270140230	3.4764667051	-3.0444660913
F	-1.0607967264	4.4801266263	-1.7273640686
F	-3.2396305179	3.5831675014	2.3739713845
F	-5.3339879508	2.6324076347	1.0505514329
F	4.4002039474	1.4620937257	3.0359469231
F	6.5966606416	0.8123690996	1.6871185706
F	4.9770889651	-3.6055485603	1.2010533635
F	2.7660818028	-2.9388744436	2.4897353235
F	2.2180979902	-1.6000024474	-5.7016071799
F	-0.0955294608	-1.0460945814	-4.5327066135
F	2.0227096320	2.2793695086	-1.9194670255
F	4.3215274451	1.7601052181	-3.1252485532
F	-3.4049563203	-1.0334313236	4.1065615180
F	-5.8158167779	-1.5156016535	3.1135041365
F	-4.0029743149	-4.8701938243	0.3190256837
F	-1.5926429554	-4.3913801879	1.3129776666
H	2.3349326003	0.9315683368	1.0582236876
H	7.9367163123	0.2797987191	-1.3116501641
H	0.2196728686	1.4670011147	4.7760547550
H	6.9889670787	0.8703200083	-3.5401893545
H	-0.9483365039	3.4347406213	3.8192082552
H	-2.1397293815	1.3377777476	-0.0701569968
H	-2.1916484447	0.1759982109	2.1052121886
H	-8.2132241743	-0.9204726049	0.6244223289
H	5.5202855486	-3.2712658340	-1.2269587878
H	1.1862206210	2.9094476918	0.1315560304
H	1.0903271717	-1.2016675585	-1.3547803293
H	4.5693298575	-2.6765557951	-3.4493444873
H	-7.8835090738	1.3051615308	-0.4313560589
H	-4.0236005247	0.0596862498	-1.8549315069
H	-4.3527659793	-2.1575462605	-0.8128644411
H	0.9882391284	-2.4060004466	0.8078710710
S	-6.5258499312	-3.2627747669	0.7889000712
S	-0.7638243587	-2.1368611558	3.1170396536
S	-0.6638556134	1.0214298079	-2.4055024339
S	4.9276452785	-0.3754668506	-5.1543959415
S	7.3580120365	-1.8733779559	0.5266993530
S	2.0707647132	-0.3520095932	3.7698483973
S	-0.7046832790	4.7804345398	1.2575658773
S	-5.7080791380	2.4262553405	-1.9553090648

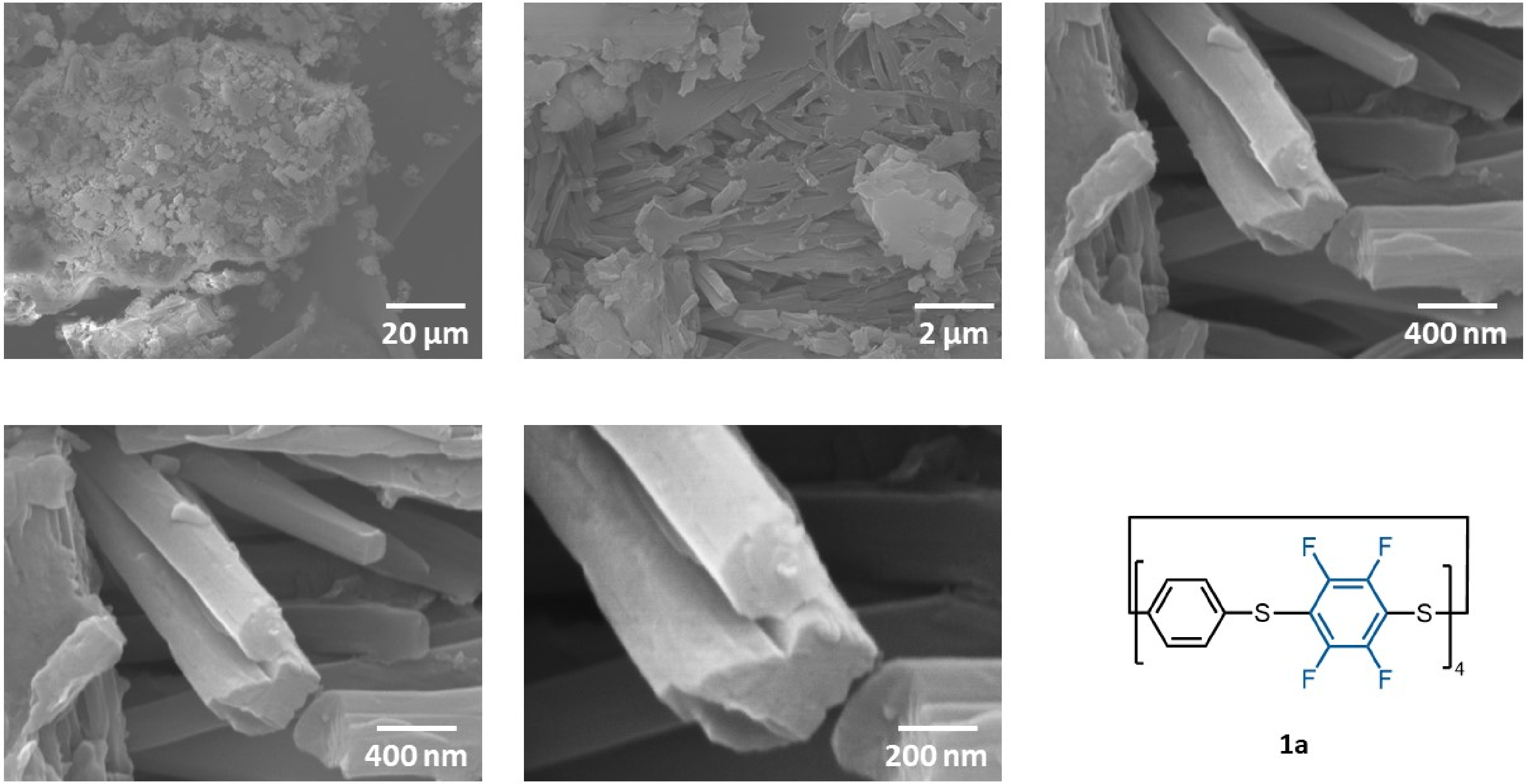

1a

9. Comparison of Macrocycle (1a) to Polymeric P1

Figure S221. SEM images of macrocycle **1a**.

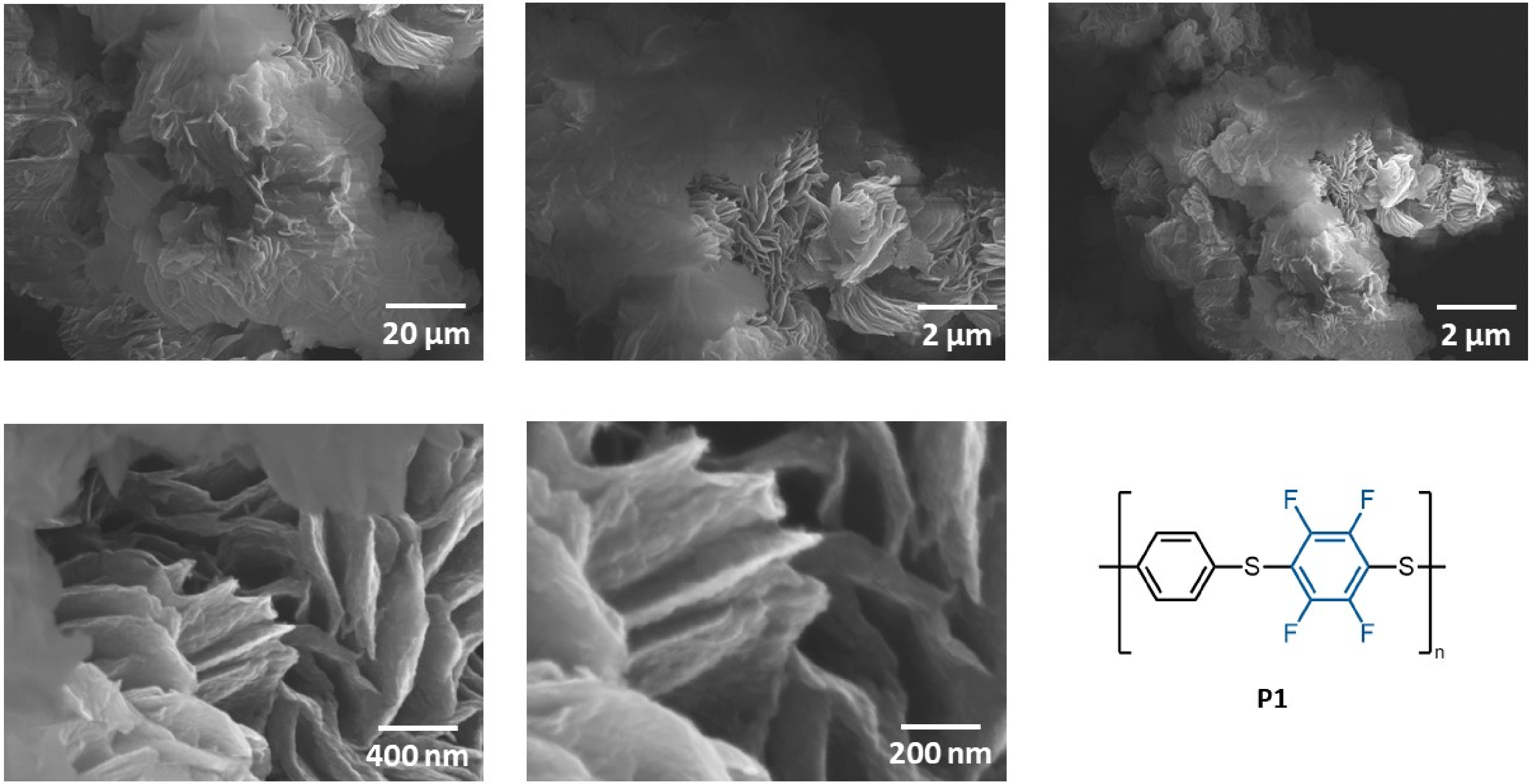

Figure S222. SEM images of insoluble polymer **P1** that forms during the synthesis of **1a**.

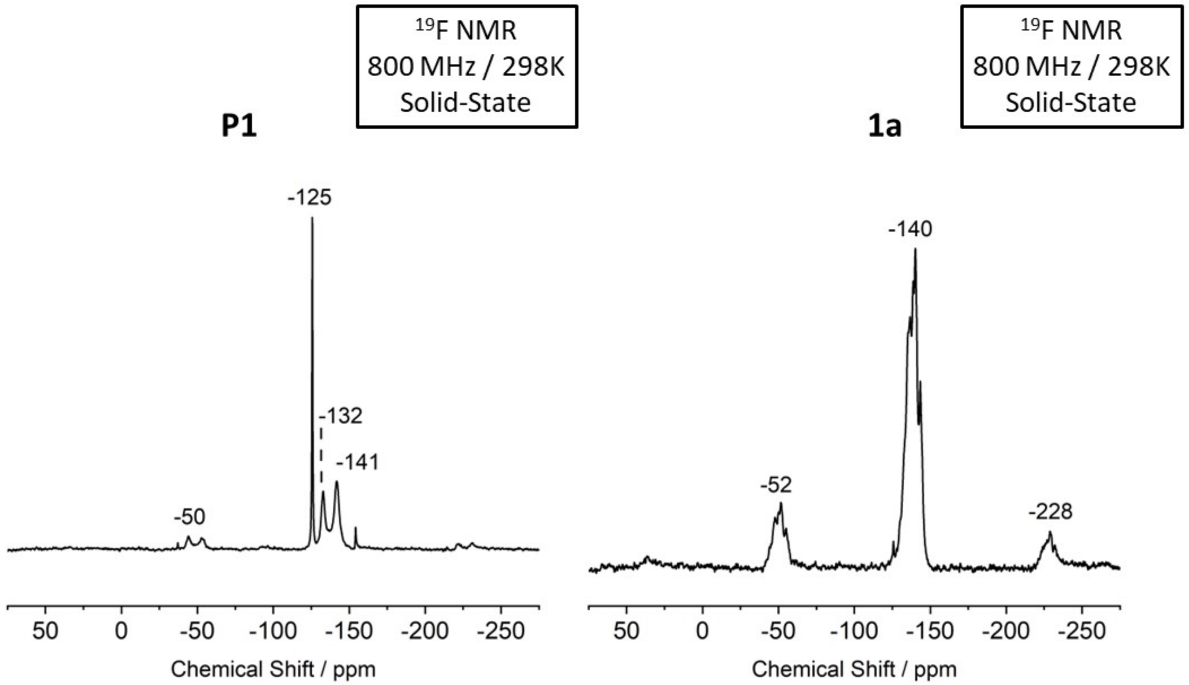

Comparison Between P1 and 1a: Solid State ^{19}F NMR

Figure 223. Comparison of solid-state ^{19}F NMR spectrum of **P1** and **1a**.

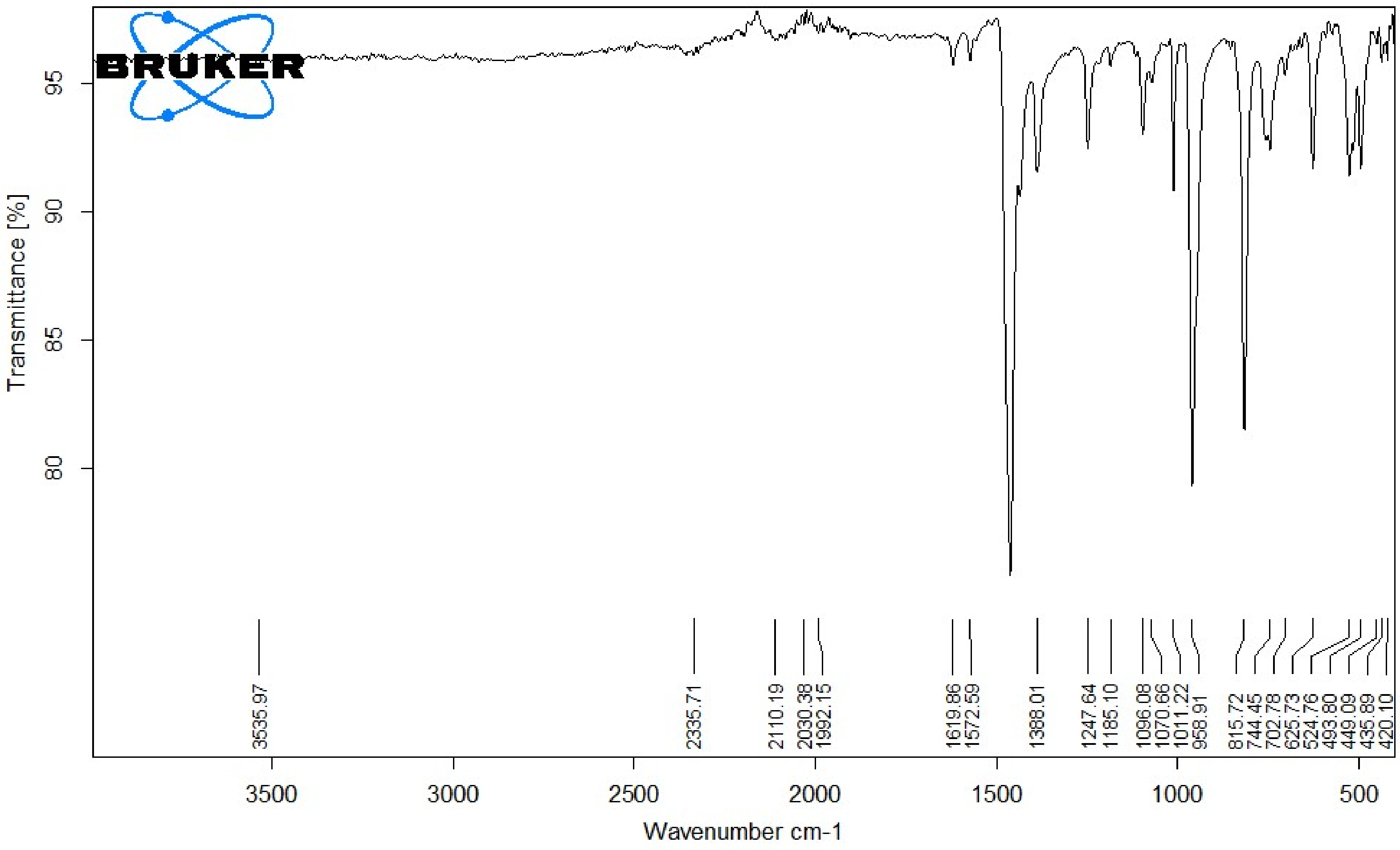

Figure 224. Infra-red spectrum of **1a**.

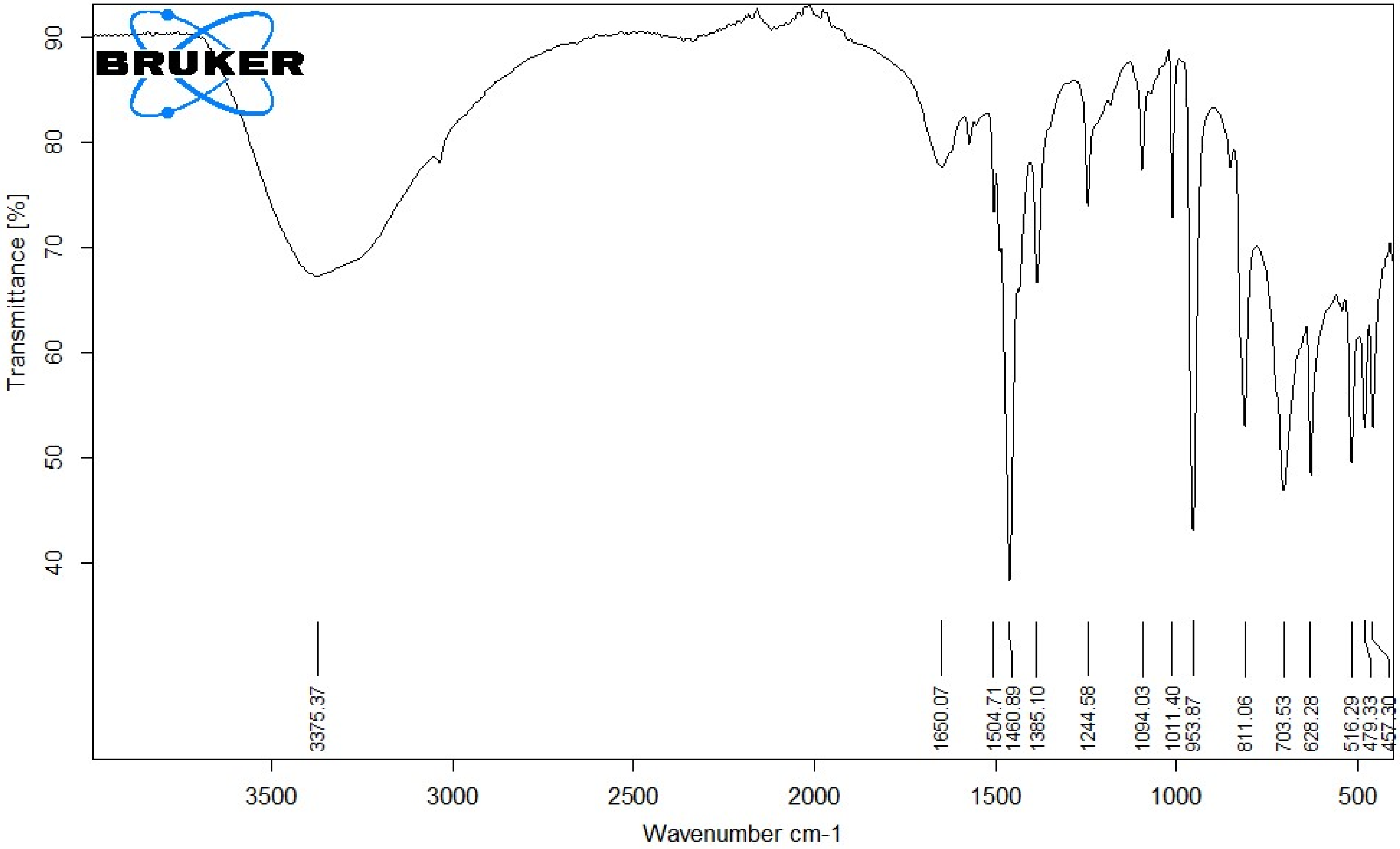

Figure 225. Infra-red spectrum of **P1**.

10. Thermogravimetric Analysis (TGA) of 1a

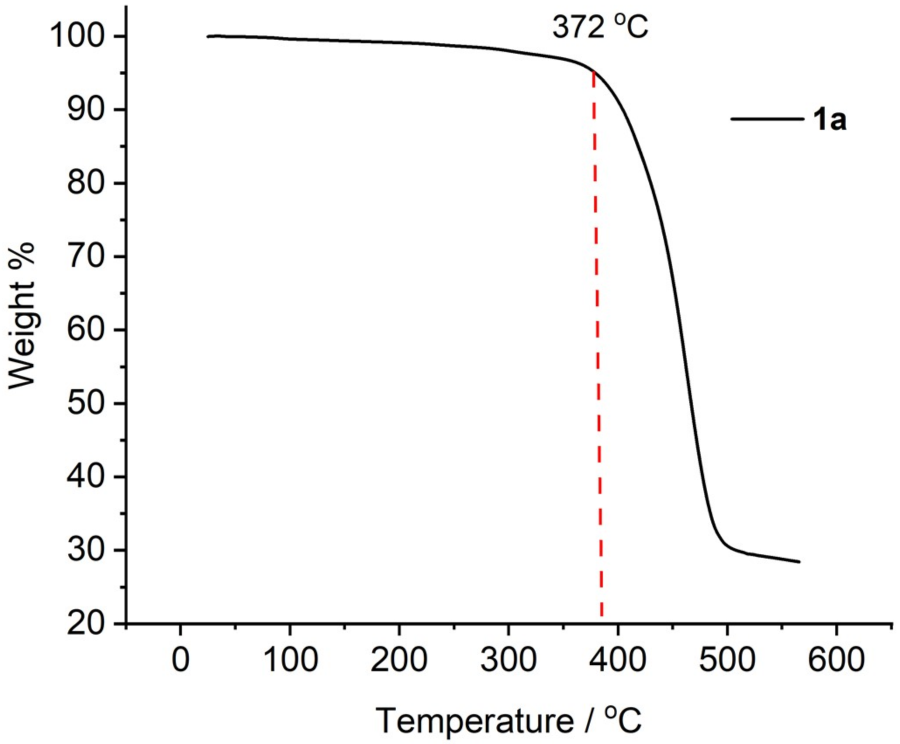

Figure S226. Thermogravimetric analysis of **1a**. Ramp 10.00 °C/min to 550 °C.

11. References

1. J. Cosier and A. M. Glazer, *J. Appl. Crystallogr.*, 1986, **19**, 105.

2. D. R. Allan, H. Nowell, S. A. Barnett, M. R. Warren, A. Wilcox, J. Christensen, L. K. Saunders, A. Peach, M. T. Hooper, L. Zaja, S. Patel, L. Cahill, R. Marshall, S. Trimnell, A. J. Foster, T. Bates, S. Lay, M. A. Williams, P. V. Hathaway, G. Winter, M. Gerstel and R. W. Wooley, *Crystals*., 2017, **7**, 22.

3. Rigaku Oxford Diffraction, (2018), CrysAlisPro Software system, version 1.171.40.45a, Rigaku Corporation, Oxford, UK.

4. G. Winter, D. G. Waterman, J. M. Parkhurst, A. S. Brewster, R. J. Gildea, M. Gerstel, L. Fuentes-Montero, M. Vollmar, T. Michels-Clark, I. D. Young, N. K. Sauter and G. Evans, *Acta Crystallogr. Sect. D-Struct. Biol.*, 2018, **74**, 85.

5. AIMLESS (CCP4: Supported Program): Journal, 2018, CCP4 7.0.062: AIMLESS, version 060.067.062: 027/005/018.

6. O. V. Dolomanov, L. J. Bourhis, R. J. Gildea, J. A. K. Howard and H. Puschmann, *J. Appl. Cryst.*, 2009, **42**, 339.

7. G. M. Sheldrick, *Acta Crystallogr. A.*, 2015, **71**, 3.

8. G. M. Sheldrick, *Acta Crystallogr. C.*, 2015, **71**, 3.

9. The facility "CheckCIF," can be found at http://checkcif.iucr.org.

10. M. S. Newman and H. A. Karnes, *J. Org. Chem*., 1966, **31**, 3980.

11. F. Maiolo, L. Testaferri, M. Tiecco and M. Tingoli, *J. Org. Chem*., 1981, **46**, 3070.

12. J. S. Anderson, E. Elkaim, A. S. Filatov, N. E. Horwitz, R. J. Papoular, O. Salinas and J. Xie, *Dalton Trans.*, 2021, **50**, 10798.

13. C. A. Carpenter, S. E. Wegwerth and C. J. Douglas, *Org. Prep. Proced. Int.*, 2019, **51**, 498.

14. S. Pallaud, *Comp. Rend., Ser. C*, 1967, **264**, 2163.

15. T. W. T. Muesmann, M. S. Wickleder and J. Christoffers, *Synthesis*, 2011, 2775.

16. M. E. Peach, *Can. J. Chem*., 1968, **46**, 2699.

17. L. J. Johnston and M. E. Peach, *J. Fluor. Chem.*, 1979, **13**, 41.

18. Y. Zou, A. M. Spokoyny, C. Zhang, M. D. Simon, H. Yu, Y.-S. Lin, B. L. Pentelute, *Org. Biomol. Chem*., 2014, **12**, 566.

19. P. Thordarson, P. *Chem. Soc. Rev.*, 2011, **40**, 1305.

20. **R.** Nouch, S. Woodward, S. D. Willcox, D. Robinson and W. Lewis, *Organometallics*, 2020, **39**, 834.

21. E. J. Billo, *Excel for Chemists: A Comprehensive Guide*, 3rd Ed., John Wiley & Sons, Inc., New York, 2011.

22. J. H. Espenson, *Chemical Kinetics and Reaction Mechanisms*, 2nd Ed., McGraw-Hill, Inc., New York, 1995.

23. For example, fits to both first order (R^2 = 0.97) and second order equal concentrations (R^2 <0.99) behaviour were inferior.

24. See pages 34 and 60–61 of reference **22**.

25. K. O. Christe, W. W. Wilson, R. D. Wilson, R. Bau, and J. A. Feng, *J. Am. Chem. Soc*., 1990, **112**, 7619.

26. Preparation of $PhSC_6F_5$ (**7**) and its substitution product is described in: Z. Liu, K. Ouyang and N. Yang, *Org. Biomol. Chem*., 2018, **16**, 988. Our data were concordant.

27. E. Epifanovsky, A. T. B. Gilbert, X. Feng, J. Lee, Y. Mao, N. Mardirossian, P. Pokhilko, A. F. White, M. P. Coons, A. L. Dempwolff, Z. Gan, D. Hait, P. R. Horn, L. D. Jacobson, I. Kaliman, J. Kussmann, A. W. Lange, K. U. Lao, D. S. Levine, J. Liu, S. C. McKenzie, A. F. Morrison, K. D. Nanda, F. Plasser, D. R. Rehn, M. L. Vidal, Z.-Q. You, Y. Zhu, B. Alam, B. J. Albrecht, A. Aldossary, E. Alguire, J. H. Andersen, V. Athavale, D. Barton, K. Begam, A. Behn, N. Bellonzi, Y. A. Bernard, E. J. Berquist, H. G. A. Burton, A. Carreras, K. Carter-Fenk, R. Chakraborty, A. D. Chien, K. D. Closser, V. Cofer-Shabica, T. Friedhoff, J. Gayvert, Q. Ge, G. Gidofalvi, M. Goldey, J. Gomes, C. E. González-Espinoza, S. Gulania, A. O. Gunina, M. W. D. Hanson-Heine, P. H. P. Harbach, A. Hauser, M. F. Herbst, M. Hernández Vera, M. Hodecker, Z. C. Holden, S. Houck, X. Huang, K. Hui, B. C. Huynh, M. Ivanov, Á. Jász, H. Ji, H. Jiang, B. Kaduk, S. Kähler, K. Khistyaev, J. Kim, G. Kis, P. Klunzinger, Z. Koczor-Benda, J. H. Koh, D. Kosenkov, L. Koulias, T. Kowalczyk, C. M. Krauter, K. Kue, A. Kunitsa, T. Kus, I. Ladjánszki, A. Landau, K. V. Lawler, D. Lefrancois, S. Lehtola, R. R. Li, Y.-P. Li, J. Liang, M. Liebenthal, H.-H. Lin, Y.-S. Lin, F. Liu, K.Y. Liu, M. Loipersberger, A. Luenser, A. Manjanath, P. Manohar, E. Mansoor, S. F. Manzer, S.-P. Mao, A. V. Marenich, T. Markovich, S. Mason, S. A. Maurer, P. F. McLaughlin, M. F. S. J. Menger, J.-M. Mewes, S. A. Mewes, P. Morgante, J. W. Mullinax, K. J. Oosterbaan, G. Paran, A. Paul, A. S. K. Paul, F. Pavošević, Z. Pei, S. Prager, E. I. Proynov, Á. Rák, E. Ramos-Cordoba, B. Rana, A. E. Rask, A. Rettig, R. M. Richard, F. Rob, E. Rossomme, T. Scheele, M. Scheurer, M. Schneider, N. Sergueev, S. M. Sharada, W. Skomorowski, D. W. Small, C. J. Stein, Y.-C. Su, E. J. Sundstrom, Z. Tao, J. Thirman, G. J. Tornai, T. Tsuchimochi, N. M. Tubman, S. P. Veccham, O. Vydrov, J. Wenzel, J. Witte, A. Yamada, K. Yao, S. Yeganeh, S. R. Yost, A. Zech, I. Y. Zhang, X. Zhang, Y. Zhang, D. Zuev, A. Aspuru-Guzik, A. T. Bell, N. A. Besley, K. B. Bravaya, B. R. Brooks, D. Casanova, J.-D. Chai, S. Coriani, C. J. Cramer, G. Cserey, A. E. DePrince, R. A. DiStasio, A. Dreuw, B. Dunietz, T. R. Furlani, W. A. Goddard, S. Hammes-Schiffer, T. Head-Gordon, W. J. Hehre, C.-P. Hsu, T.-C. Jagau, Y. Jung, A. Klamt, J. Kong, D. S. Lambrecht, W. Liang, N. J. Mayhall, C. W. McCurdy, J. B. Neaton, C. Ochsenfeld, J. A. Parkhill, R. Peverati, V. A. Rassolov, Y. Shao, L. V. Slipchenko, T. Stauch, R. P. Steele, J. E. Subotnik, A. J. W. Thom, A. Tkatchenko, D. G. Truhlar, T. Van Voorhis, T. A. Wesolowski, K. B. Whaley, H. L. Woodcock, P. M. Zimmerman, S. Faraji, P. M. W. Gill, M. Head-Gordon, J. M. Herbert, and A. I. Krylov, A. I., *J. Chem. Phys*., 2021, **155**, 084801.

28. A. E. Reed, L. A. Curtiss, and F. Weinhold, *Chem. Rev*., 1988, **88**, 899.

29. T. A. J. Halgren, *Comput. Chem*., 1996, **17**, 490.

30. Y. Zhao and D. G. Truhlar, *Theor. Chem. Acc*., 2008, **120**, 215.

31. A. Klamt and G. Schüürmann, *J. Chem. Soc., Perkin Trans. 2*., 1993, **5**, 799.

32. M. A. L. Marques and E. K. U. Gross, *Annu. Rev. Phys. Chem.*, 2004, **55**, 427.

33. E. Weinn, W. Ren, and E. Vanden-Eijnden, *Phys. Rev. B.,* 2002, **66**, 052301.
